# Supplementary material for: Photocatalyzed regioselective hydrosilylation for the divergent synthesis of geminal and vicinal borosilanes
Source: Nat Commun. 2023 May 2;14:2525. doi: 10.1038/s41467-023-38224-y (PMC10154379; doi:10.1038/s41467-023-38224-y)
Supplement: Supplementary file 1 — Supplementary Information [file 41467_2023_38224_MOESM1_ESM.pdf]

Supplementary Information

Photocatalyzed Regioselective Hydrosilylation for the Divergent Synthesis of Geminal and Vicinal Borosilanes

Kong et al.

Supplementary Methods

General Information

All chemicals and anhydrous solvents were purchased from commercial suppliers and used as received. Commercially unavailable substrates were synthesized according to the literature. ^1H NMR, ^{13}C NMR, ^{19}F NMR, ^{11}B NMR, and ^{29}Si NMR spectra were recorded on a Bruker AV-III400 (400 MHZ) or AMX500 (500 MHz) spectrometer. Chemical shifts were calibrated using residual undeuterated solvent as an internal reference ($CDCl_3$: 7.26 ppm ^1H NMR, 77.16 ppm ^{13}C NMR). Multiplicity was indicated as follows: s (singlet), d (doublet), t (triplet), q (quartet), m (multiplet), dd (doublet of doublet), brs (broad singlet). High-resolution mass spectra (HRMS) were obtained on a Finnigan/MAT 95XL-T spectrometer. GC analysis was performed on Aglilent 7820A & 5977E GC-MS. Cyclic voltammograms (CV) were collected using a VersaSTAT 3 Potentiostat Galvanostat from Princeton Applied Research. UV-vis absorption spectra and emission spectra were taken at ambient temperature using an Edinburgh FS5 spectrofluorometer. IR spectra were recorded on a bruker alpha FT-IR. Absorption maxima (ν_{max}) are reported in wavenumbers (cm^{-1}). All catalytic reactions were carried out in a microwave tube (10 mL) under an argon atmosphere with magnetic stirring. 18 W blue LED strips (2-meter, maximum emission at around 470 nm) were purchased from Inwares Pte Ltd (Singapore). 40 W 456 nm LED light was purchased from Kessil. Spectral output can be found on: https://www.kessil.com/science/PR160L.php The Asia Syringe Pump was purchased from Syrris Company (UK) for continuous flow setup. The Tefzel shut-off valves, and HPFA micro tubings were purchased from IDEX Health & Science (Oak Harbor, WA). Visualization was achieved by short wave (254 nm) ultraviolet light or by staining with iodine (I_2).

Commercially available alkenyl or allyl boronates and silanes were purchased from BLD Pharmatech Ltd., Oakwood Products Inc and Sigma-Aldrich Pty Ltd.

Preparation of Starting Materials

Commercially unavailable alkenyl and allyl boronates were prepared according to reported procedures[1-7]. The spectra data of these substrates are in accordance with the literature.

Alkenyl boronates **S1-S5** were synthesized according to the following known procedure[5].

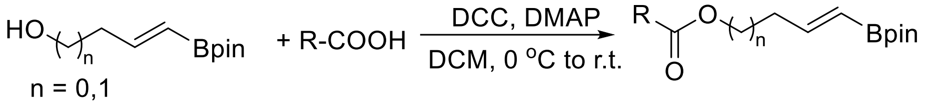

A tube equipped with a stirring bar was charged with alcohol (3.00 mmol, 1.00 equiv.), acid (4.5 mmol, 1.5 equiv.), and DCM (30 mL). The mixture was cooled to 0°C and DMAP (146.6 mg, 1.20 mmol, 0.20 equiv.) and *N, N'*-dicyclohexylcarbodiimide (2.48 g, 12.0 mmol, 2.00 equiv.) were added sequentially. The reaction was allowed to warm to room temperature and stirred for 12 hrs. The mixture was diluted with DCM (20 mL) and washed with 10% citric acid solution (20 mL) and brine (20 mL). The organic layer was dried ($MgSO_4$) and evaporated. Purification by flash column chromatography on silica gel (eluent: *n*-hexane/EtOAc mixtures) gave the desired product.

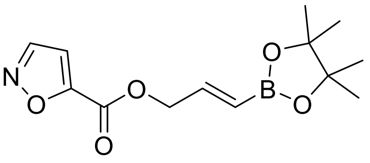

(*E*)-3-(4,4,5,5-Tetramethyl-1,3,2-dioxaborolan-2-yl)allyl isoxazole-5-carboxylate (S1). Colorless oil (77%, 0.64 g, eluent: hexane/EA = 10:1, R_f = 0.2). ^{1}H NMR (500 MHz, $CDCl_3$) δ 8.94 (s, 1H), 8.52 (s, 1H), 6.66 (dt, *J* = 18.1, 4.6 Hz, 1H), 5.74 (d, *J* = 18.2 Hz, 1H), 4.89 (dd, *J* = 4.7, 1.8 Hz, 2H), 1.26 (s, 12H). ^{13}C NMR (126 MHz, $CDCl_3$) δ 160.69, 158.26, 149.14, 144.94, 129.40, 83.56, 66.44, 24.77. The carbon signal attached to B was not observed. ^{11}B NMR (160 MHz, $CDCl_3$) δ 29.31. IR ν_{max} (DCM):

2979, 2933, 1721, 1647, 1513, 1349, 1256, 1099 cm^{-1}. HR-MS (APCI) calcd for $C_{13}H_{19}BNO_5$ [M+H]$^+$: 280.1351, found 280.1347.

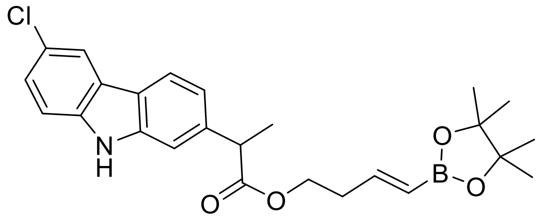

(*E*)-4-(4,4,5,5-Tetramethyl-1,3,2-dioxaborolan-2-yl)but-3-en-1-yl 2-(6-chloro-9H-carbazol-2-yl)propanoate (S2). Sticky oil (73%, 0.99 g, eluent: hexane/EA = 5:1, R_f = 0.4). ^{1}H NMR (400 MHz, $CDCl_3$) δ 8.66 (s, 1H), 7.93 – 7.84 (m, 3H), 7.26 – 7.19 (m, 2H), 7.09 (dd, *J* = 8.1, 1.5 Hz, 1H), 6.53 (dt, *J* = 18.0, 6.5 Hz, 1H), 5.44 (dt, *J* = 18.0, 1.5 Hz, 1H), 4.19 – 4.11 (m, 2H), 3.80 (q, *J* = 7.9 Hz, 1H), 2.37 (qd, *J* = 6.4, 1.5 Hz, 2H), 1.50 (d, *J* = 7.1 Hz, 3H), 1.24 (s, 12H). ^{13}C NMR (101 MHz, $CDCl_3$) δ 174.65, 149.48, 140.46, 139.07, 138.24, 125.71, 124.72, 124.33, 121.60, 120.53, 119.97, 119.58, 111.55, 109.81, 83.50, 63.14, 46.02, 35.05, 24.84, 18.84. The carbon signal attached to B was not observed. ^{11}B NMR (128 MHz, $CDCl_3$) δ 29.75. IR ν_{max} (DCM): 2963, 1727, 1640, 1413, 1261, 1092, 1028 cm^{-1}. HR-MS (APCI) calcd for $C_{25}H_{30}BClNO_4$ [M+H]$^+$: 454.1951, found 454.1946.

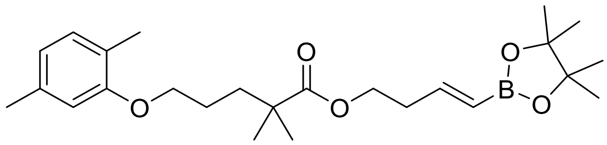

(*E*)-4-(4,4,5,5-Tetramethyl-1,3,2-dioxaborolan-2-yl)but-3-en-1-yl 5-(2,5-dimethylphenoxy)-2,2-dimethylpentanoate (S3). Sticky oil (75%, 0.97 g, eluent: hexane/EA = 10:1, R_f = 0.3). ^{1}H NMR (400 MHz, $CDCl_3$) δ 6.99 (d, *J* = 7.5 Hz, 1H), 6.65 (d, *J* = 7.5 Hz, 1H), 6.63 – 6.50 (m, 2H), 5.53 (dt, *J* = 18.0, 1.5 Hz, 1H), 4.15 (t, *J* = 6.7 Hz, 2H), 3.91 (t, *J* = 5.4 Hz, 2H), 2.48 (qd, *J* = 6.7, 1.6 Hz, 2H), 2.30 (s, 3H), 2.17 (s, 3H), 1.78 – 1.65 (m, 4H), 1.25 (s, 12H), 1.20 (s, 6H). ^{13}C NMR (101 MHz, $CDCl_3$) δ 177.71, 156.96, 149.02, 136.43, 130.28, 123.61, 120.65, 111.95, 83.19, 67.94, 62.79,

42.09, 37.07, 35.00, 25.20, 24.75, 21.42, 15.79. The carbon signal attached to B was not observed. ^{11}B NMR (128 MHz, $CDCl_3$) δ 29.89. IR ν_{max} (DCM): 2975, 2931, 1745, 1641, 1321, 1261, 1031 cm^{-1}. HR-MS (APCI) calcd for $C_{25}H_{40}BO_5$ $[M+H]^+$: 431.2963, found 431.2965.

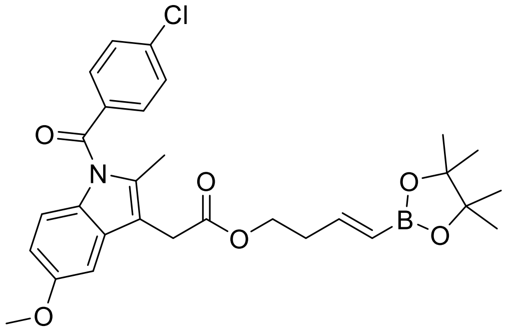

(*E*)-4-(4,4,5,5-Tetramethyl-1,3,2-dioxaborolan-2-yl)but-3-en-1-yl 2-(1-(4-chlorobenzoyl)-5-methoxy-2-methyl-1H-indol-3-yl)acetate (S4). Sticky oil (68%, 1.10 g, eluent: hexane/EA = 10:1, R_f = 0.2). ^{1}H NMR (500 MHz, $CDCl_3$) δ 7.69 – 7.64 (m, 2H), 7.50 – 7.44 (m, 2H), 6.95 (d, *J* = 2.6 Hz, 1H), 6.86 (d, *J* = 9.0 Hz, 1H), 6.66 (dd, *J* = 8.9, 2.6 Hz, 1H), 6.55 (dt, *J* = 18.0, 6.4 Hz, 1H), 5.51 (d, *J* = 18.0 Hz, 1H), 4.18 (t, *J* = 6.7 Hz, 2H), 3.84 (s, 3H), 3.65 (s, 2H), 2.50 – 2.46 (m, 2H), 2.37 (s, 3H), 1.25 (s, 12H).^{13}C NMR (126 MHz, $CDCl_3$) δ 170.79, 168.30, 156.07, 148.69, 139.23, 136.00, 133.97, 131.22, 130.67, 129.11, 114.97, 112.58, 111.78, 101.16, 83.24, 63.53, 55.74, 34.82, 30.30, 24.77, 13.38. The carbon signal attached to B was not observed. ^{11}B NMR (128 MHz, $CDCl_3$) δ 29.89. IR ν_{max} (DCM): 2964, 2930, 2834, 1733, 1674, 1645, 1260 cm^{-1}. HR-MS (APCI) calcd for $C_{29}H_{34}BClNO_6$ $[M+H]^+$: 538.2162, found 538.2158.

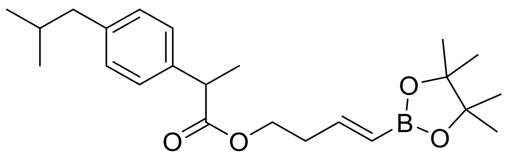

(*E*)-4-(4,4,5,5-Tetramethyl-1,3,2-dioxaborolan-2-yl)but-3-en-1-yl 2-(4-isobutylphenyl)propanoate (S5). Sticky oil (81%, 0.94 g, eluent: hexane/EA = 10:1, R_f = 0.4). ^{1}H NMR (400 MHz, $CDCl_3$) δ 7.21 – 7.17 (m, 2H), 7.10 – 7.06 (m, 2H), 6.53

(dt, J = 18.0, 6.5 Hz, 1H), 5.49 (dt, J = 18.0, 1.5 Hz, 1H), 4.22 – 4.05 (m, 2H), 3.68 (q, J = 7.2 Hz, 1H), 2.48 – 2.40 (m, 4H), 1.84 (h, J = 6.8 Hz, 1H), 1.47 (d, J = 7.2 Hz, 3H), 1.27 (s, 12H), 0.89 (d, J = 6.6 Hz, 6H). ^{13}C NMR (101 MHz, $CDCl_3$) δ 174.68, 148.89, 140.47, 137.70, 129.29, 127.20, 83.19, 63.15, 45.11, 34.88, 30.18, 24.78, 22.42, 18.55. The carbon signal attached to B was not observed. ^{11}B NMR (128 MHz, $CDCl_3$) δ 29.92. IR ν_{max} (DCM): 2970, 1740, 1360, 1322 1260, 1093, 1025 cm^{-1}. HR-MS (APCI) calcd for $C_{23}H_{36}BO_4$ [M+H]$^+$: 387.2701, found 387.2702.

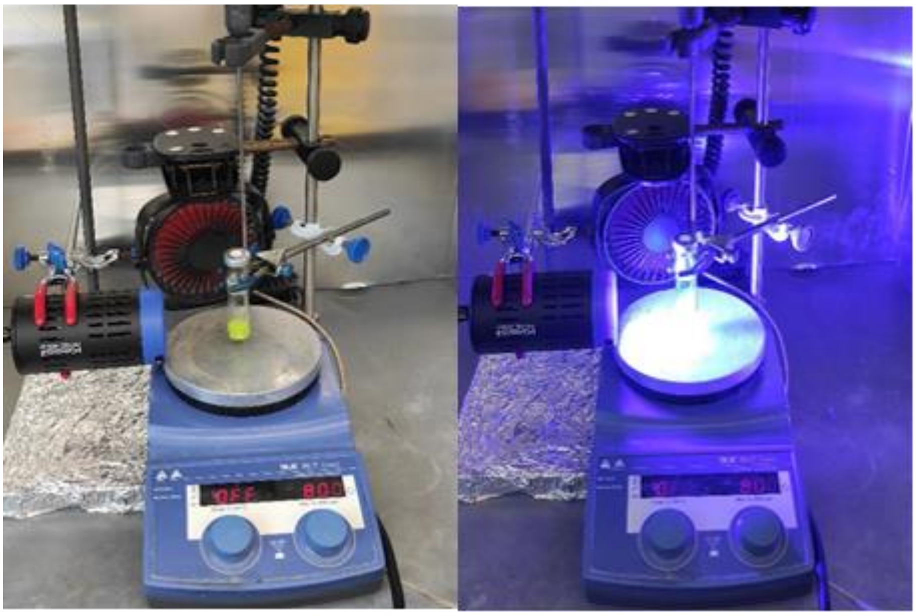

Supplementary Figure 1. Reaction setup under blue LED (40 W) irradiation

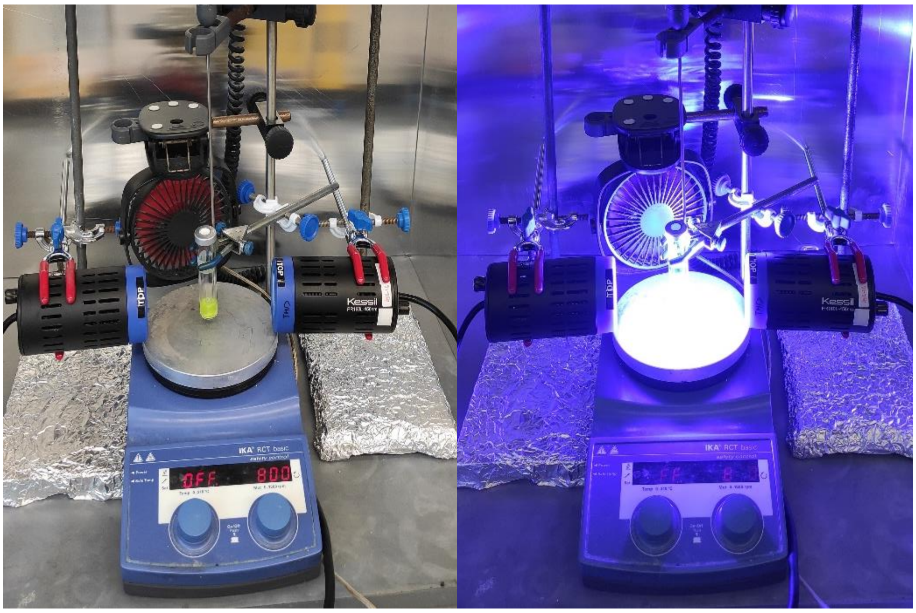

Supplementary Figure 2. Reaction setup under blue LED (80 W) irradiation

Optimization for Hydrosilylation of (*E*)-1-Pentenylboronic Acid Pinacol Ester

Supplementary Table 1. Screening of Thiols

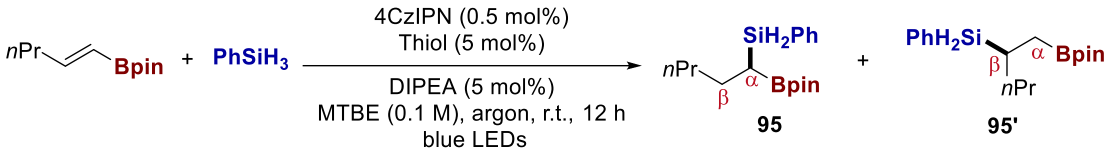

Entry	Thiol	Yield (%)	Ratio of **95**:**95'**
1	*t*BuSH	88	14:1
2	BnSH	54	14:1
**3**	**EtO_2CCH_2SH**	**92(89)[b]**	**14:1**
4	*i*Pr_3SiSH	91	14:1
5	Ph_3SiSH	90	13:1
6	PhSH	Trace	NA[c]
7	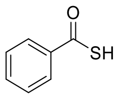	36	14:1
8	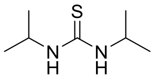	28	13:1

[a] Reaction conditions: (*E*)-1-pentenylboronic acid pinacol ester (0.2 mmol), $PhSiH_3$ (0.24 mmol), 4CzIPN (0.5 mol%), Thiol (5 mol%), DIPEA (5 mol%) in MTBE (tert-butyl methyl ether) (0.1 M) under irradiation with 40 W, 456 nm LED light at room temperature for 12 h under argon. Yield based analysis of crude ^{1}H NMR spectra using CH_2Br_2 as an internal standard. Regioselectivity was determined by GC analysis of the crude reaction mixture. [b] Isolated yield. [c] NA = not applicable.

Supplementary Table 2. Screening of Solvent

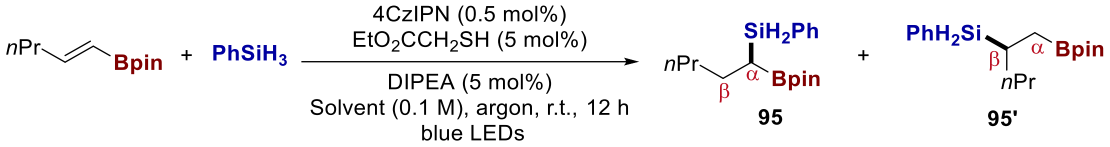

Entry	Solvent	Yield (%)	Ratio of **95**:**95'**
1	THF	86	13:1
2	1,4-Dioxane	80	13:1
**3**	**MTBE**	**92**	**14:1**
4	EtOAc	81	14:1
5	Acetone	12	14:1
6	Toluene	52	14:1
7	MeCN	0	NA[b]
8	DMF	0	NA[b]
9	DMSO	0	NA[b]

[a] Reaction conditions: (*E*)-1-pentenylboronic acid pinacol ester (0.2 mmol), $PhSiH_3$ (0.24 mmol), 4CzIPN (0.5 mol%), EtO_2CCH_2SH (5 mol%), DIPEA (5 mol%) in solvent (0.1 M) under irradiation with 40 W, 456 nm LED light at room temperature for 12 h under argon. Yield based analysis of crude ^1H NMR spectra using CH_2Br_2 as an internal standard. Regioselectivity was determined by GC analysis of the crude reaction mixture. [b] NA = not applicable. THF = Tetrahydrofuran. MTBE = *tert*-butyl methyl ether. DMF = dimethylformamide. DMSO = dimethyl sulfoxide.

Supplementary Table 3. Control Experiments

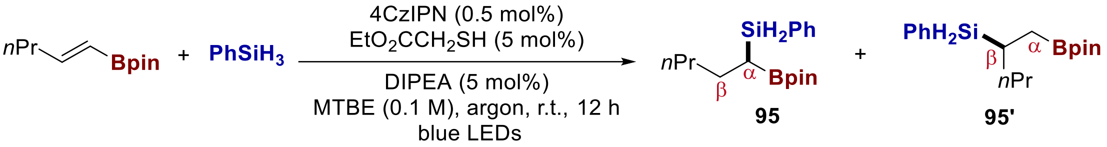

Entry	Variation	Yield (%)	Ratio of **95**:**95'**
**1**	**None**	**92**	**14:1**
2	without 4CzIPN	0	NA[b]
3	without light	0	NA[b]
4	without thiol	trace	NA[b]
5	without DIPEA	71	13:1
6	$[Mes\text{-}Acr]^+(ClO_4)^-$ instead of 4CzIPN	trace	NA[b]
7	$Ir(ppy)_2(dtbbpy)PF_6$ instead of 4CzIPN	85	11:1
8	$Ir(dFCF_3ppy)_2(dtbbpy)PF_6$ instead of 4CzIPN	73	8:1
9[c]	Without DIPEA	54	13:1
10[c]	None	92	14:1

[a] Reaction conditions: (*E*)-1-pentenylboronic acid pinacol ester (0.2 mmol), $PhSiH_3$ (0.24 mmol), 4CzIPN (0.5 mol%), EtO_2CCH_2SH (5 mol%), DIPEA (5 mol%) in MTBE (0.1 M) under irradiation with 40 W, 456 nm LED light at room temperature for 12 h under argon. Yield based analysis of crude ^1H NMR spectra using CH_2Br_2 as an internal standard. Regioselectivity was determined by GC analysis of the crude reaction mixture. [b] NA = not applicable. [c] reaction for 4 h

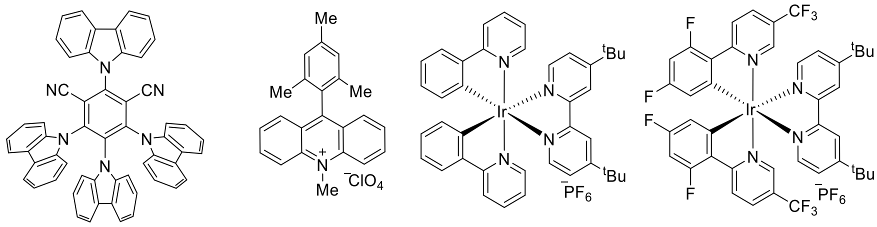

4CzIPN Mes-Acr+ClO4- Ir(ppy)2(dtbbpy)PF6 Ir(dFCF3ppy)2(dtbbpy)PF6

Optimization for Hydrosilylation of (*E*)-Styrylboronic Acid Pinacol Ester

Supplementary Table 4. Screening of Reaction Conditions

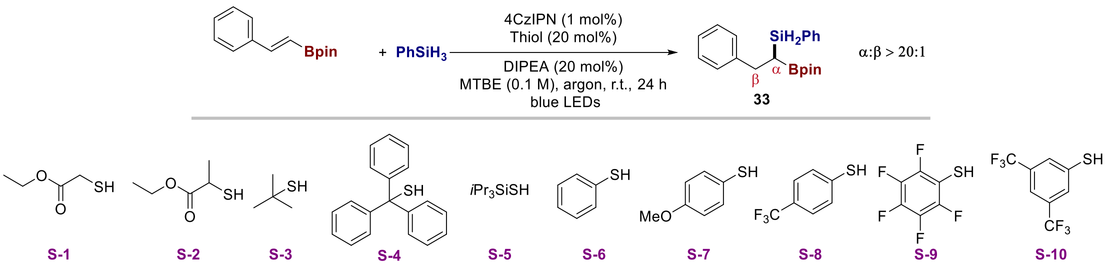

Entry	Thiol	Solvent	Yield (%)
1	S-1	MTBE	40
2	S-2	MTBE	10
3	S-3	MTBE	0
4	S-4	MTBE	0
5	S-5	MTBE	0
6	S-6	MTBE	26
7	S-7	MTBE	0
8	S-8	MTBE	27
9	S-9	MTBE	20
10	S-10	MTBE	30
11	S-1	THF	38
12	S-1	EtOAc	30
13	S-1	Et_2O	38
14	S-1	2Me-THF	22
15[b]	S-1	MTBE	53
16[b],[c]	S-1	MTBE	59
**17[b],[d]**	**S-1**	**MTBE**	**75**

[a] Reaction conditions: (*E*)-styrylboronic acid pinacol ester (0.2 mmol), $PhSiH_3$ (0.24 mmol), 4CzIPN (1 mol%), Thiol (20 mol%), DIPEA (20 mol%) in MTBE (*tert*-butyl methyl ether) (0.1 M) under irradiation with 40 W, 456 nm LED light at room temperature for 24 h under argon. Yield based analysis of crude ^1H NMR spectra using CH_2Br_2 as an internal standard. [b] under irradiation with 80 W, 456 nm LED light. [c] with 2 mol% 4CzIPN for 48 h. [d] with 4CzIPN (1+1 mol%) for 48 h.

Optimization for Hydrosilylation of Isopropenylboronic Acid Pinacol Ester

Supplementary Table 5. Screening of Reaction Conditions

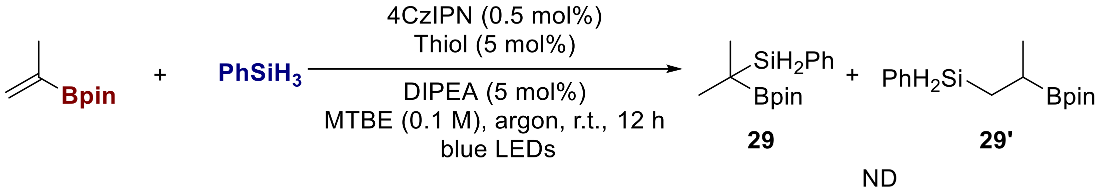

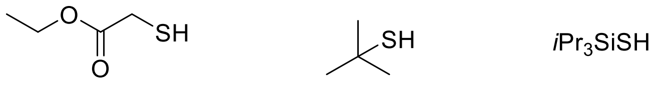

S-1 S-3 S-5

Entry	Thiol	Yield (%)
1	S-1	ND[b]
2	S-3	ND[b]
3	S-5	ND[b]

[a] Reaction conditions: isopropenylboronic acid pinacol ester (0.2 mmol), $PhSiH_3$ (0.24 mmol), 4CzIPN (0.5 mol%), Thiol (5 mol%), DIPEA (5 mol%) in MTBE (*tert*-butyl methyl ether) (0.1 M) under irradiation with 40 W, 456 nm LED light at room temperature for 12 h under argon. [b] ND = not detected.

General Procedures for Photoinduced Divergent Synthesis of Borosilanes

General Procedure I: Synthesis of β-Alkyl Geminal Borosilanes

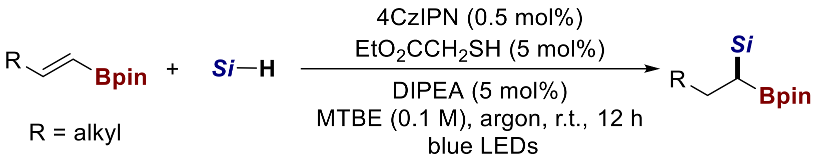

A 10 mL microwave tube equipped with a magnetic stir bar was charged with 4CzIPN (0.8 mg, 0.001 mmol, 0.5 mol%), alkenyl boronate (0.2 mmol), silane (0.24 mmol, 1.2 equiv.) and anhydrous MTBE (2 mL). The tube was capped with a Supelco aluminum crimp seal with septum (PTFE/butyl). The resulting mixture was cooled to 0 ºC using an ice-water bath and bubbled with an argon balloon for 10 min. DIPEA (1.8 μL, 0.01 mmol, 5 mol%) and EtO_2CCH_2SH (1.1 μL, 0.01 mmol, 5 mol%) were then added. After that, the reactor was placed under a blue LED (Kessil light, 40 W, 456 nm) and irradiated for 12 hrs at room temperature. The solvent was removed under vacuum. Purification by flash column chromatography on silica gel (eluent: *n*-hexane/EtOAc mixtures) gave the desired product.

General Procedure II: Synthesis of β-Aryl Geminal Borosilanes

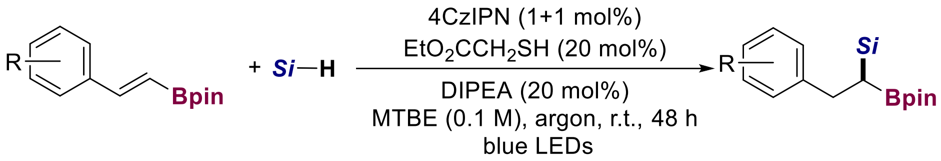

A 10 mL microwave tube equipped with a magnetic stir bar was charged with 4CzIPN (1.6 mg, 0.002 mmol, 1 mol%), alkenyl boronate (0.2 mmol), silane (0.24 mmol, 1.2 equiv.) and anhydrous MTBE (2 mL). The tube was capped with a Supelco aluminum crimp seal with septum (PTFE/butyl). The resulting mixture was cooled to 0 ºC using an ice-water bath and bubbled with an argon balloon for 10 min. DIPEA (7.2 μL, 0.04 mmol, 20 mol%), and EtO_2CCH_2SH (4.4 μL, 0.04 mmol, 20 mol%) were then added. After that, the reactor was placed under blue LED (Kessil light, 80 W, 456 nm) and irradiated for 24 hrs at room temperature. And then, add 4CzIPN (1.6 mg, 0.002 mmol)

into the microwave tube in the glovebox, and removed it from the dry box. The reaction was irradiated for additional 24 hrs under the same conditions. The solvent was removed under vacuum. Purification by flash column chromatography on silica gel (eluent: *n*-hexane/EtOAc mixtures) gave the desired product.

General Procedure III: Synthesis of Vicinal Borosilanes

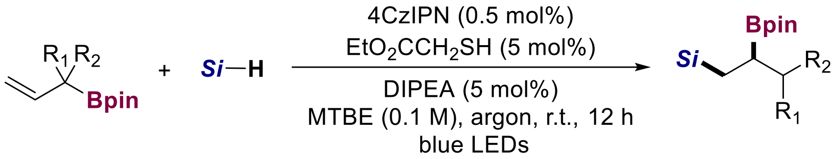

A 10 mL microwave tube equipped with a magnetic stir bar was charged with 4CzIPN (0.8 mg, 0.001 mmol, 0.5 mol%), allyl boronate (0.2 mmol), silane (0.24 mmol, 1.2 equiv.) and anhydrous MTBE (2 mL). The tube was capped with a Supelco aluminum crimp seal with septum (PTFE/butyl). The resulting mixture was cooled to 0 °C using an ice-water bath and bubbled with an argon balloon for 10 min. DIPEA (1.8 µL, 0.01 mmol, 5 mol%) and EtO_2CCH_2SH (1.1 µL, 0.01 mmol, 5 mol%) were then added. After that, the reactor was placed under a blue LED (Kessil light, 40 W, 456 nm) and irradiated for 12 hrs at room temperature. The solvent was removed under vacuum. Purification by flash column chromatography on silica gel (eluent: *n*-hexane/EtOAc mixtures) gave the desired product.

General procedure IV: Difunctionalization of Clodinafop-Propargyl

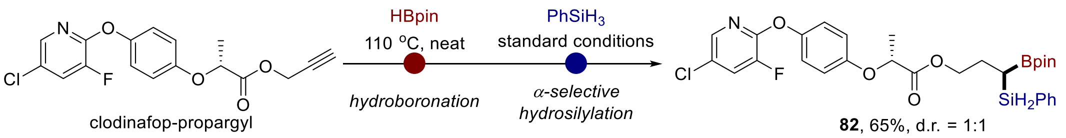

82, 65%, d.r. = 1:1

A 10 mL microwave tube equipped with a magnetic stir bar was charged with Clodinafop-propargyl (70.0 mg, 0.2 mmol) and HBpin (32.0 µL, 0.22 mmol, 1.1 equiv.) under argon, The tube was capped with a Supelco aluminum crimp seal with septum (PTFE/butyl). The resulting mixture was stirred for 16 hrs at 110 °C. Then cooled to room temperature, 4CzIPN (0.8 mg, 0.001 mmol, 0.5 mol%), $PhSiH_3$ (30 µL, 0.24 mmol, 1.2 equiv.) and anhydrous MTBE (2 mL) were added. The tube was capped with

a Supelco aluminum crimp seal with septum (PTFE/butyl). The resulting mixture was cooled to 0 ºC using an ice-water bath and bubbled with an argon balloon for 10 min. DIPEA (1.8 μL, 0.01 mmol, 5 mol%) and EtO_2CCH_2SH (1.1 μL, 0.01 mmol, 5 mol%) were then added. After that, the reactor was placed under a blue LED (Kessil light, 40 W, 456 nm) and irradiated for 12 hrs at room temperature. The solvent was removed under vacuum. Purification by flash column chromatography on silica gel (eluent: *n*-hexane/EtOAc mixtures = 10/1, R_f = 0.2) gave the desired product **82** as a colorless oil. Yield: 65% (76.1 mg).

Stepwise Synthesis of Multi-Borosilanes from $PhSiH_3$

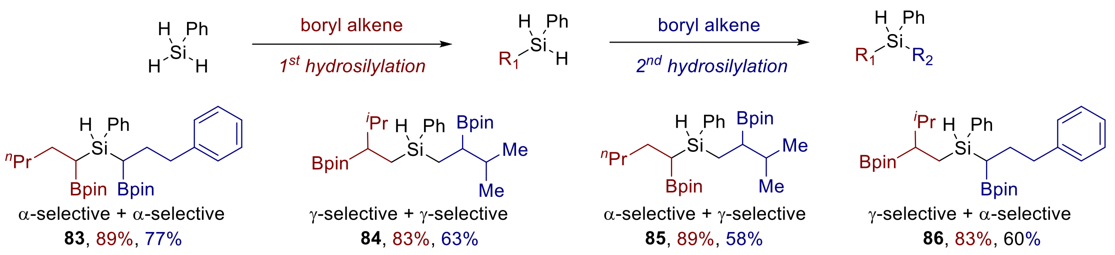

Supplementary Figure 3. Stepwise synthesis of multi-borosilanes

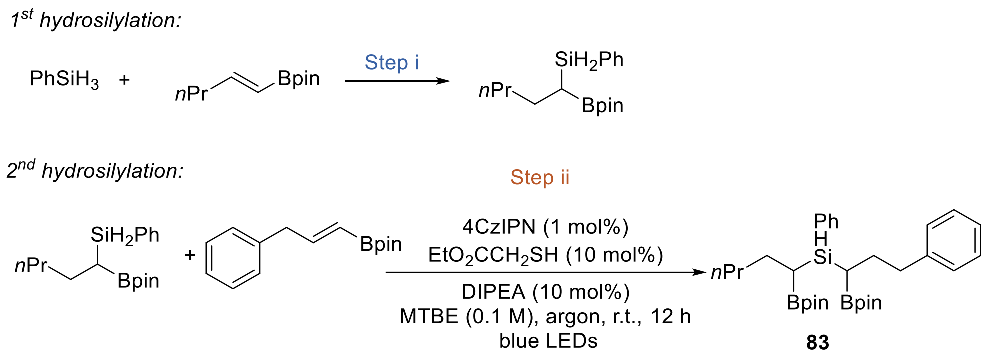

Phenyl(3-phenyl-1-(4,4,5,5-tetramethyl-1,3,2-dioxaborolan-2-yl)propyl)(1-(4,4,5,5-tetramethyl-1,3,2-dioxaborolan-2-yl)pentyl)silane (83). Step i follow **General Procedure I**, and step ii: A 10 mL microwave tube equipped with a magnetic stir bar was charged with 4CzIPN (1.6 mg, 0.002 mmol, 1 mol%), alkenyl boronate (48.8 mg, 0.2 mmol), silane (73.0 mg, 0.24 mmol, 1.2 equiv.) and anhydrous MTBE (2 mL). The tube was capped with a Supelco aluminum crimp seal with septum (PTFE/butyl). The resulting mixture was cooled to 0 °C using an ice-water bath and bubbled with an argon balloon for 10 min. DIPEA (3.6 μL, 0.02 mmol, 10 mol%) and *i*Pr_3SiSH (4.3 μL, 0.02 mmol, 10 mol%) were then added. After that, the reactor was placed under a blue LED (Kessil light, 40 W, 456 nm) and irradiated for 12 hrs at room temperature. The solvent was removed under vacuum. Purification by flash column chromatography on silica gel (eluent: *n*-hexane/EtOAc mixtures = 50/1, R_f = 0.25) gave the desired product **83** as a colorless oil. Yield: 77% (84.5 mg).

1st hydrosilylation:

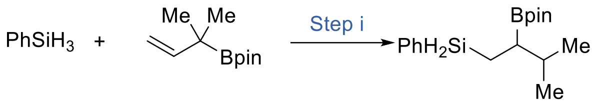

2nd hydrosilylation:

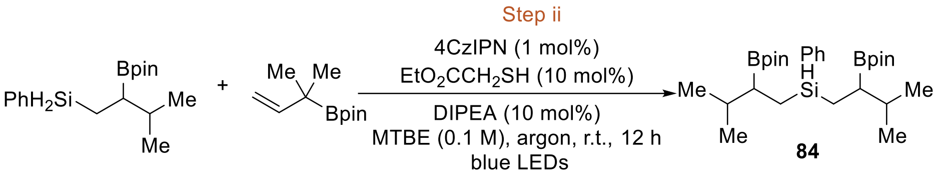

84

Bis(3-methyl-2-(4,4,5,5-tetramethyl-1,3,2-dioxaborolan-2-yl)butyl)(phenyl)silane (84). Step i follow the **General Procedure III**, and step ii: A 10 mL microwave tube equipped with a magnetic stir bar was charged with 4CzIPN (1.6 mg, 0.002 mmol, 1 mol%), allyl boronate (39.2 mg, 0.2 mmol), silane (73.0 mg, 0.24 mmol, 1.2 equiv.) and anhydrous MTBE (2 mL). The tube was capped with a Supelco aluminum crimp seal with septum (PTFE/butyl). The resulting mixture was cooled to 0 ºC using an ice-water bath and bubbled with an argon balloon for 10 min. DIPEA (3.6 μL, 0.02 mmol, 10 mol%) and *i*Pr_3SiSH (4.3 μL, 0.02 mmol, 10 mol%) were then added. After that, the reactor was placed under a blue LED (Kessil light, 40 W, 456 nm) and irradiated for 12 hrs at room temperature. The solvent was removed under vacuum. Purification by flash column chromatography on silica gel (eluent: *n*-hexane/EtOAc mixtures = 50/1, R_f = 0.3) gave the desired product **84** as a colorless oil. Yield: 63% (63.1 mg).

1st hydrosilylation:

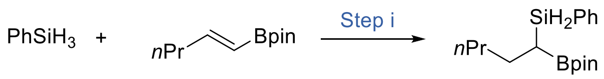

2nd hydrosilylation:

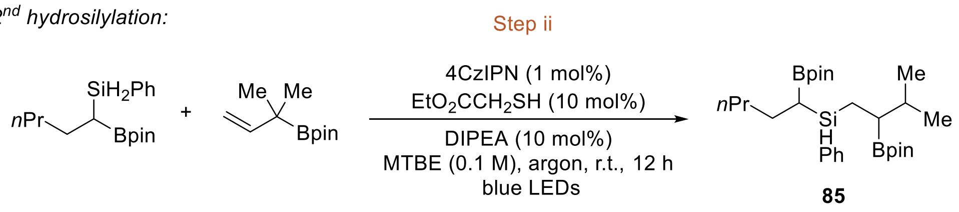

(3-Methyl-2-(4,4,5,5-tetramethyl-1,3,2-dioxaborolan-2-yl)butyl)(phenyl)(1-(4,4,5,5-tetramethyl-1,3,2-dioxaborolan-2-yl)pentyl)silane (85). Step i follow the **General Procedure I**, and step ii: A 10 mL microwave tube equipped with a magnetic stir bar was charged with 4CzIPN (1.6 mg, 0.002 mmol, 1 mol%), allyl boronate (39.2 mg, 0.2 mmol), silane (73.0 mg, 0.24 mmol, 1.2 equiv.) and anhydrous MTBE (2 mL). The tube was capped with a Supelco aluminum crimp seal with septum (PTFE/butyl). The resulting mixture was cooled to 0 ºC using an ice-water bath and bubbled with an argon balloon for 10 min. DIPEA (3.6 µL, 0.02 mmol, 10 mol%) and *i*Pr_3SiSH (4.4 µL, 0.02 mmol, 10 mol%) were then added. After that, the reactor was placed under a blue LED (Kessil light, 40 W, 456 nm) and irradiated for 12 hrs at room temperature. The solvent was removed under vacuum. Purification by flash column chromatography on silica gel (eluent: *n*-hexane/EtOAc mixtures 50/1, R_f = 0.3) gave the desired product **85** as a colorless oil. Yield: 58% (58.1 mg).

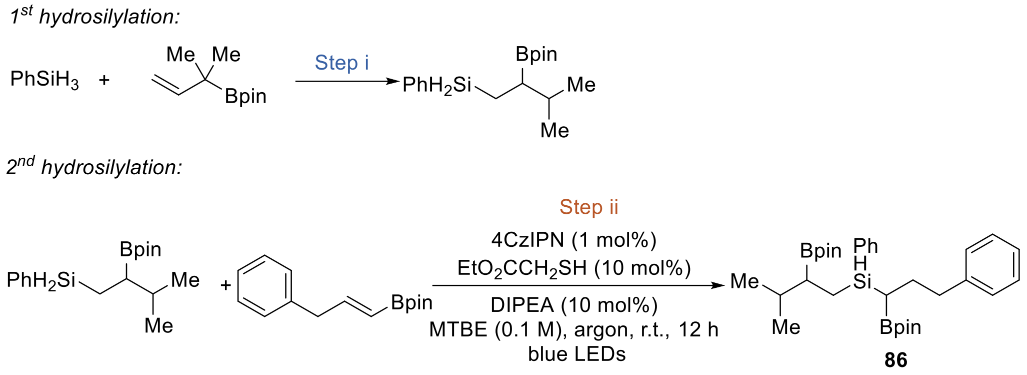

(3-Methyl-2-(4,4,5,5-tetramethyl-1,3,2-dioxaborolan-2-yl)butyl)(phenyl)(3-phenyl-1-(4,4,5,5-tetramethyl-1,3,2-dioxaborolan-2-yl)propyl)silane (86). Step i follow the **General Procedure III**, and step ii: A 10 mL microwave tube equipped with a magnetic stir bar was charged with 4CzIPN (1.6 mg, 0.002 mmol, 1 mol%), alkenyl boronate (48.8 mg, 0.2 mmol), silane (73.0 mg, 0.24 mmol, 1.2 equiv.) and anhydrous MTBE (2 mL). The tube was capped with a Supelco aluminum crimp seal with septum (PTFE/butyl). The resulting mixture was cooled to 0 ºC using an ice-water bath and bubbled with an argon balloon for 10 min. DIPEA (3.6 μL, 0.02 mmol, 10 mol%) and *i*Pr$_3$SiSH (4.3 μL, 0.02 mmol, 10 mol%) were then added. After that, the reactor was placed under a blue LED (Kessil light, 40 W, 456 nm) and irradiated for 12 hrs at room temperature. The solvent was removed under vacuum. Purification by flash column chromatography on silica gel (eluent: *n*-hexane/EtOAc mixtures = 50/1, R_f = 0.3) gave the desired product **86** as a colorless oil. Yield: 60% (65.8 mg).

Derivatization of the Borosilanes

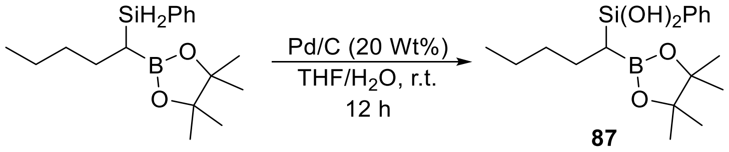

Phenyl(1-(4,4,5,5-tetramethyl-1,3,2-dioxaborolan-2-yl)pentyl)silanediol (87). Prepared according to a reported literature[8]. A tube equipped with a stirring bar was charged with Pd/C (20 wt%), evacuated and refilled with argon (x 3). Then, geminal borosilane **95** (60.8 mg, 0.2 mmol), THF (2 mL) and H_2O (0.2 mL) were added. The mixture was stirred overnight at room temperature, and filtered through a pad of silica gel washed by Et_2O (10 mL x 3). The combined filtrates were evaporated and purification by flash column chromatography on silica gel (eluent: *n*-hexane/EtOAc mixtures = 3/1, R_f = 0.45) gave the desired product **87** as a colorless oil. Yield: 85% (57.2 mg).

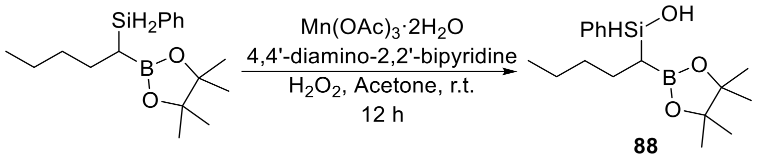

Phenyl(1-(4,4,5,5-tetramethyl-1,3,2-dioxaborolan-2-yl)pentyl)silanol (88). Prepared according to reported literature[9]. A tube equipped with a stirring bar was charged with $Mn(OAc)_3·2H_2O$ (2.7 mg, 5 mol%), 4,4'-diamino-2,2'-bipyridine (3.8 mg, 10 mol%) under air. Then, geminal borosilane **95** (60.8 mg, 0.2 mmol) and acetone (1.5 mL) were added. H_2O_2 (30 wt%, 2.5 equiv.) was then added by dropwise the mixture was stirred for 12 hrs at room temperature. Quenched with saturated $Na_2S_2O_3$ aqueous solution. The mixture was extracted with Et_2O (10 mL x 3). The organic layer was dried over anhydrous Na_2SO_4, filtered and evaporated. Purification by flash column chromatography on silica gel (eluent: *n*-hexane/EtOAc mixtures = 5/1, R_f = 0.4) gave the desired product **88** as a colorless oil. Yield: 68% (43.4 mg).

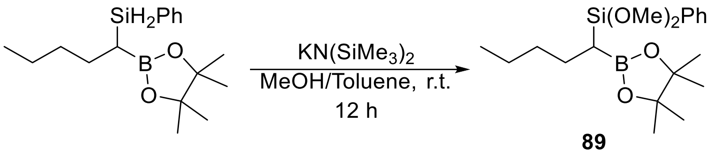

89

Dimethoxy(phenyl)(1-(4,4,5,5-tetramethyl-1,3,2-dioxaborolan-2-yl)pentyl)silane (89). Prepared according to reported literature[10]. A tube equipped with a stirring bar was charged with $KN(SiMe_3)_2$ (40.0 mg, 0.20 mmol, 1 equiv.), evacuated and refilled with argon (x 3). Then, geminal borosilane **95** (60.8 mg, 0.2 mmol), MeOH (0.3 mL) and toluene (2 mL) were added. The mixture was stirred for 12 hrs at room temperature, quenched by addition of saturated aqueous NH_4Cl (2.0 mL). The mixture was extracted with Et_2O (10 mL x 3). The organic layer was dried over anhydrous Na_2SO_4, filtered and evaporated. Purification by flash column chromatography on silica gel (eluent: *n*-hexane/EtOAc mixtures = 5/1, R_f = 0.4) gave the desired product **89** as a colorless oil. Yield: 52% (37.9 mg).

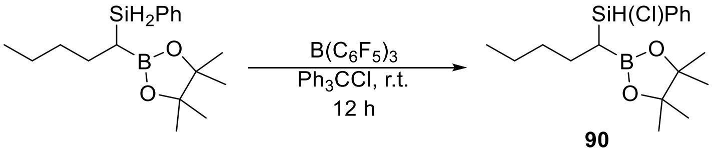

90

Chloro(phenyl)(1-(4,4,5,5-tetramethyl-1,3,2-dioxaborolan-2-yl)pentyl)silane (90). Prepared according to reported literature[11]. A tube equipped with a stirring bar was charged with $B(C_6F_5)_3$ (1.3 mg, 0.004 mmol, 2 mol%), Ph_3CCl (61.3 mg, 0.22 mmol, 1.1 equiv.), evacuated and refilled with argon (x 3). Then, geminal borosilane **95** (60.8 mg, 0.2 mmol), DCM (2 mL) were added. The mixture was stirred for 12 hrs at room temperature. The solvent was removed under vacuum. Purification by flash column chromatography on silica gel (eluent: *n*-hexane/EtOAc mixtures = 30/1, R_f = 0.25) gave the desired product **90** as a colorless oil. Yield: 57% (38.5 mg).

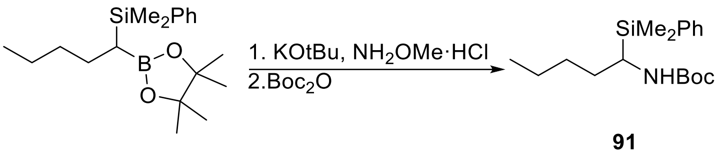

91

***tert*-Butyl (1-(dimethyl(phenyl)silyl)pentyl)carbamate (91).** Prepared according to reported literature[5]. A tube equipped with a stirring bar was charged with KO*t*Bu (67.3 mg, 0.6 mmol, 3 equiv.), NH_2OMe·HCl (25.1 mg, 0.3 mmol, 1.5 equiv.), evacuated and refilled with argon (x 3). Then, geminal borosilane **49** (66.5 mg, 0.2 mmol) and toluene (0.5 mL) were added. The mixture was stirred for 16 hrs at 80 °C. The reaction was allowed to cool to room temperature, and di-*tert*-butyl dicarbonate (1.0 M in THF, 0.24 mL, 0.24 mmol, 1.50 equiv.) was added and allowed to stir at room temperature for 1.5 hours, filtered through a pad of silica gel washed by Et_2O (10 mL x 3). The combined filtrates were evaporated and purification by flash column chromatography on silica gel (eluent: *n*-hexane/EtOAc mixtures = 3/1, R_f = 0.35) gave the desired product **91** as a colorless oil. Yield: 83% (53.3 mg).

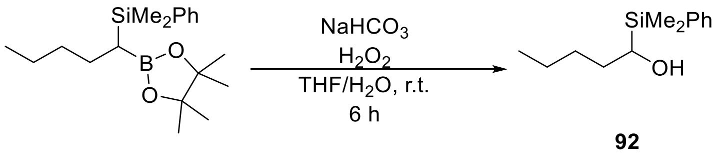

92

1-(Dimethyl(phenyl)silyl)pentan-1-ol (92). Prepared according to reported literature[10]. A tube equipped with a stirring bar was charged with $NaHCO_3$ (84 mg, 1.0 mmol, 5 equiv.). Then, geminal borosilane **49** (66.5 mg, 0.2 mmol) and H_2O (1 mL) were added. H_2O_2 (30 wt%, 10 equiv.) was then added by dropwise the mixture was stirred for 3 hrs at room temperature. Quenched with saturated $Na_2S_2O_3$ aqueous solution. The mixture was extracted with Et_2O (10 x 3 mL). The organic layer was dried over anhydrous Na_2SO_4, filtered and evaporated. Purification by flash column chromatography on silica gel (eluent: *n*-hexane/EtOAc mixtures = 5/1, R_f = 0.35) gave the desired product **92** as a colorless oil. Yield: 90% (40.0 mg).

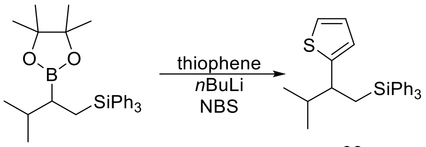

93

(3-Methyl-2-(thiophen-2-yl)butyl)triphenylsilane (93). Prepared according to reported literature[12]. A tube equipped with a stirring bar was charged with thiophene (19 μL, 0.24 mmol, 1.2 equiv.) and THF (1 mL) under argon, cooled to -78 ^{o}C and treated with *n*-BuLi (0.2 mL, 0.2 mmol, 1 M in THF). Then the mixture was allowed to warm up to 0 °C and stirred for 30 minutes. After cooling to -78 ^{o}C again, a solution of vicinal borosilane **75** (91 mg, 0.2 mmol) in THF (0.5 mL) was added dropwise. The reaction mixture was allowed to stir at -78 ^{o}C for 1 hour. NBS (42.7 mg, 0.24 mmol, 1.2 equiv.) in THF (0.5 mL) was added dropwise and the mixture was stirred at -78 ^{o}C for 1 hour. Quenched with saturated $Na_2S_2O_3$ aqueous solution (2.0 mL). The mixture was extracted with Et_2O (10 mL x 3). The organic layer was dried over anhydrous Na_2SO_4, filtered and evaporated. Purification by flash column chromatography on silica gel (eluent: *n*-hexane/EtOAc mixtures = 5/1, R_f = 0.25) gave the desired product **93** as a colorless sticky oil. Yield: 68% (59.3 mg).

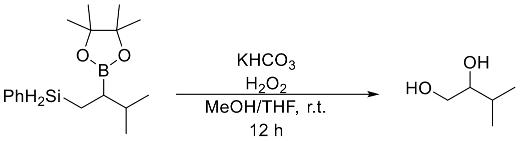

94

3-Methylbutane-1,2-diol (94). A tube equipped with a stirring bar was charged with $KHCO_3$ (100 mg, 1.0 mmol, 5 equiv.). Then, vicinal borosilane **63** (60.8 mg, 0.2 mmol), MeOH (1.0 mL) and H_2O (1 mL) were added. H_2O_2 (30 wt%, 10 equiv.) was then added by dropwise the mixture was stirred for 12 hrs at room temperature. Quenched with saturated $Na_2S_2O_3$ aqueous solution. The mixture was extracted with Et_2O (10 x 3 mL). The organic layer was dried over anhydrous Na_2SO_4, filtered and evaporated. Purification by flash column chromatography on silica gel (eluent: *n*-hexane/EtOAc mixtures = 1/1, R_f = 0.35) gave the desired product **94** as a colorless oil. Yield: 61% (12.7 mg).

General Procedures for Scaling Up

General Procedure for Scaling Up in Batch Reactor

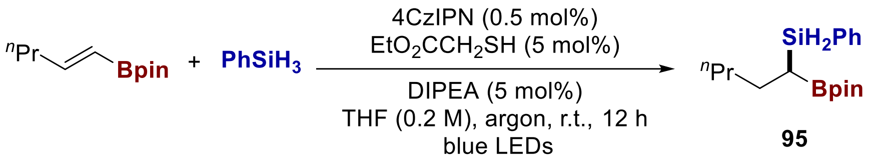

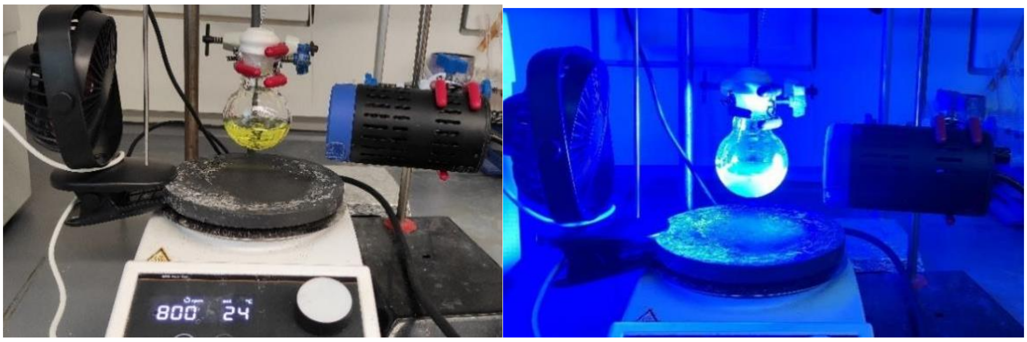

Supplementary Figure 4. Batch reactor set-up with light irradiation

The synthesis of geminal borosilanes 95 in batch reactor:

A 50 mL round bottom flask equipped with a magnetic stir bar was charged with 4CzIPN (20 mg, 0.025 mmol, 0.5 mol%), alkenyl boronate (0.98 g, 5 mmol), $PhSiH_3$ (0.65 g, 6 mmol, 1.2 equiv), DIPEA (45 μL, 0.25 mmol, 5 mol%) and EtO_2CCH_2SH (28 μL, 0.25 mmol, 5 mol%). The reagents were dissolved in anhydrous THF and the total volume of the solution was adjusted to 25 mL. The resulting mixture was cooled to 0 ^{o}C using an ice-water bath, and bubbled with an argon balloon for 20 min. After that, the reactor was placed under a blue LED (kessil light, 40 W, 456 nm) and irradiated for 12 hrs at room temperature (Supplementary Figure 4). The solvent was removed under vacuum. Silica gel chromatography (eluent: *n*-hexane/EtOAc = 50/1, R_f = 0.4) of the crude product afforded the desired compound **95** as a colorless oil in 78% yield (1.19 g).

General Procedure for Scaling Up by Continuous-Flow Synthesis

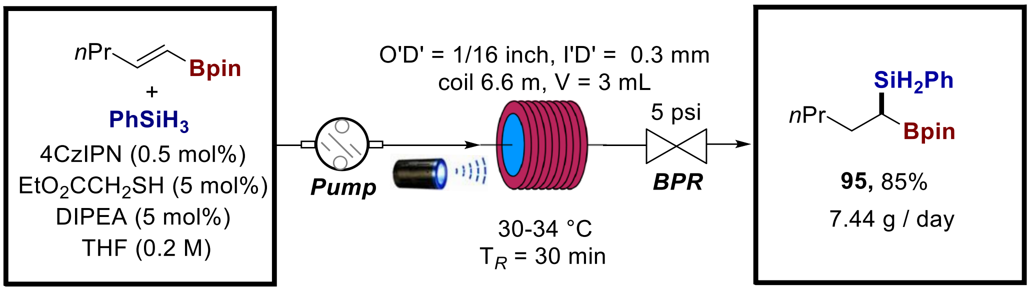

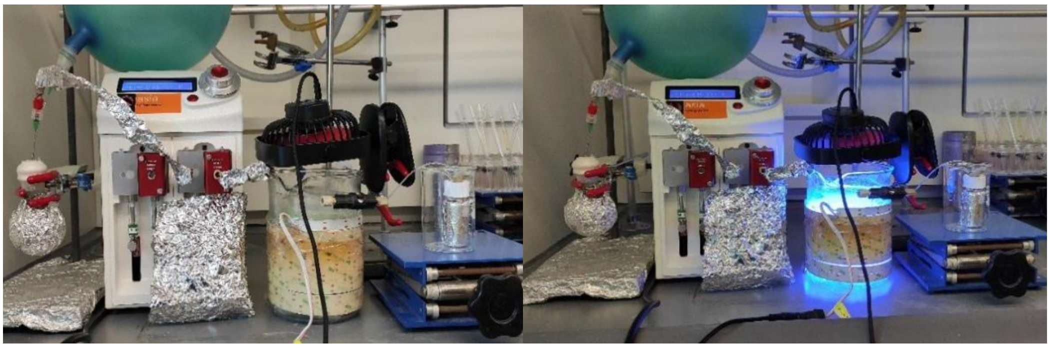

Supplementary Figure 5. Flow set-up with light irradiation

The synthesis of geminal borosilanes 95 in flow reactor:

A 50 mL round bottom flask equipped with a magnetic stir bar was charged with 4CzIPN (0.025 mmol, 20 mg), alkenyl boronate (0.98 g, 5 mmol), $PhSiH_3$ (0.65 g, 6 mmol, 1.2 equiv), DIPEA (45 μL, 0.25 mmol, 5 mol%) and EtO_2CCH_2SH (28 μL, 0.25 mmol, 5 mol%). The reagents were dissolved in anhydrous THF (Solubility of 4CzIPN in THF more than MTBE) and the total volume of the solution was adjusted to 25 mL. The resulting mixture was cooled to 0 °C using an ice-water bath, and bubbled with an argon balloon for 20 min. After that, the reaction solution was introduced to the flow apparatus (Supplementary Figure 5). The flow apparatus was purged with degassed argon to remove the air first. The Asia Syrris pump (Model No. 2200292) was then connected to the reaction mixture and the tubing with a 5 psi back-pressure regulator (BPR). The HPFA (high purity perfluoroalkoxyalkane) tubing (O.D. = 1/16 inch, I.D. = 0.3 mm, length = 6.6 m, volume = 3 mL) was rounded on a glass cylinder (I.D. = 10 cm). The reaction was placed under a blue LED strips (18 w). The flow apparatus was

cooled by two fans, keeping the ambient temperature around at 30-34 °C. The flow apparatus itself was set up with residence time (T_R) = 30 min, flow rate = 100 uL/min. After 90 min of equilibration, the product mixture was collected for 60 min. A crude sample (6 mL) was taken from the collected solution and analyzed by ^{1}H-NMR spectroscopy using CH_2Br_2 as an internal standard. Full conversion of alkenyl boronate was observed and the ^{1}H-NMR yield of product **95** was determined to be 88%. The crude NMR sample was recovered and combined with the reaction mixture. The combined crude was concentrated and purified by column chromatography (eluent: *n*-hexane/EtOAc = 50/1, R_f = 0.4) of the crude product afforded the desired compound **95** in 85% yield (the productivity was 7.44 g/day).

Supplementary Discussion

Radical Inhibition Experiments

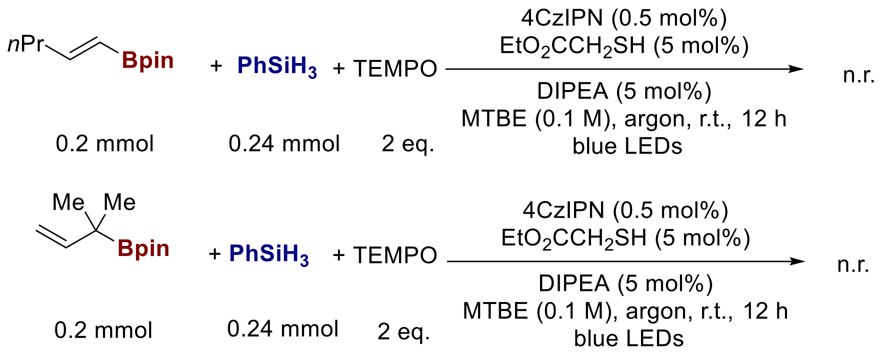

When TEMPO (2.0 equiv.) was introduced into the model reactions, no corresponding products were observed. These results indicated that a free radical process was involved.

Radical Clock Experiment

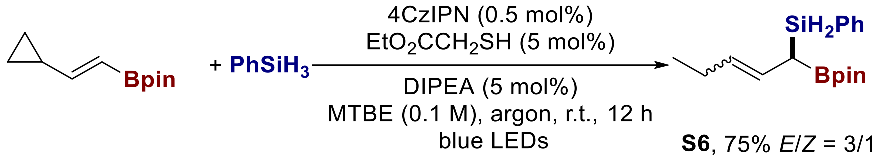

A 10 mL microwave tube equipped with a magnetic stir bar was charged with 4CzIPN (0.8 mg, 0.001 mmol, 0.5 mol%), alkenyl boronate (38.8 mg, 0.2 mmol), $PhSiH_3$ (30 µL, 0.24 mmol, 1.2 equiv.) and anhydrous MTBE (2 mL). The tube was capped with a Supelco aluminum crimp seal with septum (PTFE/butyl). The resulting mixture was cooled to 0 °C using an ice-water bath and bubbled with an argon balloon for 10 min. DIPEA (1.8 µL, 0.01 mmol, 5 mol%) and EtO_2CCH_2SH (1.1 µL, 0.01 mmol, 5 mol%) were then added. After that, the reactor was placed under a blue LED (Kessil light, 40 W, 456 nm) and irradiated for 12 hrs at room temperature. The solvent was removed under vacuum. Purification by column chromatography on silica gel (eluent: *n*-hexane/EtOAc mixtures = 100:1, R_f = 0.3) gave the desired product **S6** as a colorless oil. Yield: 75% (45.3 mg).

Phenyl(1-(4,4,5,5-tetramethyl-1,3,2-dioxaborolan-2-yl)pent-2-en-1-yl)silane (S6). ^{1}H NMR (400 MHz, $CDCl_3$) δ 7.63 – 7.55 (m, 2H), 7.40 – 7.31 (m, 3H), 5.55 – 5.25 (m, 2H), 4.40 – 4.32 (m, 2H), 2.01 – 1.93 (m, 2H), 1.90 – 1.81 (m, 1H), 1.18 (d, *J* = 8.0 Hz, 12H), 0.90 (t, *J* = 7.4 Hz, 3H) (***E*-S6**), 0.83 (t, *J* = 7.5 Hz, 3H) (***Z*-S6**). ^{13}C NMR (126 MHz, $CDCl_3$) δ 135.68, 135.63, 131.86, 129.62, 127.71, 124.39, 83.24, 25.93, 24.79, 24.69, 14.28. ^{11}B NMR (128 MHz, $CDCl_3$) δ 33.56. IR ν_{max} (DCM): 2977, 2926, 2118, 1588, 1465, 1428, 1350, 1144 cm^{-1}. HR-MS (EI) calcd for $C_{17}H_{26}BO_2Si$ [M-H]$^+$: 301.1795, found 301.1789.

^{1}H NMR Spectra of DIPEA, Thiol, and Thiol/DIPEA Mixture

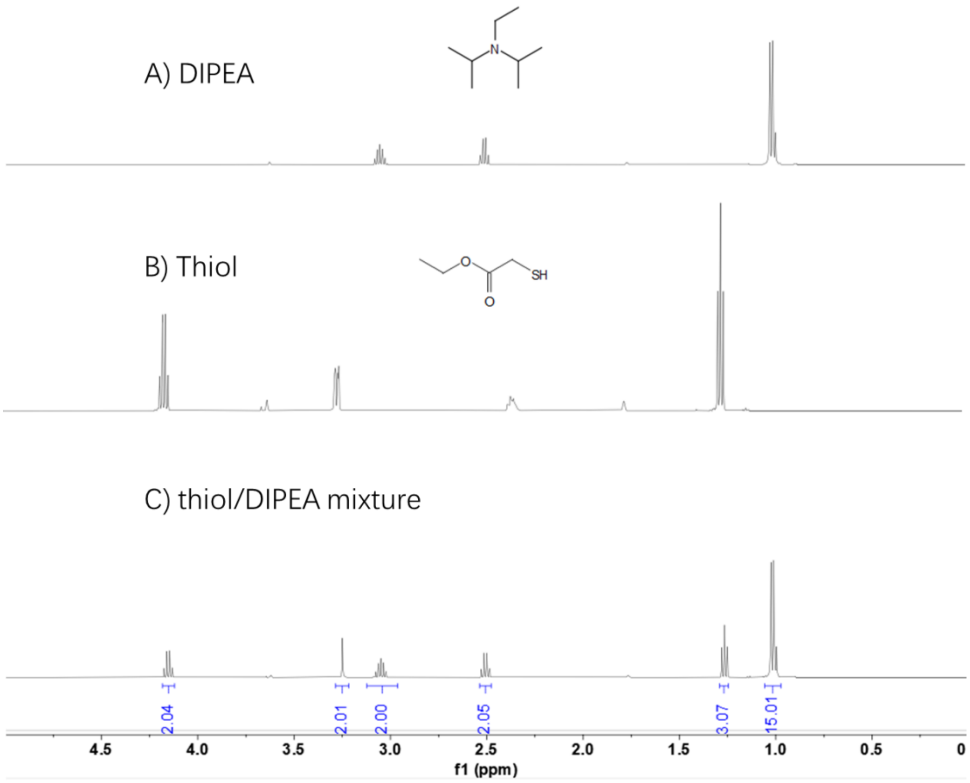

Supplementary Figure 6. ^{1}H NMR measurement

Procedure:

a) To a dry NMR tube, DIPEA (0.04 mmol) and 0.6 ml THF-d8 were added. The tube was sealed with a rubber stopper and ^{1}H NMR spectrum was recorded.

b) To a dry NMR tube, EtO_2CCH_2SH (0.04 mmol) and 0.6 ml THF-d8 were added. The tube was sealed with a rubber stopper and ^{1}H NMR spectrum was recorded.

c) To a dry NMR tube, DIPEA (0.04 mmol), EtO_2CCH_2SH (0.04 mmol) and 0.6 ml THF-d8 were added. The tube was sealed with a rubber stopper and ^{1}H NMR spectrum was recorded.

Cyclic Voltammetry (CV) Measurements

Cyclic Voltammograms were collected using a VersaSTAT 3 Potentiostat Galvanostat from Princeton Applied Research. The sample (0.01 M) and tetrabutylammonium tetrafluoroborate (0.1 M) in acetonitrile was used for tests. Measurements were performed using glassy carbon as working electrode, platinum wire as counter electrode, and 3.5 M NaCl silver-silver chloride as reference electrode in a scan rate of 0.1 V/s. Ferrocene ($E_{1/2}$ = +0.42 V vs. SCE) was added at the end of the measurements as an internal standard to determine the precise potential scale. Potential values are given versus the saturated calomel electrode (SCE).

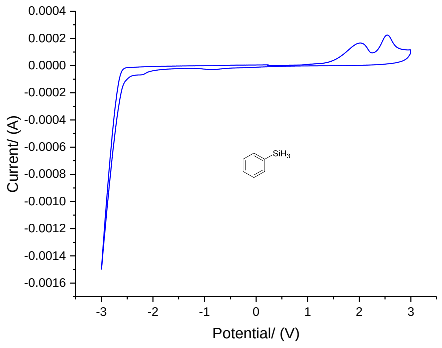

$E_{p/2}^{ox}$ (phenylsilane) = +1.63 V vs. SCE

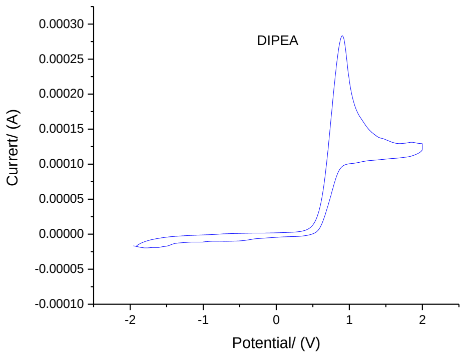

$E_{p/2}^{ox}$ (DIPEA) = +0.63 vs. SCE

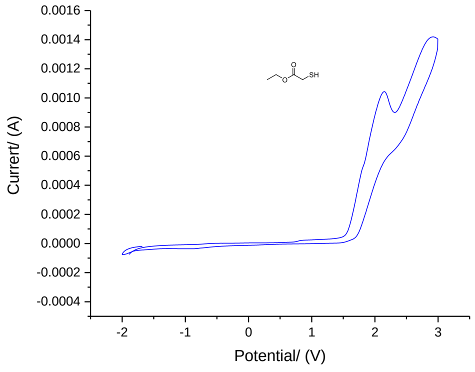

$E_{p/2}^{ox}$ (ethyl thioglycolate) = +0.75 vs. SCE

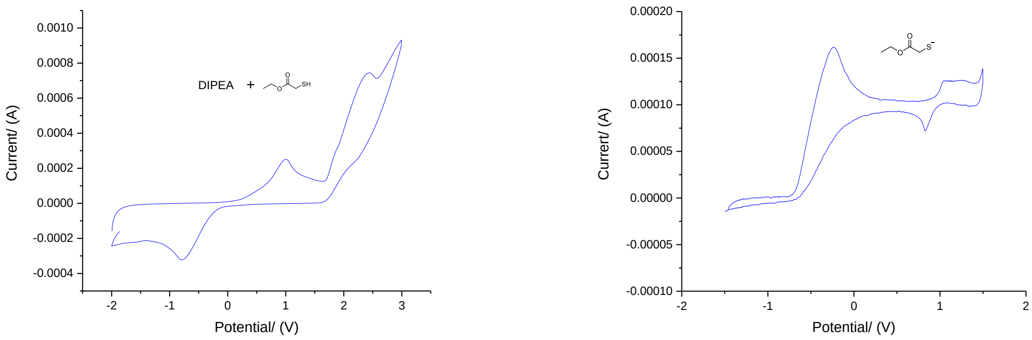

$E_{p/2}^{ox}$ (thiol/DIPEA mixture) = +0.68 V vs. SCE $E_{p/2}^{ox}$ (sodium thiolate)= -0.74 V vs. SCE

Supplementary Figure 7. CV measurements.

The cyclic voltammetry (CV) results (Supplementary Fig. 7) showed that the CV performance of the thiol/DIPEA mixture is completely different from that of DIPEA, thiol or thiolate. In particular, a new reduction peak appeared for the thiol/DIPEA mixture, which suggested formation of a new species. Since our calculation results suggest that the complexation of thiyl radical with DIEPA is beneficial for the reaction process, we speculate that the new reduction peak is associated with this thiyl radical-DIPEA complex. Moreover, very little change of chemical shifts was observed in ^{1}H NMR studies (Supplementary Fig. 6). The complexation probably occurs after the oxidation of thiol.

Stern-Volmer Fluorescence Quenching Experiments

In a typical experiment, a solution of photocatalyst 4CzIPN in anhydrous MTBE (1.25 × 10^{-4} M) was added with an appropriate amount of quencher in a quartz cuvette. Then the emission of the sample was collected. The emission intensity at 520 nm was collected with excited wavelength of 440 nm.

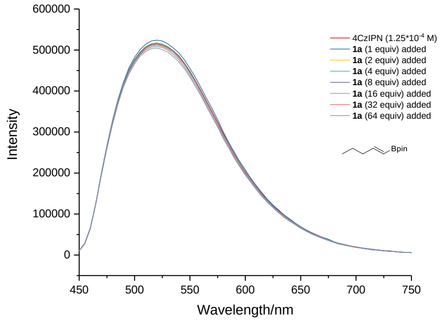

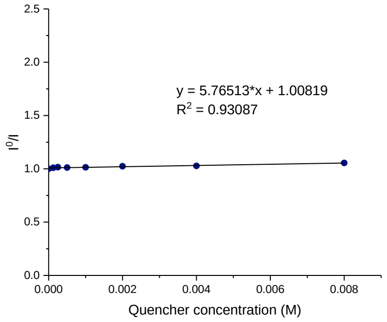

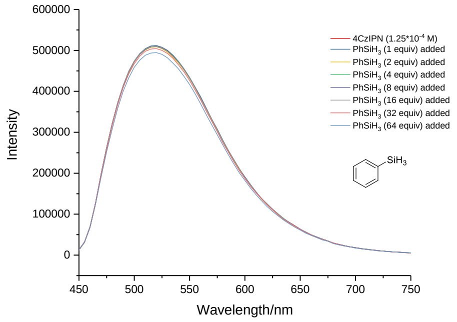

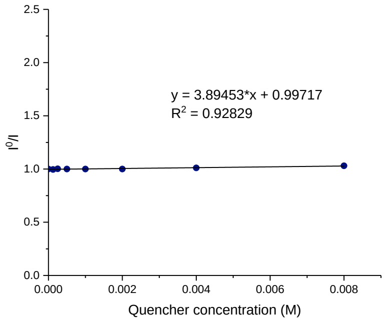

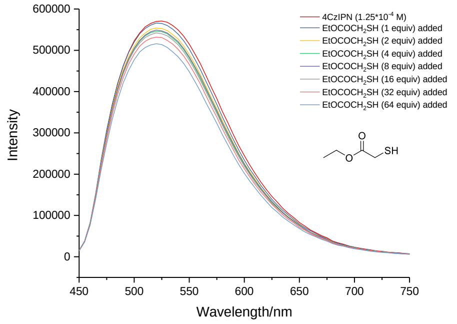

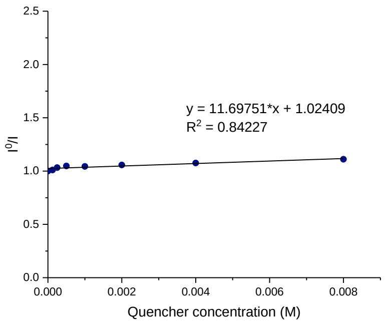

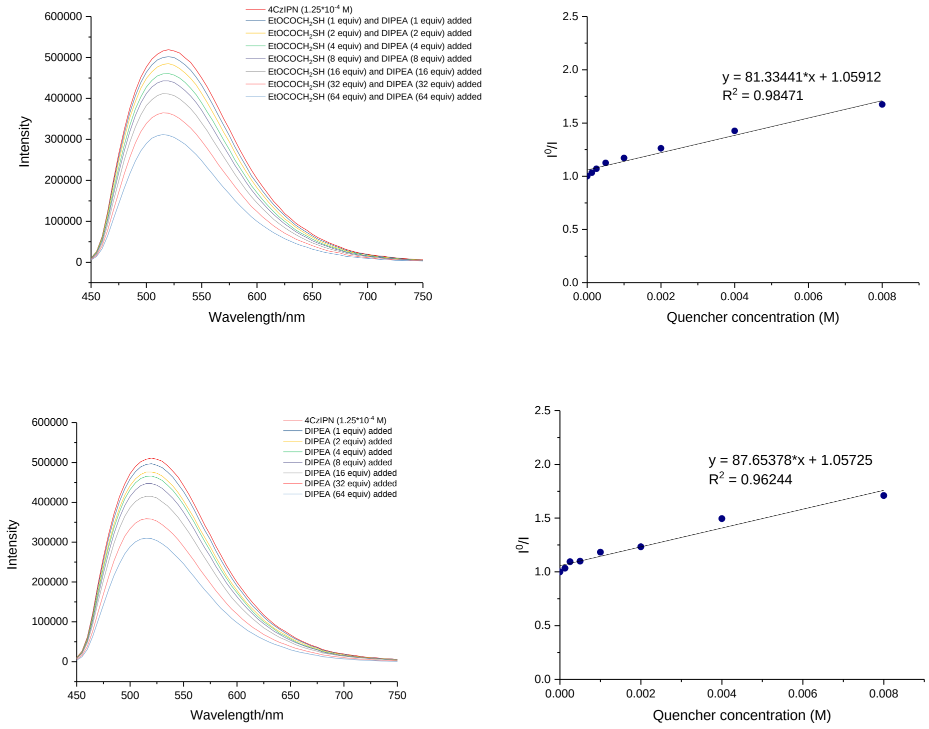

Supplementary Figure 8. Stern-Volmer fluorescence quenching studies.

Stern–Volmer fluorescence quenching studies indicated that the excited photocatalyst can be reductively quenched by the mixture of thiol and DIPEA. Based on the quenching studies, DIPEA is similarly effective as the thiol-DIPEA mixture as quencher. We cannot rule out other mechanistic pathways. For instance, the excited 4CzIPN might oxidizes the DIPEA to afford an amine radical cation species, which selectively abstract a hydrogen atom from Si-H bond to deliver the silyl radical. Subsequently, the silyl radical adds to the α-position of the alkenyl boronate to deliver an alkyl radical intermediate which undergoes polarity-matched HAT process with thiol to give the thiyl radical and the borosilane product. The thiyl radical could oxidize the reduced photocatalyst to close the photocatalytic cycle or engage in radical chain processes.

Deuterium-labeling Experiments

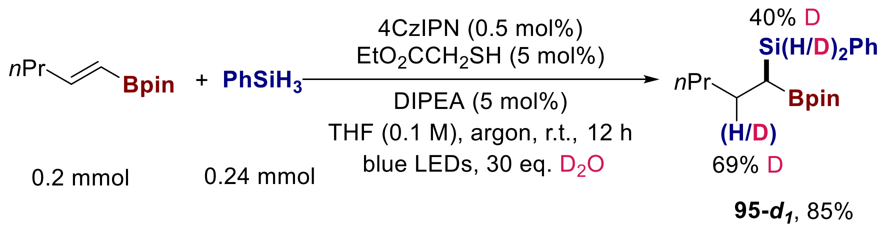

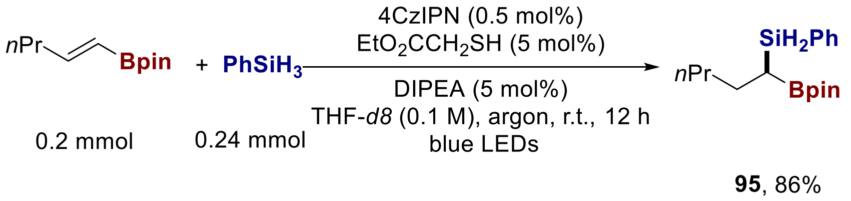

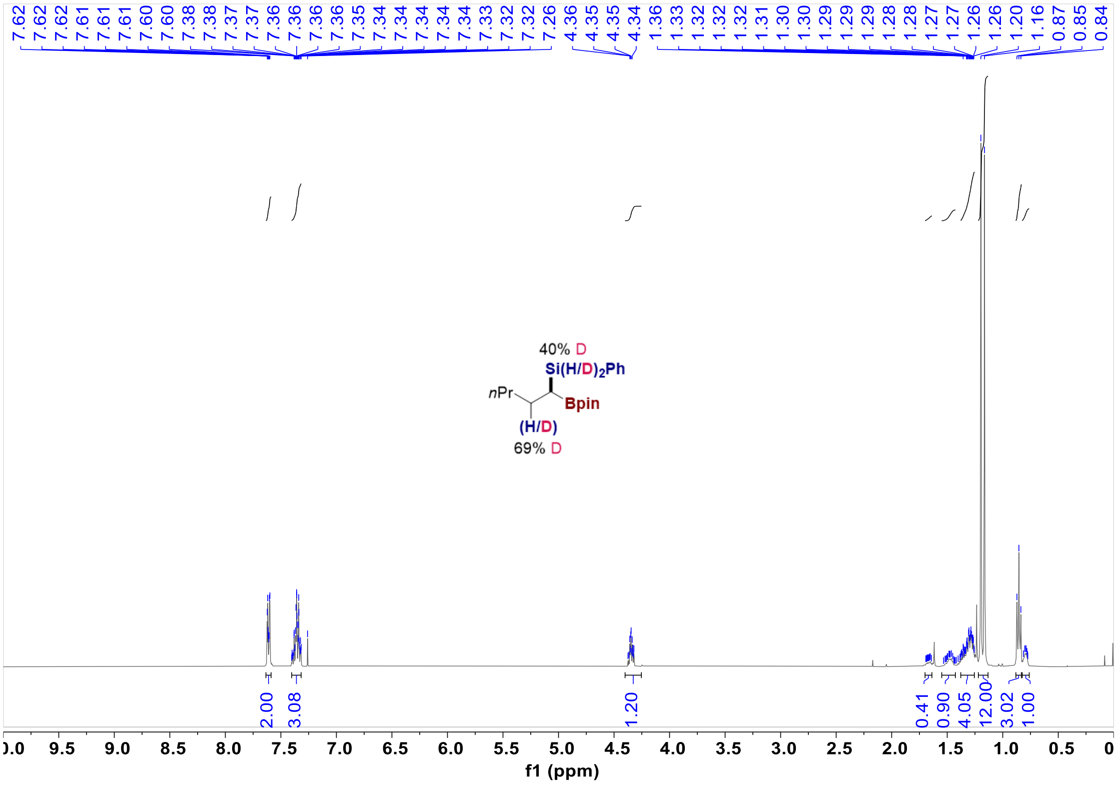

Supplementary Figure 9. ^{1}H NMR spectra of **95-*d*1**

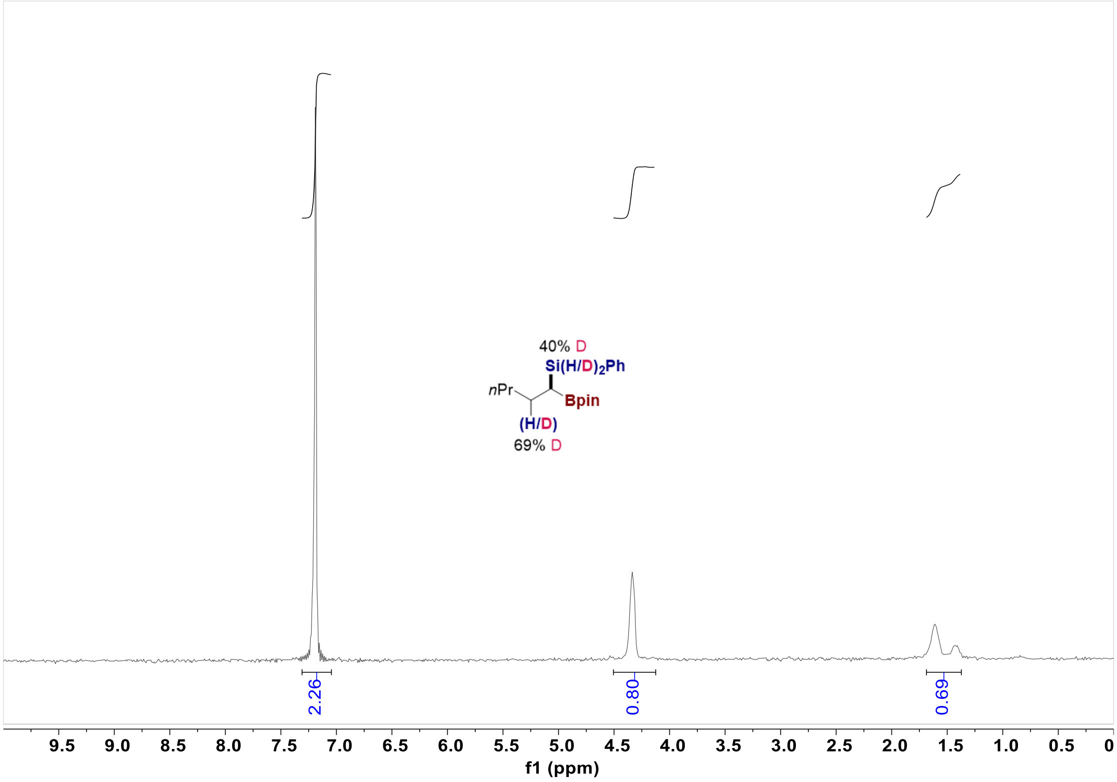

Supplementary Figure 10. ^{2}H NMR spectra of **95-*d*1**. $CDCl_3$ (2.26 equiv.) was used as an internal standard

A 10 mL microwave tube equipped with a magnetic stir bar was charged with 4CzIPN (0.8 mg, 0.001 mmol, 0.5 mol%), alkenyl boronate (39.2 mg, 0.2 mmol), $PhSiH_3$ (30 μL, 0.24 mmol, 1.2 equiv.), anhydrous THF (2 mL) and D_2O (110 μL, 6 mmol, 30 equiv.). The tube was capped with a Supelco aluminum crimp seal with septum (PTFE/butyl). The resulting mixture was cooled to 0 ºC using an ice-water bath and bubbled with an argon balloon for 10 min. DIPEA (1.8 μL, 0.01 mmol, 5 mol%) and EtO_2CCH_2SH (1.1 μL, 0.01 mmol, 5 mol%) were then added. After that, the reactor was placed under a blue LED (Kessil light, 40 W, 456 nm) and irradiated for 12 hrs at room temperature. The solvent was removed under vacuum. Purification by flash column chromatography on silica gel (eluent: *n*-hexane/EtOAc mixtures = 100:1, R_f = 0.35) gave the desired product as a colorless oil, ^{1}H NMR (d1 = 25 s) and ^{2}H NMR spectrum were recorded.

The standard reaction was also conducted in deuterated THF, and no H/D exchange occurred.

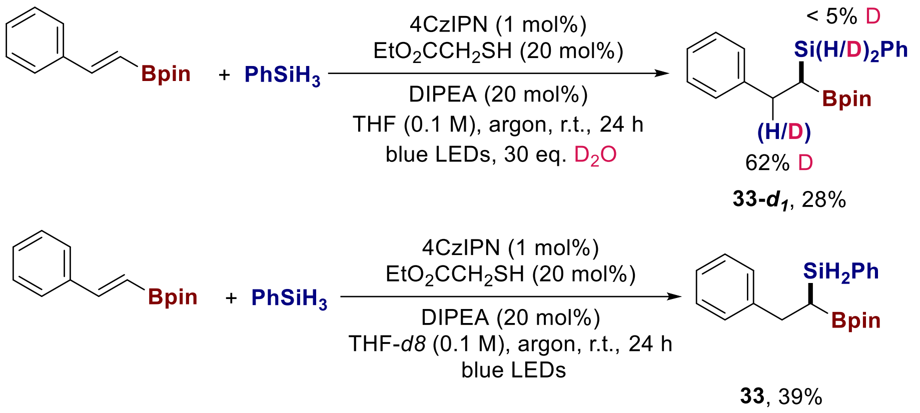

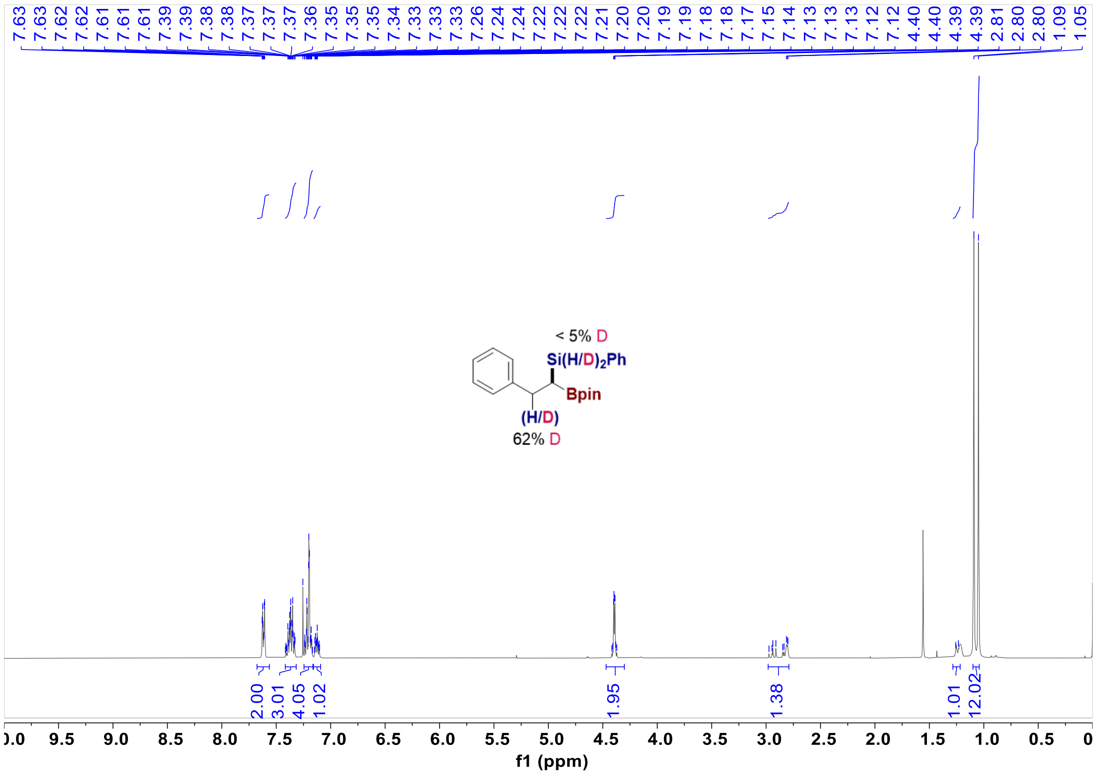

Supplementary Figure 11. ^{1}H NMR spectra of **33-*d*1**

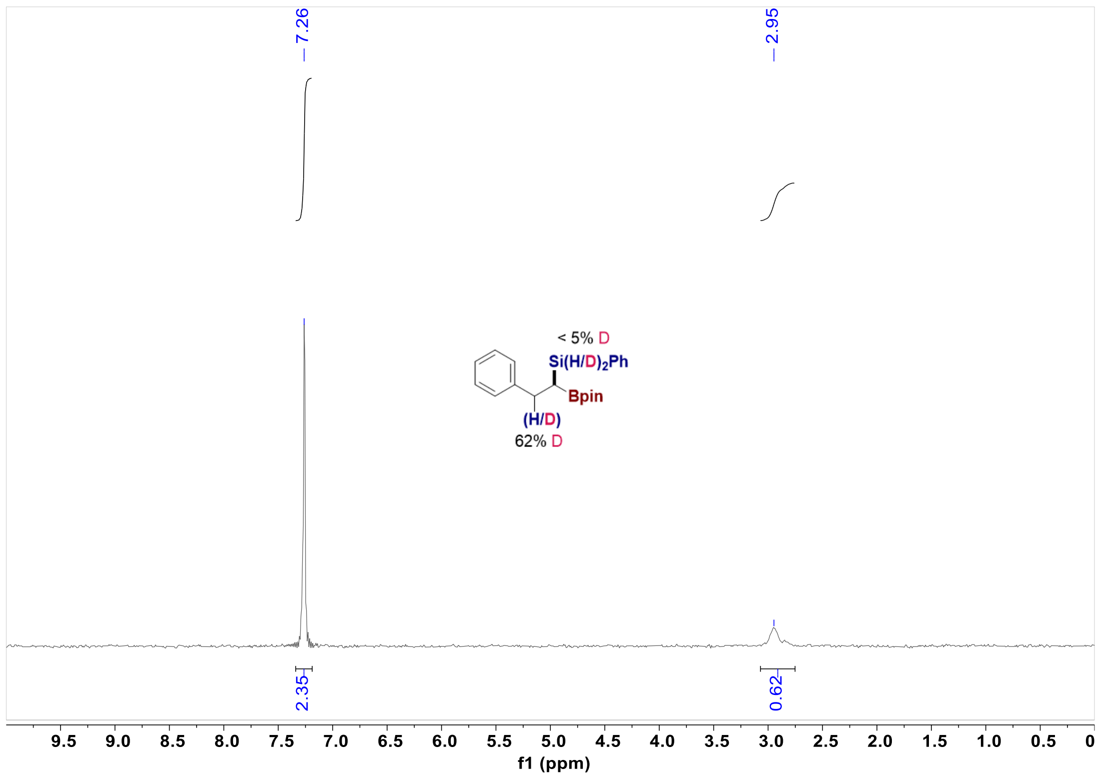

Supplementary Figure 12. ^{2}H NMR spectra of **33-*d*1**. $CDCl_3$ (2.35 equiv.) was used as an internal standard

A 10 mL microwave tube equipped with a magnetic stir bar was charged with 4CzIPN (1.6 mg, 0.002 mmol, 1 mol%), alkenyl boronate (46 mg, 0.2 mmol), $PhSiH_3$ (30 μL, 0.24 mmol, 1.2 equiv.), anhydrous THF (2 mL) and D_2O (110 μL, 6 mmol, 30 eq.). The tube was capped with a Supelco aluminum crimp seal with septum (PTFE/butyl). The resulting mixture was cooled to 0 ºC using an ice-water bath and bubbled with an argon balloon for 10 min. DIPEA (7.2 μL, 0.04 mmol, 20 mol%) and EtO_2CCH_2SH (4.4 μL, 0.04 mmol, 20 mol%) were then added. After that, the reactor was placed under blue LED (Kessil light, 80 W, 456 nm) and irradiated for 24 hrs at room temperature. The solvent was removed under vacuum. Purification by flash column chromatography on silica gel (eluent: *n*-hexane/EtOAc mixtures = 100:1, R_f = 0.3) gave the desired product as a colorless oil, ^{1}H NMR (d1 = 25 s) and ^{2}H NMR spectrum were recorded.

The standard reaction was also conducted in deuterated THF, and no H/D exchange occurred.

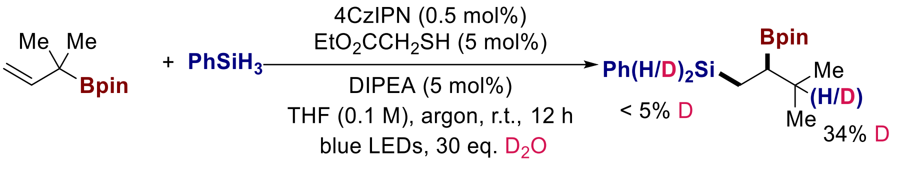

63-*d1*, 60%

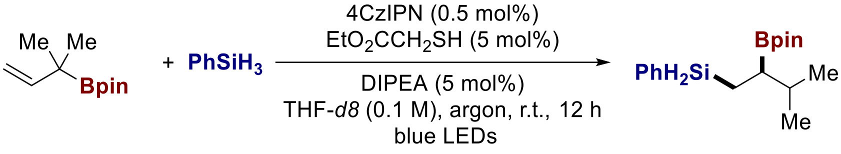

63, 75%

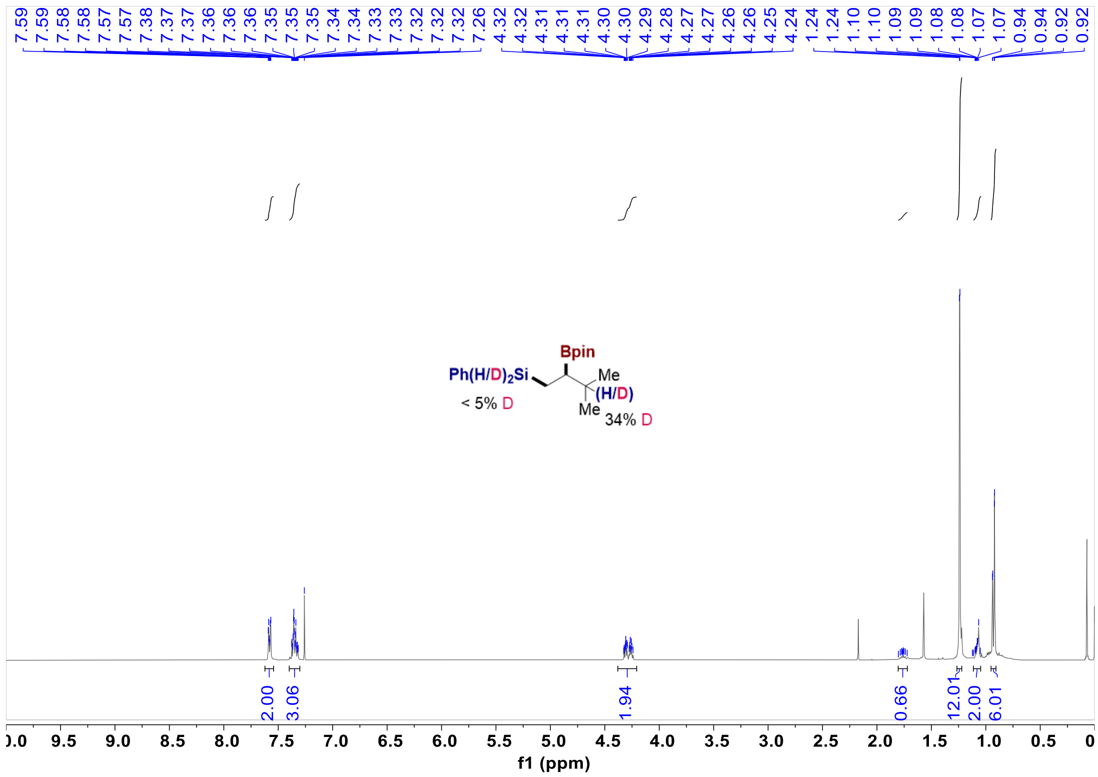

Supplementary Figure 13. ^{1}H NMR spectra of **63-*d*1**

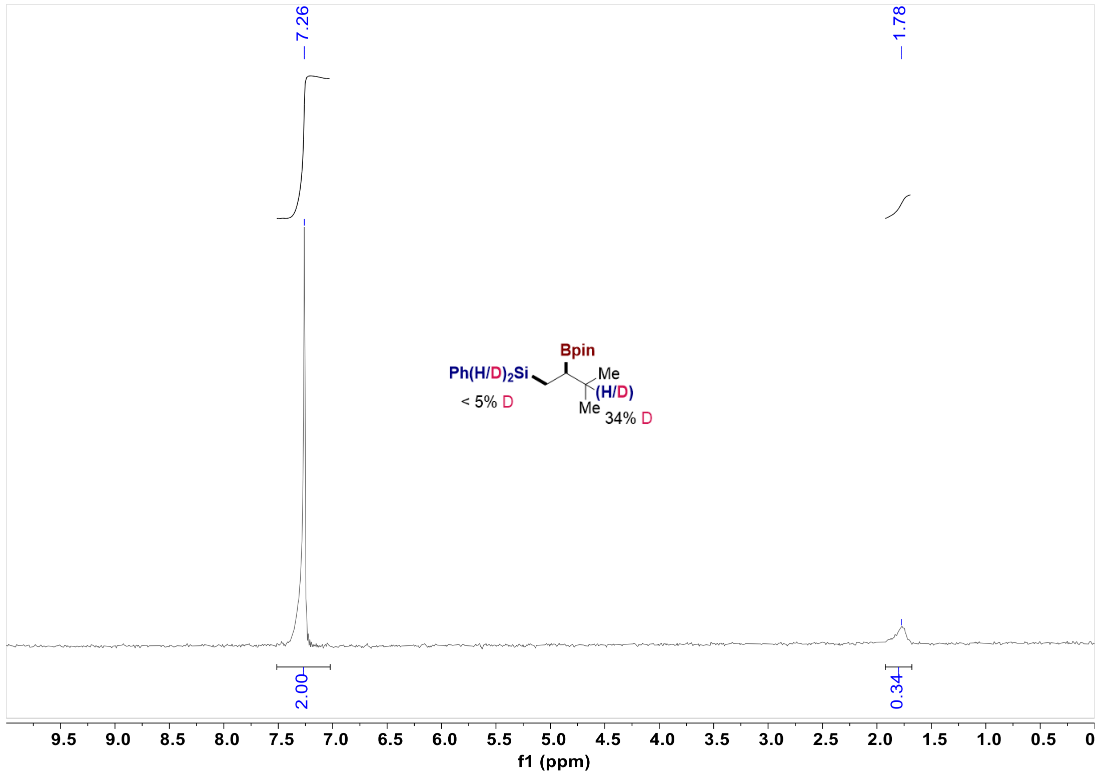

Supplementary Figure 14. ^{2}H NMR spectra of **63-*d*1**. $CDCl_3$ (2 equiv.) was used as an internal standard

A 10 mL microwave tube equipped with a magnetic stir bar was charged with 4CzIPN (0.8 mg, 0.001 mmol, 0.5 mol%), allyl boronate (39.2 mg, 0.2 mmol), $PhSiH_3$ (30 µL, 0.24 mmol, 1.2 equiv.), anhydrous THF (2 mL). The tube was capped with a Supelco aluminum crimp seal with septum (PTFE/butyl). The resulting mixture was cooled to 0 ºC using an ice-water bath and bubbled with an argon balloon for 10 min. DIPEA (1.8 µL, 0.01 mmol, 5 mol%) and EtO_2CCH_2SH (1.1 µL, 0.01 mmol, 5 mol%) were then added. After that, the reactor was placed under blue LED (Kessil light, 40 W, 456 nm) and irradiated for 12 hrs at room temperature. The solvent was removed under vacuum. Purification by flash column chromatography on silica gel (eluent: *n*-hexane/EtOAc mixtures = 100:1, R_f = 0.35) gave the desired product as a colorless oil, ^{1}H NMR (d1 = 25 s) and ^{2}H NMR spectrum were recorded.

The standard reaction was also conducted in deuterated THF, and no H/D exchange occurred.

Light On-off Experiments

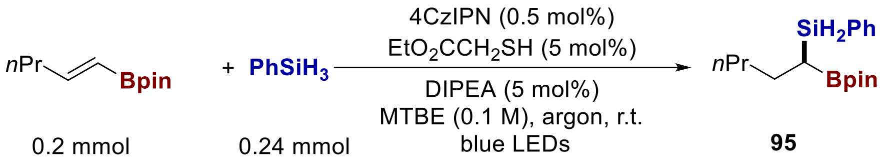

95

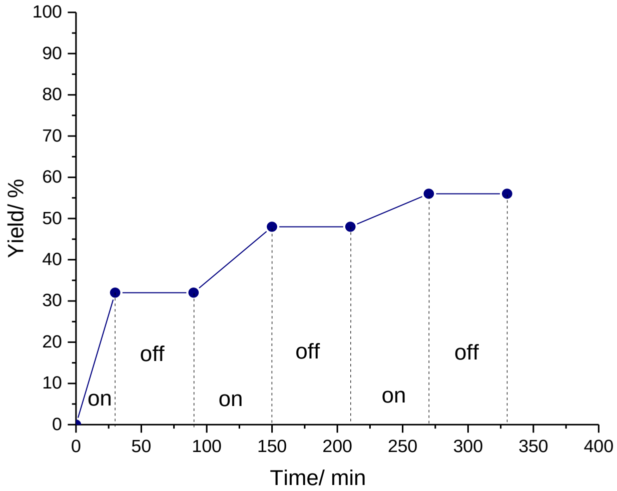

Supplementary Figure 15. Time profile of the hydrosilylation with the light ON/OFF over time. Yields were determined by crude ^{1}H NMR spectra using dibromomethane as an internal standard.

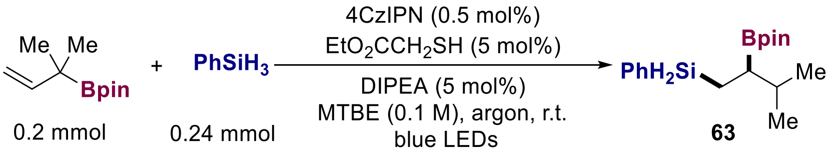

63

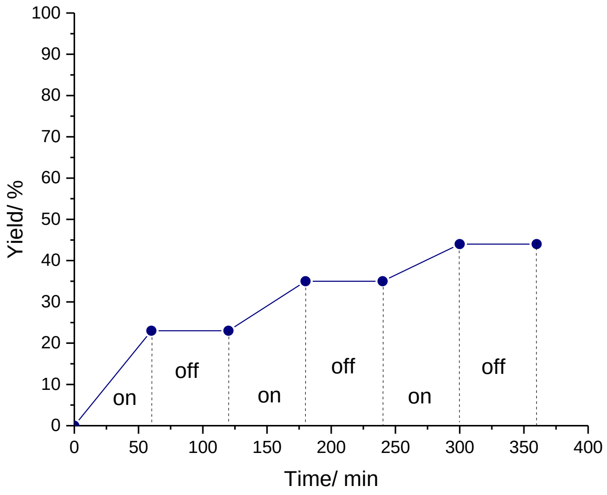

Supplementary Figure 16. Time profile of the hydrosilylation with the light ON/OFF over time. Yields were determined by crude ^{1}H NMR spectra using dibromomethane as an internal standard.

Determination of Photochemical Quantum Yields

Follow McMullen's procedure for photon flux[15], the following solutions were prepared ahead of time:

1. Ferrioxalate solution

A 0.15 M solution of potassium ferrioxolate was prepared by dissolving potassium ferrioxolate ($K_3Fe(C_2O_4)_3*3H_2O$) (1.842 g, 3.75 mmol) with the 0.05 M sulfuric acid solution prepared in a 25 mL volumetric flask. Make every precaution to prepare and store the solution in the dark.

2. Developer solution

67.8 g of sodium acetate was dissolved in 500 ml of 0.5 M sulfuric acid. 5 g of 1,10-phenanthroline was added to this solution. Store in the dark.

To determine the photon flux of the Kessil lamp, 2.0 mL of the ferrioxalate solution was placed in a 10 mL microwave tube and irradiated at λ = 456 nm with an emission slit width of 10.0 nm. After irradiation, 10 μL aliquots of the solution were taken at different time points between 0.5 and 3 minutes of irradiation. This aliquot is immediately added to 5 mL of the developer solution and the flask is wrapped in aluminum foil. A blank sample is prepared by adding 10 μL of the ferrioxalate solution to 5 mL of developer solution. The solutions were left in the dark for one hour, eventually becoming bright red. Solutions were transferred to a separate cuvette and the absorbance spectrum of the $Fe(phen)_3^{2+}$ complex was obtained. The absorbance at 510 nm ($\varepsilon = 11{,}100\ M^{-1}\ cm^{-1}$) was measured for each sample. The conversion was calculated using **eq 1**.

$$\text{mol Fe}^{2+} = \frac{V1 \cdot V3 \cdot \Delta A}{V2 \cdot l \cdot \varepsilon} \qquad \textbf{eq 1}$$

ΔA = the difference between the absorbance between the sample and the blank as measured at 510 nm.

l = the path length of the cuvette (1 cm)

ε = the extinction coefficient of $Fe(phen)_3^{2+}$ complex at 510 nm ($11{,}100\ M^{-1}\ cm^{-1}$)

V1 = the total volume of the irradiated solution (2 mL; 2×10^{-3} L)

V2 = the volume of the aliquot removed from solution (10 μL; 1×10^{-5} L)

V3 = the volume that aliquots are diluted with (5 mL; 5×10^{-3} L)

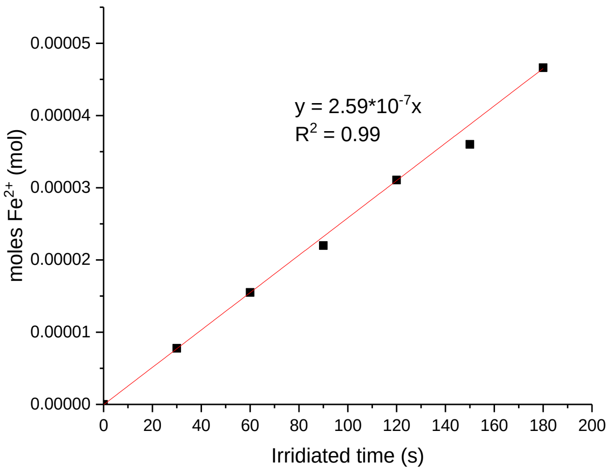

Supplementary Figure 17. Compiled linear fits for the photon flux

A plot of moles Fe^{2+} as a function of time yields a linear equation with an intercept at zero. The value of the slopes collected is 2.59 x 10^{-7} mol^{-1} s^{-1}.

The photon flux can be calculated using **eq 2**.

$$\text{Photon flux} = \frac{\text{mol Fe}^{2+}}{\Phi \cdot t \cdot f} \qquad \textbf{eq 2}$$

The documented quantum yield of the actinometer (Φ = 0.84 at 458 nm)[16] and f is the fraction of light absorbed at λ = 456 nm (0.95, vide infra)[17]. The photon flux in einsteins s^{-1}.

$$\text{Photon flux} = \frac{2.59 \times 10^{-7}}{0.84 \times 0.95} = 3.24 \times 10^{-7}$$

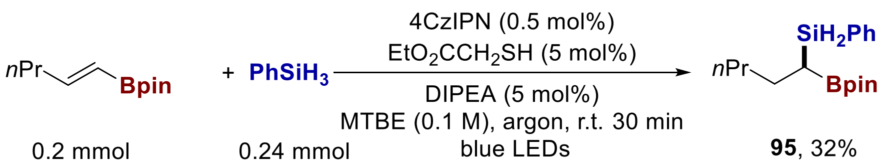

A 10 mL microwave tube equipped with a magnetic stir bar was charged with 4CzIPN (0.8 mg, 0.001 mmol, 0.5 mol%), alkenyl boronate (39.2 mg, 0.2 mmol), $PhSiH_3$ (30 μL, 0.24 mmol, 1.2 equiv.) and anhydrous MTBE (2 mL). The tube was capped with a Supelco aluminum crimp seal with septum (PTFE/butyl). The resulting mixture was cooled to 0 °C using an ice-water bath and bubbled with an argon balloon for 10 min. DIPEA (1.8 μL, 0.01 mmol, 5 mol%) and EtO_2CCH_2SH (1.1 μL, 0.01 mmol, 5 mol%) were then added. After that, the reactor was placed under a blue LED (Kessil light, 40 W, 456 nm) and irradiated for 30 min at room temperature. The solvent was removed

under vacuum. The yield of product formed was determined by crude ^{1}H NMR based on a CH_2Br_2 standard. The quantum yield was determined using **eq 3**. Essentially, all incident light (f = 1, vide infra) is absorbed by the 4CzIPN at the reaction conditions described above.

$$\Phi = \frac{\text{mol product}}{\text{flux} \cdot \text{t} \cdot \text{f}} \qquad \textbf{eq 3}$$

Experiment: alkenyl boronate (0.2 mmol), $PhSiH_3$ (0.24 mmol), 4CzIPN (0.001 mmol), EtO_2CCH_2SH (0.01 mmol) and DIPEA (0.01 mmol) in MTBE (2.0 mL) after 1800 s yielded 32% of **95**. Φ = 0.109.

$$\Phi = \frac{6.4 \times 10^{-5}}{3.24 \times 10^{-7} \times 1800 \times 1.00} = 0.109$$

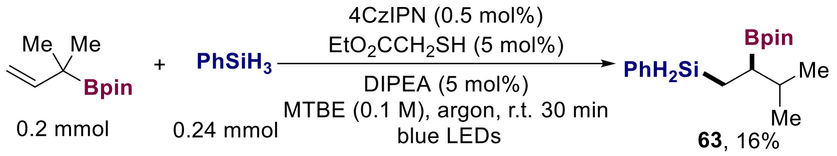

A 10 mL microwave tube equipped with a magnetic stir bar was charged with 4CzIPN (0.8 mg, 0.001 mmol, 0.5 mol%), allyl boronate (39.2 mg, 0.2 mmol), $PhSiH_3$ (30 μL, 0.24 mmol, 1.2 equiv.) and anhydrous MTBE (2 mL). The tube was capped with a Supelco aluminum crimp seal with septum (PTFE/butyl). The resulting mixture was cooled to 0 °C using an ice-water bath and bubbled with an argon balloon for 10 min. DIPEA (1.8 μL, 0.01 mmol, 5 mol%) and EtO_2CCH_2SH (1.1 μL, 0.01 mmol, 5 mol%) were then added. After that, the reactor was placed under a blue LED (Kessil light, 40 W, 456 nm) and irradiated for 30 min at room temperature. The solvent was removed under vacuum. The yield of product formed was determined by crude ^{1}H NMR based on a CH_2Br_2 standard. The quantum yield was determined using **eq 3**. Essentially, all incident light (f = 1, vide infra) is absorbed by the 4CzIPN at the reaction conditions described above.

$$\Phi = \frac{\text{mol product}}{\text{flux} \cdot \text{t} \cdot \text{f}} \qquad \textbf{eq 3}$$

Experiment: allyl boronate (0.2 mmol), $PhSiH_3$ (0.24 mmol), 4CzIPN (0.001 mmol), EtO_2CCH_2SH (0.01 mmol) and DIPEA (0.01 mmol) in MTBE (2.0 mL) after 1800 s yielded 16% of **63**. Φ = 0.055.

$$\Phi = \frac{3.2 \times 10^{-5}}{3.24 \times 10^{-7} \times 1800 \times 1.00} = 0.055$$

Proposed Mechanisms

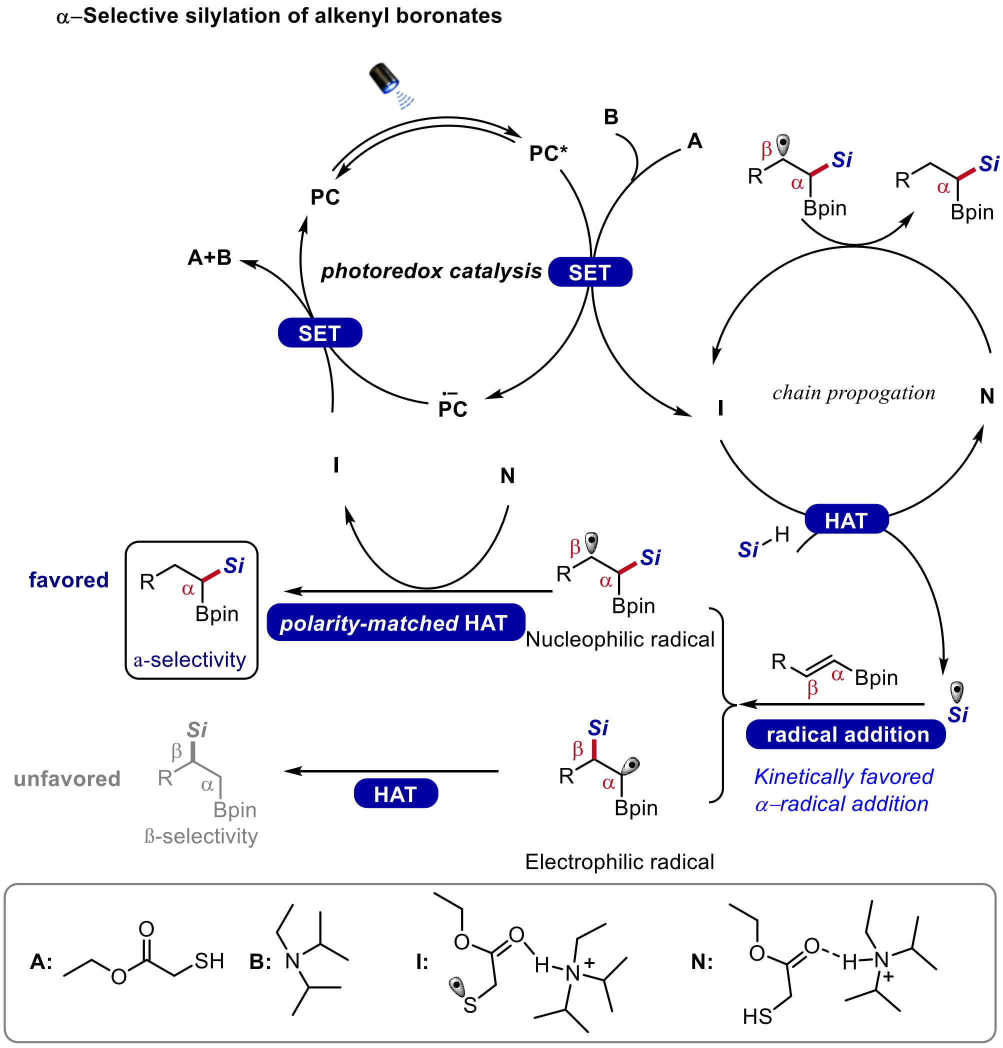

Supplementary Figure 18. Proposed mechanism for synthesis of geminal borosilanes

γ–Selective silylation of allyl boronates with concomitant 1,2-boron shift

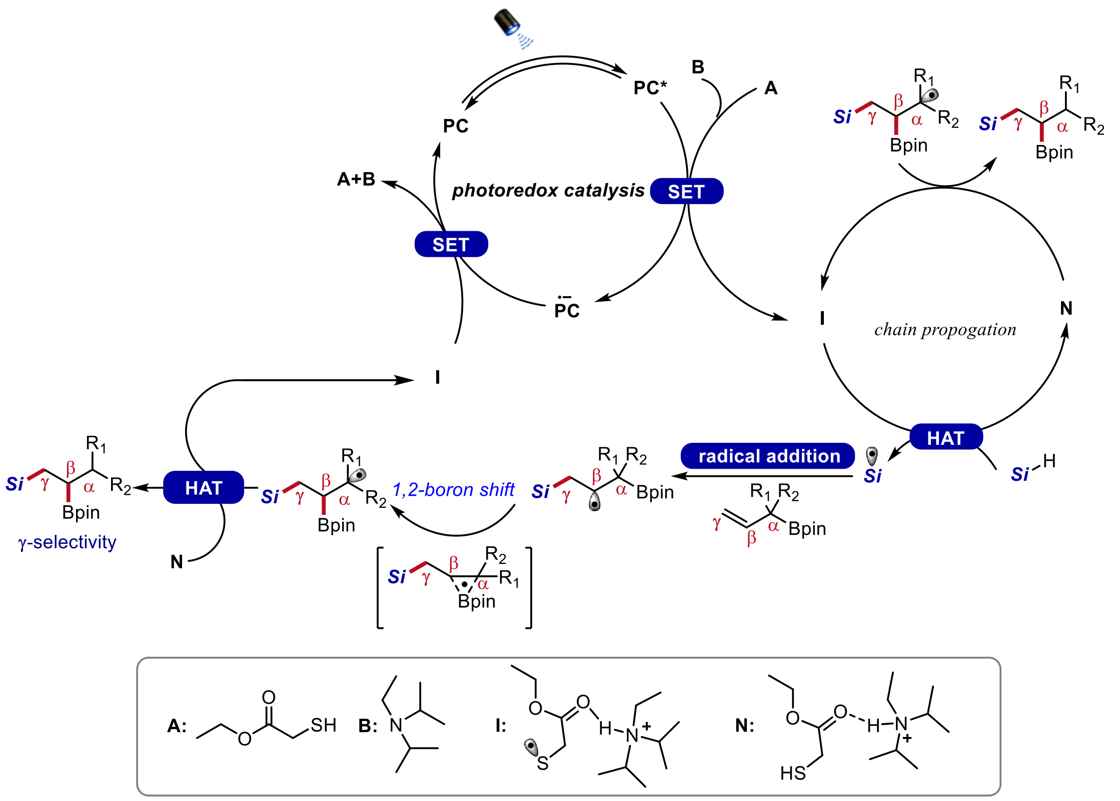

Supplementary Figure 19. Proposed mechanism for synthesis of vicinal borosilanes

Computational Details

All quantum chemical calculations were performed by the Gaussian 16 program suite[18]. Geometry optimizations and frequency analyses were carried out using M06-2X functional[19] augmented with Grimme's D3 dispersion correction[20]. The 6-31G(d,p) basis set[21-26] was used for all atoms. IEFPCM implicit solvation model[27,28] was used to account for the solvation effects (static dielectric constant and dynamic dielectric constant were manually set as 2.6 and 1.874 respectively). Possible conformations are searched at all local minimum and transition states structure in reaction for the corresponding minimum energetic pathway. All optimized geometries were confirmed by the frequency analyses while transition states were further confirmed by the intrinsic reaction coordinate (IRC) calculation. More accurate single point energies were calculated by higher level basis set that may-cc-pVTZ[29-31] for all atoms, without changing any other conditions. Thermal corrections at 298.15 K were calculated by the Shermo 2.3 program[32] with Grimme's quasi- rigid-rotor harmonic oscillator model[33]. Solvation free energies were corrected to concentration of 1 mol·L^{-1} by adding +1.89 kcal·mol^{-1} to all species. The energy barrier of SET processes was calculated by Marcus Theory[35]. External reorganization energies were obtained via non-equilibrium solvation model. All electronic structure analyses were performed using the Multiwfn 3.8 (dev) program[35]. Structures were visualized by VMD program[36].

DFT Calculations of Photoredox Catalysis Cycle

The photoredox catalytic cycle wass divided into three sections: (i) a SET process between the excited state of 4-CzIPN and ethyl thioglycolate, (ii) a HAT process between the thiyl radical and silane, (iii) a SET process for the regeneration process of 4-CzIPN. The thermally-activated delayed fluorescence (TADF) molecules with a small energy gap between $\mathbf{S_1}$ and $\mathbf{T_1}$ state (ΔEST) have found broad applications in photoredox catalysis and organic optoelectronic materials[37-39]. Previous research on such excited TADF-type photocatalysts has indicated that the SET step mainly occurred

in the **T_1** state since the population of **T_1** state is fast[40]. Energetic barriers of the SET step between 4-CzIPN and ethyl thioglycolate were calculated using Marcus theory[34]. The results are consistent with the previous reports and are shown in **Supplementary Figures 20 and 21**. The interaction between ethyl thioglycolate and DIPEA could decrease the energy barrier for single electron oxidation by the excited photocatalyst and also stabilize the formed radical cation intermediate (**H** vs. **I**) (**Supplementary Figure 20**). The energy barriers for accessing radical cation **H** or neutral radical **P** were calculated to be +40.31 kcal·mol^{-1} (**I → B + H**) and +5.01 kcal·mol^{-1} (**I → P + O**), respectively. Spin population analysis showed that spin density of the radical cations **H** and **I** were localized on the sulfur atom (**Supplementary Figure 22**)[41]. Next, it was found that the energy barrier for hydrogen atom abstraction from phenylsilane by the radical cation species **I** is significantly lower than by radical **P** (ΔΔG = 6.81 kcal·mol^{-1})(**G + L/G + K**). Finally, the SET event between the reduced photocatalyst and thiyl radical **I** for photocatalyst regeneration was also found to benefit from the complexation between thiyl radical and DIPEA. Overall, these results indicate that the complexation could promote both SET and HAT processes.

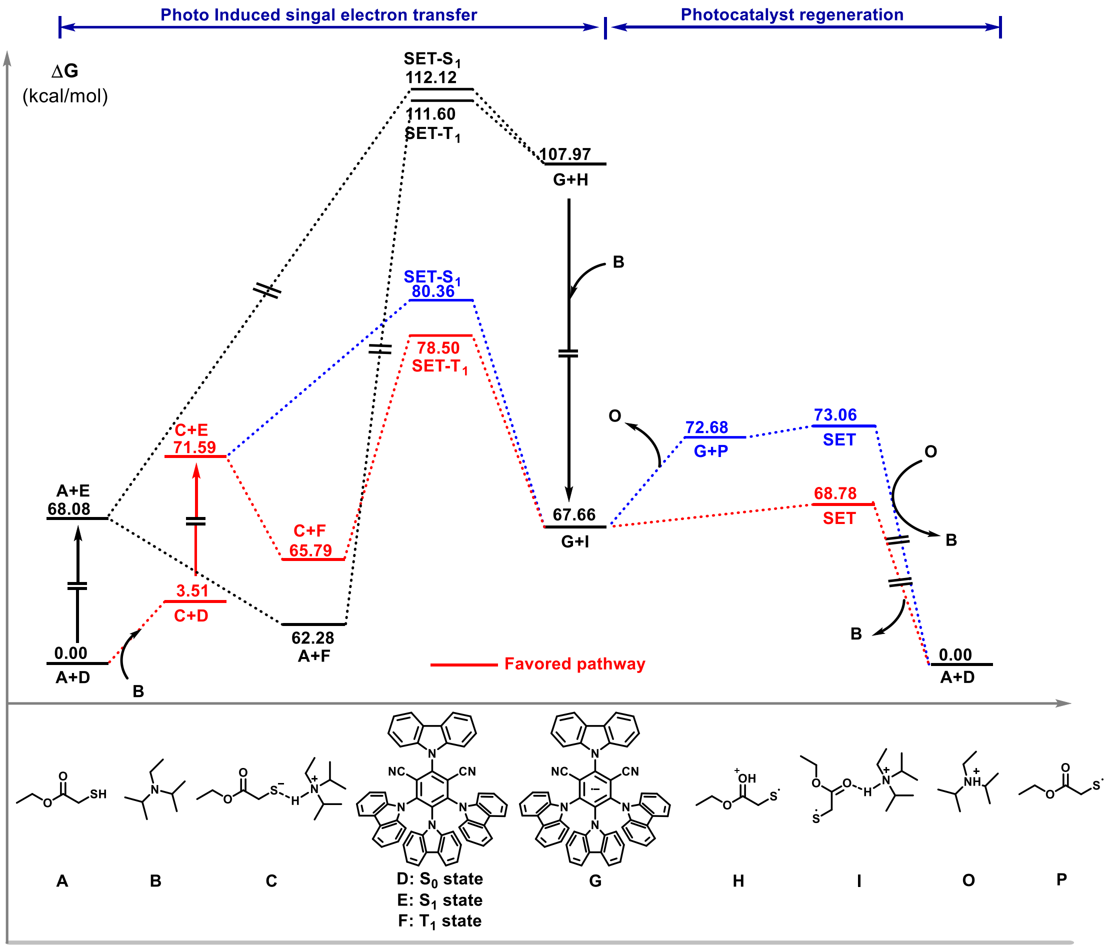

Supplementary Figure 20. Free energy diagram for steps in photocatalysis circle

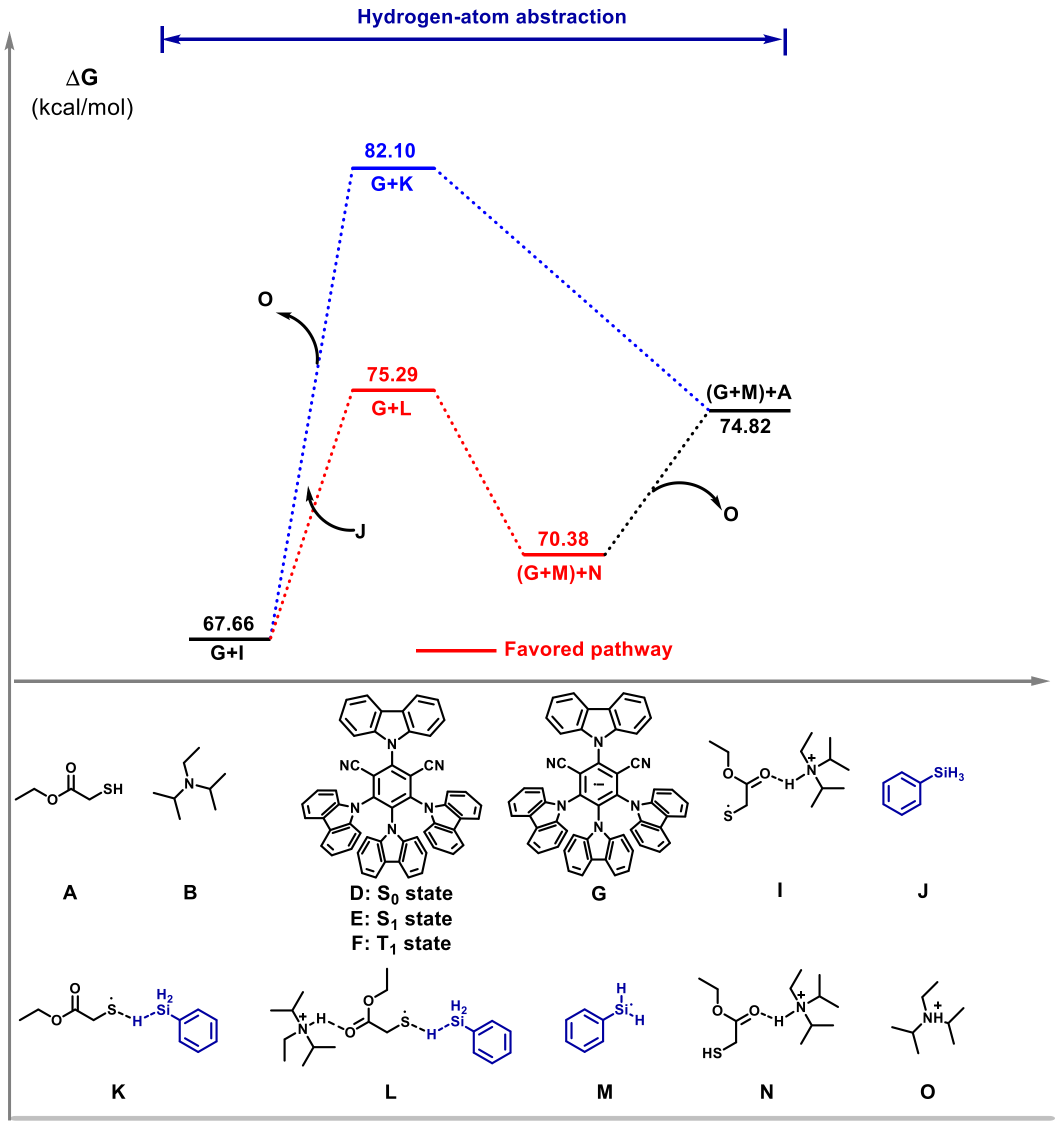

Supplementary Figure 21. Free energy diagram for hydrogen-atom transfer process

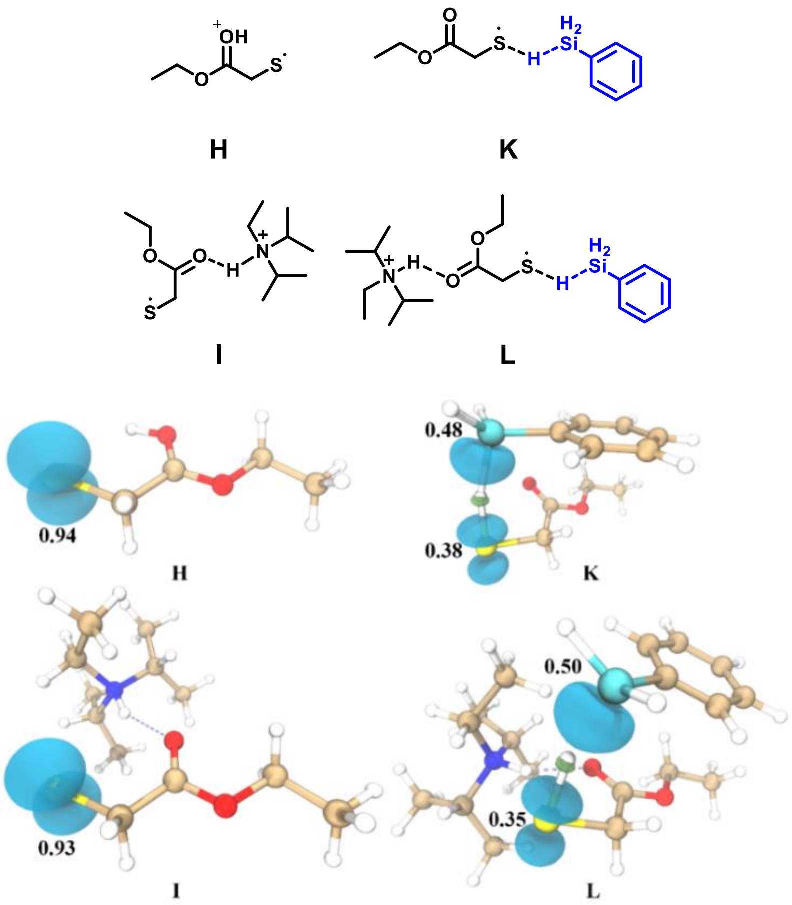

Supplementary Figure 22. Isosurface of spin density at 0.01 a.u. for S radicals **H**/**I** and transition states of hydrogen-atom abstraction **K**/**L**. Numbers are spin population from Hirshfeld atomic spaces analysis[41].

DFT Calculations of Radical Silylation of 1-Pentenylboronic Acid Pinacol Ester

The regioselectivity in the hydrosilylation of alkenyl boronates is studied by calculations. The addition of silyl radical and subsequent hydrogen atom transfer with thiols are calculated with *(E)*-1-pentenylboronic acid pinacol ester and phenylsilane as the model substates (**Supplementary Figure 23**). The calculated energy diagram illustrates that the addition of silyl radical to alkenyl boronic esters determines the regioselectivity because the transition states (**S1** or **S2**) have the highest energy in the reaction pathways. This also explains why similar regioselectivity was observed with different thiols (Supplementary Table 1). The energy barrier of silyl radical adding to α-position of **R1** is 1.64 kcal·mol-1 lower than that to β-position (**S1** vs **S2**), which means the α-addition rate is approximately 16 times faster than β-addition. This is very close to the observed selectivity in the crude reaction mixture (α/β = 14:1). Despite higher stability of the generated intermediate **T2** after β-addition, there are two reasons for the kinetic-controlled α-selectivity. The radical addition processes are nearly irreversible at room temperature, thus the equilibrium between α- and β-addition products cannot be reached. Besides, HAT from thiol **N** to the radical intermediate **T1** is both kinetically and thermodymically favored ($\Delta G^{\neq}$ = 11.71 kcal·mol-1, ΔG = -7.89 kcal·mol-1) due to polarity-match. The higher HAT rate of **T1** compared to **T2** further reduces the concentration of the radical **T1**. Overall, the kinetically favored radical addition and energetically favored back HAT process contribute to α-selective silylation of alkenyl boronates. Similar elucidation is also found for *cis*-alkenyl boronate **R2** (**Supplementary Figure 23**).

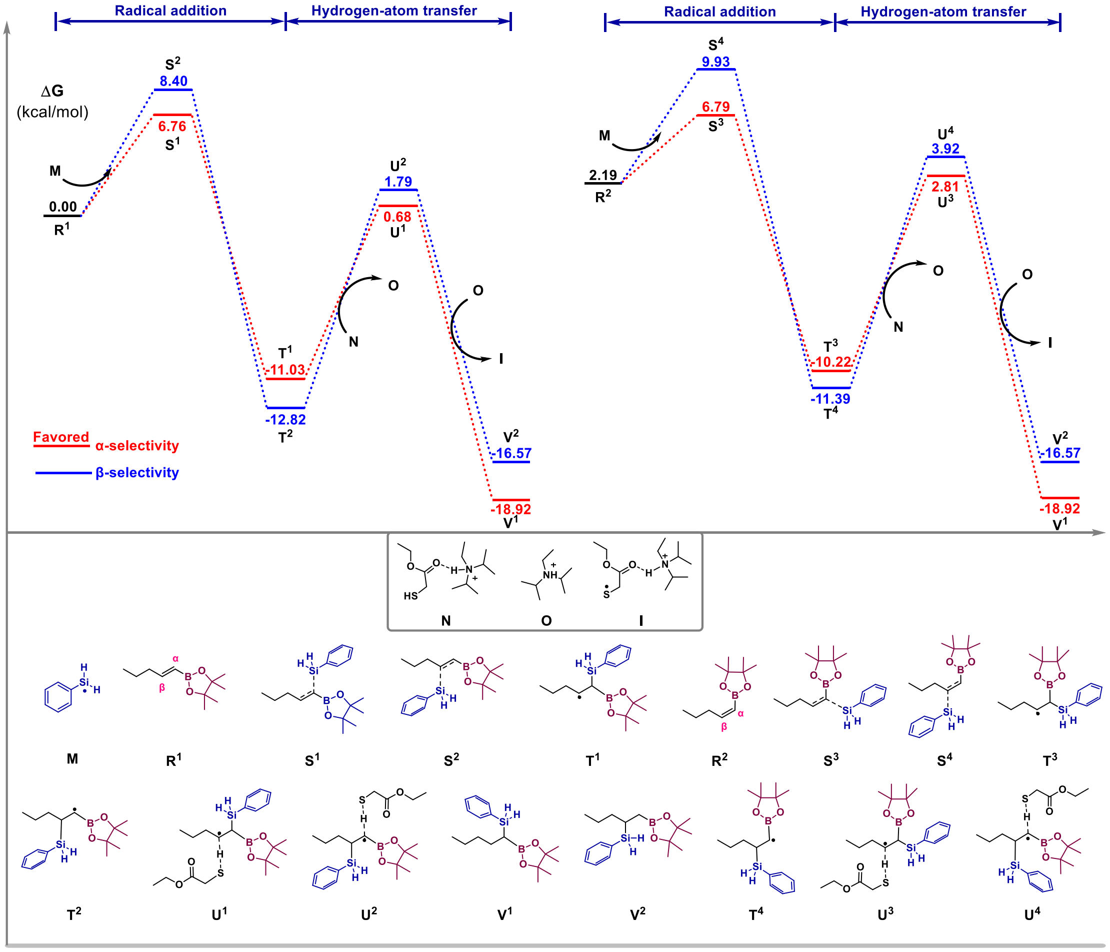

Supplementary Figure 23. Free energy diagram for hydrosilylation on 1-pentenylboronic acid pinacol ester

To examine whether different functionals give consistent results, we used 4 different types of functionals with great performance in benchmark studies (MN15-D3(BJ), PW6B95-D3, B3LYP-D3(BJ) and wB97XD) to study the α-selectivity in the silylation of alkenyl boronates. Consistent results were obtained with very similar ΔΔG values (**Supplementary Table 6**).

Supplementary Table 6. **Free Energy Under Different Methods**

	S^1	S^2	$\Delta\Delta G(S^1-S^2)$	T^1	T^2	$\Delta\Delta G(T^1-T^2)$
M06-2X-D3	6.76	8.40	-1.64	-11.03	-12.82	1.79
MN15-D3(BJ)	5.81	6.83	-1.02	-12.15	-14.33	2.18
PW6B95-D3	6.22	8.14	-1.92	-9.83	-11.57	1.74
B3LYP-D3(BJ)	5.05	7.57	-2.52	-9.18	-10.96	1.78
wB97XD	6.57	8.97	-2.40	13.30	-14.47	1.17
	U^1	U^2	$\Delta\Delta G(U^1-U^2)$	V^1	V^2	$\Delta\Delta G(V^1-V^2)$
M06-2X-D3	0.68	1.79	-1.11	-18.92	-16.57	-2.35
MN15-D3(BJ)	1.18	2.50	-1.32	-18.34	-16.24	-2.10
PW6B95-D3	0.74	2.53	-1.79	-16.70	-14.08	-2.62
B3LYP-D3(BJ)	-0.35	0.95	-1.30	-16.19	-13.72	-2.47
wB97XD	-3.60	-1.62	-1.98	-20.85	-18.26	-2.59

DFT Calculations of Radical Silylation of 1,1-Dimethyl-2-Propen-1-Boronic Acid Pinacol Ester

1,1-Dimethyl-2-propen-1-boronic acid pinacol ester and phenylsilane were chosen as the model substrates. Based on the free-energy diagram, the rate determining step is also the addition of silyl radical to allylboronic esters (**Supplementary Figure 24**). Free energy barrier for the addition of silyl radical to β-position of the allyl boronate is slightly higher than to γ-position of the allyl boronate ($\mathbf{S^6}$ vs. $\mathbf{S^5}$, ΔΔG = 1.83 kcal·mol^{-1}), probably due to steric effect. Besides, the radical intermediate $\mathbf{T^5}$ resulting from γ-addition is more stable than $\mathbf{T^6}$. At this stage, a 1,2-boron migration process influenced by the α-substituents on the allyl boronates took place. The migration was steered by thermodynamic effects to generate a more stable carbon radical **T7** which undergoes polarity-matched HAT process with thiol **N** to give vicinal borosilanes. DFT calculations indicate the migration energy barrier for α,α-dimethyl allyl boronate is low (ΔG≠ = 9.23 kcal·mol-1) and the rearranged radical intermediate **T7** is more stable than the non-migrated radical **T5** (ΔG = -1.69 kcal·mol-1). Moreover, the HAT reaction rate of rearranged radical **T7** with thiol is much faster compared to **T5**, thereby allowing selective synthesis of vicinal borosilanes. The spin delocalization of the transition state **W** is illustrated by spin population analysis (**Supplementary Figure 25**).

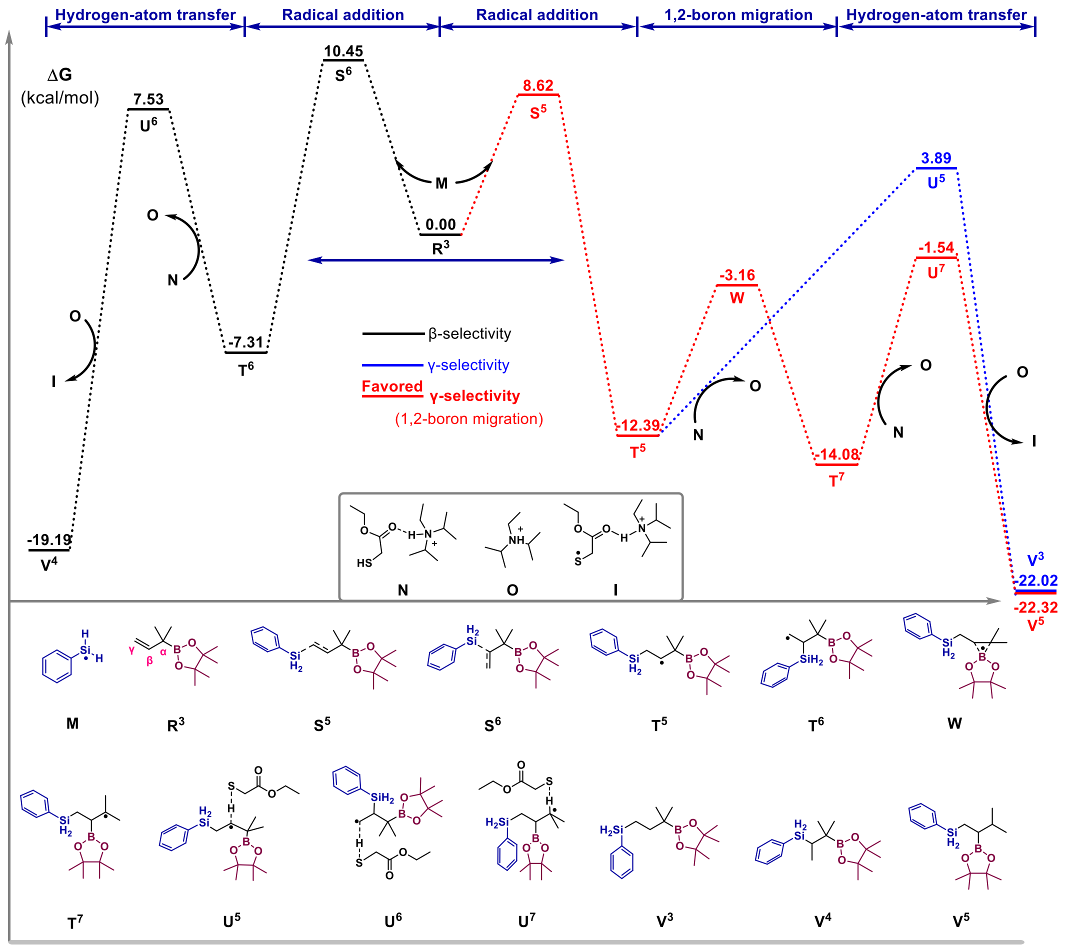

Supplementary Figure 24. Free energy diagram for hydrosilylation on 1,1-dimethyl-2-propen-1-boronic acid pinacol ester

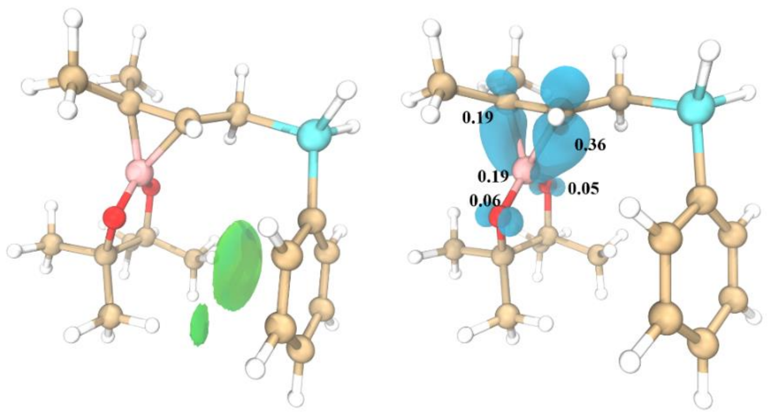

Supplementary Figure 25. Sign(λ_2)ρ colored isosurfaces of δg^{inter} = 0.005 a.u. of transition state **W** corresponding to IGMH analyses[42]. Isosurface of spin density at 0.01 a.u. for transition states **W.** Numbers are spin population from Hirshfeld atomic spaces analysis[40,41].

Supplementary Note 1

Analytical Data of the Products

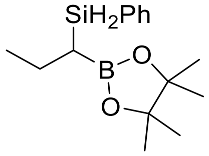

Phenyl(1-(4,4,5,5-tetramethyl-1,3,2-dioxaborolan-2-yl)propyl)silane (11). Following the general procedure I, the title compound (53.0 mg) was obtained in 96% yield. Colorless oil (eluent: hexane/EA = 100:1, R_f = 0.35). ^{1}H NMR (400 MHz, $CDCl_3$) δ 7.62 – 7.59 (m, 2H), 7.40 – 7.31 (m, 3H), 4.35 (qd, *J* = 6.4, 3.6 Hz, 2H), 1.74 – 1.62 (m, 1H), 1.62 – 1.49 (m, 1H), 1.19 (d, *J* = 11.3 Hz, 12H), 0.99 (t, *J* = 7.3 Hz, 3H), 0.75 (dq, *J* = 8.2, 4.0 Hz, 1H). ^{13}C NMR (126 MHz, $CDCl_3$) δ 135.54, 132.41, 129.53, 127.84, 83.03, 24.98, 24.50, 20.44, 17.40. The carbon signal attached to B was not observed. ^{11}B NMR (128 MHz, $CDCl_3$) δ 34.40. IR ν_{max} (DCM): 2977, 2958, 2929, 2126, 1371, 1267, 1145, 1118 cm^{-1}. HR-MS (EI) calcd for $C_{15}H_{24}BO_2Si$ [M-H]$^+$: 275.1633, found 275.1643.

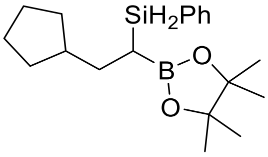

(2-Cyclopentyl-1-(4,4,5,5-tetramethyl-1,3,2-dioxaborolan-2-yl)ethyl)(phenyl)silane (12). Following the general procedure I, the title compound (40.3 mg) was obtained in 61% yield. Colorless oil (eluent: hexane/EA = 100:1, R_f = 0.35). ^{1}H NMR (500 MHz, $CDCl_3$) δ 7.64 – 7.58 (m, 2H), 7.41 – 7.30 (m, 3H), 4.34 (qd, *J* = 6.5, 3.6 Hz, 2H), 1.86 – 1.68 (m, 4H), 1.61 – 1.52 (m, 2H), 1.51 – 1.41 (m, 3H), 1.17 (d, *J* = 15.9 Hz, 12H), 1.11 – 0.99 (m, 2H), 0.91 – 0.86 (m, 1H). ^{13}C NMR (126 MHz, $CDCl_3$) δ 135.53, 132.40, 129.52, 127.83, 83.03, 42.80, 33.09, 32.68, 32.11, 25.19, 25.14, 24.91, 24.58. The carbon signal attached to B was not observed. ^{11}B NMR

(128 MHz, $CDCl_3$) δ 34.48. IR ν_{max} (DCM): 2977, 2948, 2866, 2129, 1350, 1311, 1144, 1117 cm^{-1}. HR-MS (EI) calcd for $C_{19}H_{30}BO_2Si$ $[M-H]^+$: 329.2103, found 329.2117.

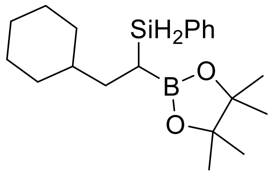

(2-Cyclohexyl-1-(4,4,5,5-tetramethyl-1,3,2-dioxaborolan-2-yl)ethyl)(phenyl)silane (13). Following the general procedure I, the title compound (43.4 mg) was obtained in 63% yield. Colorless oil (eluent: hexane/EA = 100:1, R_f = 0.35). ^1H NMR (400 MHz, $CDCl_3$) δ 7.54 – 7.52 (m, 2H), 7.32 – 7.24 (m, 3H), 4.26 (qd, *J* = 6.5, 3.6 Hz, 2H), 1.67 – 1.51 (m, 6H), 1.30 – 1.24 (m, 1H), 1.19 – 1.00 (m, 16H), 0.86 – 0.81 (m, 1H), 0.78 – 0.68 (m, 2H). ^{13}C NMR (126 MHz, $CDCl_3$) δ 135.52, 132.39, 129.52, 127.83, 83.02, 40.01, 34.35, 33.40, 32.55, 26.68, 26.38, 26.37, 24.91, 24.52. The carbon signal attached to B was not observed. ^{11}B NMR (128 MHz, $CDCl_3$) δ 34.78. IR ν_{max} (DCM): 2977, 2921, 2850, 2126, 1448, 1370, 1310, 1144 cm^{-1}. HR-MS (EI) calcd for $C_{20}H_{32}BO_2Si$ $[M-H]^+$: 343.2259, found 343.2267.

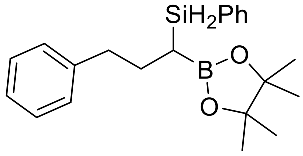

Phenyl(3-phenyl-1-(4,4,5,5-tetramethyl-1,3,2-dioxaborolan-2-yl)propyl)silane (14). Following the general procedure I, the title compound (56.4 mg) was obtained in 80% yield. Colorless oil (eluent: hexane/EA = 100:1, R_f = 0.3). ^1H NMR (400 MHz, $CDCl_3$) δ 7.52 – 7.47 (m, 2H), 7.31 – 7.22 (m, 3H), 7.19 – 7.15 (m, 2H), 7.10 – 7.04 (m, 3H), 4.28 (qd, *J* = 6.4, 3.6 Hz, 2H), 2.68 – 2.46 (m, 2H), 1.97 – 1.87 (m, 1H), 1.75 – 1.66 (m, 1H), 1.11 (d, *J* = 10.5 Hz, 12H), 0.83 – 0.77 (m, 1H). ^{13}C NMR (126 MHz, $CDCl_3$) δ 142.31, 135.55, 132.15, 129.57, 128.53, 128.24, 127.85, 125.70, 83.13, 38.85, 29.06, 25.02, 24.57. The carbon signal attached to B was not observed. ^{11}B NMR (128

MHz, $CDCl_3$) δ 34.86. IR ν_{max} (DCM): 2978, 2927, 2857, 2131, 1454, 1353, 1261, 1144 cm^{-1}. HR-MS (EI) calcd for $C_{21}H_{29}BO_2Si$ $[M]^+$: 352.2024, found 352.2029.

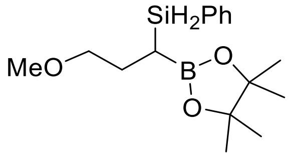

(3-Methoxy-1-(4,4,5,5-tetramethyl-1,3,2-dioxaborolan-2-yl)propyl)(phenyl)silane (15). Following the general procedure I, the title compound (49.6 mg) was obtained in 81% yield. Colorless oil (eluent: hexane/EA = 50:1, R_f = 0.25). ^1H NMR (400 MHz, $CDCl_3$) δ 7.62 – 7.59 (m, 2H), 7.40 – 7.31 (m, 3H), 4.36 (qd, *J* = 6.5, 3.7 Hz, 2H), 3.35 (td, *J* = 6.5, 1.1 Hz, 2H), 3.28 (s, 3H), 1.97 – 1.87 (m, 1H), 1.78 – 1.69 (m, 1H), 1.18 (d, *J* = 10.5 Hz, 12H), 0.87 – 0.81 (m, 1H). ^{13}C NMR (126 MHz, $CDCl_3$) δ 135.55, 132.10, 129.61, 127.87, 83.08, 74.41, 58.38, 26.91, 24.88, 24.54. The carbon signal attached to B was not observed. ^{11}B NMR (128 MHz, $CDCl_3$) δ 34.22. IR ν_{max} (DCM): 2974, 2927, 2855, 2126, 1651, 1351, 1261, 1144 cm^{-1}. HR-MS (EI) calcd for $C_{16}H_{26}BO_3Si$ $[M-H]^+$: 305.1739, found 305.1738.

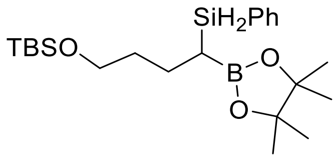

tert-Butyldimethyl(4-(phenylsilyl)-4-(4,4,5,5-tetramethyl-1,3,2-dioxaborolan-2-yl)butoxy)silane (16). Following the general procedure I, the title compound (79.8 mg) was obtained in 95% yield. Colorless oil (eluent: hexane/EA = 50:1, R_f = 0.2). ^1H NMR (400 MHz, $CDCl_3$) δ 7.61 – 7.59 (m, 2H), 7.39 – 7.31 (m, 3H), 4.35 (qd, *J* = 6.4, 3.6 Hz, 2H), 3.57 (td, *J* = 6.3, 1.6 Hz, 2H), 1.70 – 1.48 (m, 4H), 1.18 (d, *J* = 11.1 Hz, 12H), 0.87 (s, 9H), 0.84 – 0.76 (m, 1H), 0.02 (s, 6H). ^{13}C NMR (126 MHz, $CDCl_3$) δ 135.55, 132.25, 129.55, 127.85, 83.06, 62.96, 35.79, 25.99, 24.98, 24.53, 23.21, 18.36, -5.25. The carbon signal attached to B was not observed. ^{11}B NMR (128 MHz, $CDCl_3$) δ 34.03. IR ν_{max} (DCM): 2976, 2954, 2857, 2122, 1740, 1351, 1257, 1145 cm^{-1}. HR-MS (EI) calcd for $C_{22}H_{40}BO_3Si$ $[M-H]^+$: 419.2604, found 419.2613.

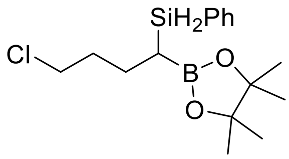

(4-Chloro-1-(4,4,5,5-tetramethyl-1,3,2-dioxaborolan-2-yl)butyl)(phenyl)silane (17). Following the general procedure I, the title compound (59.0 mg) was obtained in 91% yield. Colorless oil (eluent: hexane/EA = 50:1, R_f = 0.35). ^{1}H NMR (400 MHz, $CDCl_3$) δ 7.61 – 7.57 (m, 2H), 7.41 – 7.32 (m, 3H), 4.35 (qd, *J* = 6.4, 3.6 Hz, 2H), 3.49 (t, *J* = 6.7 Hz, 2H), 1.76 – 1.68 (m, 2H), 1.53 – 1.45 (m, 2H), 1.18 (d, *J* = 11.8 Hz, 12H), 0.85 – 0.77 (m, 1H). ^{13}C NMR (126 MHz, $CDCl_3$) δ 135.52, 132.10, 129.64, 127.90, 83.16, 44.99, 32.44, 29.92, 26.32, 24.99, 24.51. The carbon signal attached to B was not observed. ^{11}B NMR (128 MHz, $CDCl_3$) δ 34.19. IR ν_{max} (DCM): 2978, 2923, 2851, 2128, 1460, 1372, 1315, 1260, 1143 cm^{-1}. HR-MS (EI) calcd for $C_{16}H_{25}BClO_2Si$ [M-H]$^+$: 323.1400, found 323.1409.

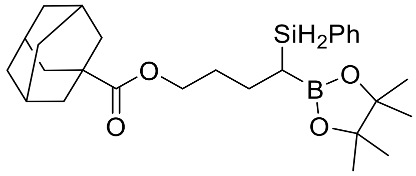

4-(Phenylsilyl)-4-(4,4,5,5-tetramethyl-1,3,2-dioxaborolan-2-yl)butyl adamantane-1-carboxylate (18). Following the general procedure I, the title compound (63.7 mg) was obtained in 68% yield. Colorless oil (eluent: hexane/EA = 50:1, R_f = 0.2). ^{1}H NMR (400 MHz, $CDCl_3$) δ 7.61 – 7.57 (m, 2H), 7.40 – 7.30 (m, 3H), 4.40 – 4.30 (m, 2H), 4.00 (t, *J* = 6.0 Hz, 2H), 2.01 – 1.96 (m, 3H), 1.87 – 1.80 (m, 6H), 1.74 – 1.62 (m, 9H), 1.60 – 1.51 (m, 1H), 1.18 (d, *J* = 10.2 Hz, 12H), 0.84 – 0.77 (m, 1H). ^{13}C NMR (126 MHz, $CDCl_3$) δ 177.67, 135.52, 132.01, 129.63, 127.95, 127.87, 83.15, 63.70, 40.67, 38.81, 36.54, 31.49, 27.99, 25.01, 24.51, 23.34. The carbon signal attached to B was not observed. ^{11}B NMR (128 MHz, $CDCl_3$) δ 33.66. IR ν_{max} (DCM): 2977, 2907, 2852, 2131, 1726, 1429, 1353, 1236, 1144 cm^{-1}. HR-MS (EI) calcd for $C_{27}H_{40}BO_4Si$ [M-H]$^+$: 467.2783, found 467.2787.

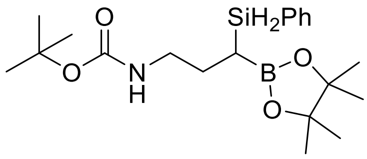

tert-Butyl (3-(phenylsilyl)-3-(4,4,5,5-tetramethyl-1,3,2-dioxaborolan-2-yl)propyl)carbamate (19). Following the general procedure I, the title compound (49.3 mg) was obtained in 63% yield. Colorless oil (eluent: hexane/EA = 10:1, R_f = 0.35). ^{1}H NMR (400 MHz, $CDCl_3$) δ 7.60 – 7.58 (m, 2H), 7.41 – 7.32 (m, 3H), 4.71 (brs, 1H), 4.39 – 4.32 (m, 2H), 3.21 – 3.10 (m, 2H), 1.85 – 1.75 (m, 1H), 1.71 – 1.64 (m, 1H), 1.42 (s, 9H), 1.19 (d, *J* = 1.4 Hz, 12H), 0.79 (dt, *J* = 11.1, 3.7 Hz, 1H). ^{13}C NMR (126 MHz, $CDCl_3$) δ 155.89, 135.53, 131.71, 129.76, 127.95, 83.40, 78.94, 42.92, 28.43, 26.89, 24.85, 24.72. The carbon signal attached to B was not observed. ^{11}B NMR (128 MHz, $CDCl_3$) δ 34.99. IR ν_{max} (DCM): 3420, 2978, 2930, 2131, 1770, 1652, 1365, 1247, 1143 cm^{-1}. HR-MS (APCI) calcd for $C_{20}H_{35}BNO_4Si$ $[M+H]^+$: 392.2423, found 392.2414.

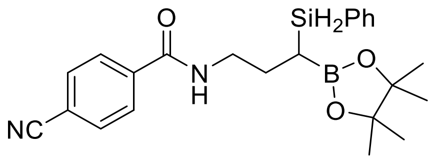

4-Cyano-N-(3-(phenylsilyl)-3-(4,4,5,5-tetramethyl-1,3,2-dioxaborolan-2-yl)propyl)benzamide (20). Following the general procedure I, the title compound (47.1 mg) was obtained in 56% yield. Colorless oil (eluent: hexane/EA = 10:1, R_f = 0.3). ^{1}H NMR (500 MHz, $CDCl_3$) δ 7.86 – 7.77 (m, 2H), 7.73 – 7.67 (m, 2H), 7.62 – 7.57 (m, 2H), 7.43 – 7.31 (m, 3H), 6.58 (t, *J* = 5.6 Hz, 1H), 4.45 – 4.34 (m, 2H), 3.57 – 3.37 (m, 2H), 1.97 – 1.79 (m, 2H), 1.15 (d, *J* = 2.1 Hz, 12H), 0.93 – 0.85 (m, 1H). ^{13}C NMR (126 MHz, $CDCl_3$) δ 165.64, 138.85, 135.53, 132.43, 132.35, 129.92, 128.05, 127.70, 118.08, 114.87, 83.63, 42.43, 26.43, 24.82, 24.67. The carbon signal attached to B was not observed. ^{11}B NMR (128 MHz, $CDCl_3$) δ 35.08. IR ν_{max} (DCM): 3440, 2131, 1652, 1312, 1261, 1142 cm^{-1}. HR-MS (EI) calcd for $C_{23}H_{28}BN_2O_3Si$ $[M-H]^+$: 419.1957, found 419.1951.

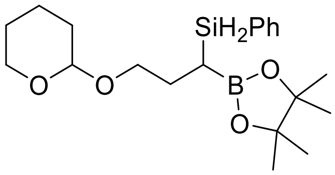

Phenyl(3-((tetrahydro-2H-pyran-2-yl)oxy)-1-(4,4,5,5-tetramethyl-1,3,2-dioxaborolan-2-yl)propyl)silane (21). Following the general procedure I, the title compound (64.0 mg) was obtained in 85% yield. d.r. = 1.2:1. Colorless oil (eluent: hexane/EA = 50:1, R_f = 0.3). ^{1}H NMR (400 MHz, $CDCl_3$) δ 7.63 – 7.59 (m, 2H), 7.39 – 7.31 (m, 3H), 4.56 – 4.53 (m, 1H), 4.37 (qd, *J* = 6.6, 3.6 Hz, 2H), 3.87 – 3.63 (m, 2H), 3.47 – 3.29 (m, 2H), 1.99 – 1.90 (m, 1H), 1.84 – 1.77 (m, 2H), 1.70 – 1.64 (m, 1H), 1.58 – 1.47 (m, 4H), 1.19 – 1.15 (m, 12H), 0.96 – 0.89 (m, 1H). ^{13}C NMR (126 MHz, $CDCl_3$) δ 135.58, 132.10, 129.61, 127.87, 98.74, 98.43, 83.14, 69.03, 68.94, 62.05, 61.97, 30.69, 30.63, 27.03, 26.99, 25.52, 25.00, 24.88, 24.51, 19.44, 19.42. The carbon signal attached to B was not observed. ^{11}B NMR (128 MHz, $CDCl_3$) δ 34.81. IR ν_{max} (DCM): 2976, 2941, 2870, 2129, 1653, 1353, 1262, 1144 cm^{-1}. HR-MS (EI) calcd for $C_{20}H_{32}BO_4Si$ [M-H]$^+$: 375.2157, found 375.2166.

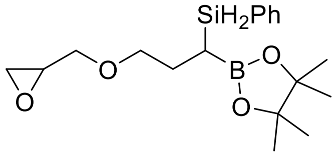

(3-(Oxiran-2-ylmethoxy)-1-(4,4,5,5-tetramethyl-1,3,2-dioxaborolan-2-yl)propyl)(phenyl)silane (22). Following the general procedure I, the title compound (31.3 mg) was obtained in 45% yield. d.r. = 1.1:1. Colorless oil (eluent: hexane/EA = 50:1, R_f = 0.3). ^{1}H NMR (400 MHz, $CDCl_3$) δ 7.62 – 7.59 (m, 2H), 7.40 – 7.31 (m, 3H), 4.36 (qd, *J* = 6.5, 3.6 Hz, 2H), 3.65 – 3.61 (m, 1H), 3.51 – 3.42 (m, 2H), 3.36 (ddd, *J* = 11.6, 5.7, 2.2 Hz, 1H), 3.14 – 3.08 (m, 1H), 2.78 – 2.75 (m, 1H), 2.59 – 2.56 (m, 1H), 1.98 – 1.89 (m, 1H), 1.81 – 1.72 (m, 1H), 1.17 (d, *J* = 10.6 Hz, 12H), 0.88 – 0.84 (m, 1H). ^{13}C NMR (126 MHz, $CDCl_3$) δ 135.56, 132.03, 129.66, 127.89, 83.15, 73.12, 71.40, 71.37, 50.85, 44.42, 26.93, 24.94, 24.53. The carbon signal attached to B was

not observed. ^{11}B NMR (128 MHz, $CDCl_3$) δ 34.65. IR ν_{max} (DCM): 2977, 2926, 2867, 2126, 1640, 1352, 1308, 1144 cm^{-1}. HR-MS (EI) calcd for $C_{18}H_{28}BO_4Si$ $[M-H]^+$: 347.1844, found 347.1836.

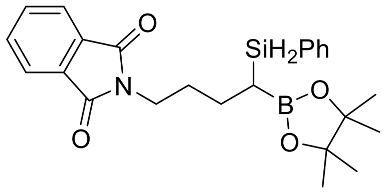

2-(4-(Phenylsilyl)-4-(4,4,5,5-tetramethyl-1,3,2-dioxaborolan-2-yl)butyl)isoindoline-1,3-dione (23). Following the general procedure I, the title compound (58.3 mg) was obtained in 67% yield. Colorless oil (eluent: hexane/EA = 20:1, R_f = 0.3). ^{1}H NMR (400 MHz, $CDCl_3$) δ 7.83 – 7.80 (m, 2H), 7.72 – 7.67 (m, 2H), 7.59 – 7.56 (m, 2H), 7.36 – 7.28 (m, 3H), 4.33 (qd, *J* = 6.5, 3.6 Hz, 2H), 3.66 – 3.60 (m, 2H), 1.81 – 1.52 (m, 4H), 1.15 (d, *J* = 11.2 Hz, 12H), 0.88 – 0.82 (m, 1H). ^{13}C NMR (126 MHz, $CDCl_3$) δ 168.35, 135.53, 133.77, 132.23, 131.94, 129.61, 127.86, 123.13, 83.18, 37.85, 31.34, 24.97, 24.46, 24.26. The carbon signal attached to B was not observed. ^{11}B NMR (128 MHz, $CDCl_3$) δ 34.48. IR ν_{max} (DCM): 2977, 2931, 2859, 2132, 1774, 1713, 1395, 1355, 1143 cm^{-1}. HR-MS (EI) calcd for $C_{24}H_{30}BNO_4Si$ $[M]^+$: 435.2032, found 435.2038.

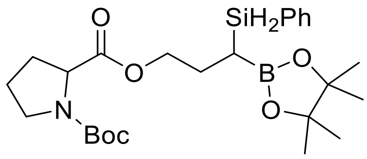

1-(tert-Butyl) 2-(3-(phenylsilyl)-3-(4,4,5,5-tetramethyl-1,3,2-dioxaborolan-2-yl)propyl) pyrrolidine-1,2-dicarboxylate (24). Following the general procedure I with racemic starting material, the title compound (85.1 mg) was obtained in 87% yield. d.r. = 1.8:1. Colorless oil (eluent: hexane/EA = 10:1, R_f = 0.25). ^{1}H NMR (400 MHz, $CDCl_3$) δ 7.54 – 7.49 (m, 2H), 7.36 – 7.23 (m, 3H), 4.35 – 4.25 (m, 2H), 4.25 – 3.91 (m, 3H), 3.52 – 3.23 (m, 2H), 2.18 – 2.00 (m, 1H), 1.96 – 1.69 (m, 5H), 1.38 – 1.31 (m, 9H), 1.11 (d, *J* = 6.4 Hz, 12H), 0.85 – 0.74 (m, 1H). ^{13}C NMR (101 MHz, $CDCl_3$) δ

173.19, 153.85, 135.52, 135.50, 131.49, 129.83, 127.98, 83.38, 79.83, 79.68, 66.17, 59.18, 58.85, 46.53, 46.32, 30.92, 28.45, 28.33, 25.98, 24.97, 24.54, 24.51, 24.28, 23.63. The carbon signal attached to B was not observed. ^{11}B NMR (128 MHz, $CDCl_3$) δ 35.45. IR ν_{max} (DCM): 2977, 2932, 2881, 2134, 1746, 1670, 1394, 1260, 1143 cm^{-1}. HR-MS (EI) calcd for $C_{25}H_{39}BNO_6Si$ $[M\text{-}H]^+$: 488.2634, found 488.2640.

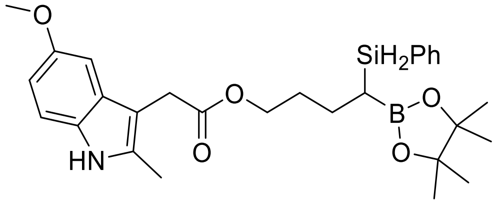

4-(Phenylsilyl)-4-(4,4,5,5-tetramethyl-1,3,2-dioxaborolan-2-yl)butyl 2-(5-methoxy-2-methyl-1H-indol-3-yl)acetate (25). Following the general procedure I, the title compound (65.9 mg) was obtained in 65% yield. Colorless oil (eluent: hexane/EA = 10:1, R_f = 0.25). ^{1}H NMR (500 MHz, $CDCl_3$) δ 7.69 (s, 1H), 7.59 – 7.56 (m, 2H), 7.41 – 7.32 (m, 3H), 7.14 – 7.12 (m, 1H), 7.00 – 6.97 (m, 1H), 6.78 – 6.75 (m, 1H), 4.34 – 4.29 (m, 2H), 4.02 (t, *J* = 6.2 Hz, 2H), 3.84 (s, 3H), 3.61 (d, *J* = 1.2 Hz, 2H), 2.36 (s, 3H), 1.77 – 1.62 (m, 3H), 1.55 – 1.49 (m, 1H), 1.16 (d, *J* = 16.3 Hz, 12H), 0.81 – 0.73 (m, 1H). ^{13}C NMR (126 MHz, $CDCl_3$) δ 171.99, 154.17, 135.54, 133.40, 132.01, 130.09, 129.66, 128.99, 127.90, 111.05, 110.87, 104.66, 100.36, 83.17, 64.52, 55.90, 31.45, 30.51, 24.97, 24.50, 23.41, 11.88. The carbon signal attached to B was not observed. ^{11}B NMR (160 MHz, $CDCl_3$) δ 33.78. IR ν_{max} (DCM): 2973, 2929, 2852, 2132, 1730, 1653, 1355, 1224, 1142 cm^{-1}. HR-MS (EI) calcd for $C_{28}H_{38}BNO_4Si$ $[M]^+$: 507.2607, found 507.2603.

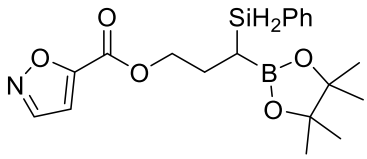

3-(Phenylsilyl)-3-(4,4,5,5-tetramethyl-1,3,2-dioxaborolan-2-yl)propyl isoxazole-5-carboxylate (26). Following the general procedure I, the title compound (51.1 mg) was obtained in 66% yield. Colorless oil (eluent: hexane/EA = 20:1, R_f = 0.3). ^{1}H NMR

(400 MHz, $CDCl_3$) δ 8.92 (d, *J* = 0.7 Hz, 1H), 8.47 (d, *J* = 0.7 Hz, 1H), 7.65 – 7.57 (m, 2H), 7.42 – 7.32 (m, 3H), 4.40 (qd, *J* = 6.6, 3.5 Hz, 2H), 4.35 – 4.25 (m, 2H), 2.13 – 2.03 (m, 1H), 1.98 – 1.89 (m, 1H), 1.18 (d, *J* = 5.7 Hz, 12H), 0.98 – 0.90 (m, 1H). ^{13}C NMR (101 MHz, $CDCl_3$) δ 161.15, 157.94, 148.84, 135.51, 131.46, 129.88, 128.02, 83.46, 67.02, 25.94, 24.96, 24.54. The carbon signal attached to B was not observed. ^{11}B NMR (128 MHz, $CDCl_3$) δ 34.45. IR ν_{max} (DCM): 2978, 2931, 2136, 1717, 1645, 1513, 1354, 1259, 1143 cm^{-1}. HR-MS (EI) calcd for $C_{19}H_{25}BNO_5Si$ $[M-H]^+$: 386.1590, found 386.1591.

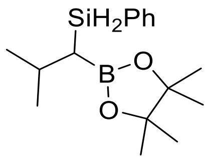

(2-Methyl-1-(4,4,5,5-tetramethyl-1,3,2-dioxaborolan-2-yl)propyl)(phenyl)silane (27). Following the general procedure I, the title compound (52.8 mg) was obtained in 91% yield. Colorless oil (eluent: hexane/EA = 100:1, R_f = 0.35). ^1H NMR (400 MHz, $CDCl_3$) δ 7.65 – 7.62 (m, 2H), 7.37 – 7.31 (m, 3H), 4.37 (qd, *J* = 6.1, 3.6 Hz, 2H), 2.05 – 1.97 (m, 1H), 1.16 (d, *J* = 18.7 Hz, 12H), 1.03 (dd, *J* = 13.4, 6.7 Hz, 6H), 0.78 – 0.75 (m, 1H). ^{13}C NMR (126 MHz, $CDCl_3$) δ 135.63, 132.75, 129.44, 127.80, 82.96, 27.37, 25.16, 24.99, 24.52. The carbon signal attached to B was not observed. ^{11}B NMR (128 MHz, $CDCl_3$) δ 33.89. IR ν_{max} (DCM): 2977, 2956, 2867, 2132, 1371, 1346, 1269, 1144 cm^{-1}. HR-MS (EI) calcd for $C_{16}H_{26}BO_2Si$ $[M-H]^+$: 289.1790, found 289.1797.

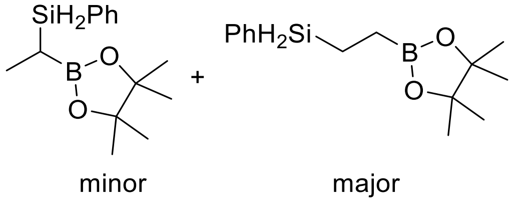

Phenyl(1-(4,4,5,5-tetramethyl-1,3,2-dioxaborolan-2-yl)ethyl)silane (**28**) and Phenyl(2-(4,4,5,5-tetramethyl-1,3,2-dioxaborolan-2-yl)ethyl)silane (**28'**). Following the general procedure I, the title compound (39.3 mg) was obtained in 75% yield. Colorless oil

(eluent: hexane/EA = 100:1, R_f = 0.35). ^{1}H NMR (400 MHz, $CDCl_3$) δ 7.63 – 7.59 (m, 2H), 7.59 – 7.55 (m, 2H), 7.41 – 7.31 (m, 3H), 4.40 – 4.28 (m, 2H) (**28**), 4.27 (t, *J* = 3.6 Hz, 2H) (**28'**), 1.23 (s, 12H) (**28'**), 1.19 (d, *J* = 3.7 Hz, 12H) (**28**), 1.15 (d, *J* = 7.3 Hz, 3H) (**28**), 1.03 – 0.97 (m, 2H) (**28**), 0.92 – 0.88 (m, 2H) (**28'**), 0.79 (dt, *J* = 7.3, 3.6 Hz, 1H) (**28**). ^{13}C NMR (126 MHz, $CDCl_3$) δ 135.54, 135.32, 132.31, 129.56, 129.45, 127.90, 127.84, 83.08, 24.87, 24.82, 24.66, 10.52, 3.28. ^{11}B NMR (128 MHz, $CDCl_3$) δ 34.51. The carbon signal attached to B was not observed. IR ν_{max} (DCM): 2978, 2931, 2875, 2124, 1644, 1342, 1220, 1145 cm^{-1}. HR-MS (EI) calcd for $C_{14}H_{22}BO_2Si$ [M-H]$^+$: 261.1477, found 261.1481.

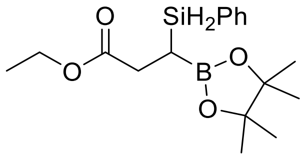

Ethyl 3-(phenylsilyl)-3-(4,4,5,5-tetramethyl-1,3,2-dioxaborolan-2-yl)propanoate (30). Following the general procedure I, the title compound (36.8 mg) was obtained in 55% yield. Colorless oil (eluent: hexane/EA = 50:1, R_f = 0.2). ^{1}H NMR (500 MHz, $CDCl_3$) δ 7.61 – 7.59 (m, 2H), 7.42 – 7.33 (m, 3H), 4.42 – 4.32 (m, 2H), 4.14 – 4.01 (m, 2H), 2.65 – 2.56 (m, 1H), 2.45 – 2.38 (m, 1H), 1.27 – 1.14 (m, 15H), 0.90 – 0.82 (m, 1H). ^{13}C NMR (126 MHz, $CDCl_3$) δ 174.19, 135.56, 131.37, 129.82, 127.99, 83.34, 60.52, 31.62, 24.86, 24.51, 14.26. The carbon signal attached to B was not observed. ^{11}B NMR (128 MHz, $CDCl_3$) δ 34.18. IR ν_{max} (DCM): 2979, 2930, 2134, 1734, 1638, 1356, 1260, 1142 cm^{-1}. HR-MS (EI) calcd for $C_{17}H_{26}BO_4Si$ [M-H]$^+$: 333.1688, found 333.1693.

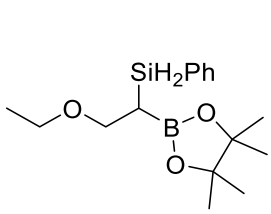

(2-Ethoxy-1-(4,4,5,5-tetramethyl-1,3,2-dioxaborolan-2-yl)ethyl)(phenyl)silane (31). Following the general procedure I, the title compound (43.3 mg) was obtained in 71% yield. Colorless oil (eluent: hexane/EA = 50:1, R_f = 0.35). ^{1}H NMR (400 MHz,

$CDCl_3$) δ 7.64 – 7.59 (m, 2H), 7.42 – 7.29 (m, 3H), 4.39 – 4.34 (m, 2H), 3.73 (t, *J* = 9.2 Hz, 1H), 3.63 – 3.54 (m, 1H), 3.41 (qd, *J* = 7.0, 2.4 Hz, 2H), 1.41 – 1.35 (m, 1H), 1.18 (d, *J* = 12.5 Hz, 12H), 1.14 (t, *J* = 7.0 Hz, 3H). ^{13}C NMR (126 MHz, $CDCl_3$) δ 135.64, 131.90, 129.57, 127.81, 83.19, 68.87, 65.69, 24.90, 24.47, 15.11. The carbon signal attached to B was not observed. ^{11}B NMR (128 MHz, $CDCl_3$) δ 33.75. IR ν_{max} (DCM): 2977, 2931, 2866, 2133, 1591, 1354, 1263, 1146 cm^{-1}. HR-MS (EI) calcd for $C_{16}H_{26}BO_3Si$ $[M-H]^+$: 305.1739, found 305.1735.

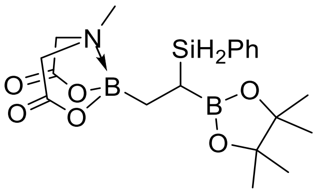

4-Methyl-8-(2-(phenylsilyl)-2-(4,4,5,5-tetramethyl-1,3,2-dioxaborolan-2-yl)ethyl)dihydro-4λ^4,8λ^4-[1,3,2]oxazaborolo[2,3-b][1,3,2]oxazaborole-2,6(3H,5H)-dione (32). Following the general procedure I, the title compound (46.7 mg) was obtained in 56% yield. Colorless oil (eluent: hexane/EA = 10:1, R_f = 0.35). ^1H NMR (400 MHz, $CDCl_3$) δ 7.68 – 7.66 (m, 2H), 7.39 – 7.32 (m, 3H), 4.42 – 4.31 (m, 2H), 3.75 (d, *J* = 1.2 Hz, 2H), 3.69 (d, *J* = 16.5 Hz, 1H), 3.52 (d, *J* = 16.5 Hz, 1H), 2.87 (s, 3H), 1.20 (d, *J* = 3.2 Hz, 12H), 0.99 – 0.93 (m, 2H), 0.71 – 0.66 (m, 1H). ^{13}C NMR (126 MHz, $CDCl_3$) δ 166.59, 135.90, 133.06, 129.55, 127.93, 83.47, 62.66, 46.01, 29.72, 25.28, 24.56. The carbon signal attached to B was not observed. ^{11}B NMR (128 MHz, $CDCl_3$) δ 34.49, 13.81. IR ν_{max} (DCM): 2978, 2935, 2114, 1726, 1652, 1312, 1263, 1146 cm^{-1}. HR-MS (APCI) calcd for $C_{19}H_{28}B_2NO_6Si$ $[M-H]^+$: 416.1877, found 416.1879.

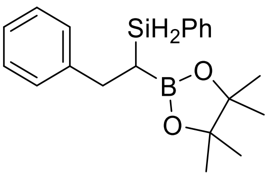

Phenyl(2-phenyl-1-(4,4,5,5-tetramethyl-1,3,2-dioxaborolan-2-yl)ethyl)silane (33). Following the general procedure II, the title compound (49.4 mg) was obtained in 73% yield. Colorless oil (eluent: hexane/EA = 100:1, R_f = 0.3). ^{1}H NMR (400 MHz, $CDCl_3$) δ 7.63 – 7.61 (m, 2H), 7.42 – 7.33 (m, 3H), 7.24 – 7.17 (m, 4H), 7.15 – 7.10 (m, 1H), 4.44 – 4.35 (m, 2H), 2.97 – 2.79 (m, 2H), 1.26 – 1.20 (m, 1H), 1.07 (d, *J* = 17.1 Hz, 12H). ^{13}C NMR (126 MHz, $CDCl_3$) δ 143.91, 135.57, 131.86, 129.70, 128.24, 128.10, 127.93, 125.67, 83.20, 32.63, 24.83, 24.47. The carbon signal attached to B was not observed. ^{11}B NMR (128 MHz, $CDCl_3$) δ 34.12. IR ν_{max} (DCM): 2977, 2929, 2857, 2129, 1639, 1429, 1353, 1242, 1143 cm^{-1}. HR-MS (EI) calcd for $C_{20}H_{27}BO_2Si$ $[M]^+$: 338.1868, found 338.1879.

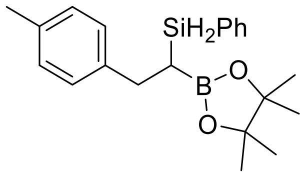

Phenyl(1-(4,4,5,5-tetramethyl-1,3,2-dioxaborolan-2-yl)-2-(p-tolyl)ethyl)silane (34). Following the general procedure II, the title compound (57.1 mg) was obtained in 81% yield. Colorless oil (eluent: hexane/EA = 100:1, R_f = 0.3). ^{1}H NMR (400 MHz, $CDCl_3$) δ 7.64 – 7.61 (m, 2H), 7.42 – 7.33 (m, 3H), 7.10 – 7.03 (m, 4H), 4.42 – 4.38 (m, 2H), 2.97 – 2.77 (m, 2H), 2.30 (s, 3H), 1.24 – 1.18 (m, 1H), 1.10 (d, *J* = 15.8 Hz, 12H). ^{13}C NMR (126 MHz, $CDCl_3$) δ 140.83, 135.59, 135.02, 131.96, 129.67, 128.78, 128.10, 127.92, 83.18, 32.20, 24.88, 24.50, 21.01. The carbon signal attached to B was not observed. ^{11}B NMR (128 MHz, $CDCl_3$) δ 33.83. IR ν_{max} (DCM): 2978, 2926, 2862, 2131, 1514, 1351, 1240, 1143 cm^{-1}. HR-MS (EI) calcd for $C_{21}H_{29}BO_2Si$ $[M]^+$: 352.2024, found 352.2015.

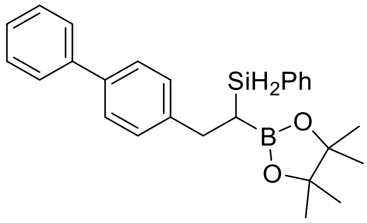

(2-([1,1'-Biphenyl]-4-yl)-1-(4,4,5,5-tetramethyl-1,3,2-dioxaborolan-2-yl)ethyl)(phenyl)silane (35). Following the general procedure II, the title compound (58.0 mg) was obtained in 70% yield. Colorless oil (eluent: hexane/EA = 50:1, R_f = 0.35). ^{1}H NMR (400 MHz, $CDCl_3$) δ 7.64 – 7.62 (m, 2H), 7.58 – 7.55 (m, 2H), 7.48 – 7.25 (m, 10H), 4.44 – 4.40 (m, 2H), 3.02 – 2.84 (m, 2H), 1.28 – 1.23 (m, 1H), 1.09 (d, *J* = 15.3 Hz, 12H). ^{13}C NMR (126 MHz, $CDCl_3$) δ 143.11, 141.22, 138.58, 135.58, 131.83, 129.71, 128.69, 128.67, 127.95, 126.97, 126.94, 126.83, 83.25, 32.30, 24.86, 24.49. The carbon signal attached to B was not observed. ^{11}B NMR (160 MHz, $CDCl_3$) δ 34.24. IR ν_{max} (DCM): 2977, 2926, 2855, 2133, 1486, 1351, 1320, 1142 cm^{-1}.HR-MS (EI) calcd for $C_{26}H_{31}BO_2Si$ $[M]^+$: 414.2181, found 414.2184.

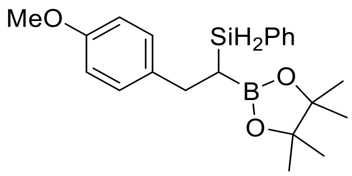

(3-(4-Methoxyphenyl)-1-(4,4,5,5-tetramethyl-1,3,2-dioxaborolan-2-yl)ethyl)(phenyl)silane (36). Following the general procedure II, the title compound (58.2 mg) was obtained in 79% yield. Colorless oil (eluent: hexane/EA = 50:1, R_f = 0.2). ^{1}H NMR (400 MHz, $CDCl_3$) δ 7.63 – 7.60 (m, 2H), 7.41 – 7.33 (m, 3H), 7.13 – 7.08 (m, 2H), 6.79 – 6.75 (m, 2H), 4.41 – 4.36 (m, 2H), 3.77 (s, 3H), 2.93 – 2.75 (m, 2H), 1.22 – 1.16 (m, 1H), 1.09 (d, *J* = 16.4 Hz, 12H). ^{13}C NMR (126 MHz, $CDCl_3$) δ 157.67, 136.12, 135.57, 131.95, 129.66, 129.15, 127.91, 113.49, 83.17, 55.27, 31.77, 24.88, 24.49. The carbon signal attached to B was not observed. ^{11}B NMR (128 MHz, $CDCl_3$) δ 35.91. IR ν_{max} (DCM): 2978, 2931, 2131, 1611, 1511, 1351, 1246, 1142 cm^{-1}. HR-MS (EI) calcd for $C_{21}H_{29}BO_3Si$ $[M]^+$: 368.1974, found 368.1984.

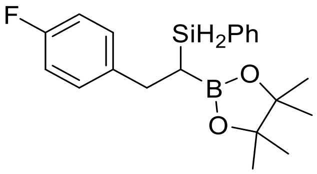

(2-(4-Fluorophenyl)-1-(4,4,5,5-tetramethyl-1,3,2-dioxaborolan-2-yl)ethyl)(phenyl)silane (37). Following the general procedure II, the title compound (53.4 mg) was obtained in 75% yield. Colorless oil (eluent: hexane/EA = 50:1, R_f = 0.3). ^{1}H NMR (400 MHz, $CDCl_3$) δ 7.63 – 7.60 (m, 2H), 7.43 – 7.33 (m, 3H), 7.17 – 7.11 (m, 2H), 6.93 – 6.87 (m, 2H), 4.41 – 4.37 (m, 2H), 2.94 – 2.76 (m, 2H), 1.23 – 1.16 (m, 1H), 1.08 (d, *J* = 18.5 Hz, 12H). ^{13}C NMR (126 MHz, $CDCl_3$) δ 161.20 (d, *J* = 243.0 Hz),139.58, 135.55, 131.70, 129.76, 129.60 (d, *J* = 7.8 Hz), 127.97, 114.75 (d, *J* = 20.9 Hz), 83.25, 31.88, 24.86, 24.47. The carbon signal attached to B was not observed. ^{19}F NMR (377 MHz, $CDCl_3$) δ -118.05. ^{11}B NMR (128 MHz, $CDCl_3$) δ 34.64. IR ν_{max} (DCM): 2979, 2930, 2133, 1601, 1509, 1429, 1351, 1220, 1146 cm^{-1}. HR-MS (EI) calcd for $C_{20}H_{26}BFO_2Si$ $[M]^+$: 356.1774, found 356.1780.

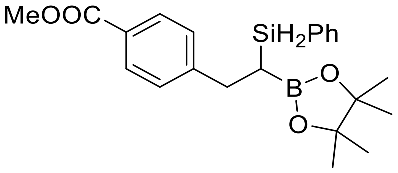

Methyl 4-(2-(phenylsilyl)-2-(4,4,5,5-tetramethyl-1,3,2-dioxaborolan-2-yl)ethyl)benzoate (38). Following the general procedure II, the title compound (44.4 mg) was obtained in 56% yield. Colorless oil (eluent: hexane/EA = 50:1, R_f = 0.2). ^{1}H NMR (400 MHz, $CDCl_3$) δ 7.92 – 7.89 (m, 2H), 7.63 – 7.61 (m, 2H), 7.42 – 7.34 (m, 3H), 7.28 – 7.24 (m, 2H), 4.43 – 4.38 (m, 2H), 3.89 (s, 3H), 3.01 – 2.83 (m, 2H), 1.25 – 1.19 (m, 1H), 1.07 (d, *J* = 19.9 Hz, 12H). ^{13}C NMR (126 MHz, $CDCl_3$) δ 167.23, 149.53, 135.56, 131.53, 129.83, 129.52, 128.28, 128.00, 127.66, 83.33, 51.93, 32.72, 24.86, 24.46. The carbon signal attached to B was not observed. ^{11}B NMR (128 MHz, $CDCl_3$) δ 35.08. IR ν_{max} (DCM): 2979, 2951, 2133, 1720, 1610, 1434, 1372, 1280, 1143 cm^{-1}. HR-MS (EI) calcd for $C_{22}H_{29}BO_4Si$ $[M]^+$: 396.1923, found 396.1927.

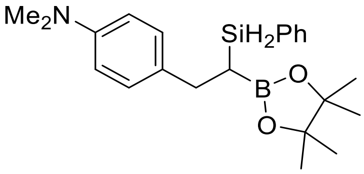

N,N-Dimethyl-4-(2-(phenylsilyl)-2-(4,4,5,5-tetramethyl-1,3,2-dioxaborolan-2-yl)ethyl)aniline (39). Following the general procedure II, the title compound (45.7 mg) was obtained in 60% yield. Colorless oil (eluent: hexane/EA = 20:1, R_f = 0.3). ^{1}H NMR (400 MHz, $CDCl_3$) δ 7.64 – 7.61 (m, 2H), 7.42 – 7.32 (m, 3H), 7.10 – 7.06 (m, 2H), 6.68 – 6.64 (m, 2H), 4.41 – 4.36 (m, 2H), 2.93 – 2.72 (m, 8H), 1.22 – 1.16 (m, 1H), 1.11 (d, J = 14.2 Hz, 12H). ^{13}C NMR (126 MHz, $CDCl_3$) δ 149.07, 135.60, 132.44, 132.16, 129.59, 128.81, 127.87, 113.00, 83.12, 41.07, 31.63, 24.91, 24.53. The carbon signal attached to B was not observed. ^{11}B NMR (128 MHz, $CDCl_3$) δ 35.03. IR ν_{max} (DCM): 2978, 2930, 2129, 1614, 1520, 1350, 1241, 1142 cm^{-1}. HR-MS (EI) calcd for $C_{22}H_{32}BNO_2Si$ $[M]^+$: 381.2290, found 381.2300.

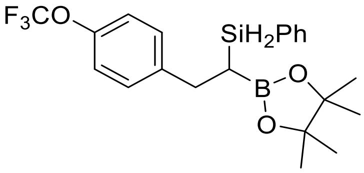

Phenyl(1-(4,4,5,5-tetramethyl-1,3,2-dioxaborolan-2-yl)-2-(4-(trifluoromethoxy)phenyl)ethyl)silane (40). Following the general procedure II, the title compound (63.3 mg) was obtained in 75% yield. Colorless oil (eluent: hexane/EA = 50:1, R_f = 0.25). ^{1}H NMR (400 MHz, $CDCl_3$) δ 7.63 – 7.59 (m, 2H), 7.43 – 7.34 (m, 3H), 7.23 – 7.19 (m, 2H), 7.10 – 7.05 (m, 2H), 4.43 – 4.38 (m, 2H), 2.96 – 2.80 (m, 2H), 1.24 – 1.18 (m, 1H), 1.07 (d, J = 18.5 Hz, 12H). ^{13}C NMR (126 MHz, $CDCl_3$) δ 147.30, 142.75, 135.53, 131.57, 129.81, 129.53, 127.99, 120.70, 120.53 (q, J = 257.04 Hz), 83.30, 32.03, 24.78, 24.43. The carbon signal attached to B was not observed. ^{19}F NMR (377 MHz, $CDCl_3$) δ -58.00. ^{11}B NMR (128 MHz, $CDCl_3$) δ 34.79. IR ν_{max} (DCM): 2980, 2932, 2132, 1508, 1352, 1263, 1165, 1142 cm^{-1}. HR-MS (EI) calcd for $C_{21}H_{26}BF_3O_3Si$ $[M]^+$: 422.1691, found 422.1692.

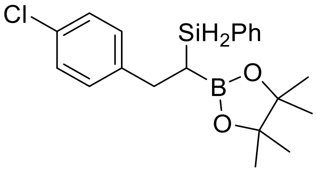

(2-(4-Chlorophenyl)-1-(4,4,5,5-tetramethyl-1,3,2-dioxaborolan-2-yl)ethyl)(phenyl)silane (41). Following the general procedure II, the title compound (52.1 mg) was obtained in 70% yield. Colorless oil (eluent: hexane/EA = 50:1, R_f = 0.3). ^{1}H NMR (400 MHz, $CDCl_3$) δ 7.63 – 7.59 (m, 2H), 7.43 – 7.33 (m, 3H), 7.20 – 7.16 (m, 2H), 7.14 – 7.10 (m, 2H), 4.42 – 4.36 (m, 2H), 2.93 – 2.75 (m, 2H), 1.21 – 1.15 (m, 1H), 1.09 (d, *J* = 18.9 Hz, 12H). ^{13}C NMR (126 MHz, $CDCl_3$) δ 142.41, 135.55, 131.61, 131.34, 129.79, 129.63, 128.16, 127.98, 83.30, 32.04, 24.89, 24.47. The carbon signal attached to B was not observed. ^{11}B NMR (128 MHz, $CDCl_3$) δ 34.59. IR ν_{max} (DCM): 2978, 2930, 2863, 2134, 1591, 1490, 1429, 1351, 1240, 1146 cm^{-1}. HR-MS (EI) calcd for $C_{20}H_{26}BClO_2Si$ $[M]^+$: 372.1478, found 372.1472.

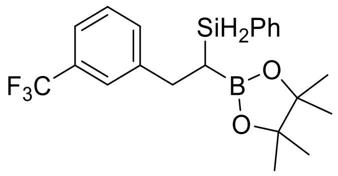

Phenyl(1-(4,4,5,5-tetramethyl-1,3,2-dioxaborolan-2-yl)-2-(3-(trifluoromethyl)phenyl)ethyl)silane (42). Following the general procedure II, the title compound (38.0 mg) was obtained in 47% yield. Colorless oil (eluent: hexane/EA = 50:1, R_f = 0.35). ^{1}H NMR (400 MHz, $CDCl_3$) δ 7.64 – 7.61 (m, 2H), 7.51 – 7.49 (m, 1H), 7.42 – 7.31 (m, 6H), 4.44 – 4.40 (m, 2H), 3.01 – 2.81 (m, 2H), 1.27 – 1.16 (m, 1H), 1.08 (d, *J* = 15.4 Hz, 12H). ^{13}C NMR (126 MHz, $CDCl_3$) δ 144.92, 135.55, 131.74, 131.45, 130.33 (q, *J* = 31.7 Hz), 129.86, 128.51, 128.02, 126.38 (q, *J* = 246.3 Hz), 125.09 (q, *J* = 3.7 Hz), 122.57 (q, *J* = 3.8 Hz), 83.38, 32.54, 24.75, 24.48. The carbon signal attached to B was not observed. ^{19}F NMR (377 MHz, $CDCl_3$) δ -62.55. ^{11}B NMR (128 MHz, $CDCl_3$) δ 34.41. IR ν_{max} (DCM): 2978, 2930, 2863, 2134, 1591, 1490, 1351, 1240, 1142 cm^{-1}. HR-MS (EI) calcd for $C_{21}H_{24}BF_3O_2Si$ $[M]^+$: 404.1585, found 404.1599.

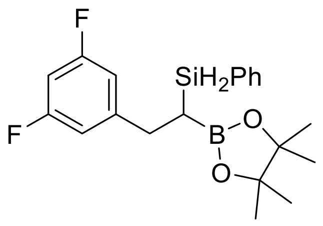

(2-(3,5-Difluorophenyl)-1-(4,4,5,5-tetramethyl-1,3,2-dioxaborolan-2-yl)ethyl)(phenyl)silane (43). Following the general procedure II, the title compound (31.4 mg) was obtained in 42% yield. Colorless oil (eluent: hexane/EA = 50:1, R_f = 0.35). ^{1}H NMR (400 MHz, $CDCl_3$) δ 7.63 – 7.59 (m, 2H), 7.43 – 7.34 (m, 3H), 6.75 – 6.68 (m, 2H), 6.60 – 6.54 (m, 1H), 4.42 – 4.37 (m, 2H), 2.93 – 2.74 (m, 2H), 1.19 – 1.14 (m, 1H), 1.10 (d, *J* = 17.6 Hz, 12H). ^{13}C NMR (126 MHz, $CDCl_3$) δ 162.83 (dd, *J* = 247.5, 13.0 Hz), 148.01, 135.52, 131.31, 129.91, 128.04, 111.01 (dd, *J* = 18.9, 5.0 Hz), 101.07 (t, *J* = 25.4 Hz), 83.43, 32.54, 24.86, 24.46. ^{19}F NMR (377 MHz, $CDCl_3$) δ -111.07. The carbon signal attached to B was not observed. ^{11}B NMR (128 MHz, $CDCl_3$) δ 34.65. IR ν_{max} (DCM): 2979, 2932, 2133, 1626, 1594, 1460, 1352, 1250, 1140 cm^{-1}. HR-MS (EI) calcd for $C_{20}H_{25}F_2BO_2Si$ $[M]^+$: 374.1679, found 374.1666.

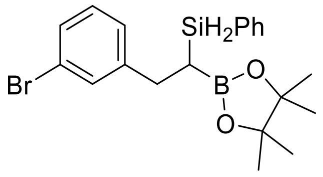

(2-(3-Bromophenyl)-1-(4,4,5,5-tetramethyl-1,3,2-dioxaborolan-2-yl)ethyl)(phenyl)silane (44). Following the general procedure II, the title compound (33.9 mg) was obtained in 41% yield. Colorless oil (eluent: hexane/EA = 50:1, R_f = 0.3). ^{1}H NMR (400 MHz, $CDCl_3$) δ 7.63 – 7.60 (m, 2H), 7.43 – 7.34 (m, 4H), 7.27 – 7.25 (m, 1H), 7.12 – 7.06 (m, 2H), 4.42 – 4.37 (m, 2H), 2.92 – 2.74 (m, 2H), 1.20 – 1.14 (m, 1H), 1.10 (d, *J* = 14.6 Hz, 12H). ^{13}C NMR (126 MHz, $CDCl_3$) δ 146.40, 135.55, 131.52, 131.40, 129.83, 129.70, 128.75, 128.00, 126.92, 122.17, 83.36, 32.39, 24.88, 24.49. The carbon signal attached to B was not observed. ^{11}B NMR (128 MHz, $CDCl_3$) δ 34.49. IR ν_{max} (DCM): 2978, 2928, 2131, 1630, 1593, 1351, 1237, 1142 cm^{-1}. HR-MS (EI) calcd for $C_{20}H_{26}BBrO_2Si$ $[M]^+$: 416.0973, found 416.0970.

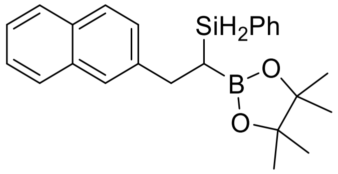

(2-(Naphthalen-2-yl)-1-(4,4,5,5-tetramethyl-1,3,2-dioxaborolan-2-yl)ethyl)(phenyl)silane (45). Following the general procedure II, the title compound (41.1 mg) was obtained in 53% yield. Colorless oil (eluent: hexane/EA = 50:1, R_f = 0.25). ^{1}H NMR (400 MHz, $CDCl_3$) δ 7.78 – 7.76 (m, 1H), 7.74 – 7.70 (m, 2H), 7.66 – 7.63 (m, 3H), 7.44 – 7.33 (m, 6H), 4.46 – 4.42 (m, 2H), 3.16 – 2.96 (m, 2H), 1.37 – 1.28 (m, 1H), 1.06 (d, *J* = 17.1 Hz, 12H). ^{13}C NMR (126 MHz, $CDCl_3$) δ 141.48, 135.60, 133.53, 131.98, 131.82, 129.74, 127.96, 127.69, 127.56, 127.46, 127.34, 126.04, 125.73, 124.97, 83.25, 32.82, 24.89, 24.46. The carbon signal attached to B was not observed. ^{11}B NMR (128 MHz, $CDCl_3$) δ 34.64. IR ν_{max} (DCM): 3051, 2977, 2928, 2132, 1634, 1507, 1348, 1257, 1142 cm^{-1}. HR-MS (EI) calcd for $C_{24}H_{29}BO_2Si$ $[M]^+$: 388.2024, found 388.2029.

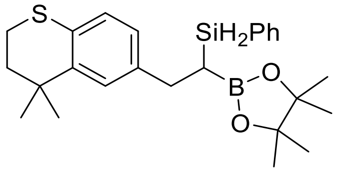

(4-(4,4-Dimethylthiochroman-6-yl)-1-(4,4,5,5-tetramethyl-1,3,2-dioxaborolan-2-yl)ethyl)(phenyl)silane (46). Following the general procedure II, the title compound (53.5 mg) was obtained in 46% yield. Colorless oil (eluent: hexane/EA = 20:1, R_f = 0.3). ^{1}H NMR (400 MHz, $CDCl_3$) δ 7.62 – 7.58 (m, 2H), 7.41 – 7.32 (m, 3H), 7.16 (d, *J* = 1.9 Hz, 1H), 6.96 – 6.86 (m, 2H), 4.41 – 4.36 (m, 2H), 3.02 – 2.97 (m, 2H), 2.91 – 2.73 (m, 2H), 1.95 – 1.89 (m, 2H), 1.29 (d, *J* = 4.6 Hz, 6H), 1.22 – 1.17 (m, 1H), 1.09 (d, *J* = 17.6 Hz, 12H). ^{13}C NMR (101 MHz, $CDCl_3$) δ 141.59, 139.62, 135.58, 131.92, 129.66, 128.26, 127.91, 126.36, 126.32, 126.13, 83.19, 37.99, 32.98, 32.30, 30.29, 30.22, 24.87, 24.56, 23.08. The carbon signal attached to B was not observed. ^{11}B NMR

(128 MHz, $CDCl_3$) δ 34.47. IR ν_{max} (DCM): 3048, 2975, 2934, 2133, 1652, 1477, 1350, 1254, 1143 cm^{-1}. HR-MS (EI) calcd for $C_{25}H_{35}BO_2SSi$ $[M]^+$: 438.2215, found 438.2210.

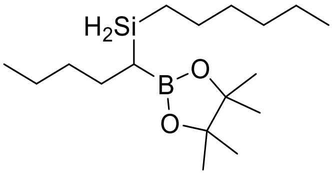

Hexyl(1-(4,4,5,5-tetramethyl-1,3,2-dioxaborolan-2-yl)pentyl)silane (47). Following the general procedure I, the title compound (31.7 mg) was obtained in 51% yield. Colorless oil (eluent: hexane/EA = 100:1, R_f = 0.4). ^{1}H NMR (400 MHz, $CDCl_3$) δ 3.72 – 3.65 (m, 2H), 1.67 – 1.58 (m, 1H), 1.46 – 1.24 (m, 13H), 1.23 (d, *J* = 5.0 Hz, 12H), 0.92 – 0.84 (m, 6H), 0.76 – 0.67 (m, 2H), 0.59 – 0.50 (m, 1H). ^{13}C NMR (126 MHz, $CDCl_3$) δ 82.85, 35.11, 32.56, 31.52, 26.88, 25.37, 25.02, 24.50, 22.57, 22.53, 14.12, 14.01, 9.32. The carbon signal attached to B was not observed. ^{11}B NMR (128 MHz, $CDCl_3$) δ 34.81. IR ν_{max} (DCM): 2977, 2958, 2923, 2856, 2120, 1541, 1466, 1352, 1309, 1146 cm^{-1}. HR-MS (EI) calcd for $C_{17}H_{36}BO_2Si$ $[M-H]^+$: 311.2572, found 311.2572.

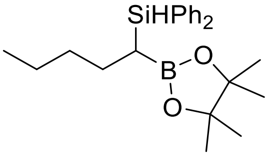

Diphenyl(1-(4,4,5,5-tetramethyl-1,3,2-dioxaborolan-2-yl)pentyl)silane (48). Following the general procedure I, the title compound (70.7 mg) was obtained in 93% yield. Colorless oil (eluent: hexane/EA = 50:1, R_f = 0.35). ^{1}H NMR (400 MHz, $CDCl_3$) δ 7.64 – 7.59 (m, 4H), 7.39 – 7.31 (m, 6H), 4.88 (d, *J* = 4.0 Hz, 1H), 1.75 – 1.66 (m, 1H), 1.54 – 1.47 (m, 2H), 1.39 – 1.21 (m, 4H), 1.06 (d, *J* = 29.5 Hz, 12H), 0.86 – 0.76 (m, 3H). ^{13}C NMR (126 MHz, $CDCl_3$) δ 135.58, 135.43, 134.51, 134.48, 129.44, 129.41, 127.79, 127.75, 82.94, 35.33, 26.33, 24.91, 24.48, 22.42, 14.00. The carbon signal attached to B was not observed. ^{11}B NMR (160 MHz, $CDCl_3$) δ 34.19. IR ν_{max} (DCM):

2975, 2951, 2858, 2113, 1645, 1362, 1350, 1260, 1143. HR-MS (EI) calcd for $C_{23}H_{32}BO_2Si$ [M-H]$^+$: 379.2259, found 379.2262.

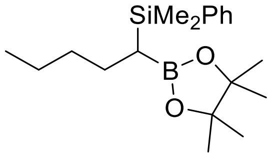

Dimethyl(phenyl)(1-(4,4,5,5-tetramethyl-1,3,2-dioxaborolan-2-yl)pentyl)silane (49). Following the general procedure I, the title compound (63.8 mg) was obtained in 96% yield. Colorless oil (eluent: hexane/EA = 50:1, R_f = 0.3). ^{1}H NMR (400 MHz, $CDCl_3$) δ 7.55 – 7.51 (m, 2H), 7.35 – 7.31 (m, 3H), 1.57 – 1.51 (m, 1H), 1.37 – 1.22 (m, 5H), 1.18 (d, J = 16.2 Hz, 12H), 0.84 – 0.77 (m, 3H), 0.63 (dd, J = 12.0, 2.5 Hz, 1H), 0.31 (d, J = 2.5 Hz, 6H). ^{13}C NMR (126 MHz, $CDCl_3$) δ 139.17, 133.85, 128.72, 127.56, 82.70, 35.51, 25.50, 25.09, 24.69, 22.43, 14.00, -2.34, -3.27. The carbon signal attached to B was not observed. ^{11}B NMR (128 MHz, $CDCl_3$) δ 35.23. IR ν_{max} (DCM): 2978, 2926, 1652, 1461, 1355, 1310, 1250, 1143 cm^{-1}. HR-MS (EI) calcd for $C_{19}H_{33}BO_2Si$ [M]$^+$: 332.2337, found 332.2340.

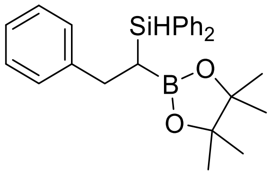

Diphenyl(2-phenyl-1-(4,4,5,5-tetramethyl-1,3,2-dioxaborolan-2-yl)ethyl)silane (50). Following the general procedure II, the title compound (70.4 mg) was obtained in 85% yield. Colorless oil (eluent: hexane/EA = 100:1, R_f = 0.2). ^{1}H NMR (500 MHz, $CDCl_3$) δ 7.68 – 7.64 (m, 4H), 7.41 – 7.34 (m, 6H), 7.23 – 7.18 (m, 4H), 7.13 – 7.10 (m, 1H), 4.97 (d, J = 4.0 Hz, 1H), 2.98 – 2.85 (m, 2H), 1.50 (dt, J = 12.0, 4.1 Hz, 1H), 0.93 (d, J = 37.0 Hz, 12H). ^{13}C NMR (126 MHz, $CDCl_3$) δ 144.18, 135.62, 135.44, 134.01, 133.93, 129.64, 129.60, 128.22, 128.07, 127.92, 127.86, 125.59, 83.09, 32.30, 24.72, 24.47. The carbon signal attached to B was not observed. ^{11}B NMR (128 MHz,

$CDCl_3$) δ 34.72. IR ν_{max} (DCM): 2977, 2929, 2856, 2120, 1653, 1429, 1352, 1241, 1143 cm^{-1}. HR-MS (EI) calcd for $C_{26}H_{31}BO_2Si$ $[M]^+$: 414.2181, found 414.2198.

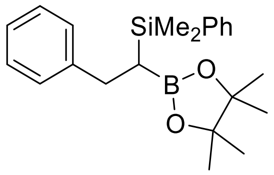

Dimethyl(phenyl)(2-phenyl-1-(4,4,5,5-tetramethyl-1,3,2-dioxaborolan-2-yl)ethyl)silane (51). Following the general procedure II, the title compound (69.6 mg) was obtained in 95% yield. Colorless oil (eluent: hexane/EA = 50:1, R_f = 0.3). ^1H NMR (400 MHz, $CDCl_3$) δ 7.62 – 7.57 (m, 2H), 7.38 – 7.35 (m, 3H), 7.22 – 7.07 (m, 5H), 2.83 – 2.66 (m, 2H), 1.10 – 1.06 (m, 7H), 1.03 (s, 6H), 0.38 (d, J = 8.9 Hz, 6H). ^{13}C NMR (126 MHz, $CDCl_3$) δ 144.78, 138.60, 133.89, 128.95, 128.16, 127.96, 127.71, 125.36, 82.86, 31.53, 24.90, 24.70, -2.37, -3.39. The carbon signal attached to B was not observed. ^{11}B NMR (128 MHz, $CDCl_3$) δ 34.87. IR ν_{max} (DCM): 2975, 2929, 2857, 1633, 1592, 1349, 1251, 1143 cm^{-1}. HR-MS (EI) calcd for $C_{21}H_{28}BO_2Si$ $[M-CH_3]^+$: 351.1946, found 351.1952.

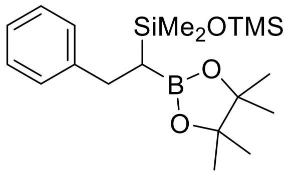

1,1,1,3,3-Pentamethyl-3-(2-phenyl-1-(4,4,5,5-tetramethyl-1,3,2-dioxaborolan-2-yl)ethyl)disiloxane (52). Following the general procedure II, the title compound (38.6 mg) was obtained in 51% yield. Colorless oil (eluent: hexane/EA = 20:1, R_f = 0.4). ^1H NMR (500 MHz, $CDCl_3$) δ 7.23 – 7.18 (m, 4H), 7.14 – 7.08 (m, 1H), 2.83 – 2.76 (m, 2H), 1.33 – 1.28 (m, 1H), 1.09 (d, J = 10.2 Hz, 12H), 0.15 (s, 6H), 0.10 (s, 9H). ^{13}C NMR (126 MHz, $CDCl_3$) δ 145.03, 128.24, 127.97, 125.29, 82.72, 30.75, 24.88, 24.65, 2.03, 0.70, 0.28. The carbon signal attached to B was not observed. ^{11}B NMR (128 MHz,

$CDCl_3$) δ 34.47. IR ν_{max} (DCM): 2978, 2958, 1652, 1454, 1378, 1353, 1253, 1145 cm^{-1}. HR-MS (EI) calcd for $C_{19}H_{35}BO_3Si_2$ $[M]^+$: 378.2212, found 378.2211.

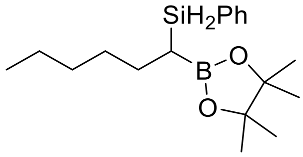

Phenyl(1-(4,4,5,5-tetramethyl-1,3,2-dioxaborolan-2-yl)hexyl)silane (53). Following the general procedure I, the title compound (55.4 mg) was obtained in 87% yield. Colorless oil (eluent: hexane/EA = 100:1, R_f = 0.35). ^1H NMR (400 MHz, $CDCl_3$) δ 7.62 – 7.59 (m, 2H), 7.40 – 7.31 (m, 3H), 4.37 – 4.31 (m, 2H), 1.72 – 1.62 (m, 1H), 1.52 – 1.44 (m, 1H), 1.39 – 1.24 (m, 6H), 1.18 (d, J = 13.4 Hz, 12H), 0.88 – 0.83 (m, 3H), 0.83 – 0.78 (m, 1H). ^{13}C NMR (101 MHz, $CDCl_3$) δ 135.53, 132.45, 129.51, 127.83, 83.01, 32.45, 31.62, 26.97, 24.97, 24.49, 22.51, 14.04. The carbon signal attached to B was not observed. ^{11}B NMR (128 MHz, $CDCl_3$) δ 34.00. IR ν_{max} (DCM): 2977, 2959, 2856, 2129, 1467, 1352, 1262, 1145 cm^{-1}. HR-MS (EI) calcd for $C_{18}H_{30}BO_2Si$ $[M-H]^+$: 317.2103, found 317.2105.

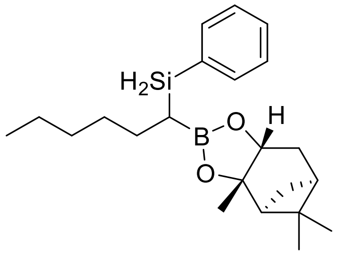

Phenyl(1-((3aS,4S,6S,7aR)-3a,5,5-trimethylhexahydro-4,6-methanobenzo[d][1,3,2]dioxaborol-2-yl)hexyl)silane (54). Following the general procedure I, the title compound (67.4 mg) was obtained in 91% yield (eluent: hexane/EA = 100:1, R_f = 0.3). Colorless oil. d.r. = 1:1. ^1H NMR (400 MHz, $CDCl_3$) δ 7.62 – 7.59 (m, 2H), 7.40 – 7.32 (m, 3H), 4.40 – 4.34 (m, 2H), 4.24 – 4.21 (m, 1H), 2.36 – 2.27 (m, 1H), 2.20 – 2.10 (m, 1H), 2.04 – 2.01 (m, 1H), 1.91 – 1.84 (m, 1H), 1.83 – 1.64 (m, 2H), 1.56 – 1.46 (m, 1H), 1.44 – 1.32 (m, 2H), 1.30 – 1.24 (m, 10H), 1.08 (dd, J = 10.9, 4.6 Hz, 1H), 0.88 – 0.82 (m, 7H). ^{13}C NMR (126 MHz, $CDCl_3$) δ

135.54, 135.51, 132.46, 132.44, 129.53, 127.86, 85.46, 85.44, 77.67, 77.64, 51.23, 39.59, 39.56, 38.15, 38.11, 35.68, 35.65, 32.45, 31.68, 31.64, 28.75, 28.60, 27.11, 27.04, 26.62, 26.46, 24.04, 22.53, 22.51, 14.06. The carbon signal attached to B was not observed. ^{11}B NMR (128 MHz, $CDCl_3$) δ 33.48. IR ν_{max} (DCM): 2984, 2923, 2858, 2128, 1652, 1429, 1376, 1278, 1120 cm^{-1}. HR-MS (EI) calcd for $C_{22}H_{35}BO_2Si$ $[M]^+$: 370.2494, found 370.2493.

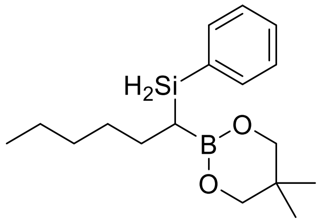

(1-(5,5-Dimethyl-1,3,2-dioxaborinan-2-yl)hexyl)(phenyl)silane (55). Following the general procedure I, the title compound (54.8 mg) was obtained in 90% yield (eluent: hexane/EA = 100:1, R_f = 0.3). Colorless oil. ^{1}H NMR (400 MHz, $CDCl_3$) δ 7.61 – 7.58 (m, 2H), 7.42 – 7.29 (m, 3H), 4.40 – 4.28 (m, 2H), 3.54 (s, 4H), 1.69 – 1.57 (m, 1H), 1.47 – 1.20 (m, 7H), 0.91 (s, 6H), 0.89 – 0.81 (m, 3H), 0.73 – 0.63 (m, 1H). ^{13}C NMR (126 MHz, $CDCl_3$) δ 135.45, 133.15, 129.32, 127.78, 72.09, 32.50, 31.79, 31.61, 26.93, 22.53, 21.87, 14.07. The carbon signal attached to B was not observed. ^{11}B NMR (128 MHz, $CDCl_3$) δ 31.19. IR ν_{max} (DCM): 2959, 2924, 2855, 2126, 1652, 1475, 1411, 1263, 1118 cm^{-1}. HR-MS (EI) calcd for $C_{17}H_{28}BO_2Si$ $[M-H]^+$: 303.1946, found 303.1948.

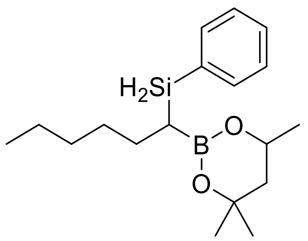

Phenyl(1-(4,4,6-trimethyl-1,3,2-dioxaborinan-2-yl)hexyl)silane (56). Following the general procedure I, the title compound (55.4 mg) was obtained in 87% yield. d.r. = 1:1. Colorless oil (eluent: hexane/EA = 100:1, R_f = 0.35). ^{1}H NMR (400 MHz, $CDCl_3$) δ 7.62 – 7.58 (m, 2H), 7.38 – 7.30 (m, 3H), 4.32 – 4.26 (m, 2H), 4.12 – 4.03 (m, 1H),

1.69 – 1.60 (m, 2H), 1.44 – 1.24 (m, 8H), 1.19 – 1.13 (m, 9H), 0.88 – 0.84 (m, 3H), 0.64 – 0.58 (m, 1H). ^{13}C NMR (126 MHz, $CDCl_3$) δ 135.59, 133.53, 129.18, 127.64, 70.57, 70.42, 64.68, 64.53, 46.00, 45.91, 32.30, 32.27, 31.79, 31.18, 31.15, 28.05, 27.75, 26.98, 23.18, 23.16, 22.54, 14.10. The carbon signal attached to B was not observed. ^{11}B NMR (128 MHz, $CDCl_3$) δ 30.29. IR ν_{max} (DCM): 2972, 2927, 2855, 2127, 1428, 1386, 1241, 1210, 1116 cm^{-1}. HR-MS (EI) calcd for $C_{18}H_{30}BO_2Si$ [M-H]$^+$: 317.2103, found 317.2104.

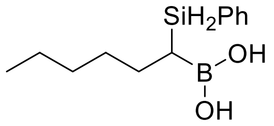

(1-(Phenylsilyl)hexyl)boronic acid (57). Following the general procedure I, the title compound (24.1 mg) was obtained in 51% yield. Colorless oil (eluent: hexane/EA = 5:1, R_f = 0.25). ^{1}H NMR (400 MHz, $CDCl_3$) δ 7.54 – 7.50 (m, 2H), 7.39 – 7.29 (m, 3H), 4.32 – 4.28 (m, 2H), 1.71 – 1.61 (m, 1H), 1.43 – 1.38 (m, 1H), 1.31 – 1.17 (m, 7H), 0.89 – 0.82 (m, 4H). ^{13}C NMR (126 MHz, $CDCl_3$) δ 135.27, 132.18, 129.63, 127.95, 32.65, 32.50, 31.84, 26.47, 22.51, 14.12. The carbon signal attached to B was not observed. ^{11}B NMR (128 MHz, $CDCl_3$) δ 33.79. IR ν_{max} (DCM): 2957, 2925, 2855, 2135, 1653, 1429, 1354, 1261, 1116 cm^{-1}. The mass result for **57** not found, it can further convert to **53** (α:β > 20:1) in 90% NMR yield.

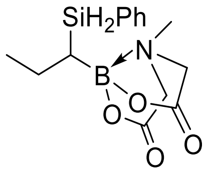

4-Methyl-8-(1-(phenylsilyl)propyl)dihydro-4λ^4,8λ^4-[1,3,2]oxazaborolo[2,3-b][1,3,2]oxazaborole-2,6(3H,5H)-dione (58). Following the general procedure I, the title compound (50.7 mg) was obtained in 83% yield. Colorless oil (eluent: hexane/EA = 10:1, R_f = 0.3). ^{1}H NMR (400 MHz, $CDCl_3$) δ 7.65 – 7.63 (m, 2H), 7.38 – 7.32 (m, 3H), 4.43 – 4.33 (m, 2H), 3.92 (t, *J* = 16.3 Hz, 2H), 3.65 (dd, *J* = 16.7, 7.5 Hz, 2H),

2.87 (s, 3H), 1.53 (p, J = 7.2 Hz, 2H), 0.99 (t, J = 7.3 Hz, 3H), 0.40 – 0.35 (m, 1H). ^{13}C NMR (126 MHz, $CDCl_3$) δ 167.71, 167.59, 135.73, 135.67, 132.98, 129.59, 128.03, 127.97, 62.77, 62.70, 46.00, 20.24, 15.96. The carbon signal attached to B was not observed. ^{11}B NMR (128 MHz, $CDCl_3$) δ 14.42. IR ν_{max} (DCM): 2958, 2930, 2869, 2130, 1747, 1455, 1260, 1119 cm^{-1}. HR-MS (APCI) calcd for $C_{13}H_{17}BNO_4Si$ [M-CH_3]$^+$: 290.1025, found 290.1035.

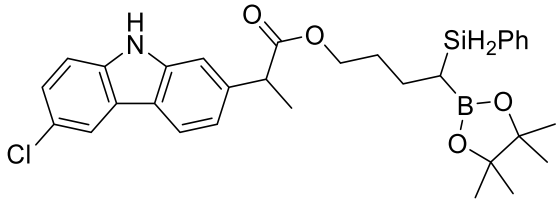

4-(Phenylsilyl)-4-(4,4,5,5-tetramethyl-1,3,2-dioxaborolan-2-yl)butyl 2-(6-chloro-9H-carbazol-2-yl)propanoate (59). Following the general procedure I, the title compound (68.5 mg) was obtained in 61% yield. d.r. = 1:1. Colorless oil (eluent: hexane/EA = 10:1, R_f = 0.35). ^{1}H NMR (400 MHz, $CDCl_3$) δ 8.26 (d, J = 57.8 Hz, 1H), 8.00 – 7.97 (m, 1H), 7.92 (dd, J = 8.1, 1.7 Hz, 1H), 7.54 – 7.51 (m, 2H), 7.41 – 7.26 (m, 6H), 7.17 – 7.14 (m, 1H), 4.31 – 4.19 (m, 2H), 4.14 – 3.97 (m, 2H), 3.86 (q, J = 7.1 Hz, 1H), 1.80 – 1.60 (m, 3H), 1.56 (dd, J = 7.1, 0.7 Hz, 3H), 1.52 – 1.40 (m, 1H), 1.16 (dd, J = 14.5, 8.2 Hz, 12H), 0.80 – 0.73 (m, 1H). ^{13}C NMR (126 MHz, $CDCl_3$) δ 174.69, 140.44, 140.37, 139.48, 139.30, 138.08, 135.49, 135.46, 131.91, 131.85, 129.71, 129.69, 127.91, 125.75, 124.88, 124.84, 124.39, 121.61, 121.56, 120.53, 119.99, 119.78, 119.62, 111.53, 111.51, 109.66, 109.59, 83.33, 83.24, 64.78, 64.69, 46.06, 45.93, 31.60, 31.39, 25.04, 24.98, 24.48, 24.45, 23.67, 23.50, 19.05, 18.68. The carbon signal attached to B was not observed. ^{11}B NMR (128 MHz, $CDCl_3$) δ 35.20. IR ν_{max} (DCM): 3363, 2976, 2932, 2133, 1716, 1611, 1471, 1353, 1142 cm^{-1}. HR-MS (APCI) calcd for $C_{31}H_{36}BClO_4Si$ [M-H]$^+$: 560.2190, found 560.2201.

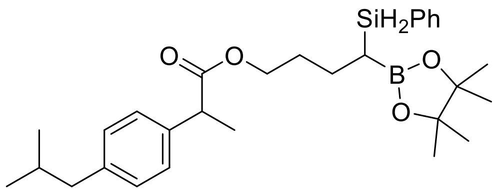

4-(Phenylsilyl)-4-(4,4,5,5-tetramethyl-1,3,2-dioxaborolan-2-yl)butyl 2-(4-isobutylphenyl)propanoate (60). Following the general procedure I with racemic starting material, the title compound (68.2 mg) was obtained in 69% yield. d.r. = 1.2:1. Colorless oil (eluent: hexane/EA = 20:1, R_f = 0.3). ^{1}H NMR (500 MHz, $CDCl_3$) δ 7.60 – 7.58 (m, 2H), 7.40 – 7.32 (m, 3H), 7.18 (d, *J* = 8.1 Hz, 2H), 7.07 (d, *J* = 8.2 Hz, 2H), 4.36 – 4.30 (m, 2H), 4.06 – 3.97 (m, 2H), 3.66 (qd, *J* = 7.2, 2.1 Hz, 1H), 2.44 (d, *J* = 7.2 Hz, 2H), 1.88 – 1.80 (m, 1H), 1.72 – 1.48 (m, 4H), 1.46 (d, *J* = 7.2 Hz, 3H), 1.17 (d, *J* = 15.9 Hz, 12H), 0.90 (d, *J* = 6.7 Hz, 6H), 0.80 – 0.75 (m, 1H). ^{13}C NMR (126 MHz, $CDCl_3$) δ 174.73, 140.40, 137.89, 137.86, 135.53, 132.02, 129.66, 129.27, 127.90, 127.19, 127.17, 83.16, 64.48, 64.45, 45.19, 45.16, 45.07, 31.37, 30.19, 24.98, 24.50, 23.34, 23.33, 22.43, 18.54, 18.49. The carbon signal attached to B was not observed. ^{11}B NMR (128 MHz, $CDCl_3$) δ 35.30. IR ν_{max} (DCM): 2976, 2931, 2868, 2132, 1734, 1465, 1353, 1316, 1144 cm^{-1}. HR-MS (EI) calcd for $C_{29}H_{43}BO_4Si$ $[M]^+$: 494.3018, found 494.3021.

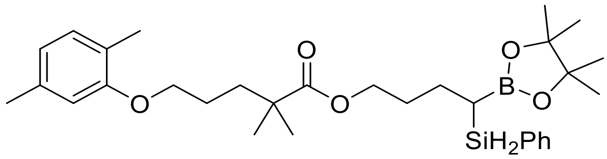

4-(Phenylsilyl)-4-(4,4,5,5-tetramethyl-1,3,2-dioxaborolan-2-yl)butyl 5-(2,5-dimethylphenoxy)-2,2-dimethylpentanoate (61). Following the general procedure I, the title compound (92.6 mg) was obtained in 86% yield. Colorless oil (eluent: hexane/EA = 20:1, R_f = 0.25). ^{1}H NMR (400 MHz, $CDCl_3$) δ 7.62 – 7.58 (m, 2H), 7.40 – 7.32 (m, 3H), 7.01 (d, *J* = 7.5 Hz, 1H), 6.66 (d, *J* = 7.5 Hz, 1H), 6.61 (s, 1H), 4.42 – 4.32 (m, 2H), 4.04 (t, *J* = 6.3 Hz, 2H), 3.89 (d, *J* = 5.5 Hz, 2H), 2.31 (s, 3H), 2.18 (s, 3H), 1.80 – 1.55 (m, 8H), 1.19 (d, *J* = 2.8 Hz, 12H), 1.17 (s, 6H), 0.85 – 0.79 (m, 1H). ^{13}C NMR (126 MHz, $CDCl_3$) δ 177.78, 157.00, 136.43, 135.52, 131.98, 130.29, 129.68,

127.92, 123.60, 120.66, 111.95, 83.18, 67.98, 64.12, 42.08, 37.11, 31.51, 25.20, 24.99, 24.51, 23.45, 21.44, 15.81. The carbon signal attached to B was not observed. ^{11}B NMR (128 MHz, $CDCl_3$) δ 34.96. IR ν_{max} (DCM): 2976, 2867, 2131, 1726, 1586, 1509, 1353, 1143 cm^{-1}. HR-MS (EI) calcd for $C_{31}H_{47}BO_5Si$ $[M]^+$: 538.3280, found 538.3270.

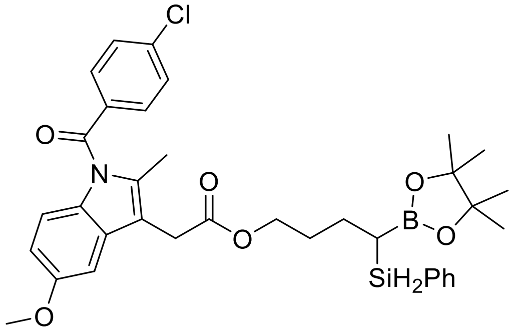

4-(Phenylsilyl)-4-(4,4,5,5-tetramethyl-1,3,2-dioxaborolan-2-yl)butyl 2-(1-(4-chlorobenzoyl)-5-methoxy-2-methyl-1H-indol-3-yl)acetate (62). Following the general procedure I, the title compound (67.1 mg) was obtained in 52% yield. Colorless oil (eluent: hexane/EA = 10:1, R_f = 0.4). ^{1}H NMR (500 MHz, $CDCl_3$) δ 7.66 – 7.63 (m, 2H), 7.60 – 7.54 (m, 2H), 7.49 – 7.43 (m, 2H), 7.41 – 7.29 (m, 3H), 6.95 (d, J = 2.6 Hz, 1H), 6.86 (d, J = 8.9 Hz, 1H), 6.66 (dd, J = 9.0, 2.6 Hz, 1H), 4.36 – 4.27 (m, 2H), 4.05 (t, J = 6.2 Hz, 2H), 3.82 (s, 3H), 3.62 (s, 2H), 2.36 (s, 3H), 1.79 – 1.61 (m, 3H), 1.55 (s, 1H), 1.16 (d, J = 16.1 Hz, 12H), 0.80 – 0.77 (m, 1H). ^{13}C NMR (126 MHz, $CDCl_3$) δ 170.87, 168.30, 156.06, 139.22, 135.92, 135.51, 133.98, 131.89, 131.19, 130.80, 130.71, 129.71, 129.12, 127.92, 114.96, 112.74, 111.72, 101.24, 83.20, 64.89, 55.70, 31.38, 30.36, 24.97, 24.50, 23.42, 13.37. The carbon signal attached to B was not observed. ^{11}B NMR (128 MHz, $CDCl_3$) δ 35.92. IR ν_{max} (DCM): 2976, 2929, 2132, 1728, 1486, 1353, 1310, 1217, 1142 cm^{-1}. HR-MS (APCI) calcd for $C_{35}H_{42}BClNO_6Si$ $[M+H]^+$: 646.2557, found 646.2564.

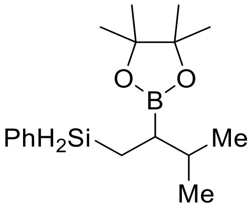

(3-Methyl-2-(4,4,5,5-tetramethyl-1,3,2-dioxaborolan-2-yl)butyl)(phenyl)silane (63). Following the general procedure I, the title compound (50.5 mg) was obtained in 83% yield. Colorless oil (eluent: hexane/EA = 100:1, R_f = 0.3). ^{1}H NMR (400 MHz, $CDCl_3$) δ 7.59 – 7.57 (m, 2H), 7.40 – 7.32 (m, 3H), 4.33 – 4.24 (m, 2H), 1.80 – 1.72 (m, 1H), 1.24 (d, *J* = 1.7 Hz, 12H), 1.10 – 1.05 (m, 2H), 1.00 – 0.95 (m, 1H), 0.93 (d, *J* = 6.7 Hz, 6H). ^{13}C NMR (101 MHz, $CDCl_3$) δ 135.28, 133.25, 129.38, 127.89, 83.11, 31.97, 25.03, 24.92, 21.72, 21.03, 8.53. The carbon signal attached to B was not observed. ^{11}B NMR (128 MHz, $CDCl_3$) δ 34.51. ^{29}Si NMR (99 MHz, $CDCl_3$) δ -30.18. IR ν_{max} (DCM): 2972, 2956, 2870, 2131, 1733, 1371, 1319, 1271, 1143 cm^{-1}. HR-MS (EI) calcd for $C_{17}H_{28}BO_2Si$ $[M-H]^+$: 303.1946, found 303.1948.

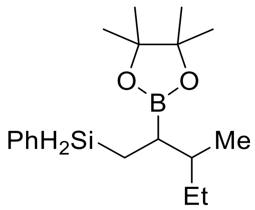

(3-Methyl-2-(4,4,5,5-tetramethyl-1,3,2-dioxaborolan-2-yl)pentyl)(phenyl)silane (64). Following the general procedure I, the title compound (51.6 mg) was obtained in 81% yield. d.r. = 1.3:1. Colorless oil (eluent: hexane/EA = 100:1, R_f = 0.3). ^{1}H NMR (400 MHz, $CDCl_3$) δ 7.59 – 7.57 (m, 2H), 7.40 – 7.31 (m, 3H), 4.32 – 4.23 (m, 2H), 1.49 – 1.38 (m, 2H), 1.28 – 1.21 (m, 13H), 1.18 – 1.10 (m, 2H), 0.95 – 0.81 (m, 7H). ^{13}C NMR (126 MHz, $CDCl_3$) δ 135.28, 133.27, 129.37, 127.88, 83.10, 39.10, 38.12, 28.69, 27.72, 25.03, 24.89, 24.81, 17.79, 17.51, 12.20, 11.96, 9.05, 6.70. The carbon signal attached to B was not observed. ^{11}B NMR (128 MHz, $CDCl_3$) δ 34.63. IR ν_{max} (DCM): 2975, 2953, 2877, 2127, 1740, 1372, 1320, 1275, 1144 cm^{-1}. HR-MS (EI) calcd for $C_{18}H_{30}BO_2Si$ $[M-H]^+$: 317.2103, found 317.2112.

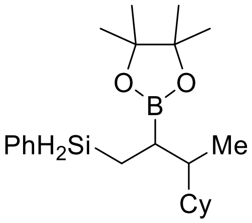

(3-Cyclohexyl-2-(4,4,5,5-tetramethyl-1,3,2-dioxaborolan-2-yl)butyl)(phenyl)silane (65). Following the general procedure I, the title compound (62.5 mg) was obtained in 84% yield. d.r. = 1.2:1. Colorless oil (eluent: hexane/EA = 100:1, R_f = 0.3). ^{1}H NMR (400 MHz, $CDCl_3$) δ 7.52 – 7.49 (m, 2H), 7.32 – 7.24 (m, 3H), 4.27 – 4.15 (m, 2H), 1.64 – 1.48 (m, 8H), 1.23 – 1.14 (m, 15H), 1.08 – 1.00 (m, 2H), 0.93 – 0.85 (m, 2H), 0.80 – 0.76 (m, 3H). ^{13}C NMR (126 MHz, $CDCl_3$) δ 135.27, 133.25, 129.38, 127.89, 83.10, 42.22, 41.28, 40.97, 40.66, 31.78, 29.18, 28.48, 26.91, 26.82, 26.78, 26.71, 25.09, 25.03, 24.94, 24.80, 15.01, 14.40, 9.57, 5.96. The carbon signal attached to B was not observed. ^{11}B NMR (128 MHz, $CDCl_3$) δ 34.87. IR ν_{max} (DCM): 2978, 2923, 2851, 2125, 1653, 1370, 1316, 1261, 1144 cm^{-1}. HR-MS (EI) calcd for $C_{22}H_{36}BO_2Si$ [M-H]$^+$: 371.2572, found 371.2584.

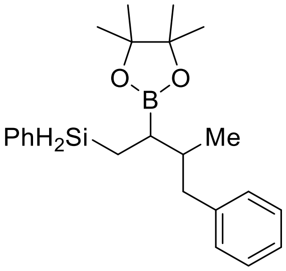

(3-Methyl-4-phenyl-2-(4,4,5,5-tetramethyl-1,3,2-dioxaborolan-2-yl)butyl)(phenyl)silane (66). Following the general procedure I, the title compound (40.3 mg) was obtained in 53% yield. d.r. = 1.5:1. Colorless oil (eluent: hexane/EA = 100:1, R_f = 0.25). ^{1}H NMR (400 MHz, $CDCl_3$) δ 7.57 – 7.54 (m, 2H), 7.38 – 7.30 (m, 3H), 7.26 – 7.22 (m, 2H), 7.18 – 7.12 (m, 3H), 4.32 – 4.22 (m, 2H), 2.66 – 2.44 (m, 2H), 1.74 – 1.59 (m, 2H), 1.52 – 1.39 (m, 1H), 1.22 (d, J = 3.5 Hz, 12H), 1.15 – 1.05 (m, 1H), 0.98 – 0.95 (m, 3H). ^{13}C NMR (126 MHz, $CDCl_3$) δ 143.13, 135.32, 133.17, 129.44, 128.43, 128.39, 128.26, 127.93, 125.53, 83.15, 38.16, 37.15, 36.91, 36.21,

34.15, 33.84, 25.09, 24.93, 18.25, 17.98, 9.00, 6.85. The carbon signal attached to B was not observed. ^{11}B NMR (128 MHz, $CDCl_3$) δ 34.54. IR ν_{max} (DCM): 2977, 2928, 2870, 2125, 1652, 1407, 1379, 1316, 1144 cm^{-1}. HR-MS (EI) calcd for $C_{23}H_{32}BO_2Si$ $[M-H]^+$: 379.2259, found 379.2266.

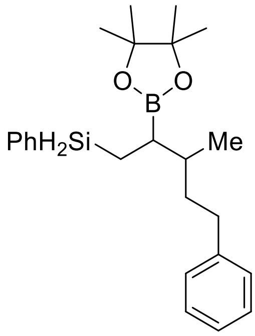

(3-Methyl-5-phenyl-2-(4,4,5,5-tetramethyl-1,3,2-dioxaborolan-2-yl)pentyl)(phenyl)silane (67). Following the general procedure I, the title compound (43.4 mg) was obtained in 55% yield. d.r. = 1.4:1. Colorless oil (eluent: hexane/EA = 100:1, R_f = 0.25). ^{1}H NMR (400 MHz, $CDCl_3$) δ 7.65 – 7.52 (m, 2H), 7.38 – 7.32 (m, 3H), 7.25 – 7.21 (m, 2H), 7.18 – 7.07 (m, 3H), 4.41 – 4.21 (m, 2H), 2.80 (ddd, *J* = 13.2, 5.2, 2.0 Hz, 1H), 2.69 – 2.50 (m, 1H), 2.39 – 2.26 (m, 1H), 2.00 – 1.78 (m, 2H), 1.30 – 1.22 (m, 14H), 1.09 – 1.00 (m, 2H), 0.86 (dd, *J* = 11.6, 6.8 Hz, 3H).^{13}C NMR (126 MHz, $CDCl_3$) δ 141.83, 135.77, 135.29, 133.23, 129.44, 129.41, 129.23, 129.20, 128.34, 128.23, 128.11, 128.08, 127.94, 127.91, 125.59, 125.56, 83.22, 42.52, 41.66, 39.58, 38.71, 35.52, 33.57, 25.16, 25.05, 24.93, 24.89, 17.79, 17.50, 13.60, 9.17, 7.20. The carbon signal attached to B was not observed. ^{11}B NMR (128 MHz, $CDCl_3$) δ 34.98. IR ν_{max} (DCM): 2978, 2928, 2127, 1645, 1454, 1378, 1316, 1260, 1117 cm^{-1}. HR-MS (EI) calcd for $C_{24}H_{34}BO_2Si$ $[M-H]^+$: 393.2416, found 393.2409.

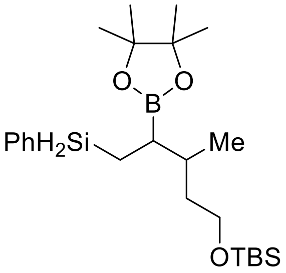

tert-Butyldimethyl((3-methyl-5-(phenylsilyl)-4-(4,4,5,5-tetramethyl-1,3,2-dioxaborolan-2-yl)pentyl)oxy)silane (68). Following the general procedure I, the title compound (68.1 mg) was obtained in 76% yield. d.r. = 1.2:1. Colorless oil (eluent: hexane/EA = 50:1, R_f = 0.2). ^{1}H NMR (400 MHz, $CDCl_3$) δ 7.59 – 7.56 (m, 2H), 7.39 – 7.31 (m, 3H), 4.33 – 4.24 (m, 2H), 3.62 – 3.52 (m, 2H), 1.57 – 1.35 (m, 4H), 1.23 (d, *J* = 2.5 Hz, 12H), 1.18 – 1.07 (m, 2H), 0.92 (d, *J* = 6.9 Hz, 3H), 0.89 (s, 9H), 0.04 (s, 6H). ^{13}C NMR (126 MHz, $CDCl_3$) δ 135.29, 133.23, 129.39, 127.89, 83.08, 63.68, 37.17, 36.32, 32.20, 31.18, 30.90, 26.02, 25.05, 24.90, 24.83, 18.33, 17.97, 9.01, 6.89, -5.22. The carbon signal attached to B was not observed. ^{11}B NMR (128 MHz, $CDCl_3$) δ 35.24. IR ν_{max} (DCM): 2956, 2925, 2857, 2126, 1652, 1471, 1379, 1257, 1119 cm^{-1}. HR-MS (EI) calcd for $C_{24}H_{44}BO_3Si$ $[M-H]^+$: 447.2917, found 447.2912.

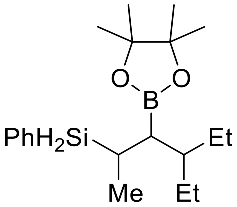

(4-Ethyl-3-(4,4,5,5-tetramethyl-1,3,2-dioxaborolan-2-yl)hexan-2-yl)(phenyl)silane (69). Following the general procedure I, the title compound (36.0 mg) was obtained in 52% yield. d.r. = 1:1. Colorless oil (eluent: hexane/EA = 100:1, R_f = 0.3). ^{1}H NMR (400 MHz, $CDCl_3$) δ 7.59 – 7.57 (m, 2H), 7.39 – 7.31 (m, 3H), 4.30 – 4.21 (m, 2H), 1.53 – 1.39 (m, 4H), 1.28 – 1.16 (m, 15H), 1.08 (d, *J* = 7.5 Hz, 3H), 0.80 (q, *J* = 7.3 Hz, 6H). ^{13}C NMR (126 MHz, $CDCl_3$) δ 135.72, 132.71, 129.32, 127.81, 82.98, 41.60, 25.24, 25.00, 24.97, 23.97, 15.91, 15.69, 11.94, 10.94. The carbon signal attached to B was not observed. ^{11}B NMR (128 MHz, $CDCl_3$) δ 34.45. IR ν_{max} (DCM): 2962, 2932, 2874,

2130, 1652, 1459, 1371, 1261, 1142 cm^{-1}. HR-MS (EI) calcd for $C_{20}H_{34}BO_2Si$ [M-H]$^+$: 345.2416, found 345.2426.

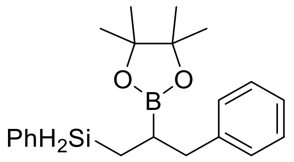

Phenyl(3-phenyl-2-(4,4,5,5-tetramethyl-1,3,2-dioxaborolan-2-yl)propyl)silane (70). Following the general procedure I, the title compound (52.1 mg) was obtained in 74% yield. Colorless oil (eluent: hexane/EA = 100:1, R_f = 0.3). ^{1}H NMR (400 MHz, $CDCl_3$) δ 7.59 – 7.55 (m, 2H), 7.37 – 7.31 (m, 3H), 7.26 – 7.21 (m, 2H), 7.18 – 7.12 (m, 3H), 4.40 – 4.29 (m, 2H), 2.74 – 2.53 (m, 2H), 2.05 – 1.94 (m, 1H), 1.82 – 1.74 (m, 1H), 1.19 (d, *J* = 10.6 Hz, 12H), 0.90 – 0.84 (m, 1H). ^{13}C NMR (126 MHz, $CDCl_3$) δ 135.55, 132.08, 129.60, 128.99, 128.54, 128.25, 127.87, 125.71, 83.15, 38.88, 29.08, 25.04, 24.54. The carbon signal attached to B was not observed. ^{11}B NMR (128 MHz, $CDCl_3$) δ 34.65. IR ν_{max} (DCM): 2976, 2929, 2857, 2128, 1652, 1428, 1354, 1261, 1143 cm^{-1}. HR-MS (EI) calcd for $C_{21}H_{29}BO_2Si$ [M]$^+$: 352.2024, found 352.2032.

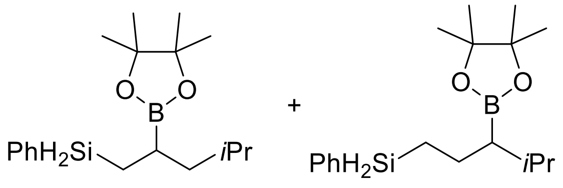

minor major

(4-Methyl-2-(4,4,5,5-tetramethyl-1,3,2-dioxaborolan-2-yl)pentyl)(phenyl)silane (71) and (4-Methyl-3-(4,4,5,5-tetramethyl-1,3,2-dioxaborolan-2-yl)pentyl)(phenyl)silane (71'). Following the general procedure I, the title compound (47.7 mg) was obtained in 75% yield. Colorless oil (eluent: hexane/EA = 100:1, R_f = 0.35). ^{1}H NMR (400 MHz, $CDCl_3$) δ 7.59 – 7.55 (m, 2H), 7.40 – 7.32 (m, 3H), 4.32 – 4.30 (m, 2 H) (**71**),4.28 (t, *J* = 3.6 Hz, 2H) (**71'**), 1.77 – 1.68 (m, 1H), 1.64 – 1.58 (m, 1H), 1.56 – 1.45 (m, 1H) , 1.25 (d, *J* = 1.5 Hz, 12H) (**71'**), 1.23 (s, 12H) (**71**), 1.00 – 0.89 (m, 9H) , 0.86 – 0.83 (m, 6H) (**71**). ^{13}C NMR (126 MHz, $CDCl_3$) δ 135.23, 132.79,

129.43, 127.93, 82.89, 43.28, 29.36, 26.96, 25.02, 24.86, 24.45, 22.77, 22.57, 22.32, 21.72, 11.39, 9.79. The carbon signal attached to B was not observed. ^{11}B NMR (128 MHz, $CDCl_3$) δ 34.99. IR ν_{max} (DCM): 2977, 2928, 2869, 2124, 1652, 1464, 1379, 1262, 1144 cm^{-1}. HR-MS (EI) calcd for $C_{18}H_{30}BO_2Si$ [M-H]$^+$: 317.2103, found 317.2099.

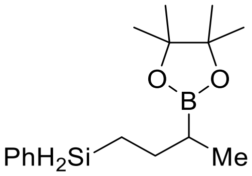

Phenyl(3-(4,4,5,5-tetramethyl-1,3,2-dioxaborolan-2-yl)butyl)silane (72). Following the general procedure I, the title compound (47.0 mg) was obtained in 81% yield. Colorless oil (eluent: hexane/EA = 100:1, R_f = 0.4). ^{1}H NMR (400 MHz, $CDCl_3$) δ 7.59 – 7.56 (m, 2H), 7.40 – 7.32 (m, 3H), 4.28 (t, *J* = 3.7 Hz, 2H), 1.67 – 1.57 (m, 2H), 1.50 – 1.39 (m, 1H), 1.23 (s, 12H), 1.09 – 1.03 (m, 1H), 1.00 – 0.93 (m, 4H). ^{13}C NMR (126 MHz, $CDCl_3$) δ 135.24, 132.80, 129.43, 127.93, 82.88, 28.52, 24.80, 24.75, 15.13, 9.27. The carbon signal attached to B was not observed. ^{11}B NMR (128 MHz, $CDCl_3$) δ 34.82. IR ν_{max} (DCM): 2977, 2926, 2871, 2123, 1643, 1463, 1371, 1261, 1144 cm^{-1}. HR-MS (EI) calcd for $C_{16}H_{26}BO_2Si$ [M-H]$^+$: 289.1790, found 289.1779.

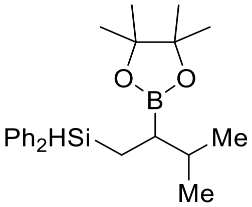

(3-Methyl-2-(4,4,5,5-tetramethyl-1,3,2-dioxaborolan-2-yl)butyl)diphenylsilane (73). Following the general procedure I, the title compound (49.4 mg) was obtained in 65% yield. Colorless oil (eluent: hexane/EA = 100:1, R_f = 0.4). ^{1}H NMR (400 MHz, $CDCl_3$) δ 7.58 – 7.56 (m, 4H), 7.38 – 7.31 (m, 6H), 4.86 – 4.84 (m, 1H), 1.82 – 1.73 (m, 1H), 1.23 – 1.09 (m, 15H), 0.93 (dd, *J* = 6.8, 3.5 Hz, 6H). ^{13}C NMR (126 MHz, $CDCl_3$) δ 135.31, 135.21, 134.81, 129.39, 129.34, 127.92, 127.85, 83.06, 32.20, 24.99, 24.97, 21.72, 20.93, 10.59. The carbon signal attached to B was not observed. ^{11}B NMR

(128 MHz, $CDCl_3$) δ 34.68. IR ν_{max} (DCM): 2977, 2957, 2870, 2119, 1589, 1464, 1379, 1214, 1144 cm^{-1}. HR-MS (EI) calcd for $C_{23}H_{32}BO_2Si$ $[M-H]^+$: 379.2259, found 379.2276.

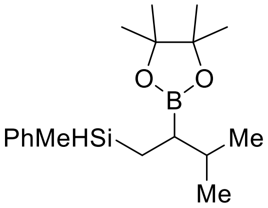

Methyl(3-methyl-2-(4,4,5,5-tetramethyl-1,3,2-dioxaborolan-2-yl)butyl)(phenyl)silane (74). Following the general procedure I, the title compound (45.2 mg) was obtained in 71% yield. d.r. = 1.2:1. Colorless oil (eluent: hexane/EA = 100:1, R_f = 0.25). ^1H NMR (400 MHz, $CDCl_3$) δ 7.57 – 7.52 (m, 2H), 7.37 – 7.31 (m, 3H), 4.37 – 4.31 (m, 1H), 1.78 – 1.69 (m, 1H), 1.22 (s, 12H), 1.09 – 0.99 (m, 2H), 0.92 – 0.89 (m, 6H), 0.84 – 0.77 (m, 1H), 0.33 (dd, J = 3.8, 1.1 Hz, 3H). ^{13}C NMR (126 MHz, $CDCl_3$) δ 134.43, 134.38, 129.06, 127.77, 83.01, 82.99, 32.16, 32.05, 25.00, 24.97, 21.71, 21.05, 20.88, 11.81, 11.75, -4.85, -5.62. The carbon signal attached to B was not observed. ^{11}B NMR (128 MHz, $CDCl_3$) δ 34.37. IR ν_{max} (DCM): 2959, 2930, 2871, 2114, 1652, 1371, 1318, 1261, 1144 cm^{-1}. HR-MS (EI) calcd for $C_{18}H_{30}BO_2Si$ $[M-H]^+$: 317.2103, found 317.2113.

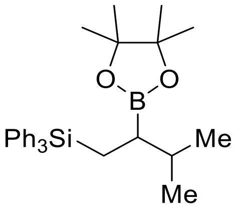

(3-Methyl-2-(4,4,5,5-tetramethyl-1,3,2-dioxaborolan-2-yl)butyl)triphenylsilane (75). Following the general procedure I, the title compound (68.4 mg) was obtained in 75% yield. sticky oil (eluent: hexane/EA = 50:1, R_f = 0.2). ^1H NMR (400 MHz, $CDCl_3$) δ 7.56 – 7.52 (m, 6H), 7.39 – 7.30 (m, 9H), 1.74 – 1.67 (m, 2H), 1.36 – 1.30 (m, 2H), 1.02 (d, J = 9.5 Hz, 12H), 0.89 (dd, J = 6.8, 5.7 Hz, 6H). ^{13}C NMR (126 MHz, $CDCl_3$) δ 135.98, 135.69, 129.18, 127.69, 82.93, 32.98, 24.92, 24.86, 21.74, 20.47, 11.23. The

carbon signal attached to B was not observed. ^{11}B NMR (128 MHz, $CDCl_3$) δ 33.93. IR ν_{max} (DCM): 2963, 2929, 2870, 1652, 1464, 1372, 1261, 1108 cm^{-1}. HR-MS (ESI) calcd for $C_{29}H_{37}BNaO_2Si$ $[M+Na]^+$: 479.2548, found 479.2554.

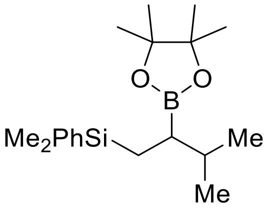

Dimethyl(3-methyl-2-(4,4,5,5-tetramethyl-1,3,2-dioxaborolan-2-yl)butyl)(phenyl)silane (76). Following the general procedure I, the title compound (47.2 mg) was obtained in 71% yield. Colorless oil (eluent: hexane/EA = 50:1, R_f = 0.25). ^1H NMR (400 MHz, $CDCl_3$) δ 7.53 – 7.50 (m, 2H), 7.34 – 7.31 (m, 3H), 1.74 – 1.69 (m, 1H), 1.19 (s, 12H), 1.12 – 0.98 (m, 3H), 0.88 (dd, *J* = 6.8, 1.7 Hz, 6H), 0.26 (d, *J* = 0.9 Hz, 6H). ^{13}C NMR (126 MHz, $CDCl_3$) δ 133.71, 128.63, 127.61, 82.89, 32.25, 25.09, 24.98, 21.43, 20.85, 13.12, -2.38, -2.76. The carbon signal attached to B was not observed. ^{11}B NMR (128 MHz, $CDCl_3$) δ 34.03. IR ν_{max} (DCM): 2956, 2870, 1645, 1464, 1371, 1316, 1249, 1144, 1112 cm^{-1}. HR-MS (ESI) calcd for $C_{19}H_{33}BNaO_2Si$ $[M+Na]^+$: 355.2235, found 355.2240.

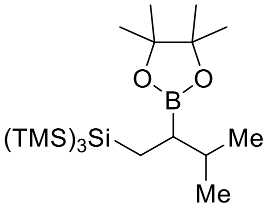

1,1,1,3,3,3-Hexamethyl-2-(3-methyl-2-(4,4,5,5-tetramethyl-1,3,2-dioxaborolan-2-yl)butyl)-2-(trimethylsilyl)trisilane (77). Following the general procedure I, the title compound (76.4 mg) was obtained in 86% yield. Colorless oil (eluent: hexane/EA = 100:1, R_f = 0.4). ^1H NMR (400 MHz, $CDCl_3$) δ 1.75 – 1.67 (m, 1H), 1.24 (d, *J* = 1.3 Hz, 12H), 1.09 – 1.03 (m, 2H), 0.94 (dd, *J* = 19.9, 6.8 Hz, 6H), 0.81 – 0.73 (m, 1H), 0.16 (s, 27H). ^{13}C NMR (126 MHz, $CDCl_3$) δ 82.87, 32.13, 25.18, 24.95, 22.84, 19.74, 5.69, 1.45. The carbon signal attached to B was not observed. ^{11}B NMR (128 MHz,

$CDCl_3$) δ 34.79. IR ν_{max} (DCM): 2952, 2894, 1651, 1371, 1312, 1244, 1215, 1144 cm^{-1}. HR-MS (EI) calcd for $C_{20}H_{49}BO_2Si_4$ $[M]^+$: 444.2897, found 444.2892.

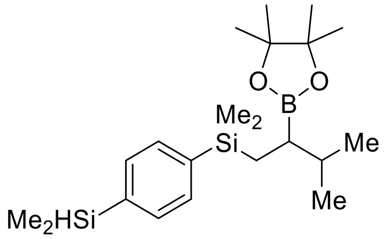

(4-(Dimethylsilyl)phenyl)dimethyl(3-methyl-2-(4,4,5,5-tetramethyl-1,3,2-dioxaborolan-2-yl)butyl)silane (78). Following the general procedure I, the title compound (64.0 mg) was obtained in 82% yield. Colorless oil (eluent: hexane/EA = 50:1, R_f = 0.3). ^1H NMR (400 MHz, $CDCl_3$) δ 7.53 – 7.49 (m, 4H), 4.43 – 4.38 (m, 1H), 1.76 – 1.68 (m, 1H), 1.17 (s, 12H), 1.06 – 0.94 (m, 2H), 0.89 (d, *J* = 6.8 Hz, 6H), 0.76 – 0.67 (m, 1H), 0.33 (d, *J* = 3.7 Hz, 6H), 0.26 (d, *J* = 1.3 Hz, 6H). ^{13}C NMR (126 MHz, $CDCl_3$) δ 141.17, 137.69, 133.17, 133.15, 82.89, 32.31, 25.10, 24.96, 21.42, 20.87, 13.09, -2.53, -2.76, -3.83. The carbon signal attached to B was not observed. ^{11}B NMR (128 MHz, $CDCl_3$) δ 34.73. IR ν_{max} (DCM): 2957, 2807, 2119, 1652, 1464, 1378, 1316, 1249, 1135 cm^{-1}. HR-MS (ESI) calcd for $C_{21}H_{39}BNaO_2Si_2$ $[M+Na]^+$: 413.2474, found 413.2472.

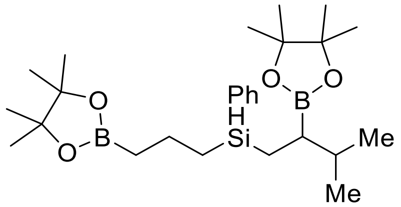

(3-Methyl-2-(4,4,5,5-tetramethyl-1,3,2-dioxaborolan-2-yl)butyl)(phenyl)(3-(4,4,5,5-tetramethyl-1,3,2-dioxaborolan-2-yl)propyl)silane (79). Following the general procedure I, the title compound (76.5 mg) was obtained in 81% yield. d.r. = 1:1. Colorless oil (eluent: hexane/EA = 50:1, R_f = 0.2). ^1H NMR (400 MHz, $CDCl_3$) δ 7.57 – 7.51 (m, 2H), 7.35 – 7.29 (m, 3H), 4.28 – 4.21 (m, 1H), 1.77 – 1.66 (m, 1H), 1.56 – 1.47 (m, 2H), 1.27 – 1.15 (m, 24H), 1.05 – 0.96 (m, 2H), 0.93 – 0.80 (m, 11H). ^{13}C NMR (126 MHz, $CDCl_3$) δ 134.82, 134.79, 128.92, 127.70, 127.67, 82.95, 82.83, 32.14,

25.03, 24.98, 24.84, 21.74, 20.98, 20.92, 19.25, 19.20, 15.80, 15.07, 10.39, 10.33. The carbon signal attached to B was not observed. ^{11}B NMR (128 MHz, $CDCl_3$) δ 34.59. IR ν_{max} (DCM): 2978, 2928, 2872, 2109, 1652, 1371, 1315, 1261, 1214, 1145 cm^{-1}. HR-MS (ESI) calcd for $C_{26}H_{46}B_2NaO_4Si$ $[M+Na]^+$: 495.3244, found 495.3242.

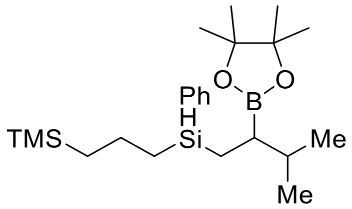

Trimethyl(3-((3-methyl-2-(4,4,5,5-tetramethyl-1,3,2-dioxaborolan-2-yl)butyl)(phenyl)silyl)propyl)silane (80). Following the general procedure I, the title compound (72.8 mg) was obtained in 87% yield. d.r. = 1:1. Colorless oil (eluent: hexane/EA = 50:1, R_f = 0.3). ^{1}H NMR (400 MHz, $CDCl_3$) δ 7.56 – 7.50 (m, 2H), 7.36 – 7.30 (m, 3H), 4.28 – 4.23 (m, 1H), 1.78 – 1.67 (m, 1H), 1.49 – 1.34 (m, 2H), 1.27 – 1.17 (m, 12H), 1.07 – 0.97 (m, 2H), 0.95 – 0.82 (m, 9H), 0.60 – 0.55 (m, 2H), -0.06 (d, *J* = 2.0 Hz, 9H). ^{13}C NMR (126 MHz, $CDCl_3$) δ 134.78, 134.74, 128.97, 127.73, 127.70, 82.97, 32.15, 25.04, 24.98, 21.73, 21.70, 21.08, 20.96, 20.88, 19.18, 19.11, 17.05, 16.41, 10.31, 10.26, -1.57. The carbon signal attached to B was not observed. ^{11}B NMR (128 MHz, $CDCl_3$) δ 34.77. IR ν_{max} (DCM): 2977, 2954, 2872, 2110, 1647, 1372, 1317, 1247, 1214, 1144 cm^{-1}. HR-MS (EI) calcd for $C_{23}H_{42}BO_2Si_2$ $[M-H]^+$: 417.2811, found 417.2811.

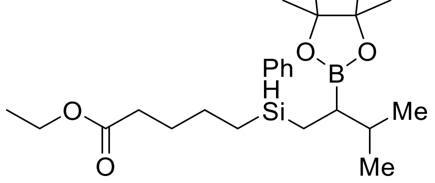

Ethyl 5-((3-methyl-2-(4,4,5,5-tetramethyl-1,3,2-dioxaborolan-2-yl)butyl)(phenyl)silyl)pentanoate (81). Following the general procedure I, the title compound (76.1 mg) was obtained in 88% yield. d.r. = 1:1. Colorless oil (eluent: hexane/EA = 50:1, R_f = 0.2). ^{1}H NMR (400 MHz, $CDCl_3$) δ 7.53 – 7.49 (m, 2H), 7.37

– 7.29 (m, 3H), 4.28 – 4.22 (m, 1H), 4.09 (qd, J = 7.1, 0.9 Hz, 2H), 2.28 – 2.24 (m, 2H), 1.76 – 1.62 (m, 3H), 1.46 – 1.33 (m, 2H), 1.26 – 1.17 (m, 15H), 1.05 – 0.94 (m, 2H), 0.96 – 0.79 (m, 9H). ^{13}C NMR (126 MHz, $CDCl_3$) δ 173.80, 136.04, 135.87, 134.74, 134.71, 129.09, 127.79, 127.76, 83.00, 60.15, 34.09, 32.14, 28.50, 28.46, 25.02, 24.97, 24.22, 24.16, 21.71, 21.67, 21.01, 20.91, 14.24, 12.42, 11.70, 10.29, 10.13. The carbon signal attached to B was not observed. ^{11}B NMR (128 MHz, $CDCl_3$) δ 34.39. IR ν_{max} (DCM): 2978, 2958, 2870, 2111, 1736, 1647, 1372, 1317, 1261, 1144 cm^{-1}. HR-MS (APCI) calcd for $C_{24}H_{40}BO_4Si$ [M-H]$^+$: 431.2783, found 431.2792.

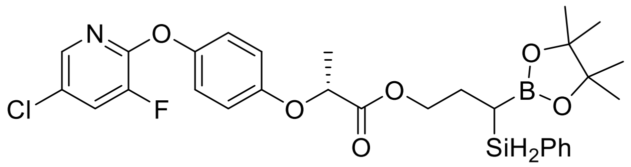

3-(Phenylsilyl)-3-(4,4,5,5-tetramethyl-1,3,2-dioxaborolan-2-yl)propyl (2R)-2-(4-((5-chloro-3-fluoropyridin-2-yl)oxy)phenoxy)propanoate (82). d.r. = 1:1. ^{1}H NMR (400 MHz, $CDCl_3$) δ 7.86 (d, J = 2.2 Hz, 1H), 7.60 – 7.57 (m, 2H), 7.48 (dd, J = 9.1, 2.2 Hz, 1H), 7.39 – 7.32 (m, 3H), 7.07 – 7.03 (m, 2H), 6.91 – 6.86 (m, 2H), 4.69 (qd, J = 6.8, 1.7 Hz, 1H), 4.40 – 4.34 (m, 2H), 4.23 – 4.11 (m, 2H), 2.01 – 1.91 (m, 1H), 1.87 – 1.78 (m, 1H), 1.60 (dd, J = 6.8, 3.1 Hz, 3H), 1.21 – 1.11 (m, 12H), 0.89 – 0.81 (m, 1H). ^{13}C NMR (126 MHz, $CDCl_3$) δ 172.02, 171.98, 154.99, 151.42, 151.33, 147.03 (d, J_{C-F} = 266.1 Hz), 147.04, 140.20, 140.15, 135.52, 131.51, 129.83, 127.98, 124.98, 124.83, 122.25, 116.17, 116.12, 83.40, 73.22, 73.18, 66.56, 66.54, 25.91, 25.86, 24.96, 24.53, 18.67, 18.63. The carbon signal attached to B was not observed. ^{19}F NMR (377 MHz, $CDCl_3$) δ -134.35. ^{11}B NMR (128 MHz, $CDCl_3$) δ 33.99. IR ν_{max} (DCM): 2978, 2931, 2135, 1754, 1504, 1450, 1354, 1208, 1142 cm^{-1}. HR-MS (EI) calcd for $C_{29}H_{34}BClFNO_6Si$ [M]$^+$: 585.1916, found 585.1910.

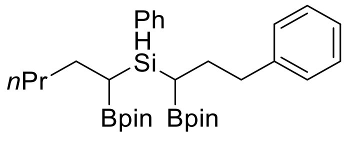

Phenyl(3-phenyl-1-(4,4,5,5-tetramethyl-1,3,2-dioxaborolan-2-yl)propyl)(1-(4,4,5,5-tetramethyl-1,3,2-dioxaborolan-2-yl)pentyl)silane (83). d.r. = 1:1:1:1. ^{1}H NMR (400 MHz, $CDCl_3$) δ 7.61 – 7.53 (m, 2H), 7.32 – 7.09 (m, 8H), 4.36 – 4.29 (m, 1H), 2.78 – 2.66 (m, 1H), 2.60 – 2.45 (m, 1H), 1.99 – 1.84 (m, 1H), 1.61 – 1.53 (m, 1H), 1.31 – 0.98 (m, 30H), 0.90 – 0.78 (m, 5H). ^{13}C NMR (126 MHz, $CDCl_3$) δ 142.65, 142.52, 135.56, 135.43, 129.15, 129.12, 128.62, 128.59, 128.57, 128.22, 128.19, 128.16, 127.45, 127.38, 127.34, 125.62, 125.57, 125.54, 82.87, 82.82, 82.75, 82.68, 39.37, 39.28, 39.20, 39.05, 35.37, 35.30, 35.25, 29.14, 28.84, 28.18, 28.10, 26.56, 26.25, 25.80, 25.70, 25.21, 25.12, 25.08, 25.01, 24.98, 24.95, 24.92, 24.79, 24.70, 24.63, 24.59, 24.54, 24.52, 24.50, 22.54, 22.50, 22.47, 14.04. The carbon signal attached to B was not observed. ^{11}B NMR (128 MHz, $CDCl_3$) δ 33.59. IR ν_{max} (DCM): 2977, 2927, 2857, 2111, 1653, 1349, 1309, 1260, 1144 cm^{-1}. HR-MS (APCI) calcd for $C_{32}H_{49}B_2O_4Si$ [M-H]$^{+}$: 547.3581, found 547.3588.

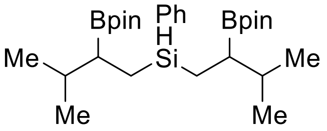

Bis(3-methyl-2-(4,4,5,5-tetramethyl-1,3,2-dioxaborolan-2-yl)butyl)(phenyl)silane (84). ^{1}H NMR (400 MHz, $CDCl_3$) δ 7.58 – 7.50 (m, 2H), 7.33 – 7.28 (m, 3H), 4.27 – 4.22 (m, 1H), 1.79 – 1.67 (m, 2H), 1.23 – 1.14 (m, 24H), 1.08 – 1.02 (m, 2H), 0.93 – 0.80 (m, 16H). ^{13}C NMR (126 MHz, $CDCl_3$) δ 136.49, 136.27, 134.97, 134.94, 128.85, 127.66, 127.62, 127.58, 82.86, 32.18, 32.05, 32.01, 25.00, 24.97, 24.92, 21.84, 21.74, 20.92, 20.84, 20.79, 10.92, 10.81, 10.74, 10.60. The carbon signal attached to B was not observed. ^{11}B NMR (128 MHz, $CDCl_3$) δ 33.61. IR ν_{max} (DCM): 2977, 2956, 2871, 2111, 1643, 1465, 1372, 1262, 1145 cm^{-1}. HR-MS (APCI) calcd for $C_{28}H_{49}B_2O_4Si$ [M-H]$^{+}$: 499.3581, found 499.3597.

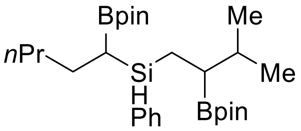

(3-Methyl-2-(4,4,5,5-tetramethyl-1,3,2-dioxaborolan-2-yl)butyl)(phenyl)(1-(4,4,5,5-tetramethyl-1,3,2-dioxaborolan-2-yl)pentyl)silane (85). d.r. = 1:1:1:1. ^{1}H NMR (400 MHz, $CDCl_3$) δ 7.63 – 7.50 (m, 2H), 7.32 – 7.27 (m, 3H), 4.37 – 4.22 (m, 1H), 1.68 – 1.51 (m, 2H), 1.34 – 1.00 (m, 32H), 0.96 – 0.79 (m, 9H), 0.75 – 0.65 (m, 1H). ^{13}C NMR (126 MHz, $CDCl_3$) δ 135.56, 135.26, 135.19, 135.07, 128.98, 128.96, 128.93, 127.59, 127.53, 127.48, 127.32, 127.28, 82.87, 82.84, 82.78, 82.73, 82.70, 82.67, 35.36, 35.31, 35.28, 32.17, 32.15, 32.06, 26.27, 26.22, 26.19, 26.11, 25.15, 25.10, 25.04, 25.01, 24.99, 24.96, 24.94, 24.92, 24.87, 24.79, 24.68, 24.65, 24.62, 24.58, 24.55, 24.53, 22.51, 22.45, 21.85, 21.78, 21.66, 21.64, 20.99, 20.95, 20.86, 14.03, 10.38, 10.31, 10.27, 10.19. The carbon signal attached to B was not observed. ^{11}B NMR (128 MHz, $CDCl_3$) δ 33.71. IR ν_{max} (DCM): 2977, 2927, 2872, 2111, 1653, 1456, 1371, 1261, 1144 cm^{-1}. HR-MS (APCI) calcd for $C_{28}H_{49}B_2O_4Si$ $[M\text{-}H]^+$: 499.3581, found 499.3599.

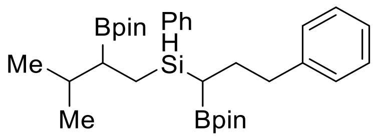

(3-Methyl-2-(4,4,5,5-tetramethyl-1,3,2-dioxaborolan-2-yl)butyl)(phenyl)(3-phenyl-1-(4,4,5,5-tetramethyl-1,3,2-dioxaborolan-2-yl)propyl)silane (86). d.r. = 1:1:1:1. ^{1}H NMR (400 MHz, $CDCl_3$) δ 7.58 – 7.47 (m, 2H), 7.34 – 7.27 (m, 3H), 7.25 – 7.20 (m, 2H), 7.17 – 7.09 (m, 3H), 4.33 – 4.26 (m, 1H), 2.74 – 2.65 (m, 1H), 2.56 – 2.44 (m, 1H), 1.98 – 1.86 (m, 1H), 1.80 – 1.62 (m, 2H), 1.23 – 1.11 (m, 24H), 1.07 – 1.01 (m, 1H), 0.94 – 0.83 (m, 8H), 0.78 – 0.74 (m, 1H). ^{13}C NMR (126 MHz, $CDCl_3$) δ 142.59, 135.25, 135.20, 135.09, 129.01, 128.56, 128.18, 127.64, 127.60, 127.54, 125.58, 82.86, 39.29, 39.19, 32.14, 32.03, 28.77, 28.53, 25.23, 25.17, 25.13, 25.02, 24.98, 24.91, 24.86, 24.72, 24.63, 21.84, 21.78, 21.63, 20.98, 20.83, 10.34, 10.24, 10.12, 10.04. The carbon signal attached to B was not observed. ^{11}B NMR (128 MHz, $CDCl_3$) δ 34.88. IR ν_{max} (DCM): 2977, 2928, 2868, 2112, 1604, 1371, 1313, 1262, 1144 cm^{-1}. HR-MS (APCI) calcd for $C_{32}H_{49}B_2O_4Si$ $[M\text{-}H]^+$: 547.3581, found 547.3593.

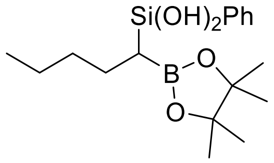

Phenyl(1-(4,4,5,5-tetramethyl-1,3,2-dioxaborolan-2-yl)pentyl)silanediol (87). ^{1}H NMR (400 MHz, $CDCl_3$) δ 7.71 – 7.65 (m, 2H), 7.44 – 7.30 (m, 3H), 3.64 (s, 2H), 1.67 – 1.41 (m, 2H), 1.36 – 1.23 (m, 4H), 1.20 (d, *J* = 7.7 Hz, 12H), 0.81 (t, *J* = 7.1 Hz, 3H), 0.76 (dd, *J* = 10.9, 4.2 Hz, 1H). ^{13}C NMR (101 MHz, $CDCl_3$) δ 135.13, 134.15, 130.08, 127.74, 83.44, 35.12, 24.90, 24.48, 24.28, 22.49, 13.96. The carbon signal attached to B was not observed. ^{11}B NMR (128 MHz, $CDCl_3$) δ 35.38. IR ν_{max} (DCM): 2977, 2928, 2858, 2110, 1466, 1352, 1307, 1263, 1214, 1145 cm^{-1}. HR-MS (EI) calcd for $C_{17}H_{28}BO_4Si$ $[M\text{-}H]^+$: 335.1844, found 335.1850.

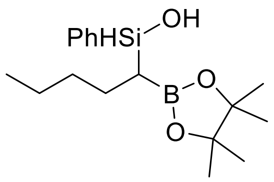

Phenyl(1-(4,4,5,5-tetramethyl-1,3,2-dioxaborolan-2-yl)pentyl)silanol (88). d.r. = 1:1. ^{1}H NMR (400 MHz, $CDCl_3$) δ 7.65 – 7.60 (m, 2H), 7.45 – 7.32 (m, 3H), 5.02 (d, *J* = 24.5 Hz, 1H), 2.45 (d, *J* = 5.0 Hz, 1H), 1.69 – 1.59 (m, 1H), 1.50 – 1.43 (m, 1H), 1.40 – 1.23 (m, 4H), 1.24 – 1.18 (m, 12H), 0.86 – 0.81 (m, 4H). ^{13}C NMR (126 MHz, $CDCl_3$) δ 133.95, 133.86, 130.06, 127.87, 127.84, 83.26, 35.02, 34.89, 24.97, 24.60, 24.58, 24.40, 24.06, 22.51, 13.97, 13.94. The carbon signal attached to B was not observed. ^{11}B NMR (128 MHz, $CDCl_3$) δ 34.55. IR ν_{max} (DCM): 2963, 2928, 2858, 2123, 1615, 1351, 1307, 1261, 1145 cm^{-1}. HR-MS (EI) calcd for $C_{17}H_{28}BO_3Si$ $[M\text{-}H]^+$: 319.1895, found 319.1886.

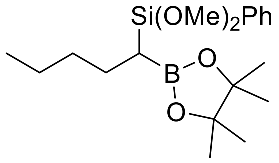

Dimethoxy(phenyl)(1-(4,4,5,5-tetramethyl-1,3,2-dioxaborolan-2-yl)pentyl)silane (89). ^{1}H NMR (400 MHz, $CDCl_3$) δ 7.67 – 7.64 (m, 2H), 7.40 – 7.32 (m, 3H), 3.58 (d, *J* = 7.6 Hz, 6H), 1.25 – 1.16 (m, 18H), 0.85 – 0.80 (m, 4H). ^{13}C NMR (126 MHz, $CDCl_3$) δ 134.66, 132.91, 129.94, 127.70, 82.95, 50.75, 35.27, 24.97, 24.56, 24.44, 22.48, 13.99. The carbon signal attached to B was not observed. ^{11}B NMR (128 MHz, $CDCl_3$) δ 35.29. IR ν_{max} (DCM): 2958, 2930, 2870, 1653, 1349, 1307, 1260, 1145, 1120 cm^{-1}. HR-MS (EI) calcd for $C_{19}H_{33}BO_4Si$ $[M]^+$: 364.2236, found 364.2246.

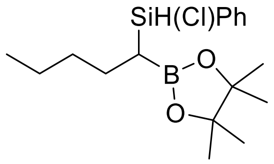

Chloro(phenyl)(1-(4,4,5,5-tetramethyl-1,3,2-dioxaborolan-2-yl)pentyl)silane (90). d.r. = 1.3:1. ^{1}H NMR (400 MHz, $CDCl_3$) δ 7.63 – 7.58 (m, 2H), 7.37 – 7.28 (m, 3H), 5.12 – 5.07 (m, 1H), 1.36 – 1.16 (m, 6H), 1.17 – 1.00 (m, 12H), 0.86 – 0.76 (m, 4H). ^{13}C NMR (126 MHz, $CDCl_3$) δ 134.09, 132.60, 129.65, 127.55, 127.52, 82.79, 35.13, 25.00, 24.95, 24.55, 24.43, 22.50, 13.97. The carbon signal attached to B was not observed. ^{11}B NMR (128 MHz, $CDCl_3$) δ 35.28. IR ν_{max} (DCM): 2961, 2927, 2857, 2115, 1653, 1351, 1310, 1261, 1145 cm^{-1}. HR-MS (EI) calcd for $C_{17}H_{27}BClO_2Si$ $[M-H]^+$: 337.1556, found 337.1566.

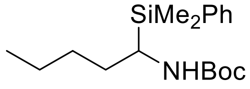

tert-Butyl (1-(dimethyl(phenyl)silyl)pentyl)carbamate (91). ^{1}H NMR (400 MHz, $CDCl_3$) δ 7.55 – 7.48 (m, 2H), 7.42 – 7.32 (m, 3H), 4.14 (d, *J* = 10.4 Hz, 1H), 3.33 (td, *J* = 10.4, 4.0 Hz, 1H), 1.49 – 1.45 (m, 1H), 1.41 (s, 9H), 1.31 – 1.19 (m, 5H), 0.87 – 0.81 (m, 3H), 0.32 (d, *J* = 2.0 Hz, 6H). ^{13}C NMR (126 MHz, $CDCl_3$) δ 156.23, 136.57, 134.05, 129.30, 127.89, 78.77, 40.39, 31.22, 29.34, 28.43, 22.46, 14.00, -4.57, -5.18. IR ν_{max} (DCM): 2959, 2929, 2858, 1695, 1495, 1365, 1259, 1214, 1111 cm^{-1}. HR-MS (APCI) calcd for $C_{18}H_{32}NO_2Si$ $[M+H]^+$: 322.2197, found 322.2192.

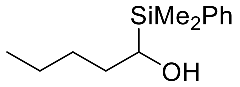

1-(Dimethyl(phenyl)silyl)pentan-1-ol (92). ^{1}H NMR (400 MHz, $CDCl_3$) δ 7.58 – 7.55 (m, 2H), 7.40 – 7.36 (m, 3H), 3.53 – 3.49 (m, 1H), 1.56 – 1.50 (m, 3H), 1.36 – 1.23 (m, 3H), 1.06 (d, *J* = 1.1 Hz, 1H), 0.88 (t, *J* = 7.1 Hz, 3H), 0.34 (d, *J* = 4.0 Hz, 6H). ^{13}C NMR (126 MHz, $CDCl_3$) δ 136.83, 134.15, 129.32, 127.91, 65.51, 33.09, 29.04, 22.55, 14.06, -5.33, -5.64. IR ν_{max} (DCM): 2957, 2928, 2871, 2858, 1645, 1465, 1427, 1249, 1112 cm^{-1}. HR-MS (EI) calcd for $C_{13}H_{21}OSi$ $[M-H]^+$: 221.1356, found 221.1354.

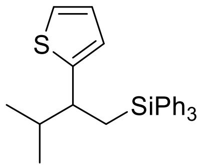

(3-Methyl-2-(thiophen-2-yl)butyl)triphenylsilane (93). ^{1}H NMR (400 MHz, $CDCl_3$) δ 7.33 – 7.30 (m, 6H), 7.28 – 7.24 (m, 3H), 7.21 – 7.17 (m, 6H), 6.88 – 6.86 (m, 1H), 6.61 – 6.59 (m, 1H), 6.36 – 6.35 (m, 1H), 3.00 – 2.94 (m, 1H), 1.88 – 1.65 (m, 3H), 0.73 (dd, *J* = 21.0, 6.7 Hz, 6H). ^{13}C NMR (101 MHz, $CDCl_3$) δ 148.59, 135.73, 135.15, 129.25, 127.74, 125.99, 124.89, 122.63, 43.06, 36.19, 20.69, 19.32, 19.02. IR ν_{max} (DCM): 3068, 2958, 2925, 2871, 1821, 1656, 1427, 1384, 1263, 1109 cm^{-1}. HR-MS (APCI) calcd for $C_{27}H_{29}SSi$ $[M+H]^+$: 413.1754, found 413.1760.

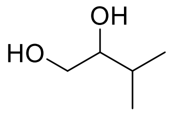

3-Methylbutane-1,2-diol (94). ^{1}H NMR (500 MHz, $CDCl_3$) δ 3.75 – 3.70 (m, 1H), 3.54 – 3.50 (m, 1H), 3.46 – 3.42 (m, 1H), 2.04 (br, 2H), 1.71 (h, *J* = 6.8 Hz, 1H), 0.95 (dd, *J* = 28.9, 6.8 Hz, 6H). The NMR data were consistent with literature reports[43].

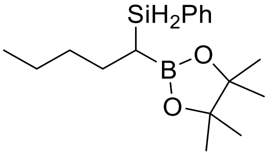

Phenyl(1-(4,4,5,5-tetramethyl-1,3,2-dioxaborolan-2-yl)pentyl)silane (95). Following the general procedure I, the title compound (54.1 mg) was obtained in 89% yield. Colorless oil. ^{1}H NMR (400 MHz, $CDCl_3$) δ 7.61 (dd, *J* = 7.7, 1.7 Hz, 2H), 7.41 – 7.30 (m, 3H), 4.34 (qd, *J* = 6.4, 3.6 Hz, 2H), 1.72 – 1.62 (m, 1H), 1.53 – 1.43 (m, 1H), 1.40 – 1.24 (m, 4H), 1.18 (d, *J* = 13.1 Hz, 12H), 0.85 (t, *J* = 7.1 Hz, 3H), 0.82 – 0.77 (m, 1H). ^{13}C NMR (126 MHz, $CDCl_3$) δ 135.53, 132.43, 129.52, 127.83, 83.01, 35.02, 26.68, 24.96, 24.49, 22.47, 13.98. The carbon signal attached to B was not observed. ^{11}B NMR (160 MHz, $CDCl_3$) δ 34.19. ^{29}Si NMR (99 MHz, $CDCl_3$) δ -28.10. IR ν_{max} (DCM): 2977, 2927, 2858, 2128, 1465, 1351, 1310, 1261, 1144 cm^{-1}. HR-MS (EI) calcd for $C_{17}H_{28}BO_2Si$ $[M-H]^+$: 303.1946, found 303.1960.

Supplementary Note 2

NMR spectra

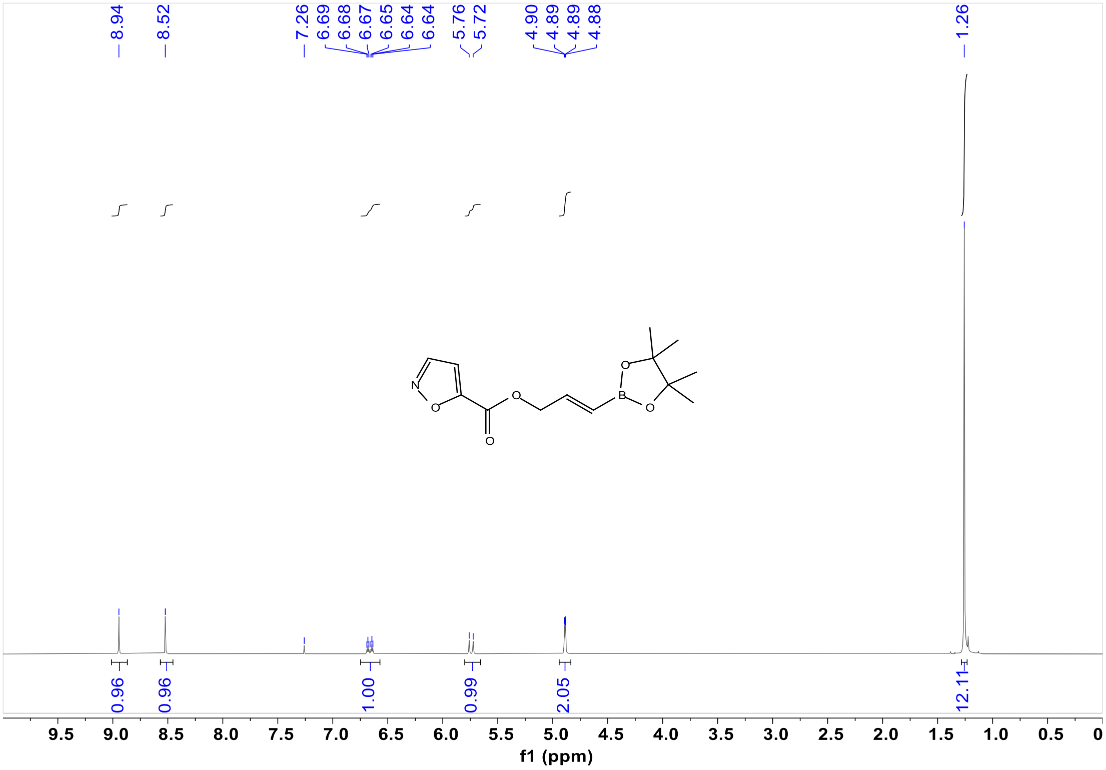

Supplementary Figure 26. ^{1}H NMR spectra for **S1**

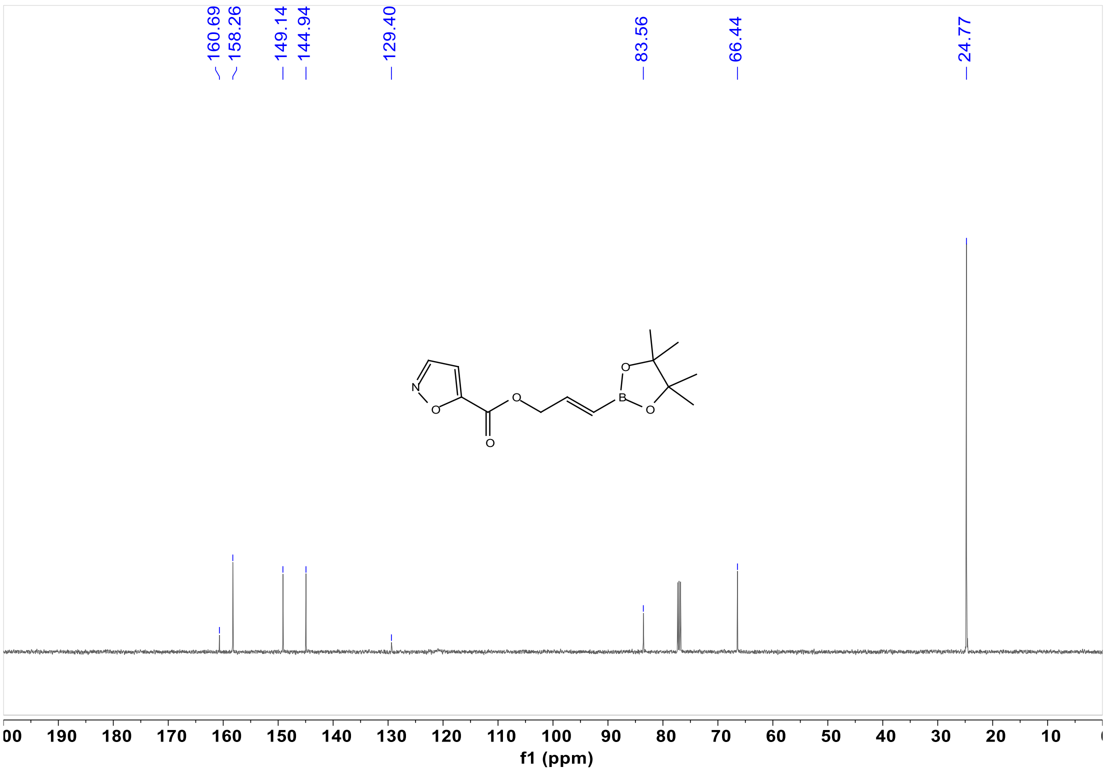

Supplementary Figure 27. ^{13}C NMR spectra for **S1**

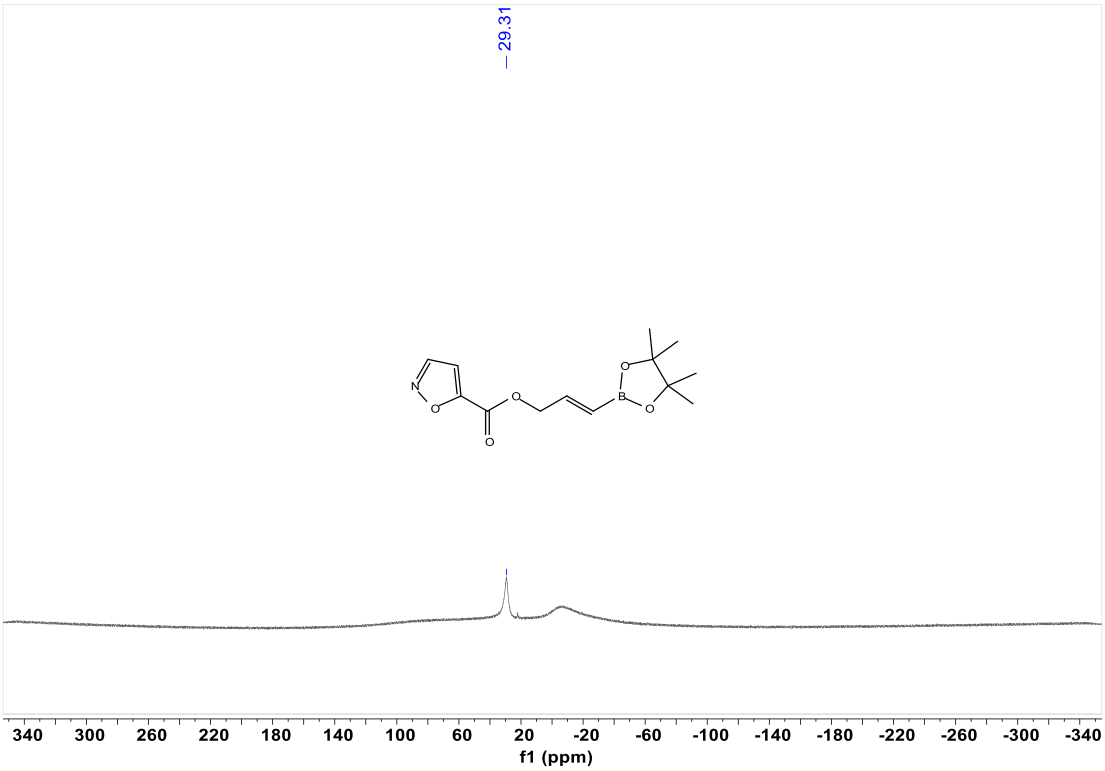

Supplementary Figure 28. ^{11}B NMR spectra for **S1**

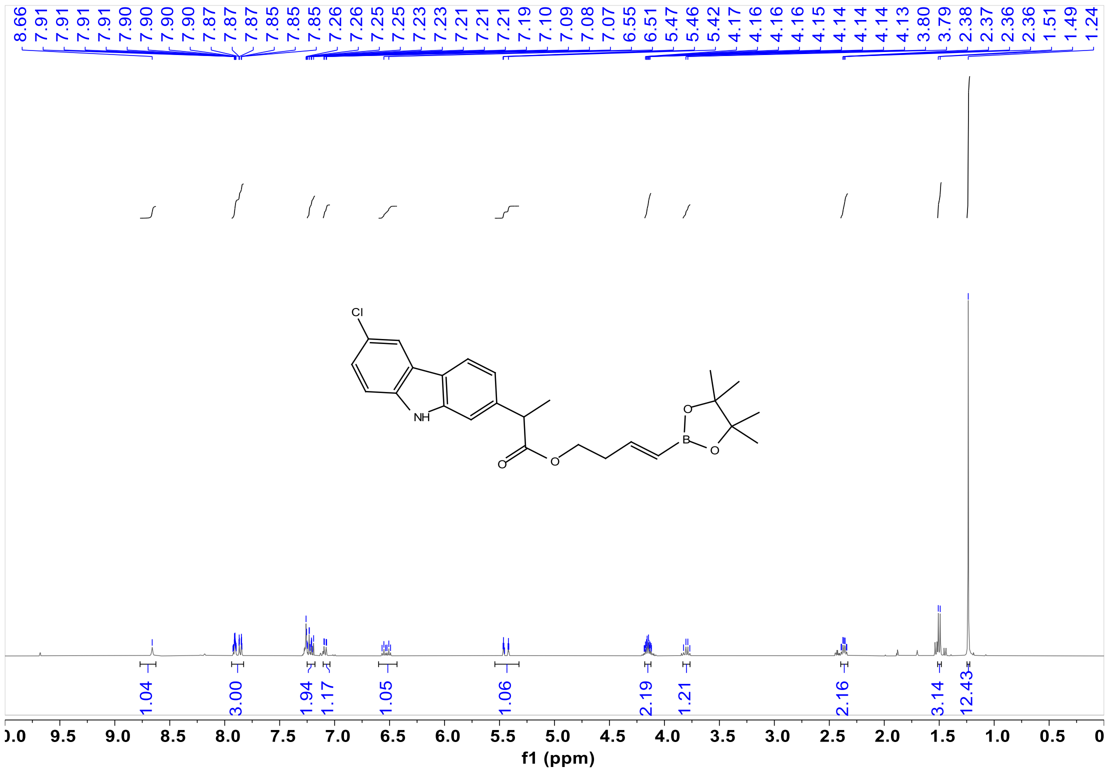

Supplementary Figure 29. ^{1}H NMR spectra for **S2**

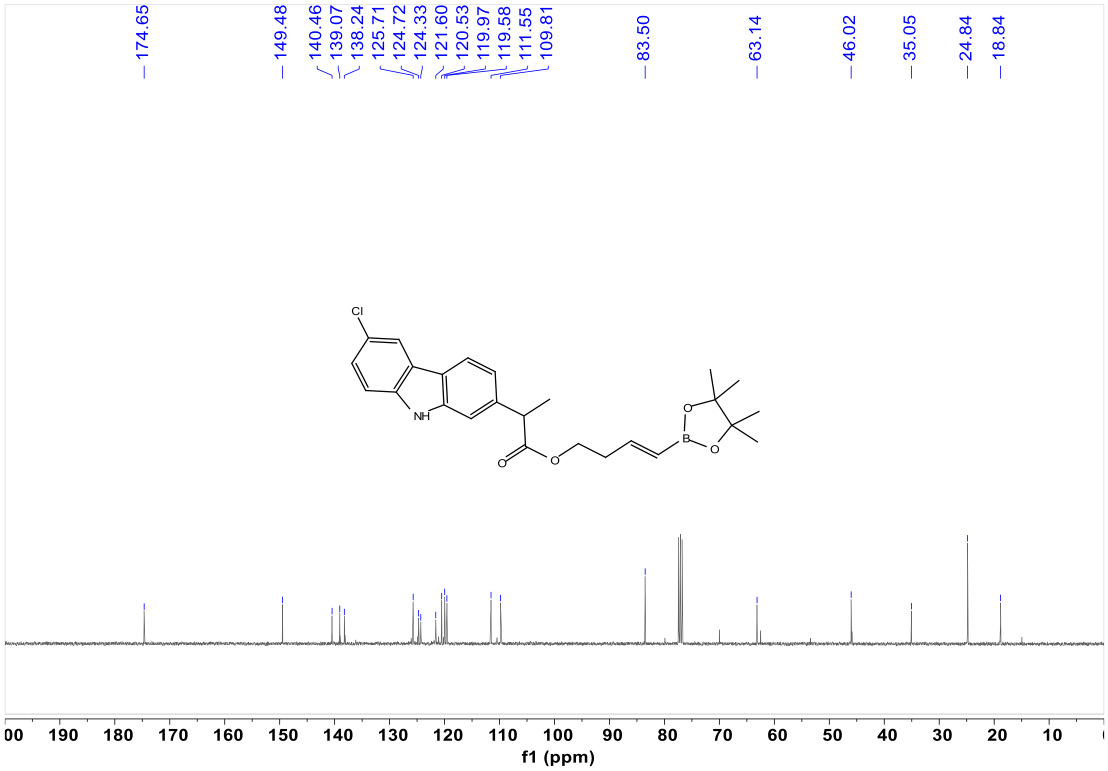

Supplementary Figure 30. ^{13}C NMR spectra for **S2**

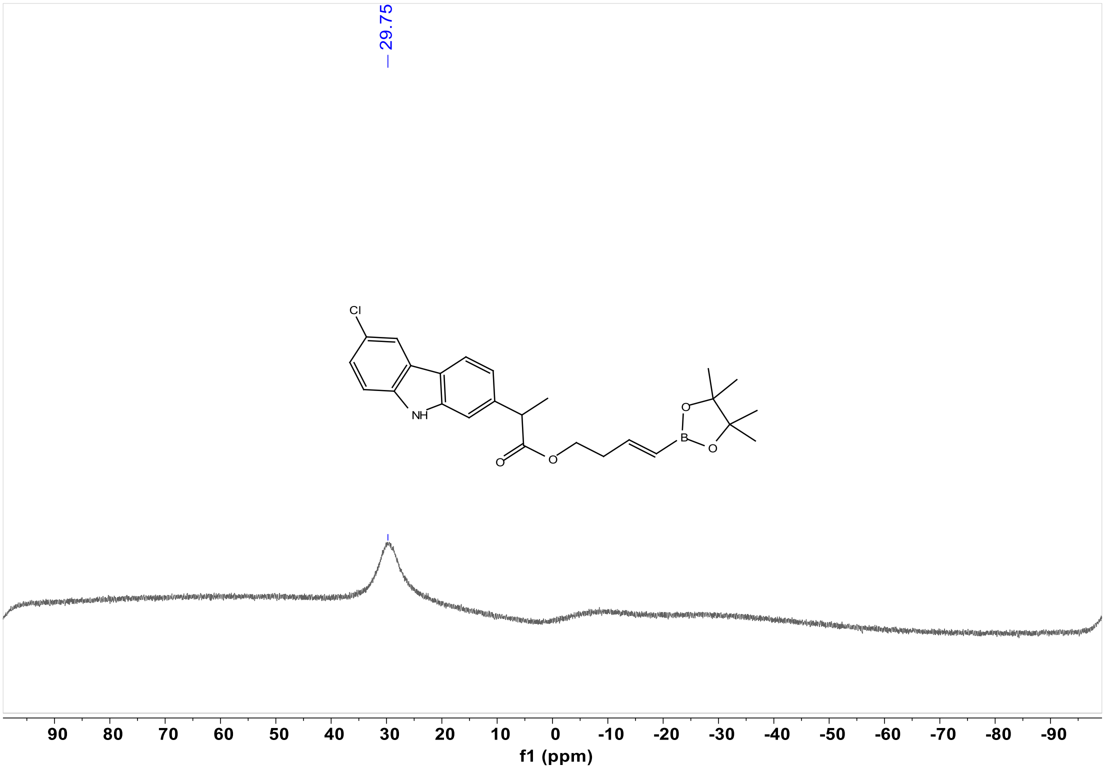

Supplementary Figure 31. ^{11}B NMR spectra for **S2**

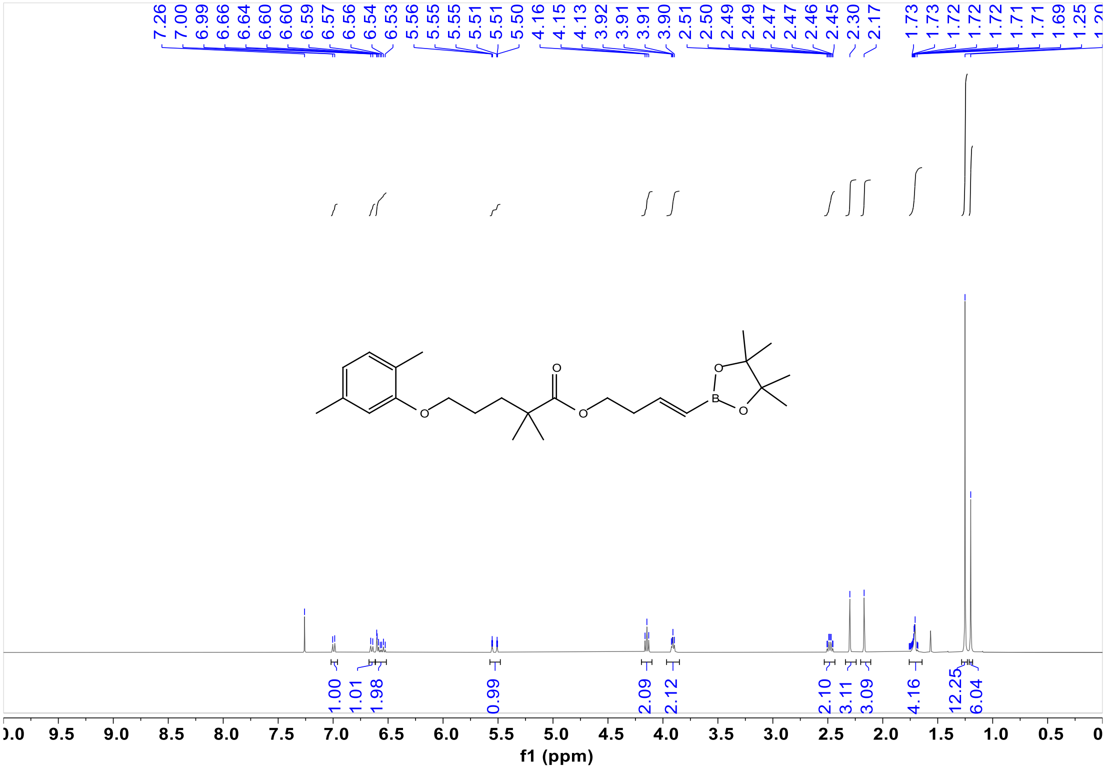

Supplementary Figure 32. ^{1}H NMR spectra for **S3**

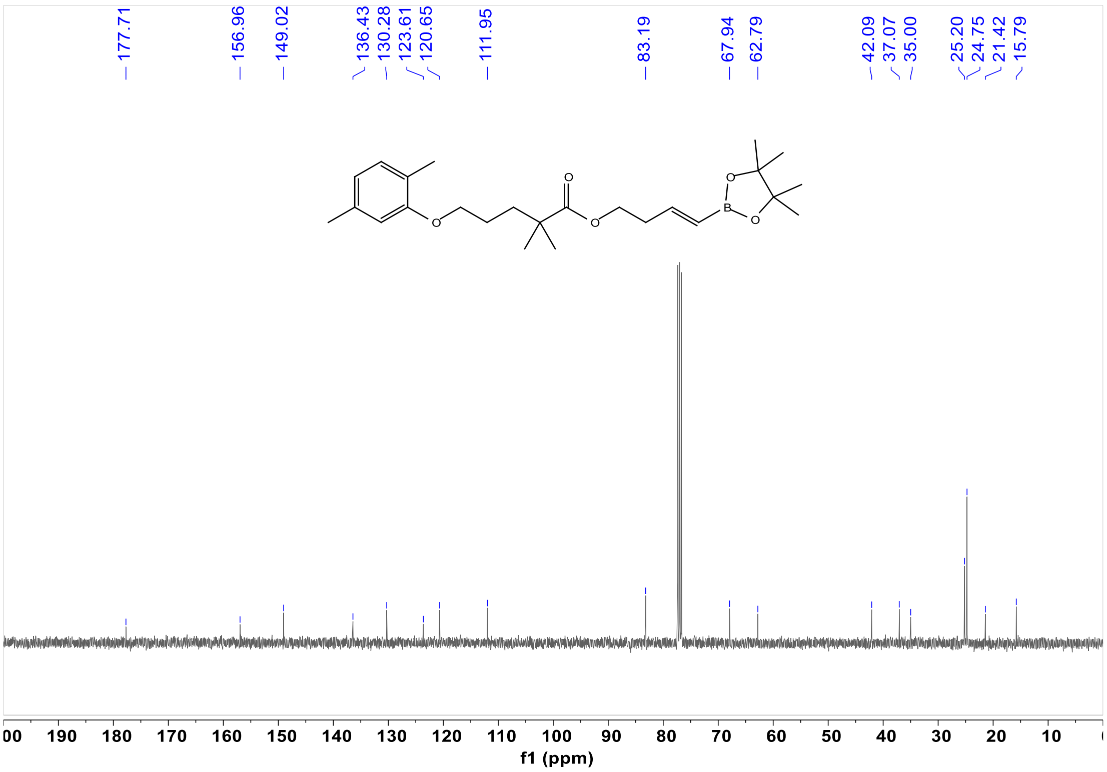

Supplementary Figure 33. ^{13}C NMR spectra for **S3**

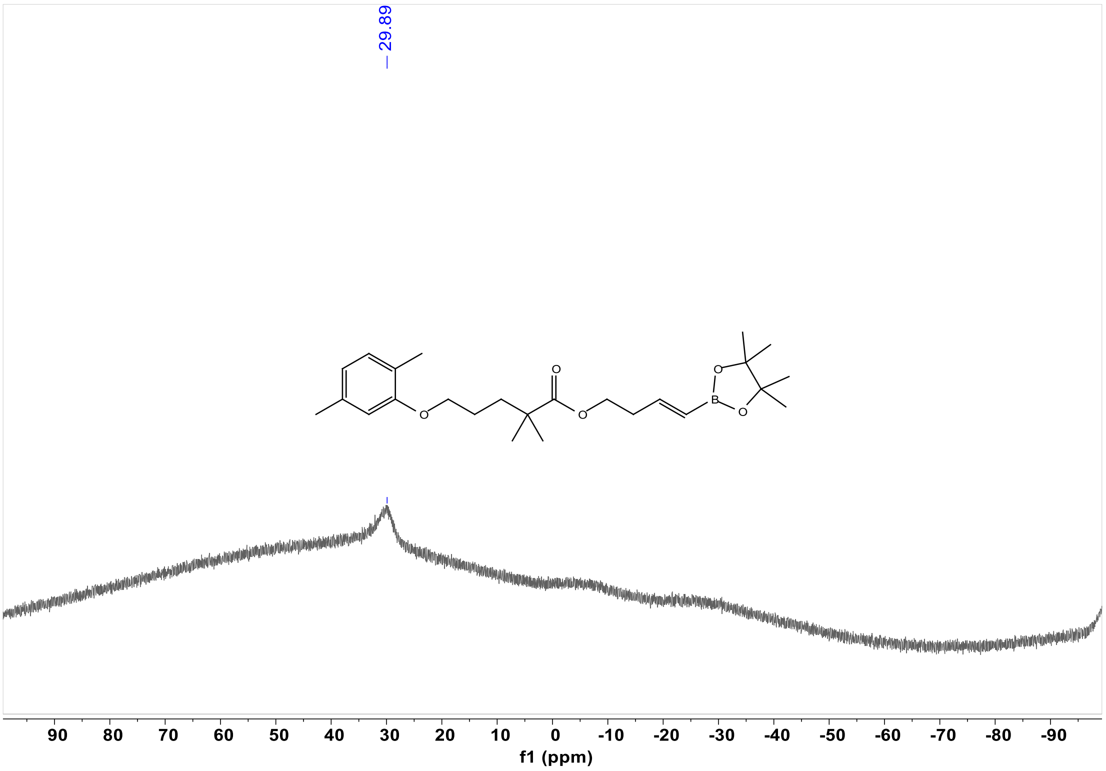

Supplementary Figure 34. ^{11}B NMR spectra for **S3**

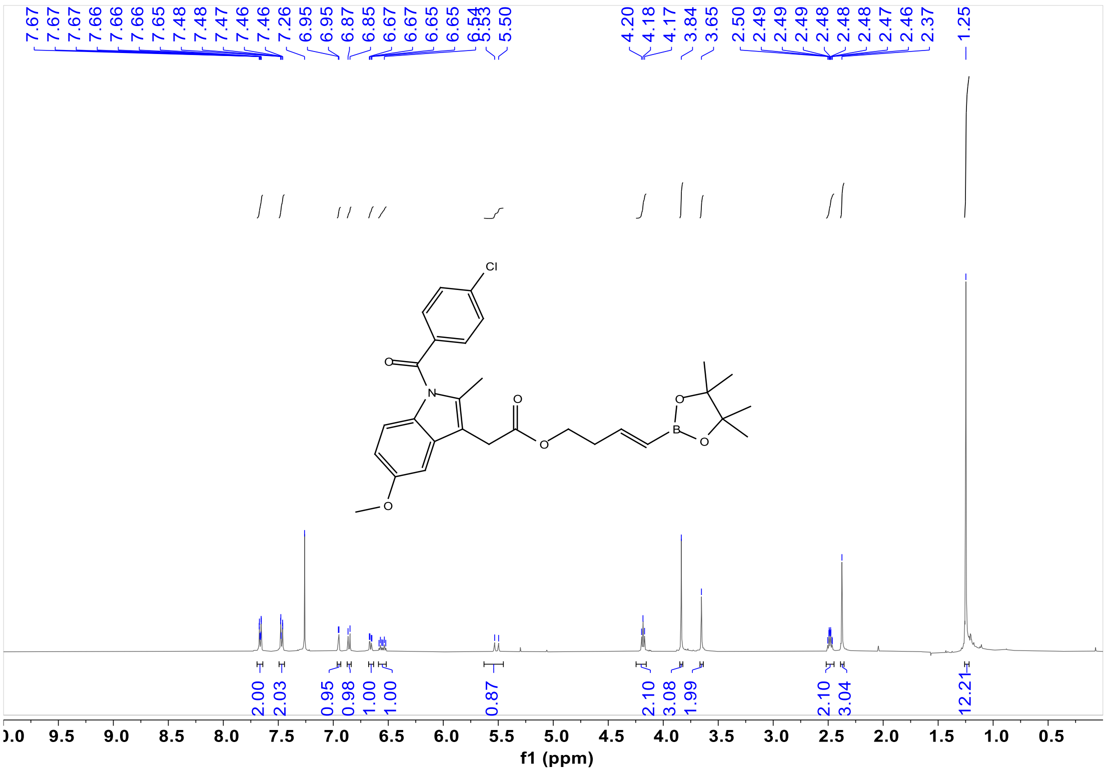

Supplementary Figure 35. ^{1}H NMR spectra for **S4**

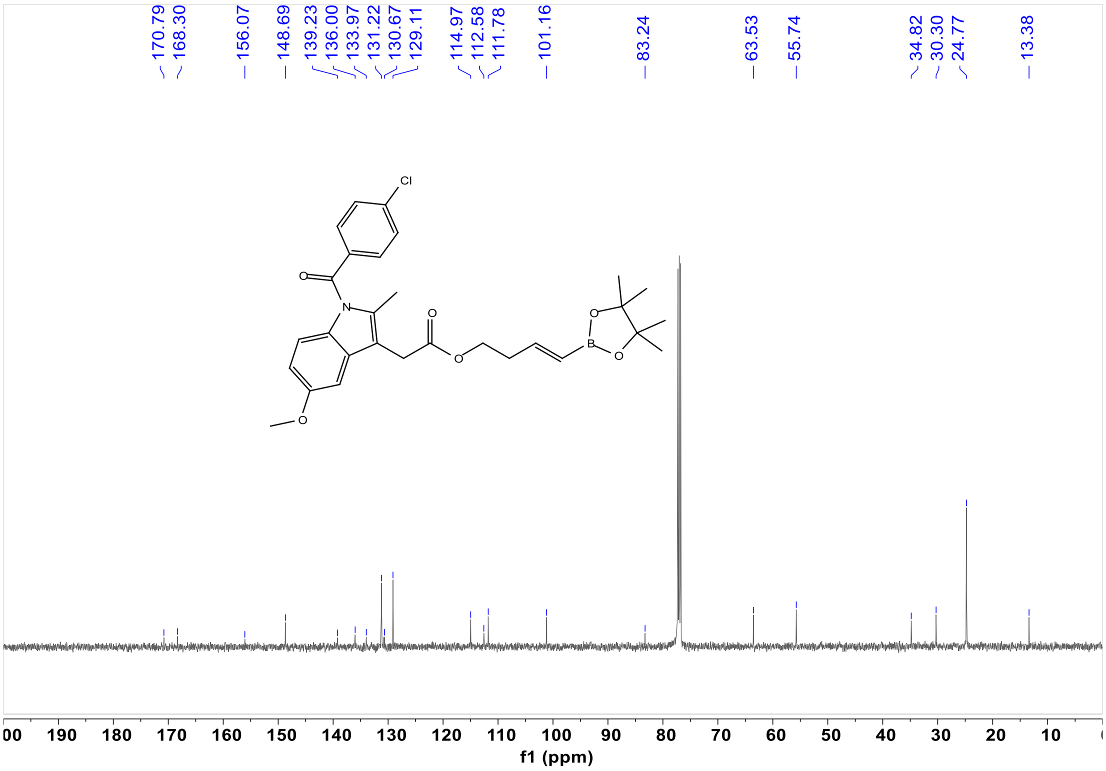

Supplementary Figure 36. ^{13}C NMR spectra for **S4**

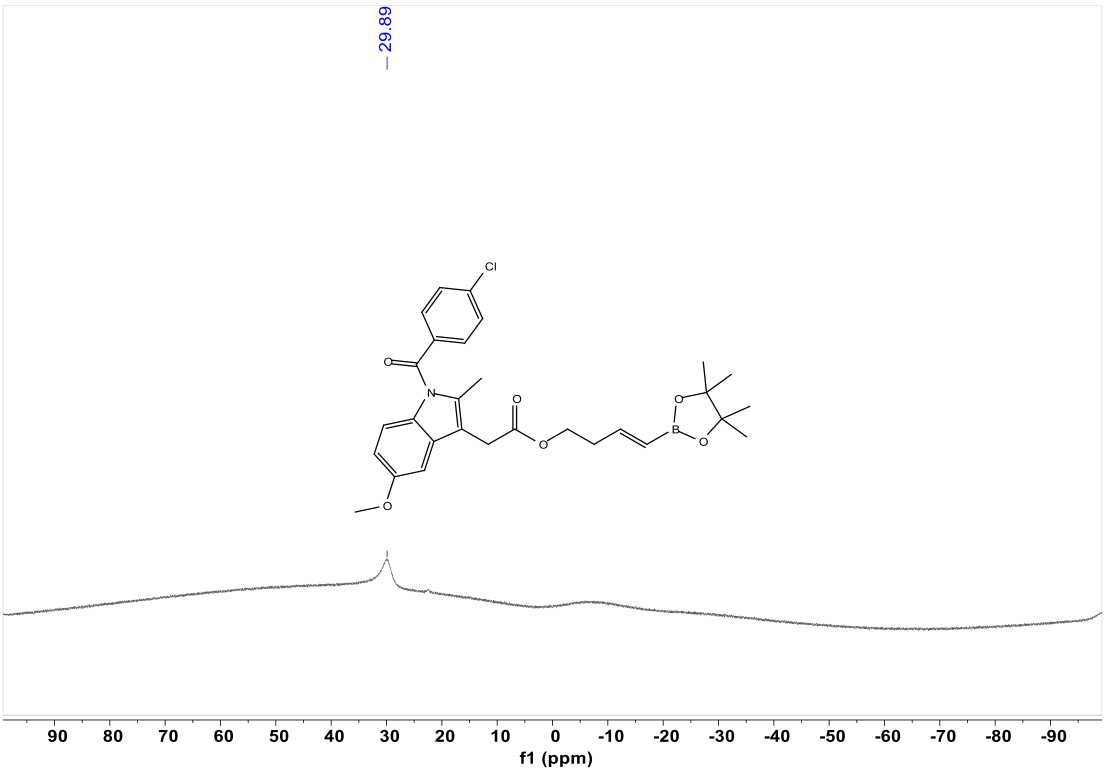

Supplementary Figure 37. ^{11}B NMR spectra for **S4**

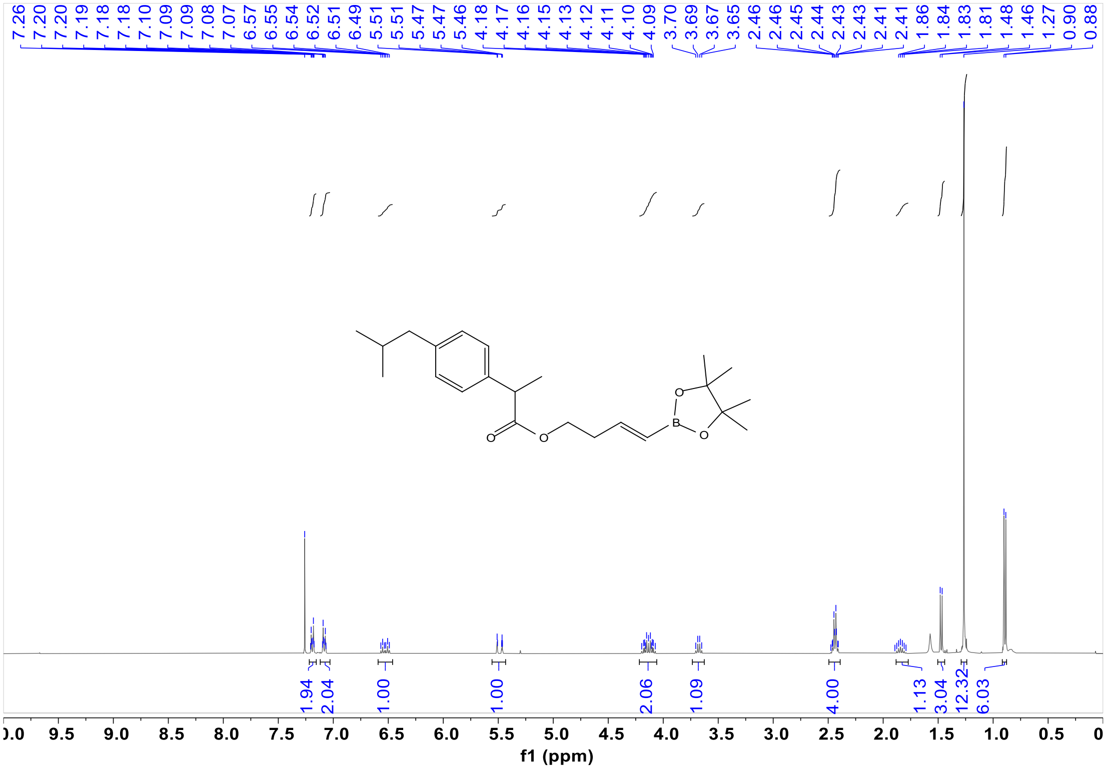

Supplementary Figure 38. ^{1}H NMR spectra for **S5**

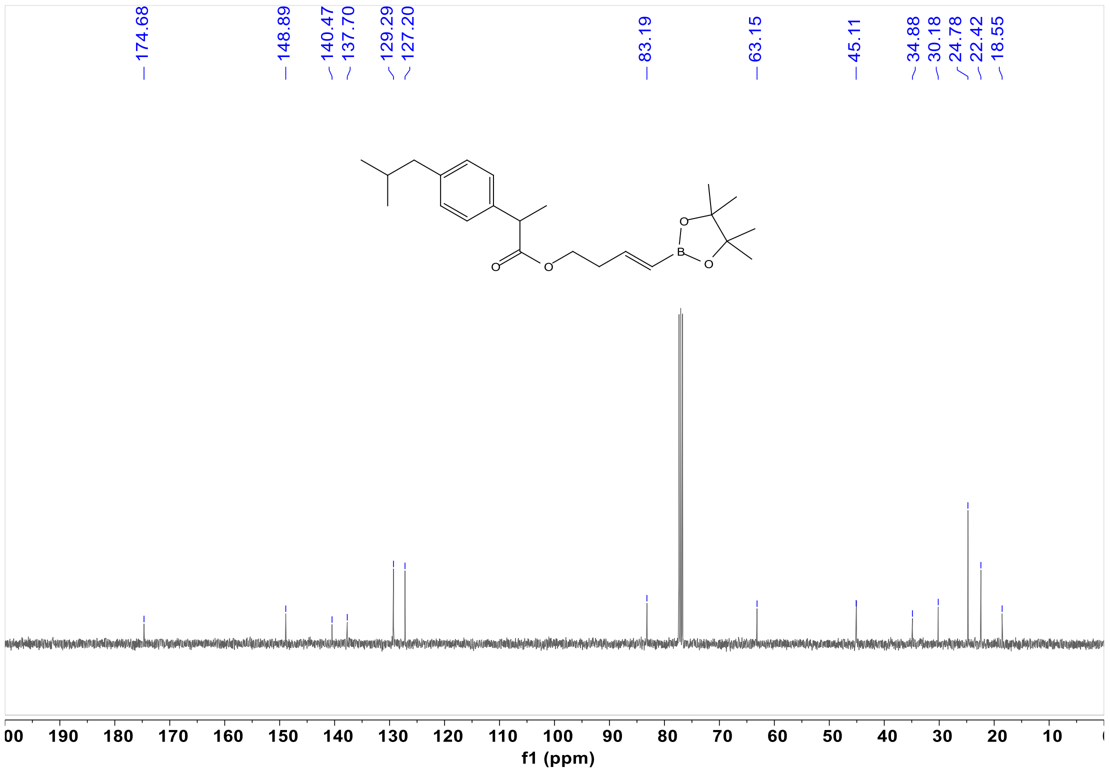

Supplementary Figure 39. ^{13}C NMR spectra for **S5**

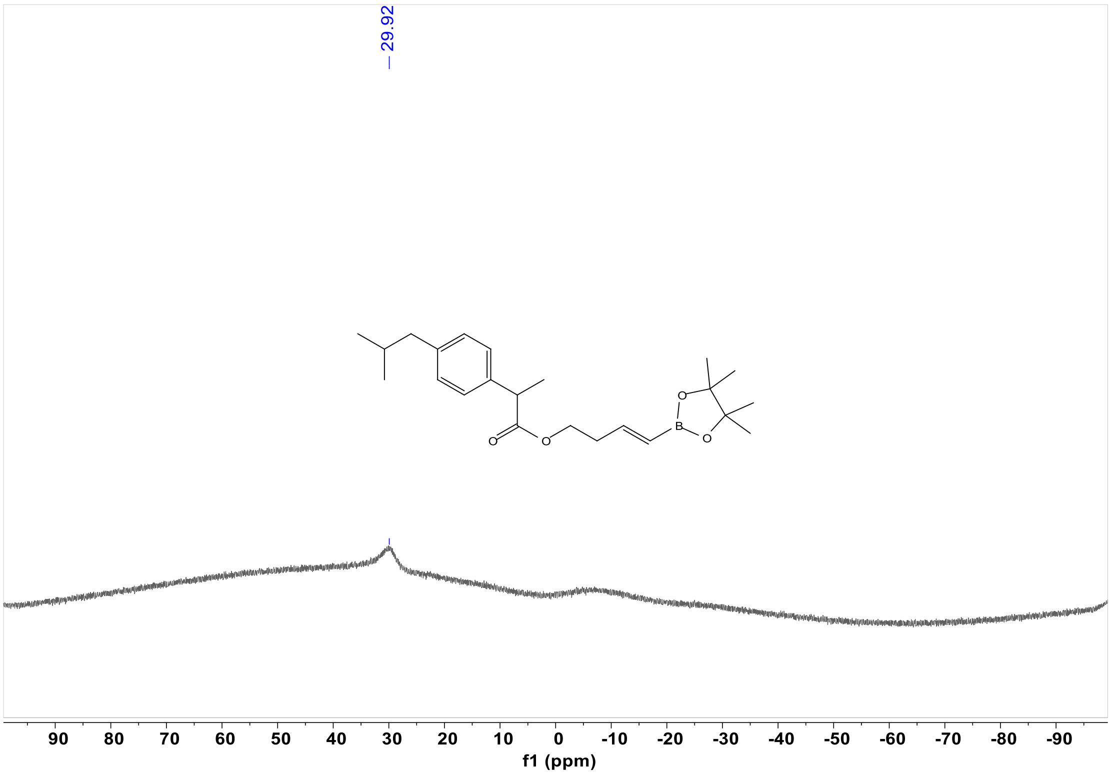

Supplementary Figure 40. ^{11}B NMR spectra for **S5**

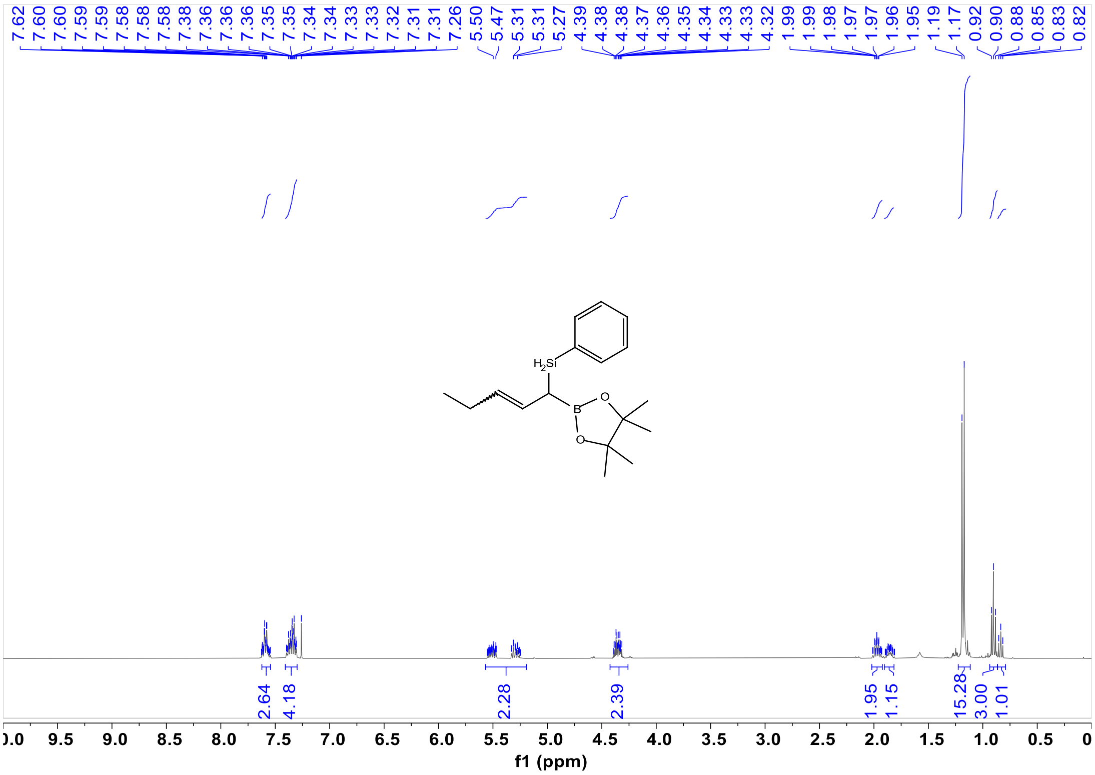

Supplementary Figure 41. ^{1}H NMR spectra for **S6**

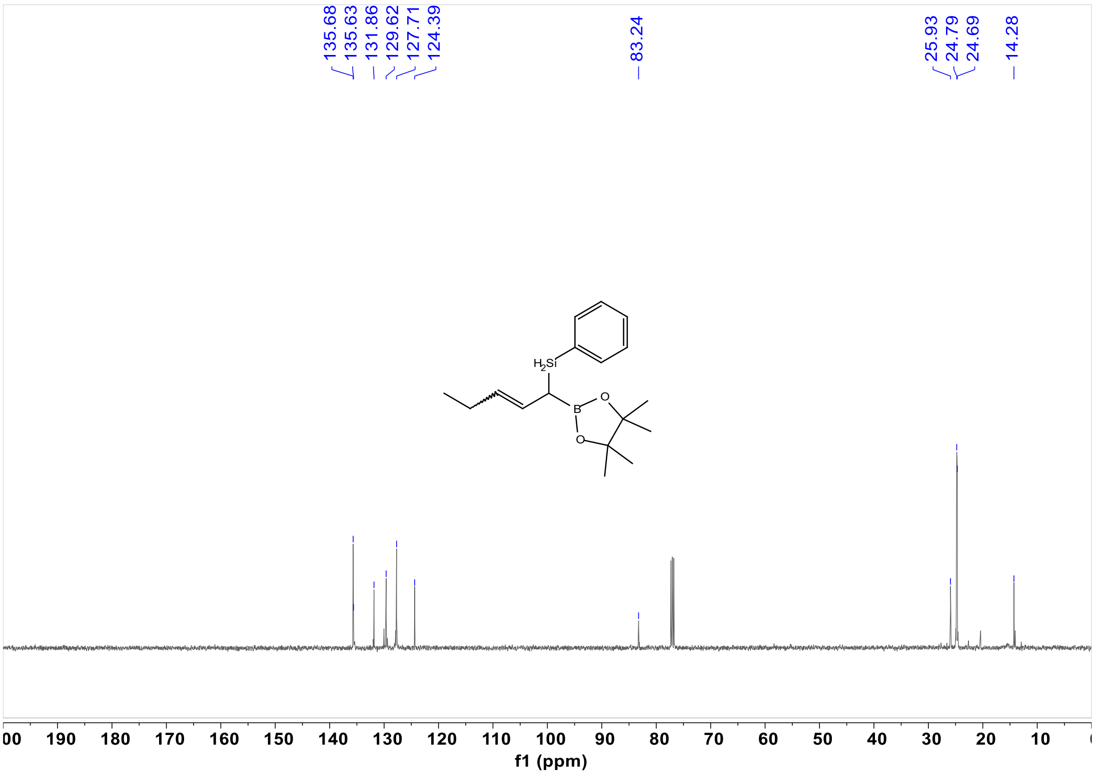

Supplementary Figure 42. ^{13}C NMR spectra for **S6**

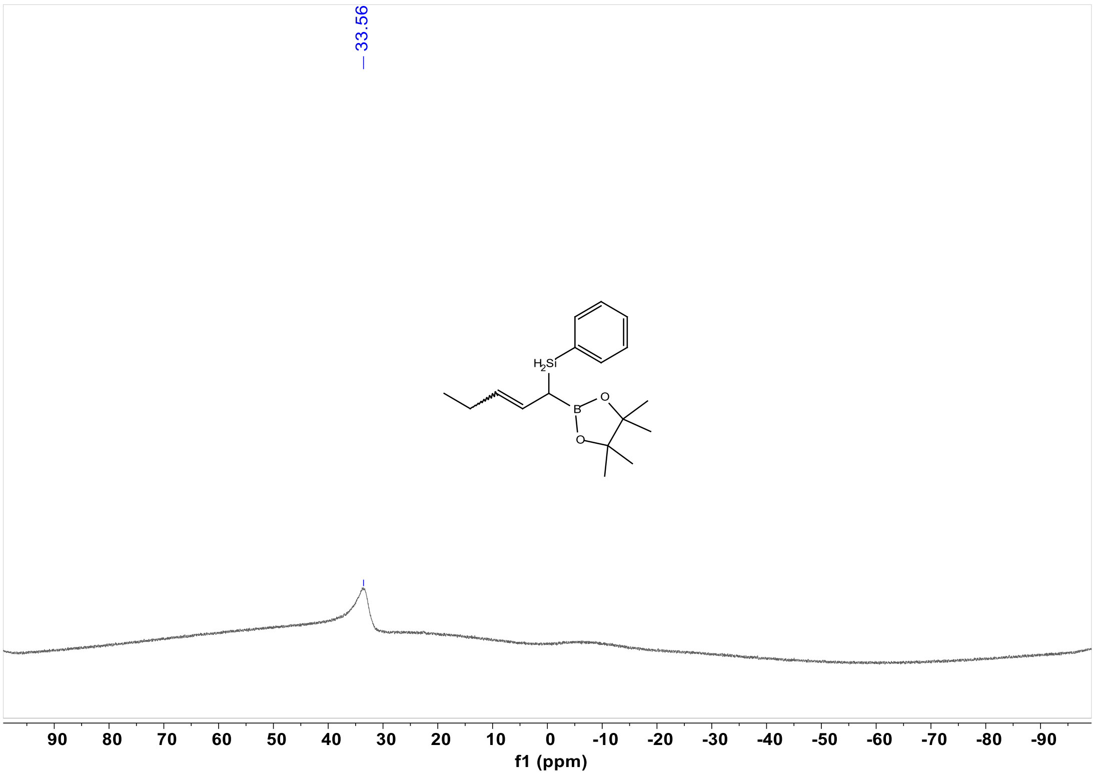

Supplementary Figure 43. ^{11}B NMR spectra for **S6**

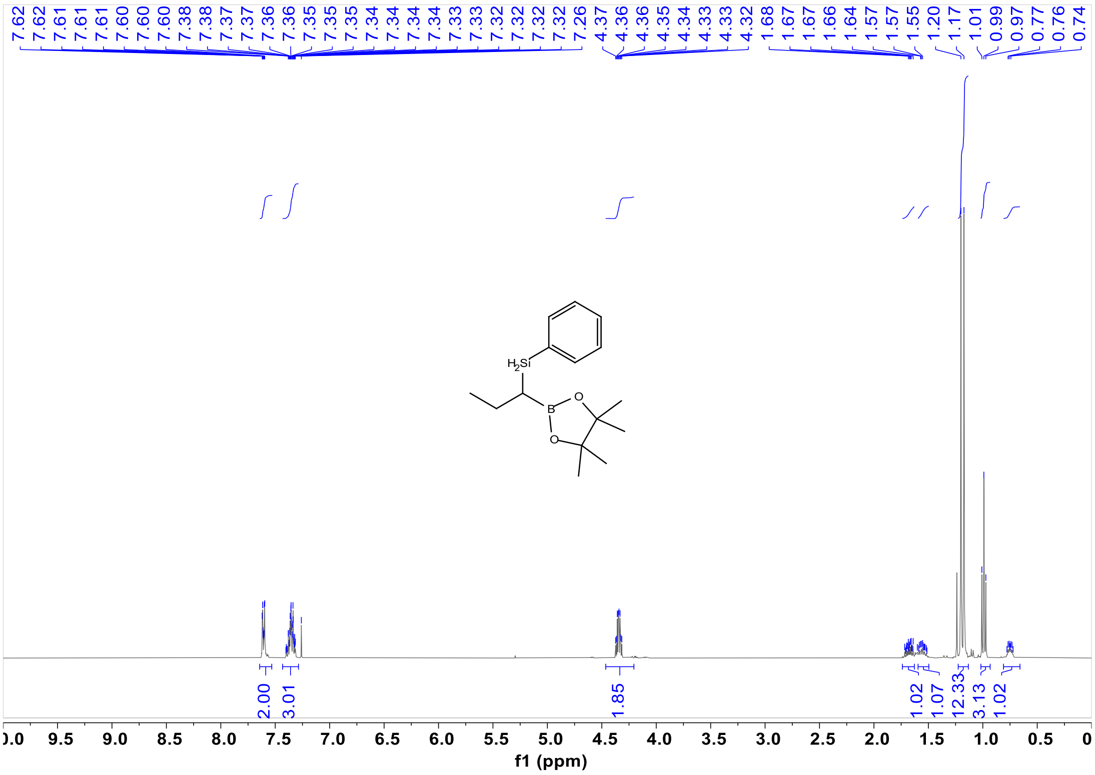

Supplementary Figure 44. ^{1}H NMR spectra for **11**

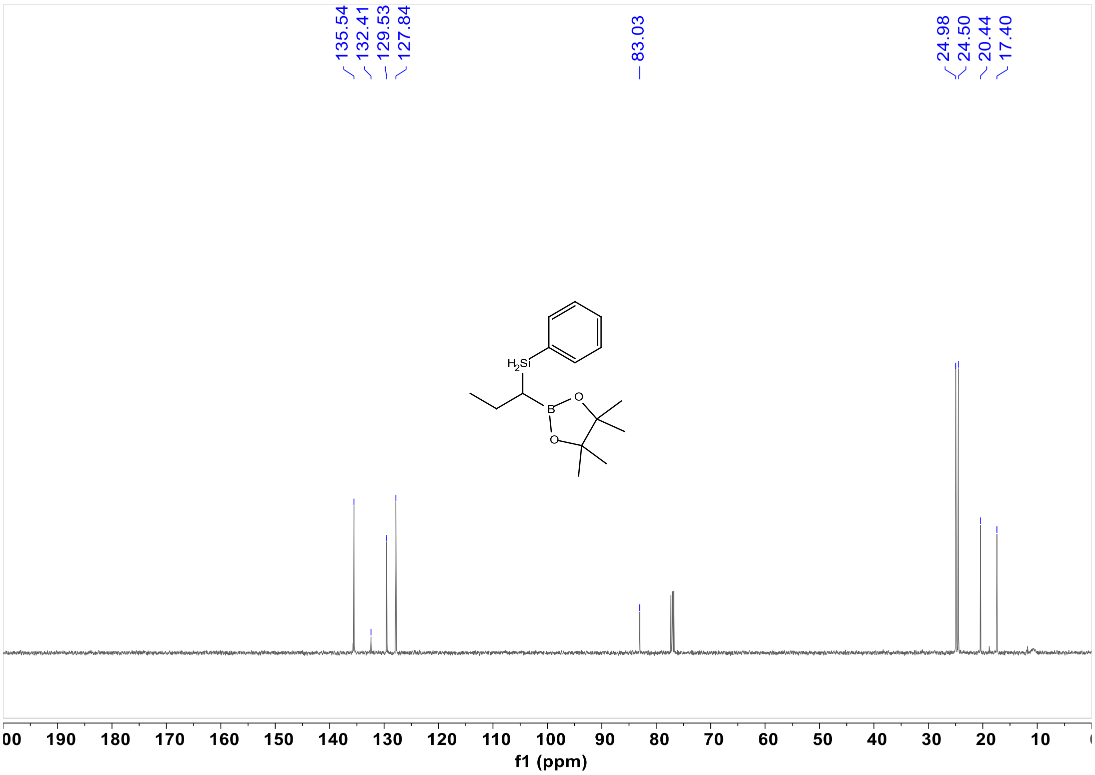

Supplementary Figure 45. ^{13}C NMR spectra for **11**

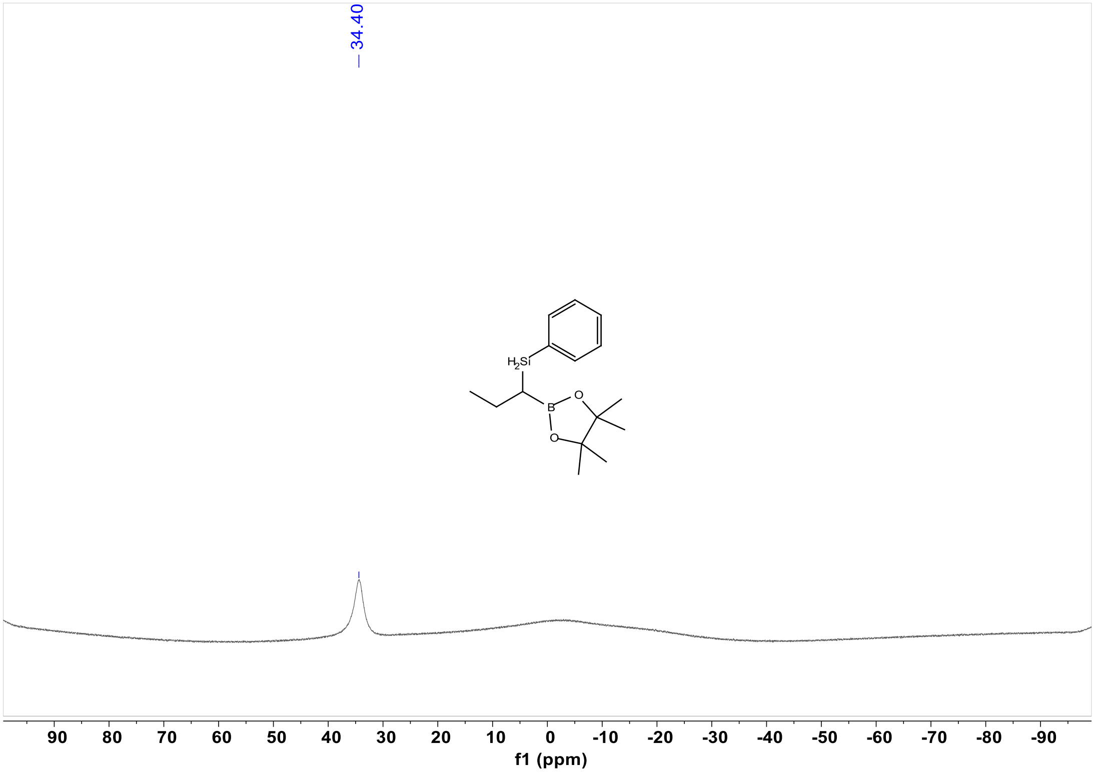

Supplementary Figure 46. ^{11}B NMR spectra for **11**

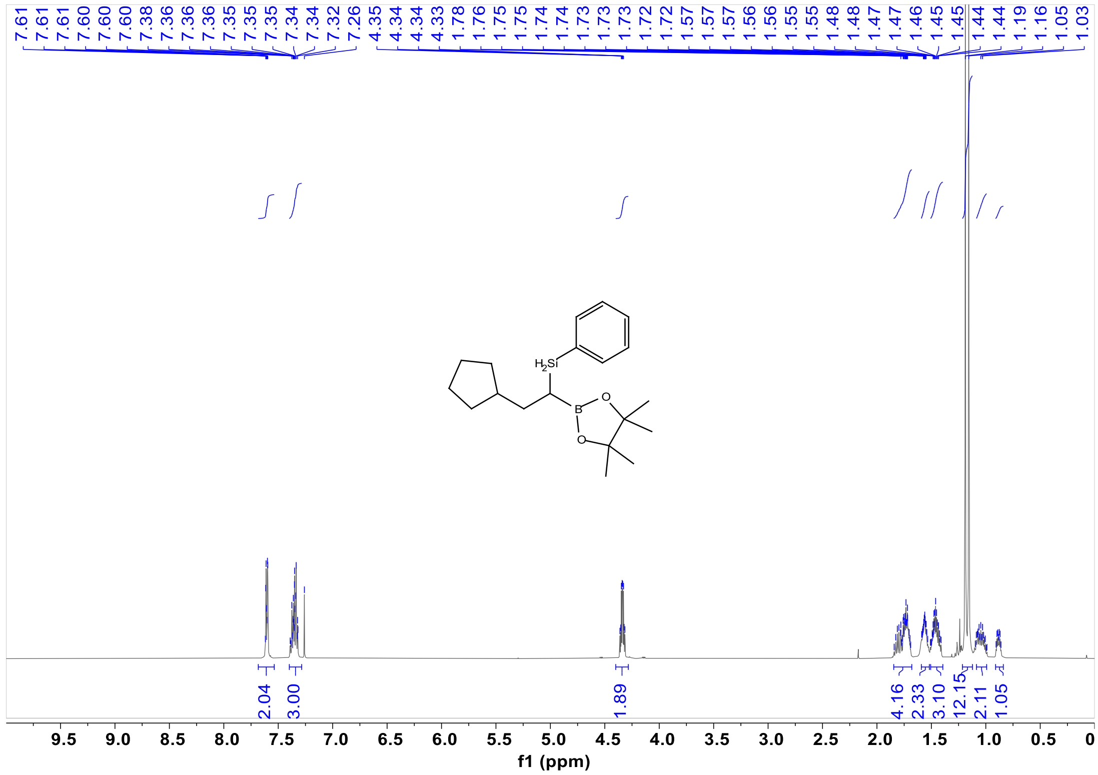

Supplementary Figure 47. ^{1}H NMR spectra for **12**

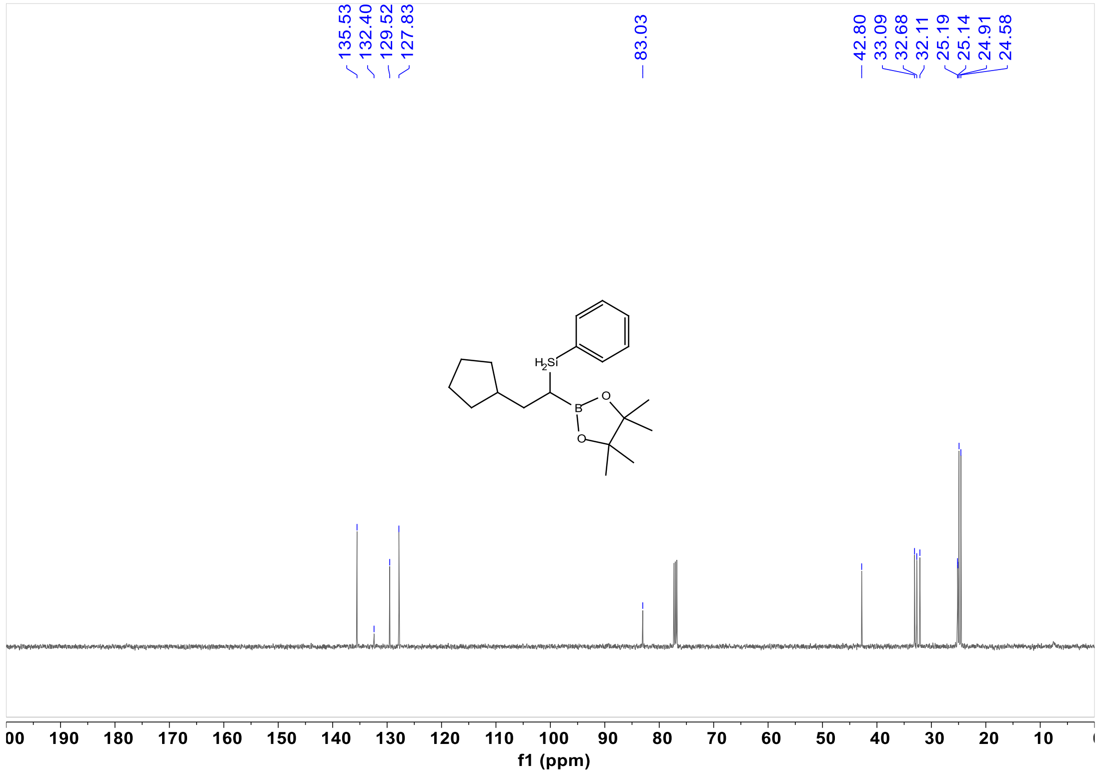

Supplementary Figure 48. ^{13}C NMR spectra for **12**

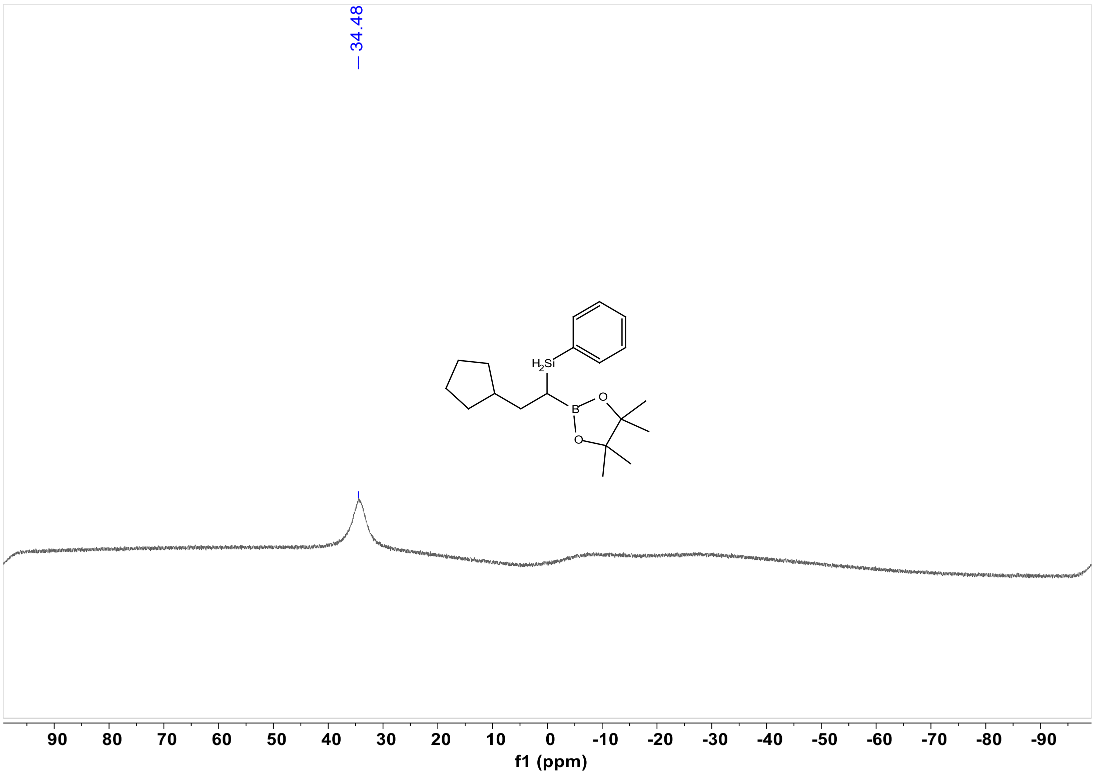

Supplementary Figure 49. ^{11}B NMR spectra for **12**

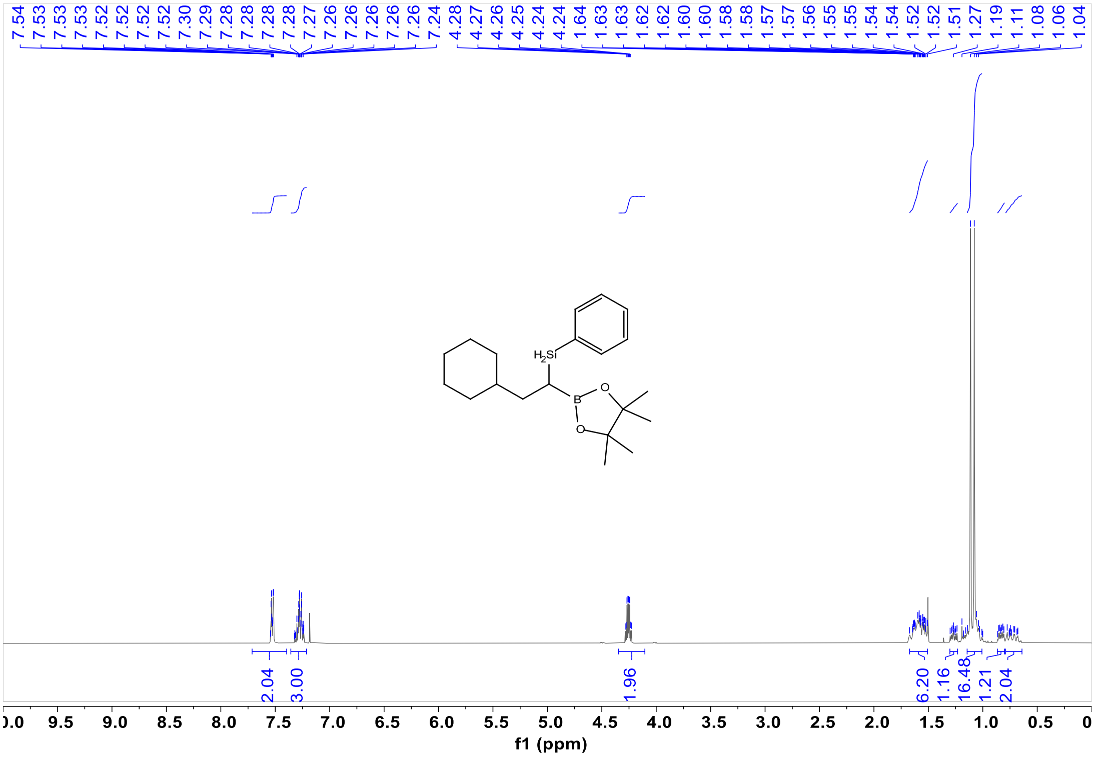

Supplementary Figure 50. ^{1}H NMR spectra for **13**

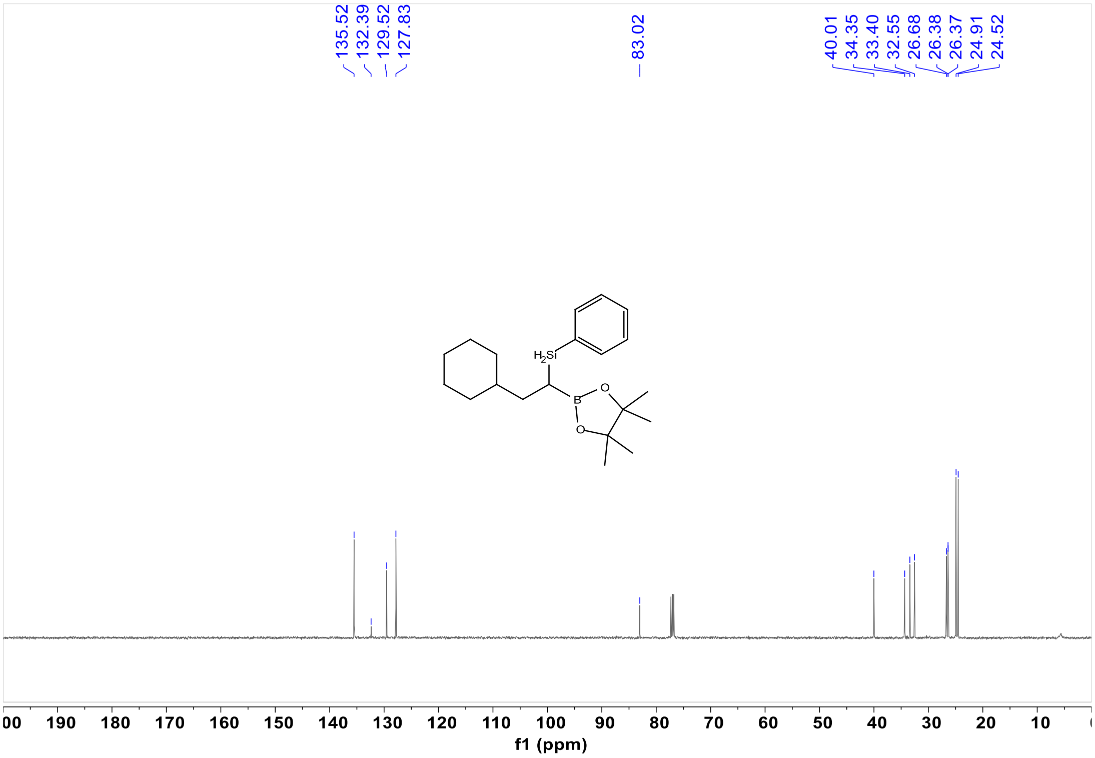

Supplementary Figure 51. ^{13}C NMR spectra for **13**

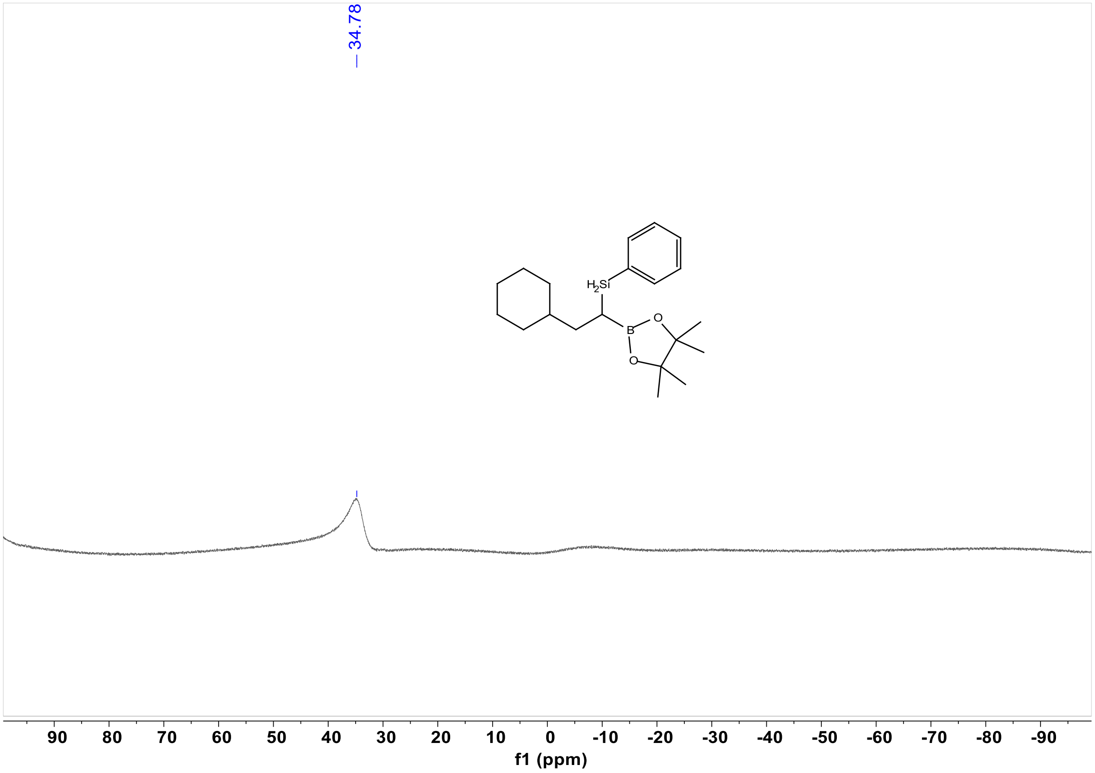

Supplementary Figure 52. ^{11}B NMR spectra for **13**

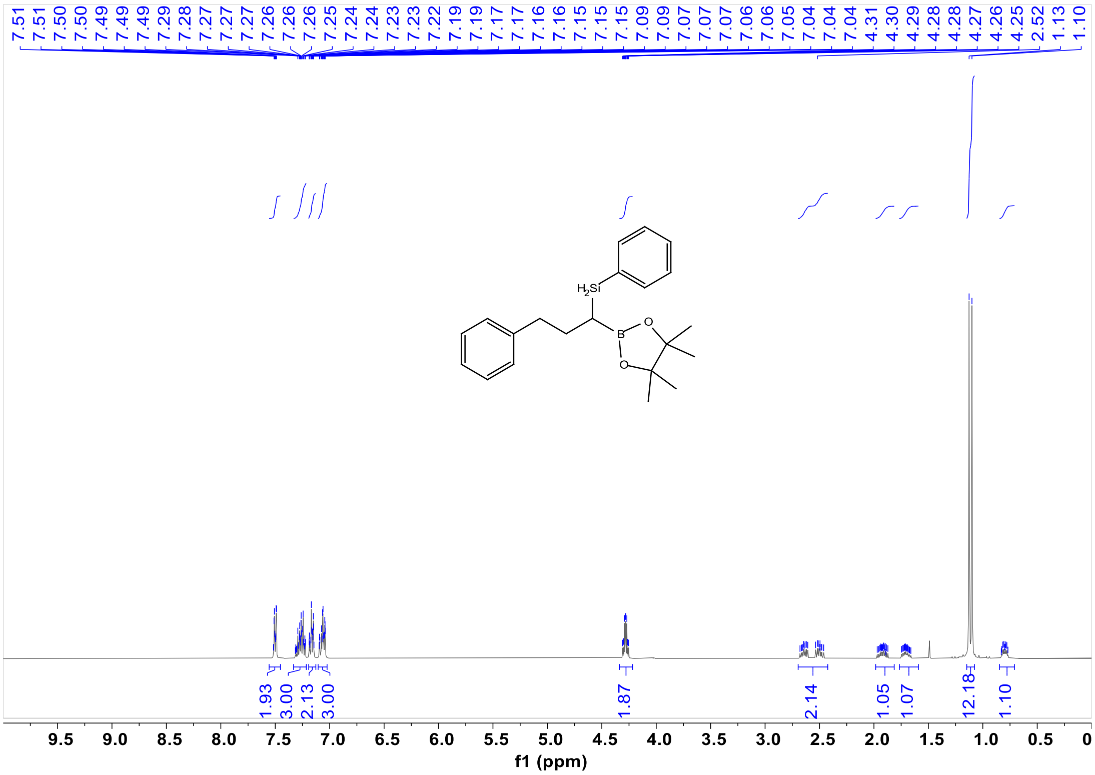

Supplementary Figure 53. ^{1}H NMR spectra for **14**

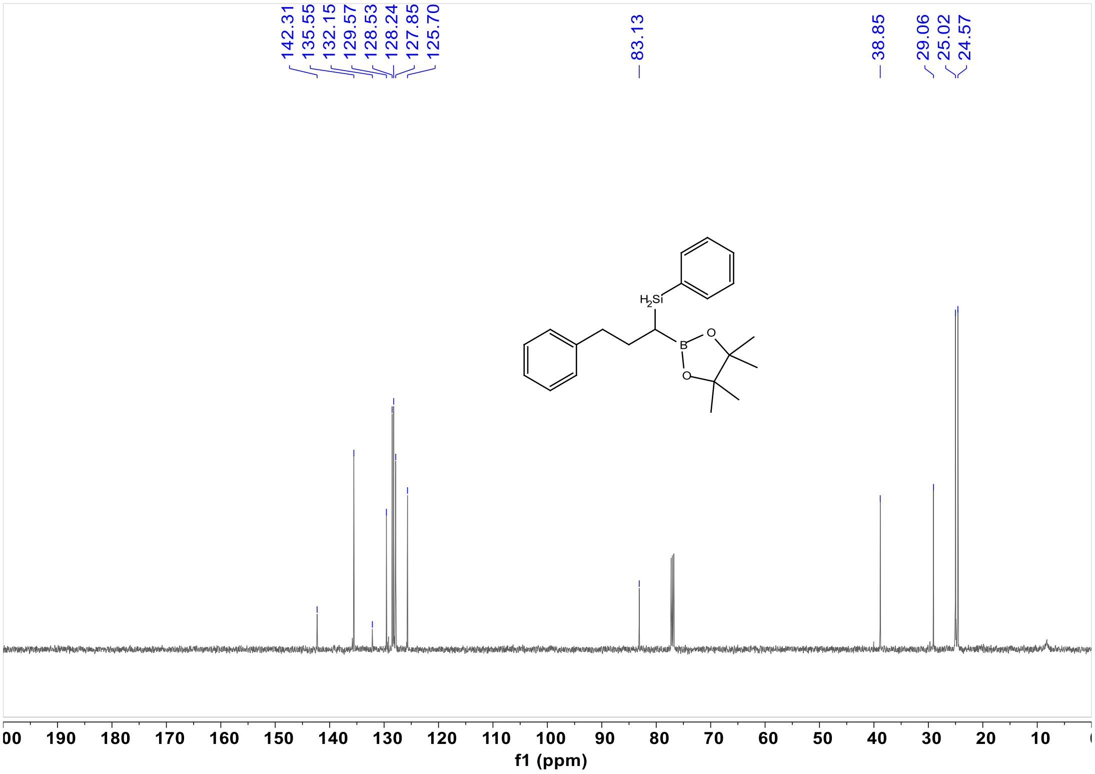

Supplementary Figure 54. ^{13}C NMR spectra for **14**

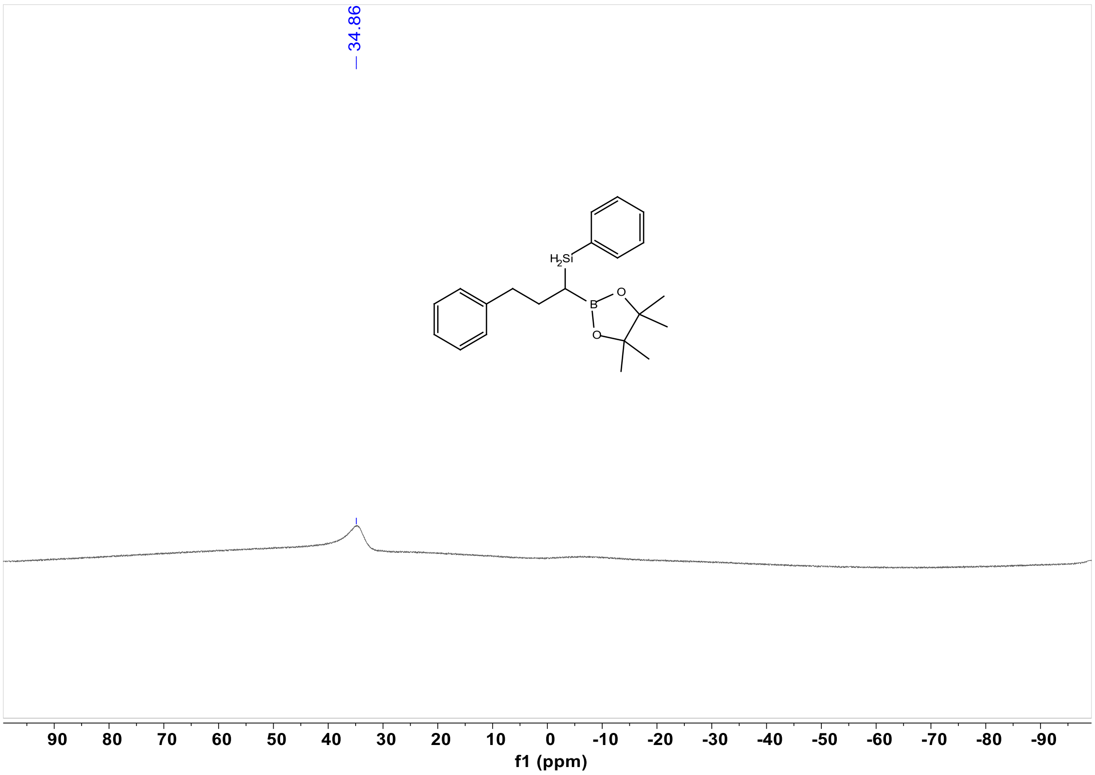

Supplementary Figure 55. ^{11}B NMR spectra for **14**

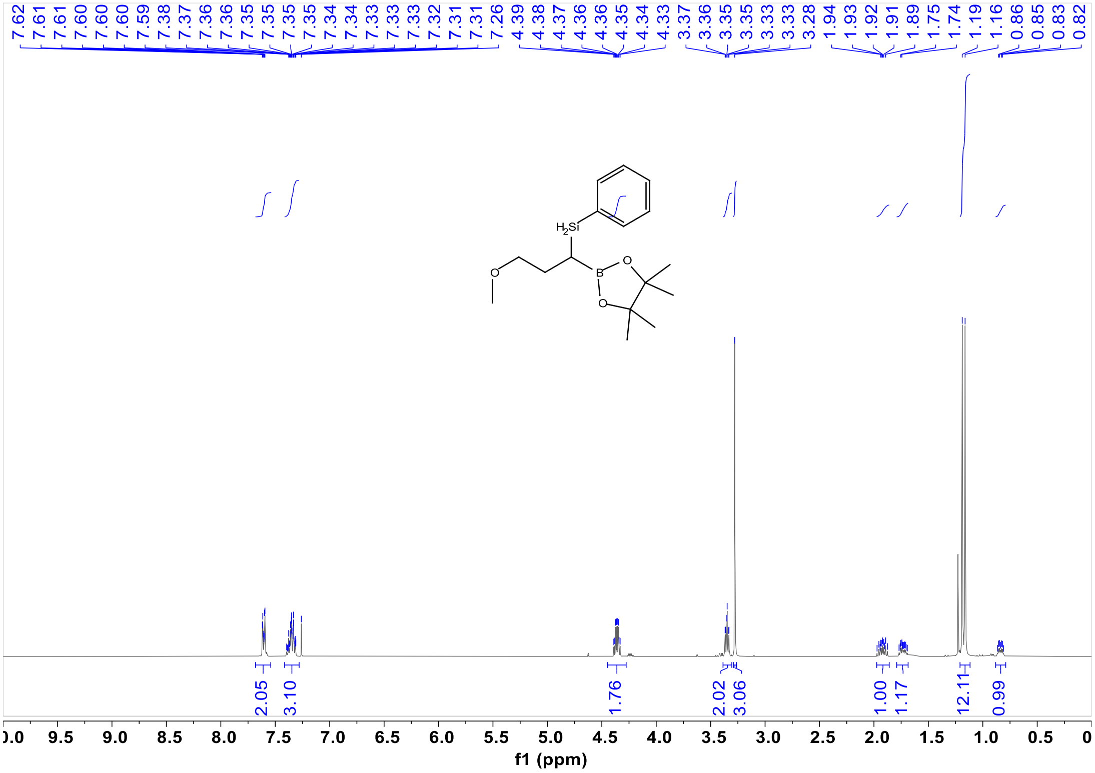

Supplementary Figure 56. ^{1}H NMR spectra for **15**

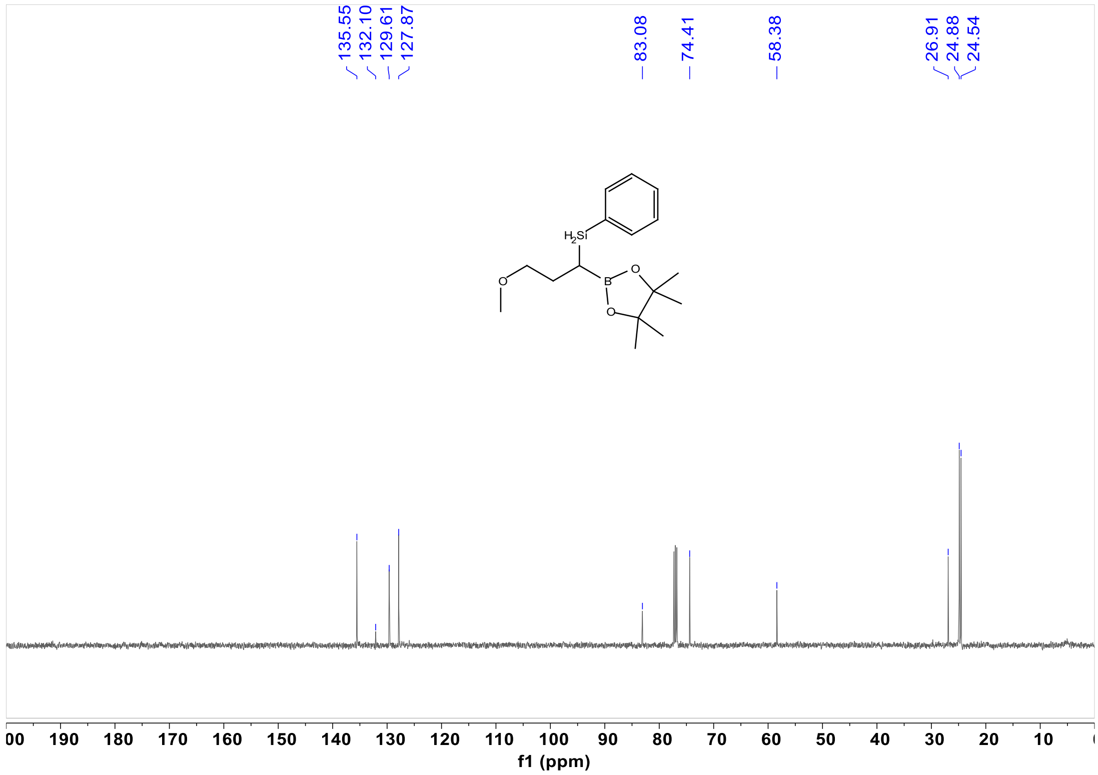

Supplementary Figure 57. ^{13}C NMR spectra for **15**

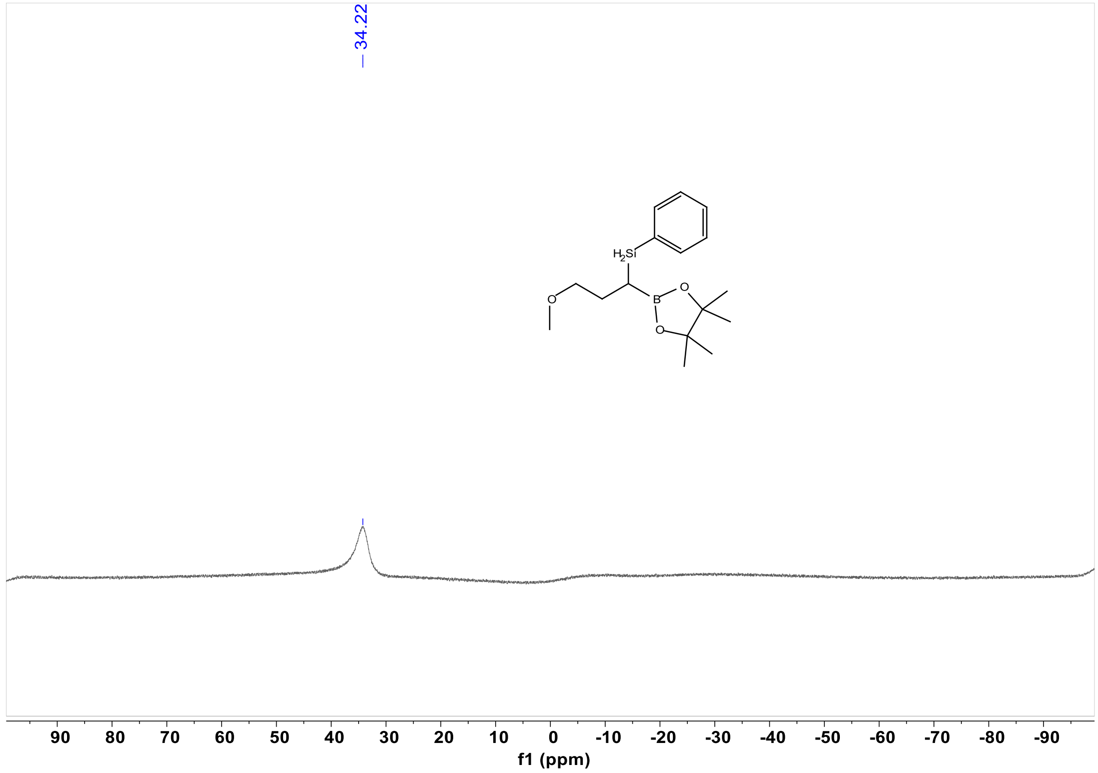

Supplementary Figure 58. ^{11}B NMR spectra for **15**

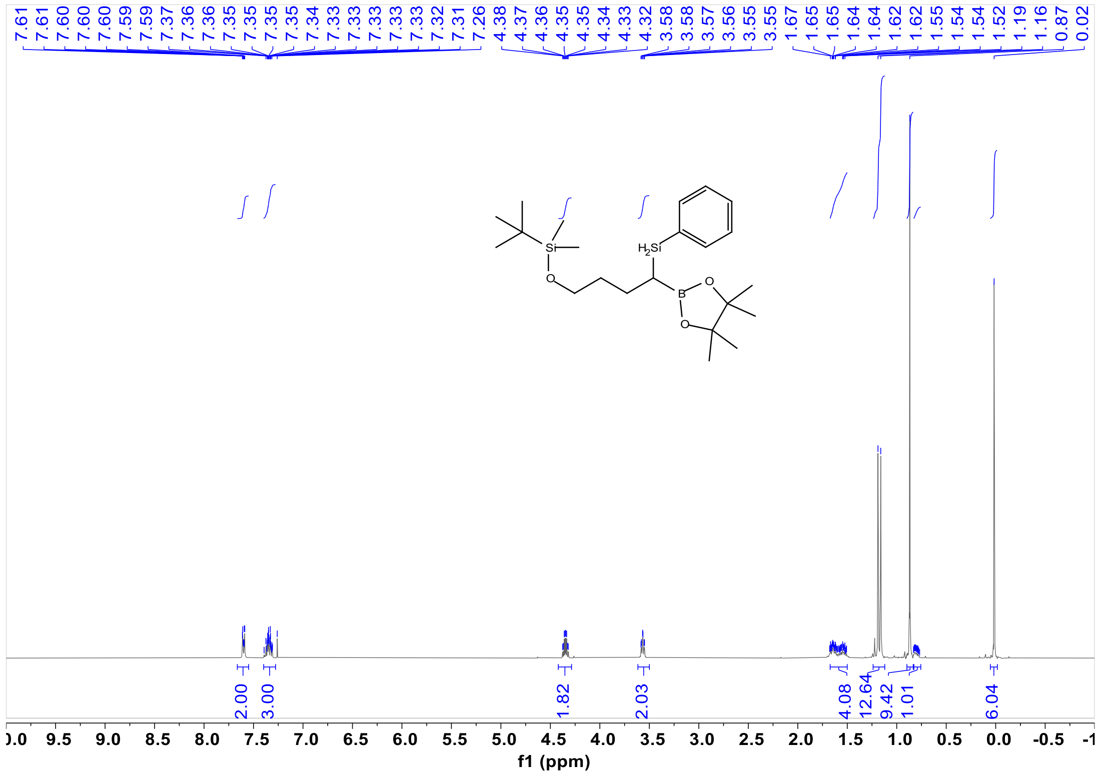

Supplementary Figure 59. ^{1}H NMR spectra for **16**

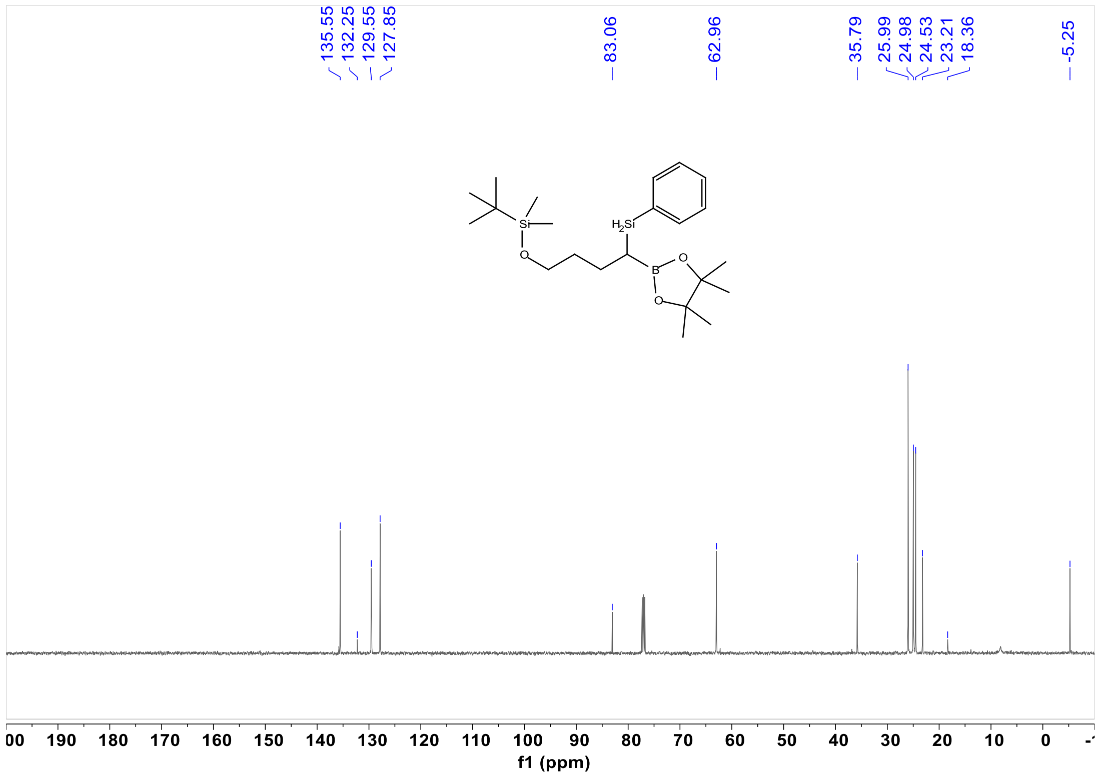

Supplementary Figure 60. ^{13}C NMR spectra for **16**

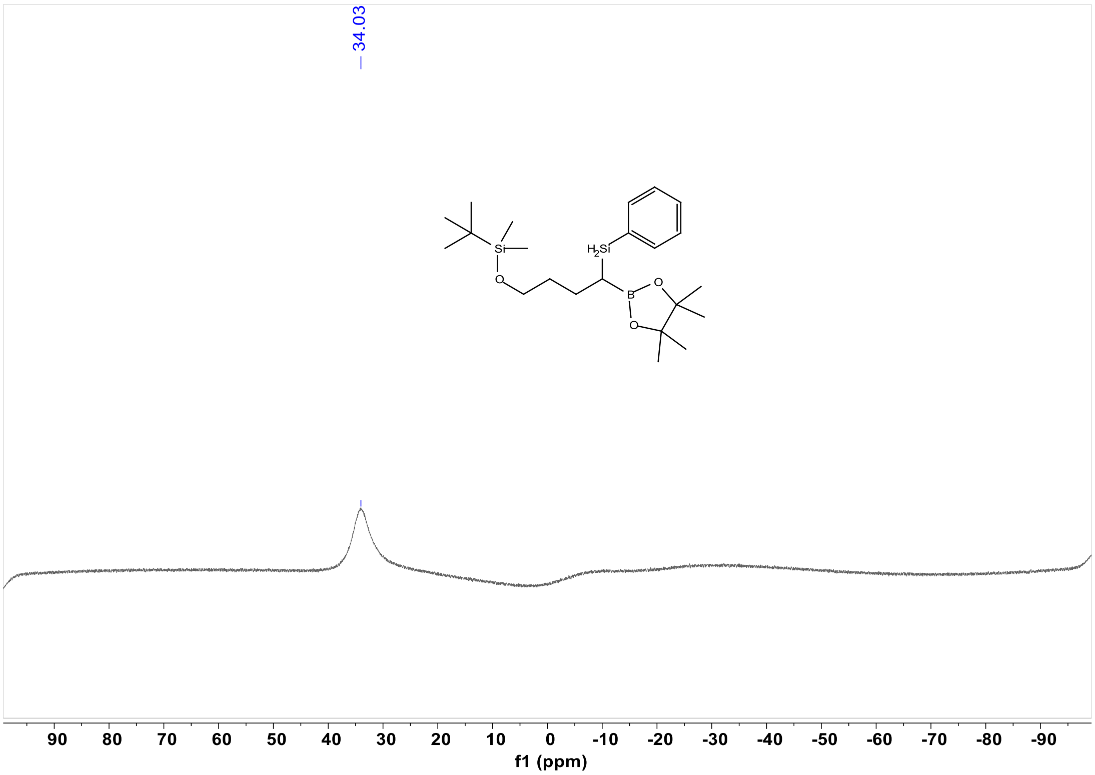

Supplementary Figure 61. ^{11}B NMR spectra for **16**

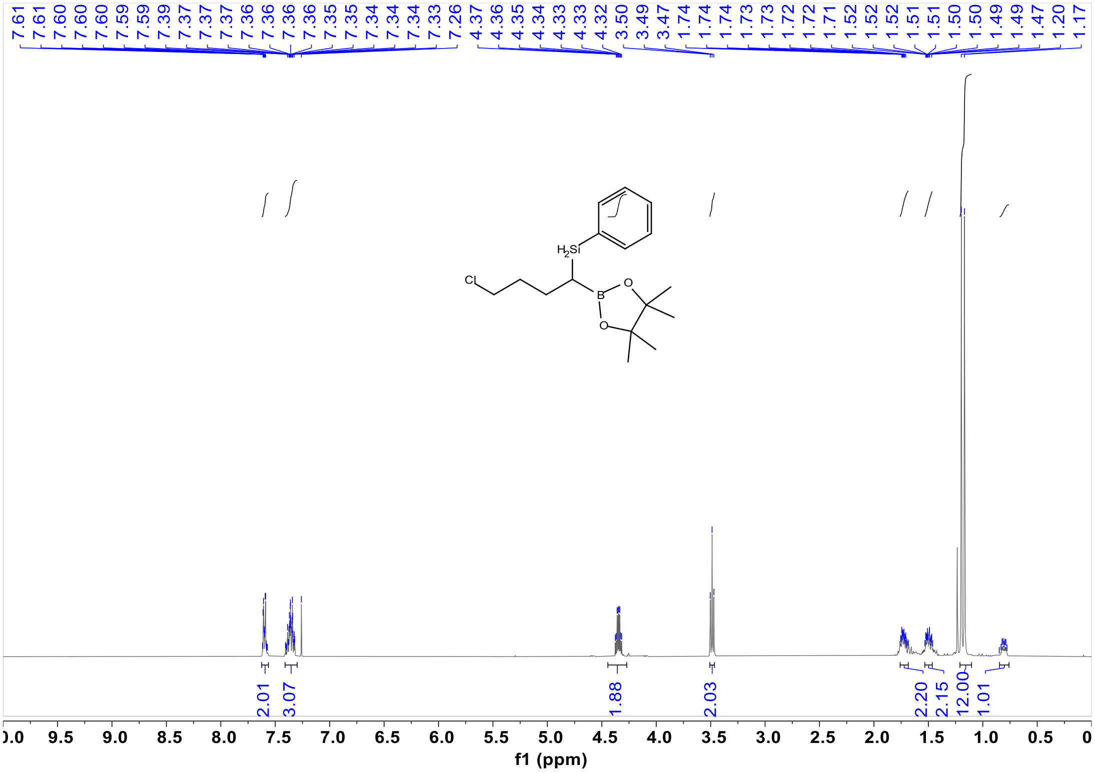

Supplementary Figure 62. ^{1}H NMR spectra for **17**

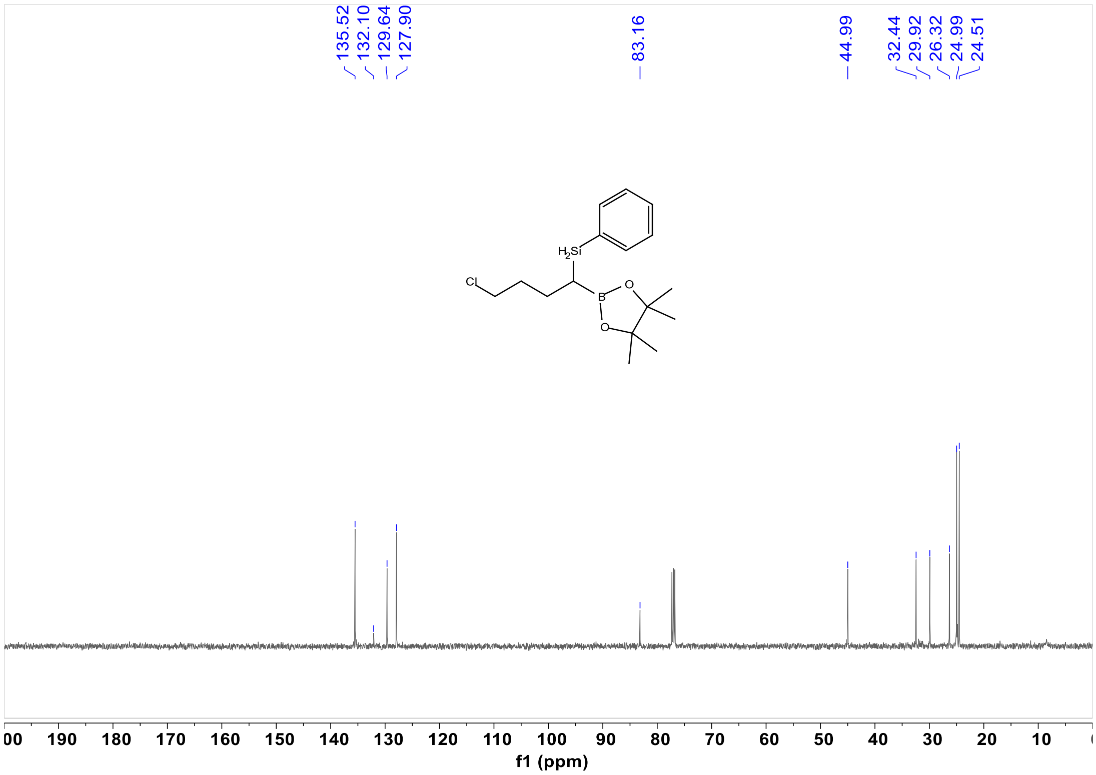

Supplementary Figure 63. ^{13}C NMR spectra for **17**

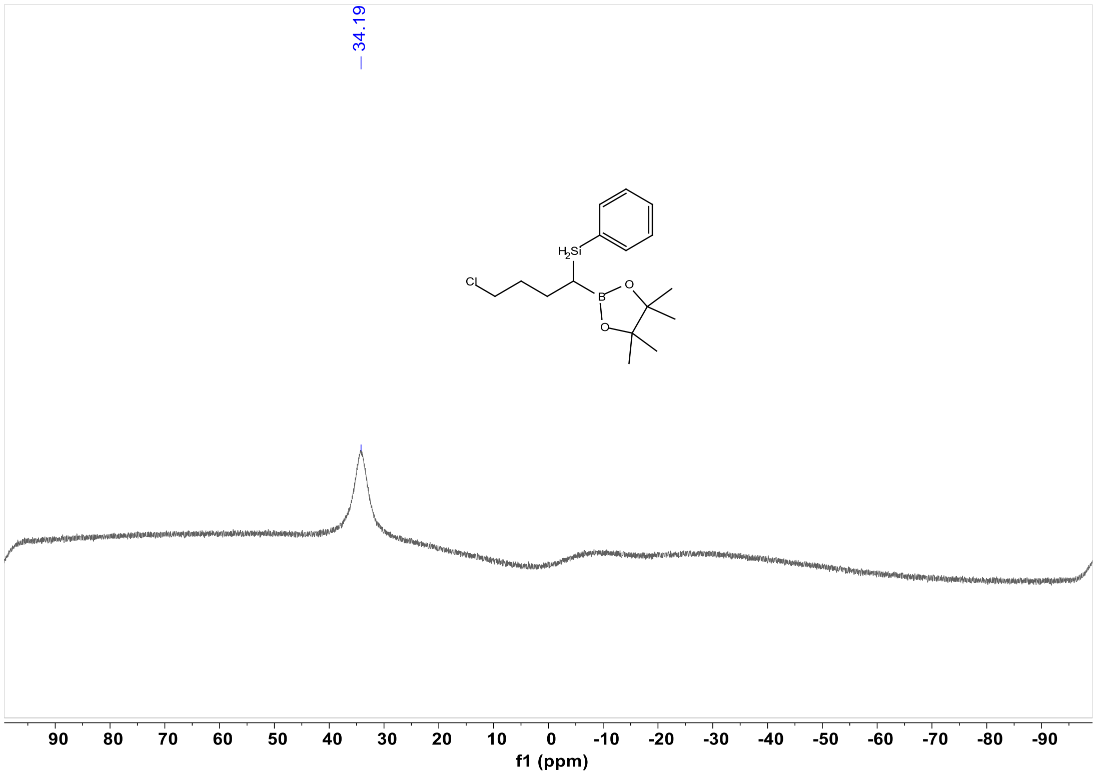

Supplementary Figure 64. ^{11}B NMR spectra for **17**

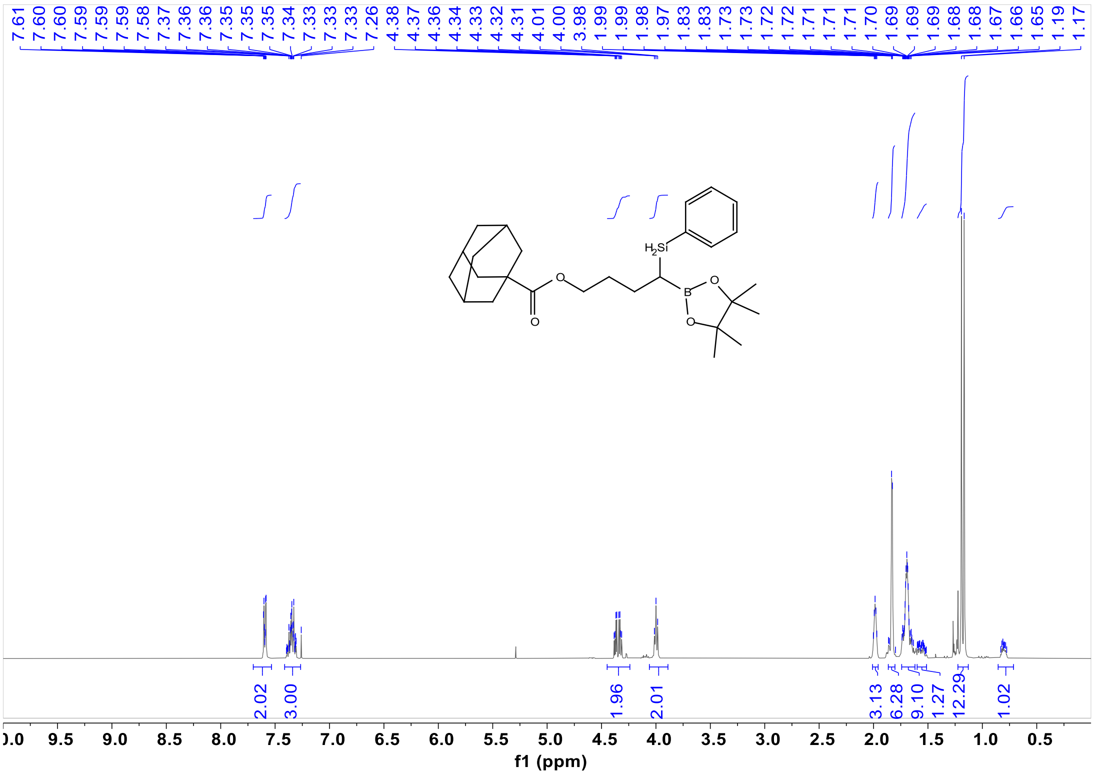

Supplementary Figure 65. ^{1}H NMR spectra for **18**

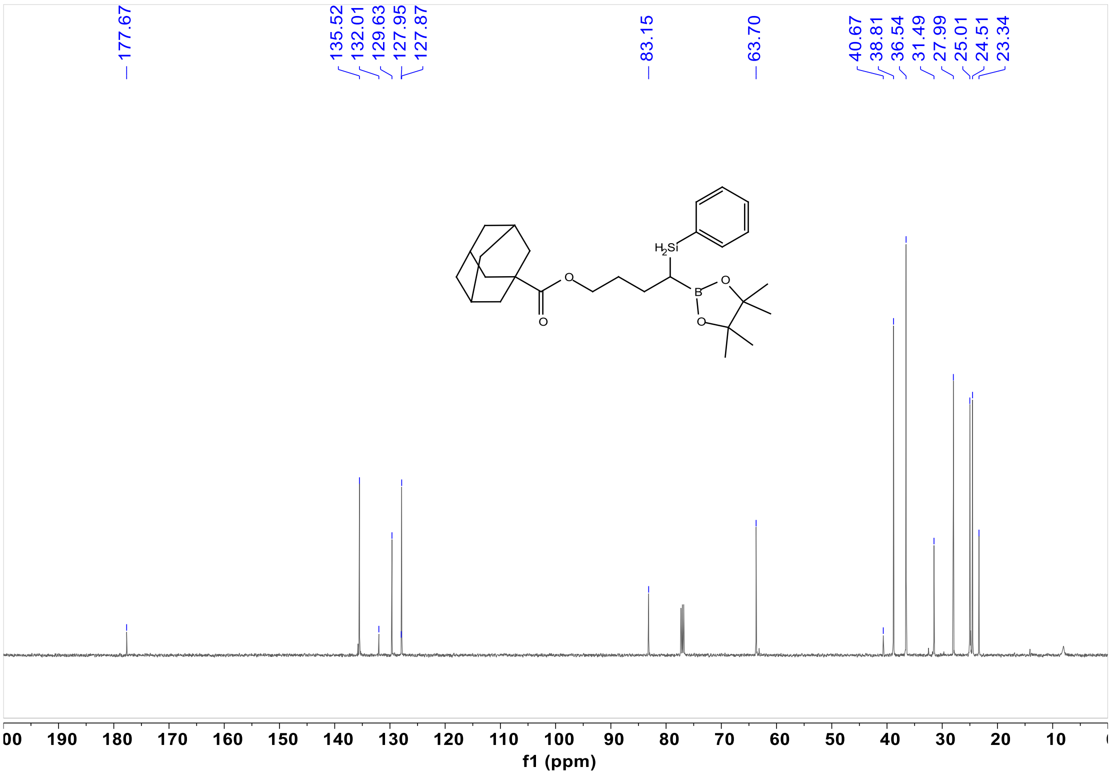

Supplementary Figure 66. ^{13}C NMR spectra for **18**

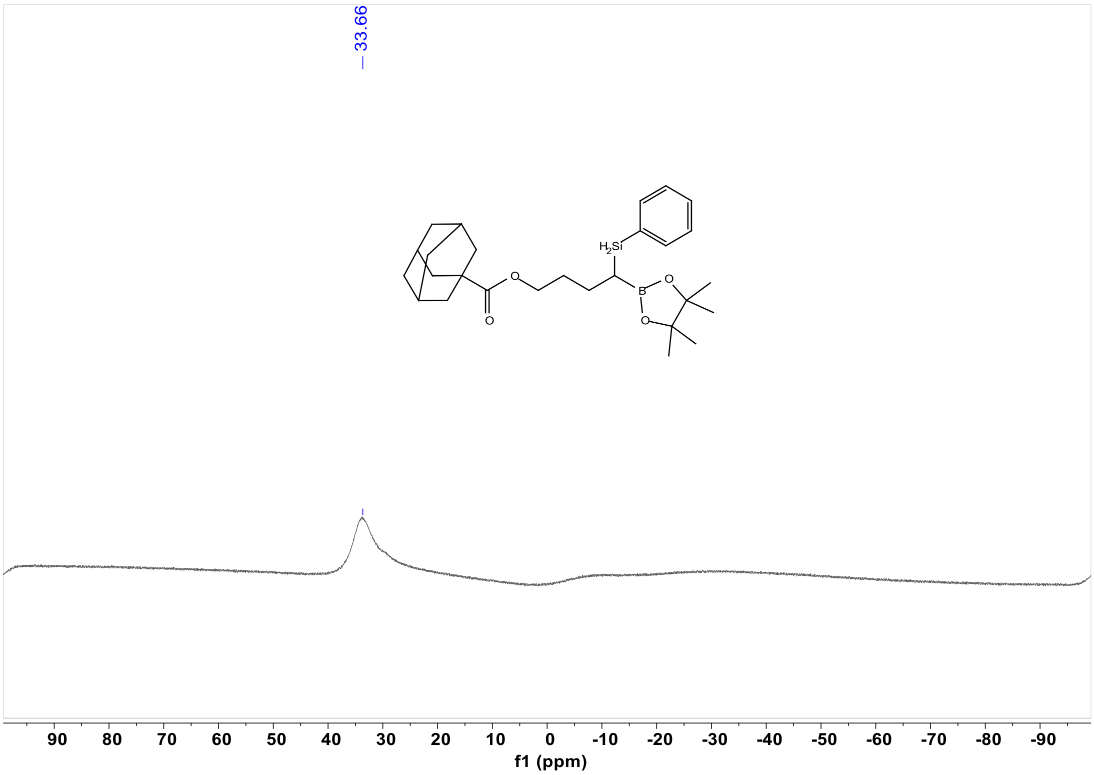

Supplementary Figure 67. ^{11}B NMR spectra for **18**

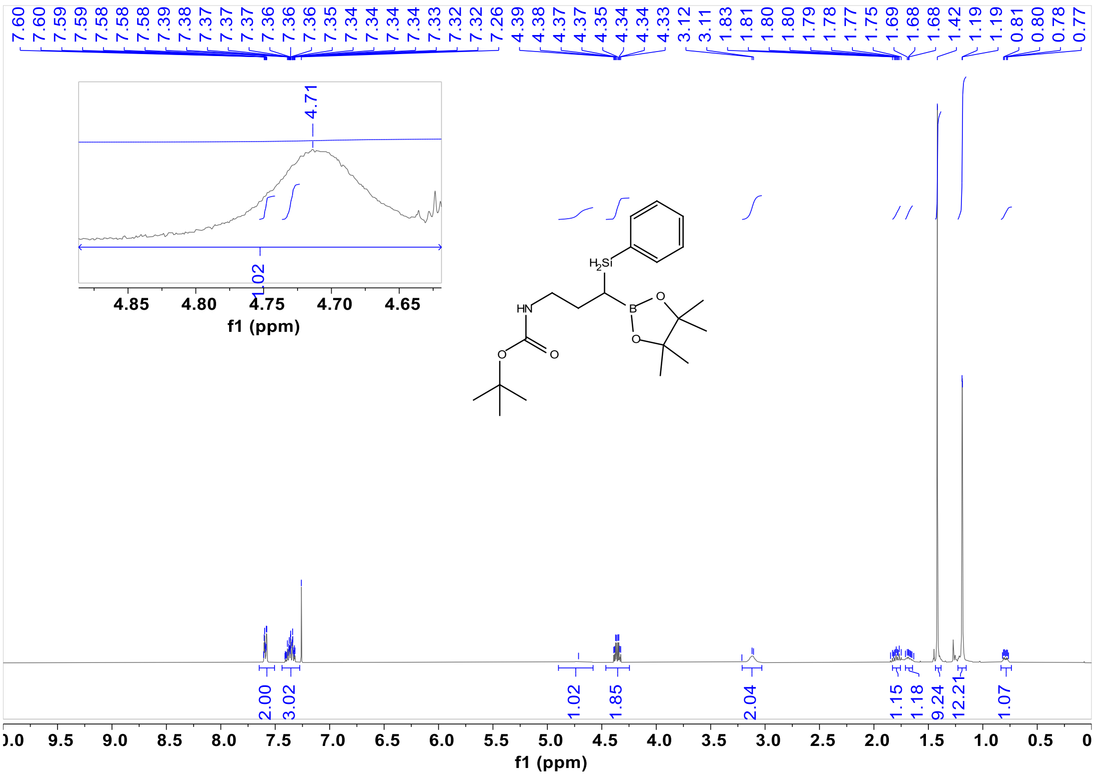

Supplementary Figure 68. ^{1}H NMR spectra for **19**

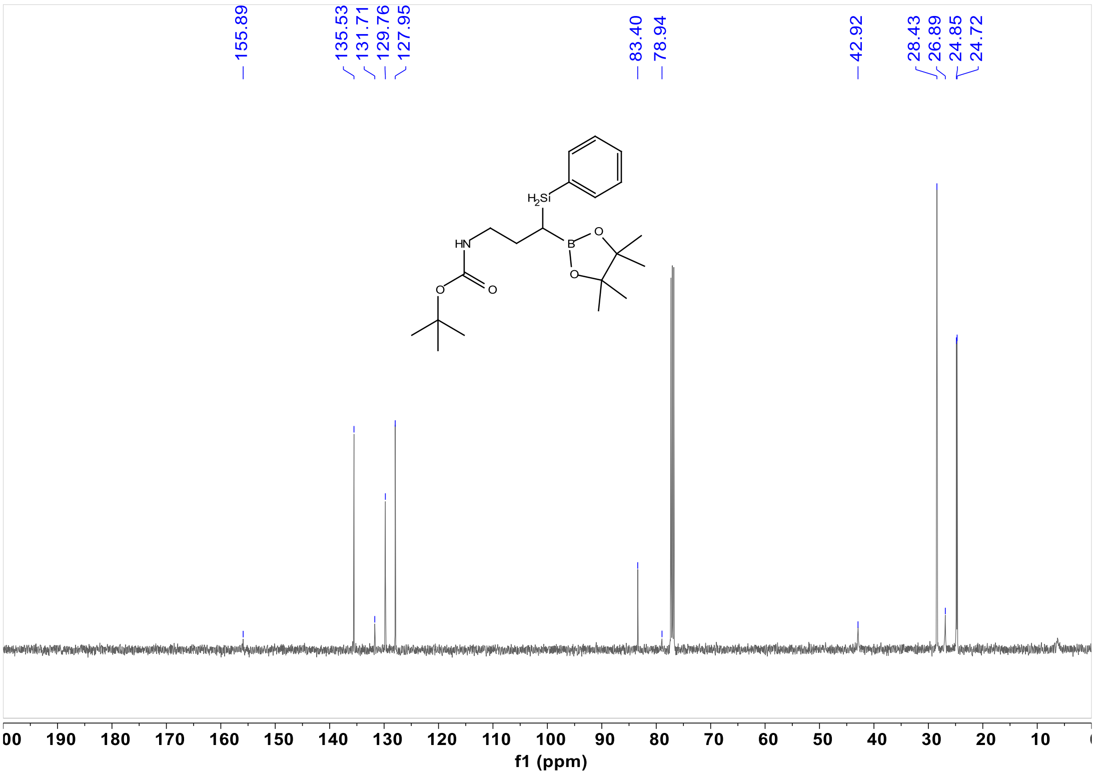

Supplementary Figure 69. ^{13}C NMR spectra for **19**

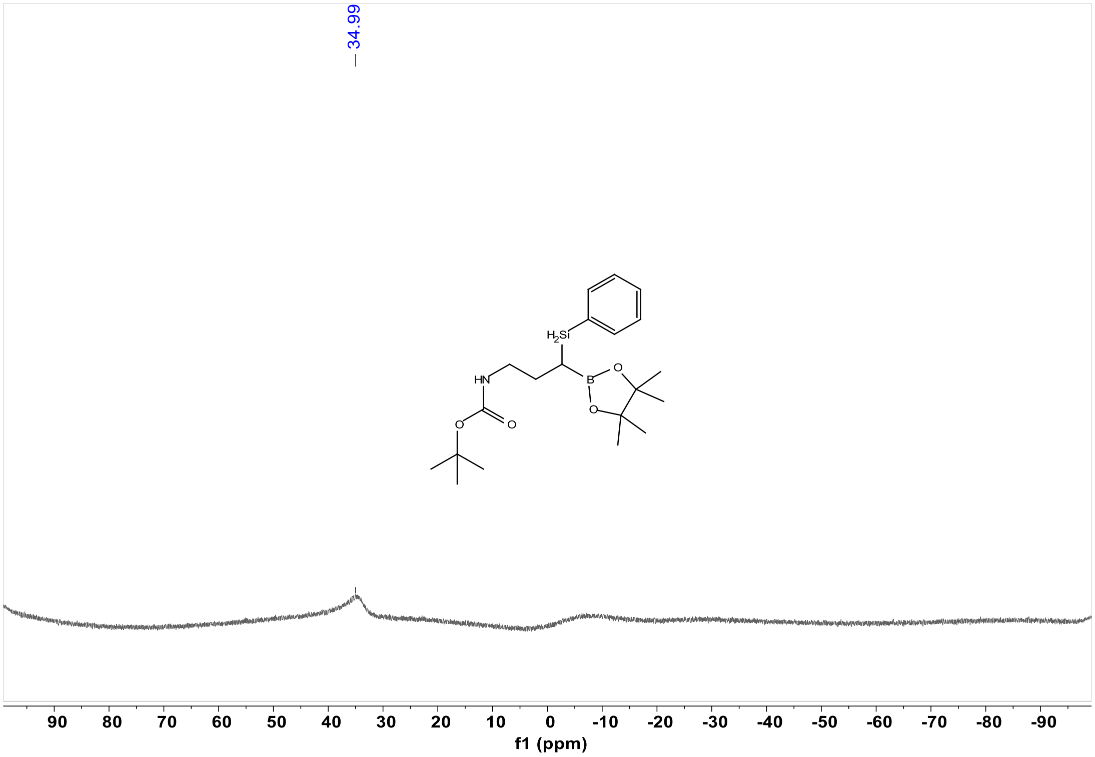

Supplementary Figure 70. ^{11}B NMR spectra for **19**

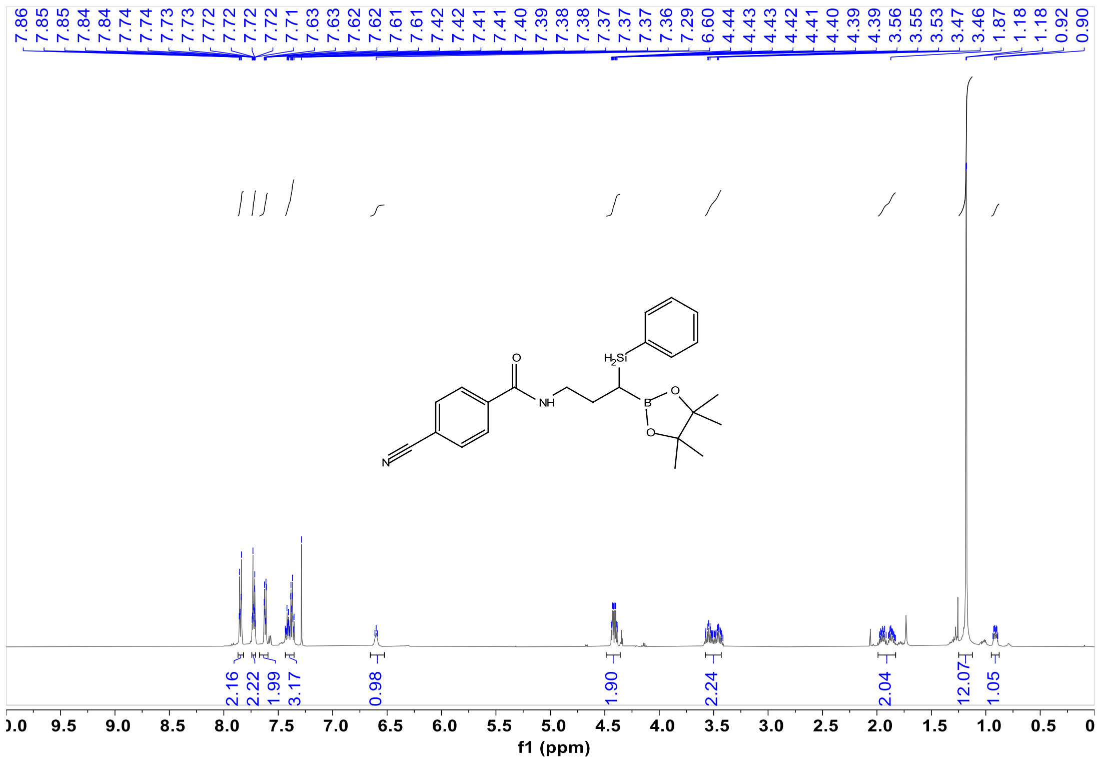

Supplementary Figure 71. ^{1}H NMR spectra for **20**

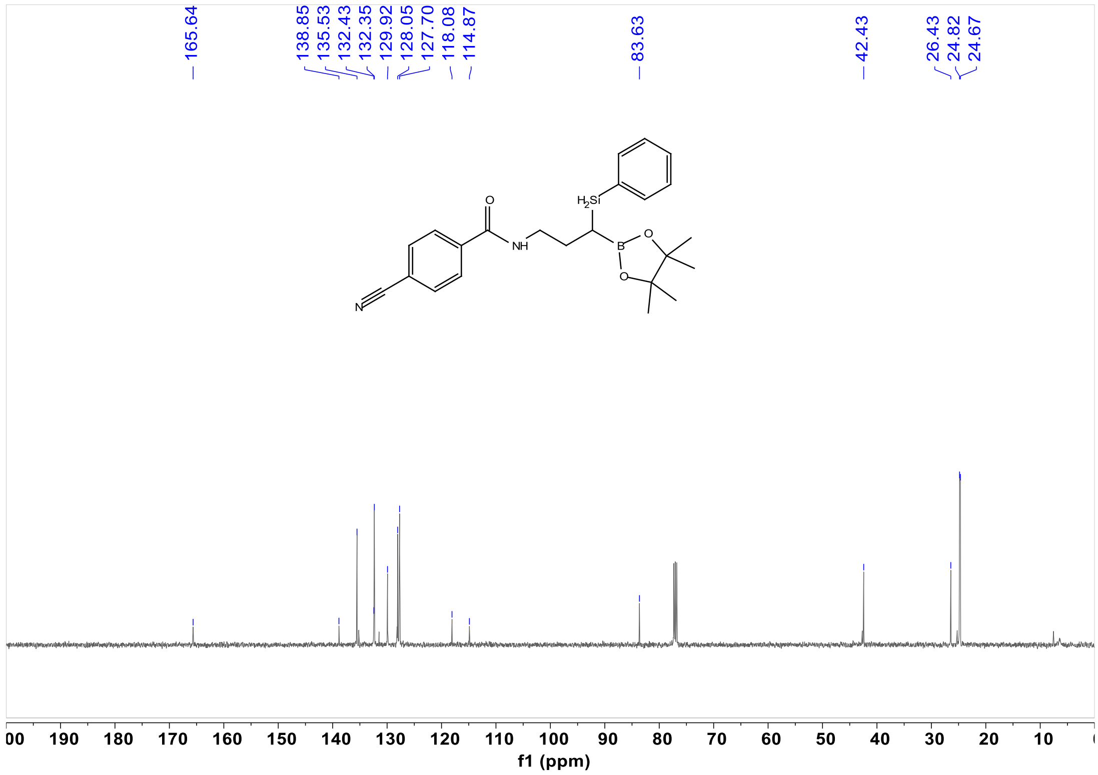

Supplementary Figure 72. ^{13}C NMR spectra for **20**

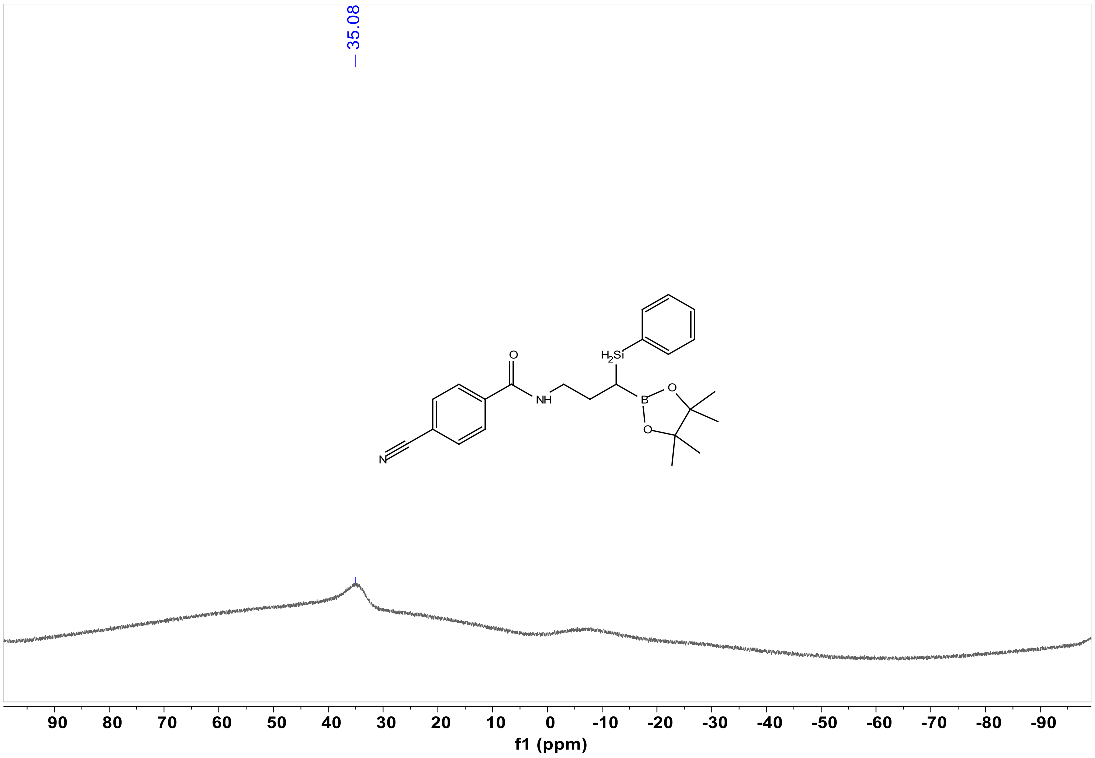

Supplementary Figure 73. ^{11}B NMR spectra for **20**

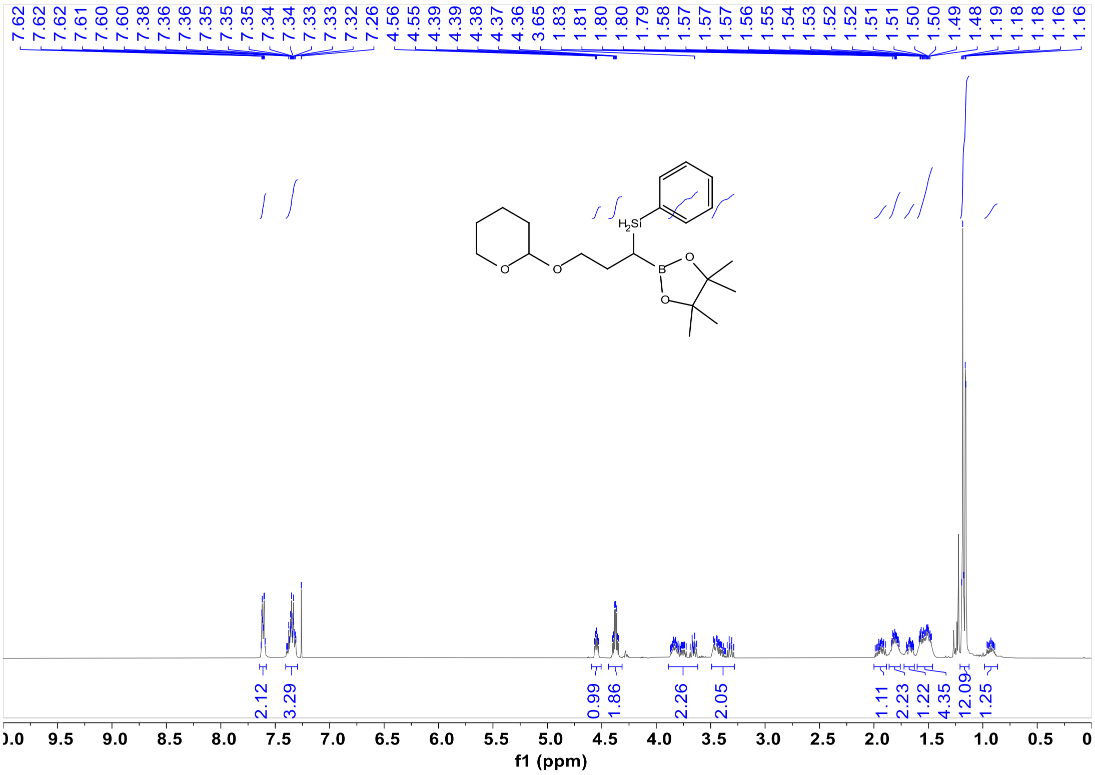

Supplementary Figure 74. ^{1}H NMR spectra for **21**

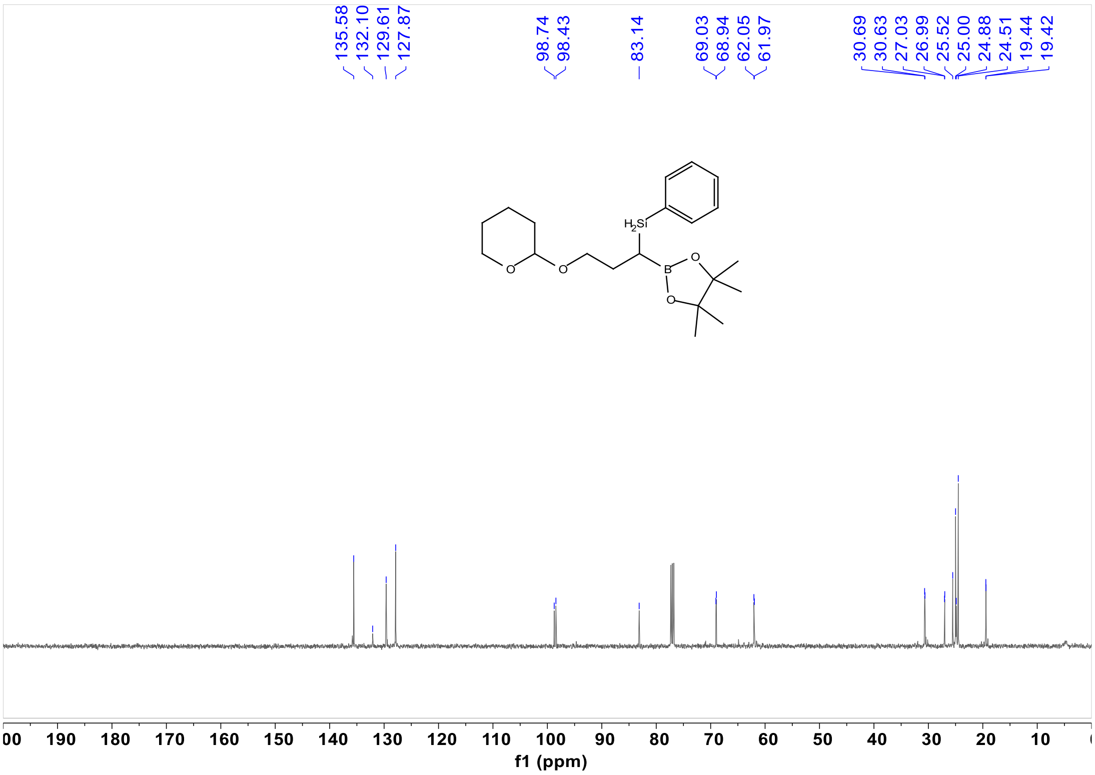

Supplementary Figure 75. ^{13}C NMR spectra for **21**

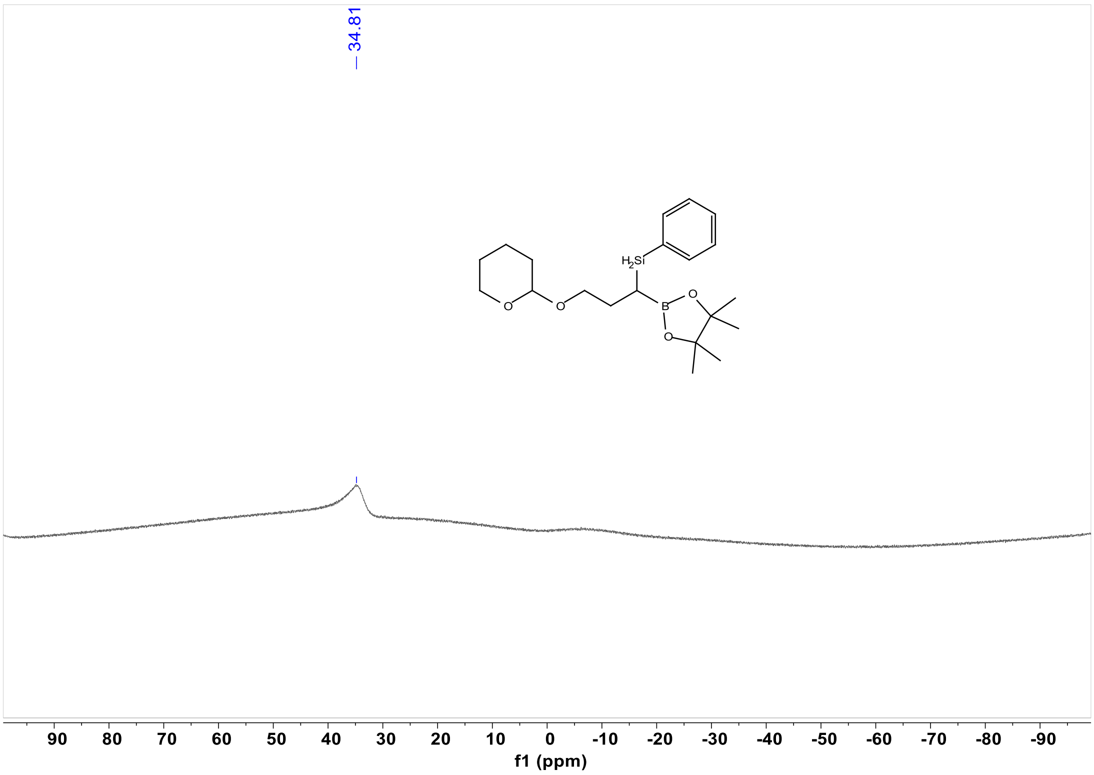

Supplementary Figure 76. ^{11}B NMR spectra for **21**

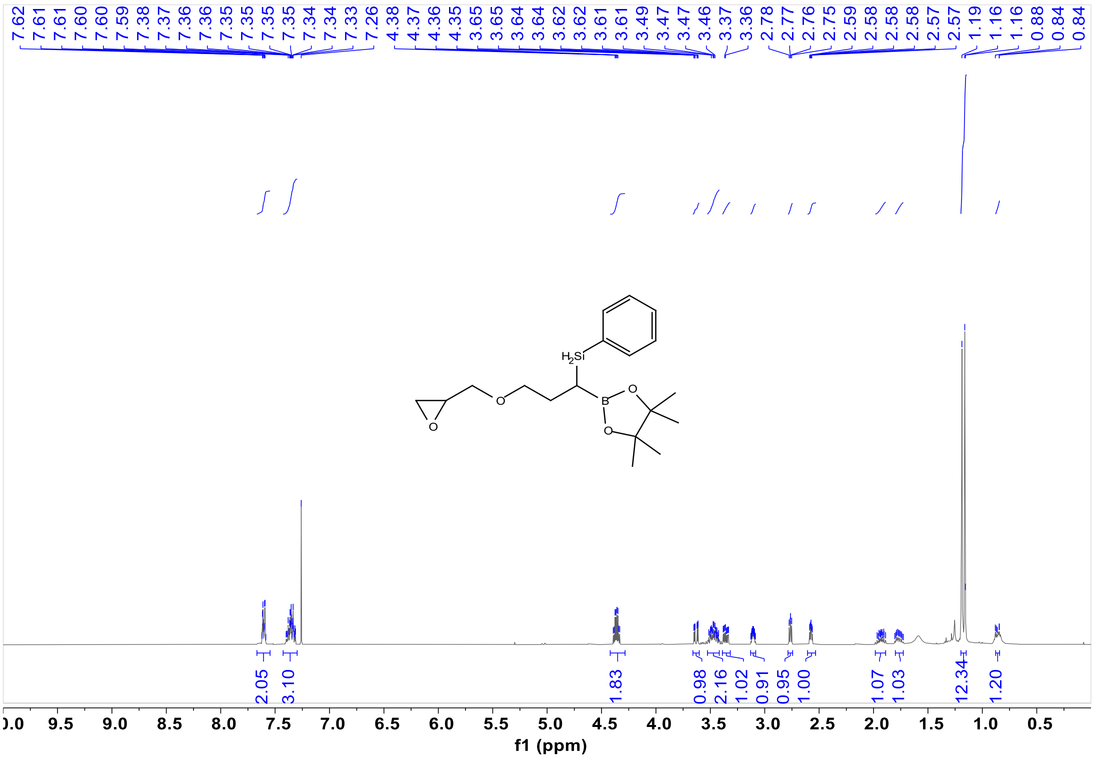

Supplementary Figure 77. ^{1}H NMR spectra for **22**

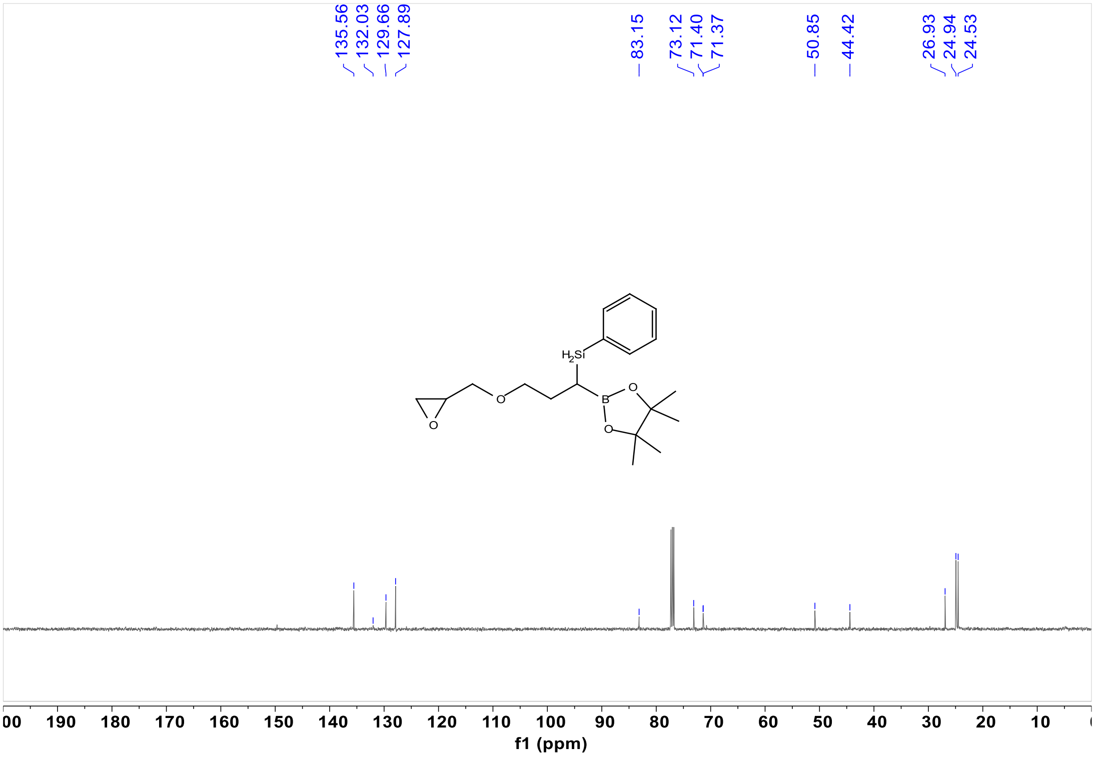

Supplementary Figure 78. ^{13}C NMR spectra for **22**

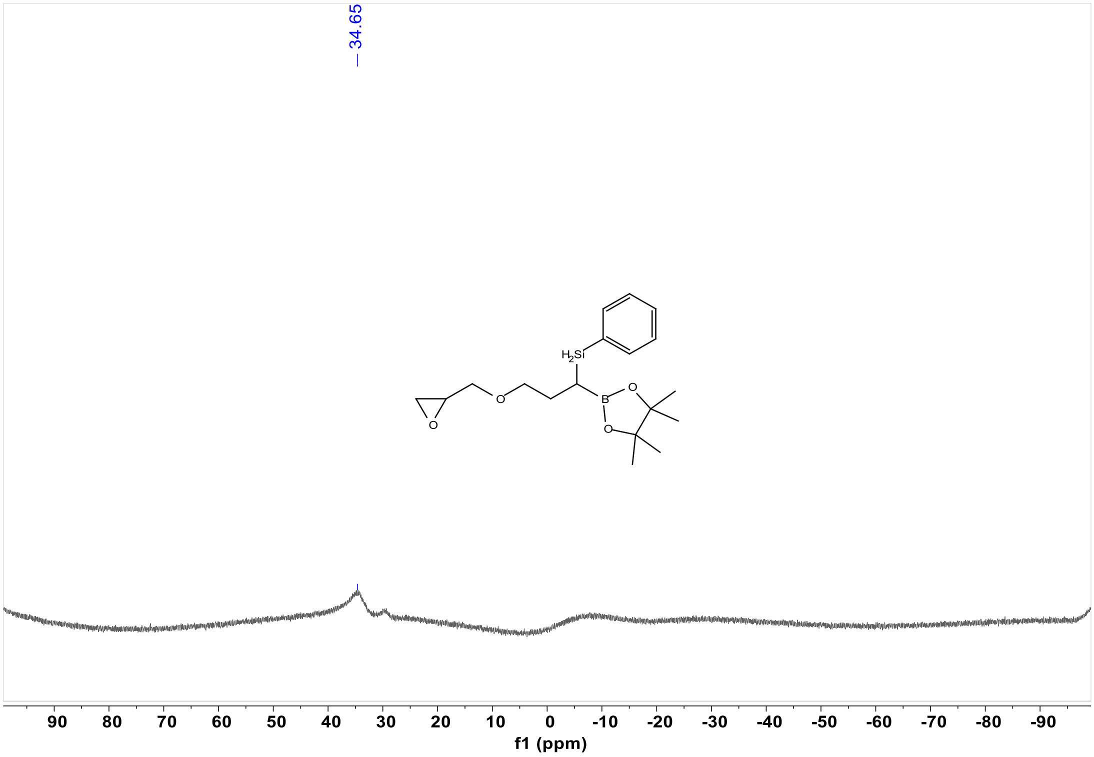

Supplementary Figure 79. ^{11}B NMR spectra for **22**

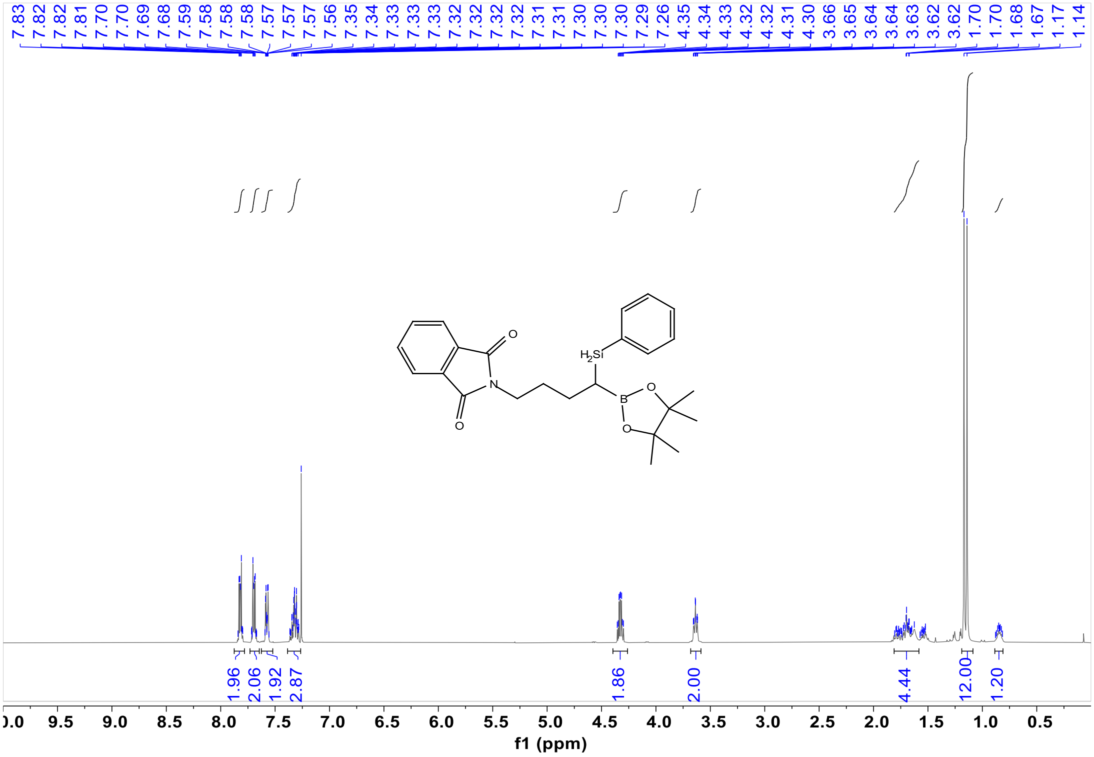

Supplementary Figure 80. ^{1}H NMR spectra for **23**

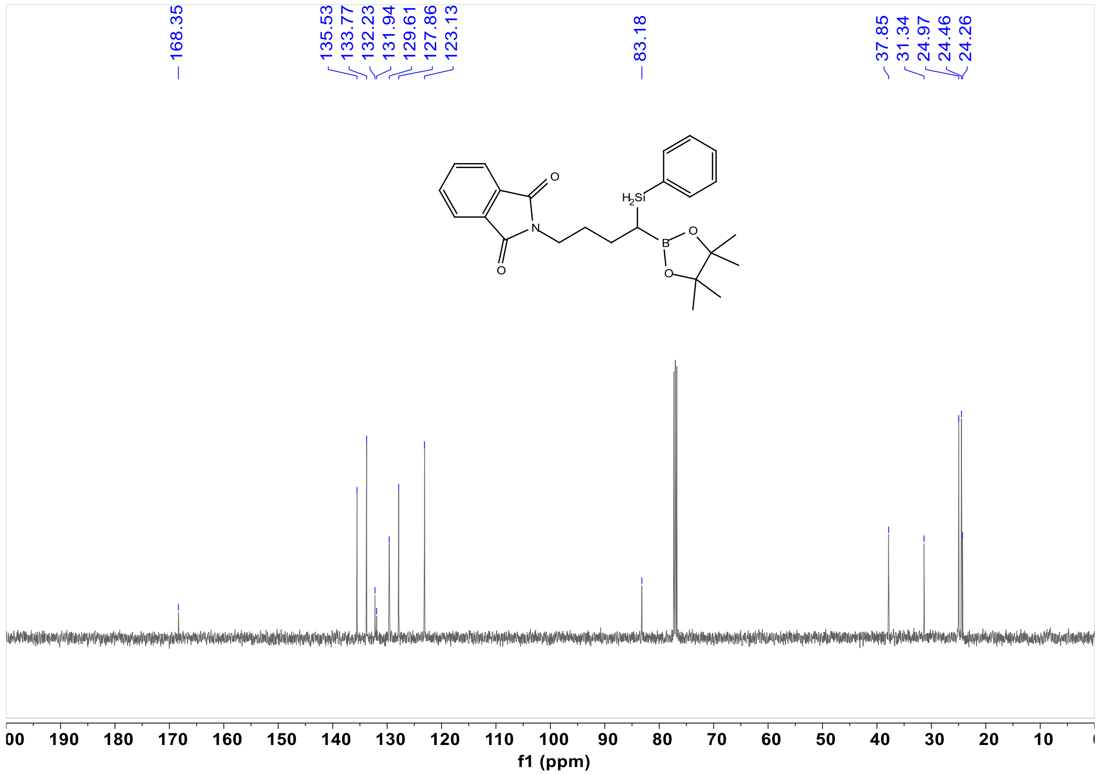

Supplementary Figure 81. ^{13}C NMR spectra for **23**

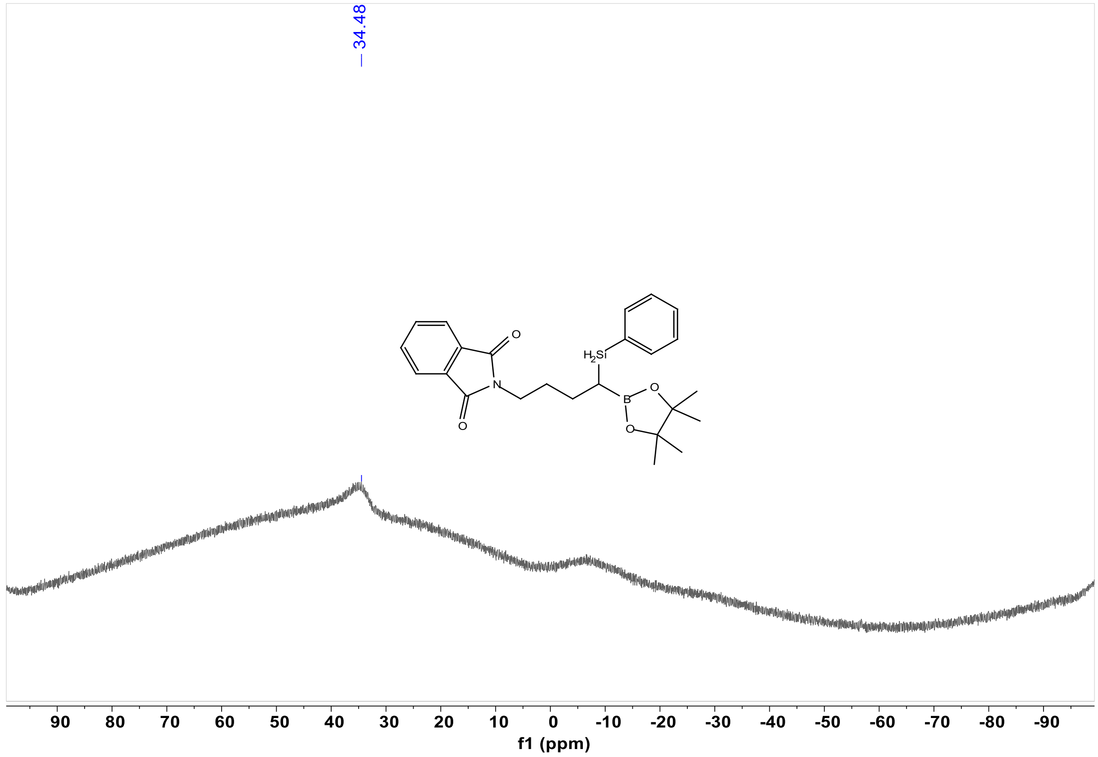

Supplementary Figure 82. ^{11}B NMR spectra for **23**

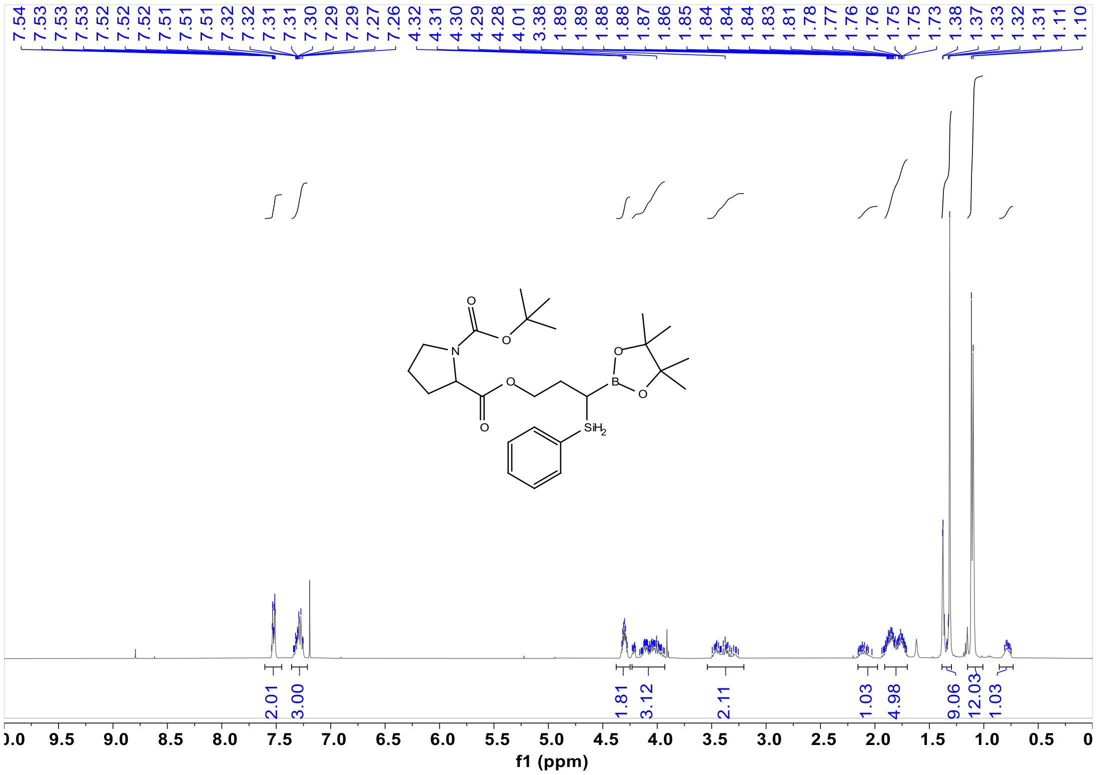

Supplementary Figure 83. ^{1}H NMR spectra for **24**

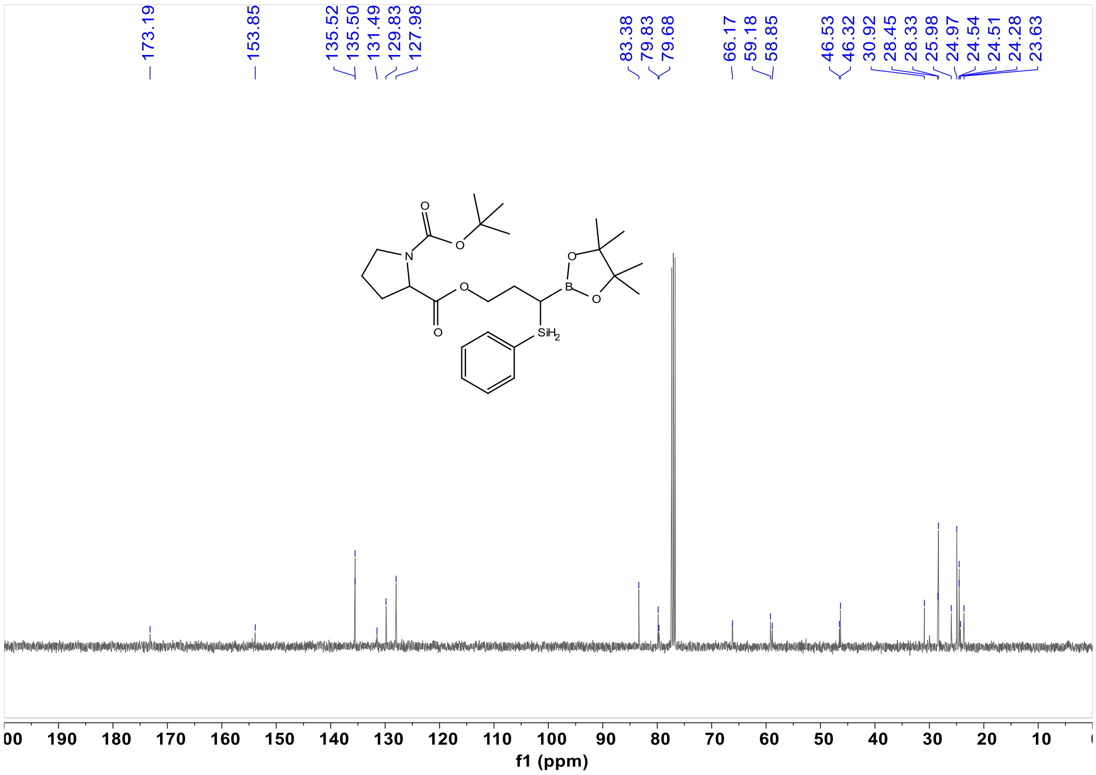

Supplementary Figure 84. ^{13}C NMR spectra for **24**

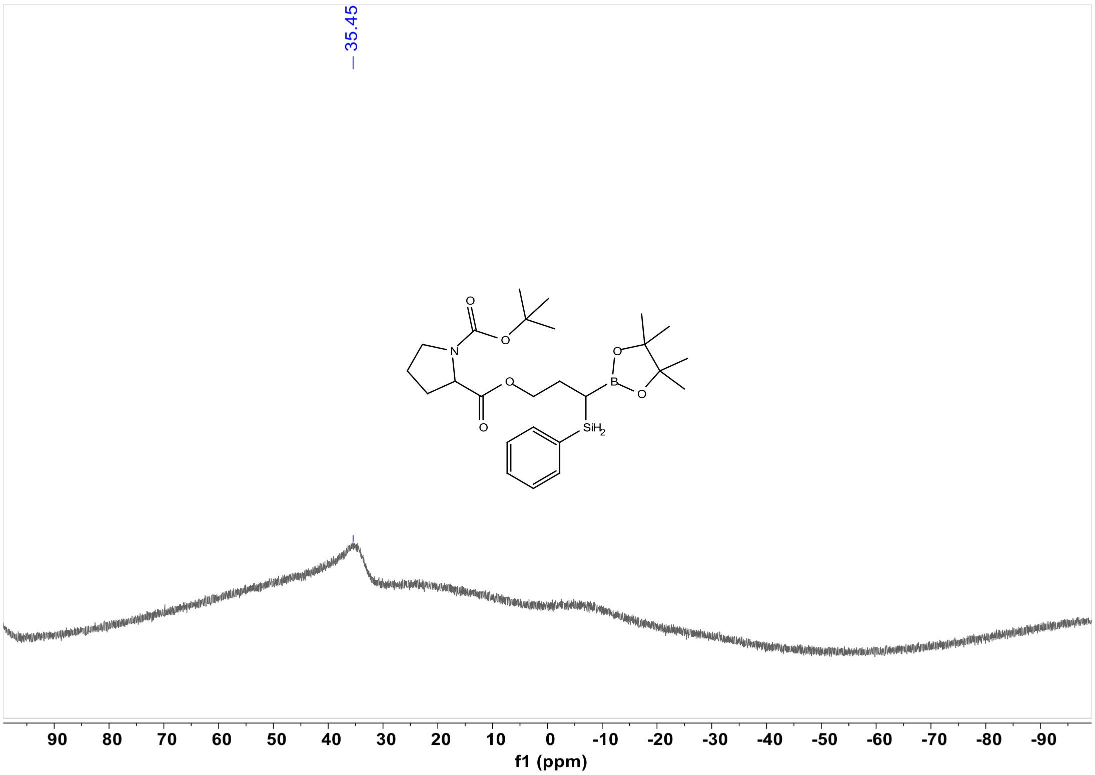

Supplementary Figure 85. ^{11}B NMR spectra for **24**

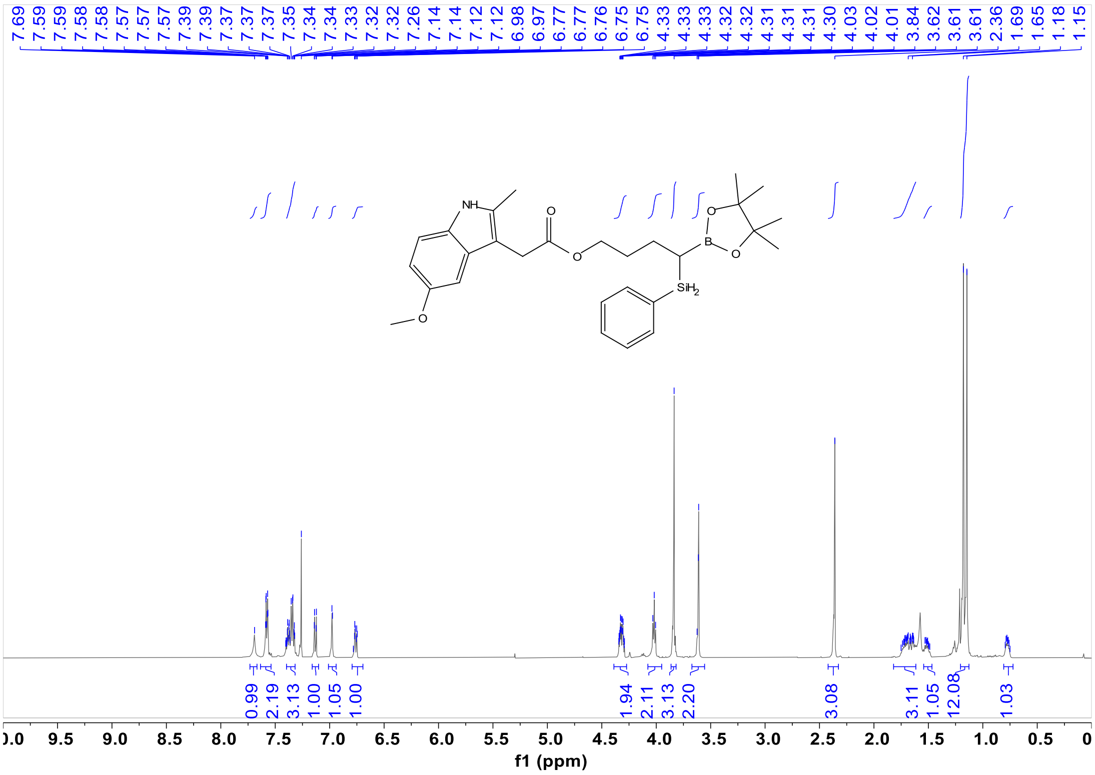

Supplementary Figure 86. ^{1}H NMR spectra for **25**

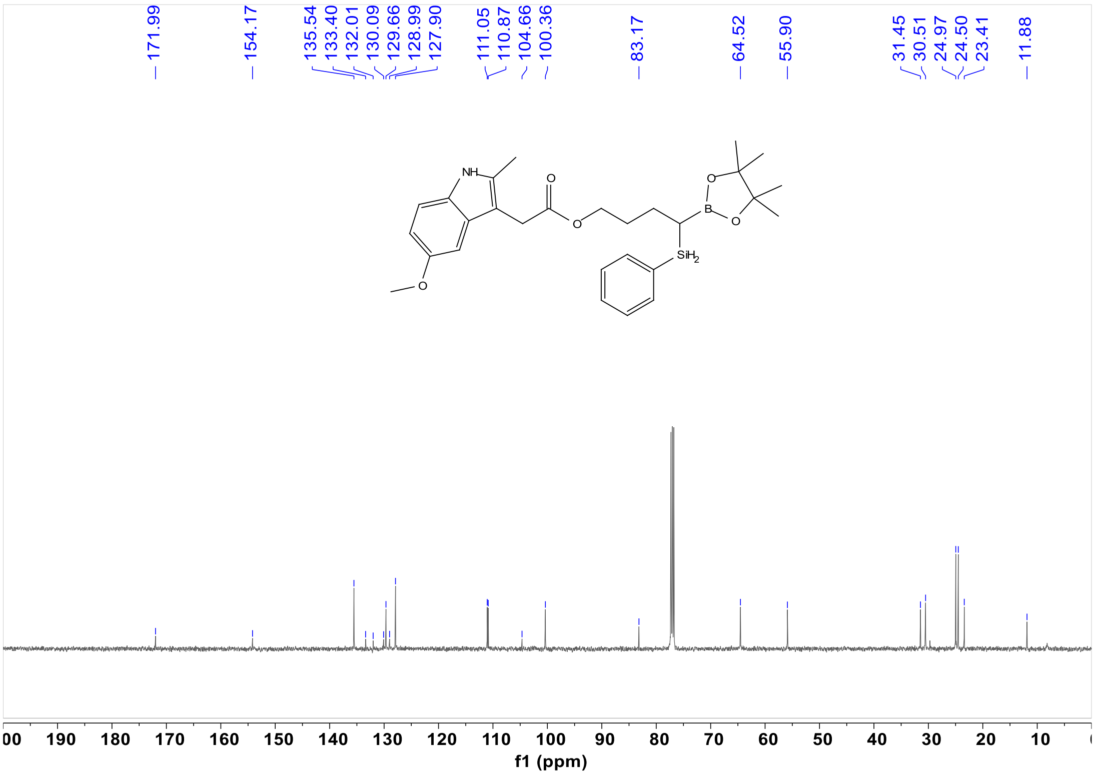

Supplementary Figure 87. ^{13}C NMR spectra for **25**

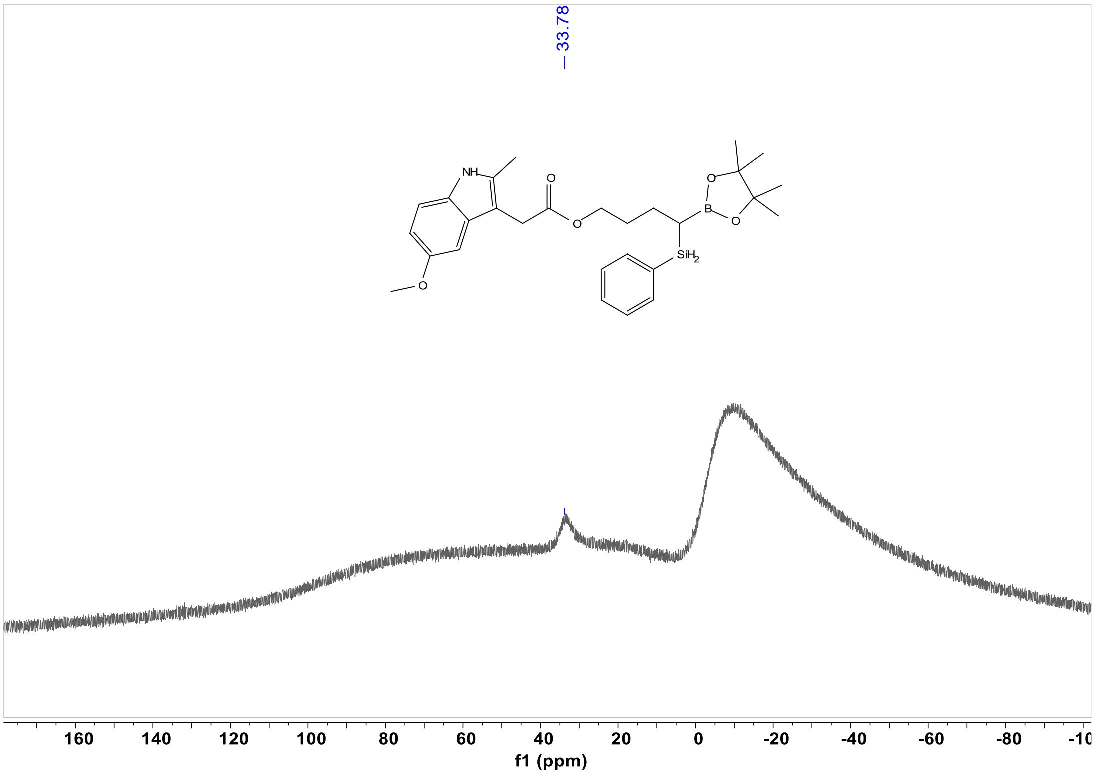

Supplementary Figure 88. ^{11}B NMR spectra for **25**

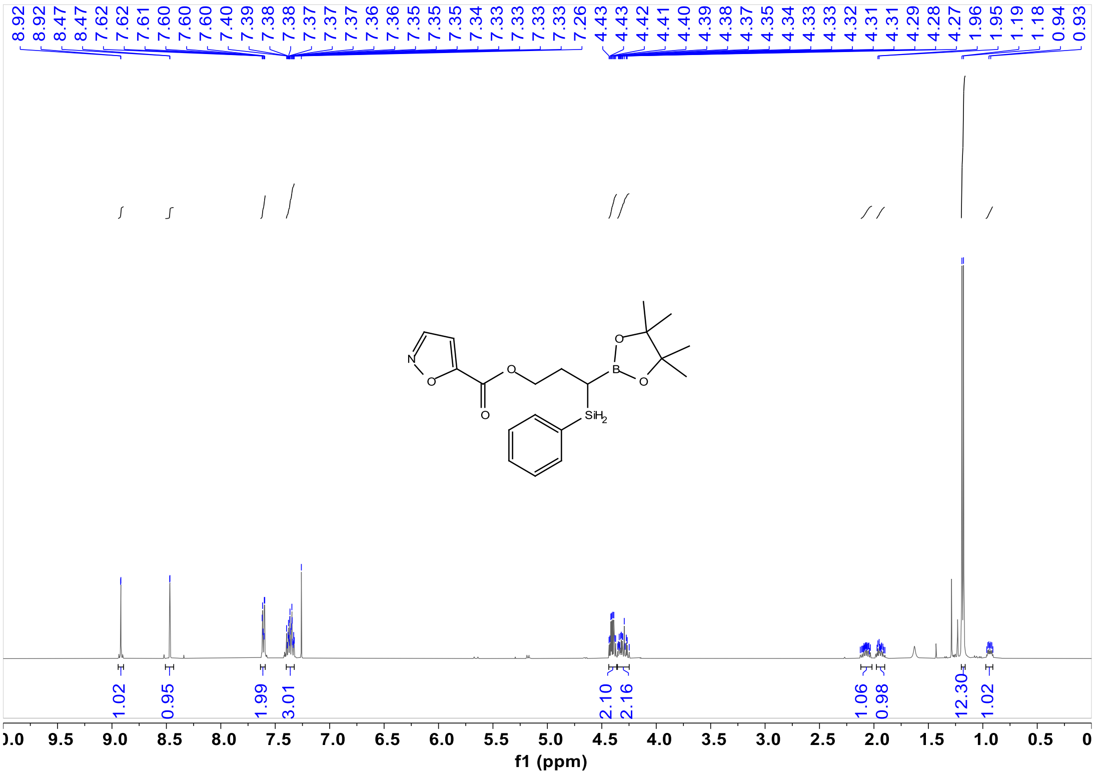

Supplementary Figure 89. ^{1}H NMR spectra for **26**

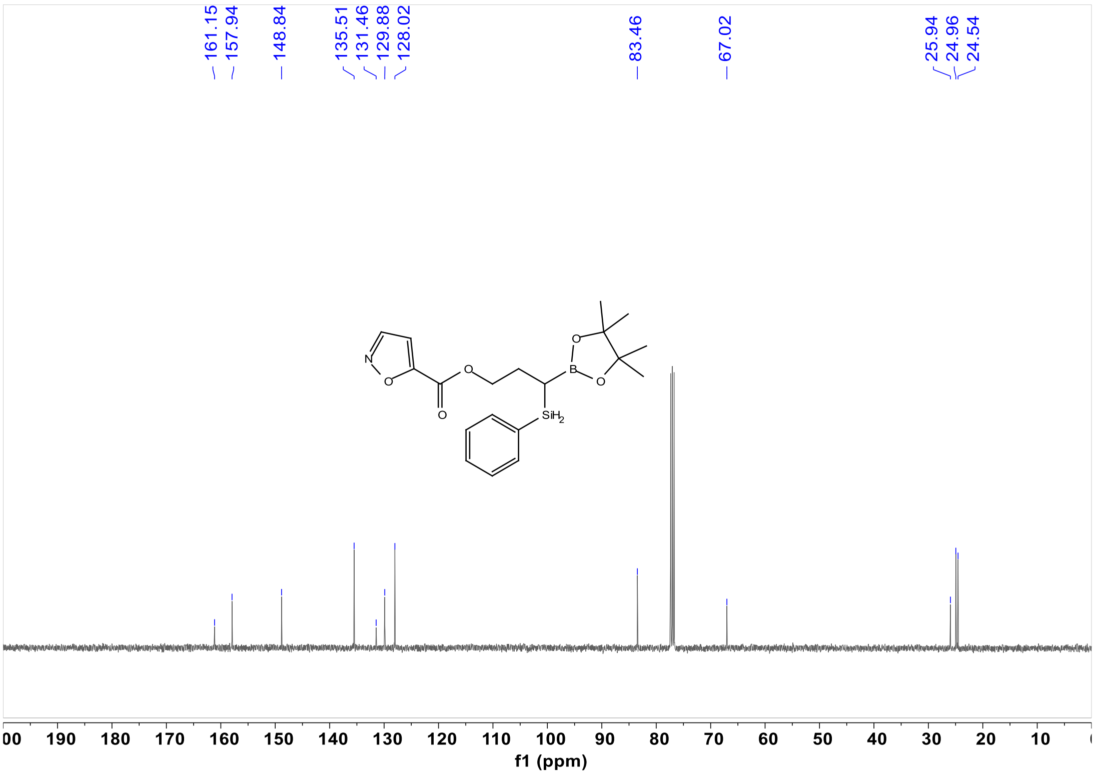

Supplementary Figure 90. ^{13}C NMR spectra for **26**

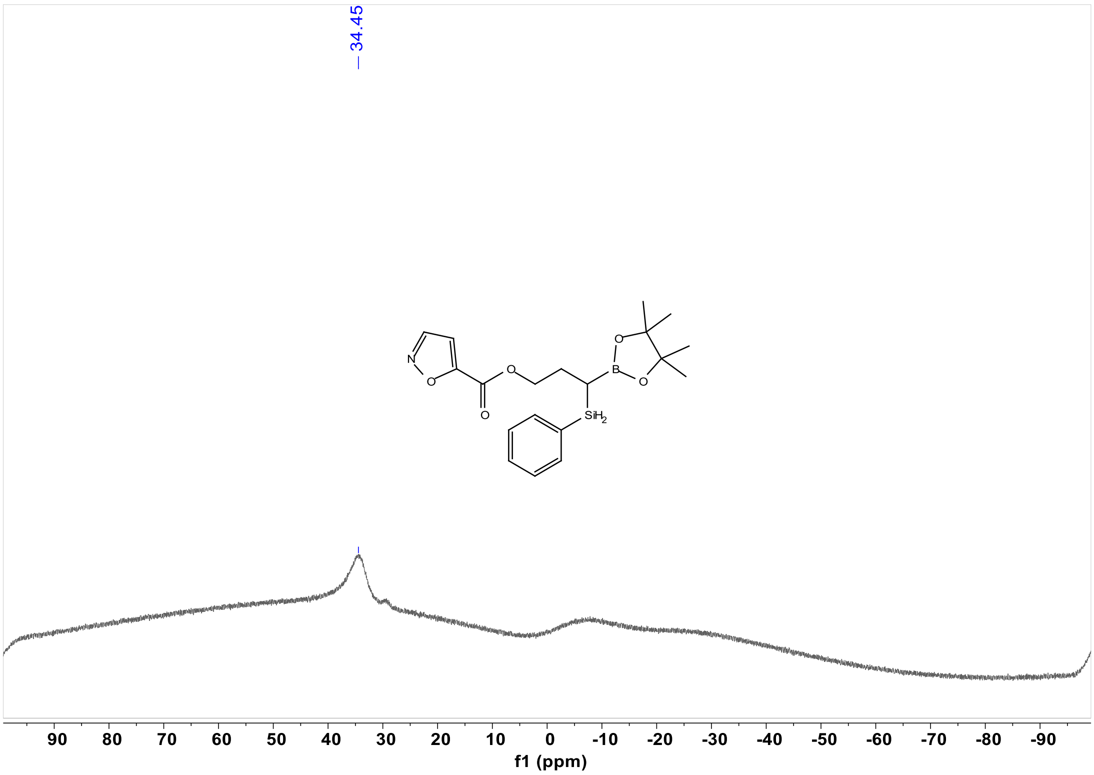

Supplementary Figure 91. ^{11}B NMR spectra for **26**

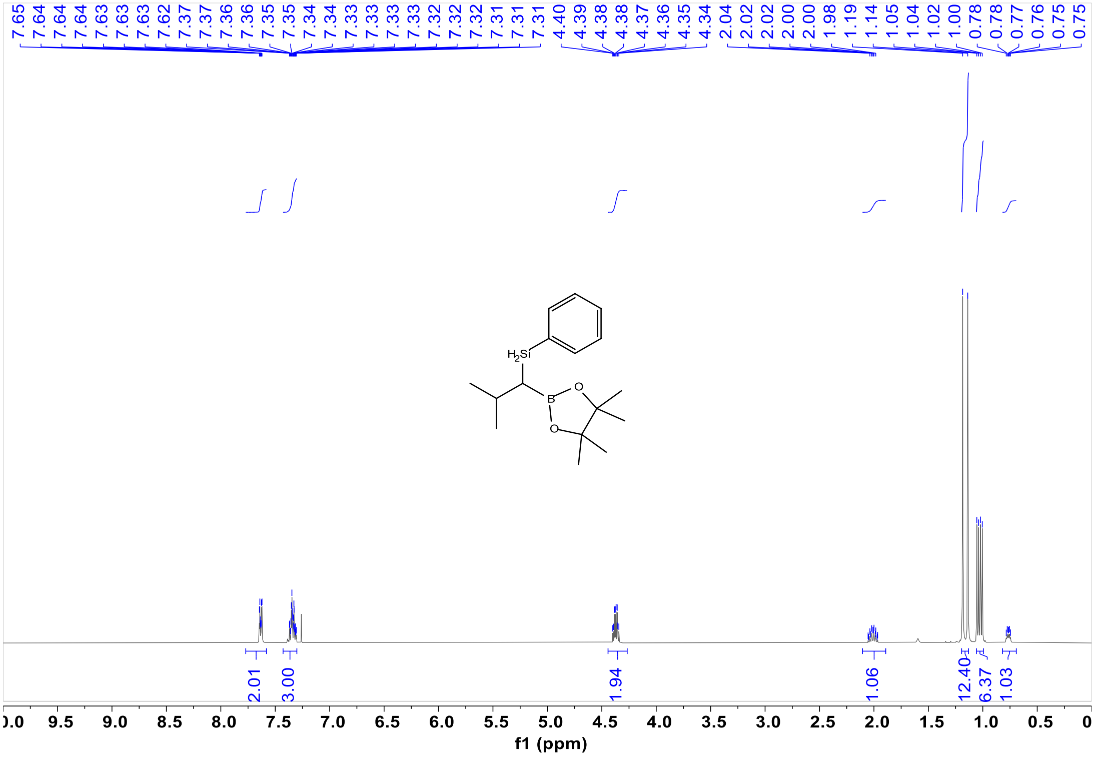

Supplementary Figure 92. ^{1}H NMR spectra for **27**

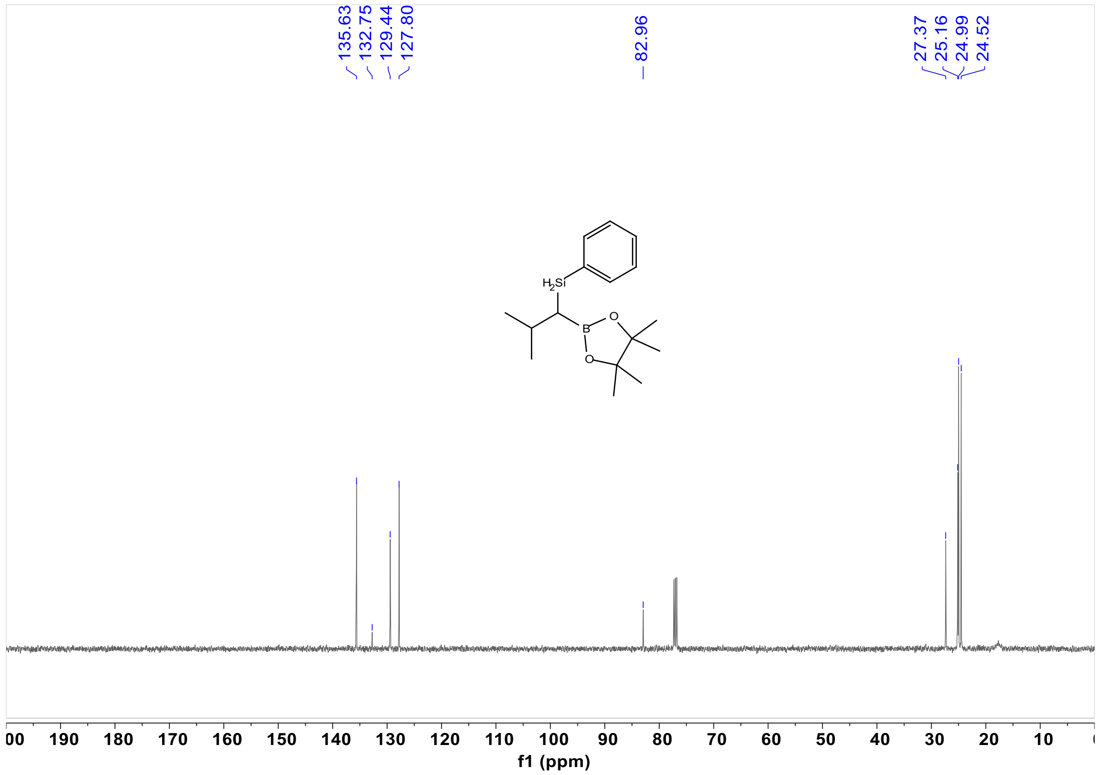

Supplementary Figure 93. ^{13}C NMR spectra for **27**

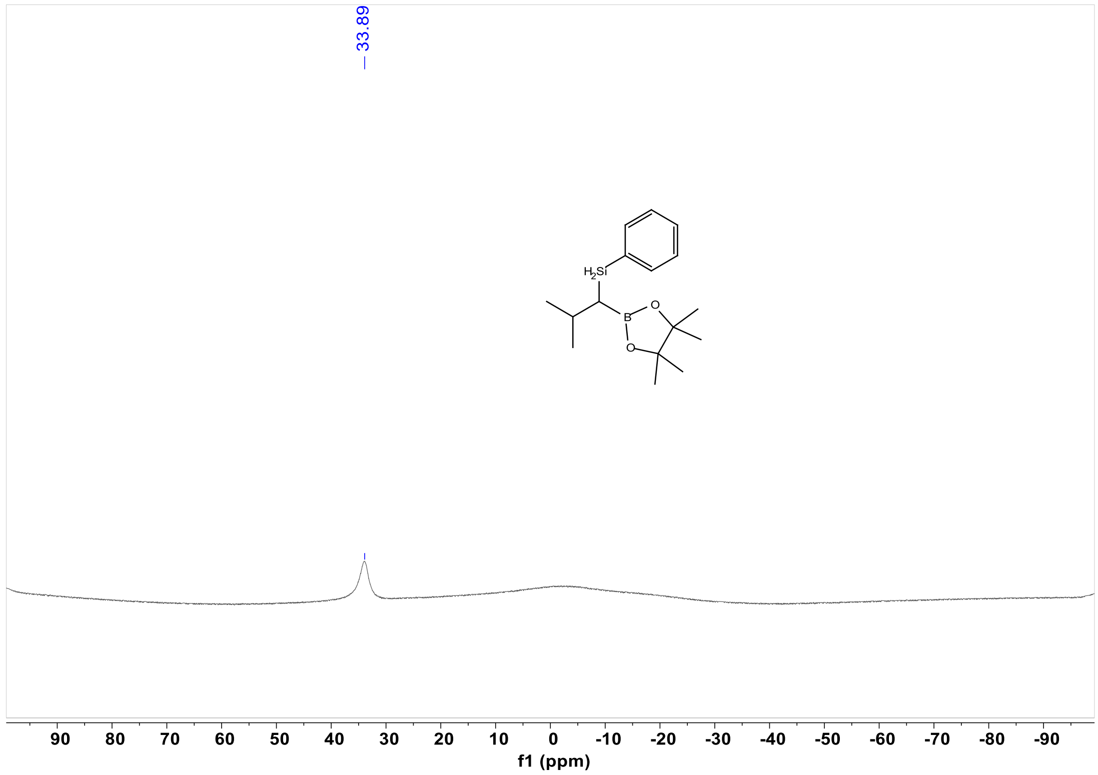

Supplementary Figure 94. ^{11}B NMR spectra for **27**

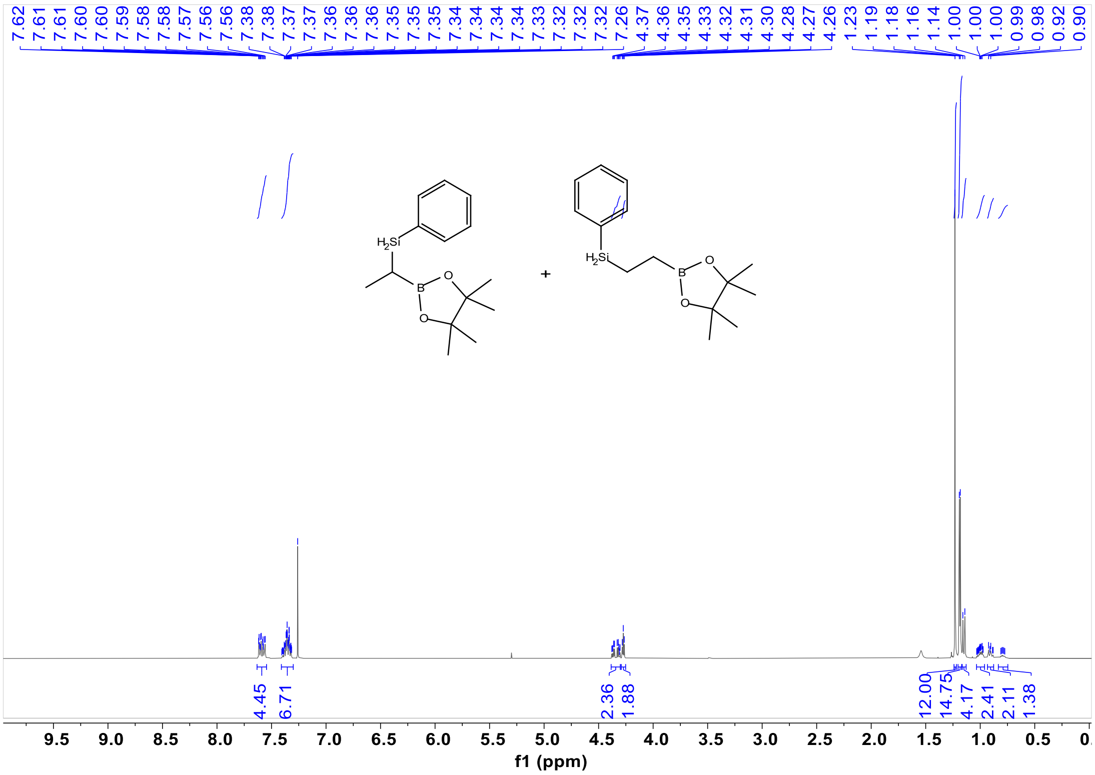

Supplementary Figure 95. ^{1}H NMR spectra for **28** and **28'**

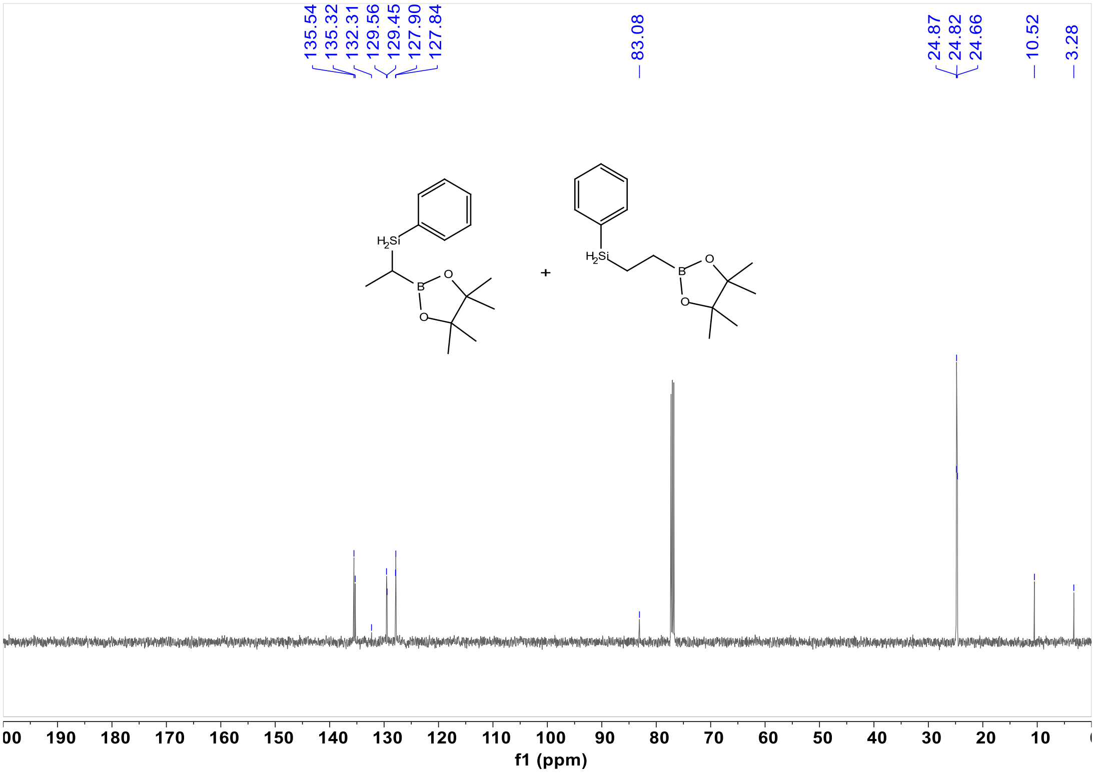

Supplementary Figure 96. ^{13}C NMR spectra for **28** and **28'**

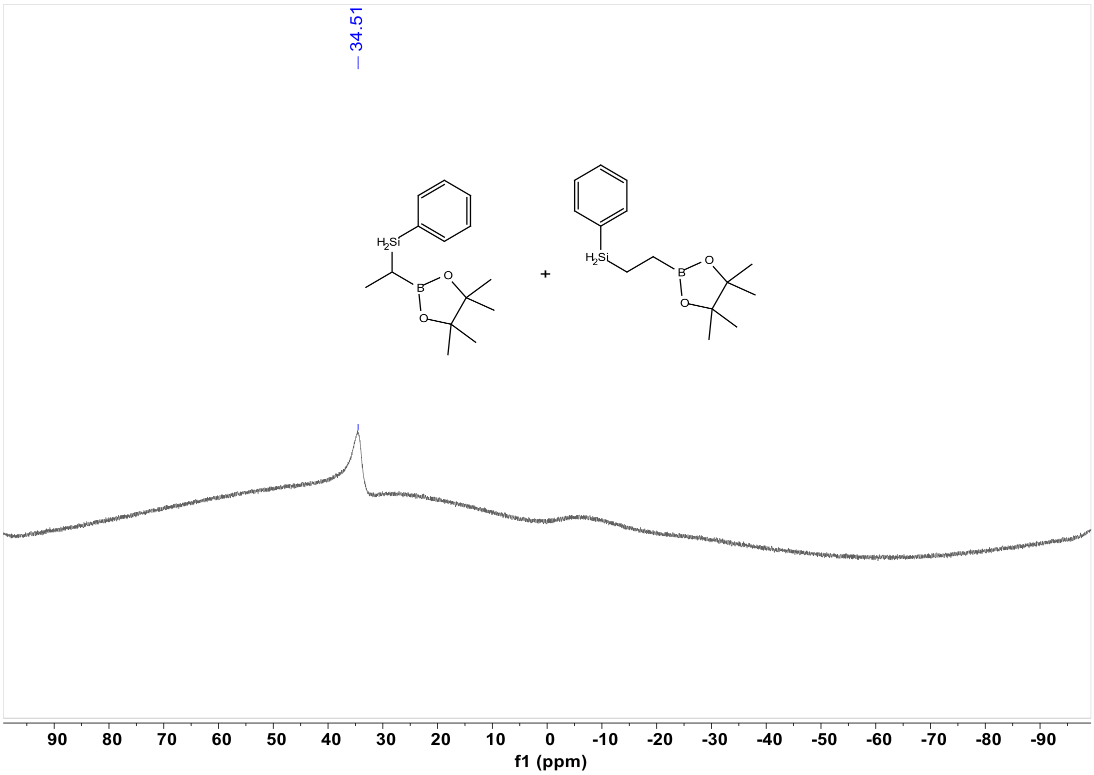

Supplementary Figure 97. ^{11}B NMR spectra for **28** and **28'**

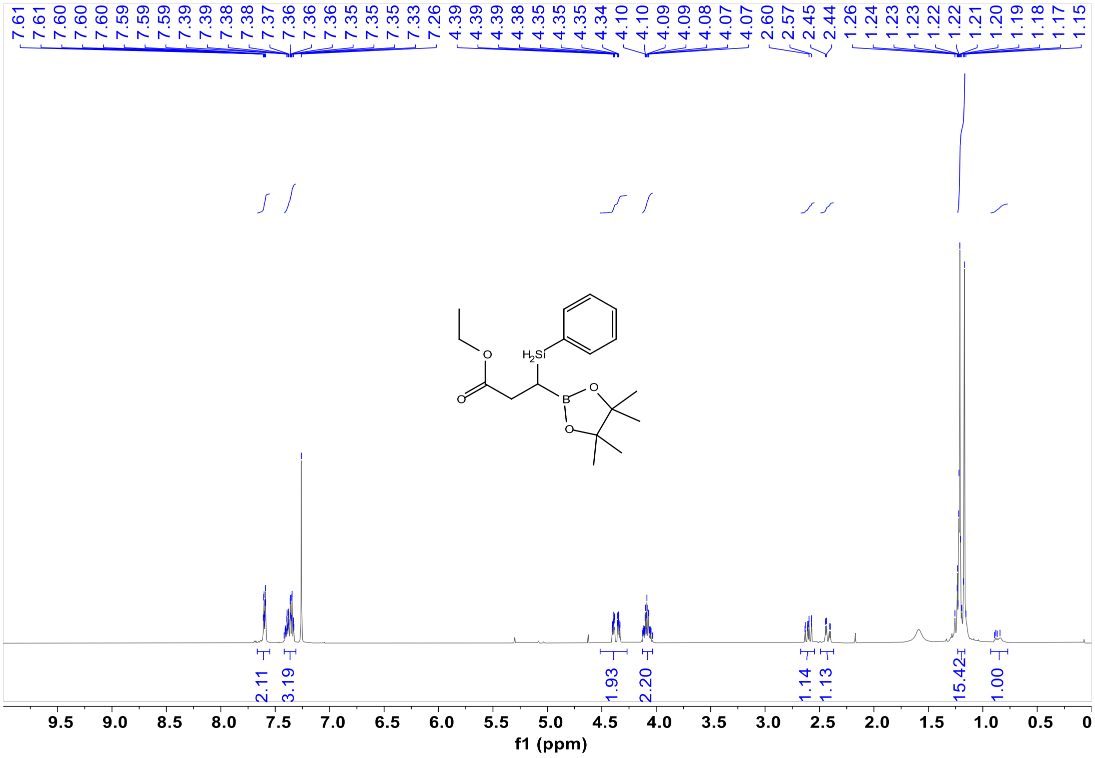

Supplementary Figure 98. ^{1}H NMR spectra for **30**

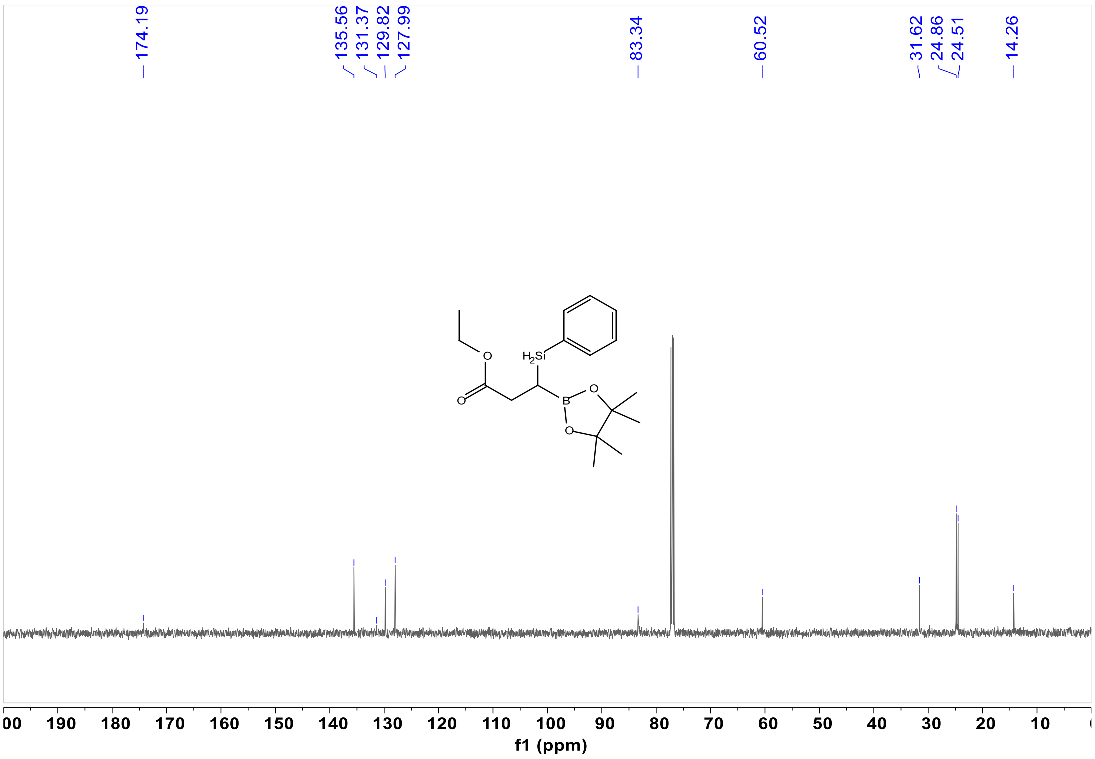

Supplementary Figure 99. ^{13}C NMR spectra for **30**

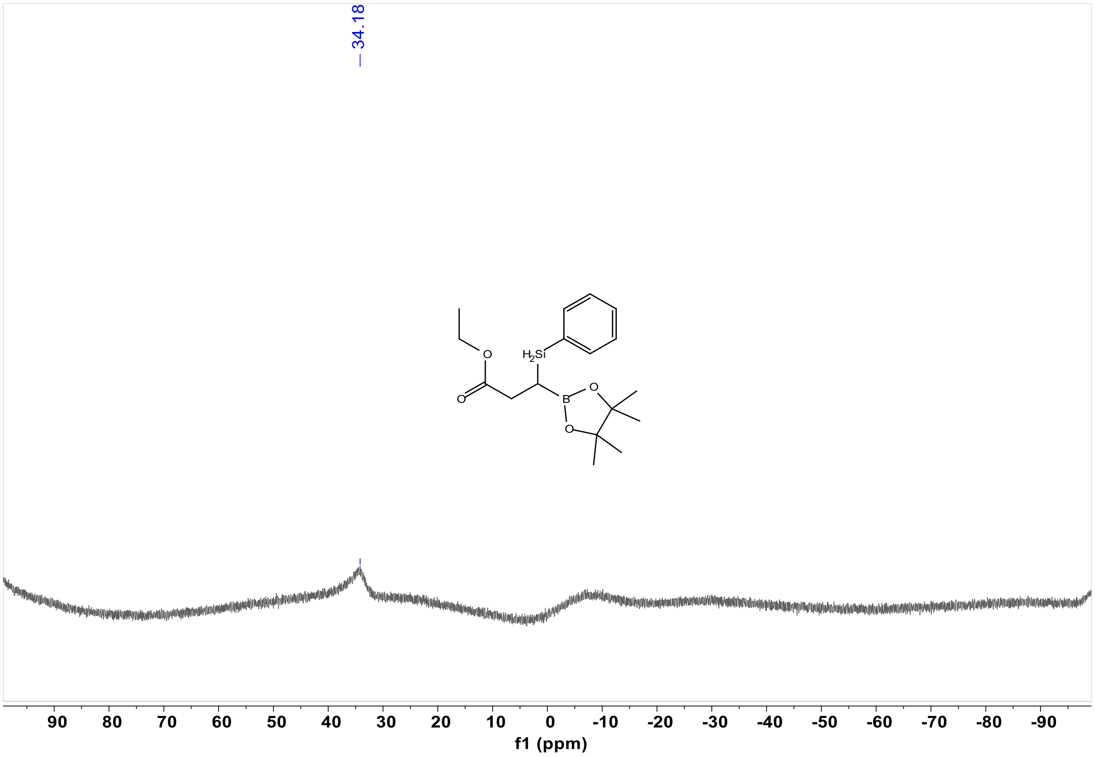

Supplementary Figure 100. ^{11}B NMR spectra for **30**

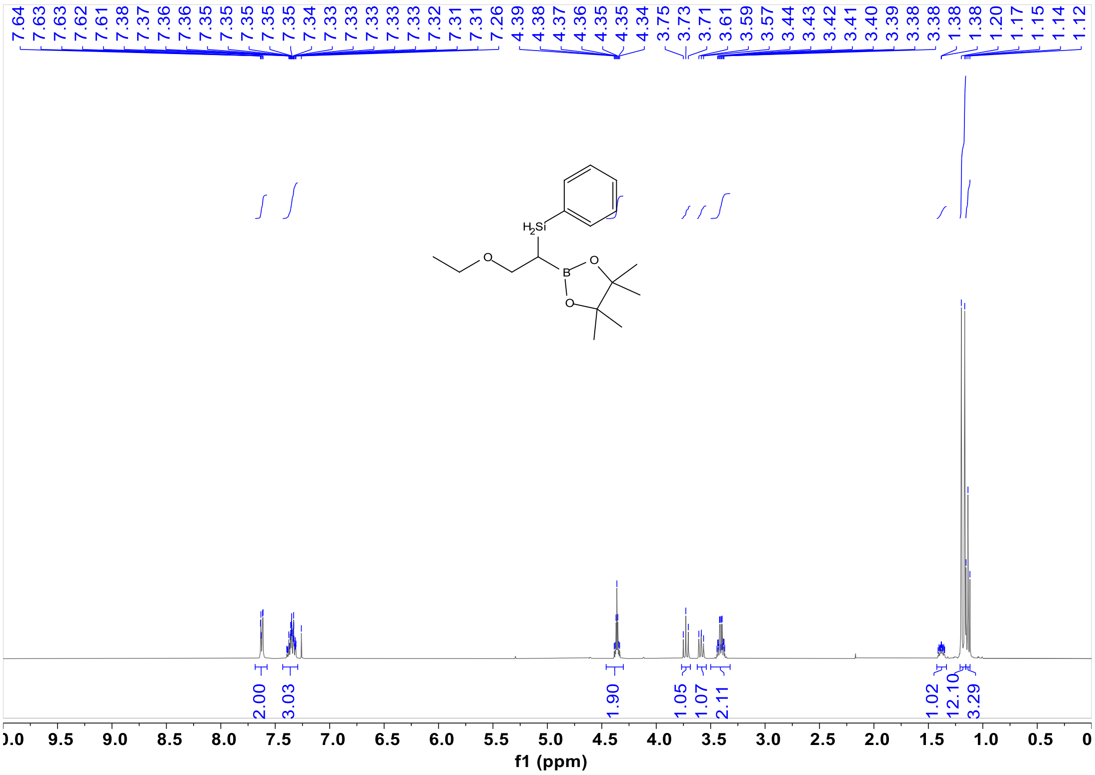

Supplementary Figure 101. ^{1}H NMR spectra for **31**

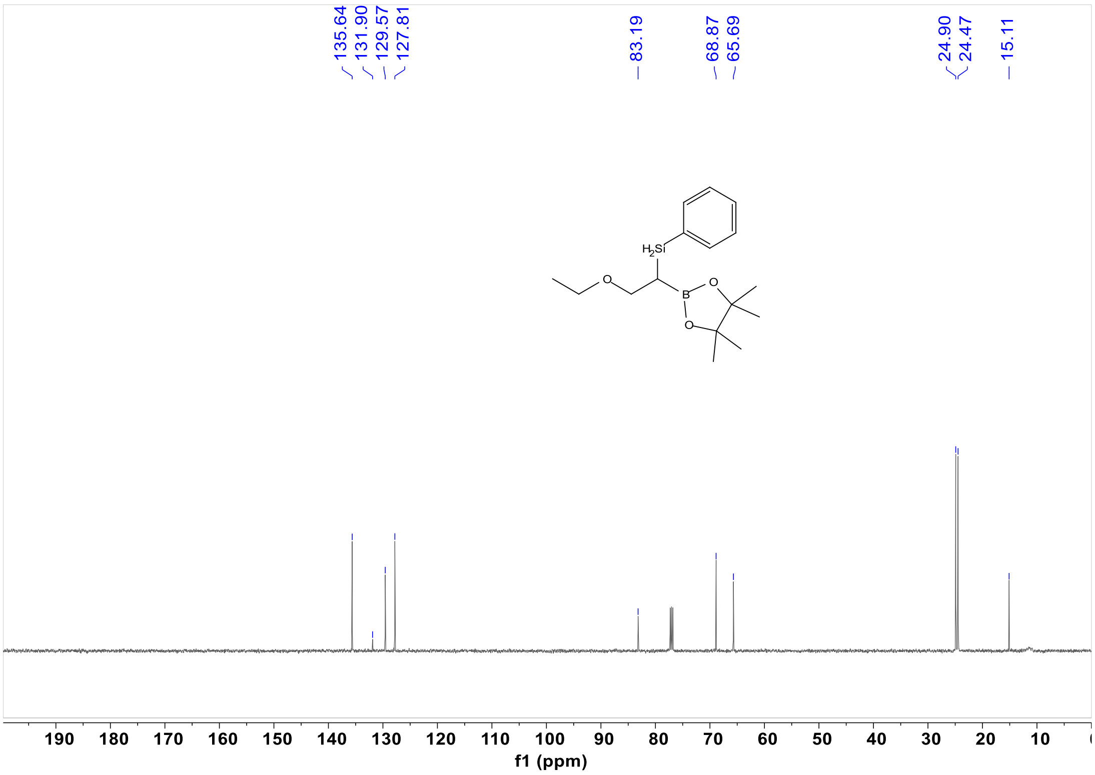

Supplementary Figure 102. ^{13}C NMR spectra for **31**

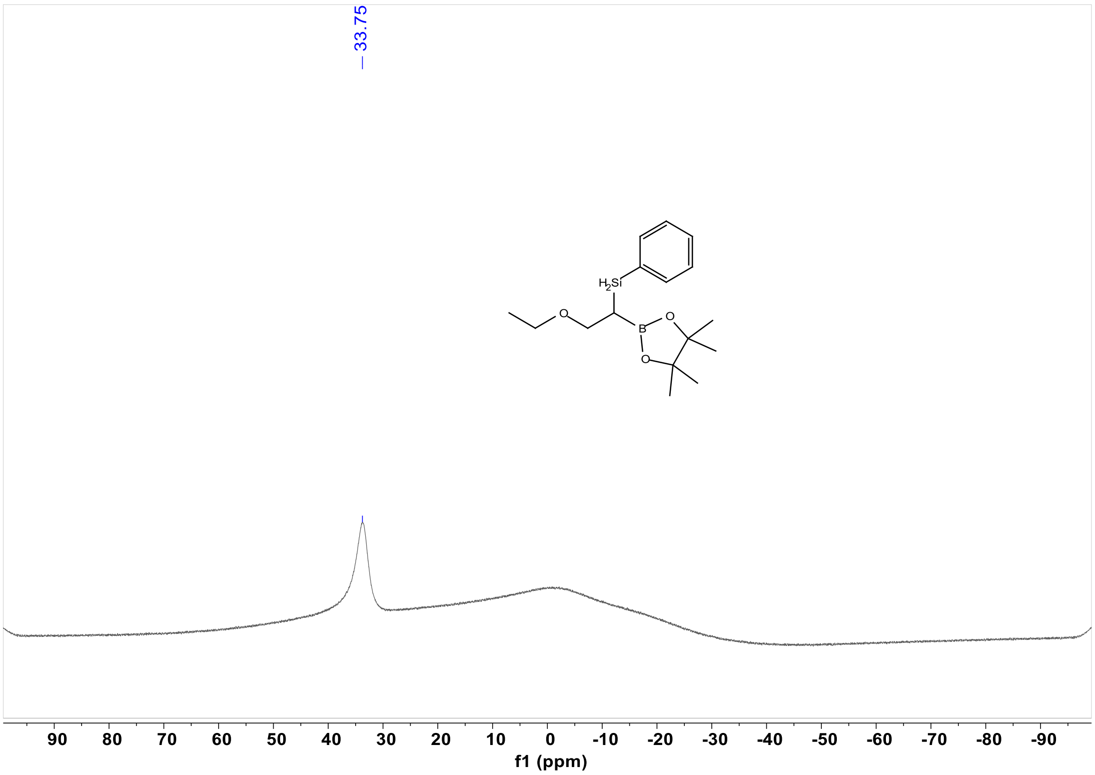

Supplementary Figure 103. ^{11}B NMR spectra for **31**

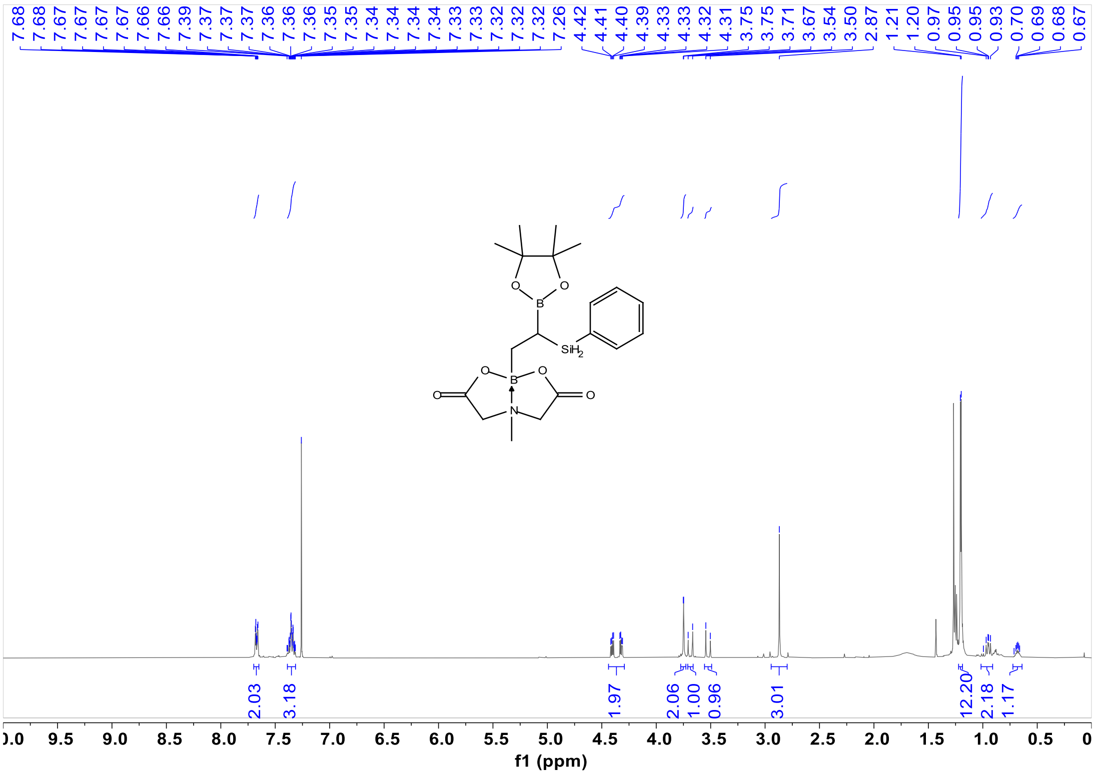

Supplementary Figure 104. ^{1}H NMR spectra for **32**

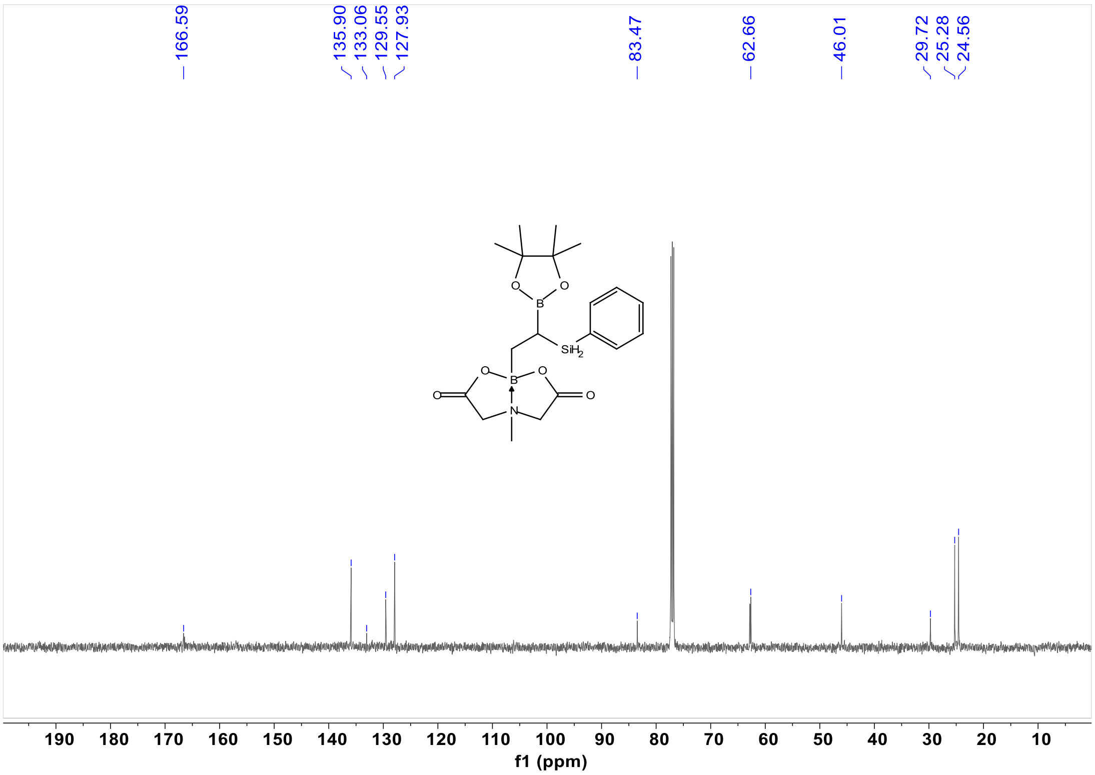

Supplementary Figure 105. ^{13}C NMR spectra for **32**

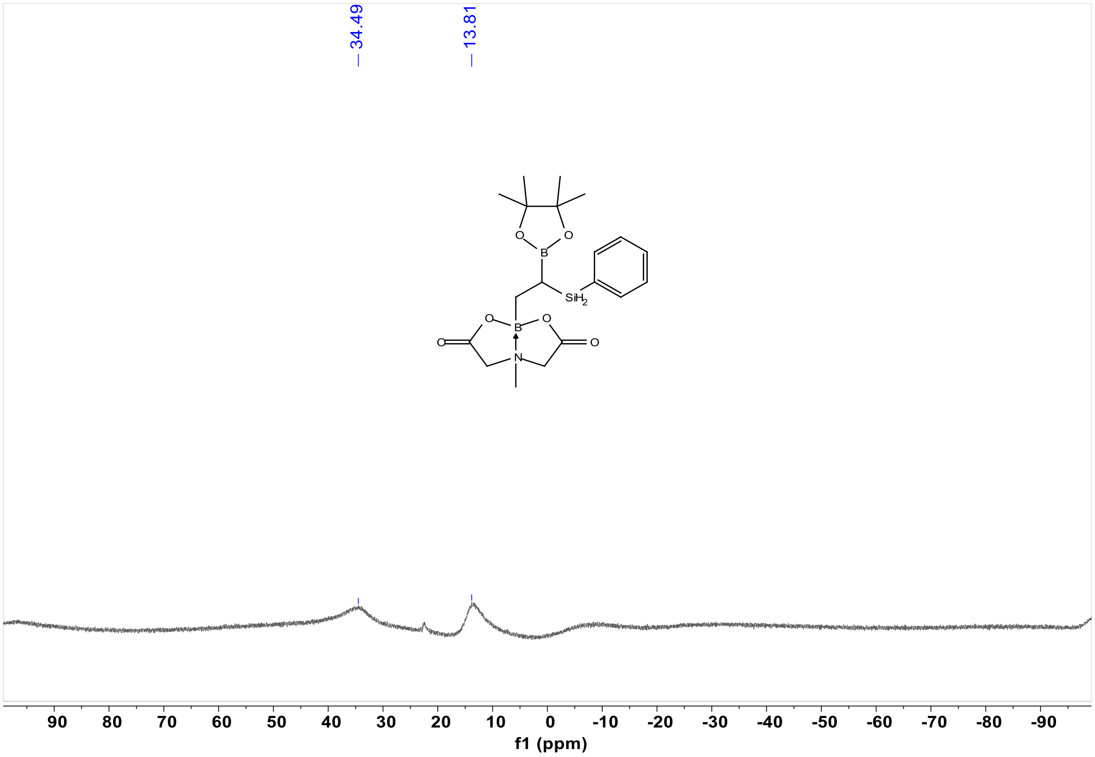

Supplementary Figure 106. ^{11}B NMR spectra for **32**

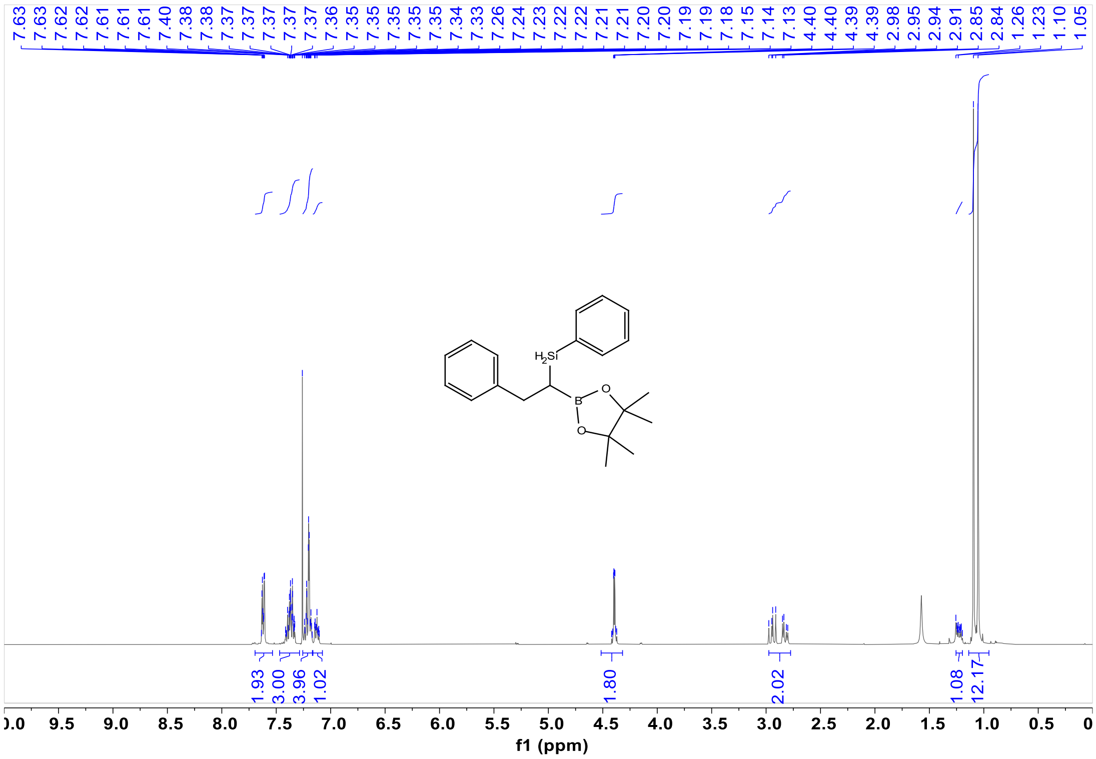

Supplementary Figure 107. ^{1}H NMR spectra for **33**

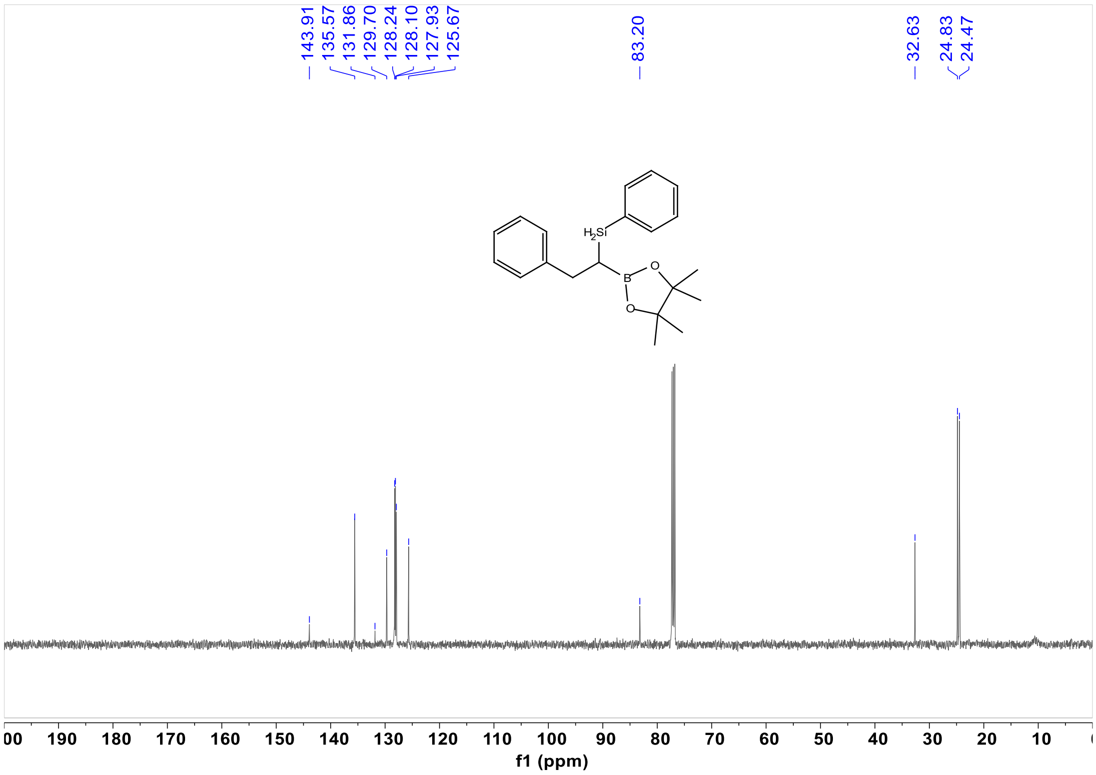

Supplementary Figure 108. ^{13}C NMR spectra for **33**

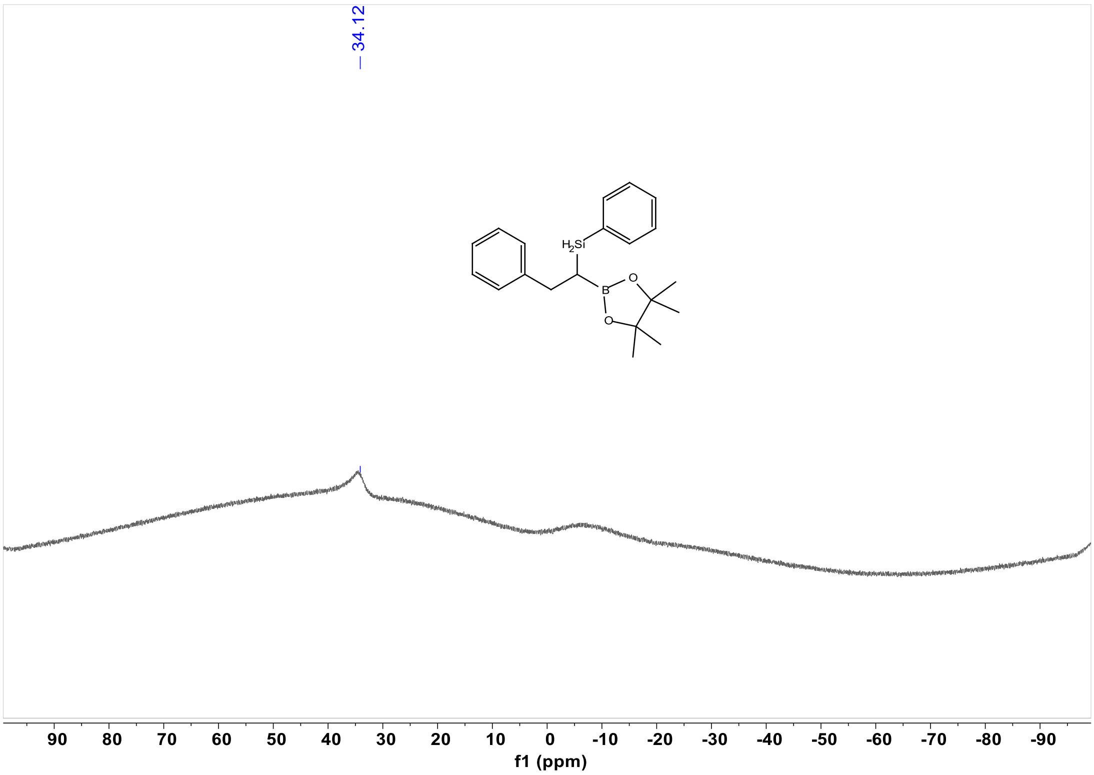

Supplementary Figure 109. ^{11}B NMR spectra for **33**

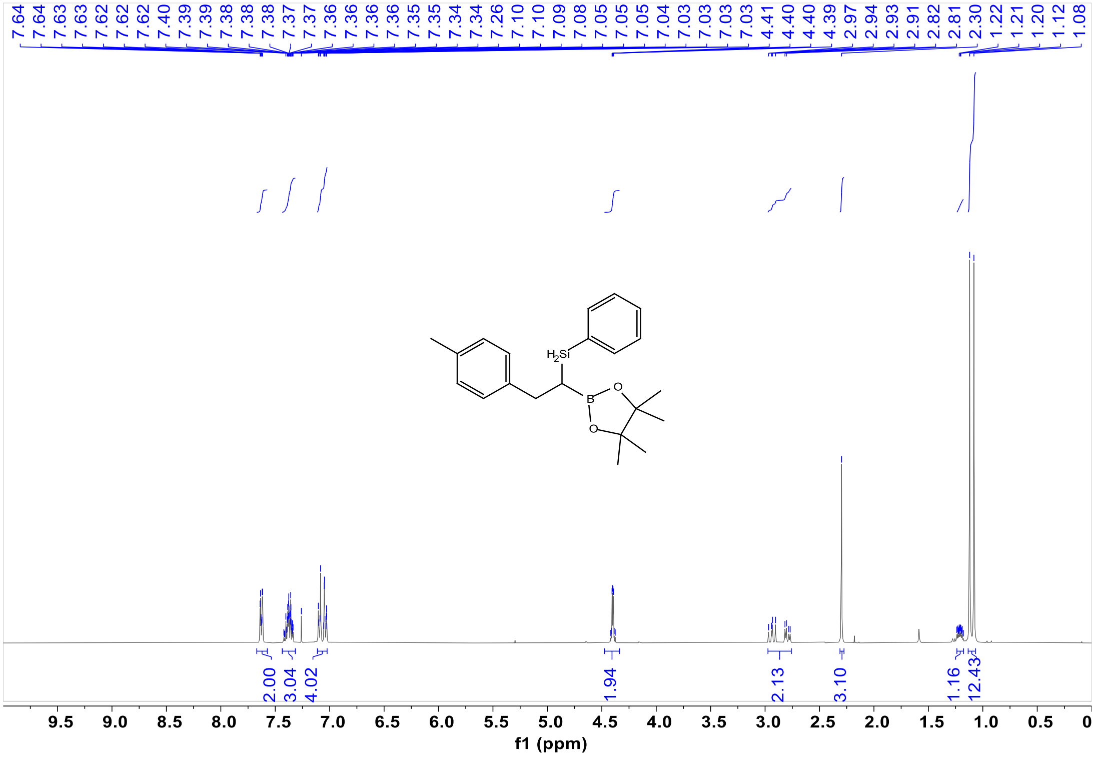

Supplementary Figure 110. ^{1}H NMR spectra for **34**

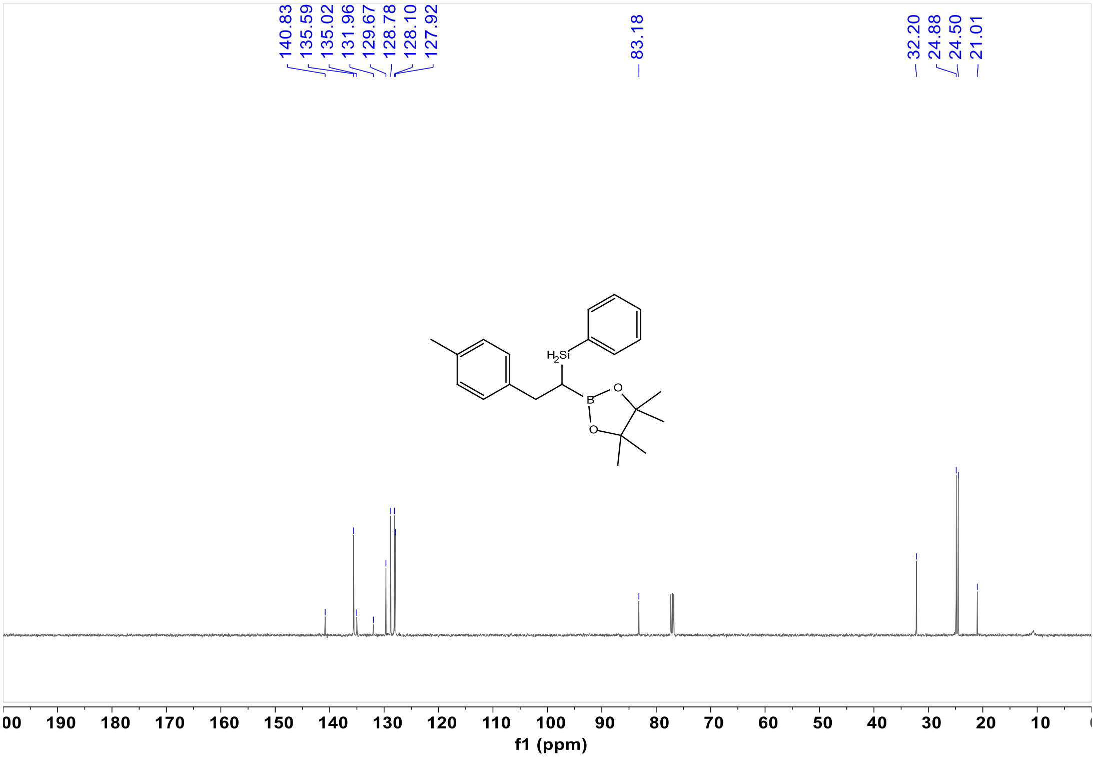

Supplementary Figure 111. ^{13}C NMR spectra for **34**

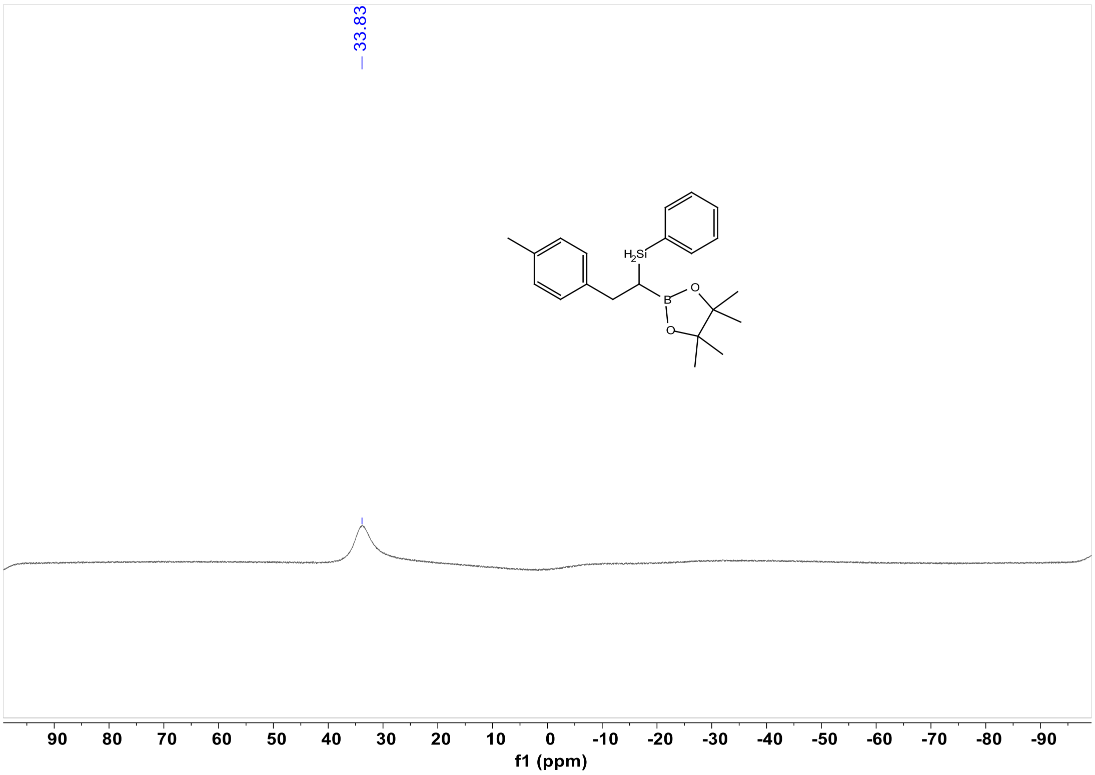

Supplementary Figure 112. ^{11}B NMR spectra for **34**

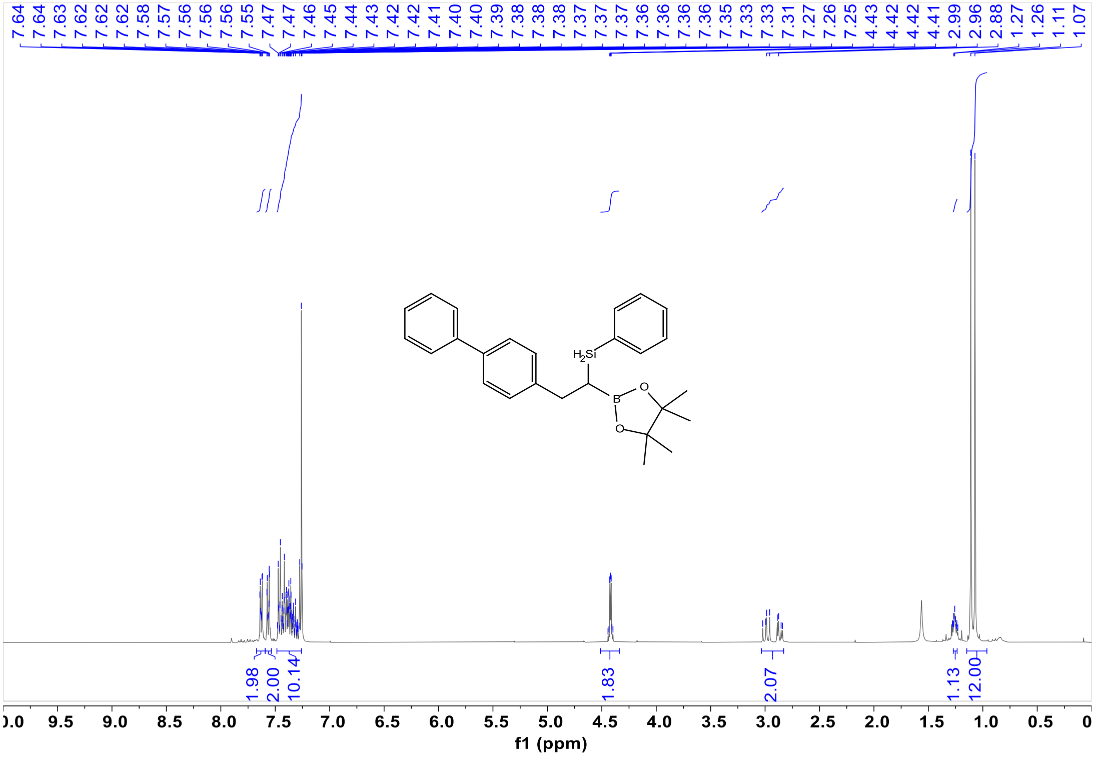

Supplementary Figure 113. ^{1}H NMR spectra for **35**

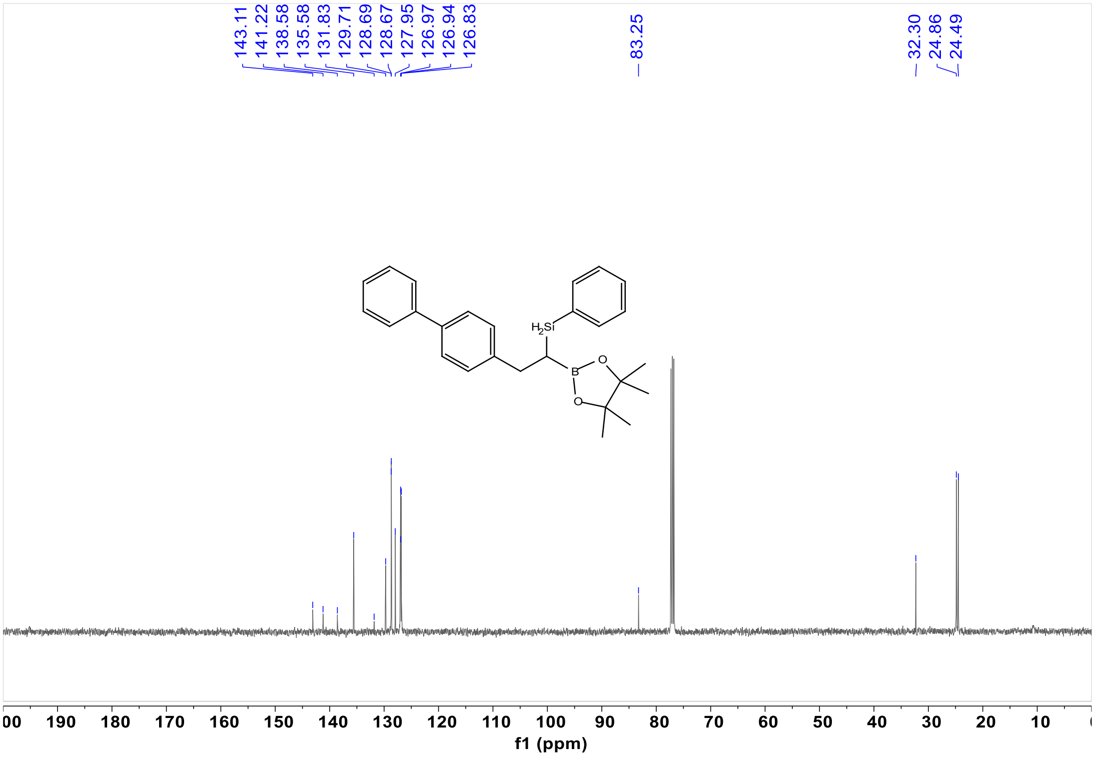

Supplementary Figure 114. ^{13}C NMR spectra for **35**

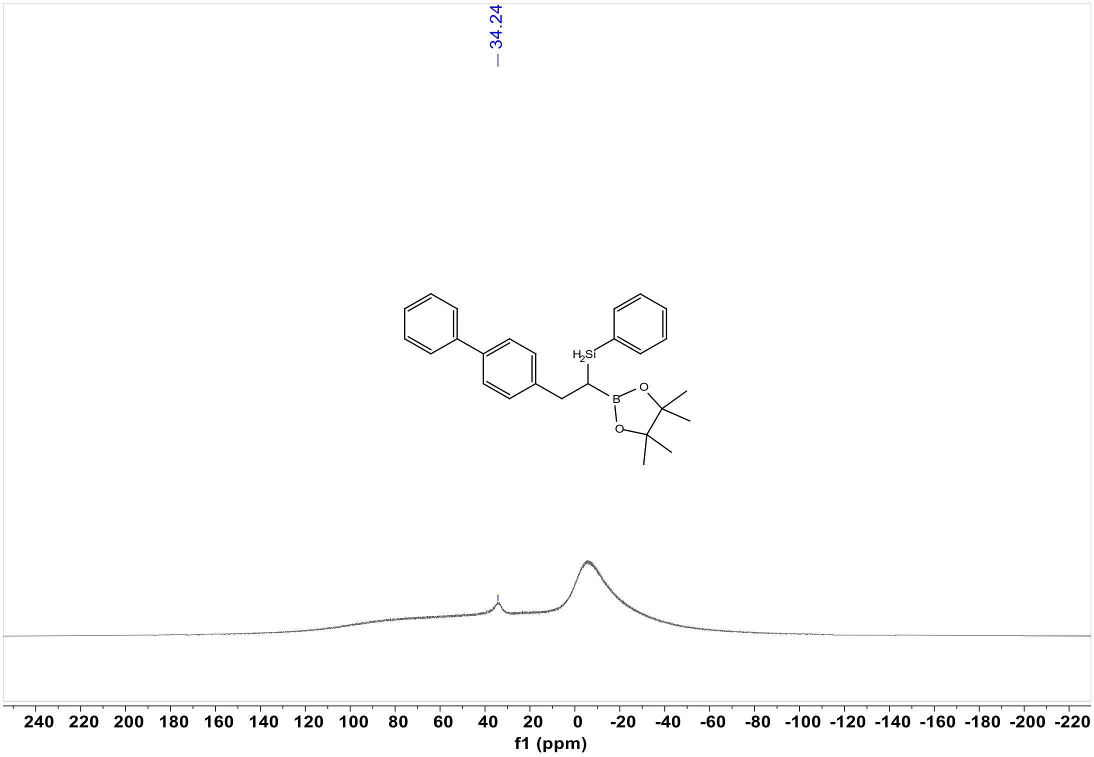

Supplementary Figure 115. ^{11}B NMR spectra for **35**

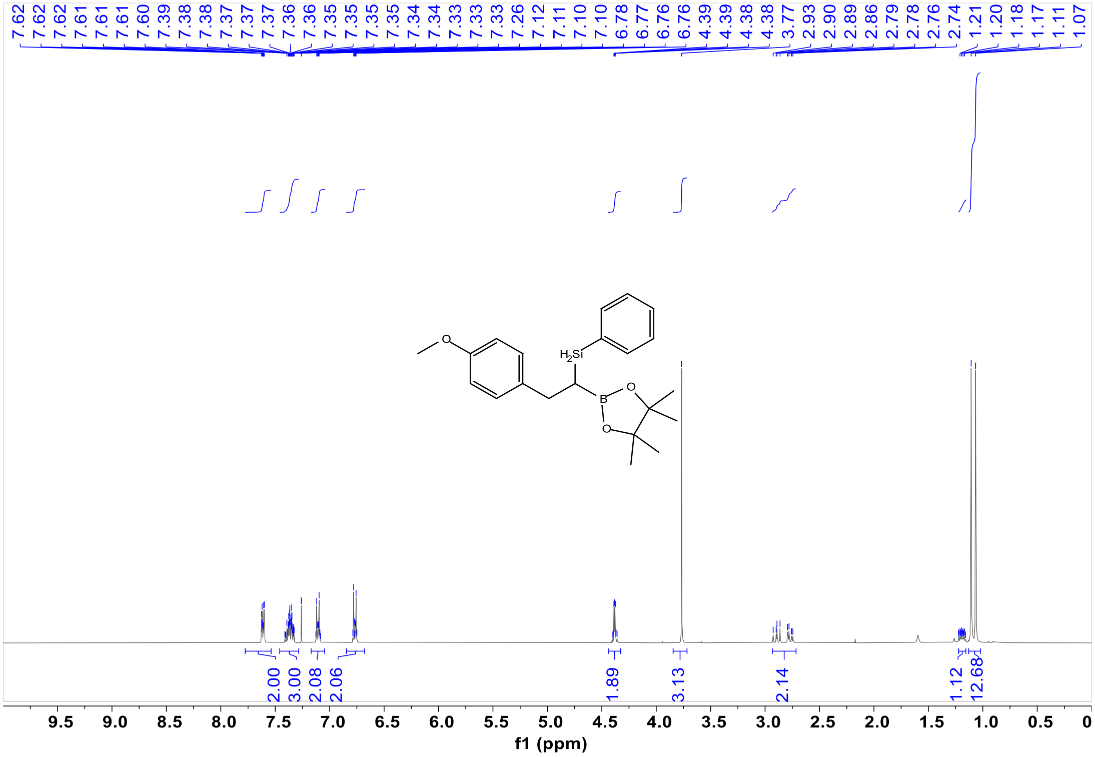

Supplementary Figure 116. ^{1}H NMR spectra for **36**

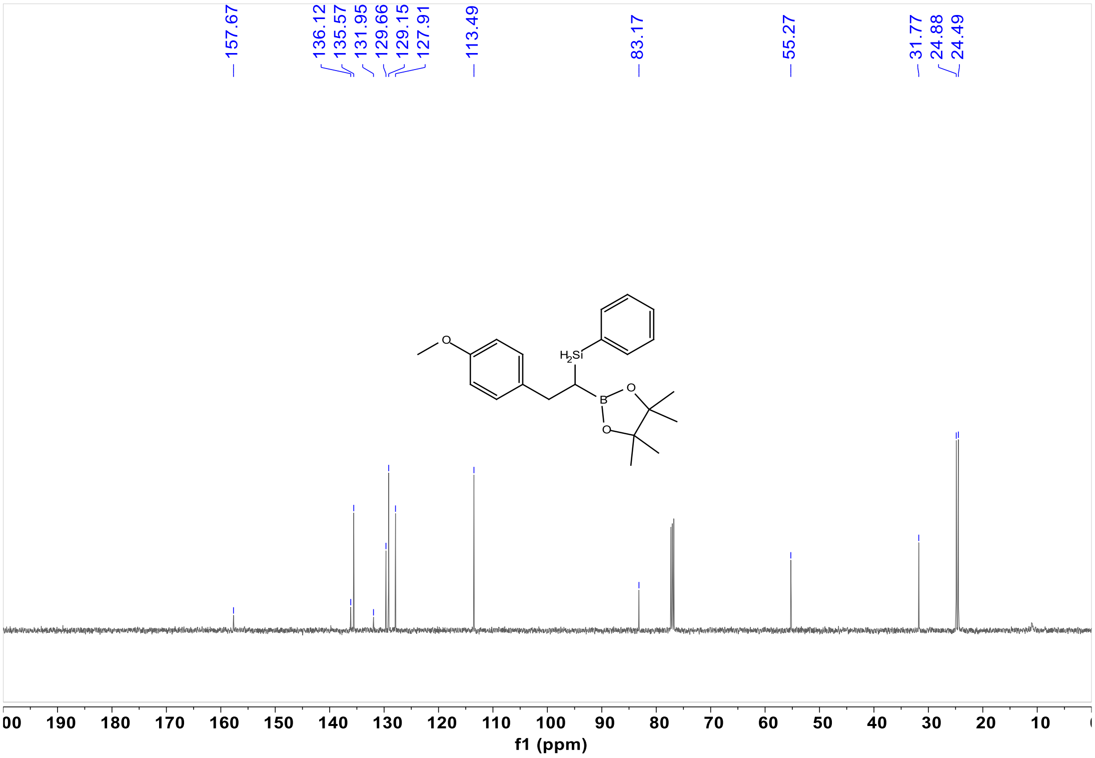

Supplementary Figure 117. ^{13}C NMR spectra for **36**

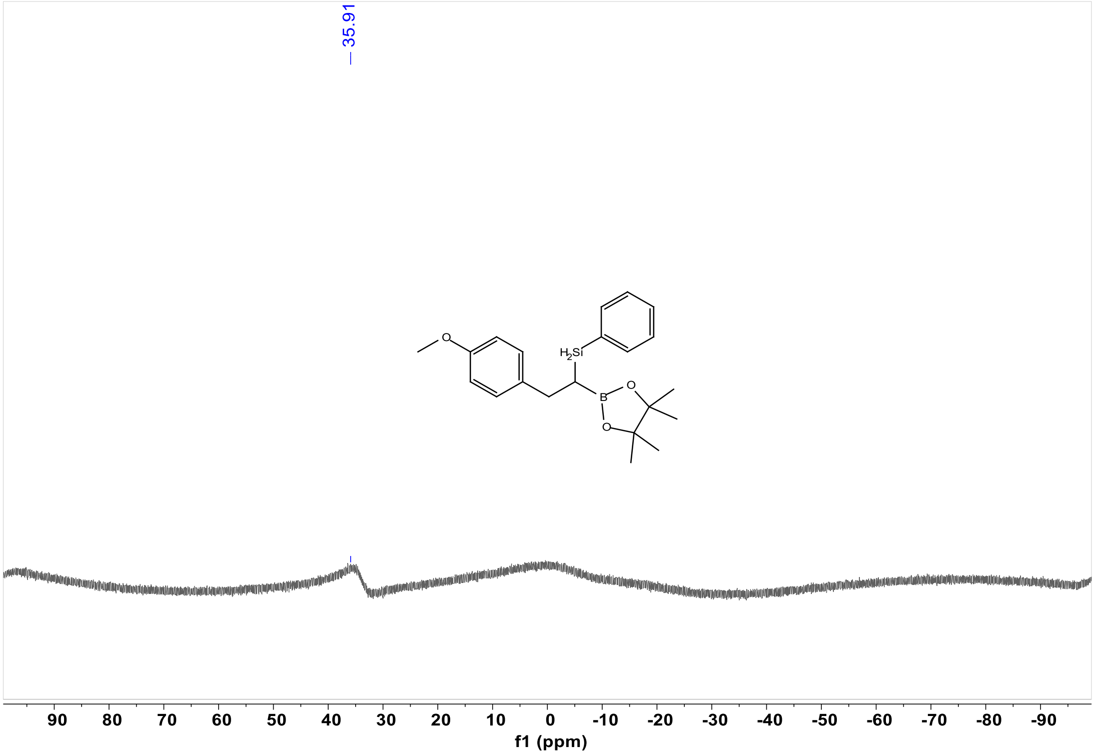

Supplementary Figure 118. ^{11}B NMR spectra for **36**

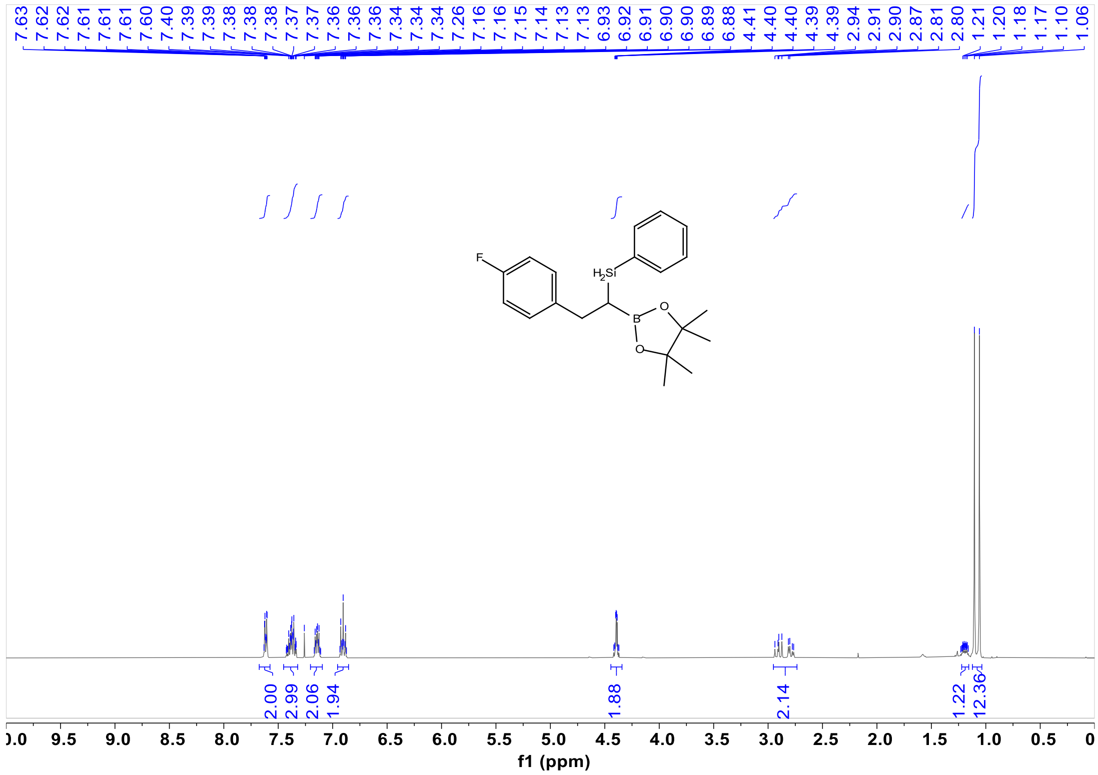

Supplementary Figure 119. ^{1}H NMR spectra for **37**

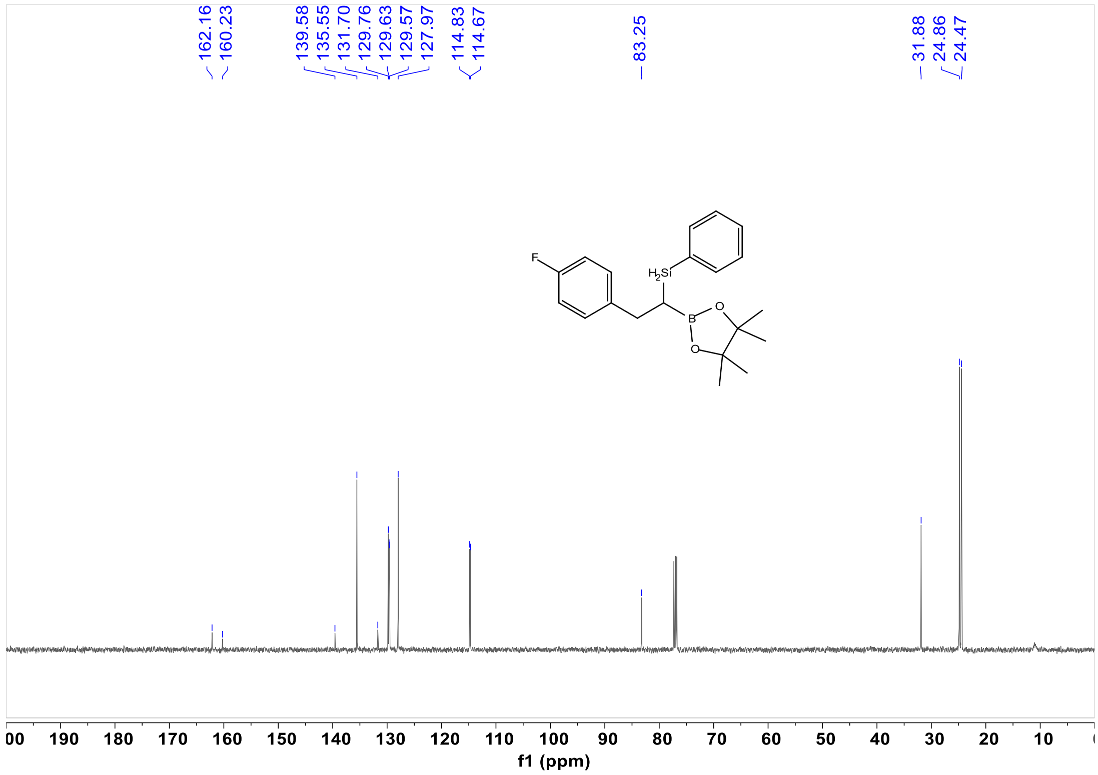

Supplementary Figure 120. ^{13}C NMR spectra for **37**

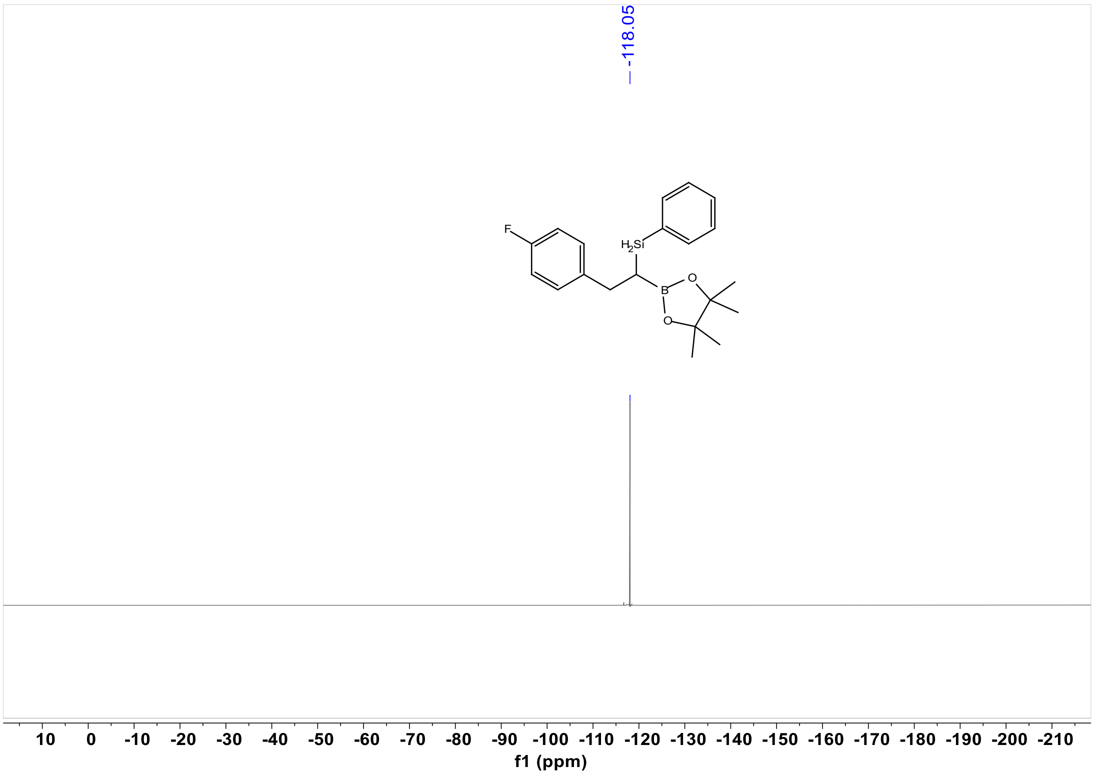

Supplementary Figure 121. ^{19}F NMR spectra for **37**

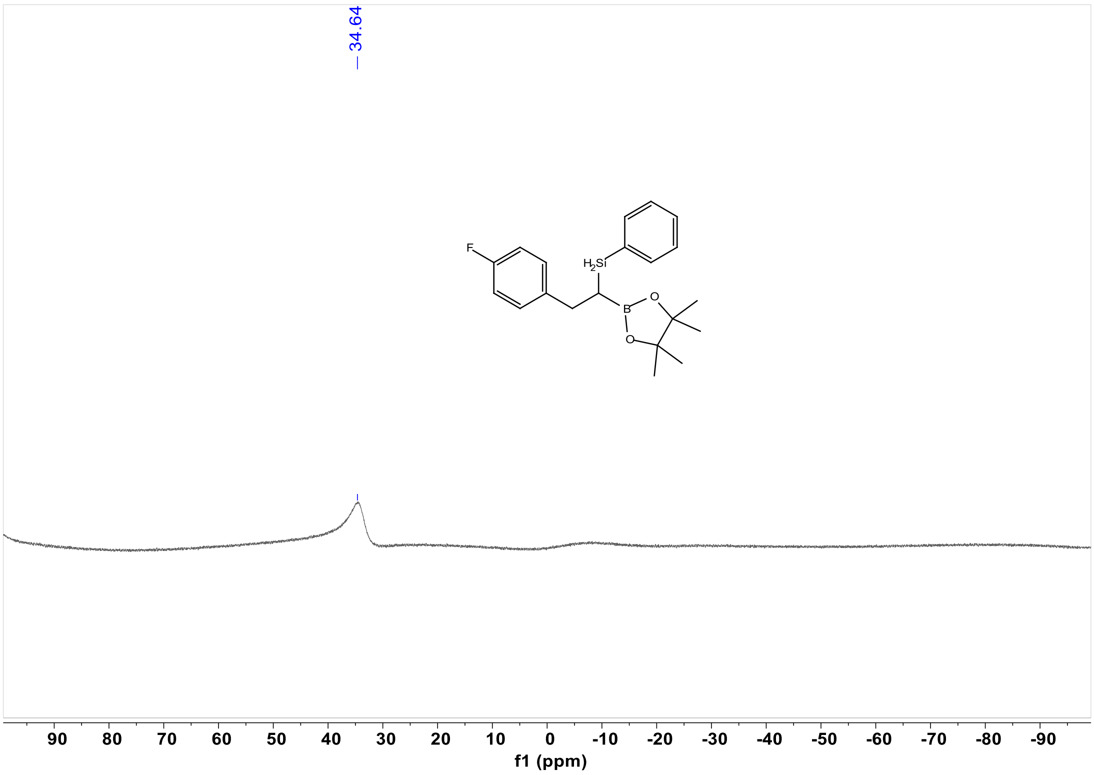

Supplementary Figure 122. ^{11}B NMR spectra for **37**

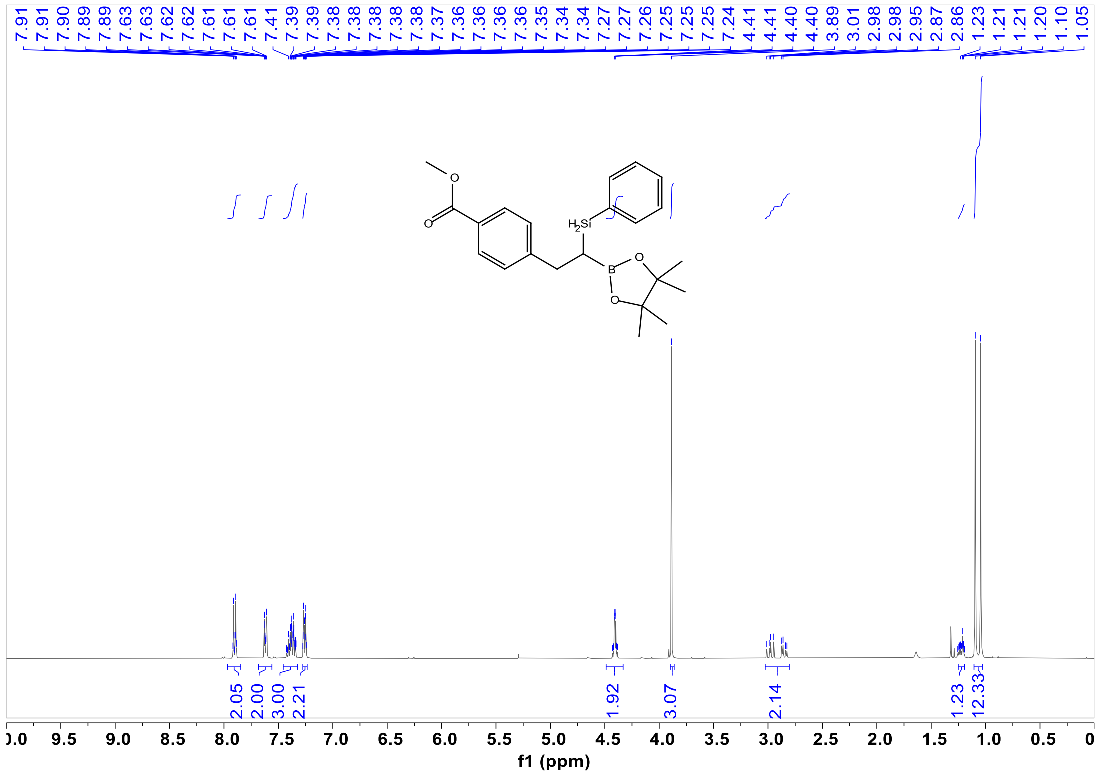

Supplementary Figure 123. ^{1}H NMR spectra for **38**

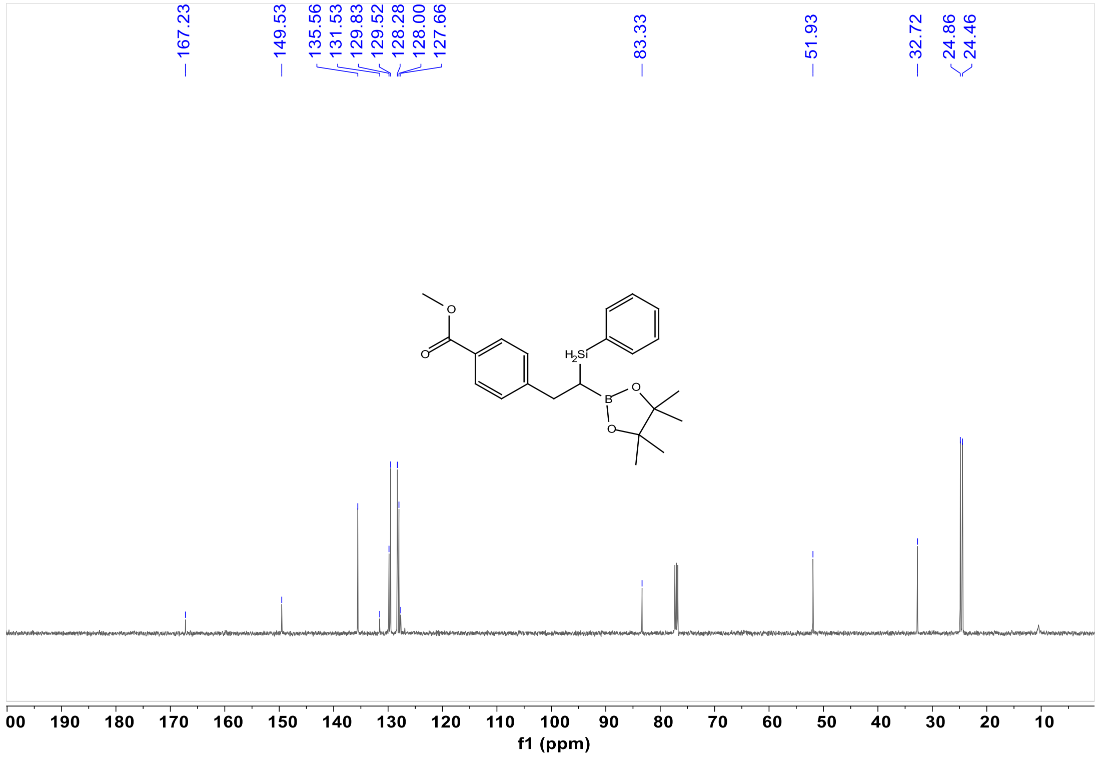

Supplementary Figure 124. ^{13}C NMR spectra for **38**

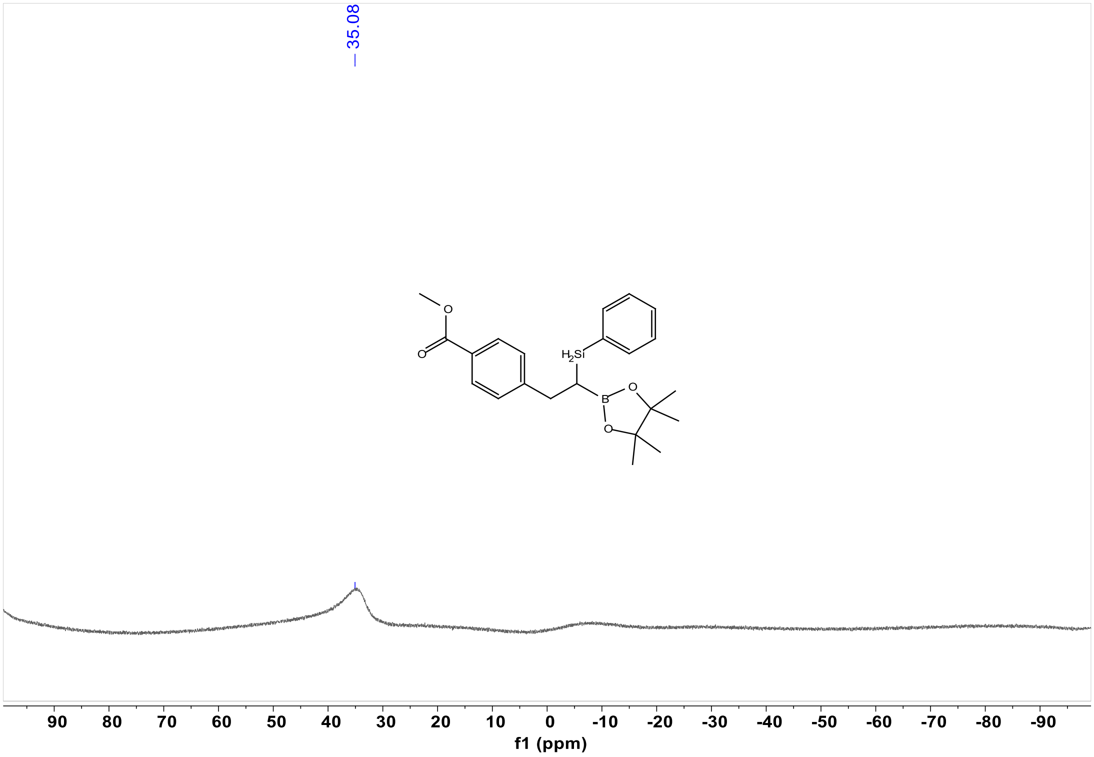

Supplementary Figure 125. ^{11}B NMR spectra for **38**

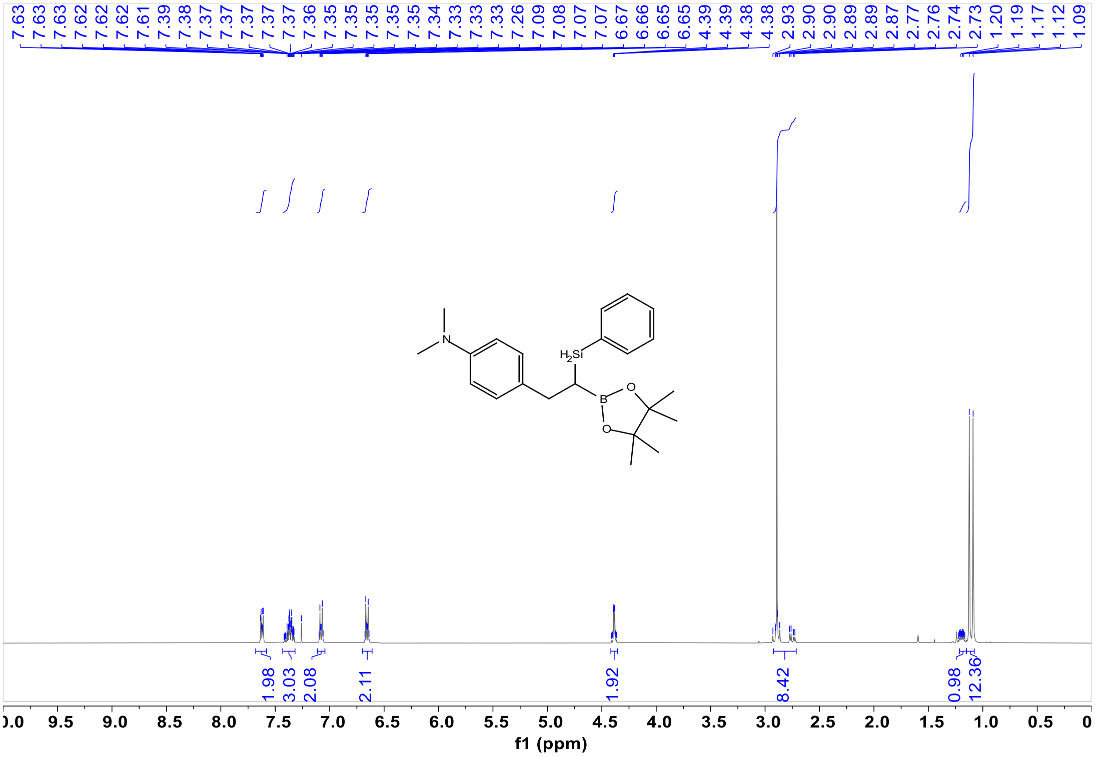

Supplementary Figure 126. ^{1}H NMR spectra for **39**

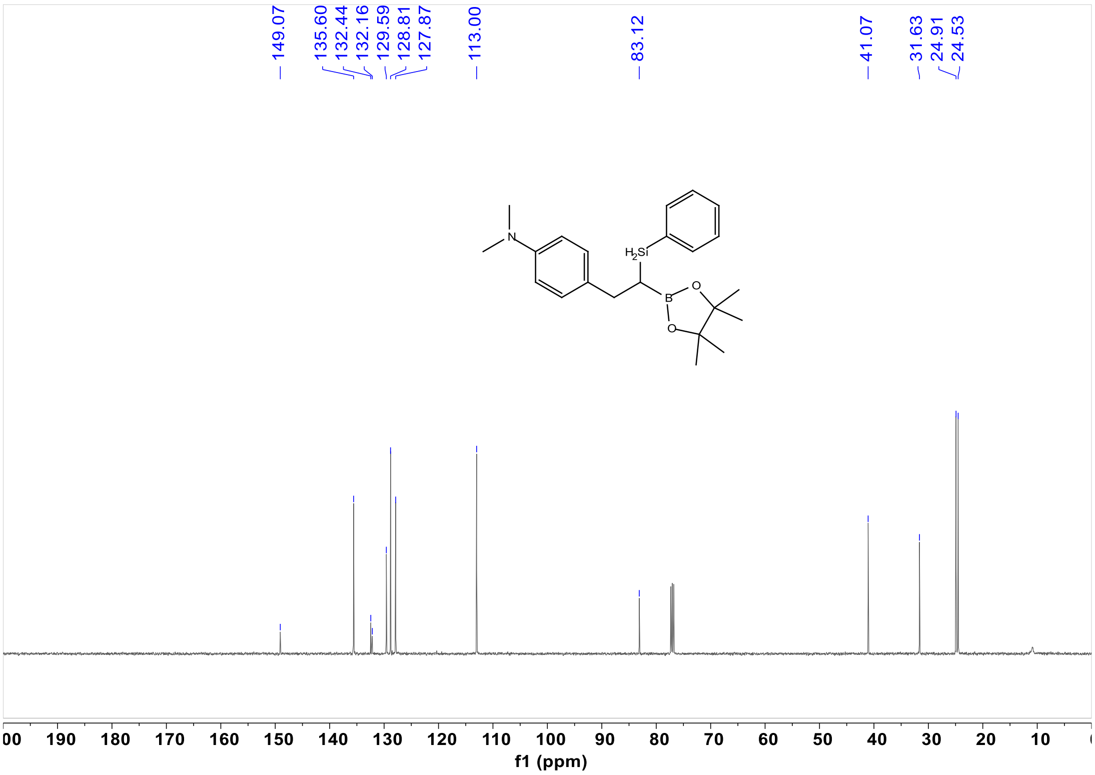

Supplementary Figure 127. ^{13}C NMR spectra for **39**

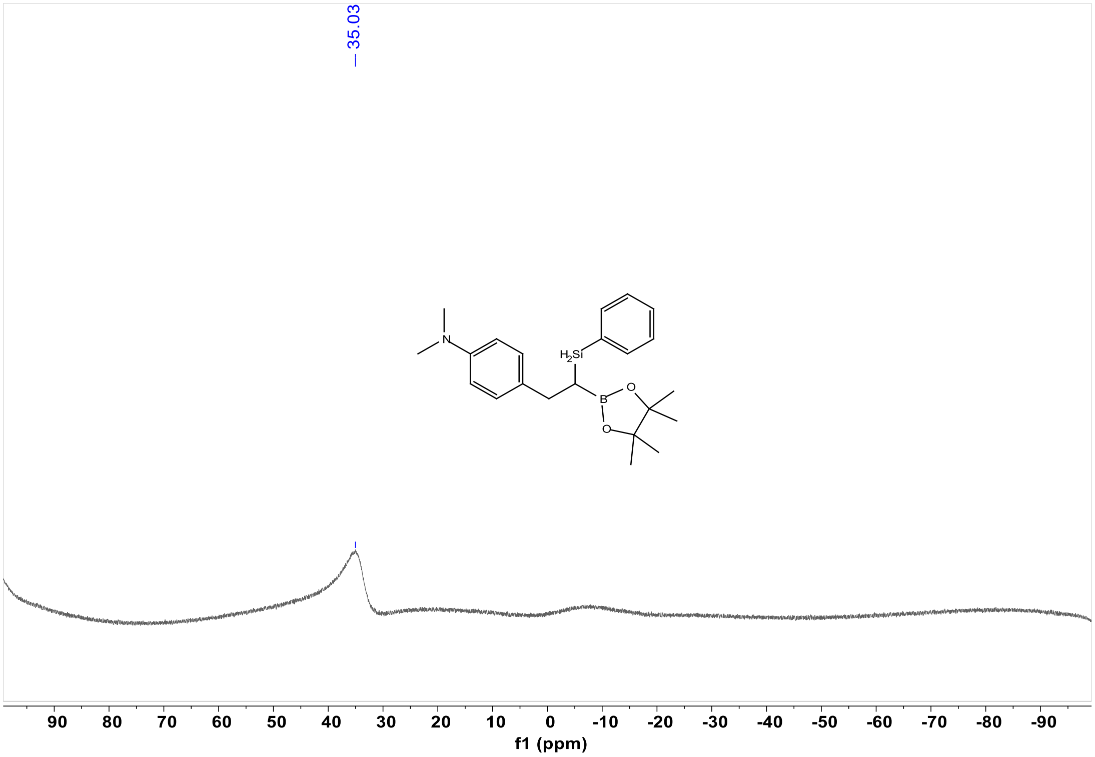

Supplementary Figure 128. ^{11}B NMR spectra for **39**

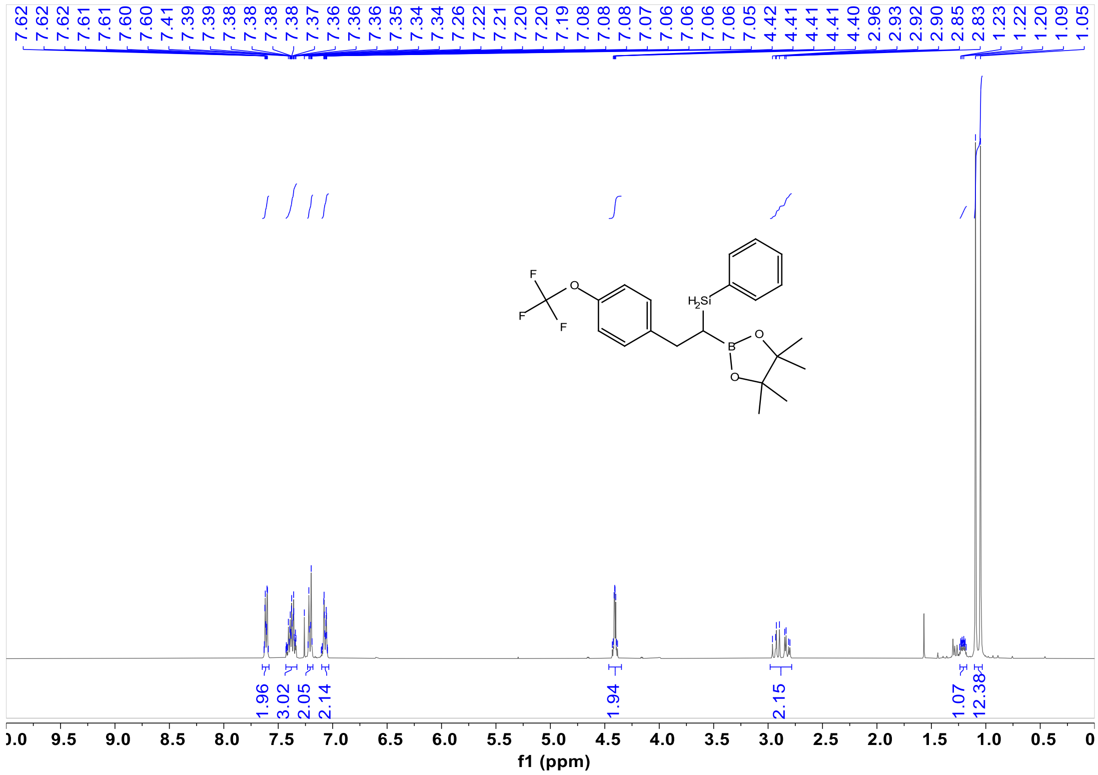

Supplementary Figure 129. ^{1}H NMR spectra for **40**

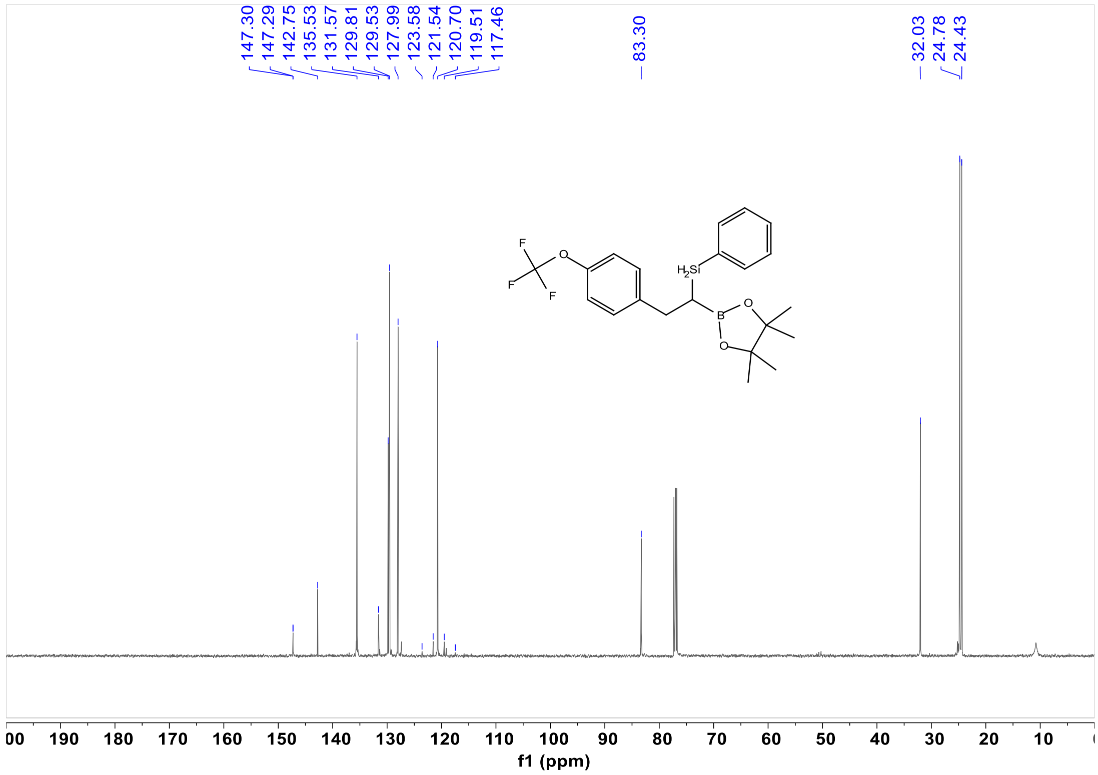

Supplementary Figure 130. ^{13}C NMR spectra for **40**

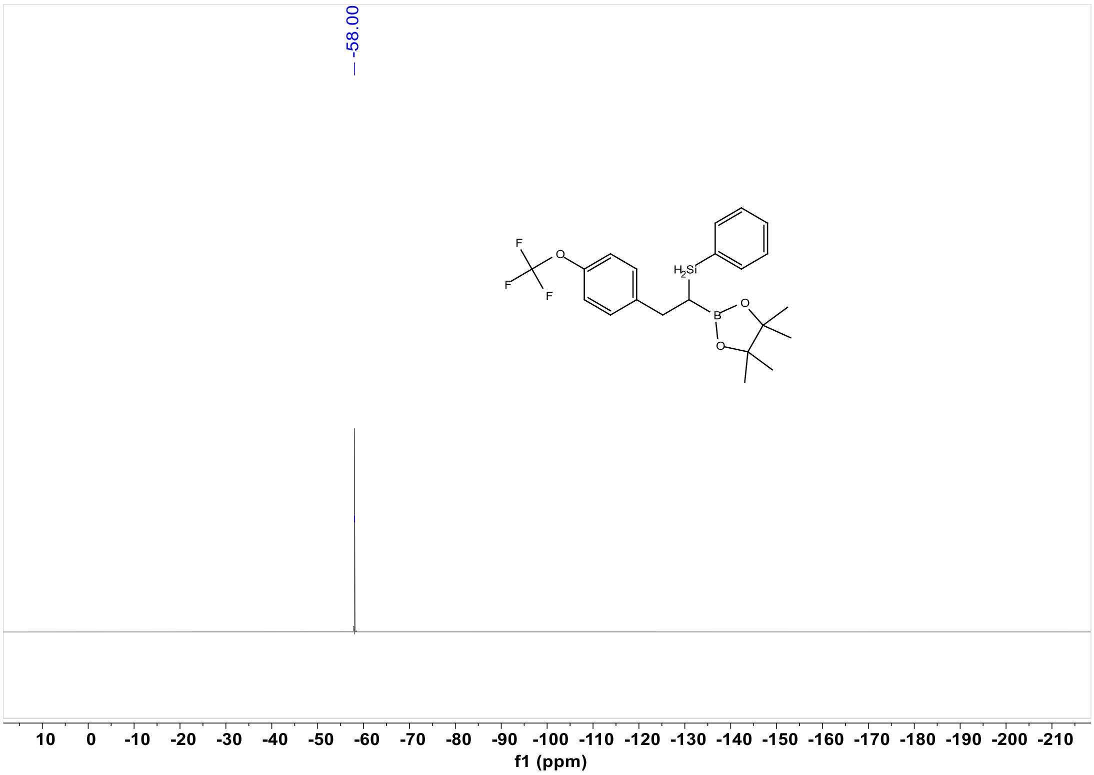

Supplementary Figure 131. ^{19}F NMR spectra for **40**

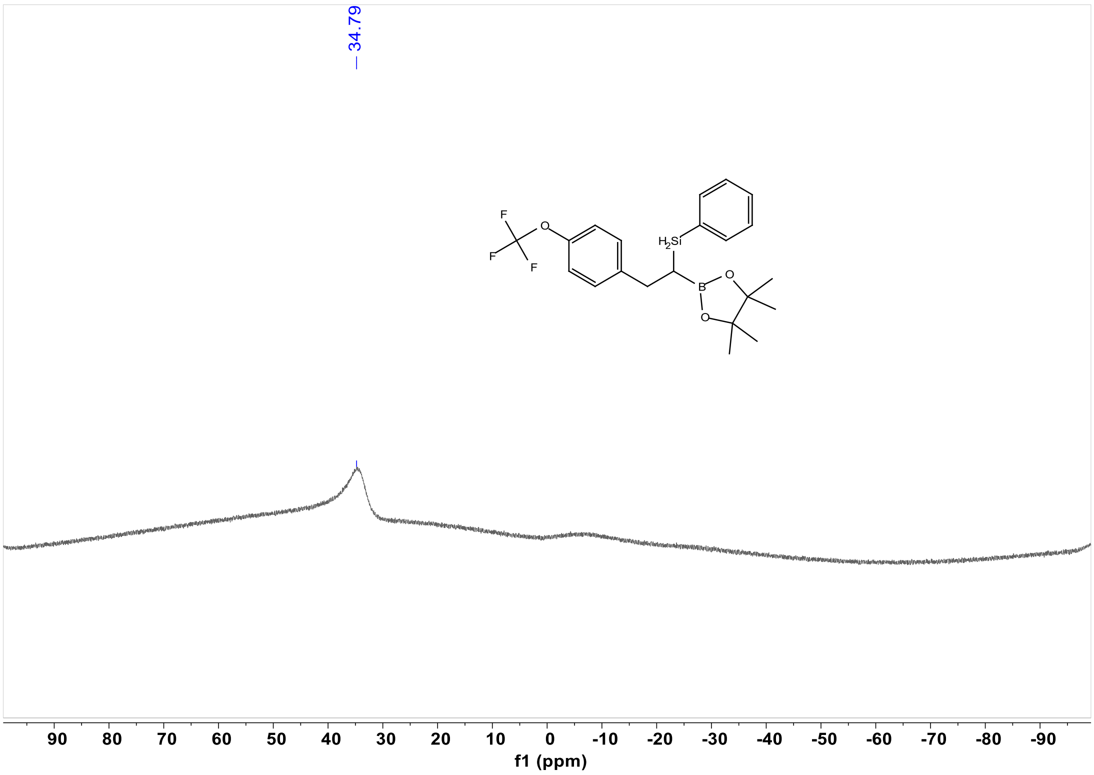

Supplementary Figure 132. ^{11}B NMR spectra for **40**

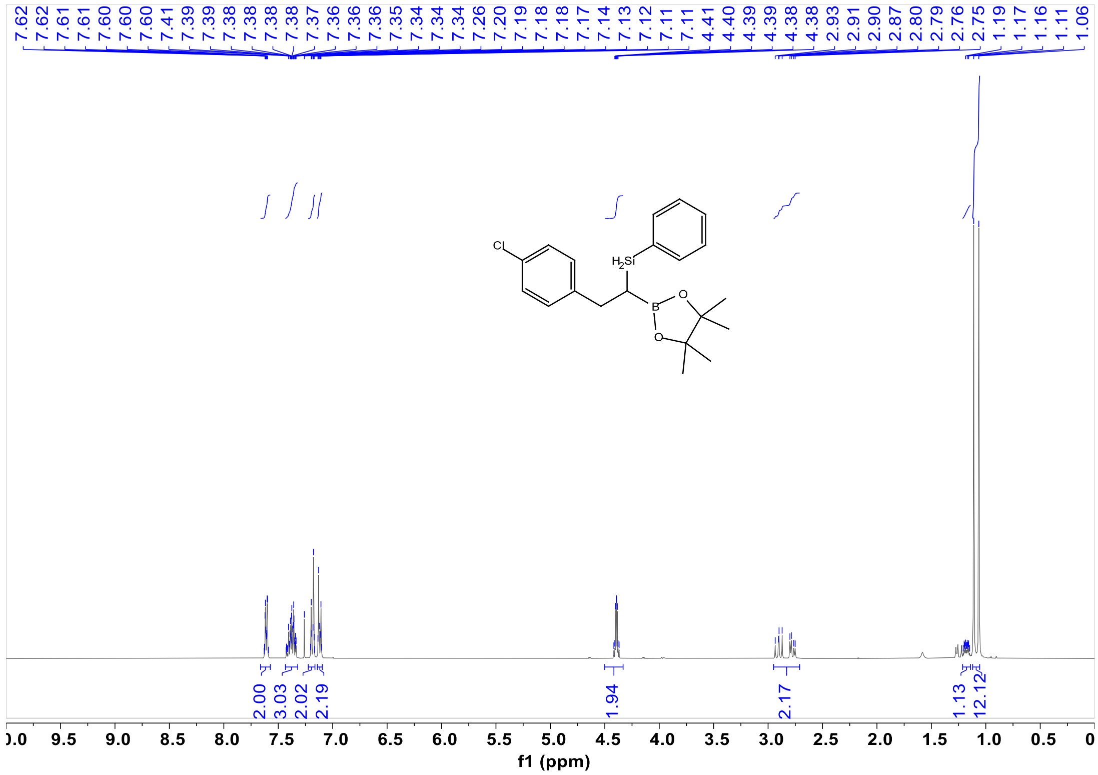

Supplementary Figure 133. ^{1}H NMR spectra for **41**

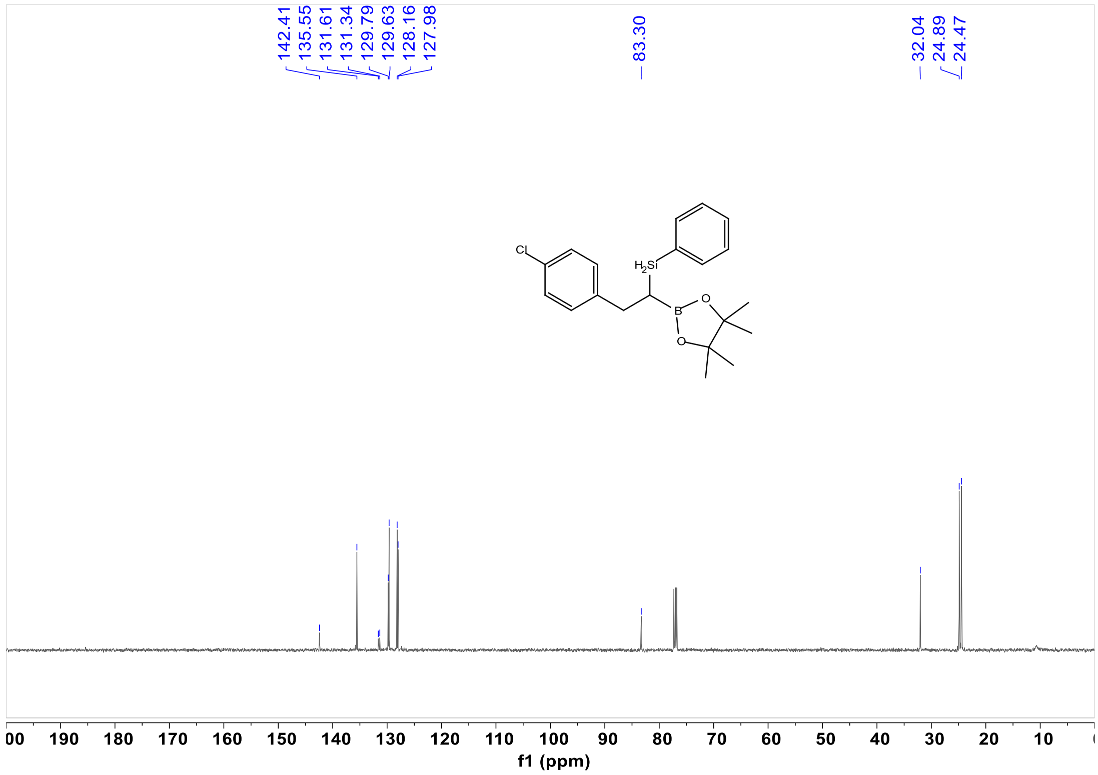

Supplementary Figure 134. ^{13}C NMR spectra for **41**

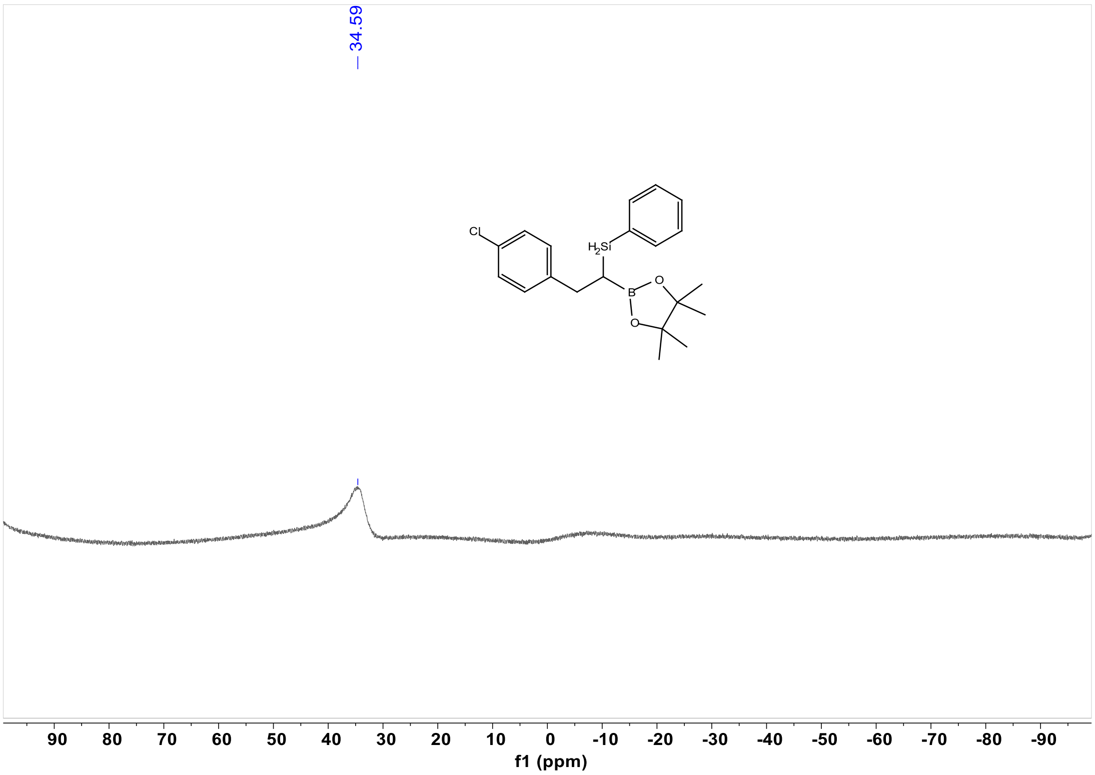

Supplementary Figure 135. ^{11}B NMR spectra for **41**

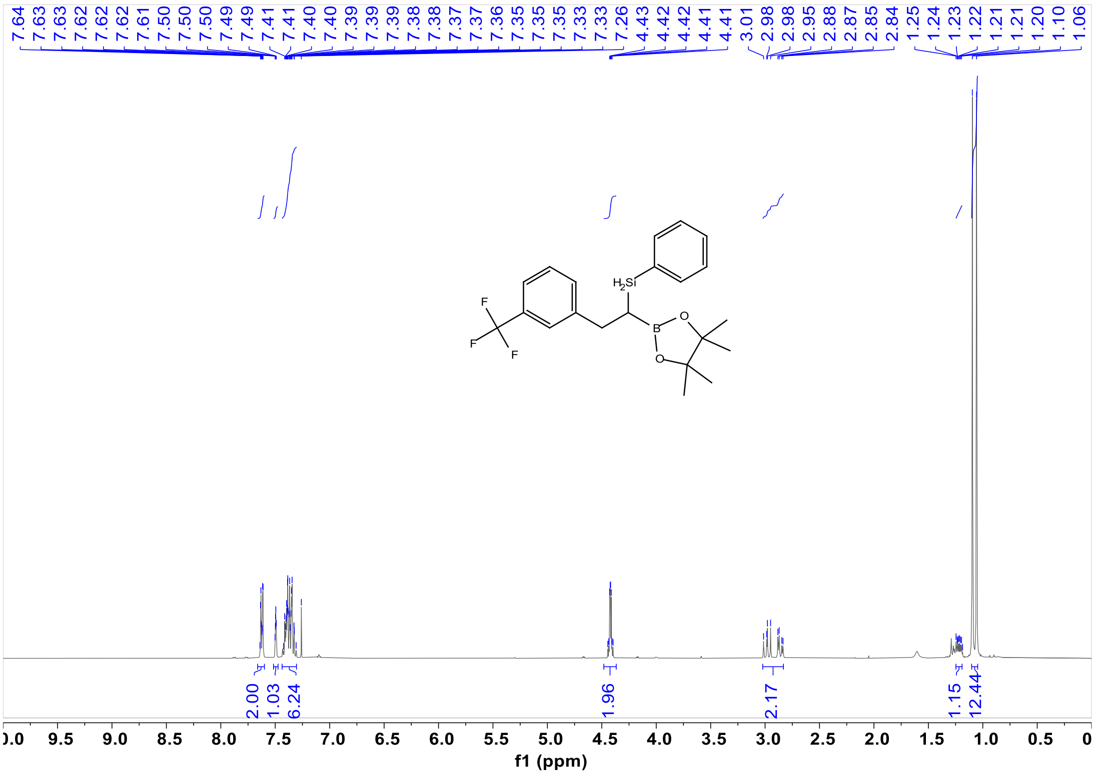

Supplementary Figure 136. ^{1}H NMR spectra for **42**

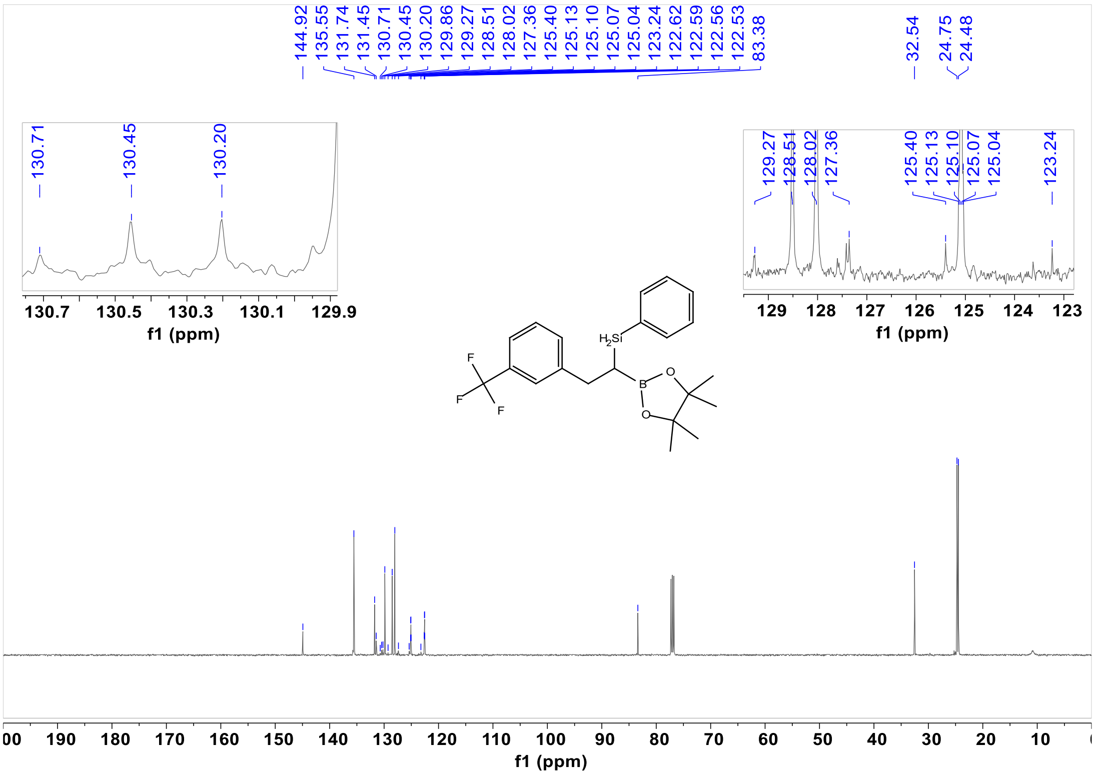

Supplementary Figure 137. ^{13}C NMR spectra for **42**

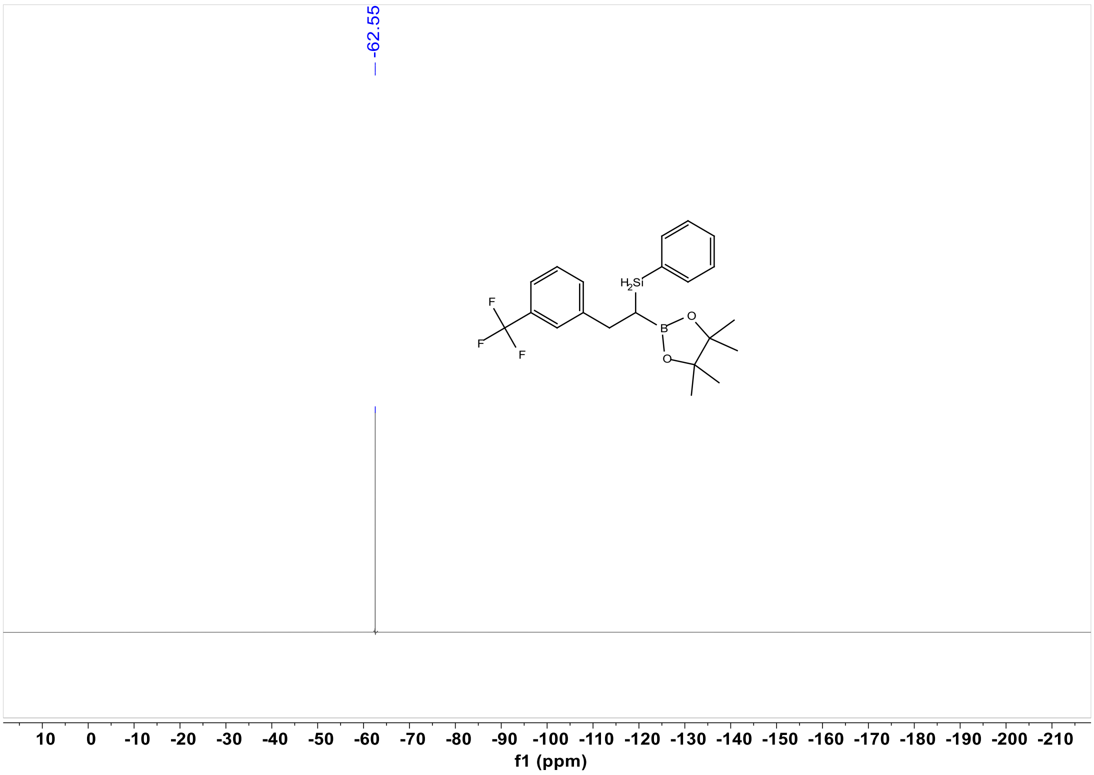

Supplementary Figure 138. ^{19}F NMR spectra for **42**

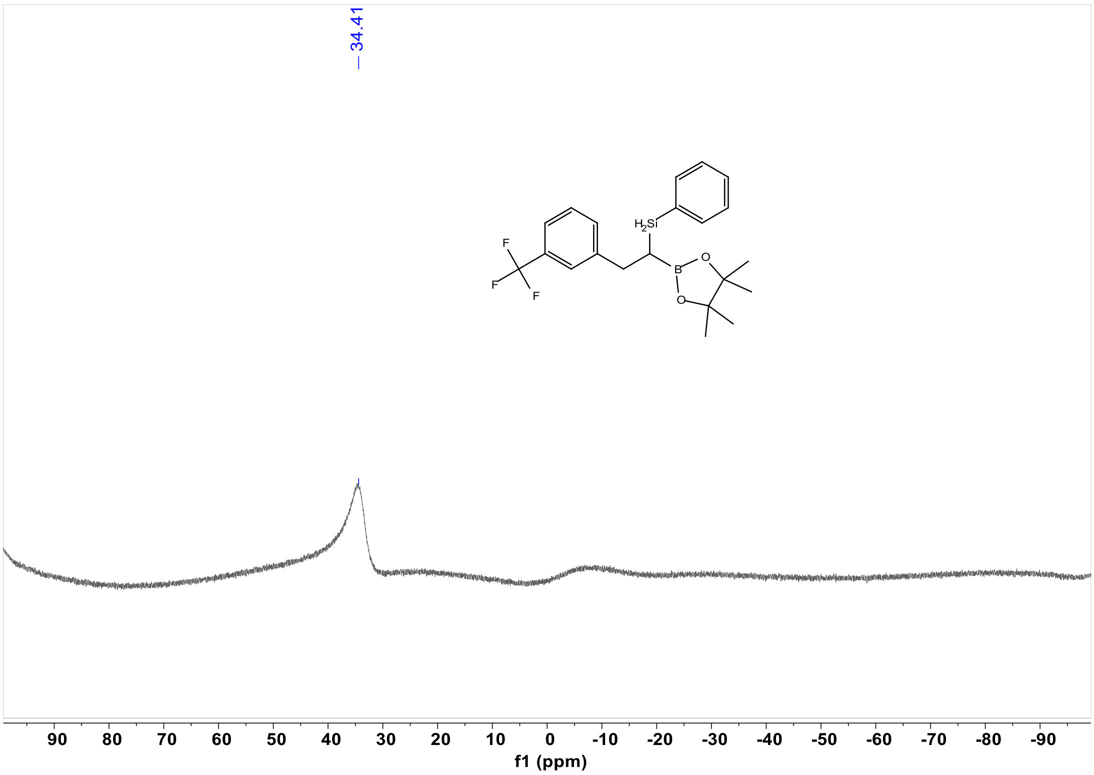

Supplementary Figure 139. ^{11}B NMR spectra for **42**

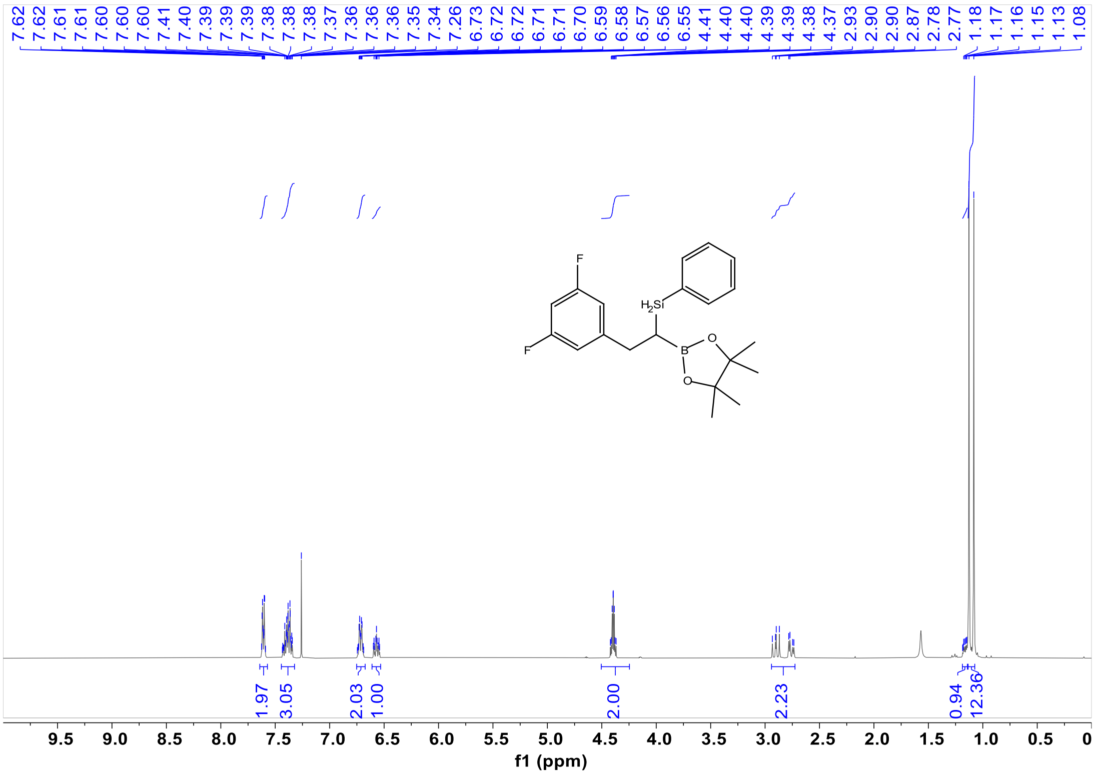

Supplementary Figure 140. ^{1}H NMR spectra for **43**

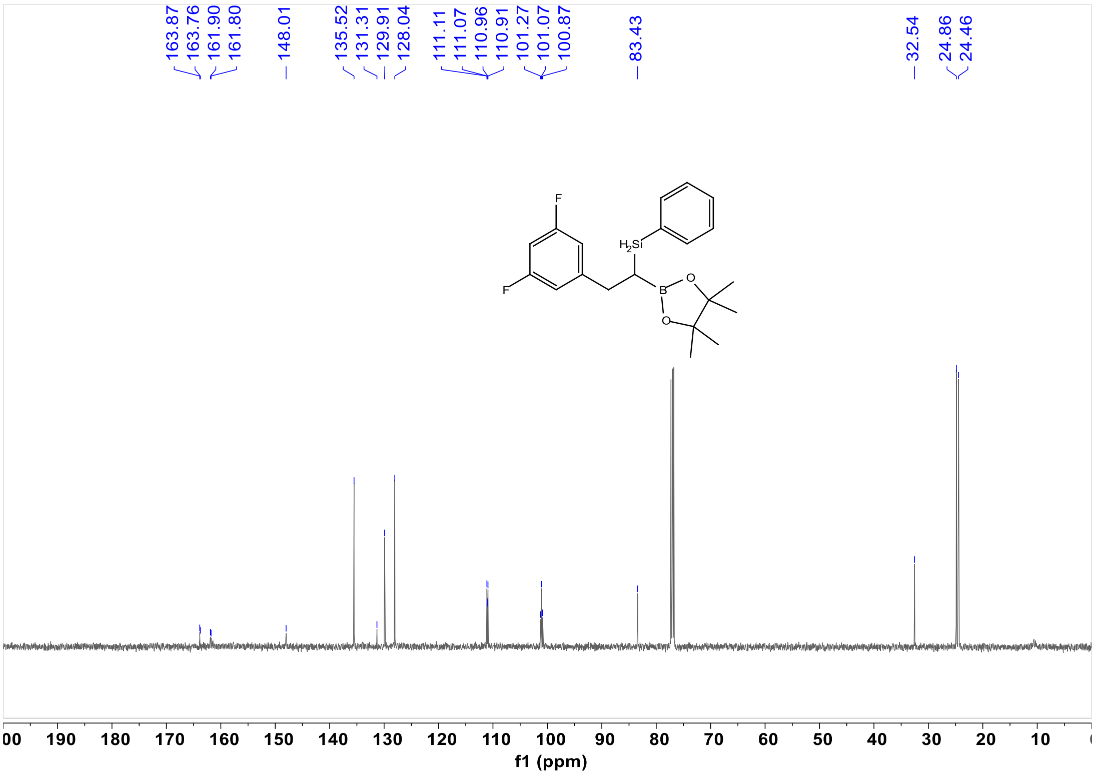

Supplementary Figure 141. ^{13}C NMR spectra for **43**

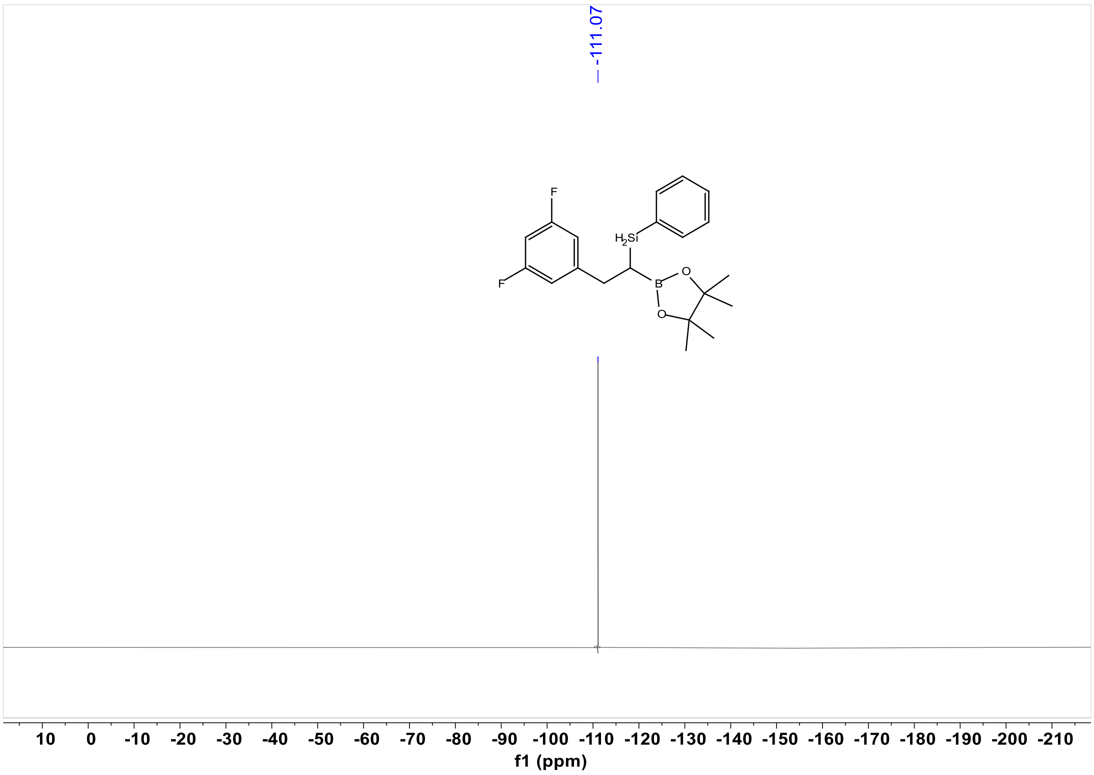

Supplementary Figure 142. ^{19}F NMR spectra for **43**

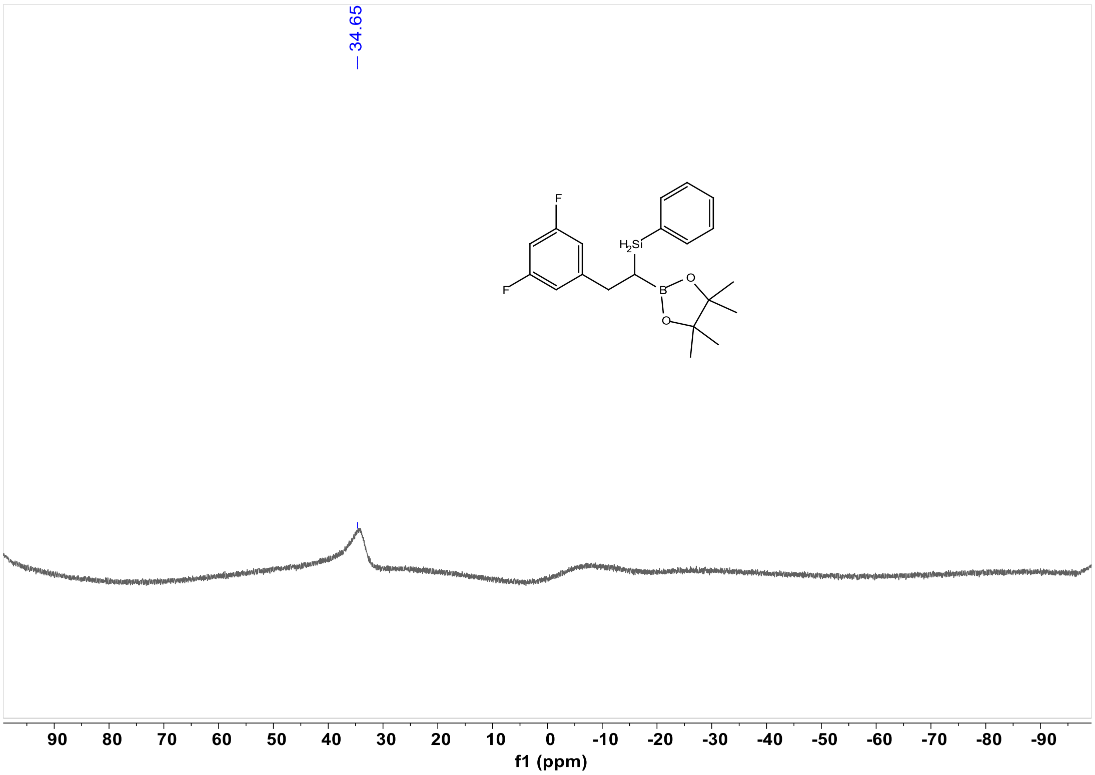

Supplementary Figure 143. ^{11}B NMR spectra for **43**

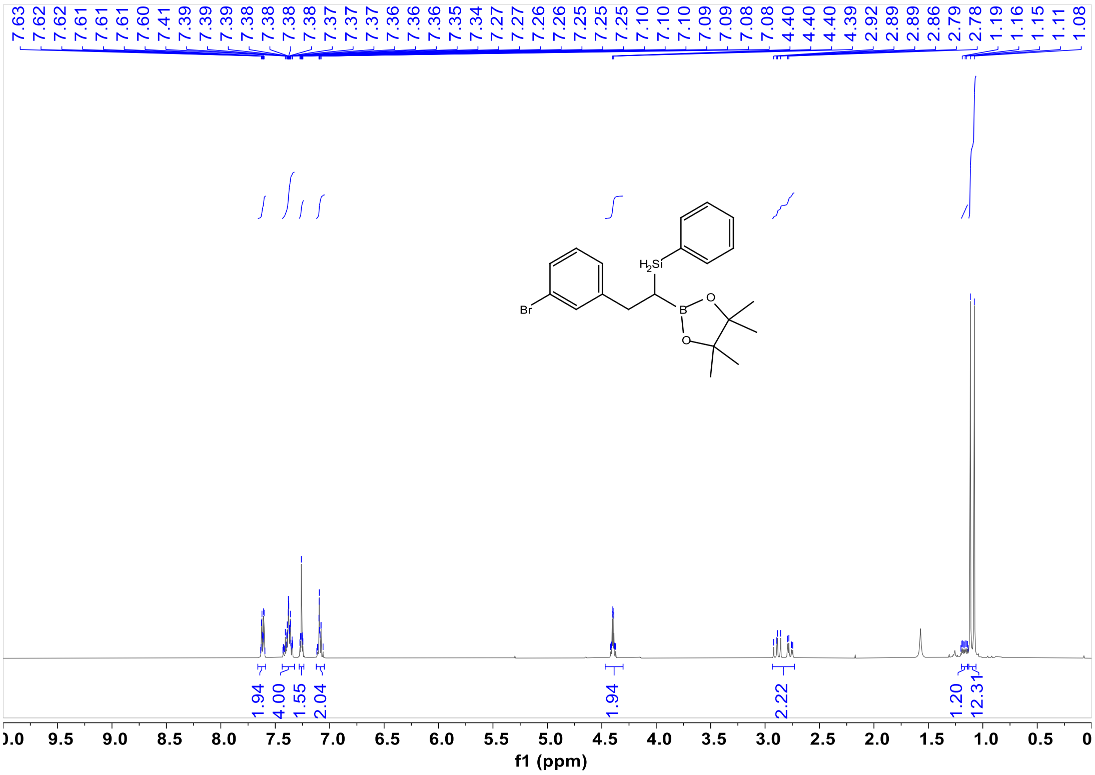

Supplementary Figure 144. ^{1}H NMR spectra for **44**

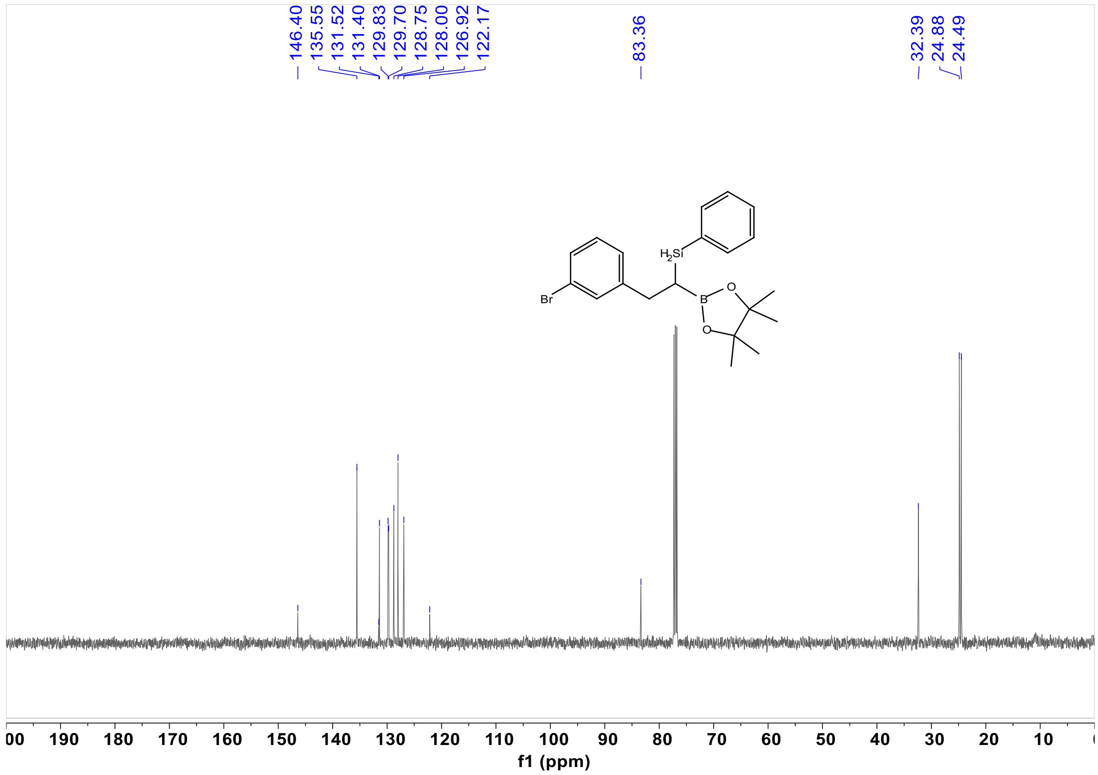

Supplementary Figure 145. ^{13}C NMR spectra for **44**

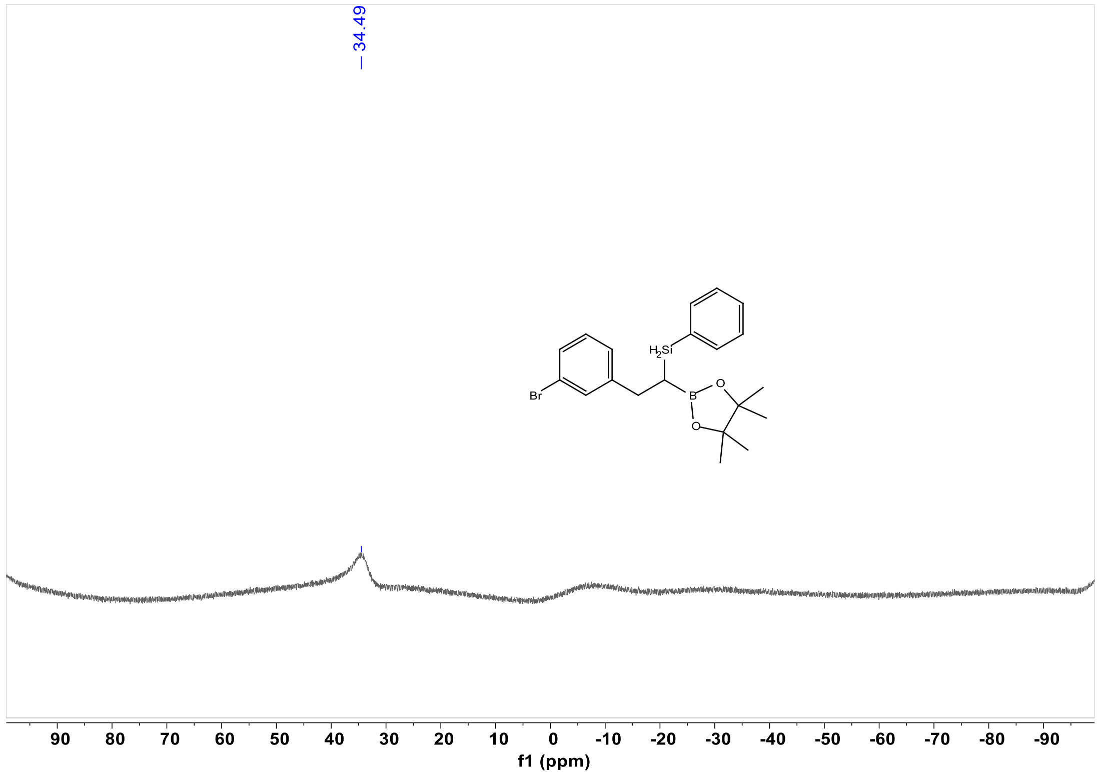

Supplementary Figure 146. ^{11}B NMR spectra for **44**

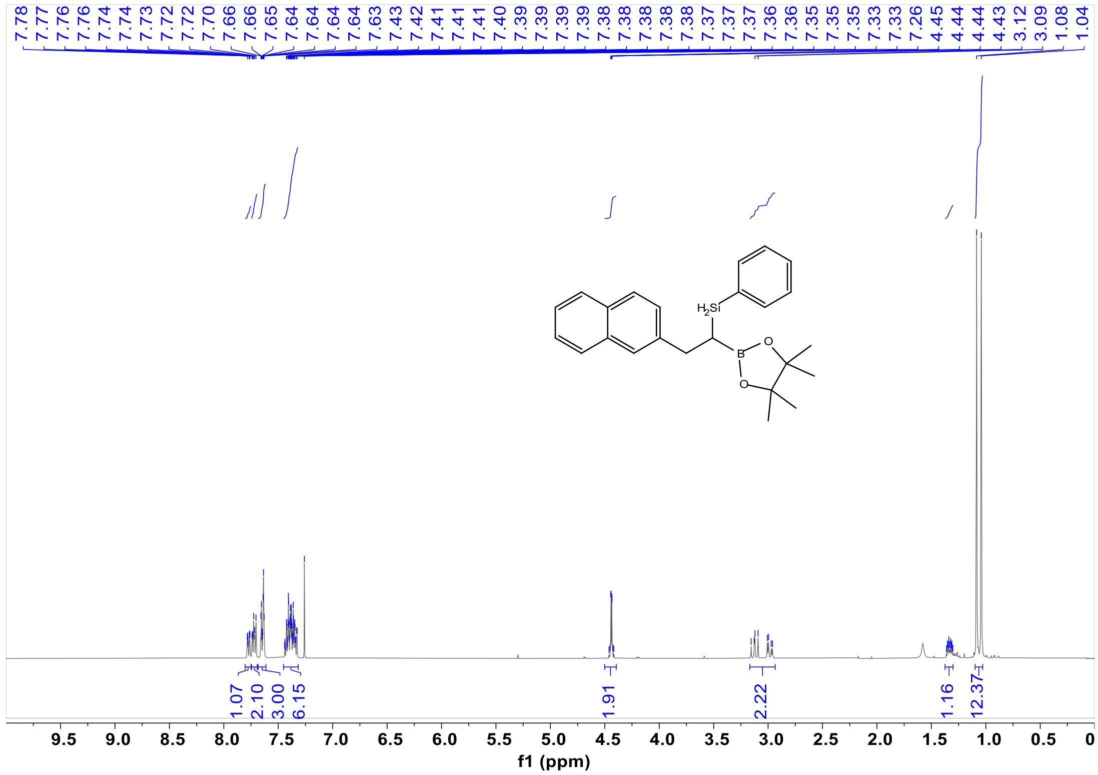

Supplementary Figure 147. ^{1}H NMR spectra for **45**

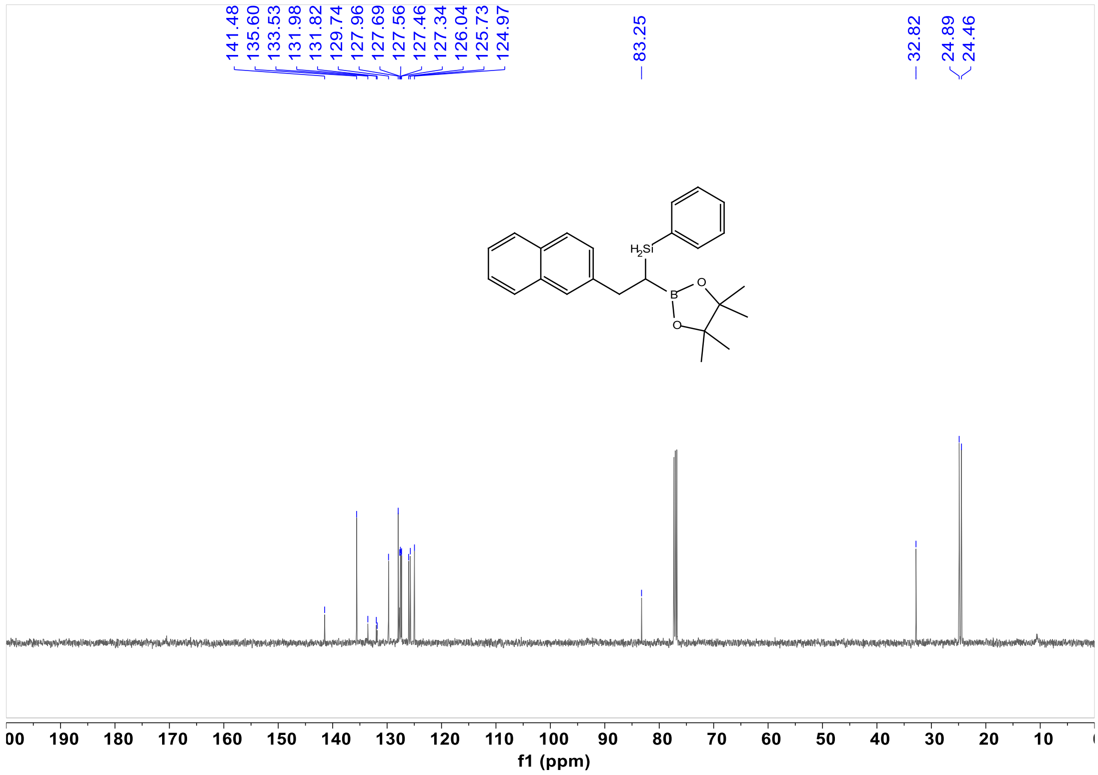

Supplementary Figure 148. ^{13}C NMR spectra for **45**

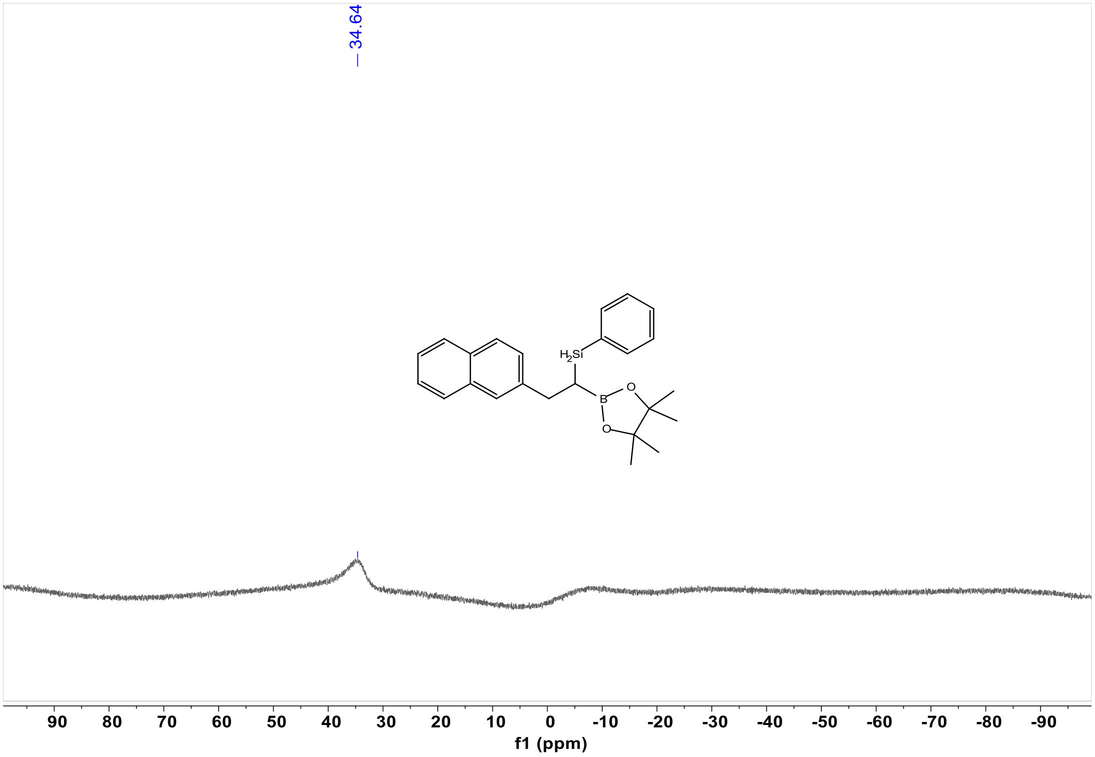

Supplementary Figure 149. ^{11}B NMR spectra for **45**

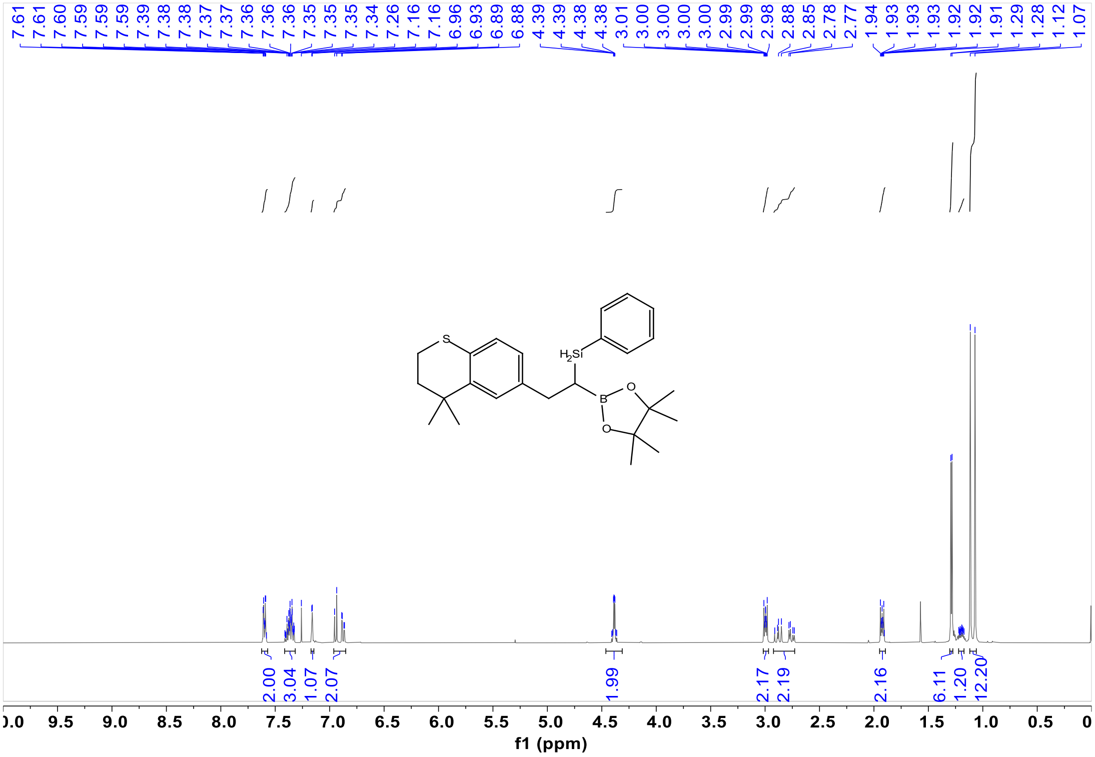

Supplementary Figure 150. ^{1}H NMR spectra for **46**

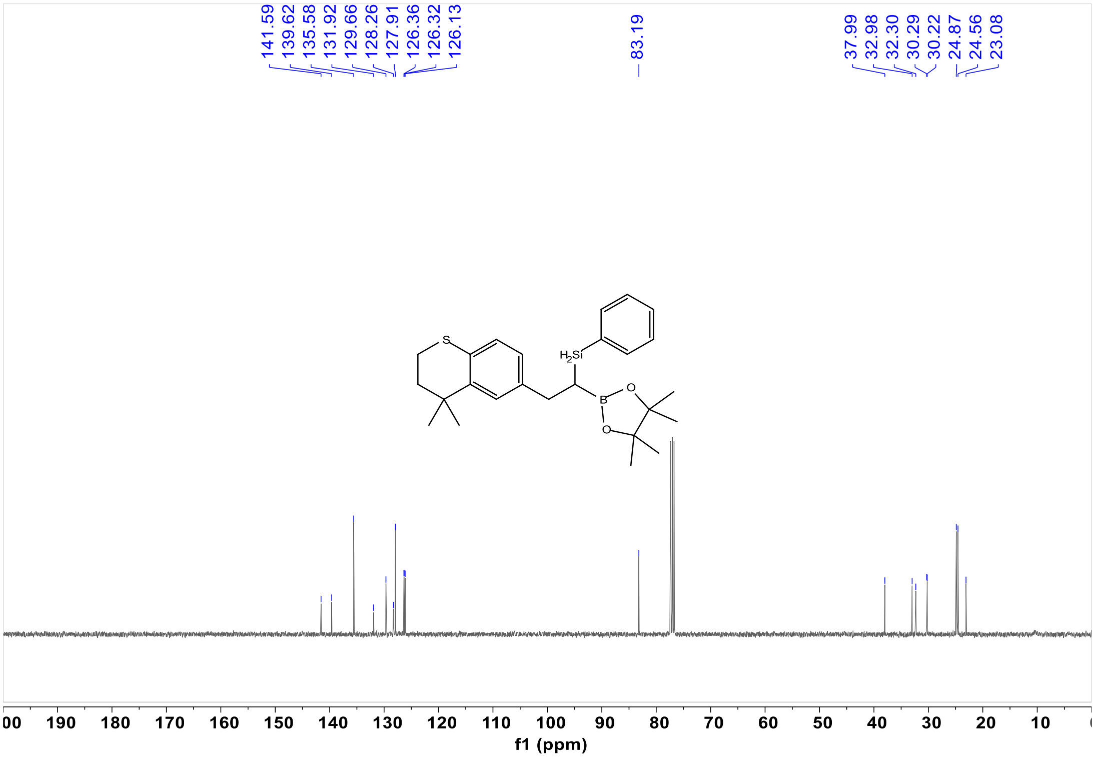

Supplementary Figure 151. ^{13}C NMR spectra for **46**

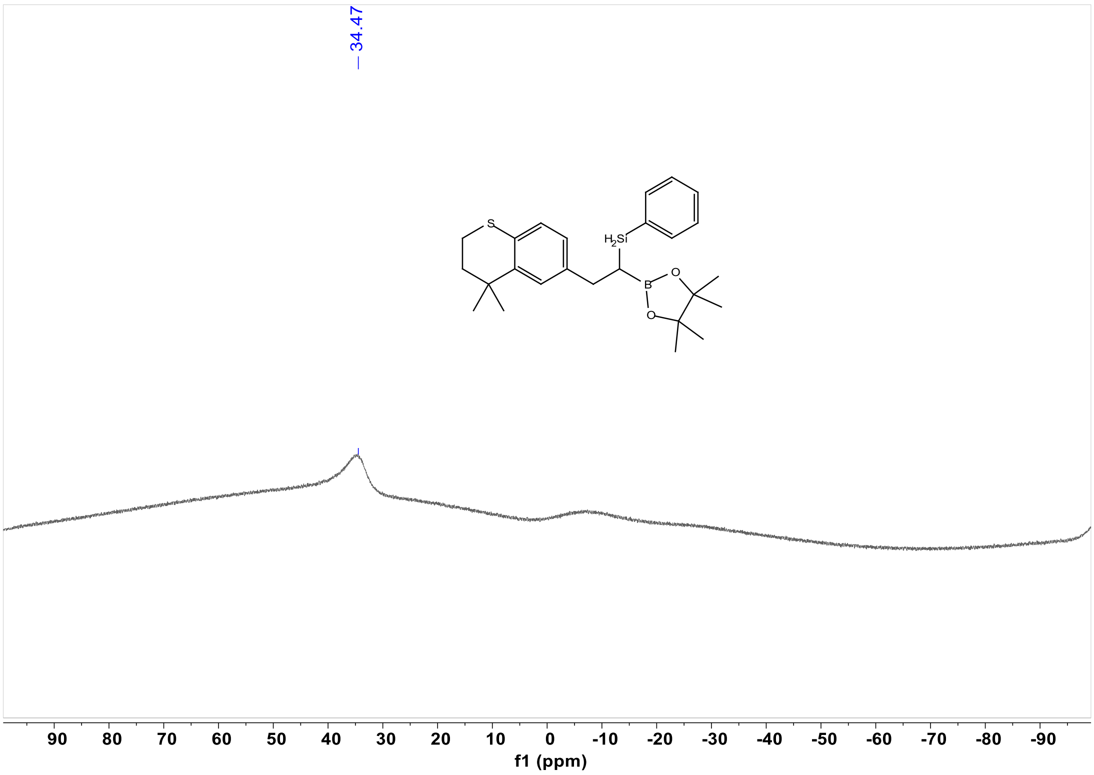

Supplementary Figure 152. ^{11}B NMR spectra for **46**

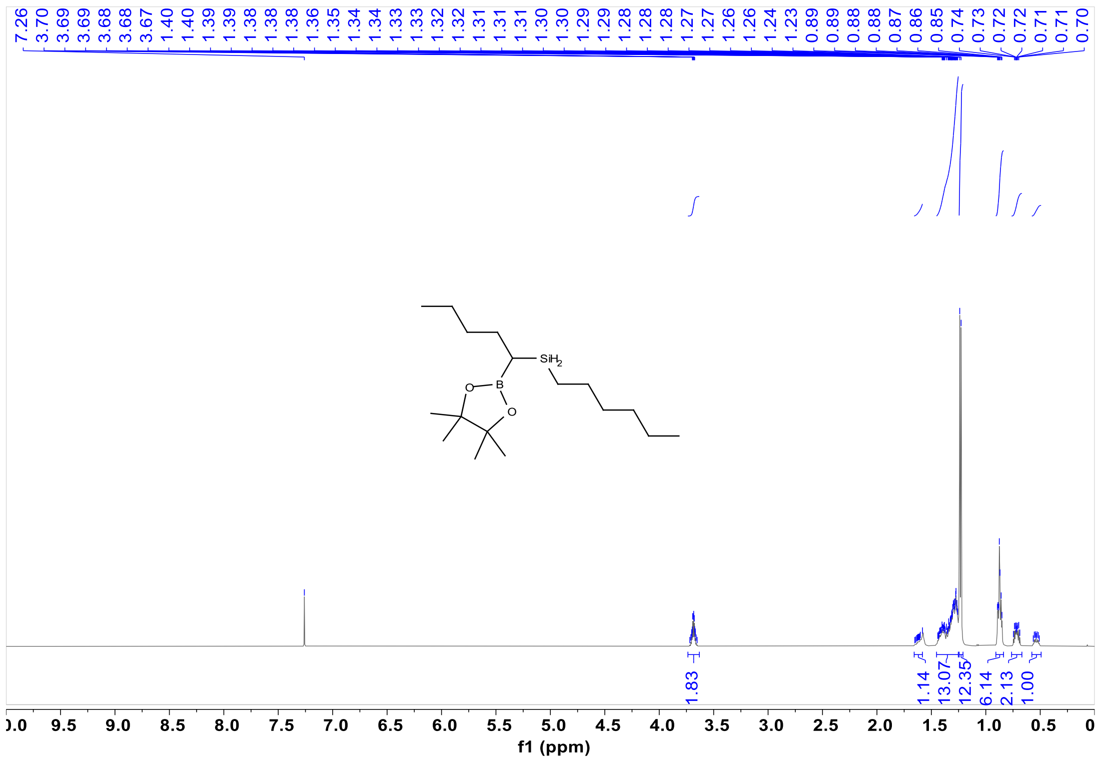

Supplementary Figure 153. ^{1}H NMR spectra for **47**

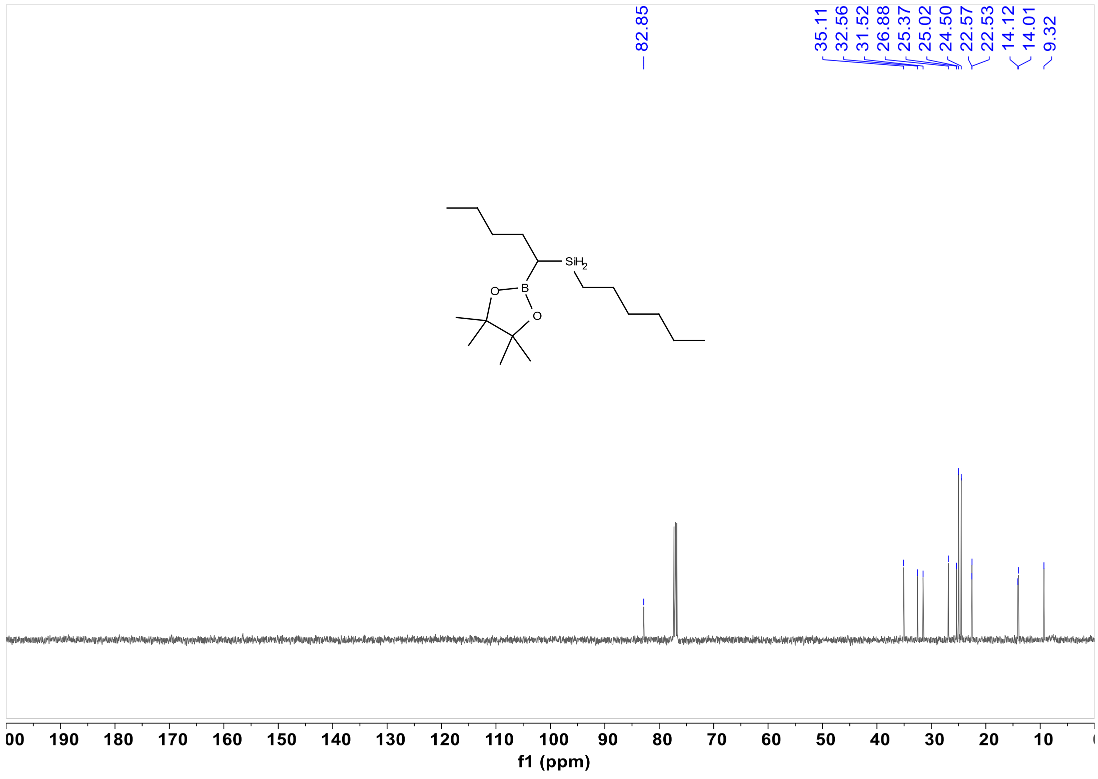

Supplementary Figure 154. ^{13}C NMR spectra for **47**

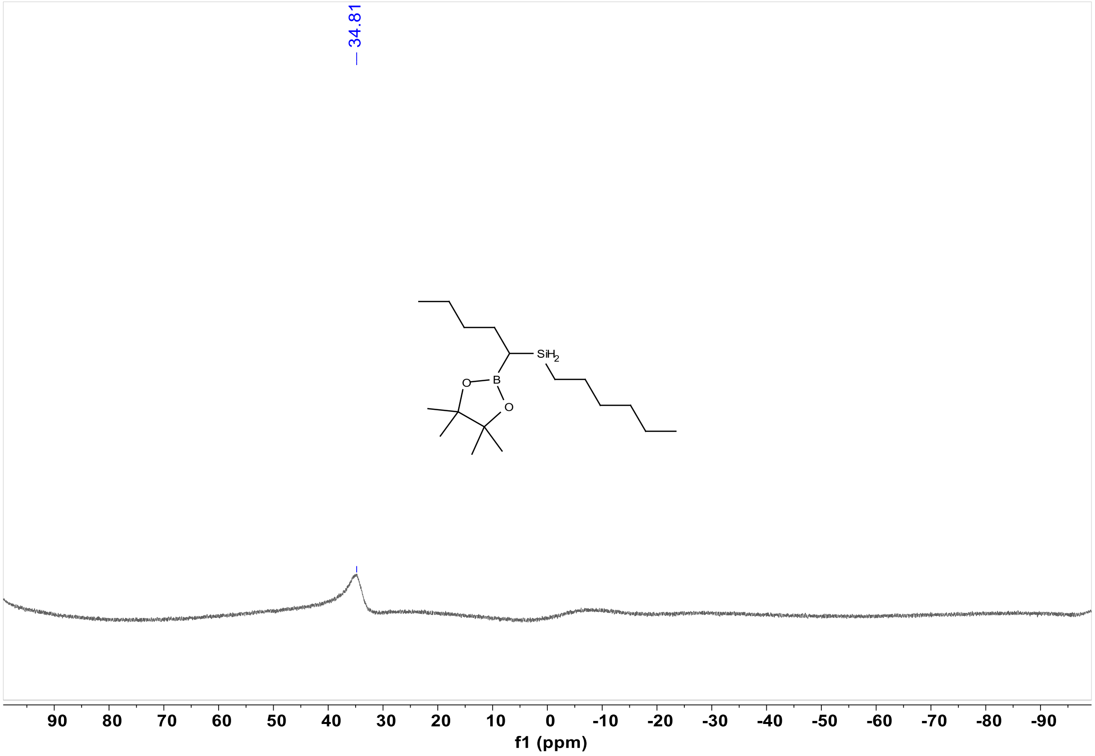

Supplementary Figure 155. ^{11}B NMR spectra for **47**

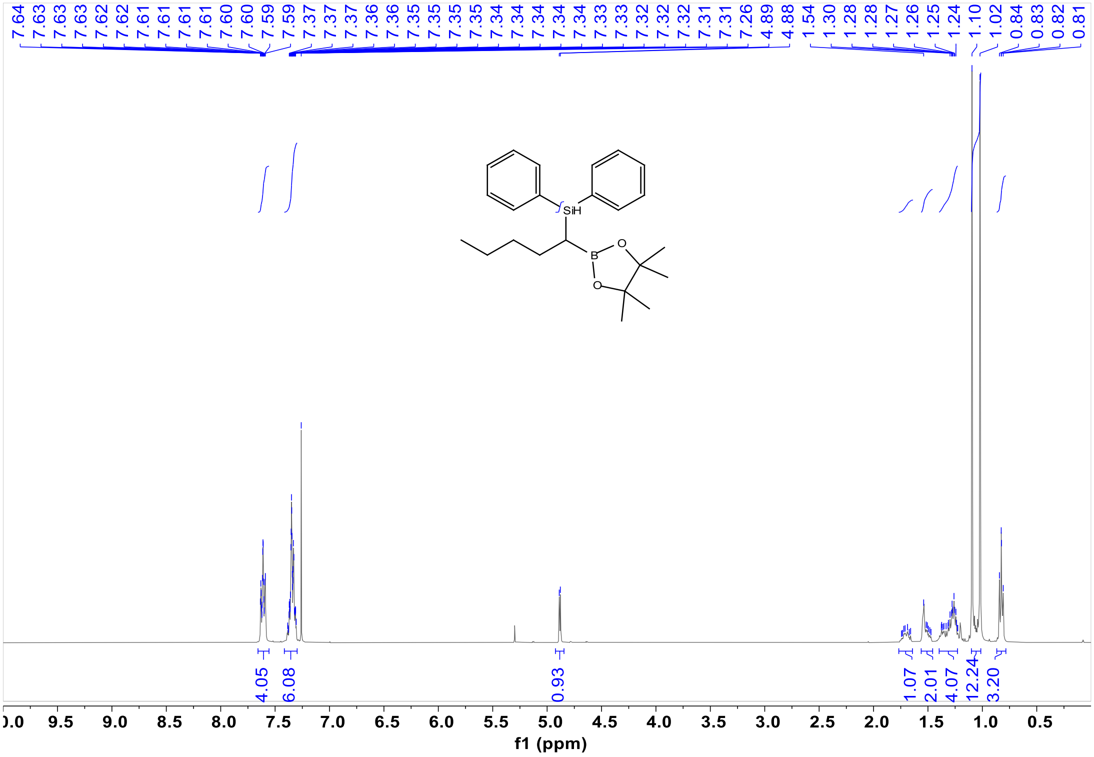

Supplementary Figure 156. ^{1}H NMR spectra for **48**

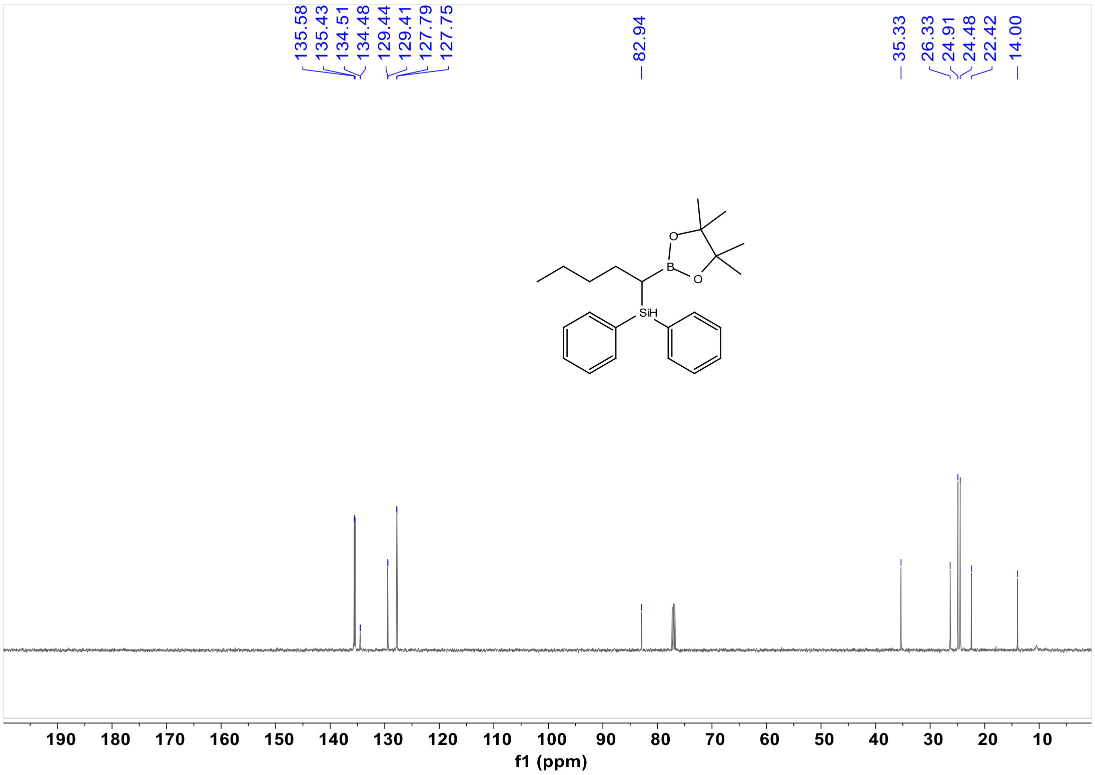

Supplementary Figure 157. ^{13}C NMR spectra for **48**

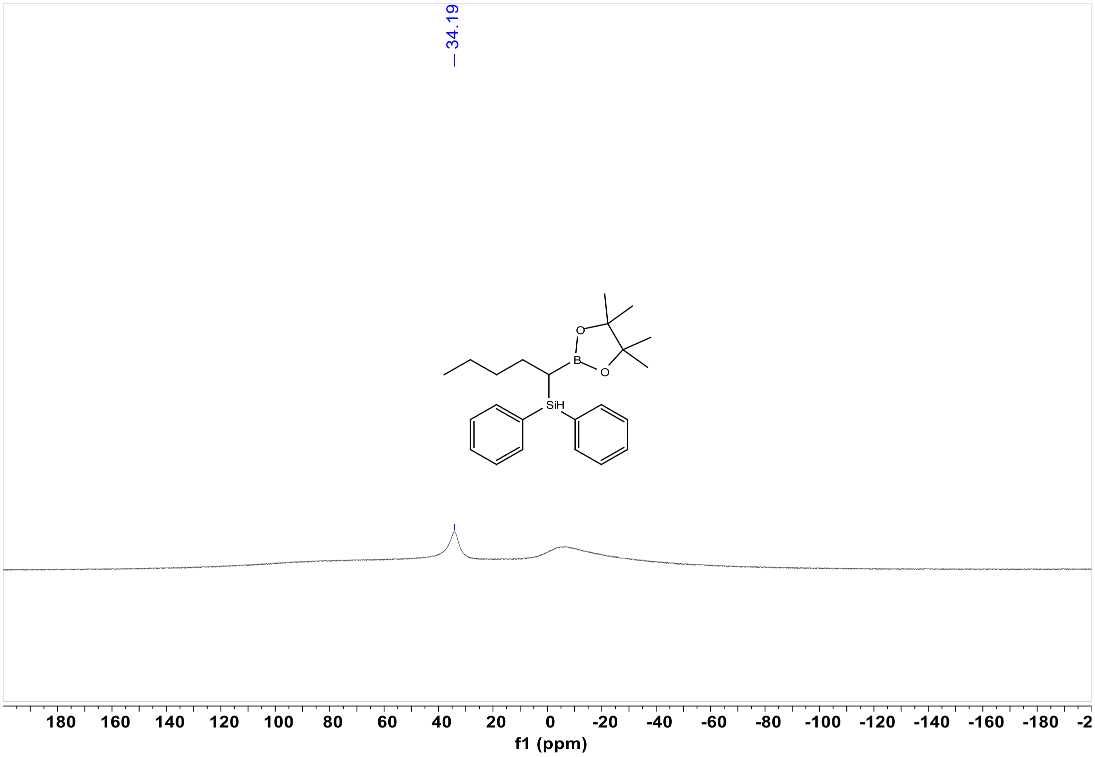

Supplementary Figure 158. ^{11}B NMR spectra for **48**

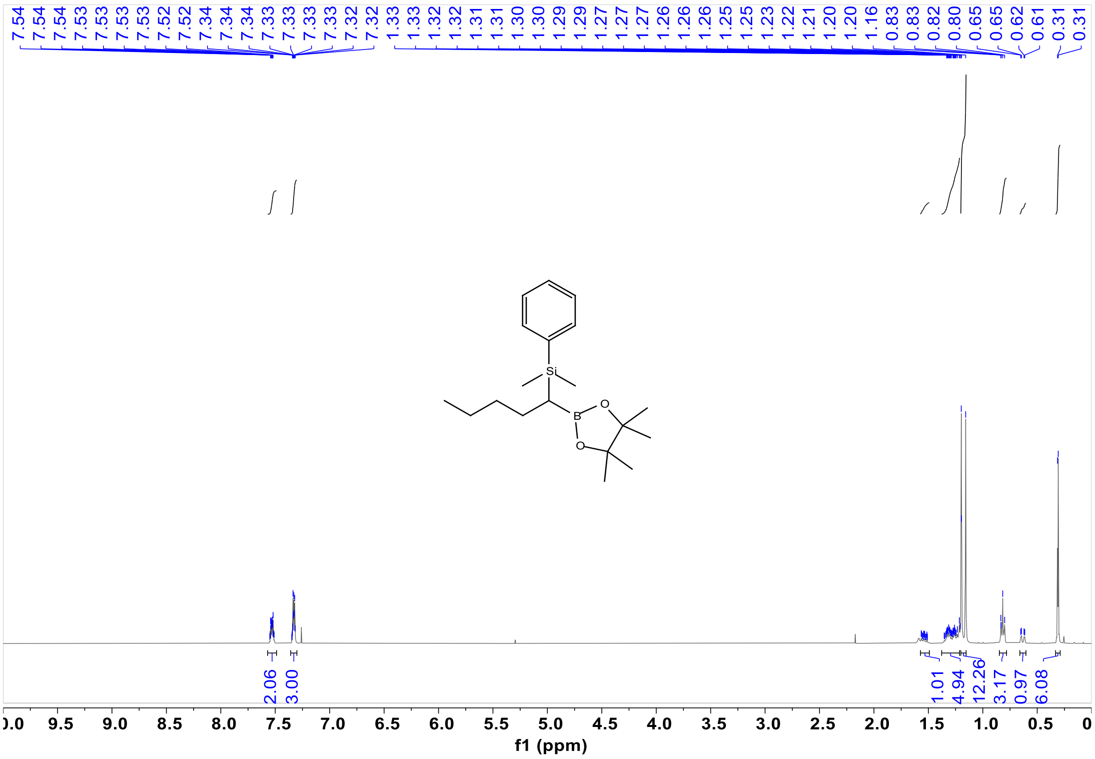

Supplementary Figure 159. ^{1}H NMR spectra for **49**

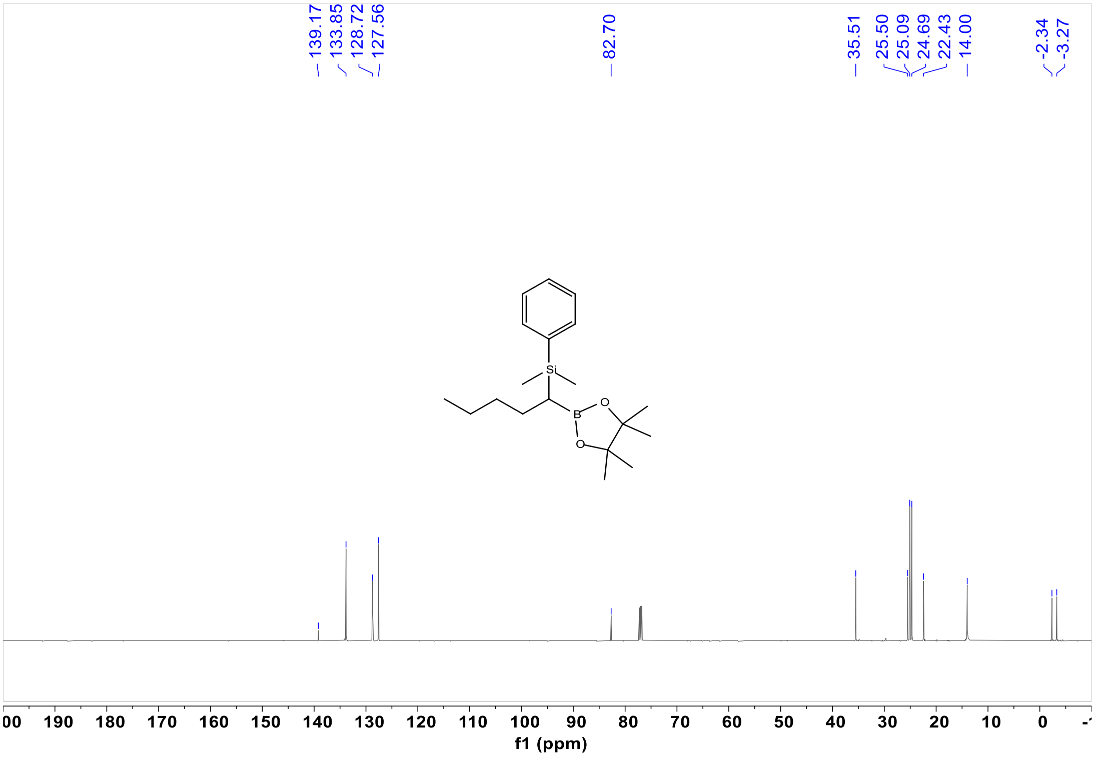

Supplementary Figure 160. ^{13}C NMR spectra for **49**

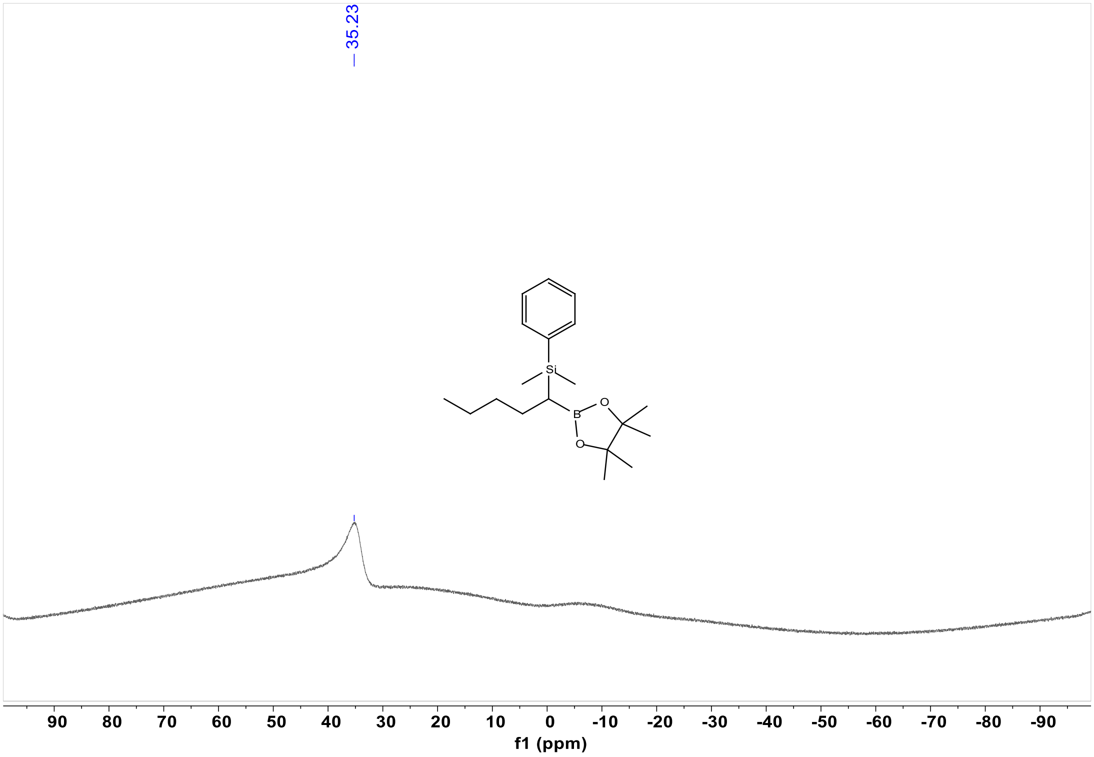

Supplementary Figure 161. ^{11}B NMR spectra for **49**

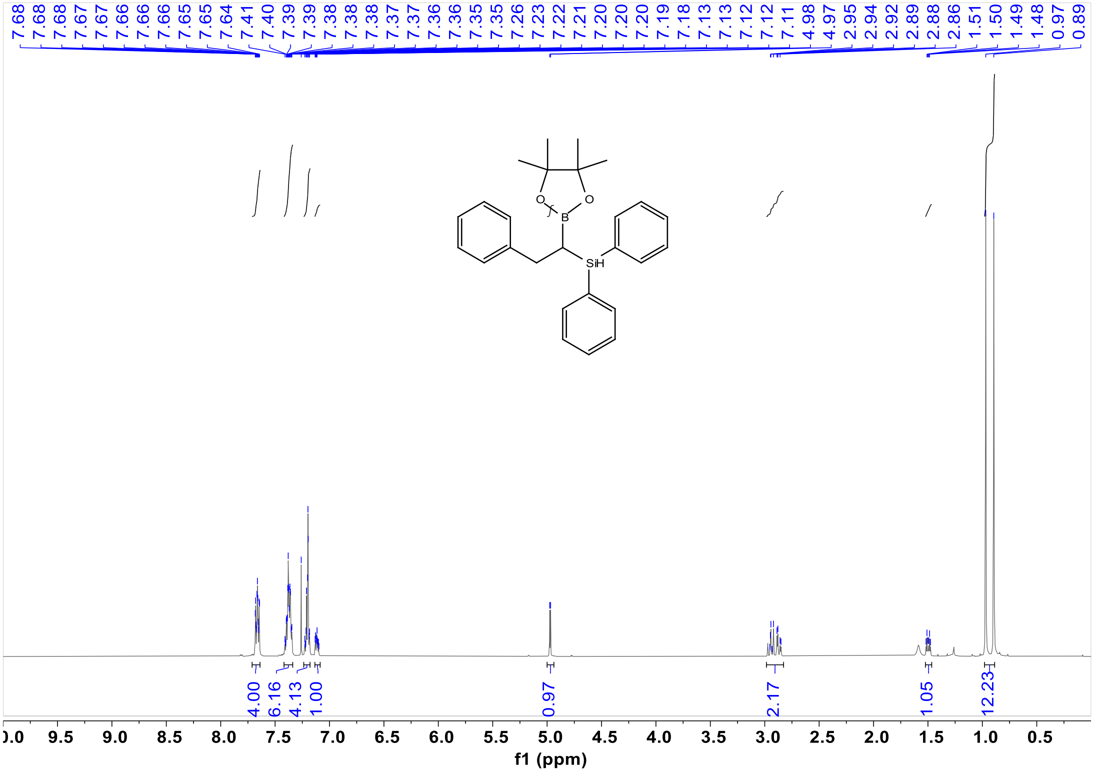

Supplementary Figure 162. ^{1}H NMR spectra for **50**

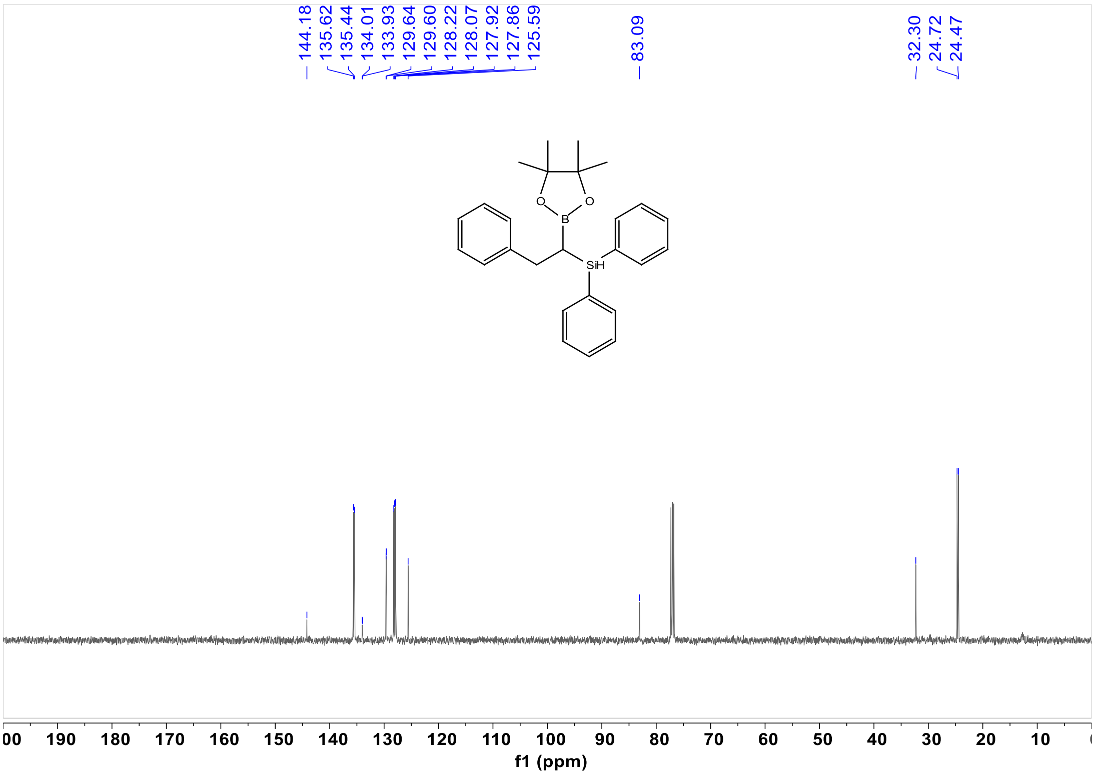

Supplementary Figure 163. ^{13}C NMR spectra for **50**

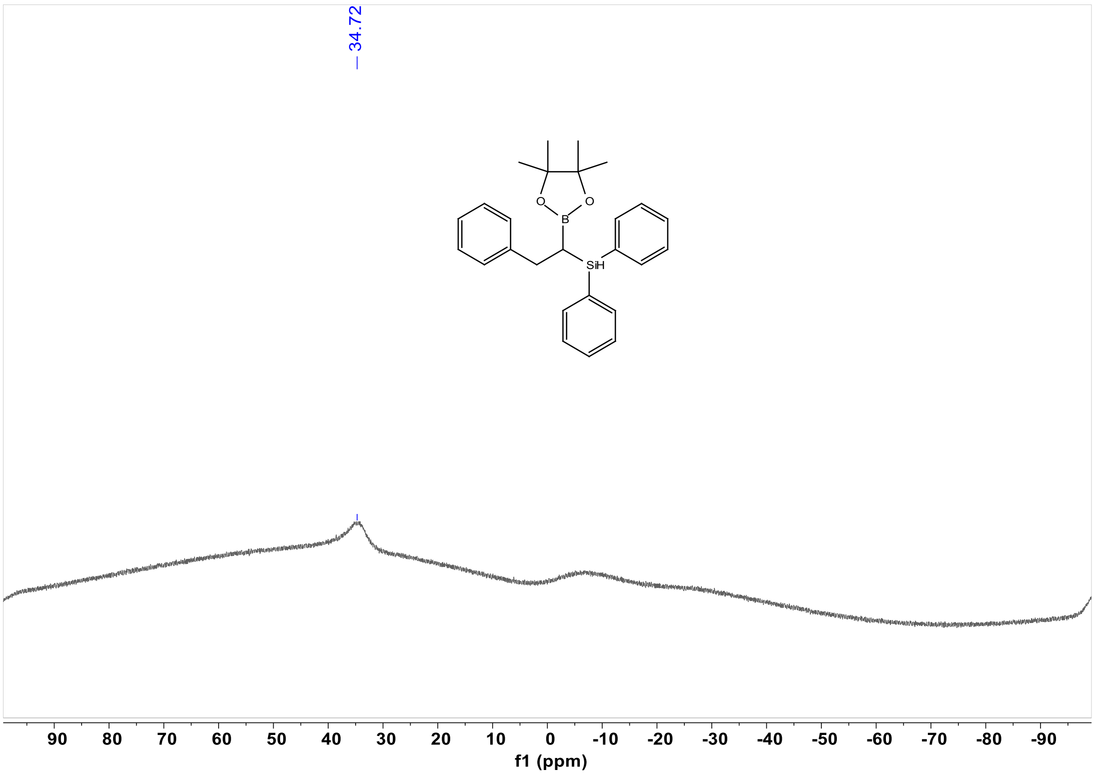

Supplementary Figure 164. ^{11}B NMR spectra for **50**

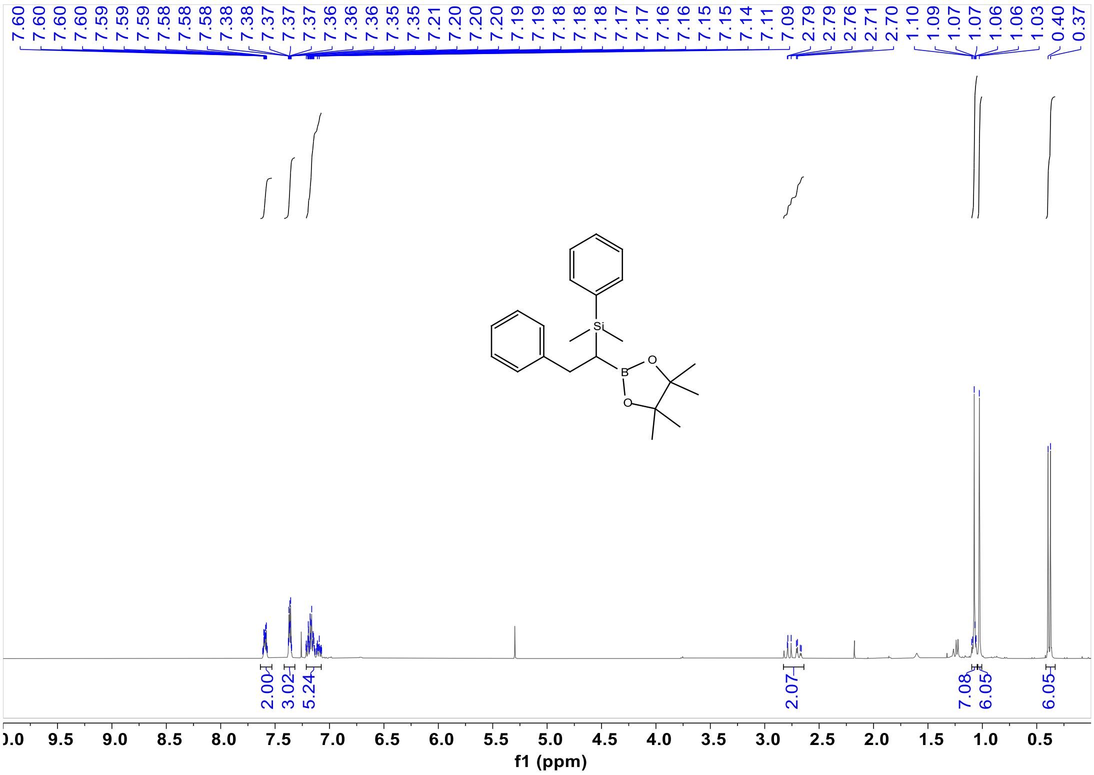

Supplementary Figure 165. ^{1}H NMR spectra for **51**

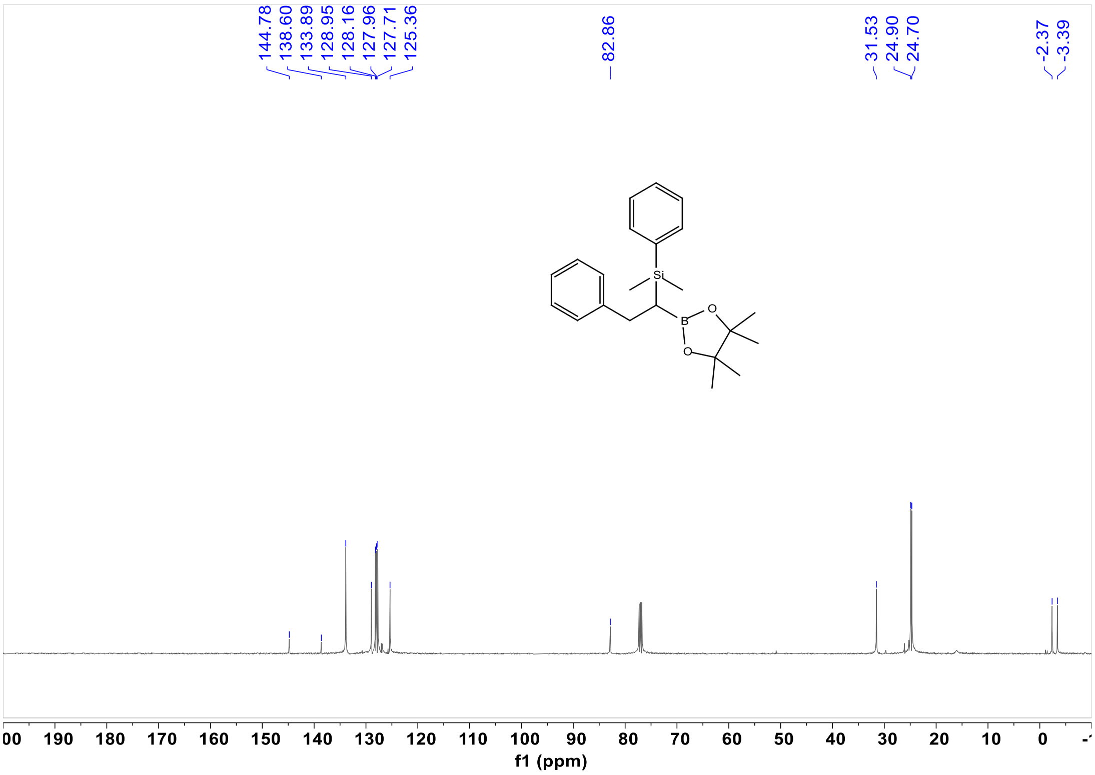

Supplementary Figure 166. ^{13}C NMR spectra for **51**

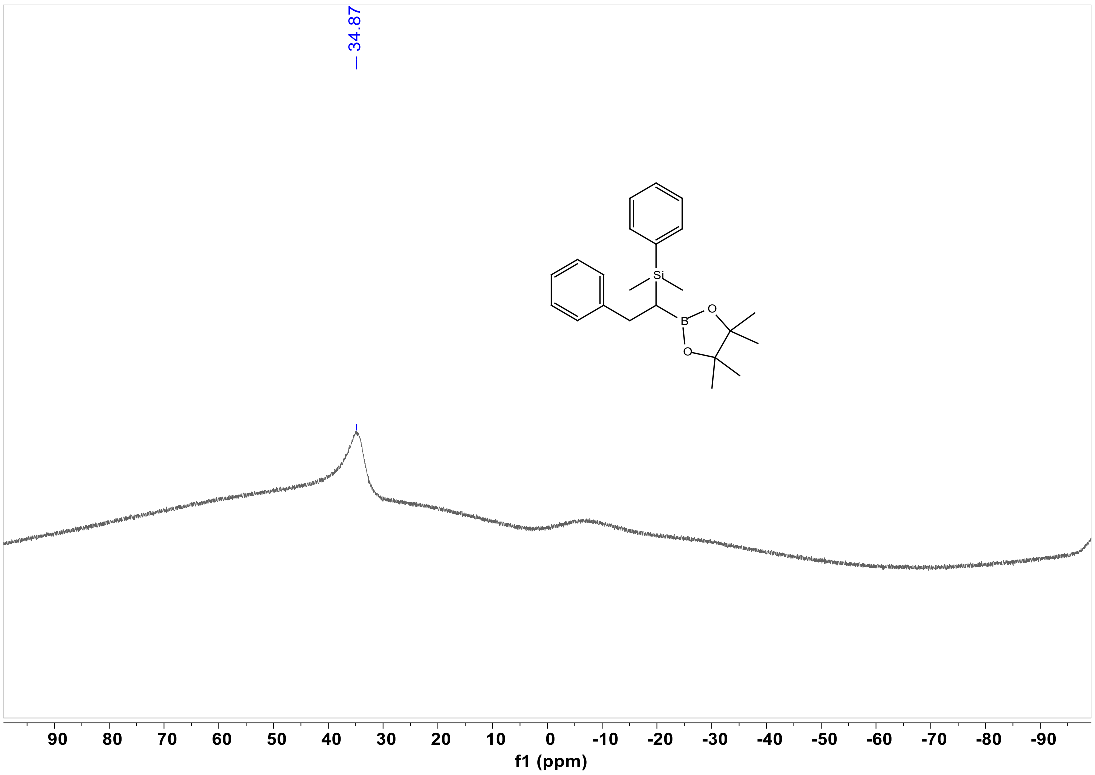

Supplementary Figure 167. ^{11}B NMR spectra for **51**

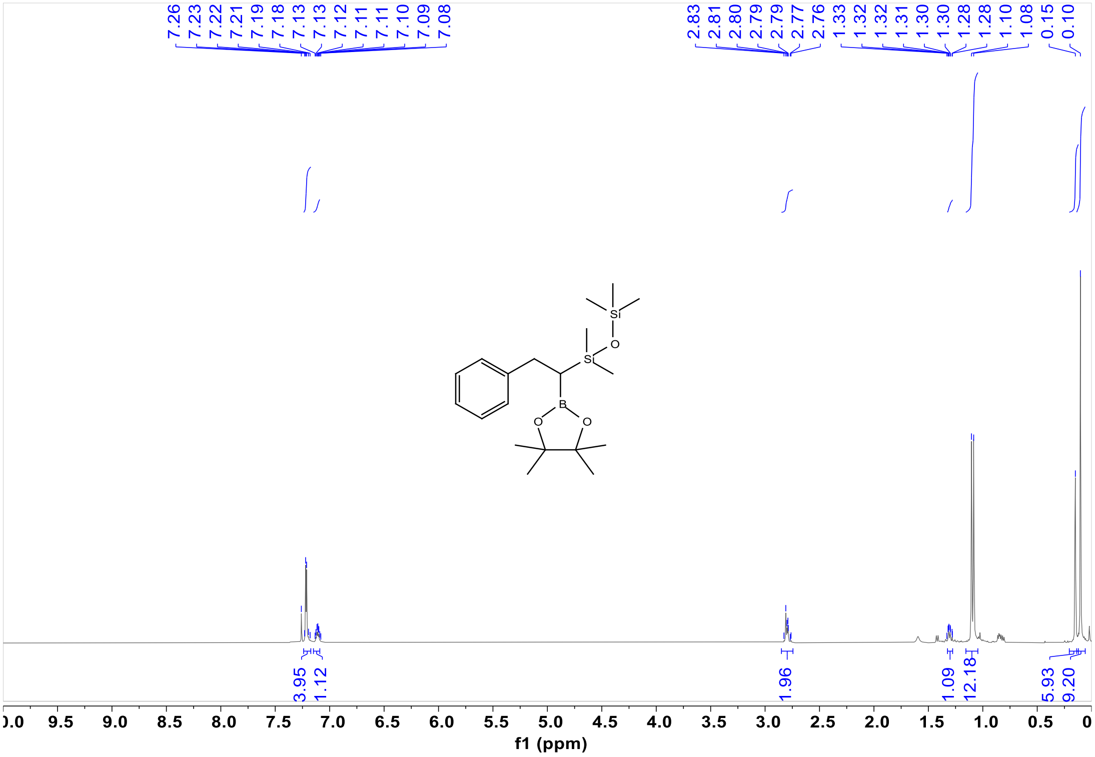

Supplementary Figure 168. ^{1}H NMR spectra for **52**

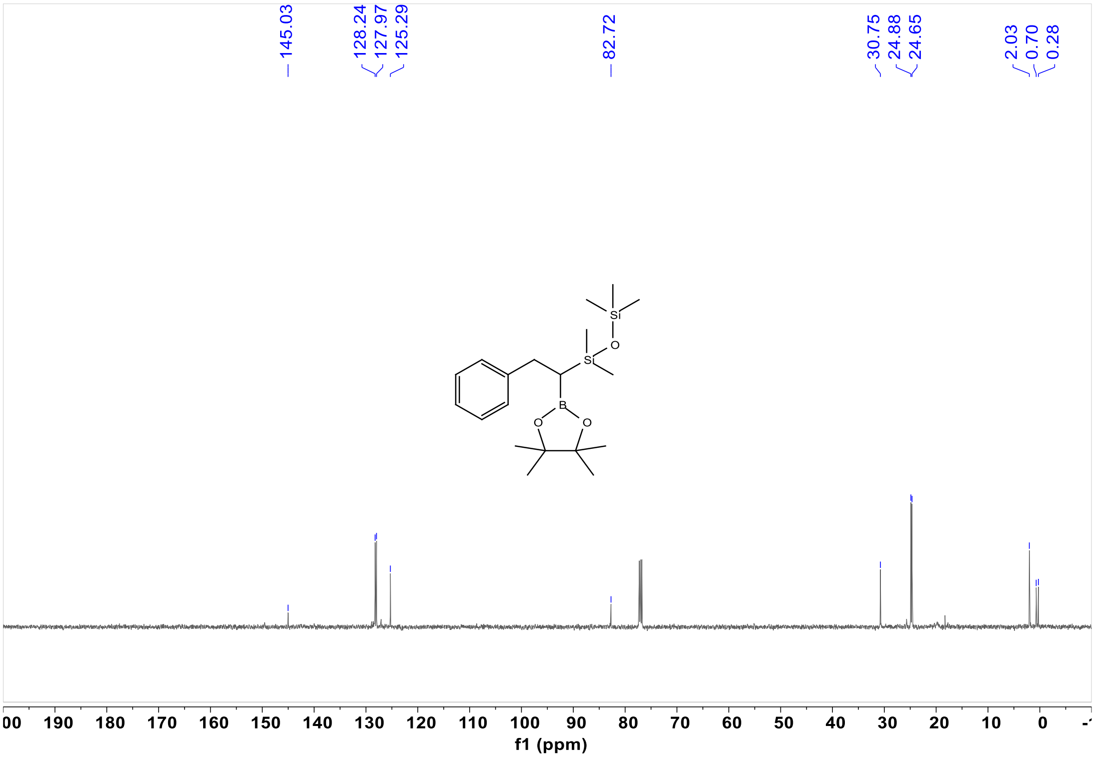

Supplementary Figure 169. ^{13}C NMR spectra for **52**

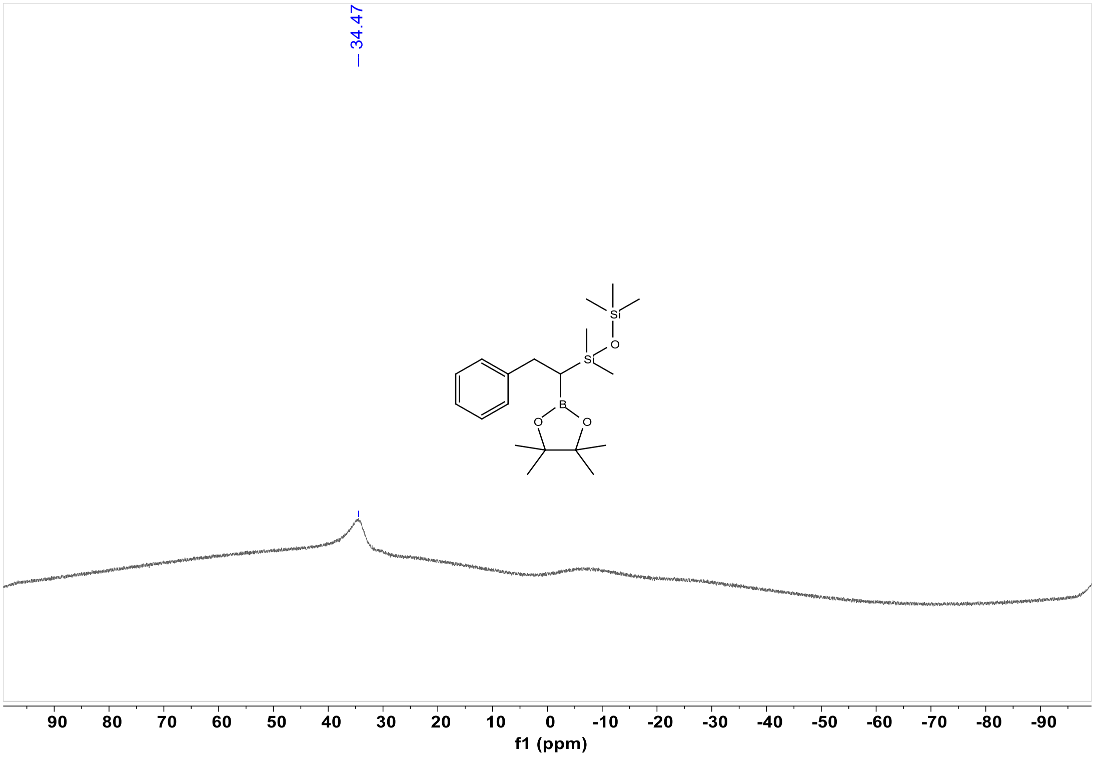

Supplementary Figure 170. ^{11}B NMR spectra for **52**

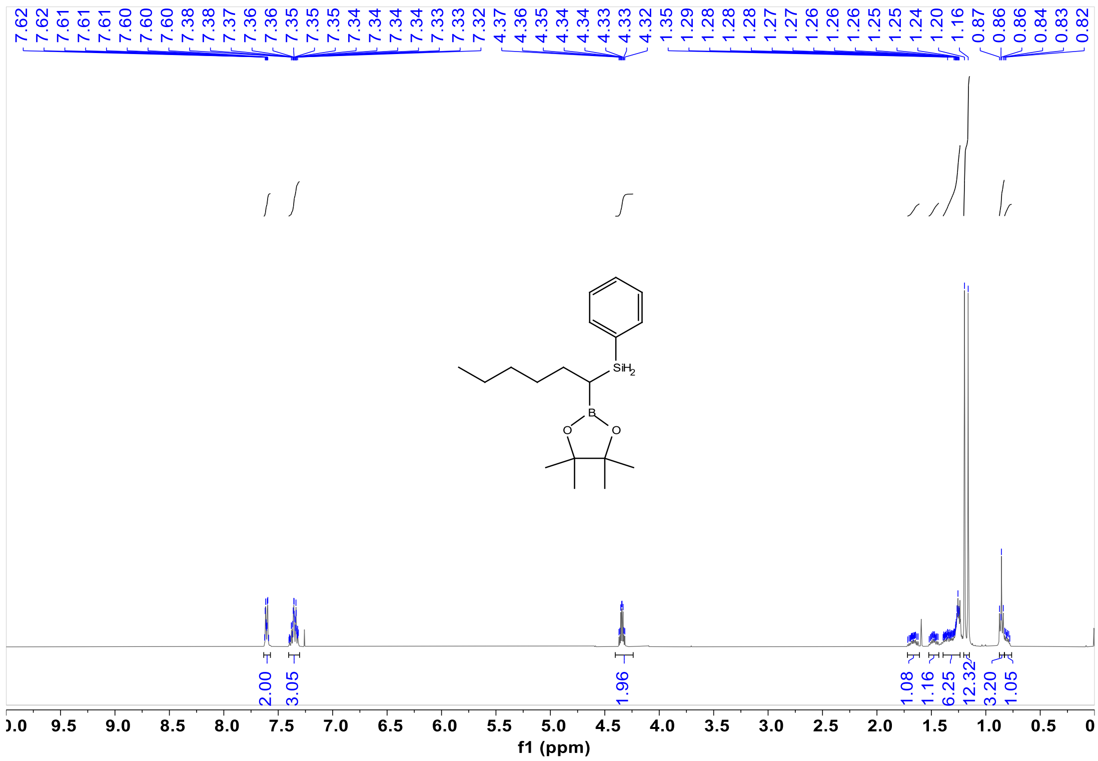

Supplementary Figure 171. ^{1}H NMR spectra for **53**

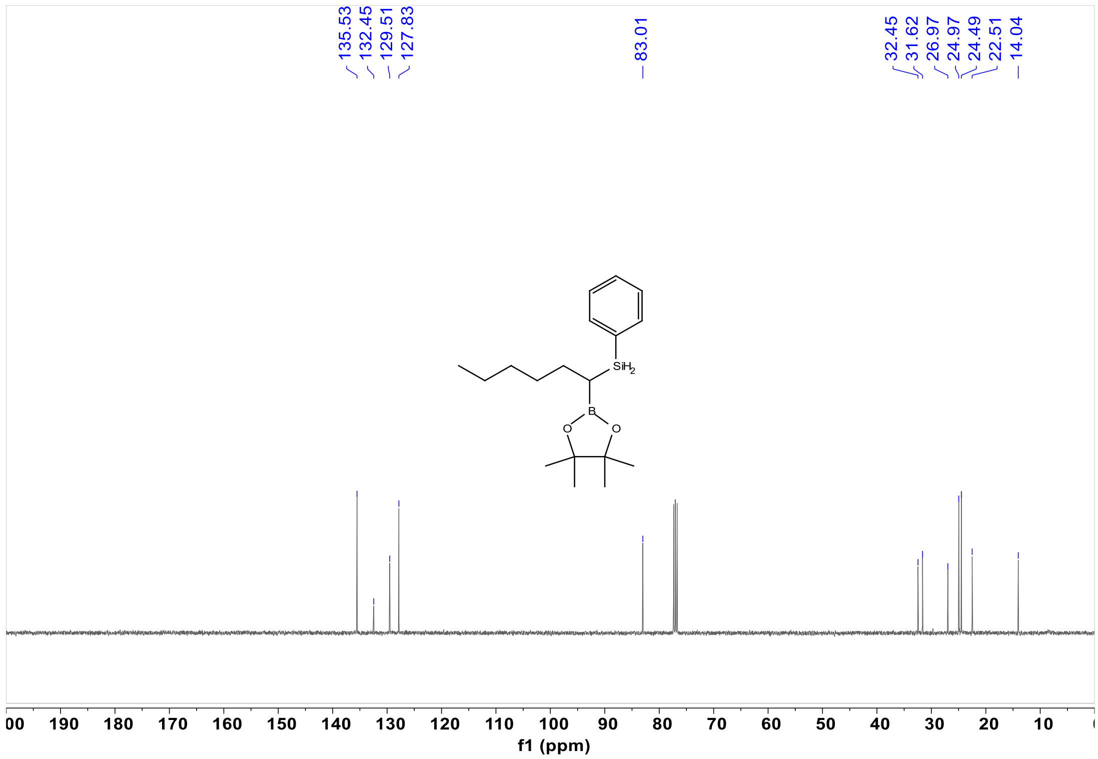

Supplementary Figure 172. ^{13}C NMR spectra for **53**

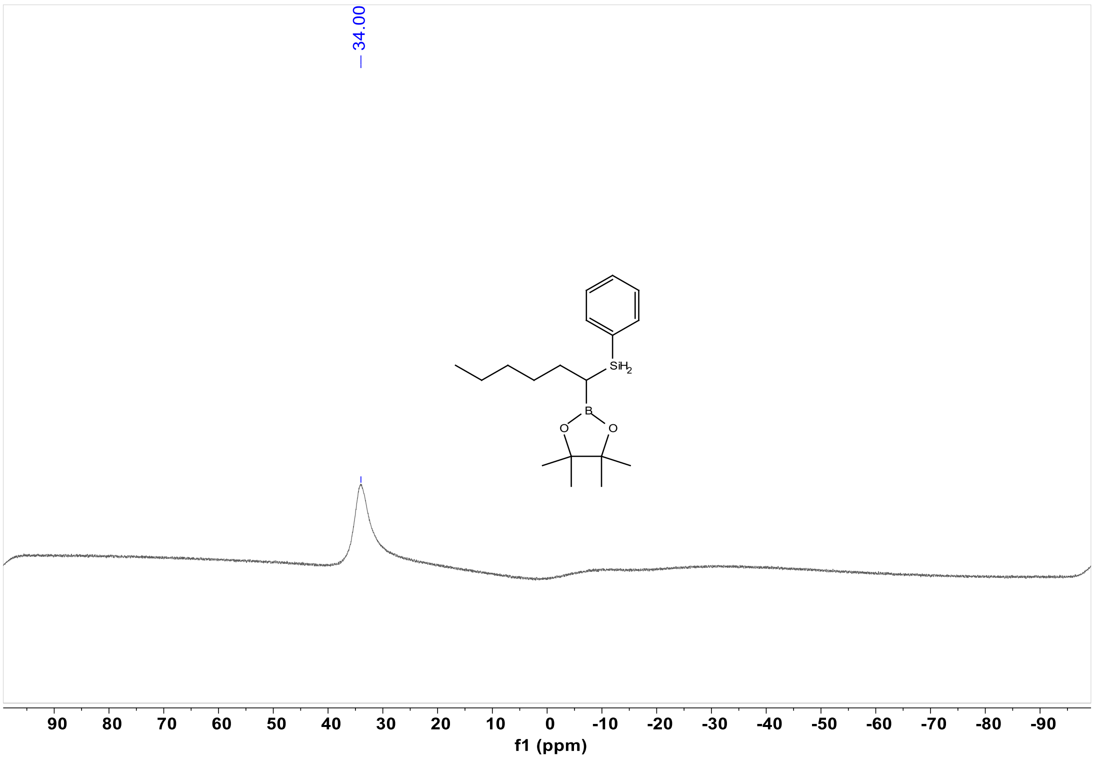

Supplementary Figure 173. ^{11}B NMR spectra for **53**

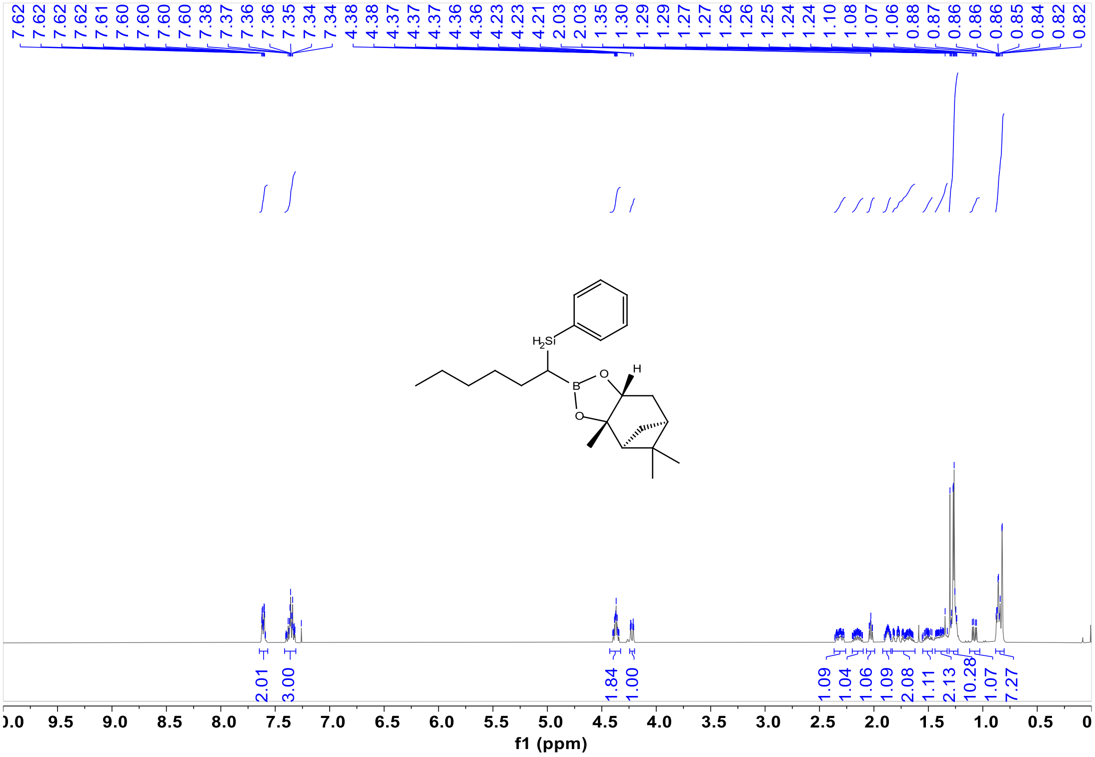

Supplementary Figure 174. ^{1}H NMR spectra for **54**

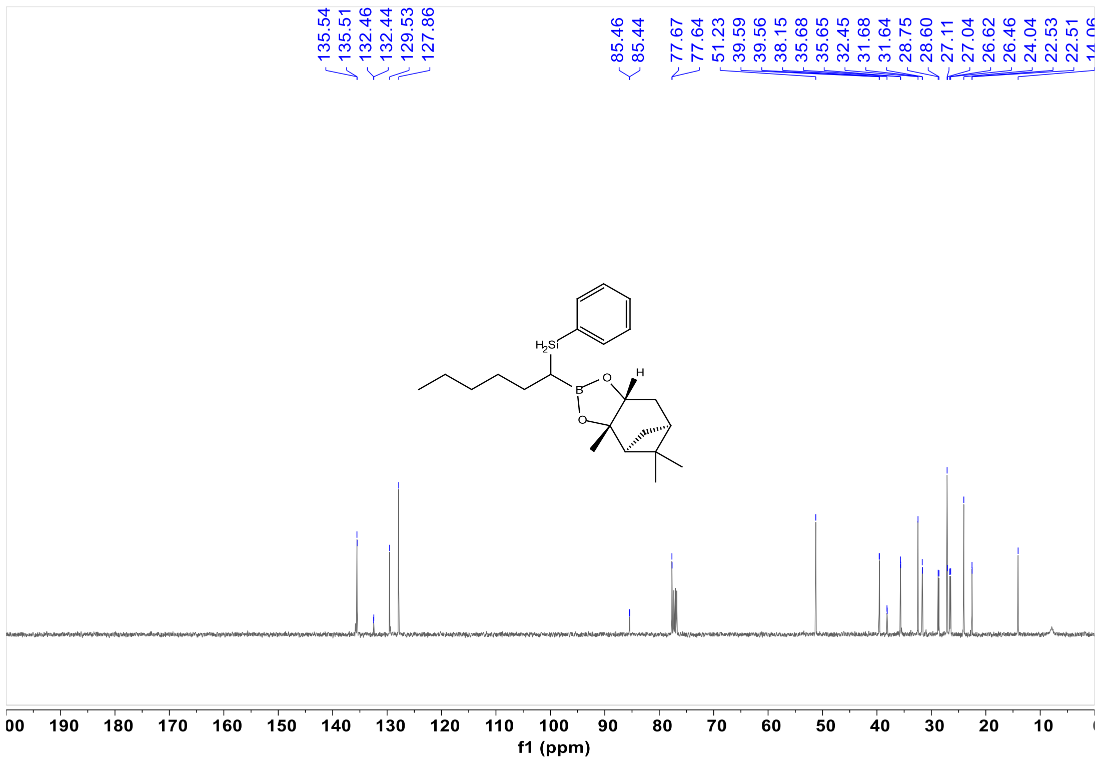

Supplementary Figure 175. ^{13}C NMR spectra for **54**

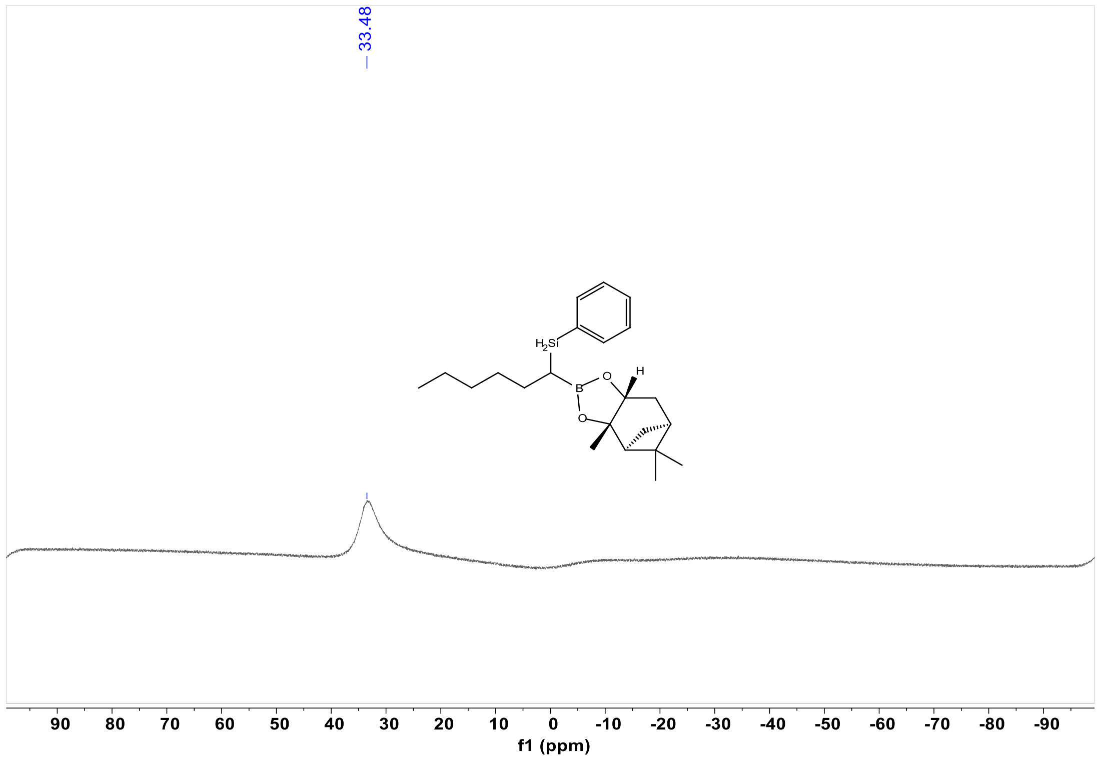

Supplementary Figure 176. ^{11}B NMR spectra for **54**

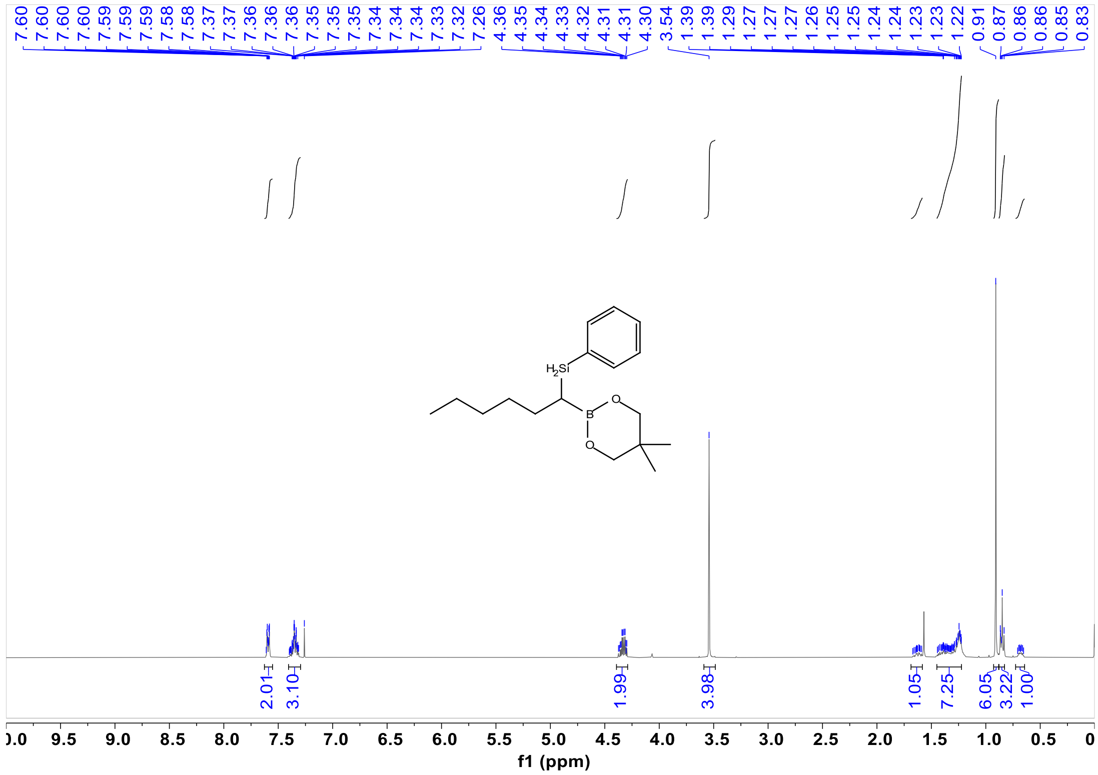

Supplementary Figure 177. ^{1}H NMR spectra for **55**

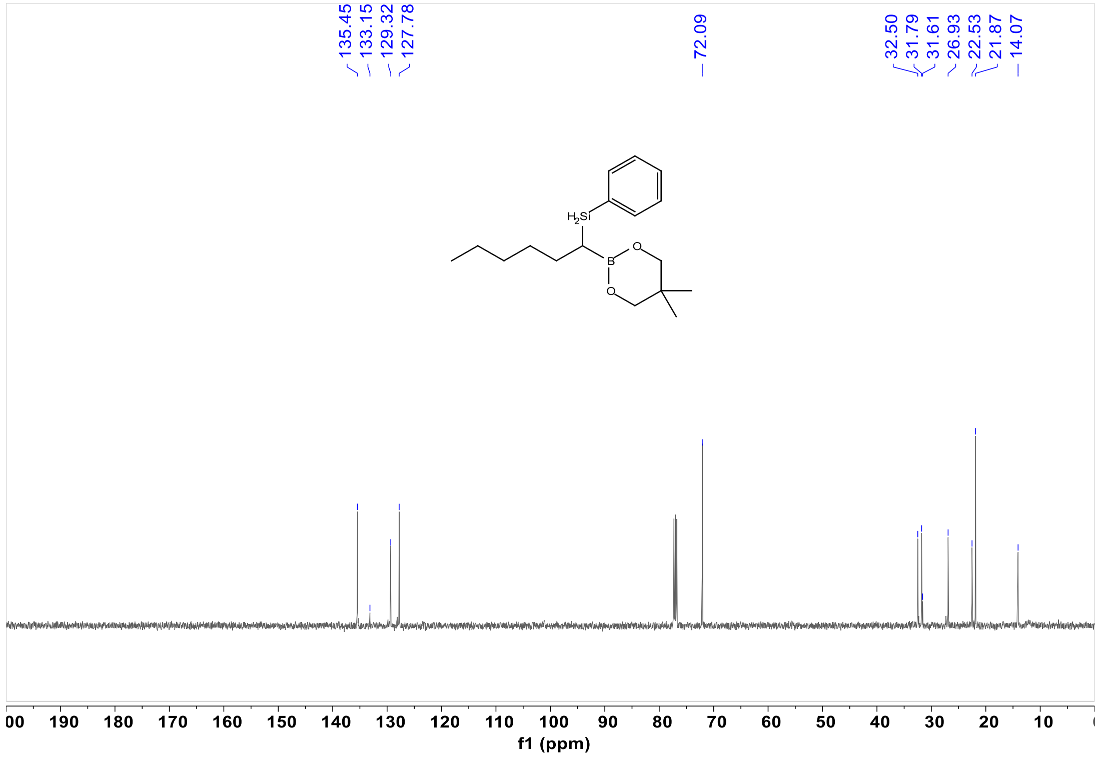

Supplementary Figure 178. ^{13}C NMR spectra for **55**

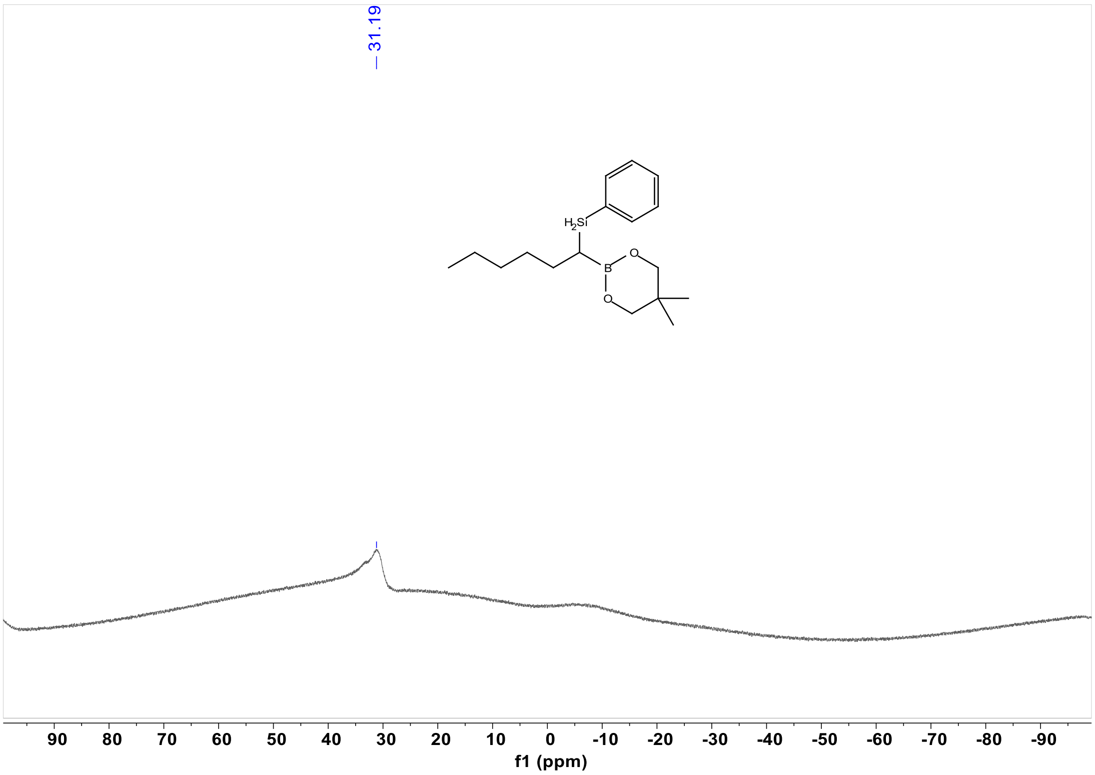

Supplementary Figure 179. ^{11}B NMR spectra for **55**

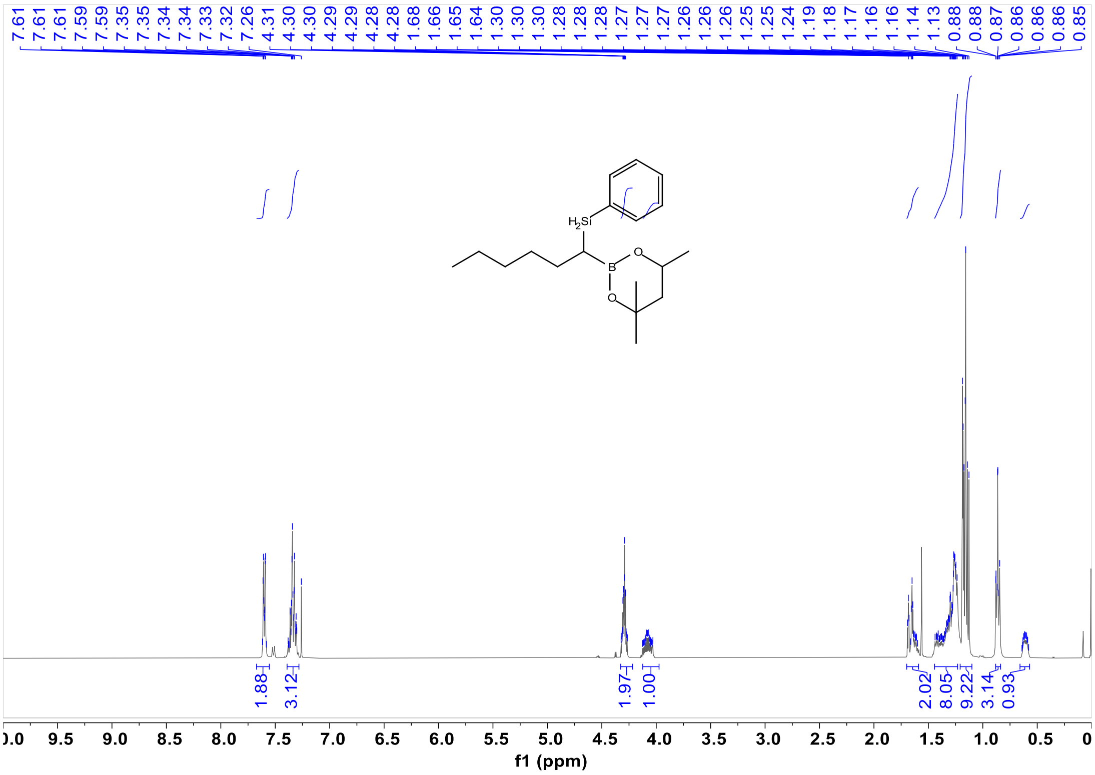

Supplementary Figure 180. ^{1}H NMR spectra for **56**

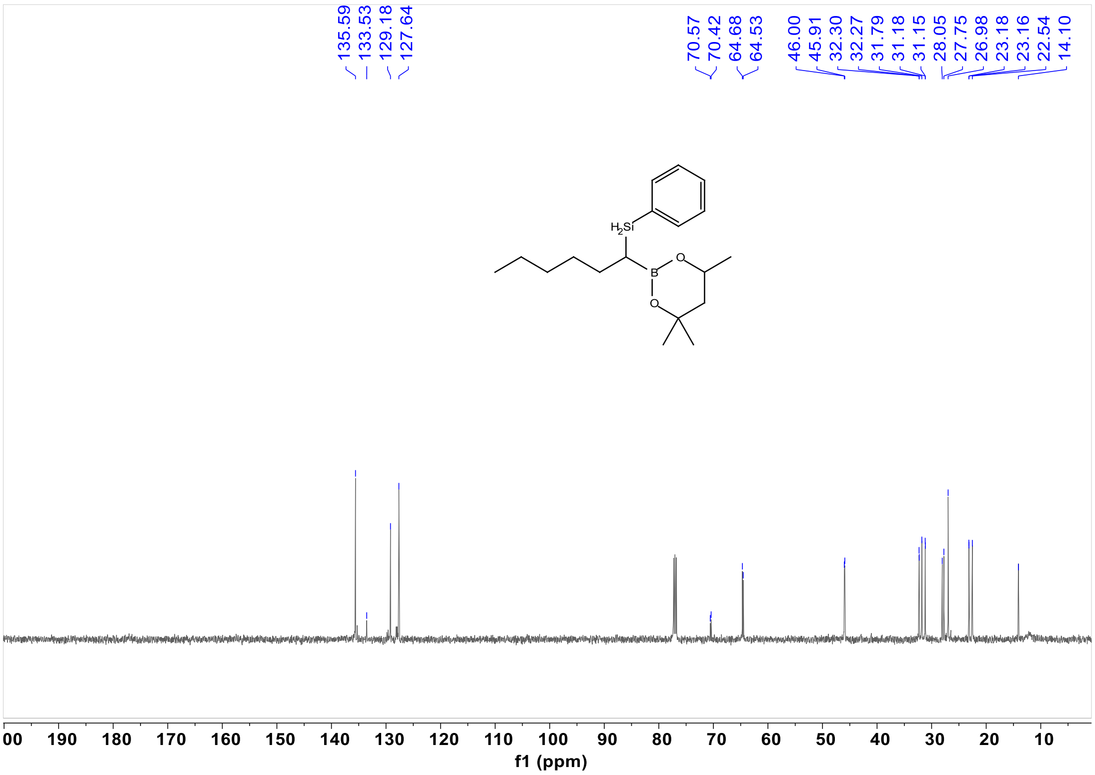

Supplementary Figure 181. ^{13}C NMR spectra for **56**

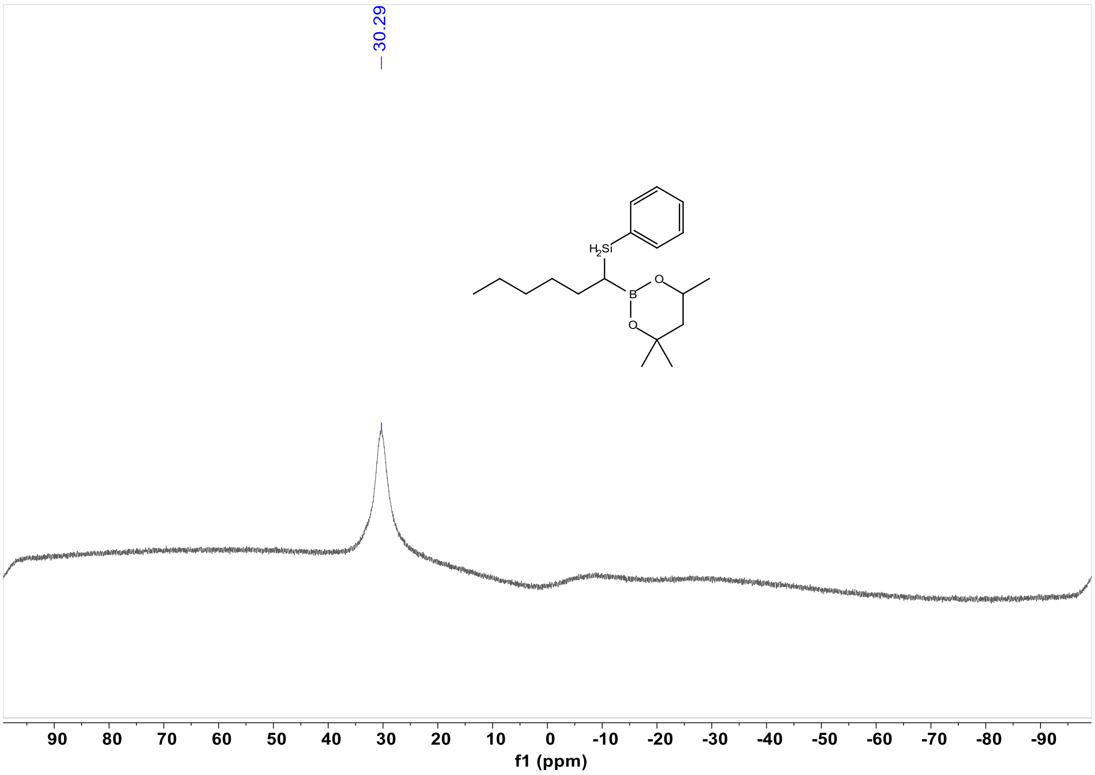

Supplementary Figure 182. ^{11}B NMR spectra for **56**

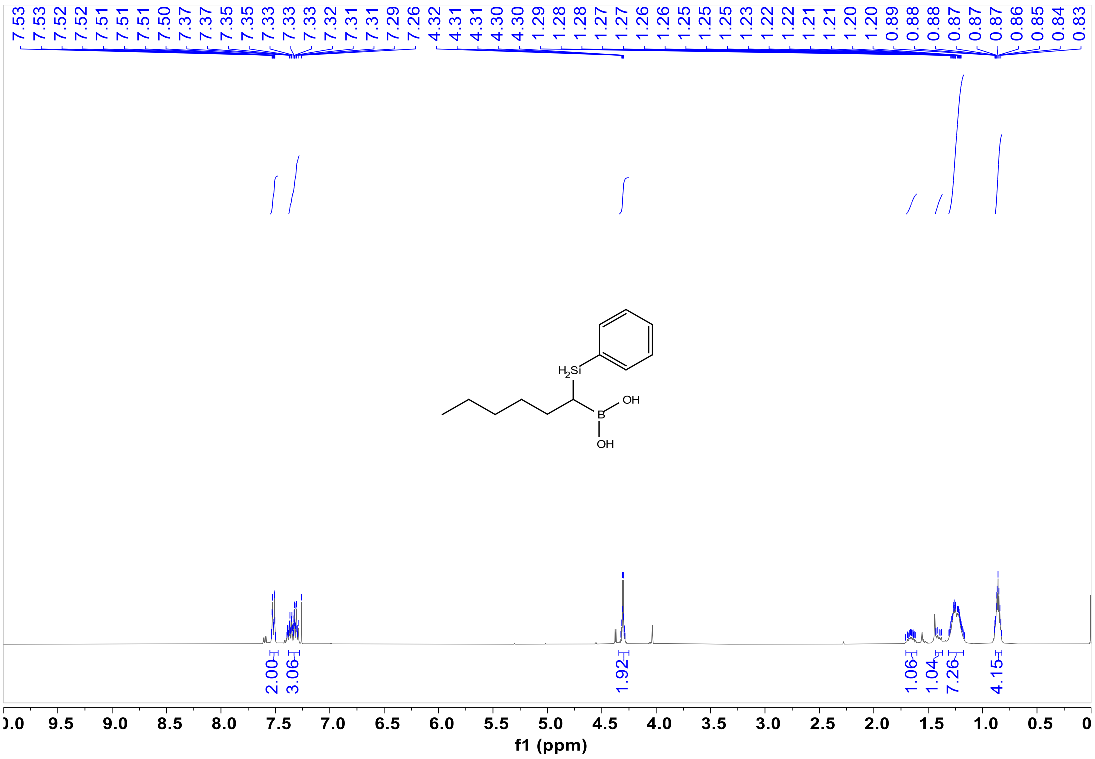

Supplementary Figure 183. ^{1}H NMR spectra for **57**

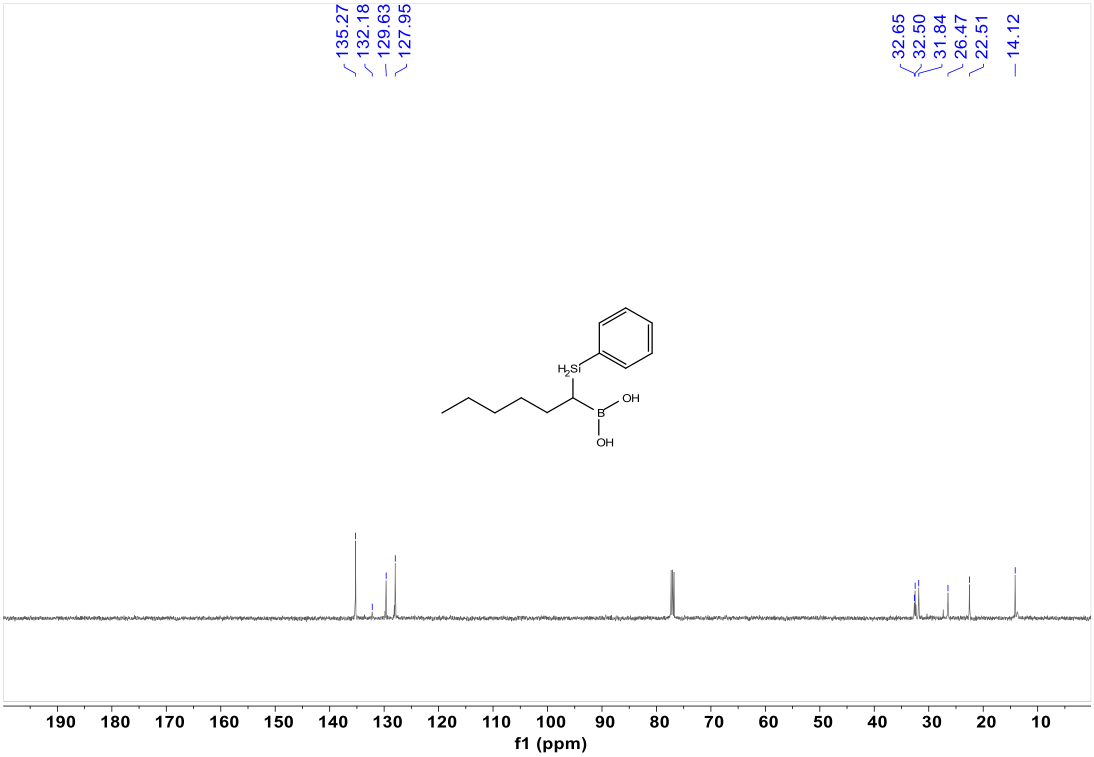

Supplementary Figure 184. ^{13}C NMR spectra for **57**

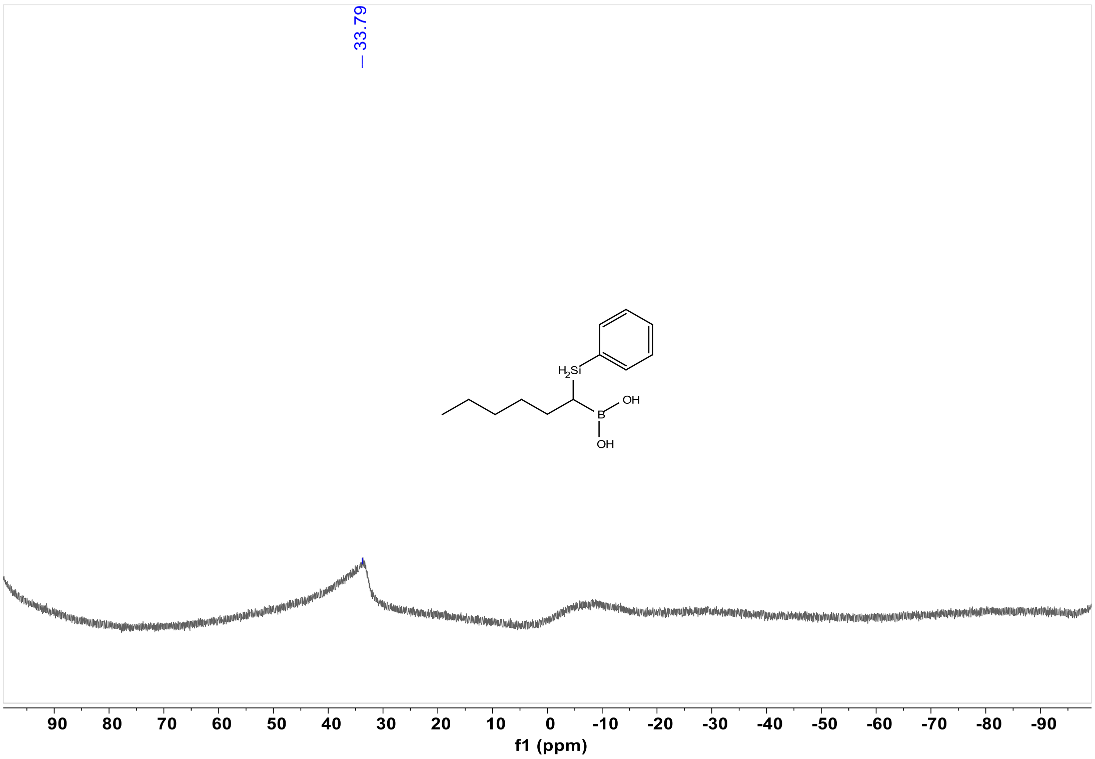

Supplementary Figure 185. ^{11}B NMR spectra for **57**

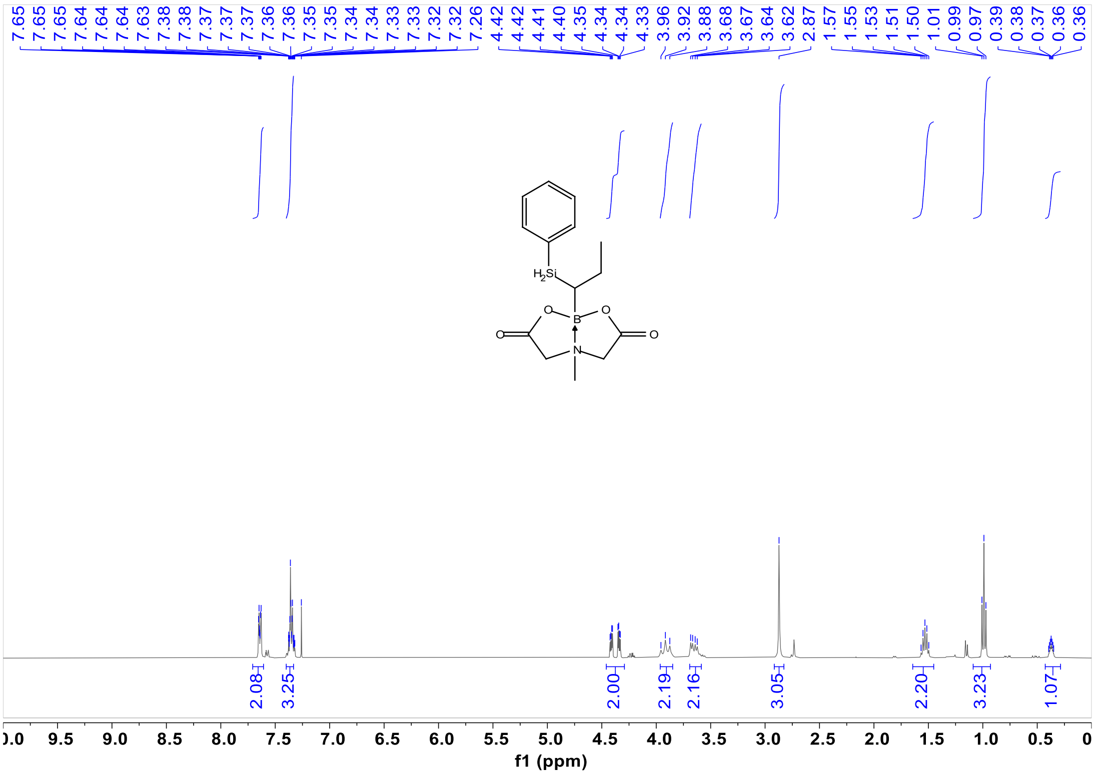

Supplementary Figure 186. ^{1}H NMR spectra for **58**

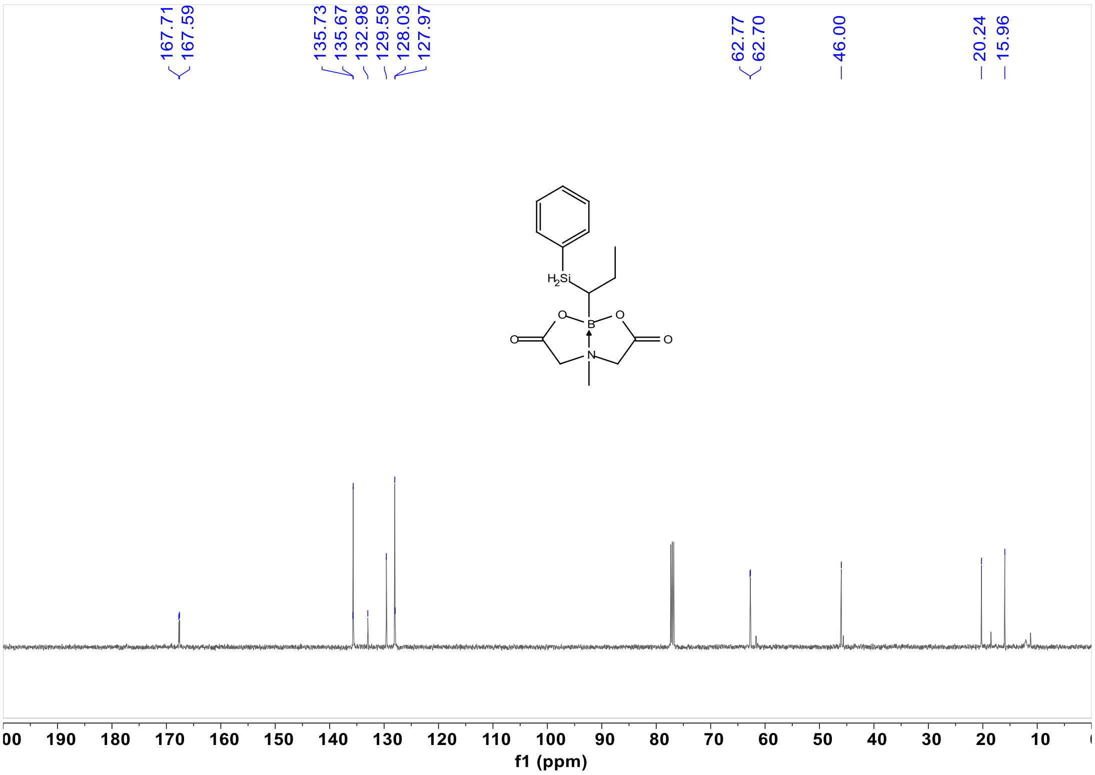

Supplementary Figure 187. ^{13}C NMR spectra for **58**

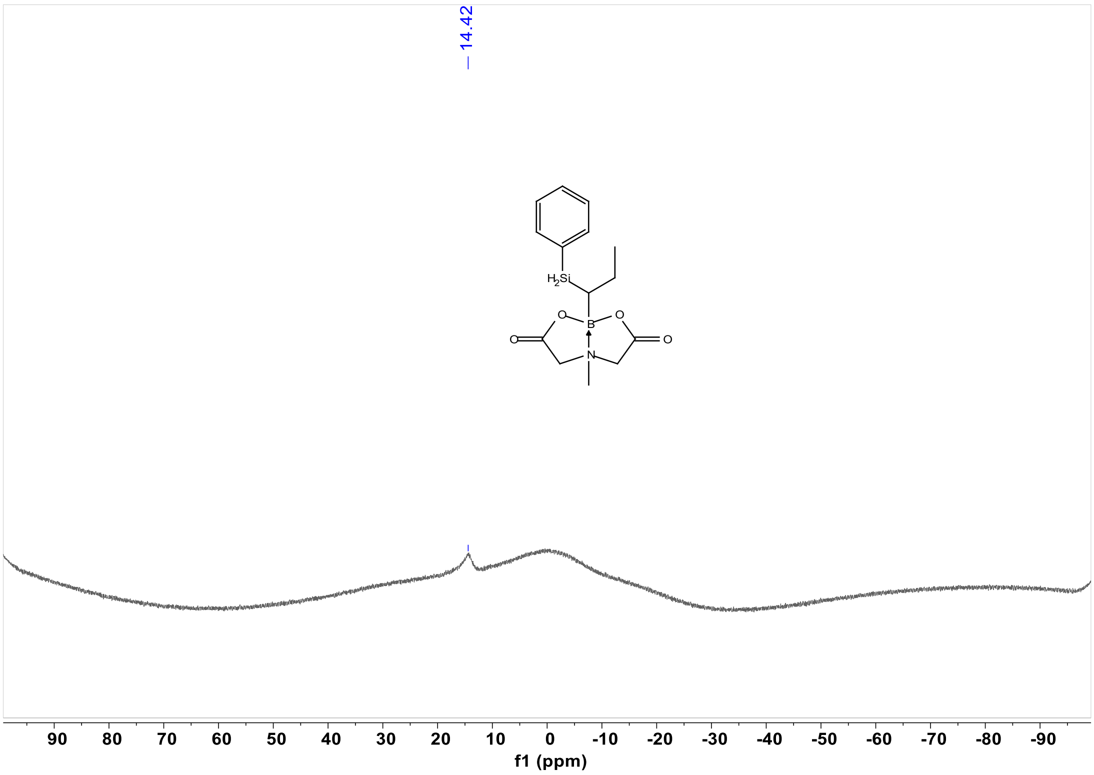

Supplementary Figure 188. ^{11}B NMR spectra for **58**

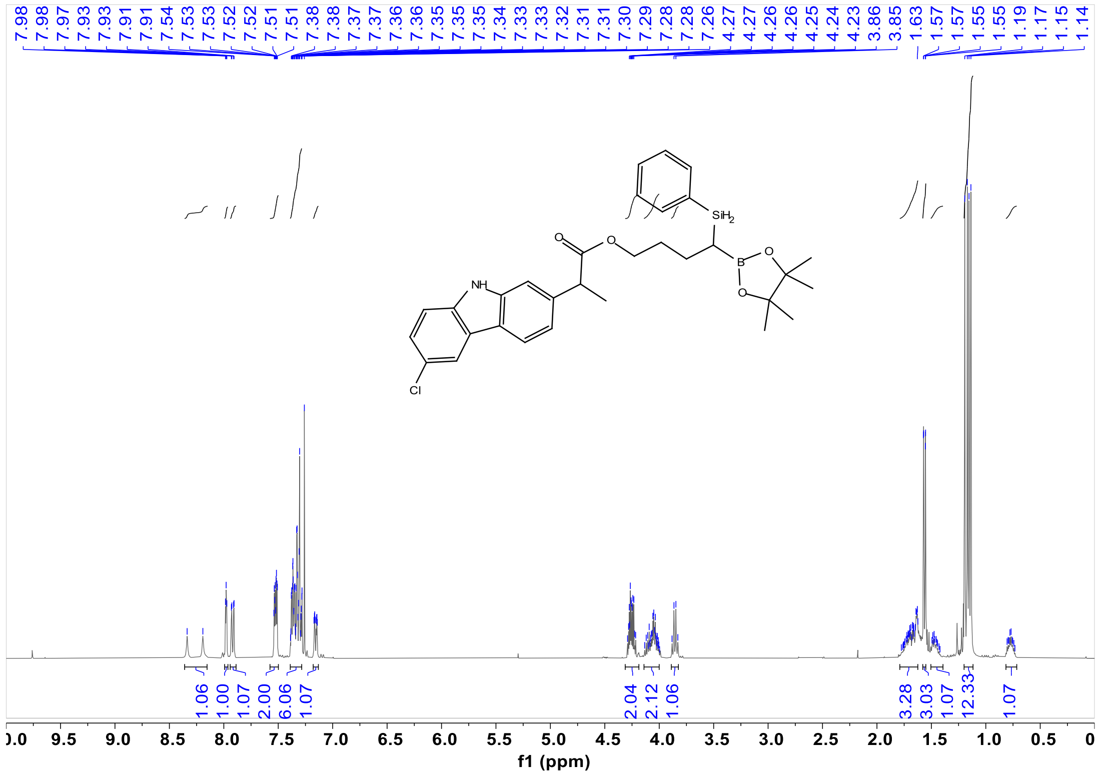

Supplementary Figure 189. ^{1}H NMR spectra for **59**

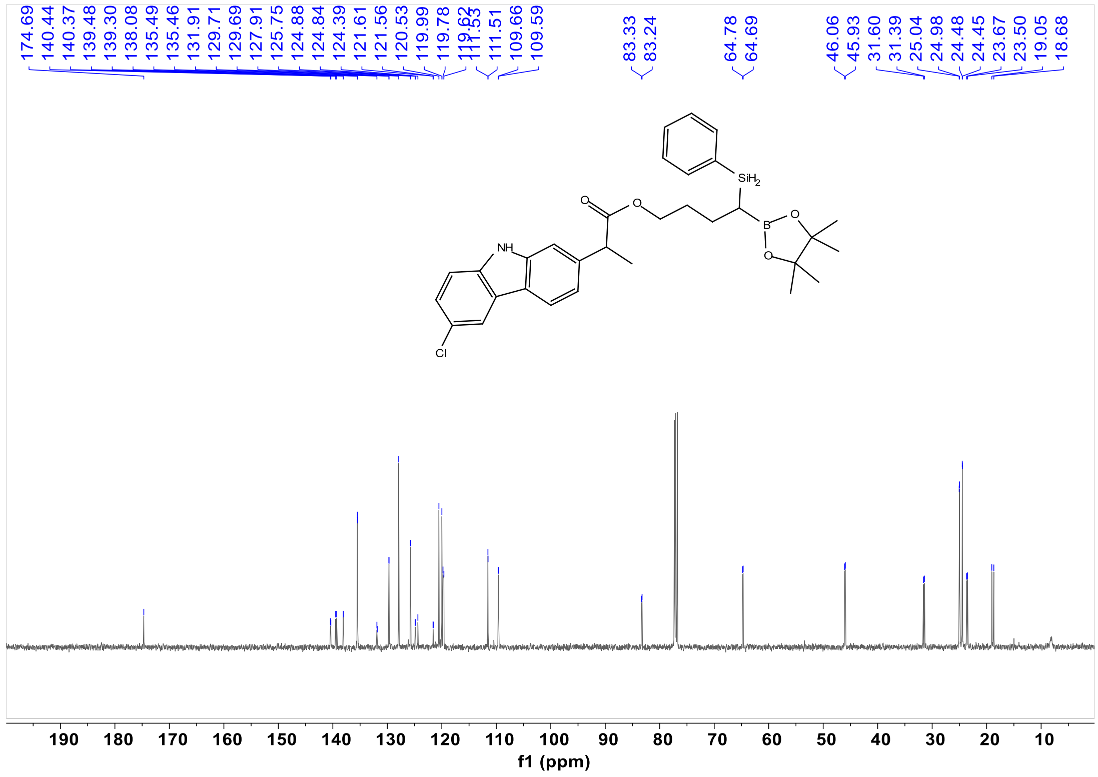

Supplementary Figure 190. ^{13}C NMR spectra for **59**

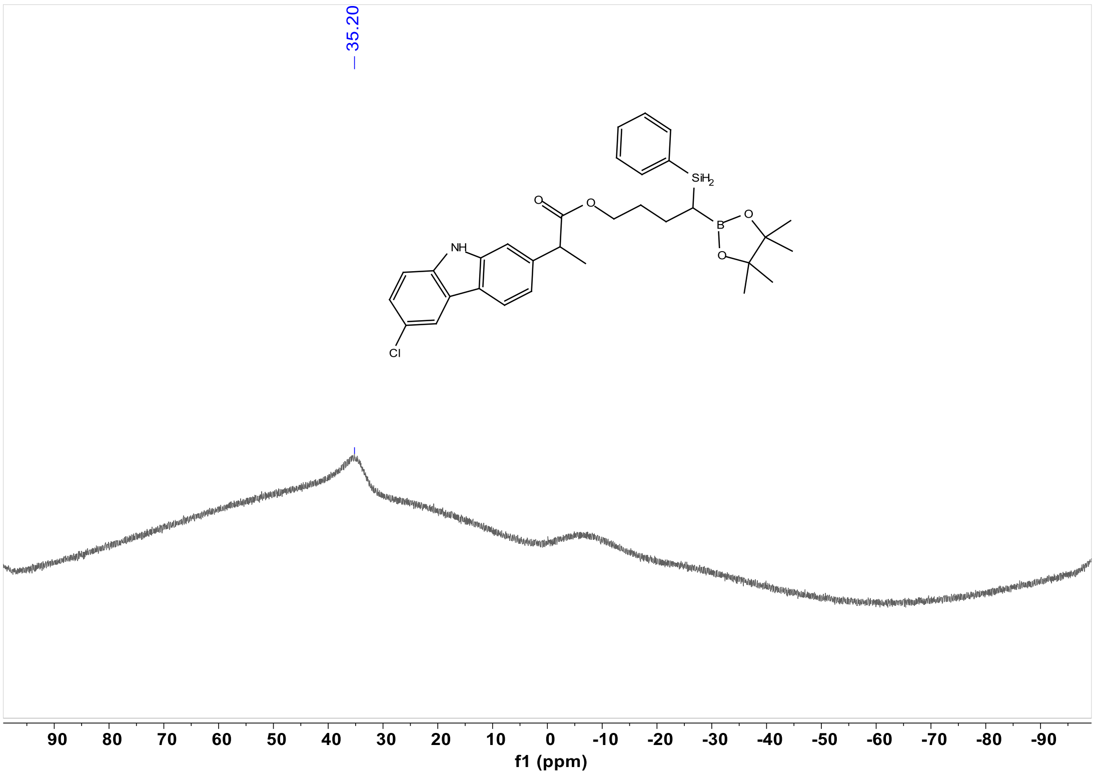

Supplementary Figure 191. ^{11}B NMR spectra for **59**

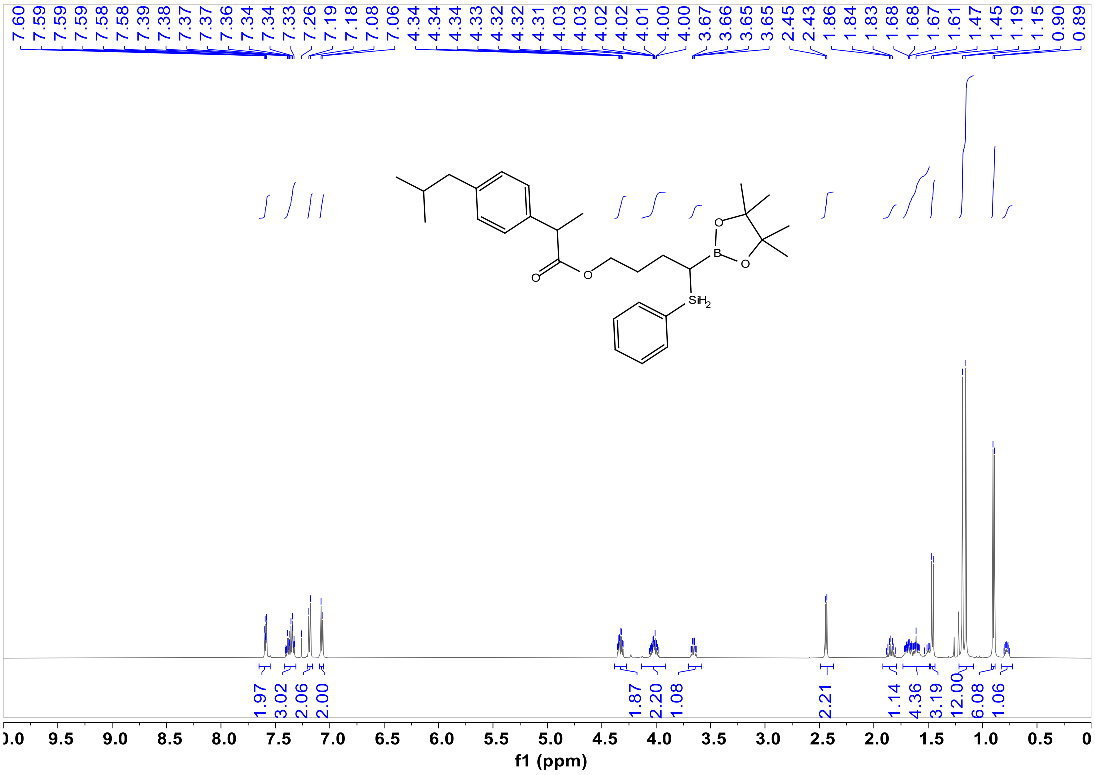

Supplementary Figure 192. ^{1}H NMR spectra for **60**

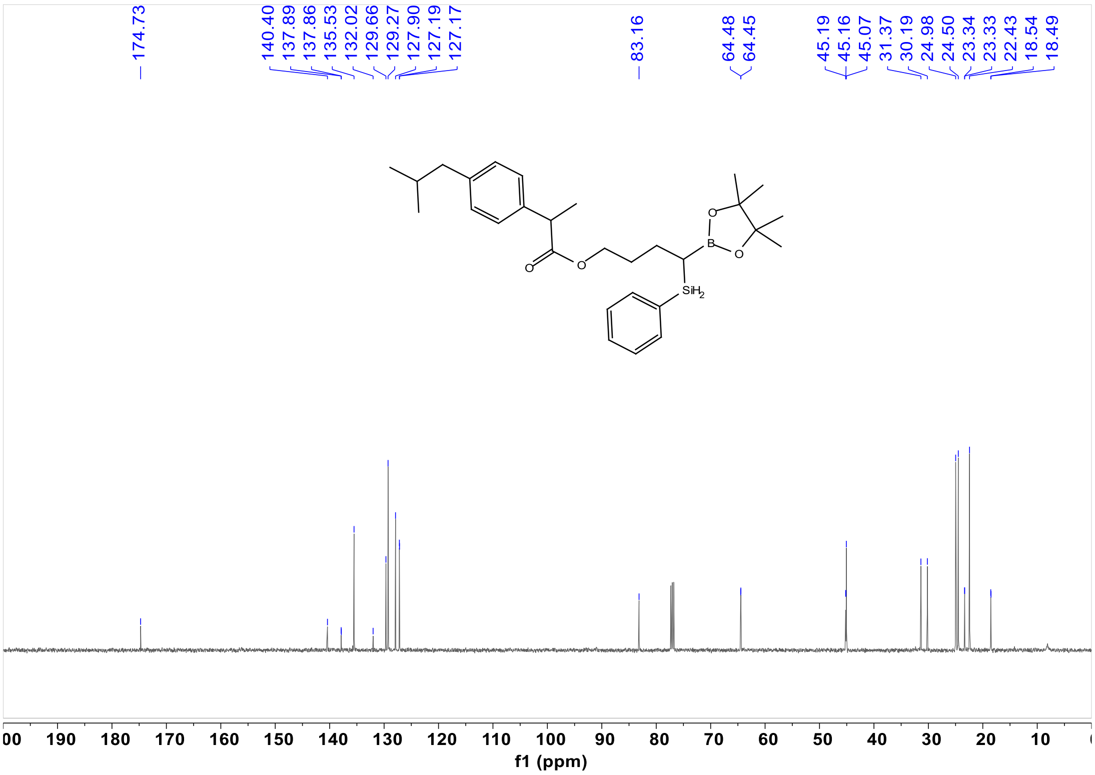

Supplementary Figure 193. ^{13}C NMR spectra for **60**

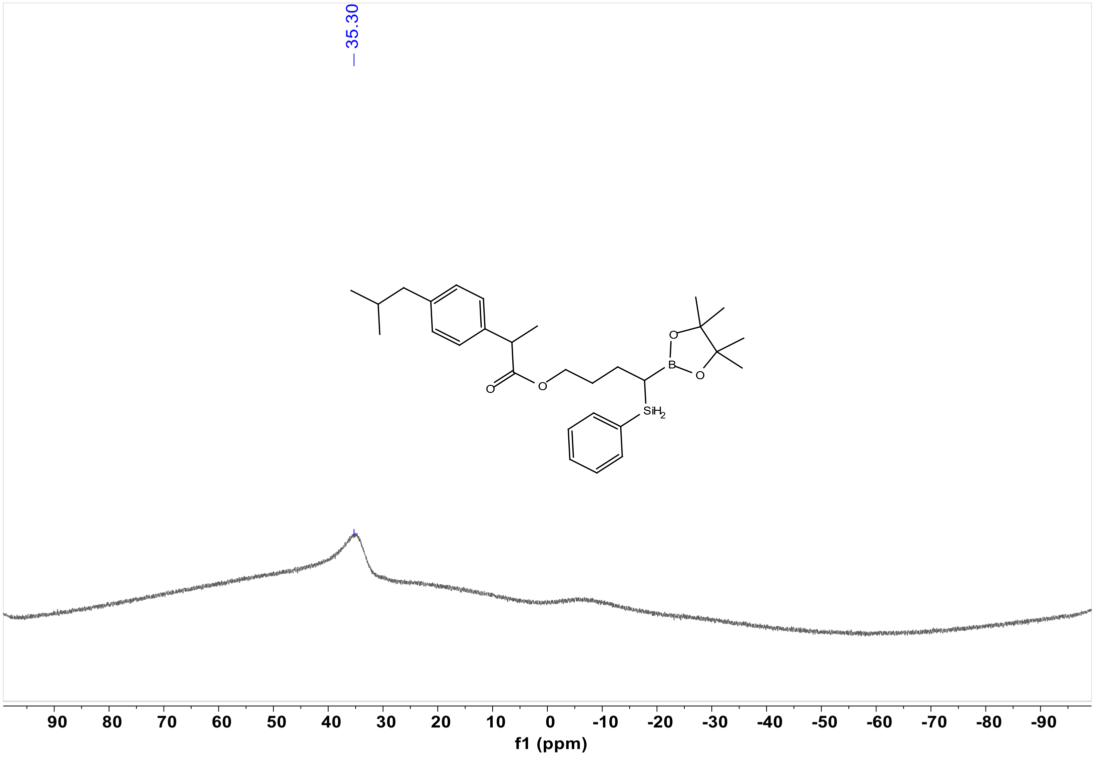

Supplementary Figure 194. ^{11}B NMR spectra for **60**

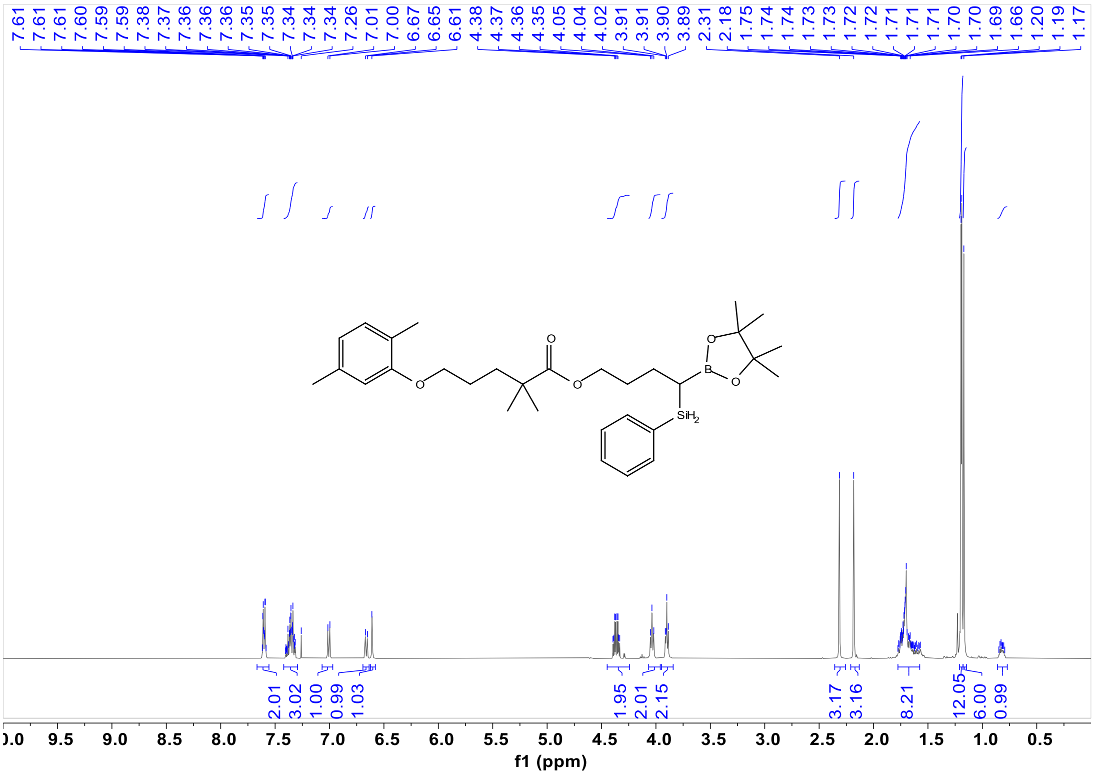

Supplementary Figure 195. ^{1}H NMR spectra for **61**

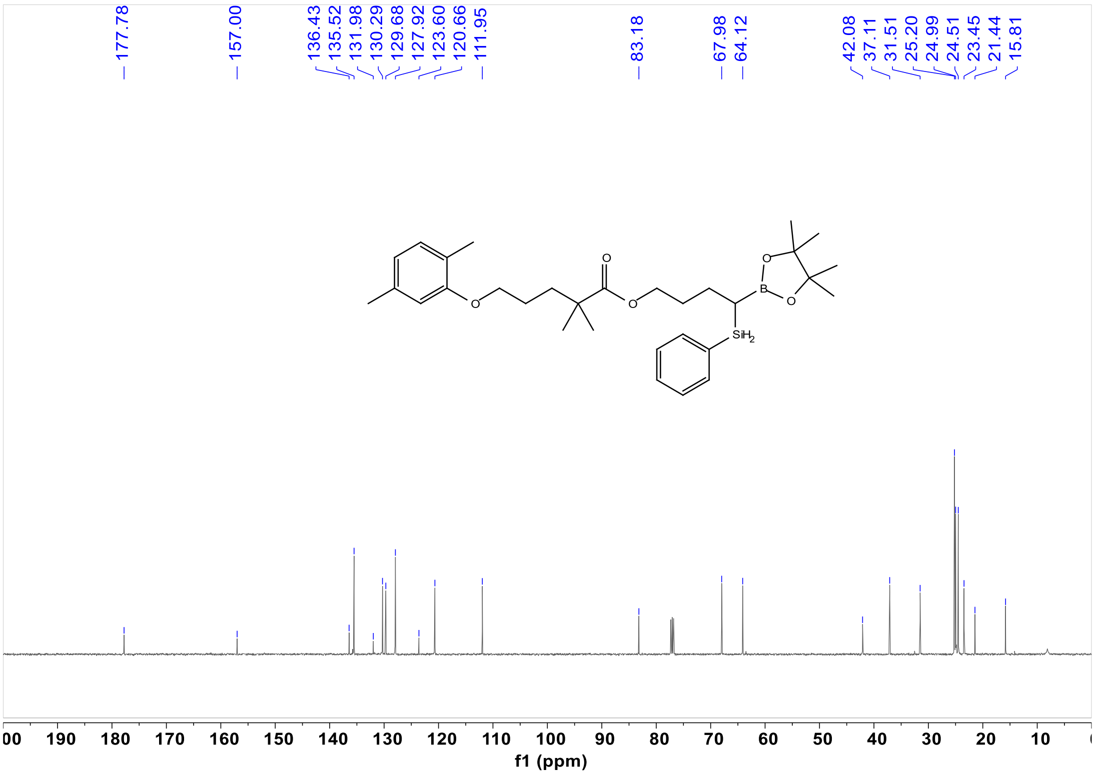

Supplementary Figure 196. ^{13}C NMR spectra for **61**

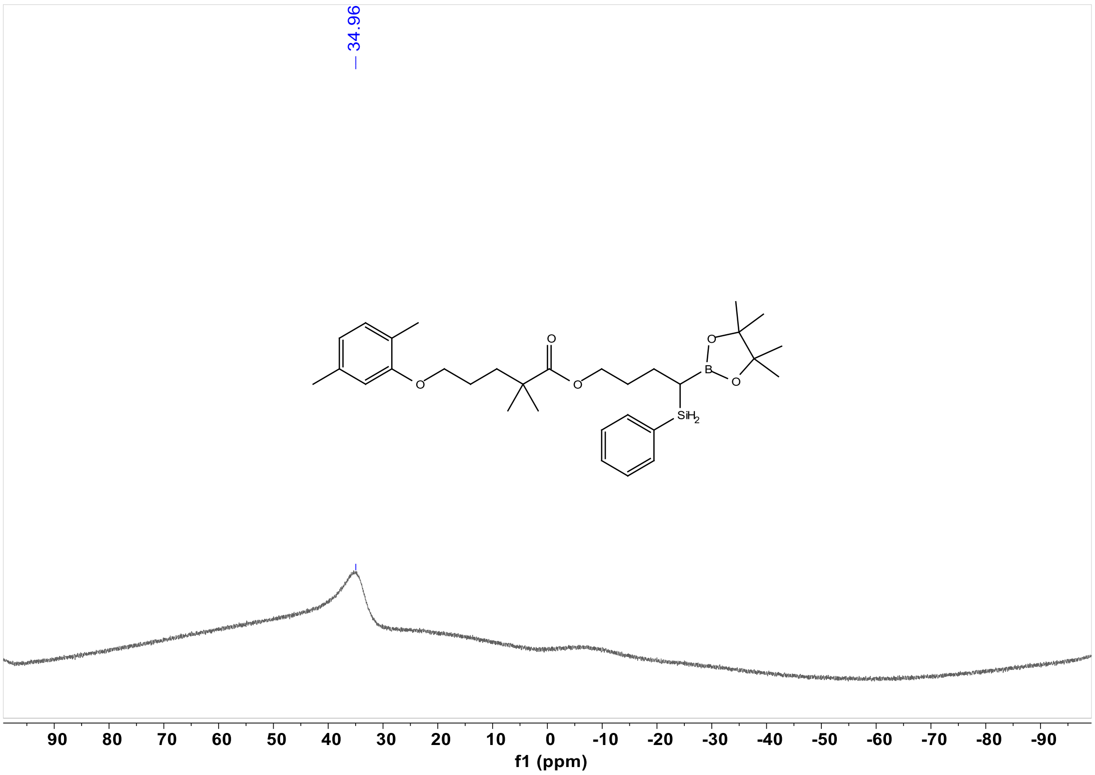

Supplementary Figure 197. ^{11}B NMR spectra for **61**

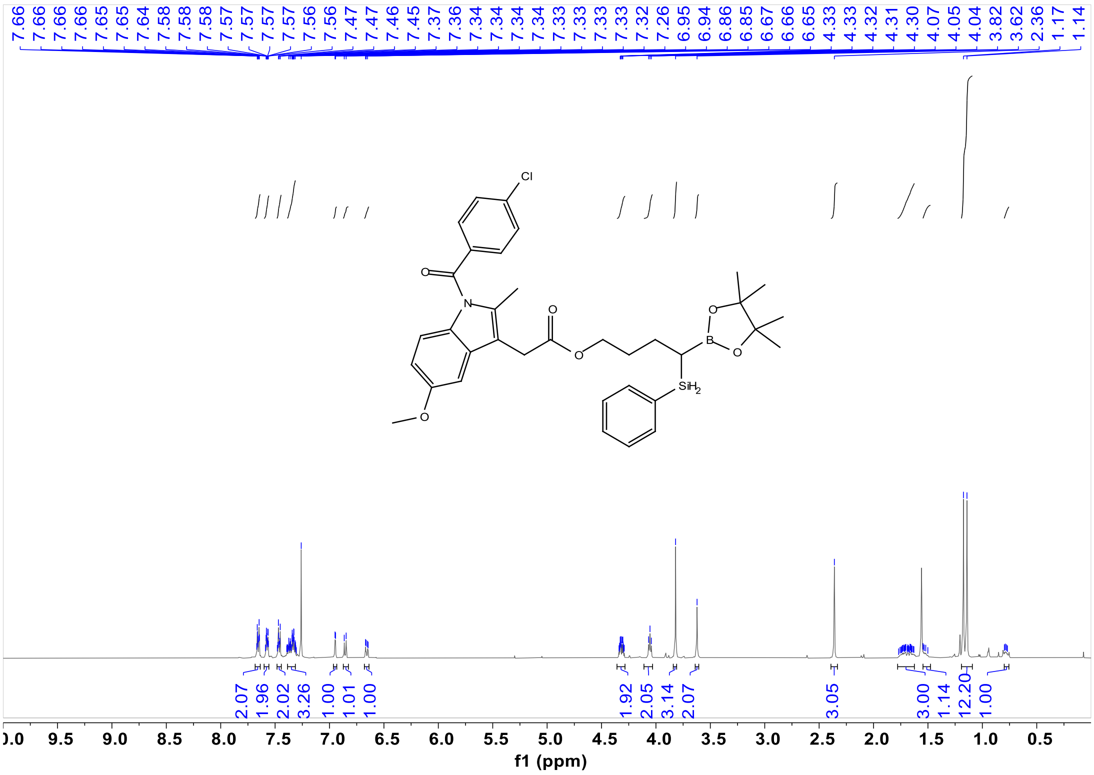

Supplementary Figure 198. ^{1}H NMR spectra for **62**

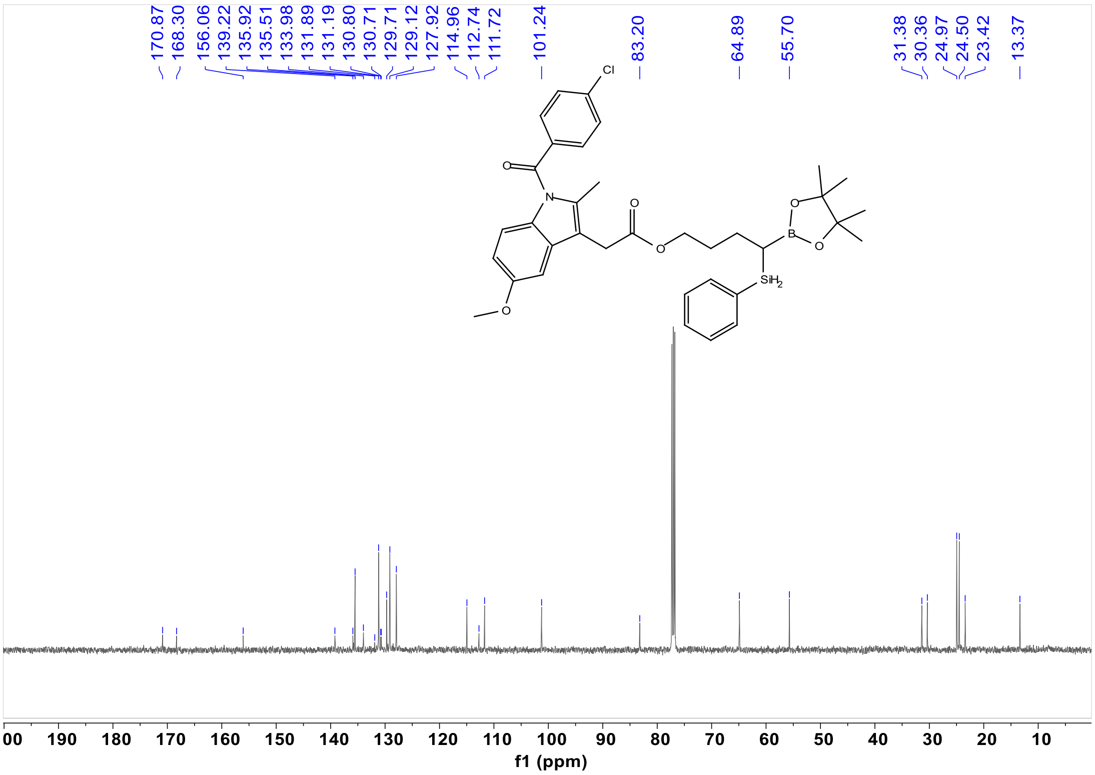

Supplementary Figure 199. ^{13}C NMR spectra for **62**

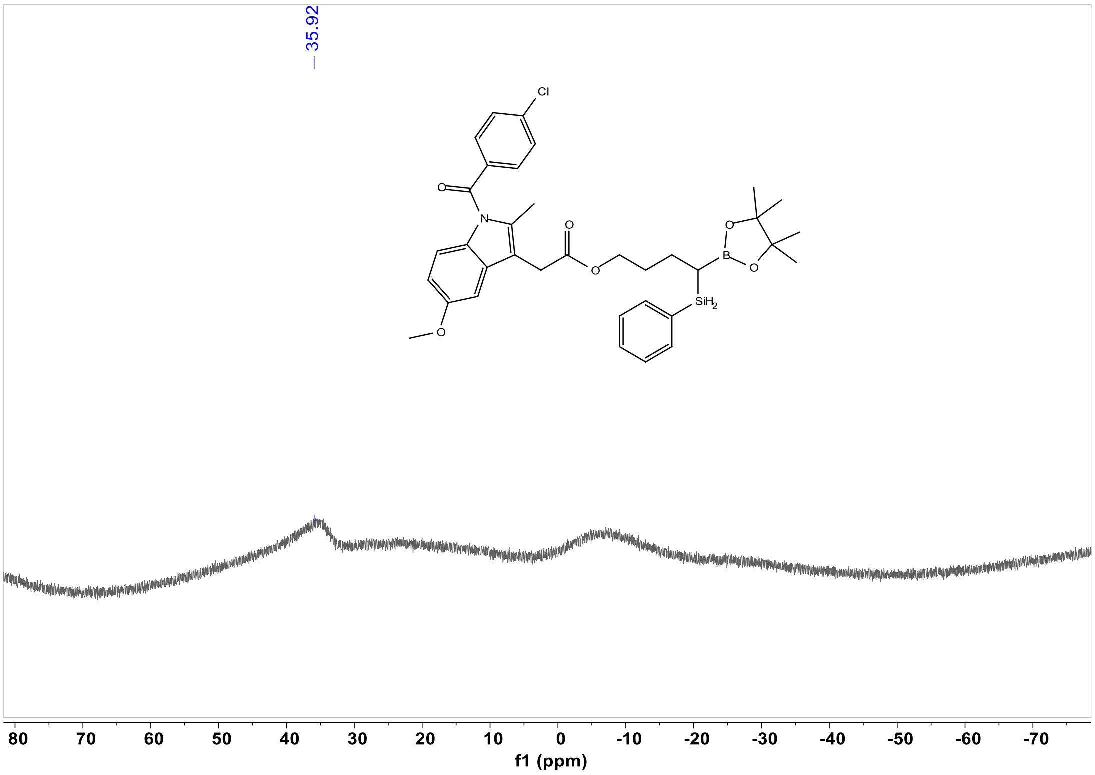

Supplementary Figure 200. ^{11}B NMR spectra for **62**

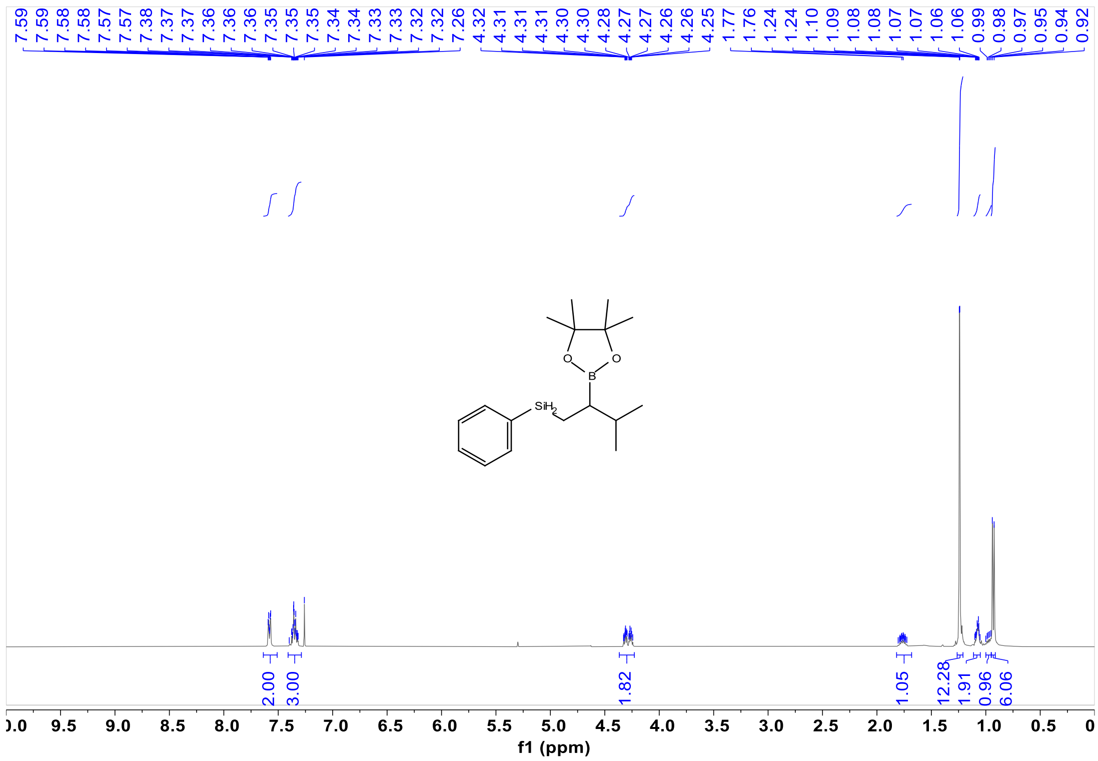

Supplementary Figure 201. ^{1}H NMR spectra for **63**

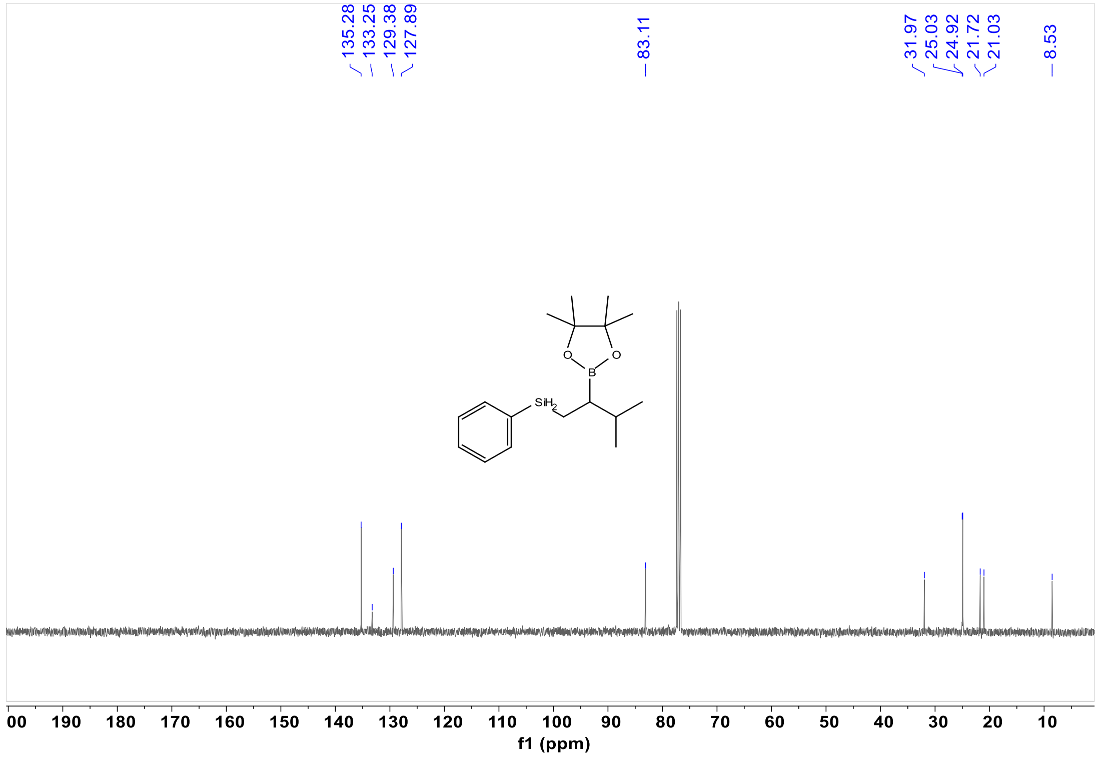

Supplementary Figure 202. ^{13}C NMR spectra for **63**

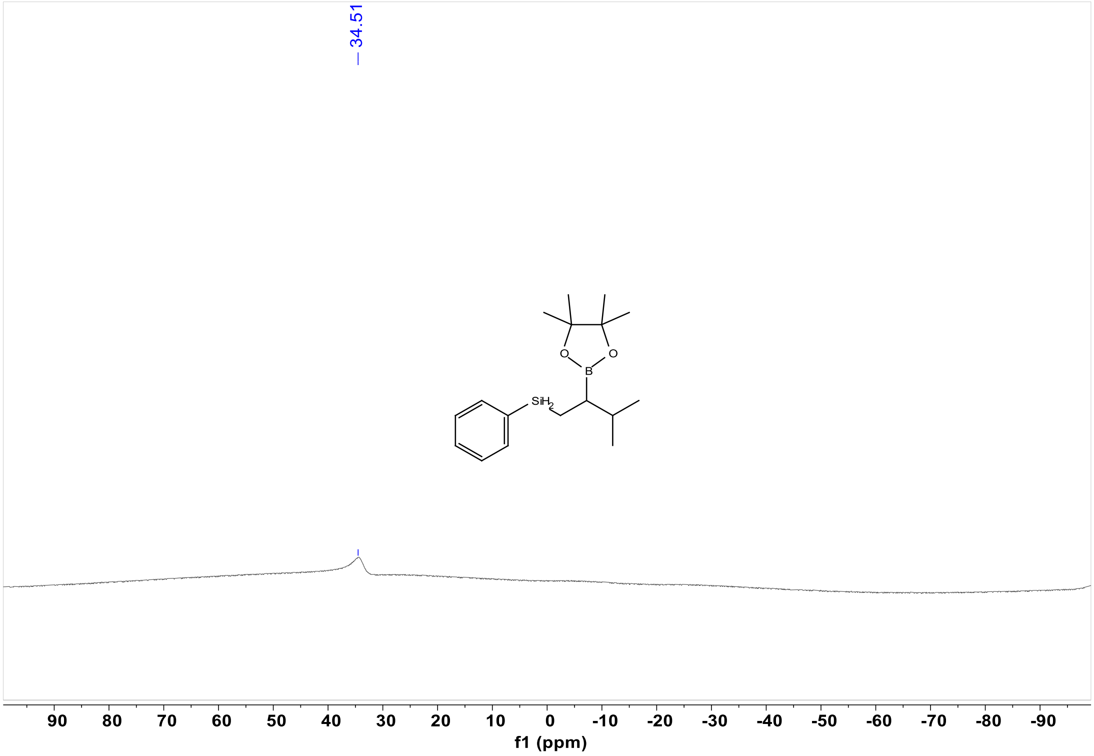

Supplementary Figure 203. ^{11}B NMR spectra for **63**

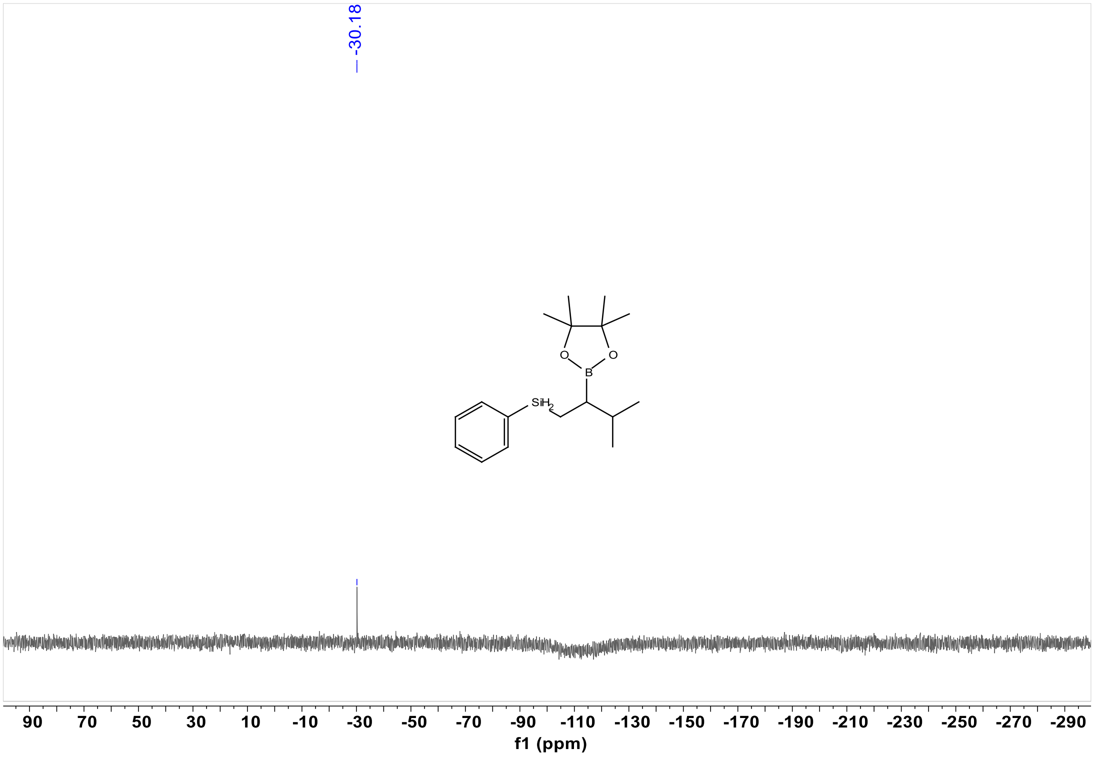

Supplementary Figure 204. ^{29}Si NMR spectra for **63**

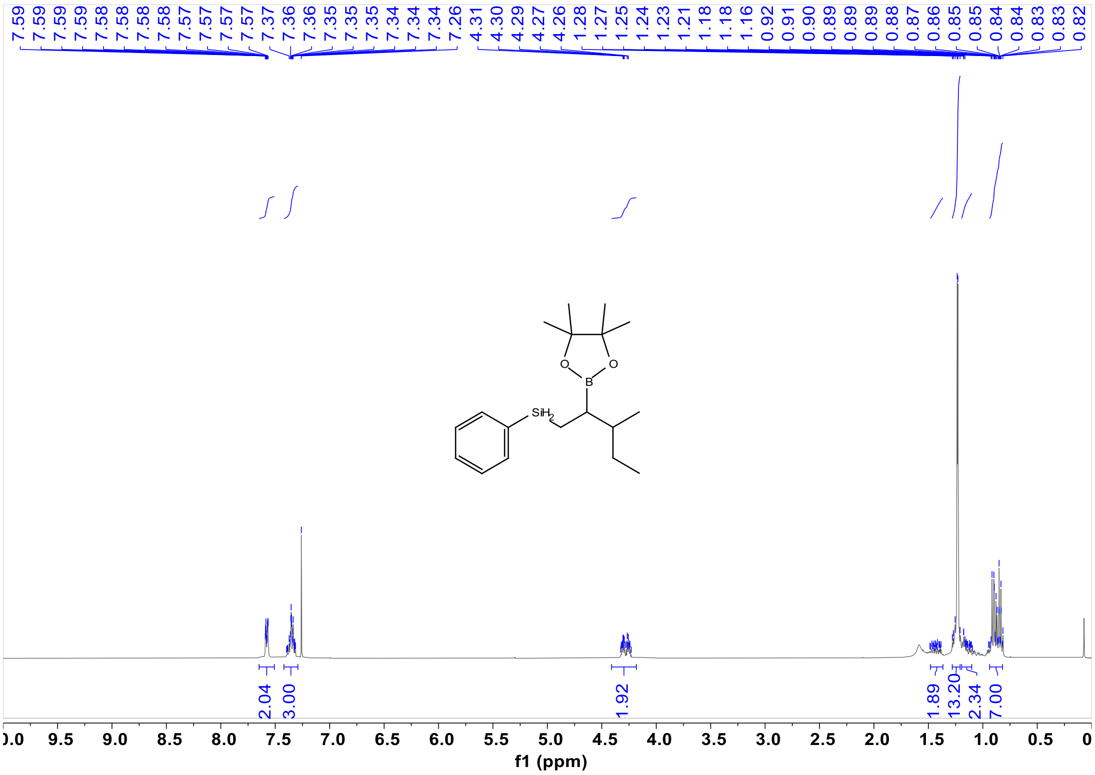

Supplementary Figure 205. ^{1}H NMR spectra for **64**

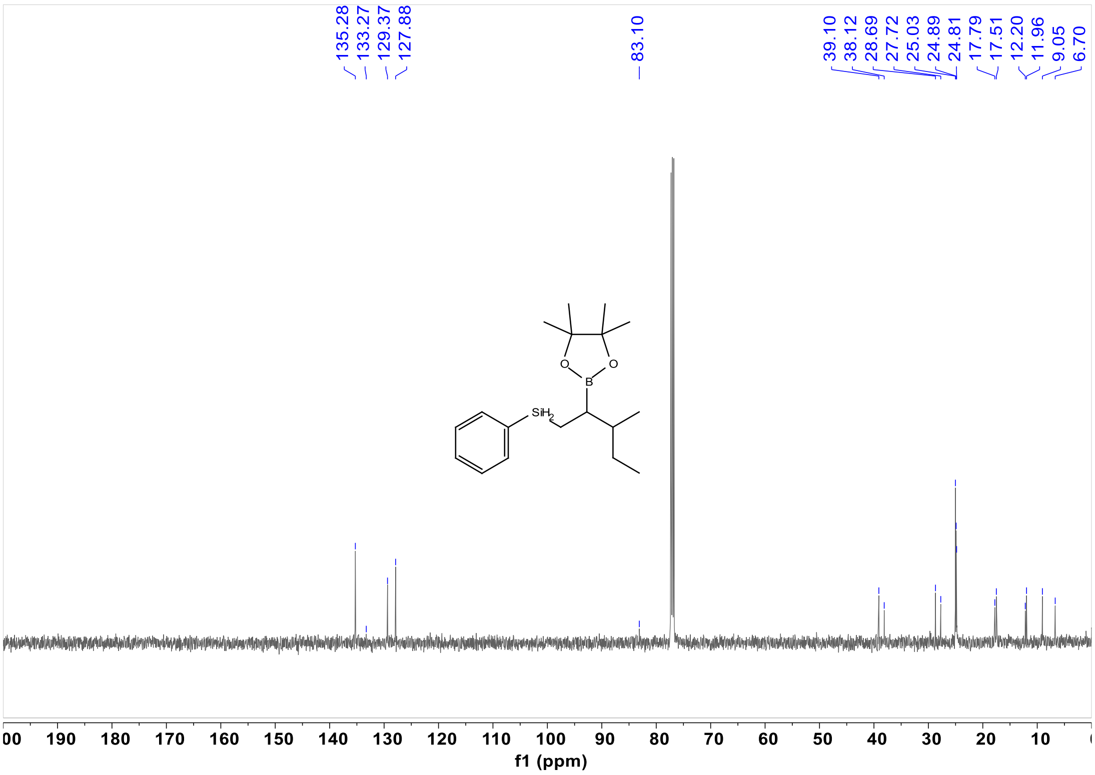

Supplementary Figure 206. ^{13}C NMR spectra for **64**

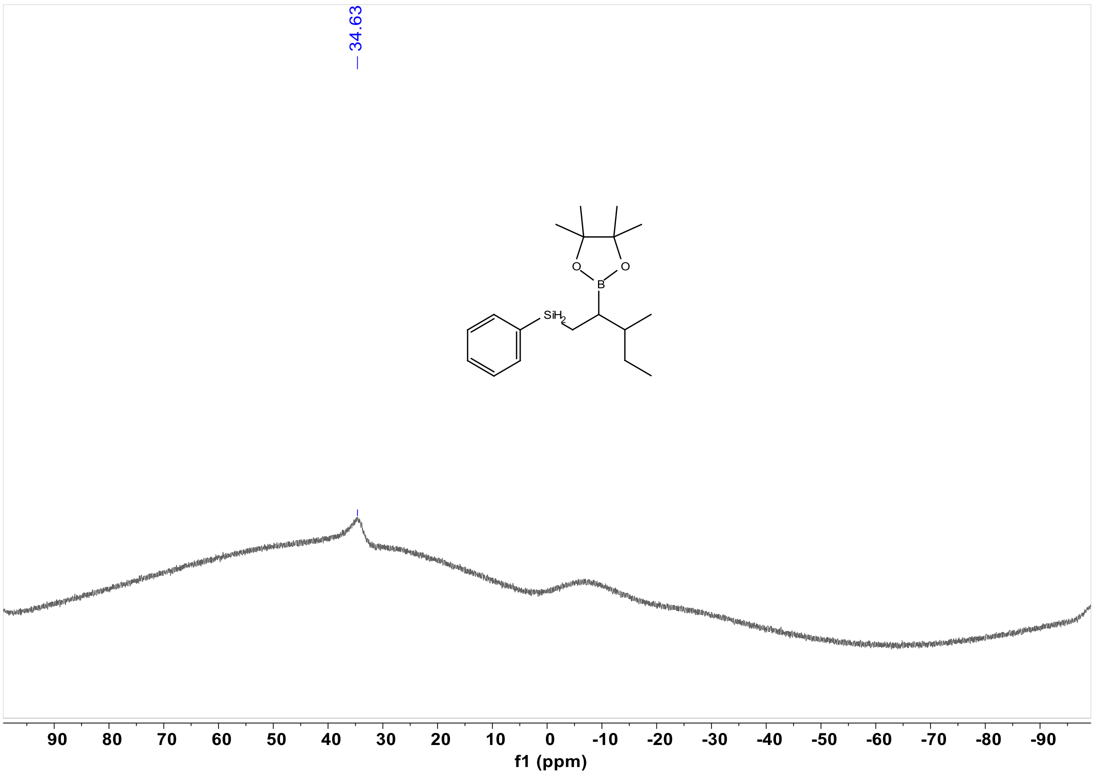

Supplementary Figure 207. ^{11}B NMR spectra for **64**

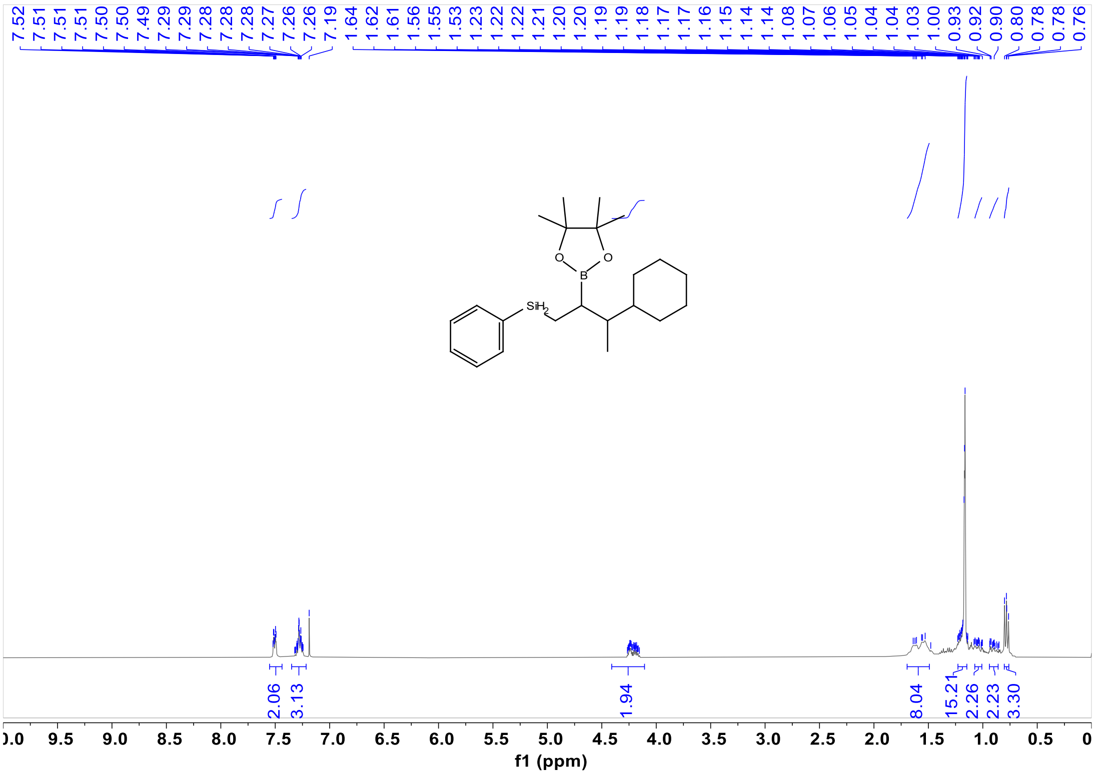

Supplementary Figure 208. ^{1}H NMR spectra for **65**

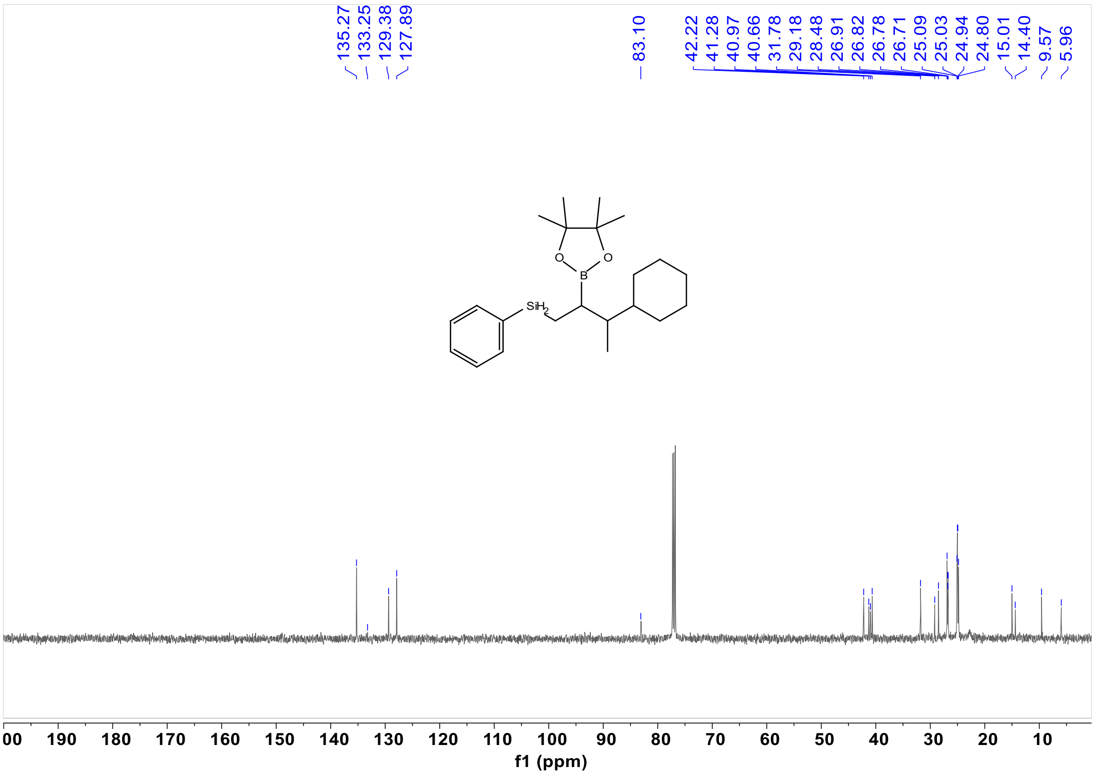

Supplementary Figure 209. ^{13}C NMR spectra for **65**

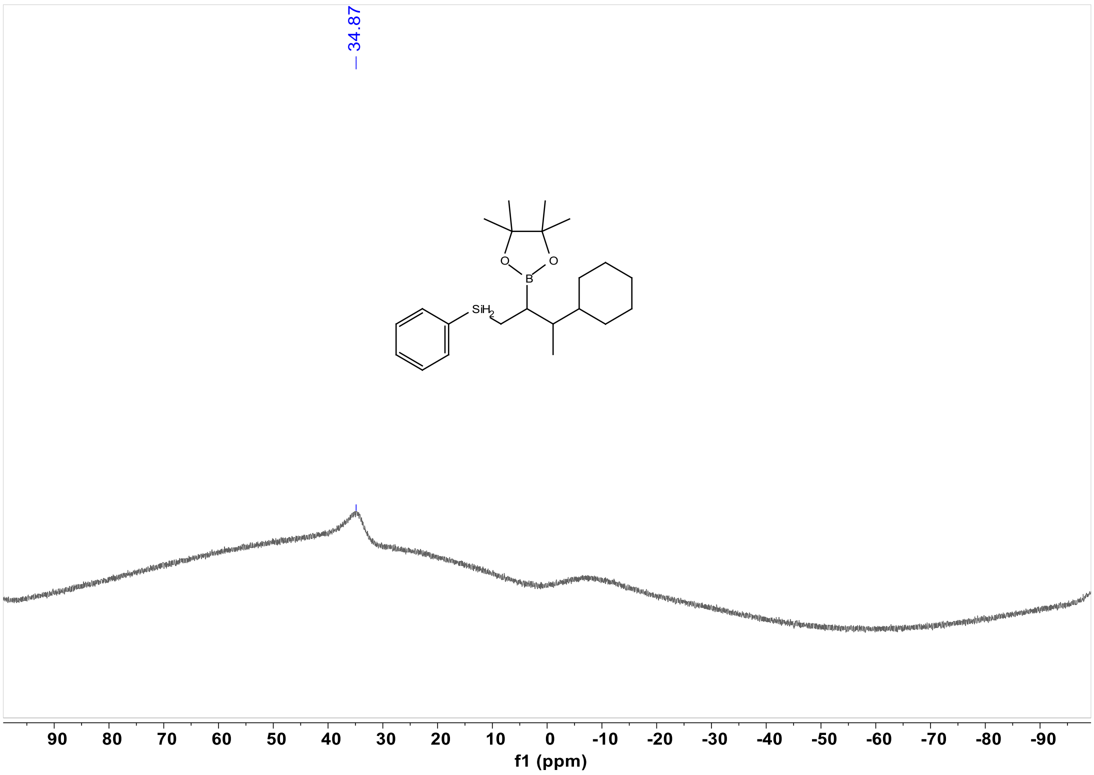

Supplementary Figure 210. ^{11}B NMR spectra for **65**

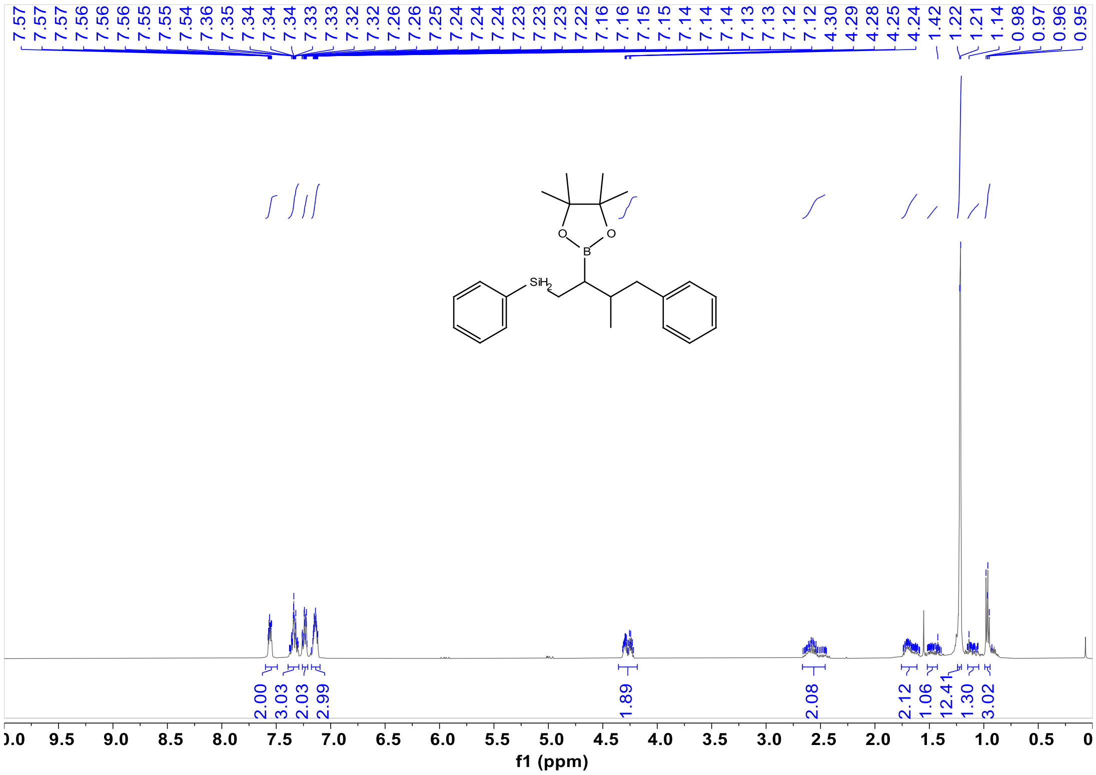

Supplementary Figure 211. ^{1}H NMR spectra for **66**

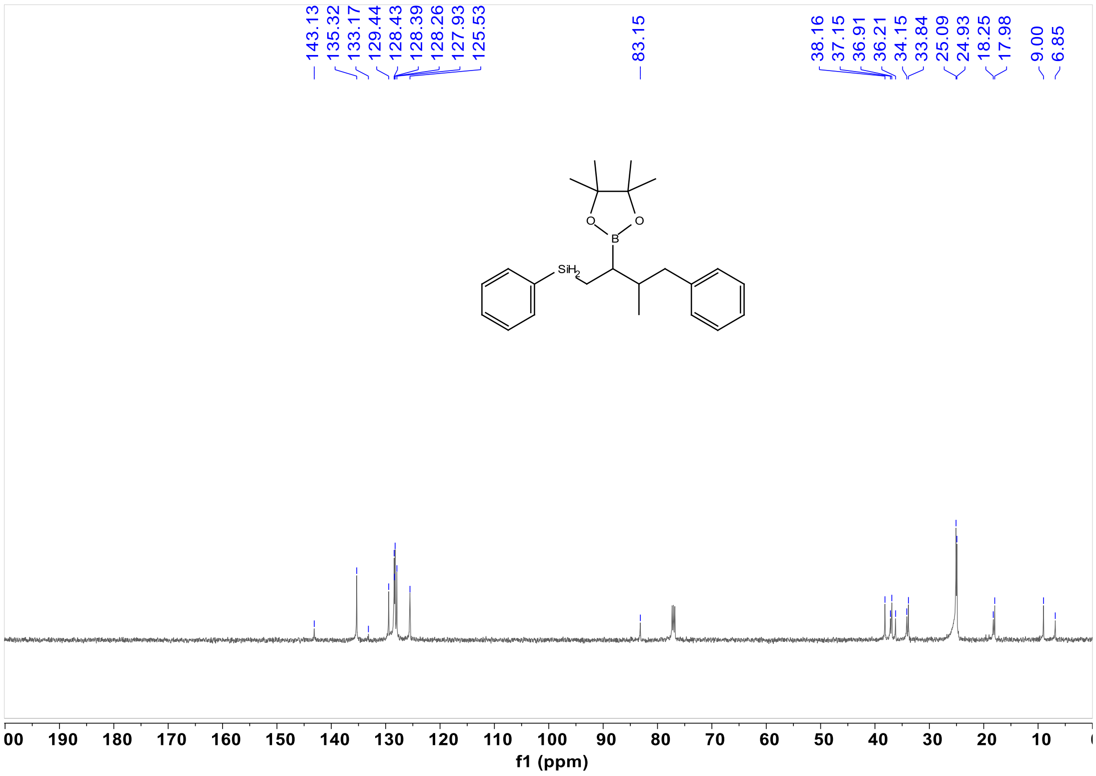

Supplementary Figure 212. ^{13}C NMR spectra for **66**

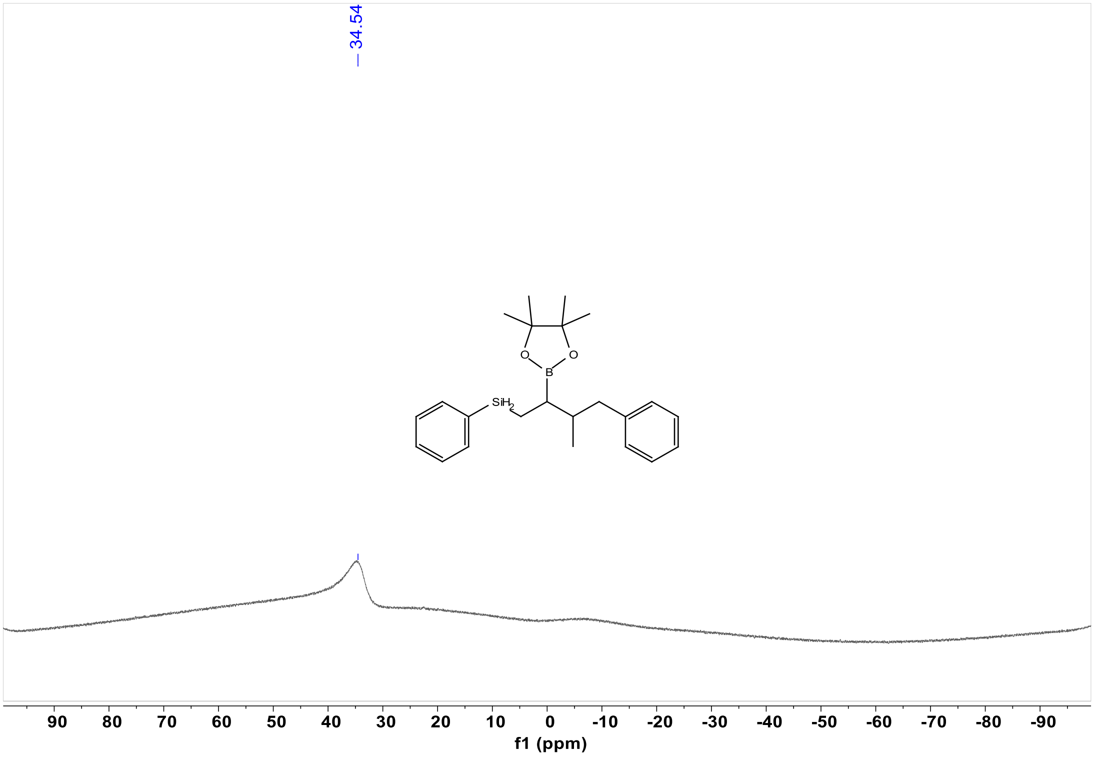

Supplementary Figure 213. ^{11}B NMR spectra for **66**

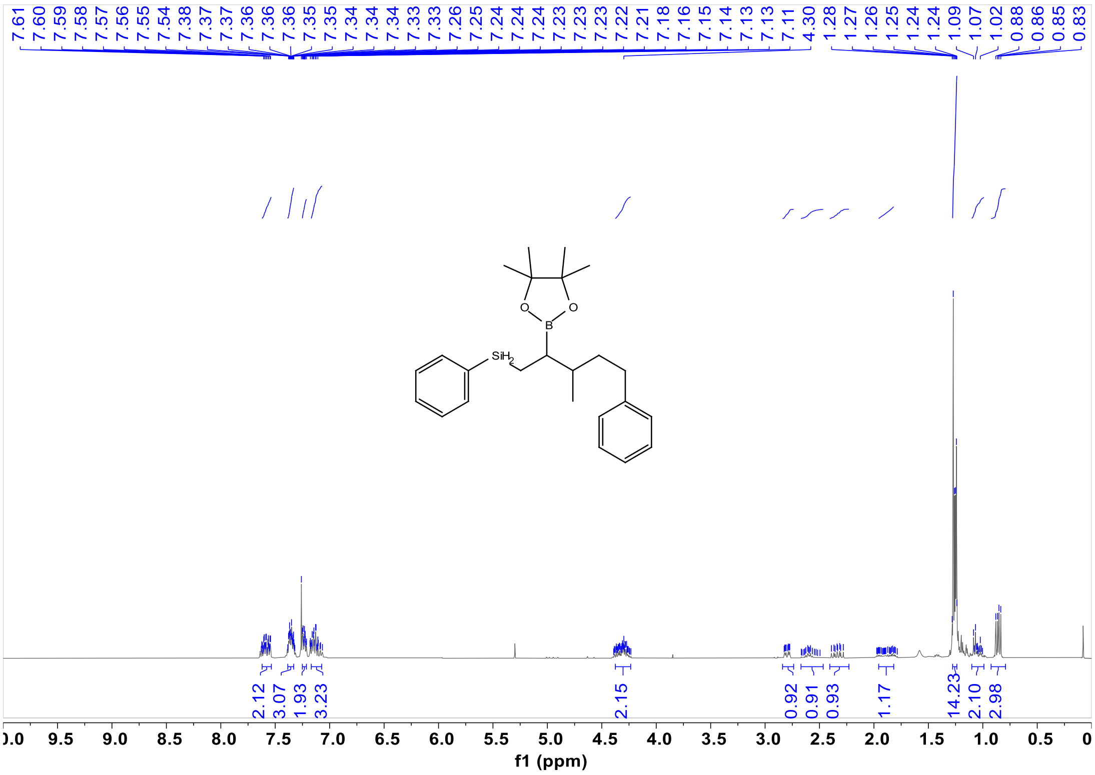

Supplementary Figure 214. ^{1}H NMR spectra for **67**

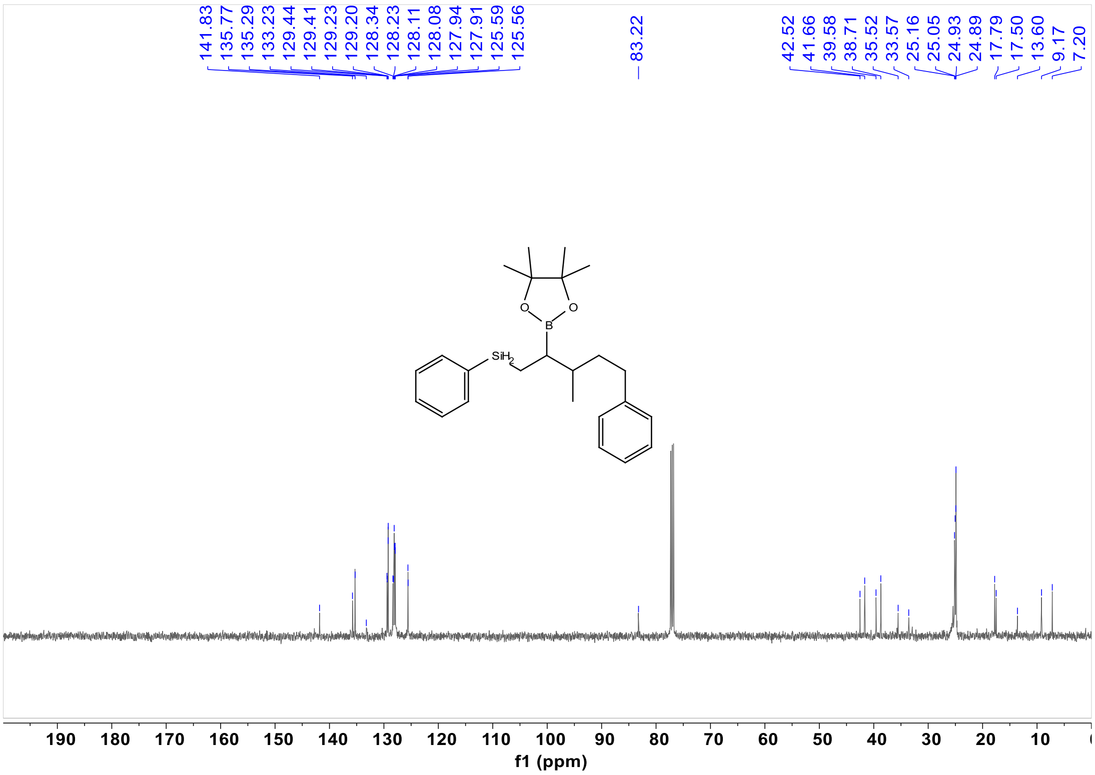

Supplementary Figure 215. ^{13}C NMR spectra for **67**

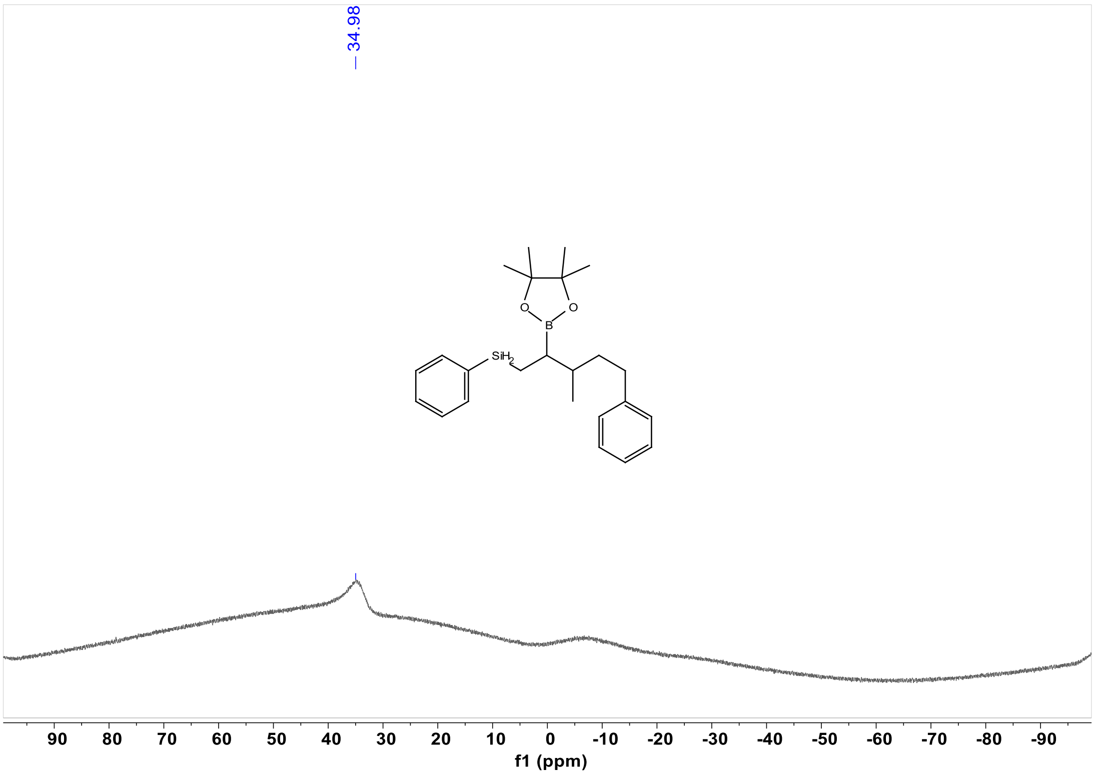

Supplementary Figure 216. ^{11}B NMR spectra for **67**

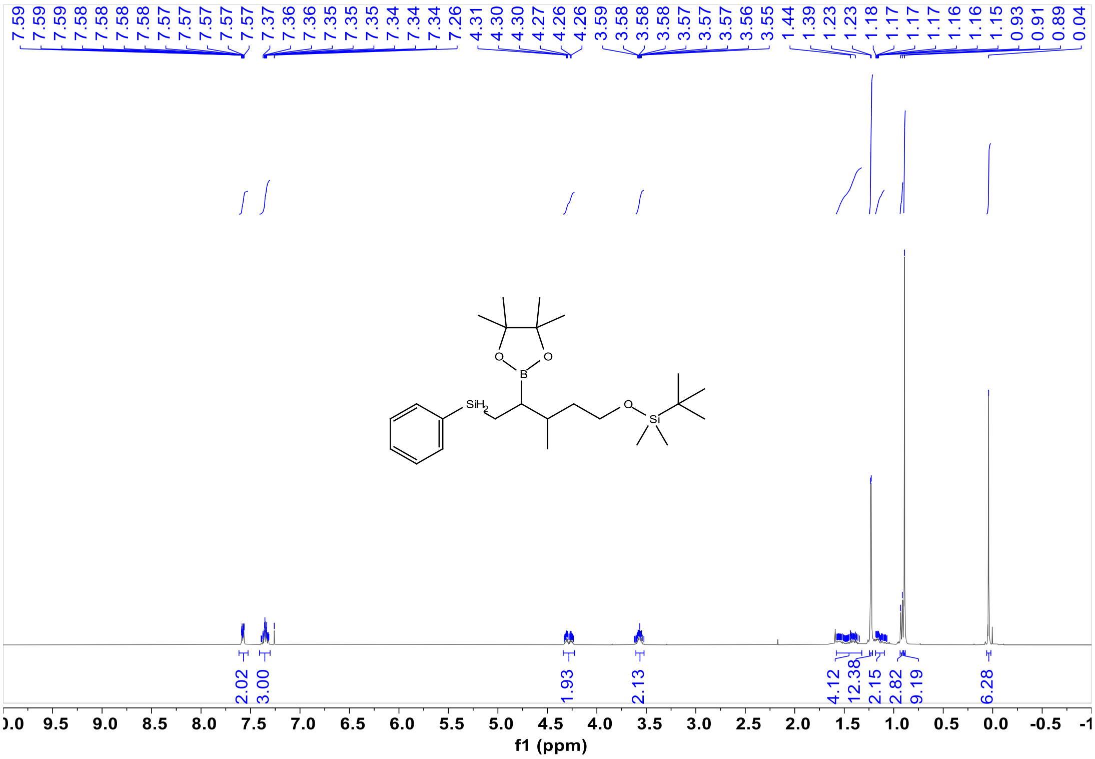

Supplementary Figure 217. ^{1}H NMR spectra for **68**

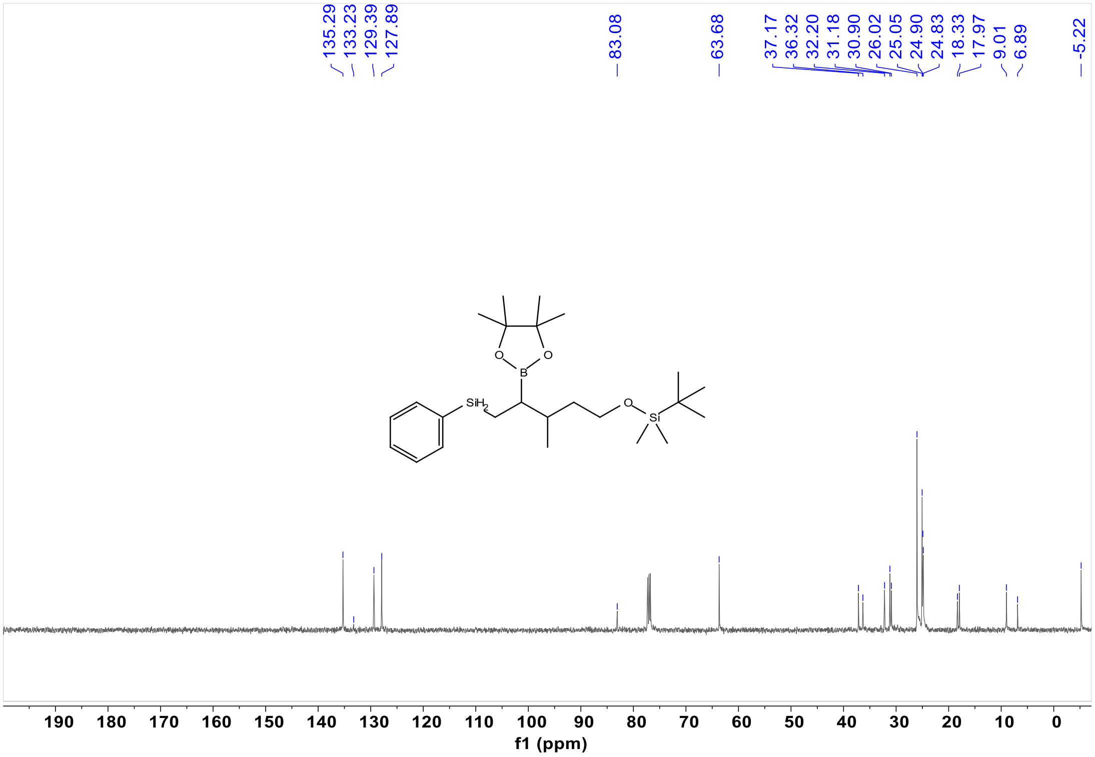

Supplementary Figure 218. ^{13}C NMR spectra for **68**

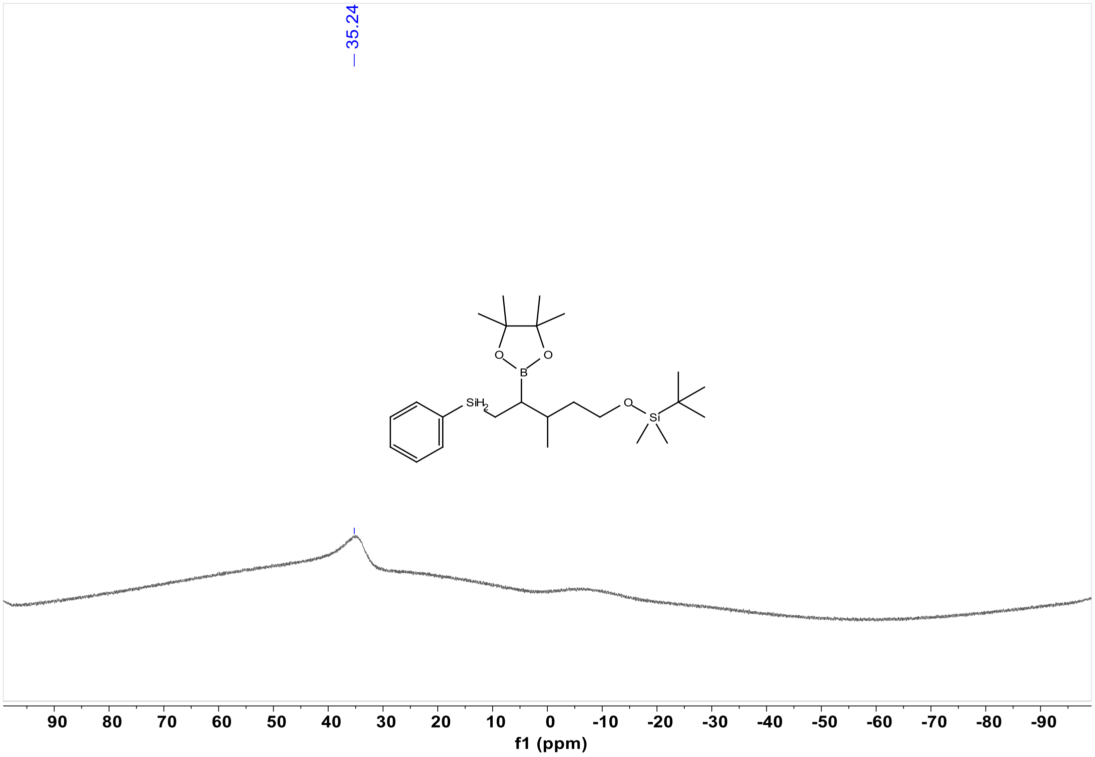

Supplementary Figure 219. ^{11}B NMR spectra for **68**

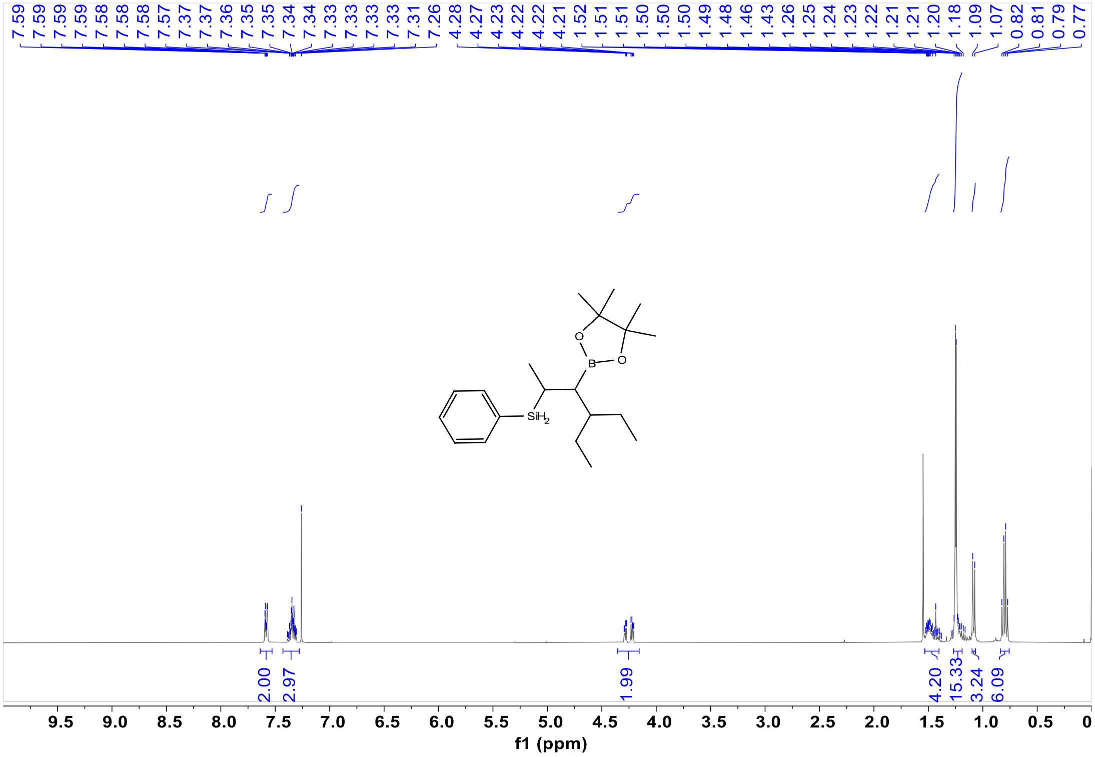

Supplementary Figure 220. ^{1}H NMR spectra for **69**

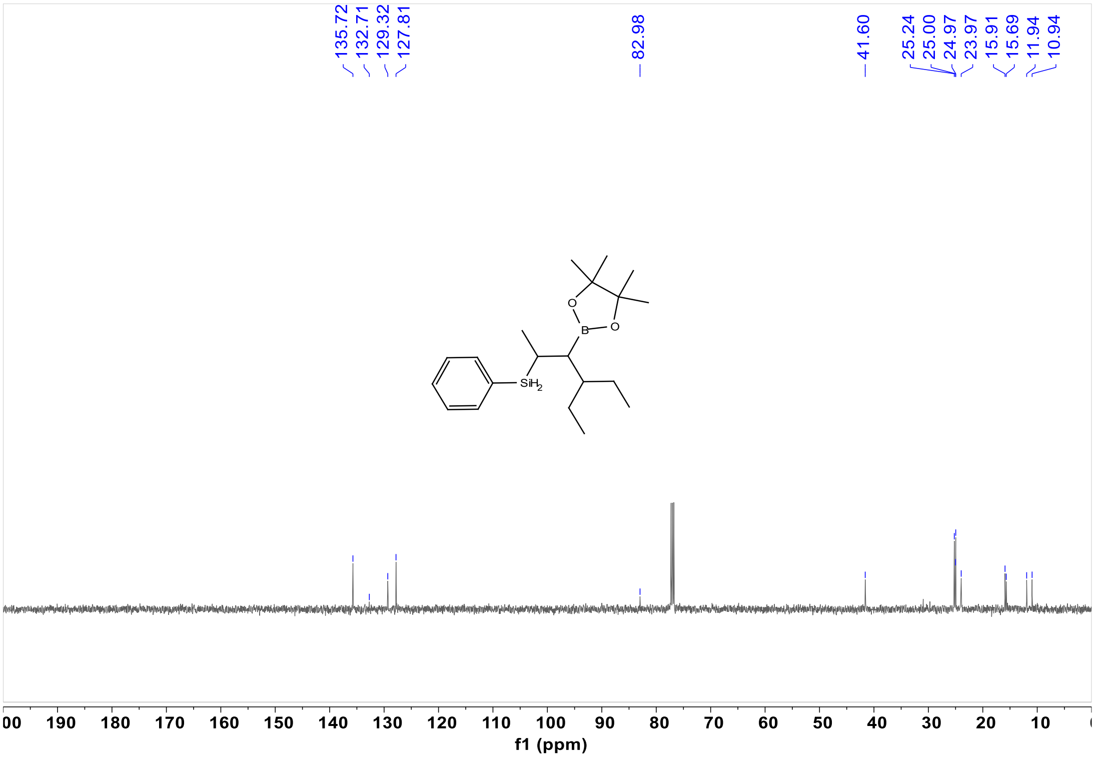

Supplementary Figure 221. ^{13}C NMR spectra for **69**

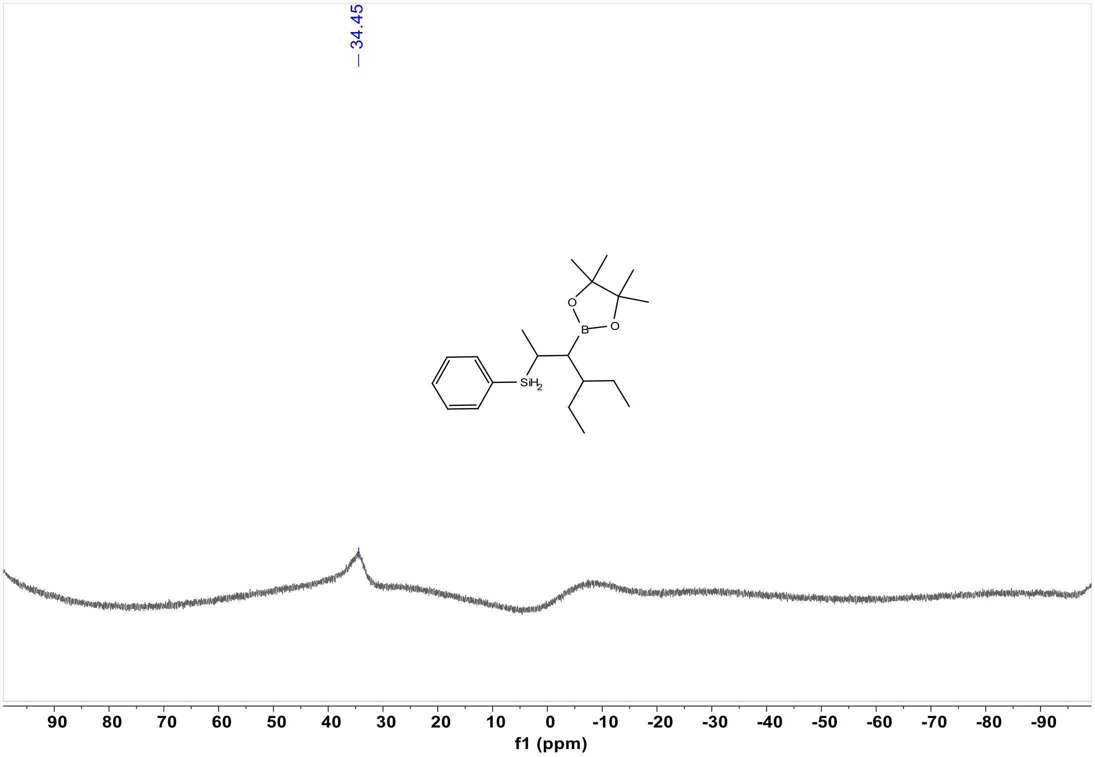

Supplementary Figure 222. ^{11}B NMR spectra for **69**

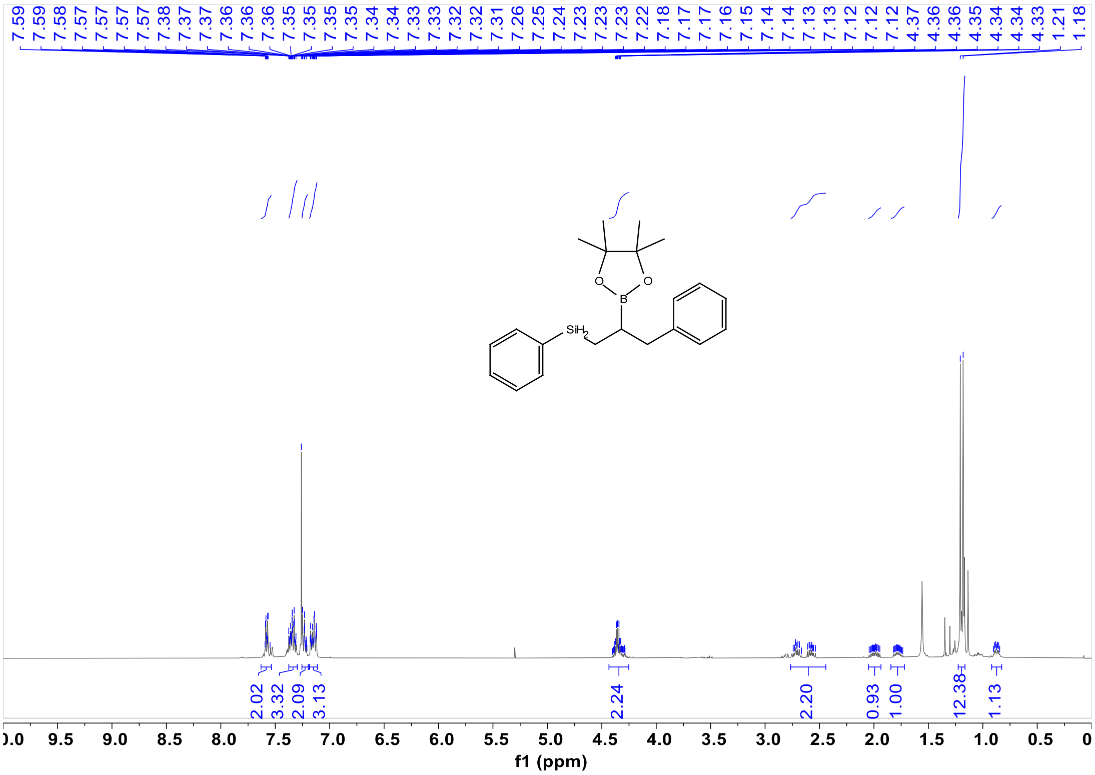

Supplementary Figure 223. ^{1}H NMR spectra for **70**

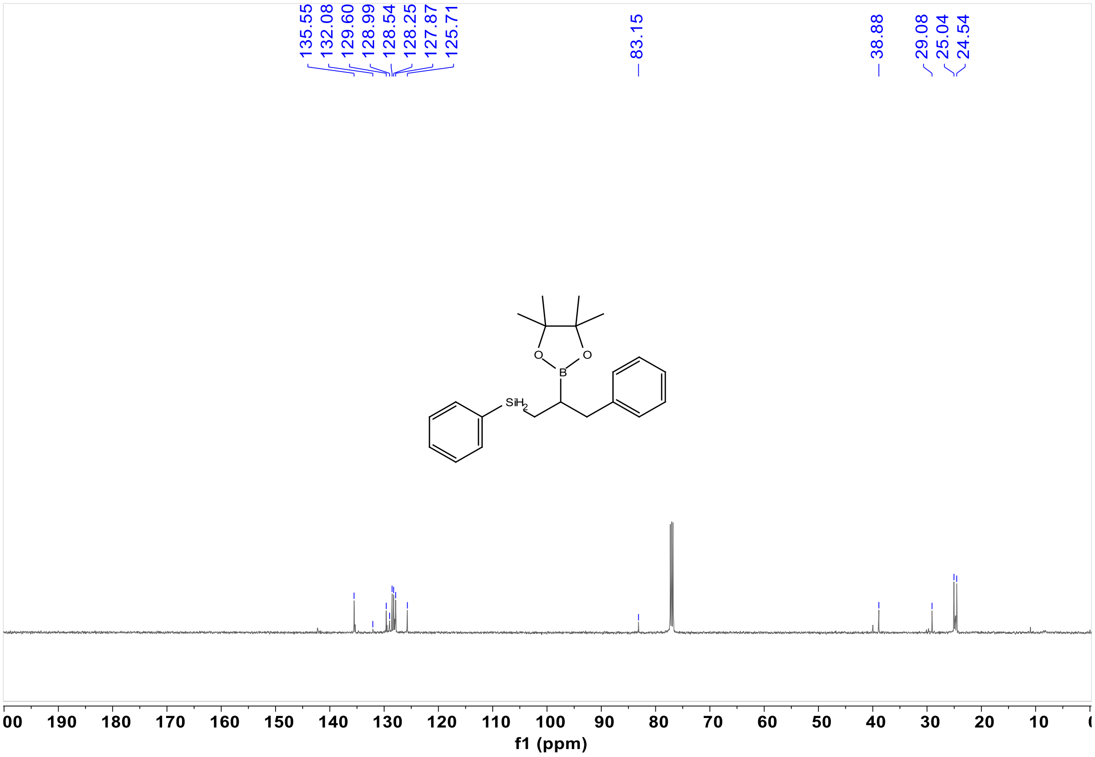

Supplementary Figure 224. ^{13}C NMR spectra for **70**

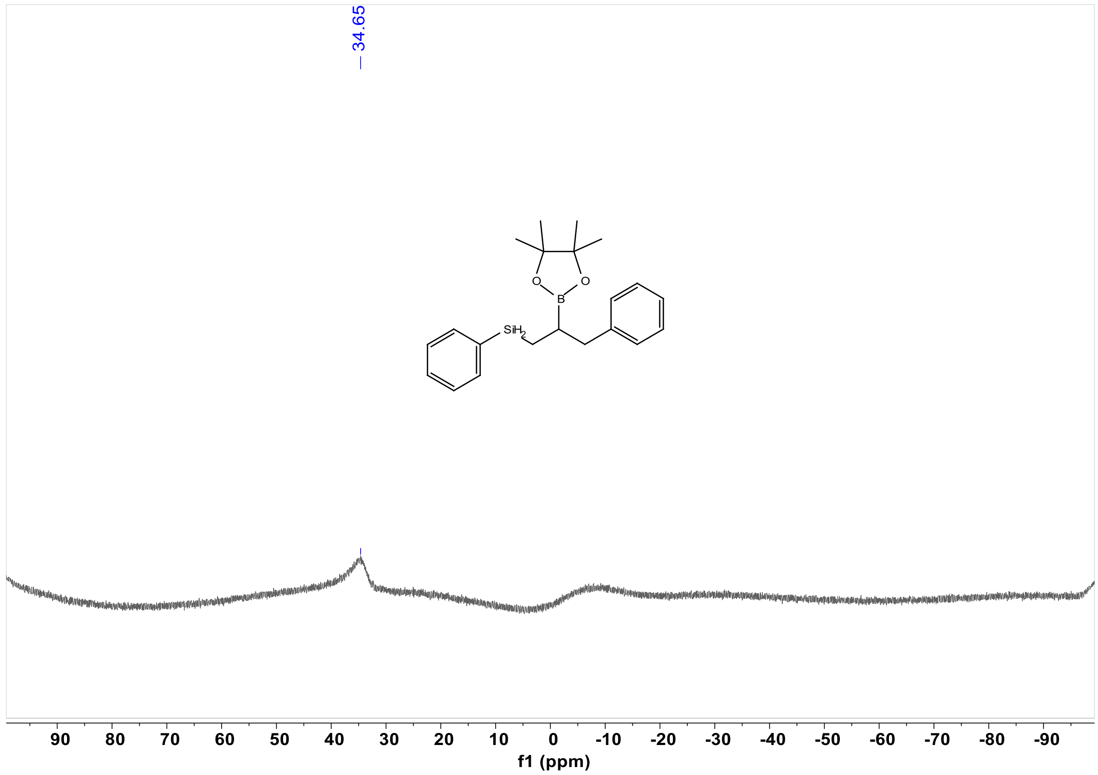

Supplementary Figure 225. ^{11}B NMR spectra for **70**

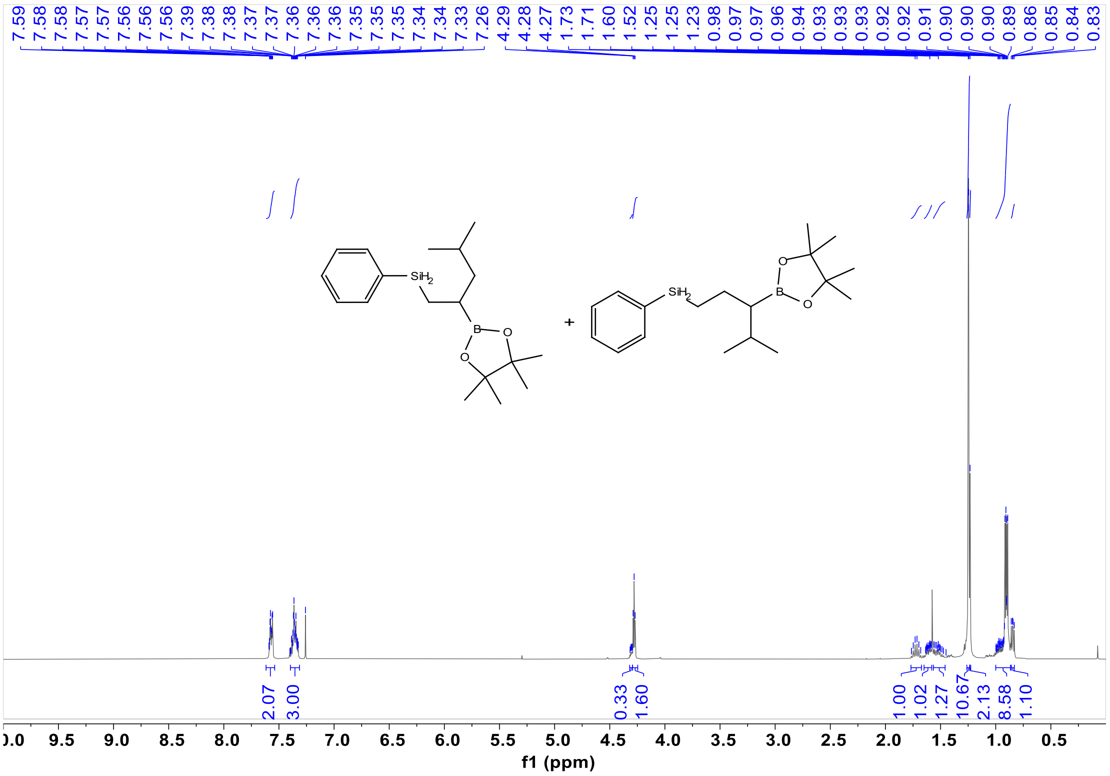

Supplementary Figure 226. ^{1}H NMR spectra for **71** and **71'**

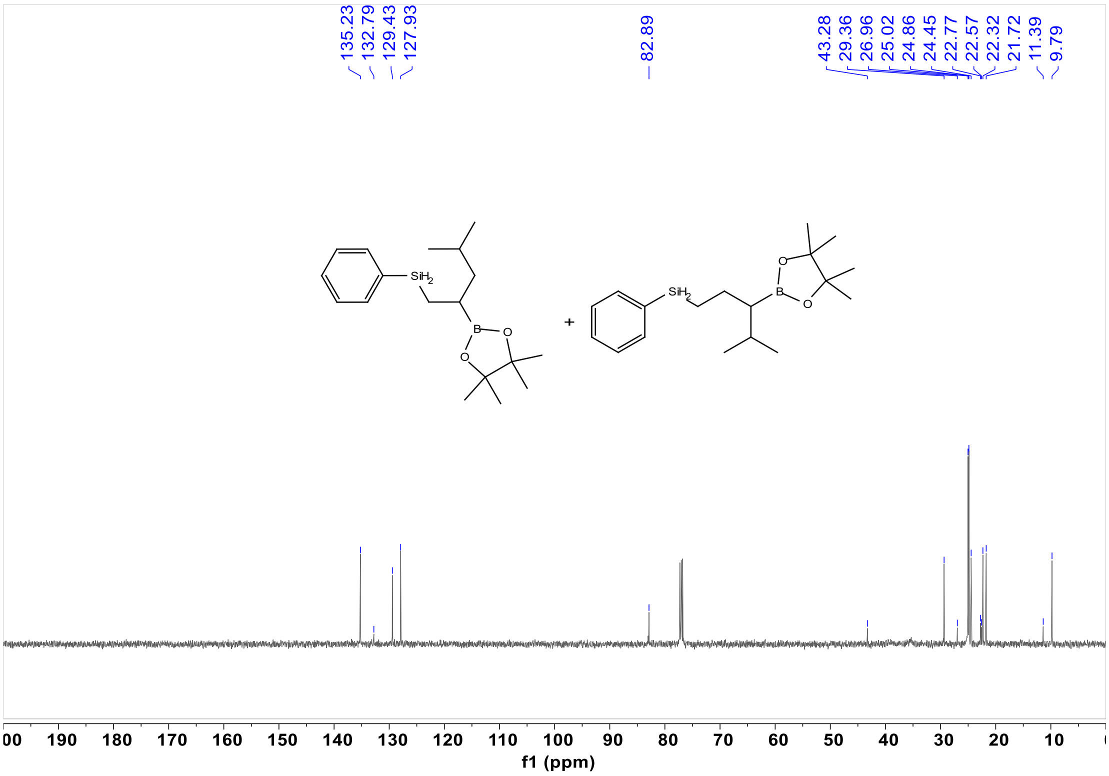

Supplementary Figure 227. ^{13}C NMR spectra for **71** and **71'**

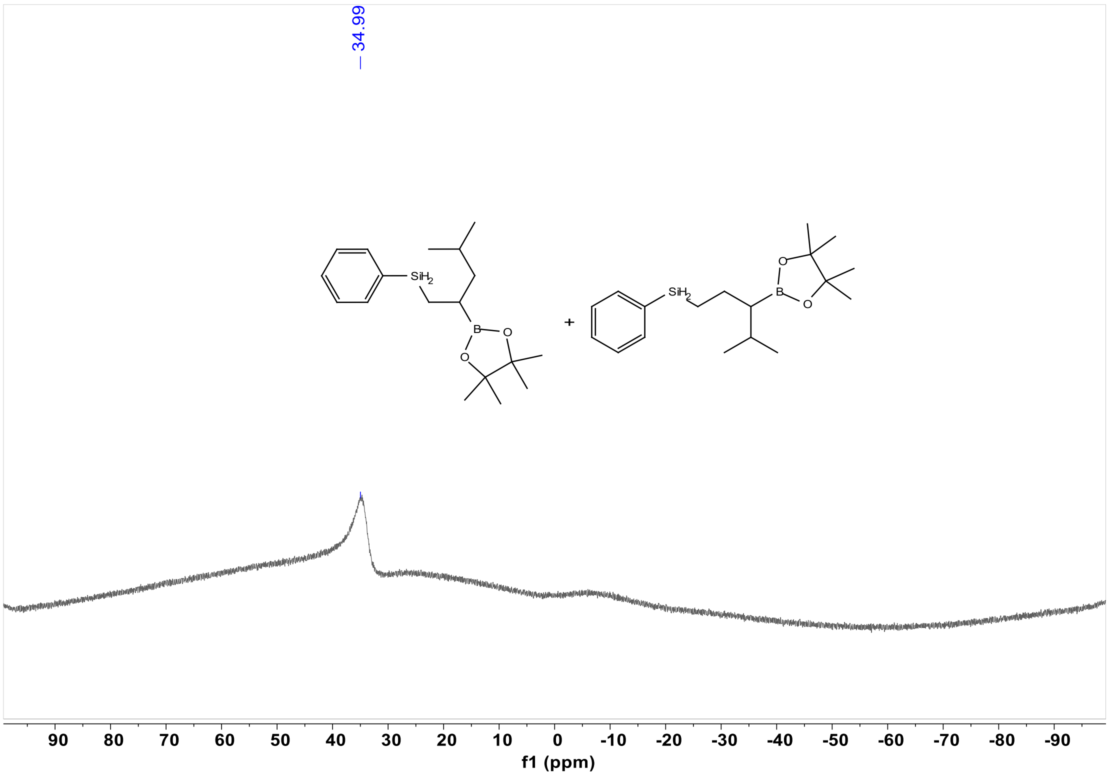

Supplementary Figure 228. ^{11}B NMR spectra for **71** and **71'**

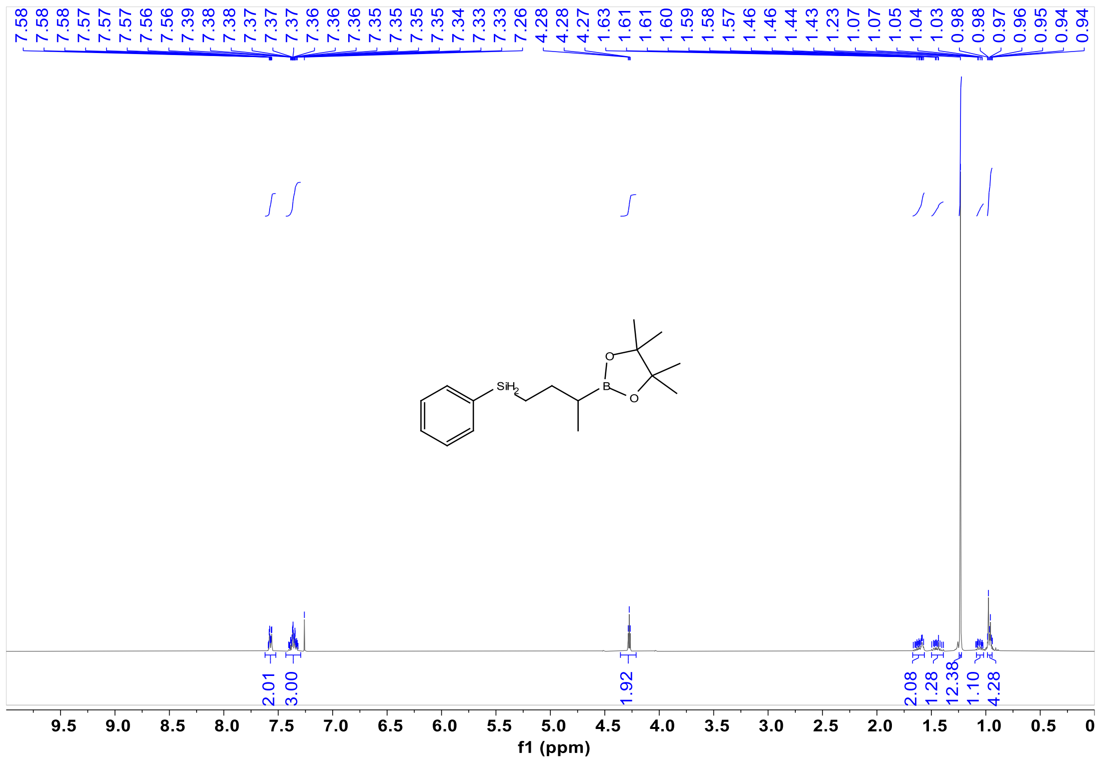

Supplementary Figure 229. ^{1}H NMR spectra for **72**

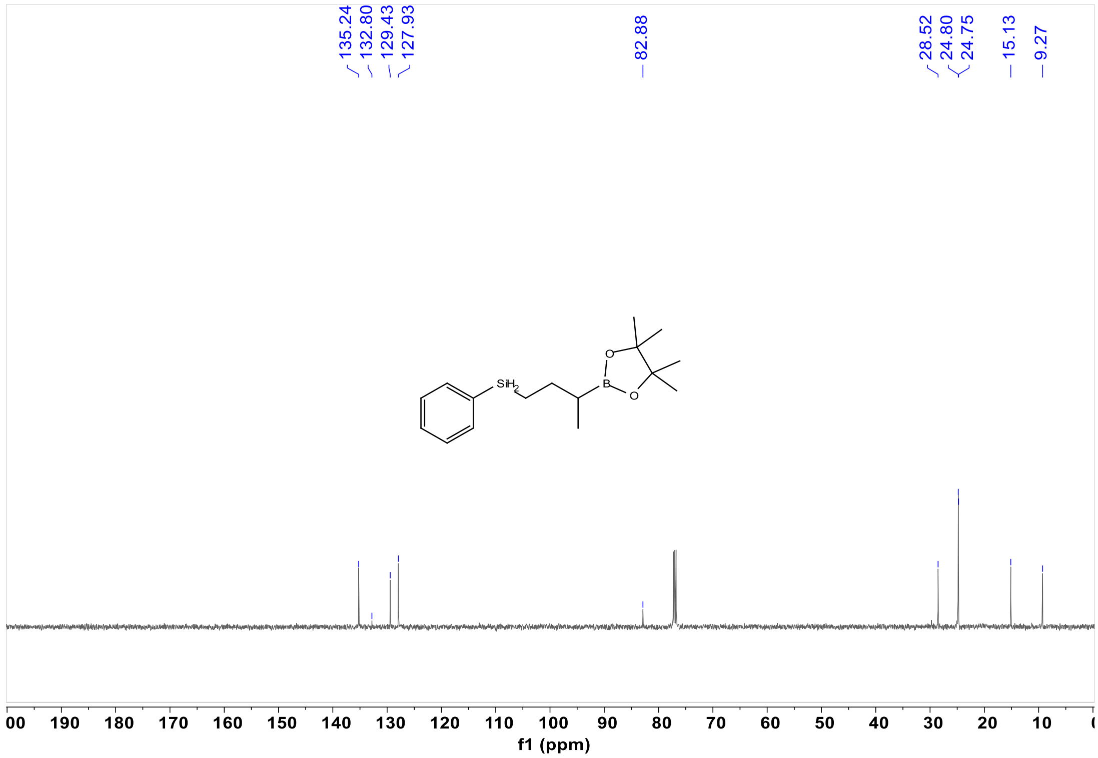

Supplementary Figure 230. ^{13}C NMR spectra for **72**

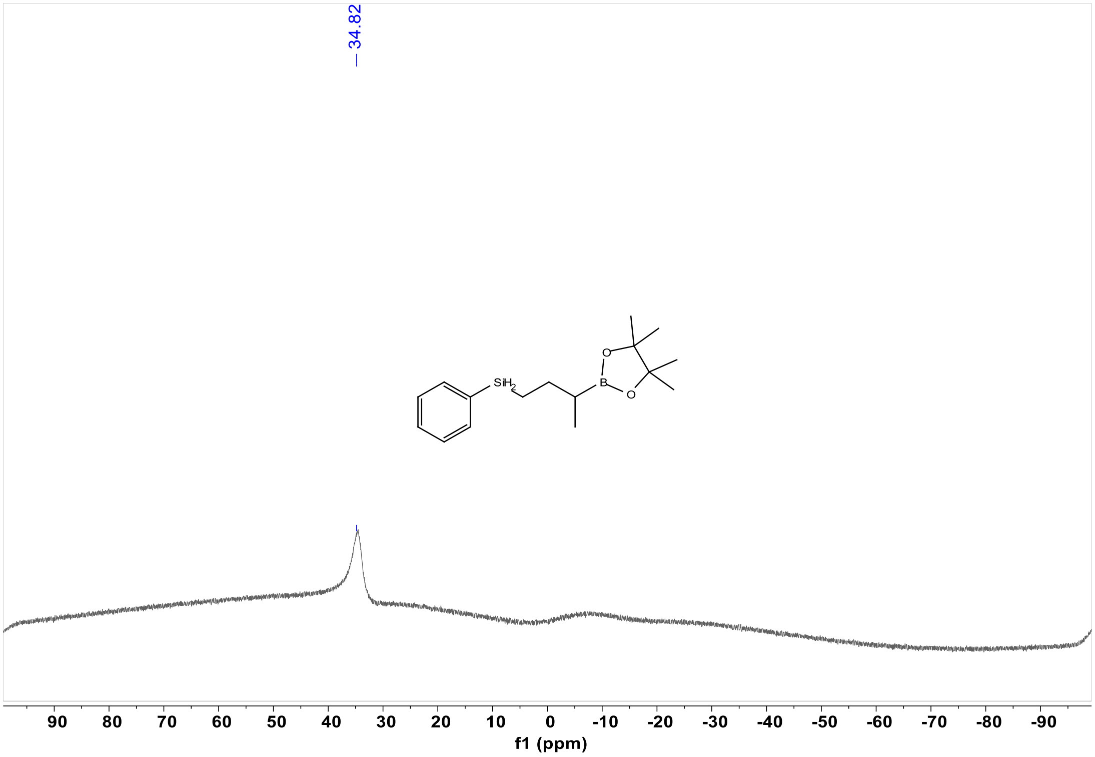

Supplementary Figure 231. ^{11}B NMR spectra for **72**

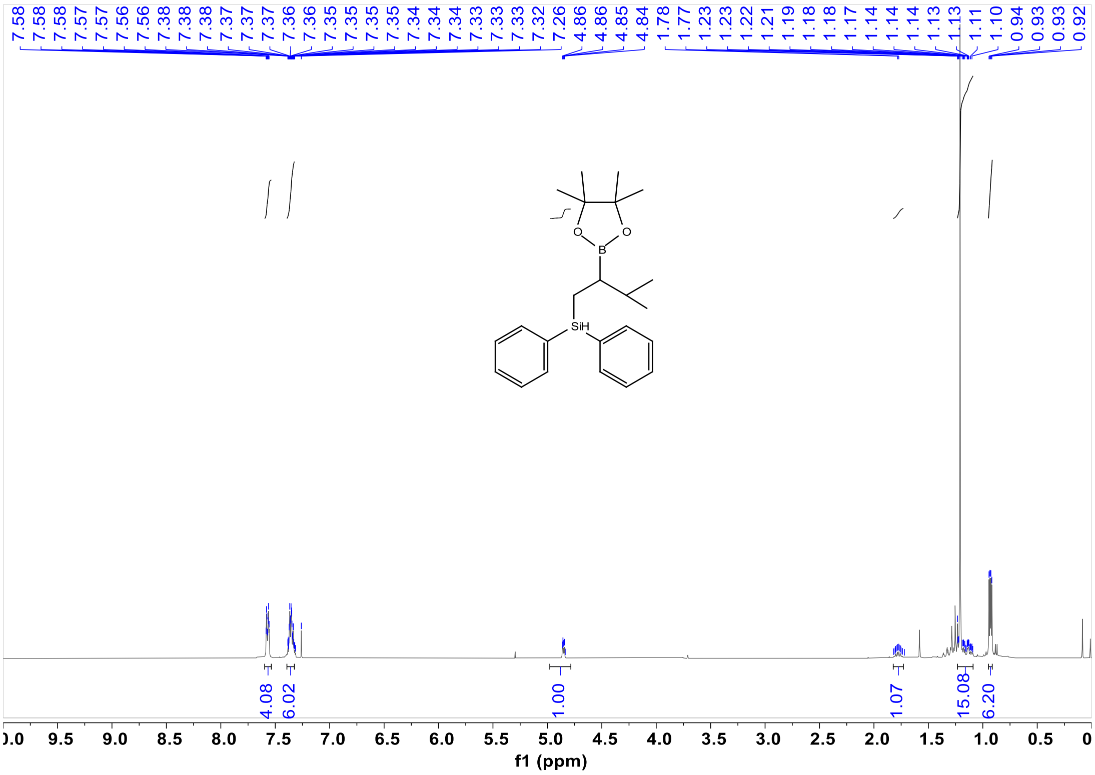

Supplementary Figure 232. ^{1}H NMR spectra for **73**

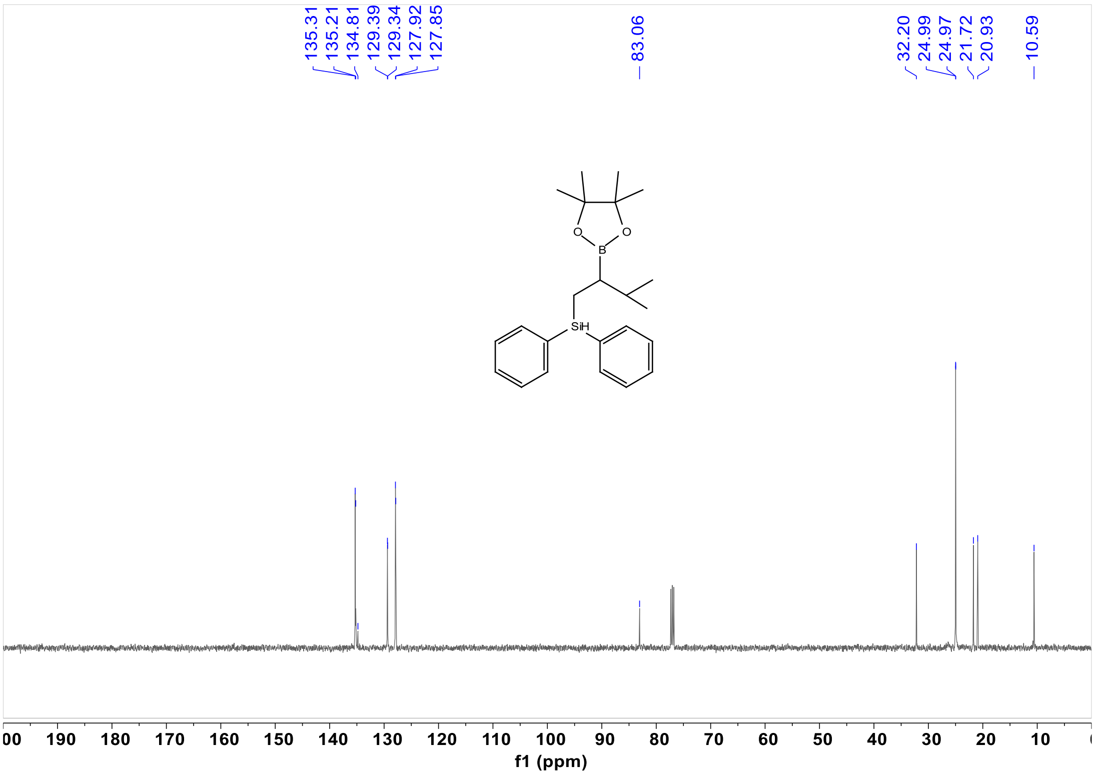

Supplementary Figure 233. ^{13}C NMR spectra for **73**

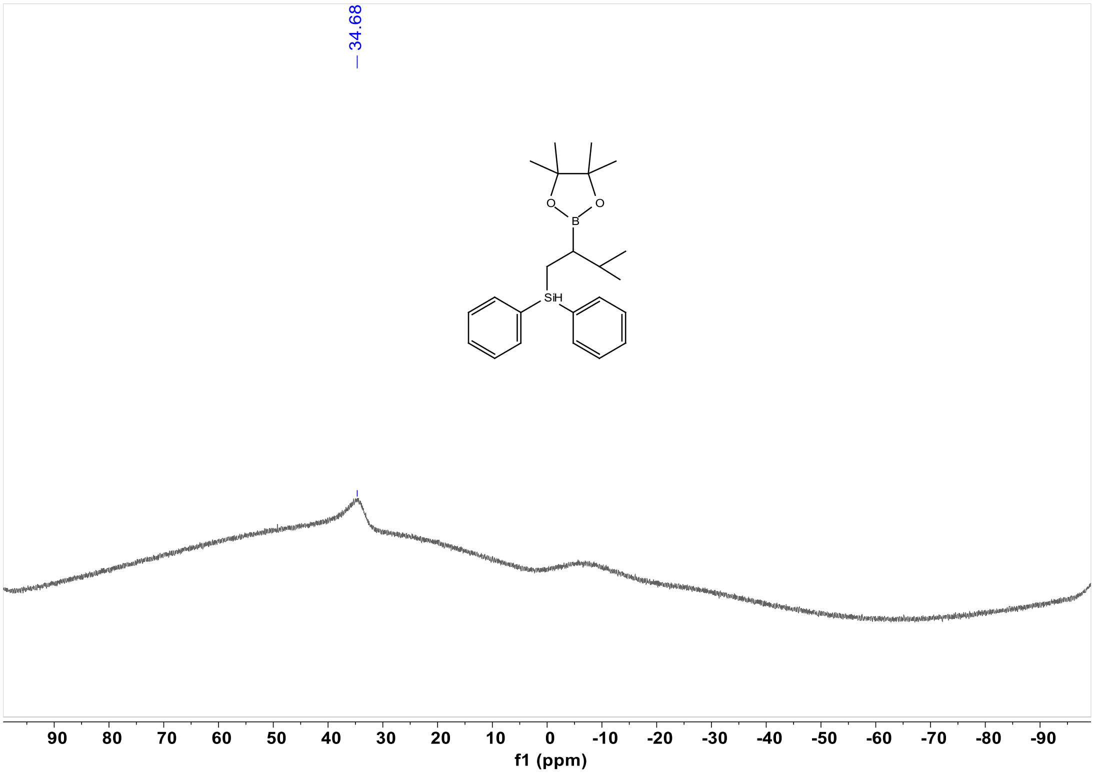

Supplementary Figure 234. ^{11}B NMR spectra for **73**

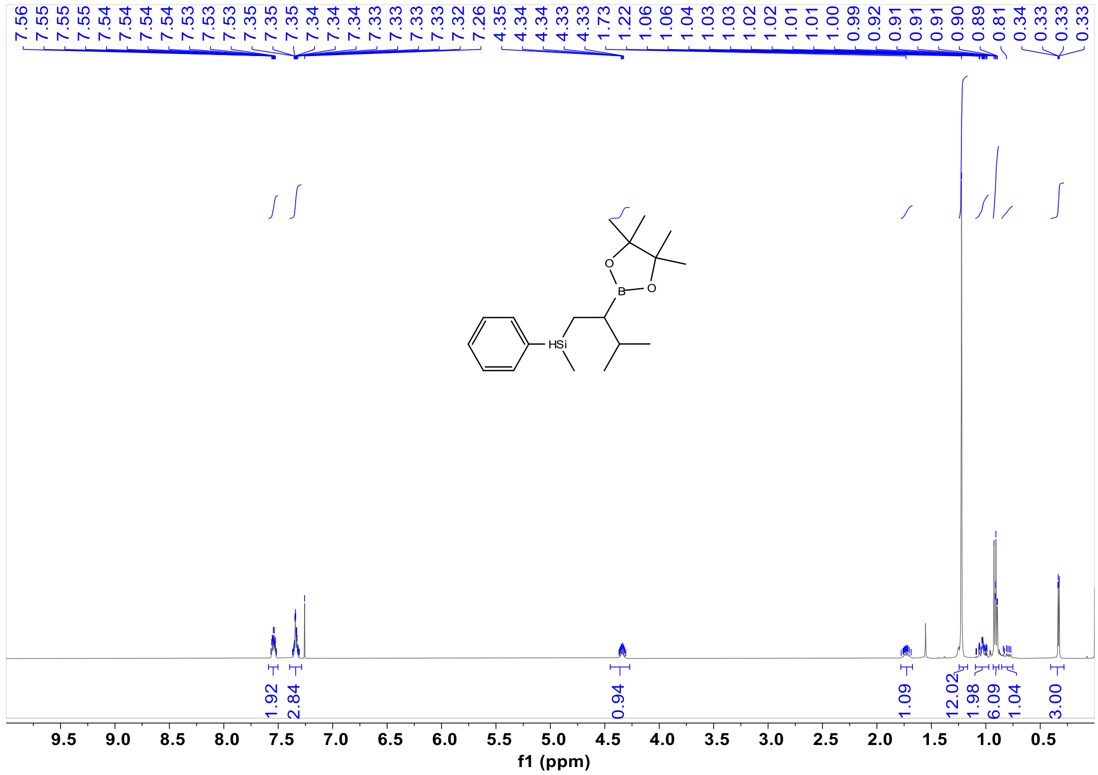

Supplementary Figure 235. ^{1}H NMR spectra for **74**

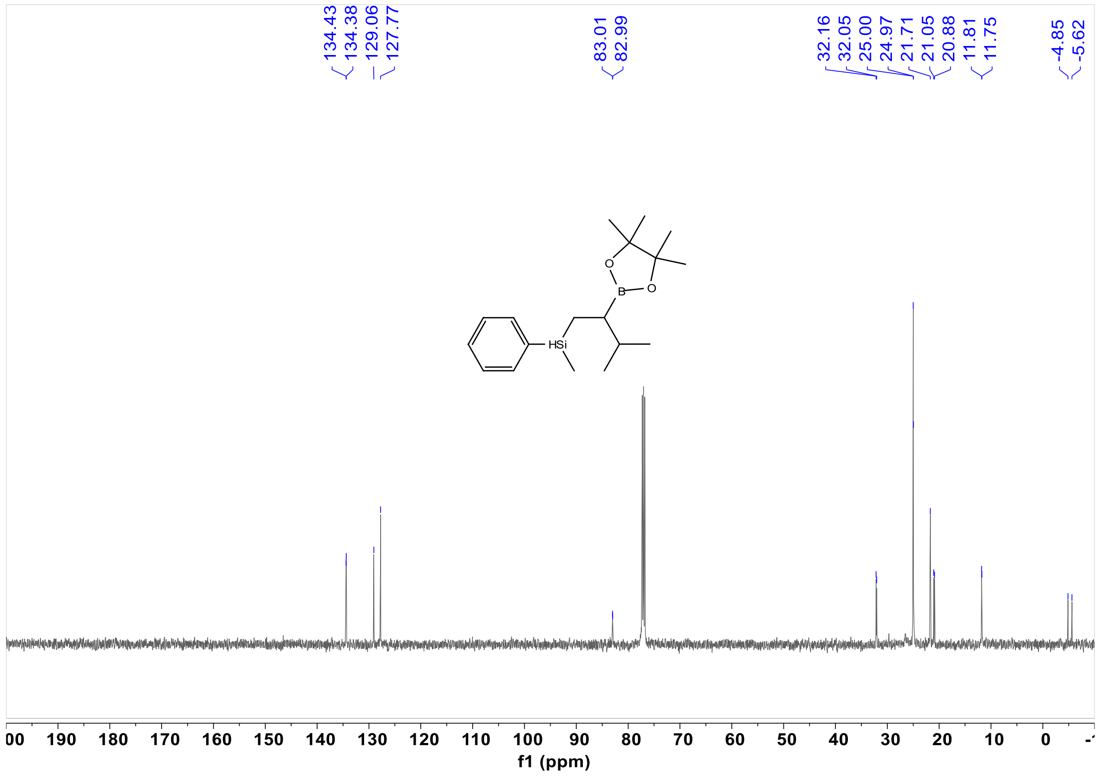

Supplementary Figure 236. ^{13}C NMR spectra for **74**

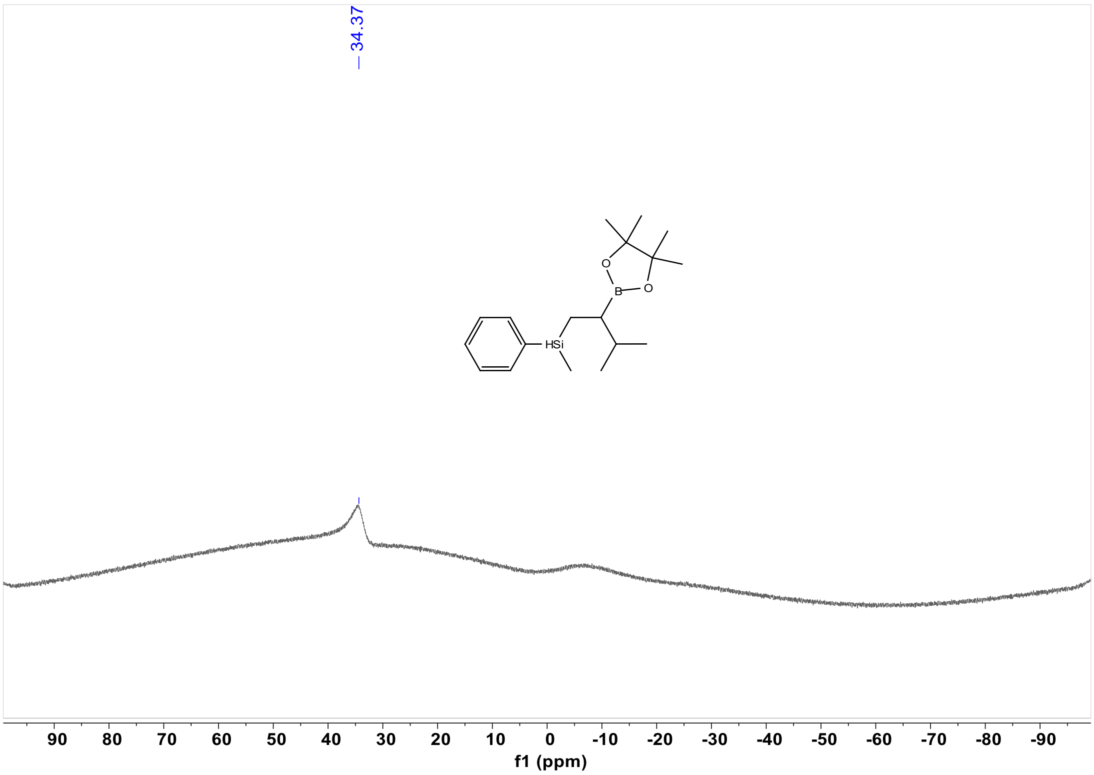

Supplementary Figure 237. ^{11}B NMR spectra for **74**

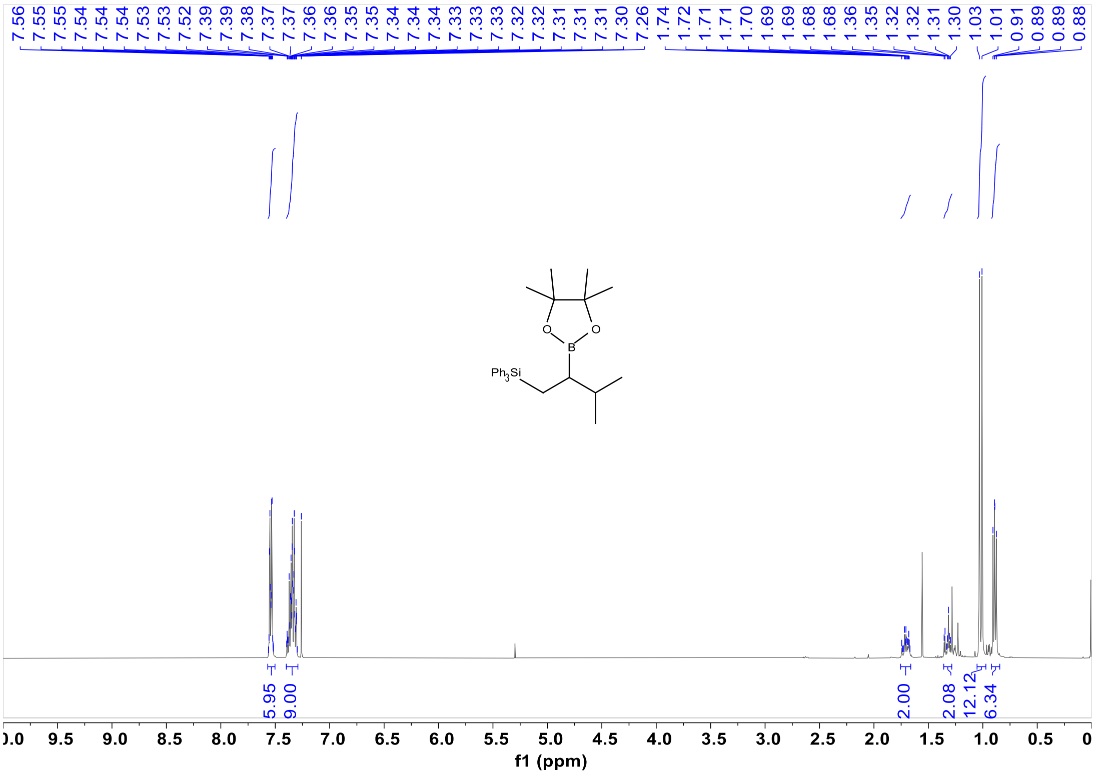

Supplementary Figure 238. ^{1}H NMR spectra for **75**

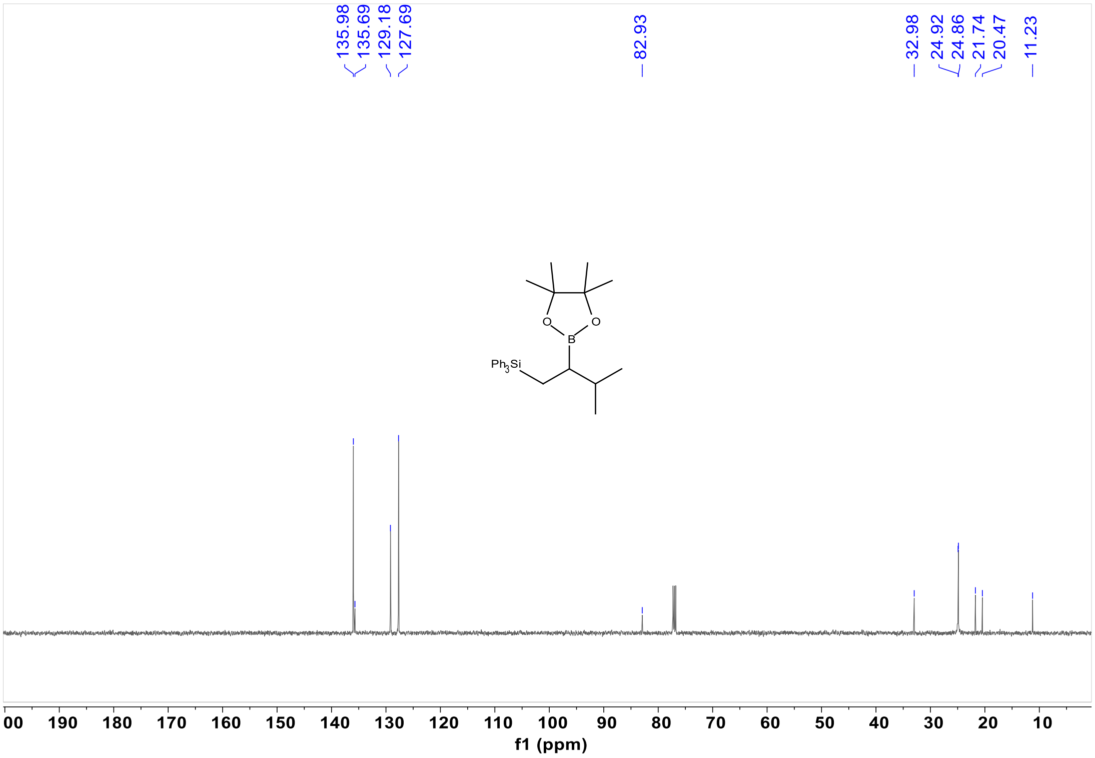

Supplementary Figure 239. ^{13}C NMR spectra for **75**

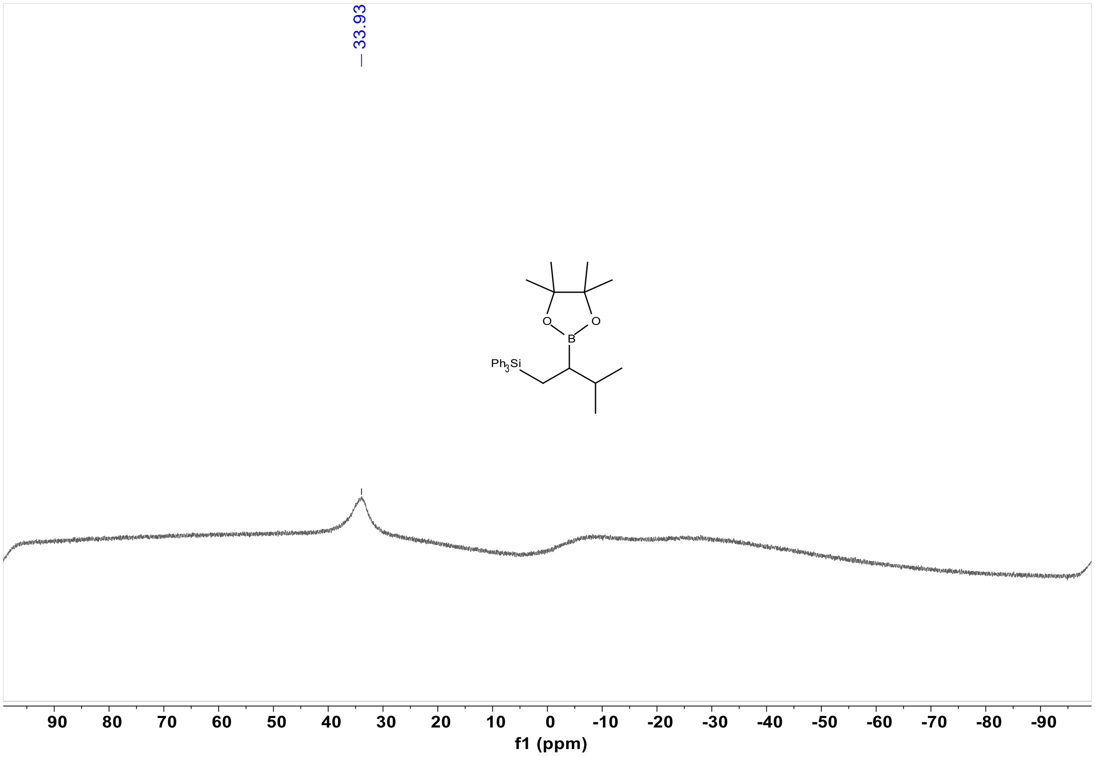

Supplementary Figure 240. ^{11}B NMR spectra for **75**

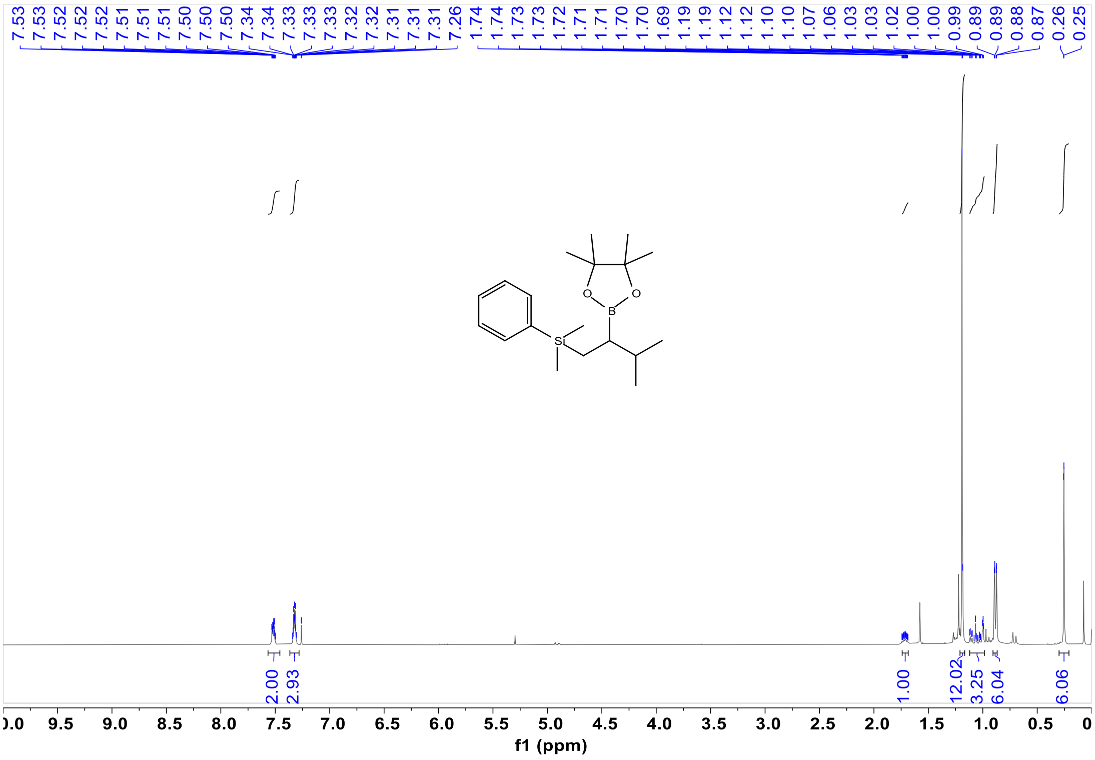

Supplementary Figure 241. ^{1}H NMR spectra for **76**

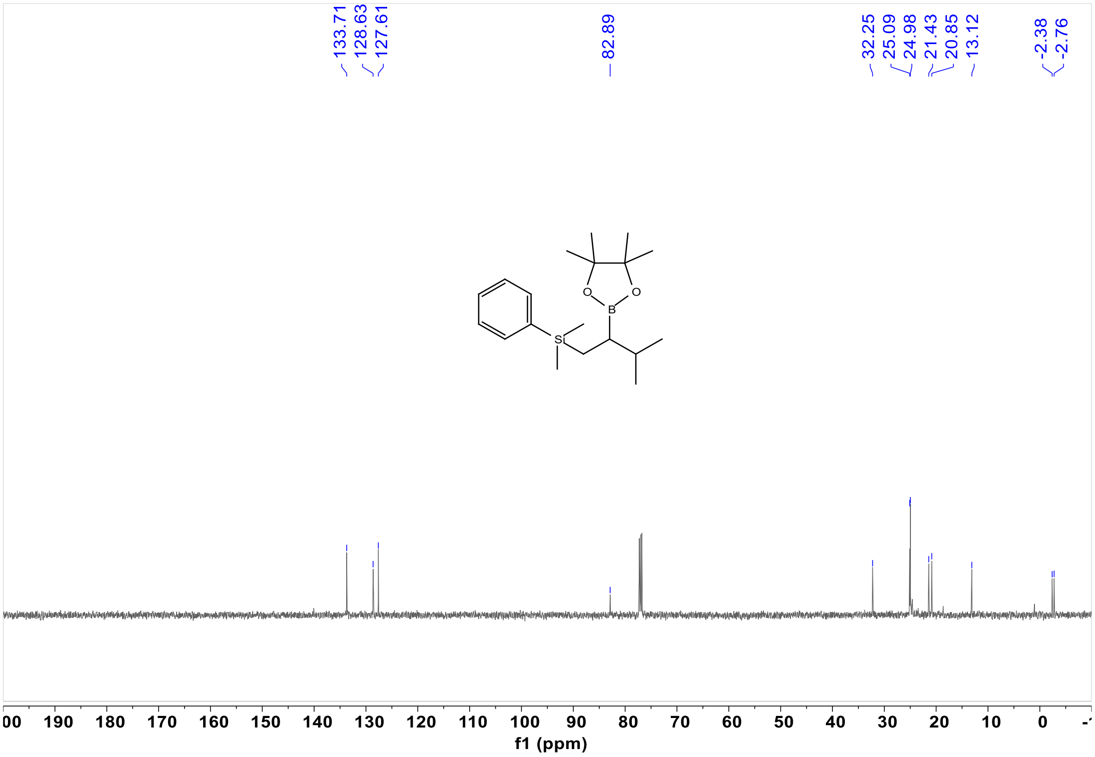

Supplementary Figure 242. ^{13}C NMR spectra for **76**

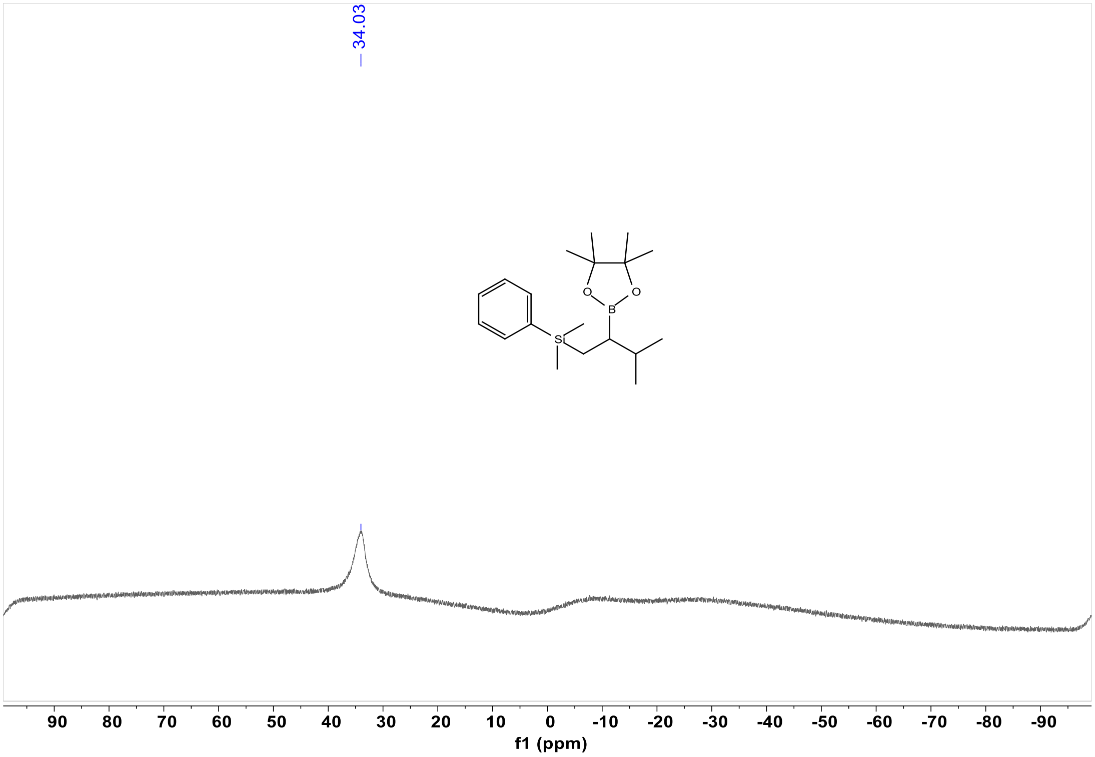

Supplementary Figure 243. ^{11}B NMR spectra for **76**

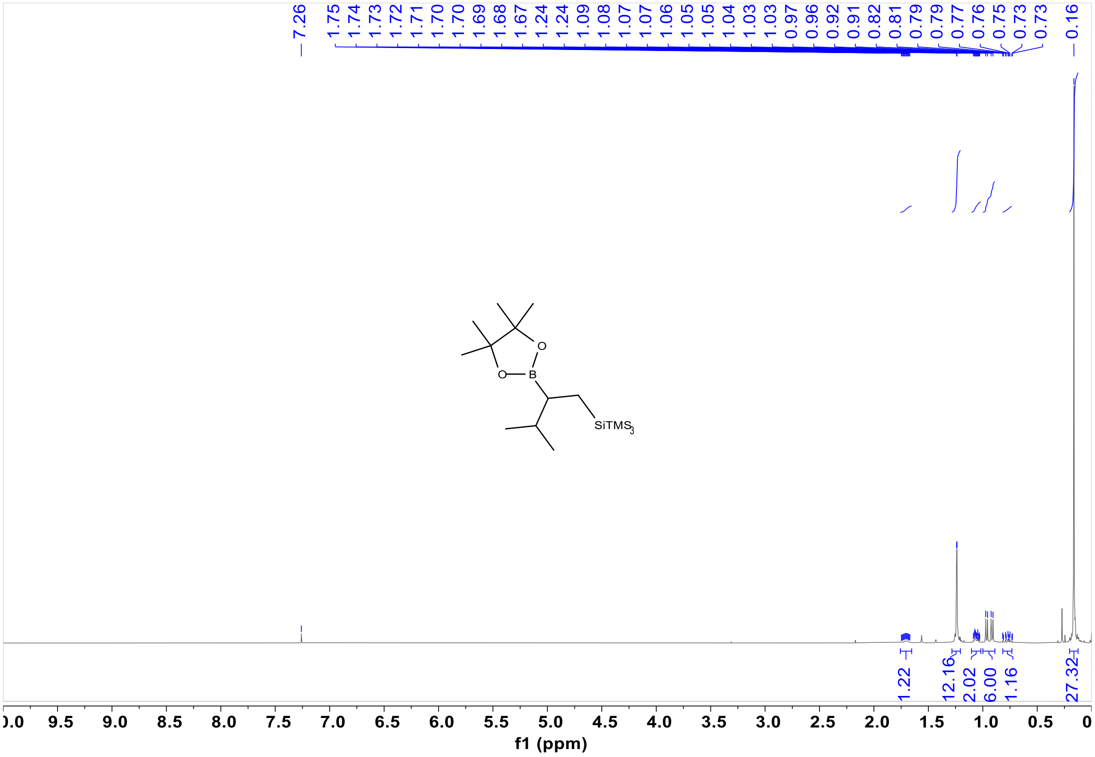

Supplementary Figure 244. ^{1}H NMR spectra for **77**

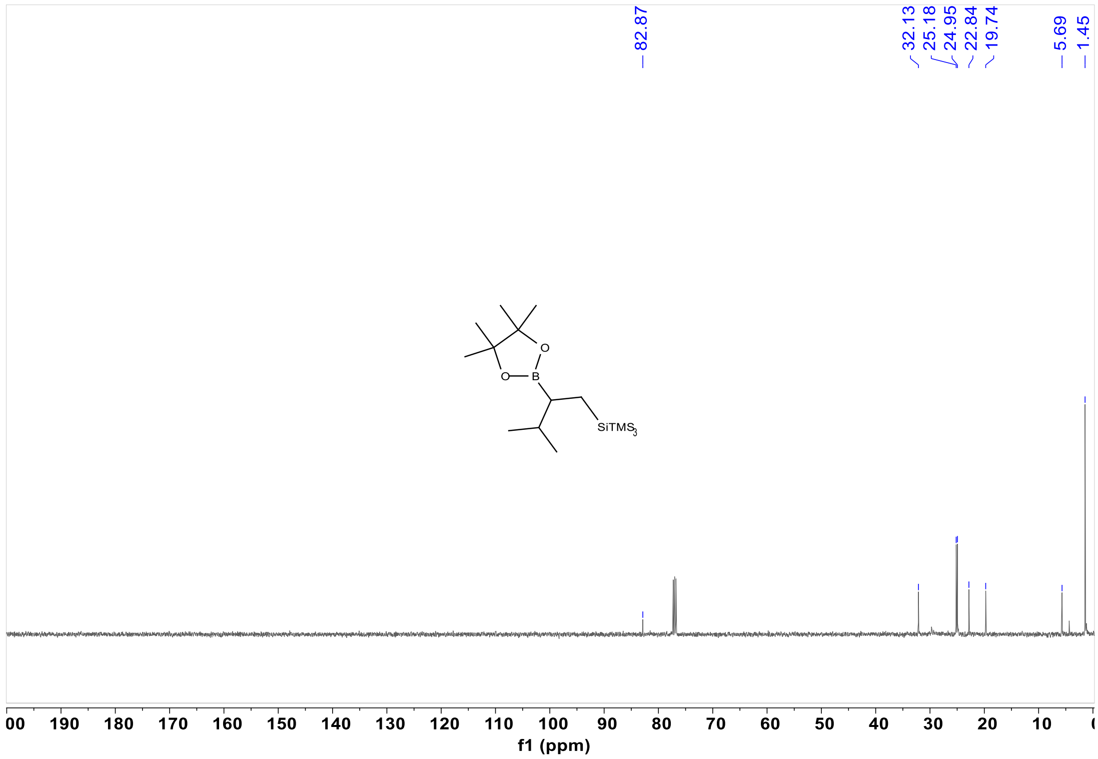

Supplementary Figure 245. ^{13}C NMR spectra for **77**

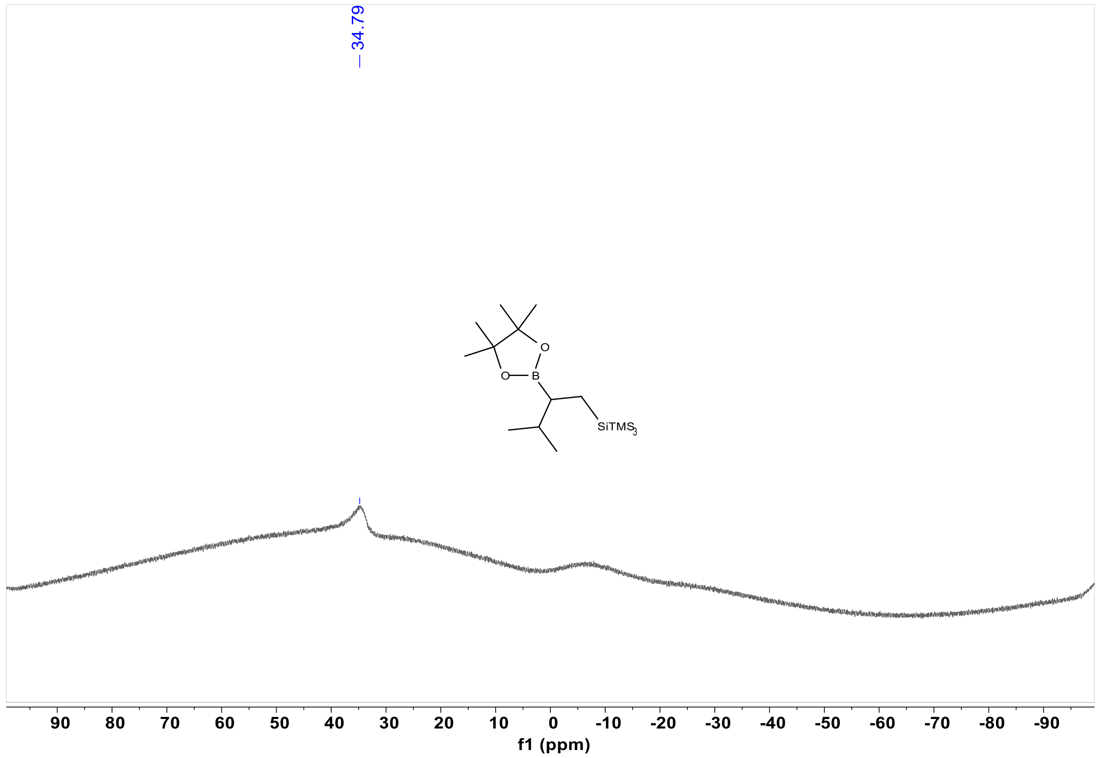

Supplementary Figure 246. ^{11}B NMR spectra for **77**

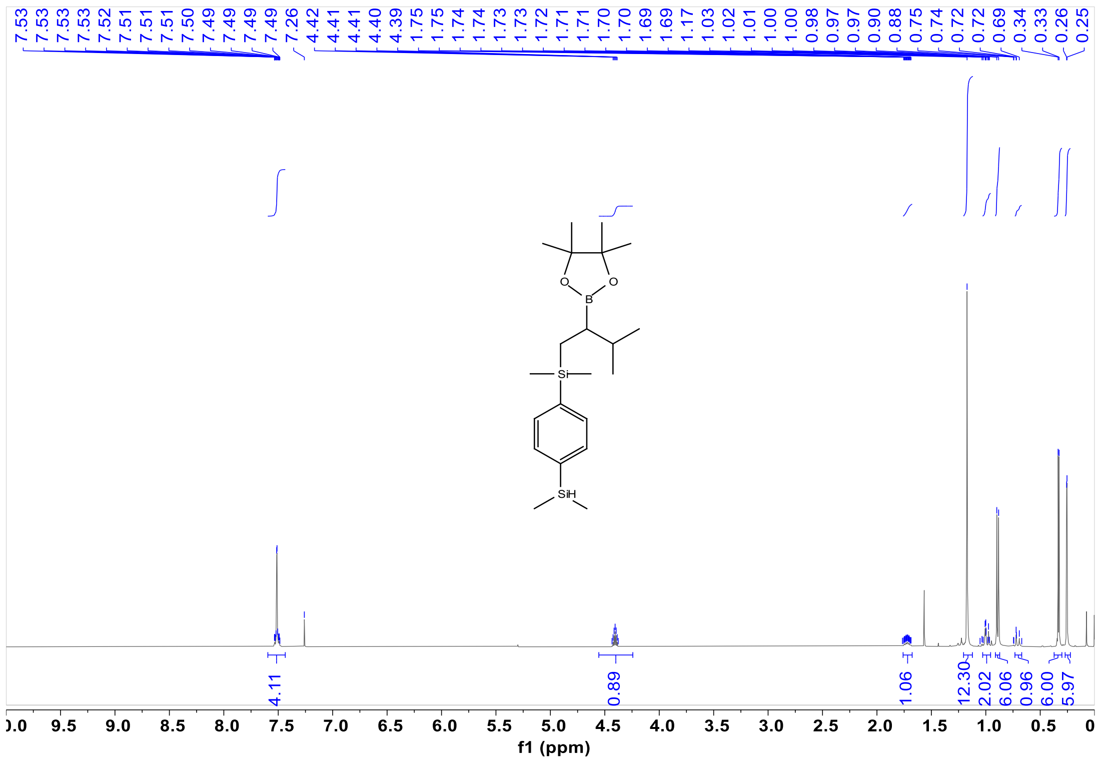

Supplementary Figure 247. ^{1}H NMR spectra for **78**

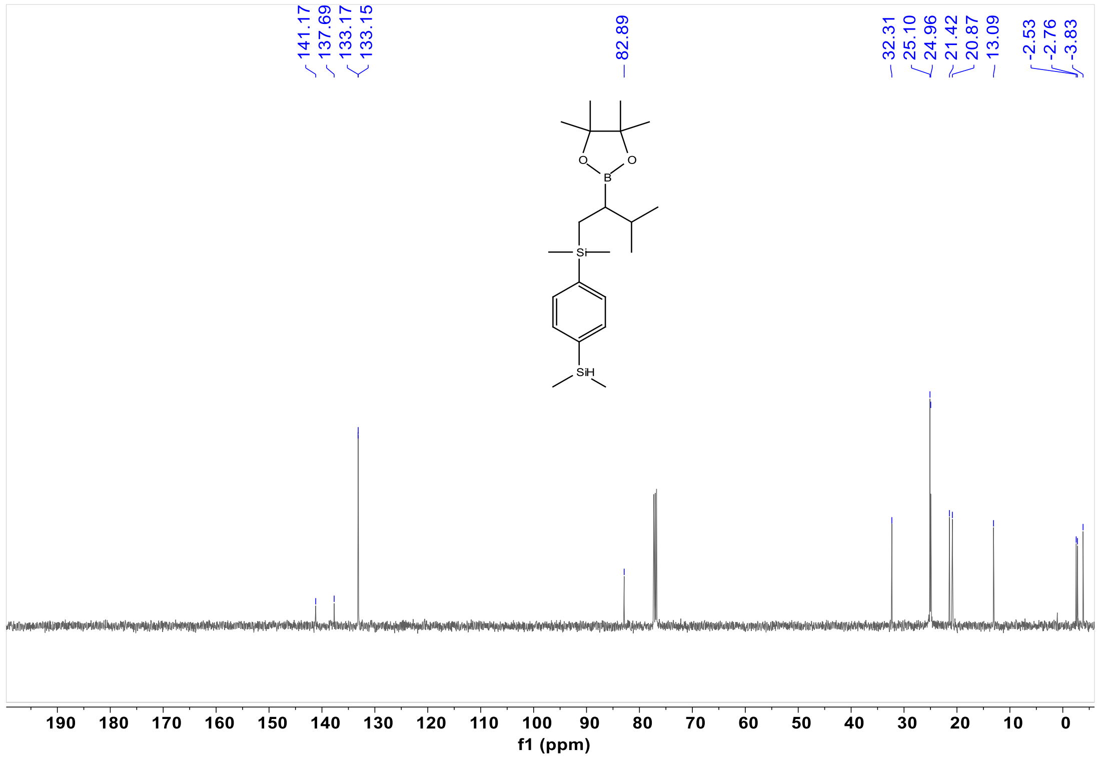

Supplementary Figure 248. ^{13}C NMR spectra for **78**

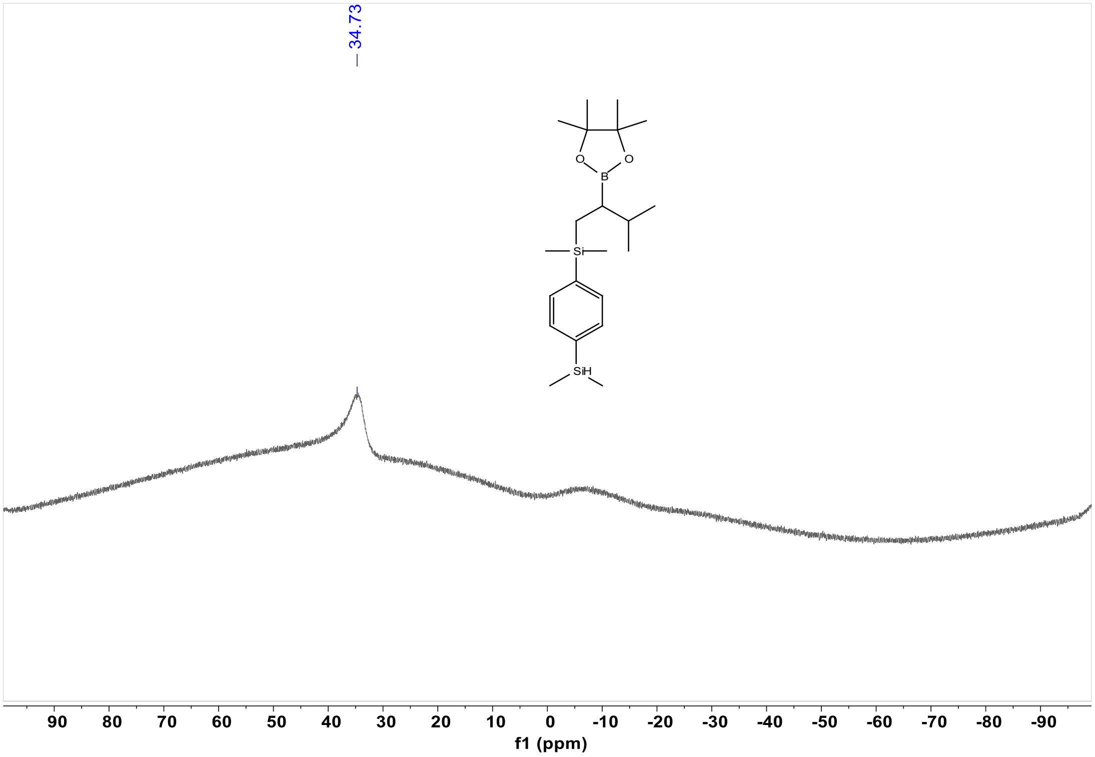

Supplementary Figure 249. ^{11}B NMR spectra for **78**

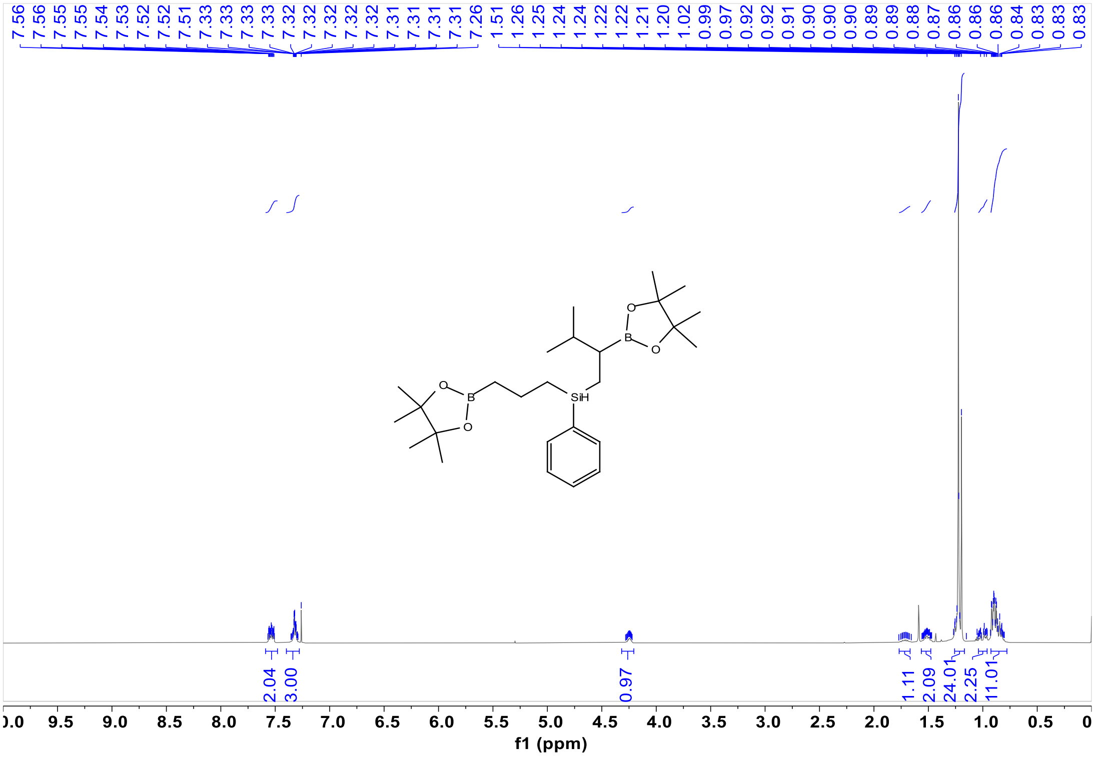

Supplementary Figure 250. ^{1}H NMR spectra for **79**

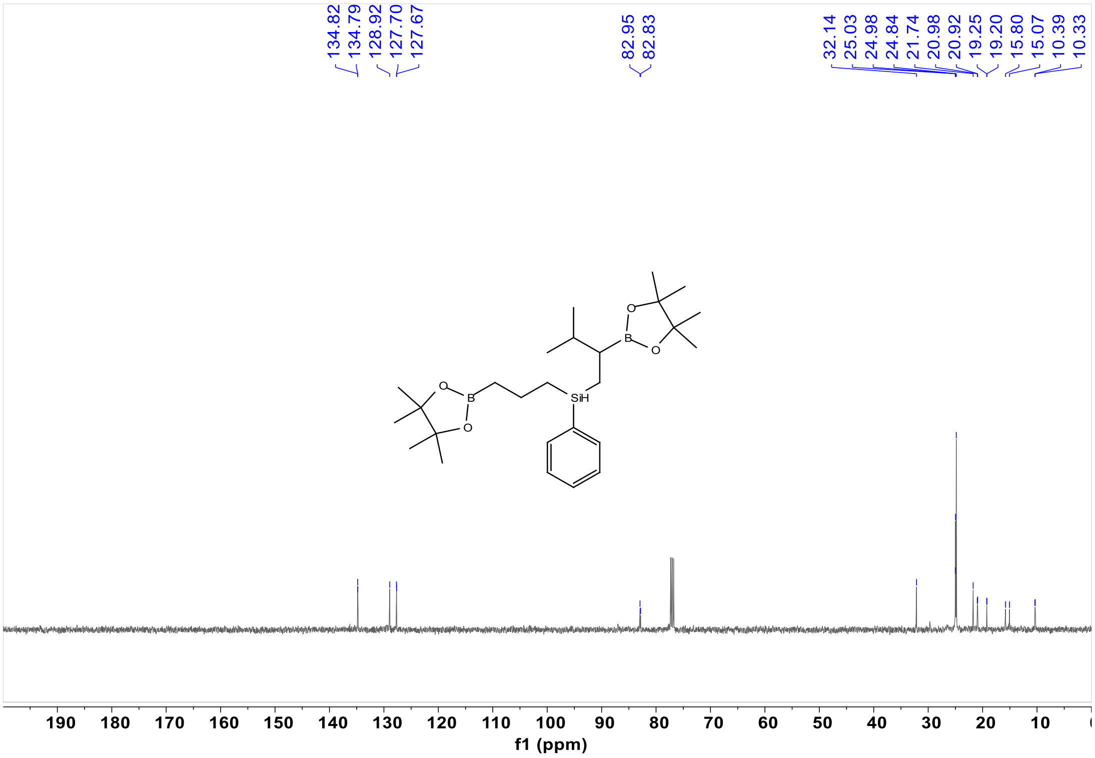

Supplementary Figure 251. ^{13}C NMR spectra for **79**

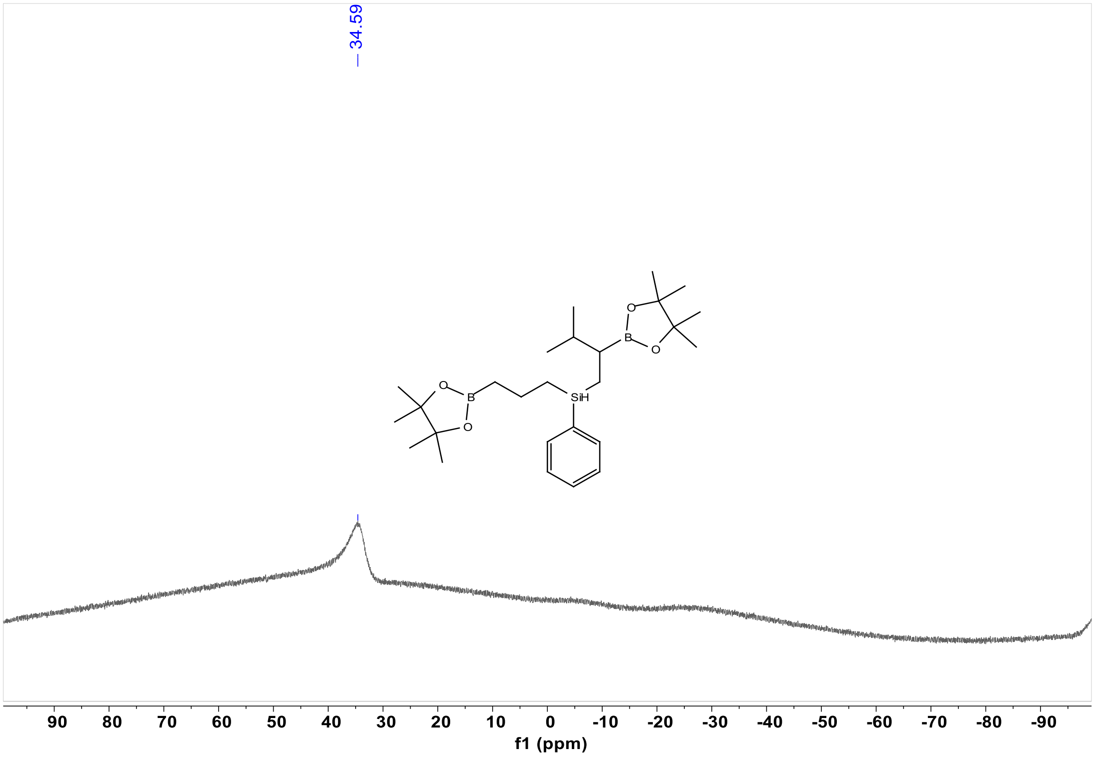

Supplementary Figure 252. ^{11}B NMR spectra for **79**

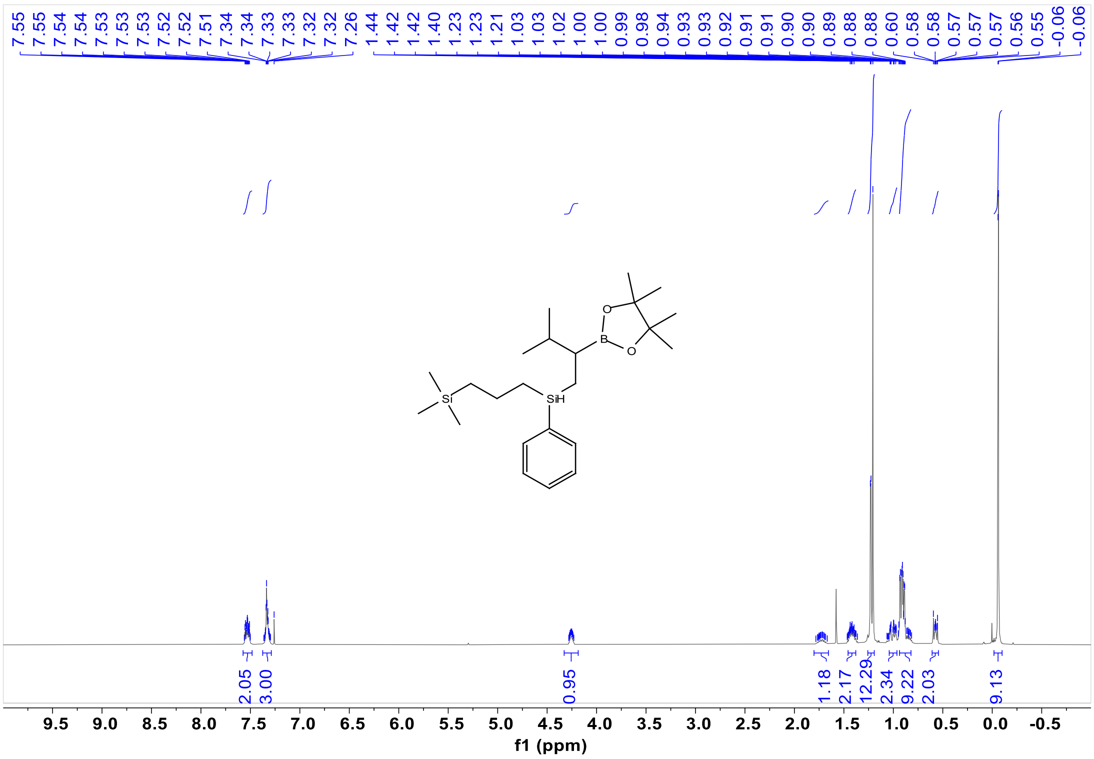

Supplementary Figure 253. ^{1}H NMR spectra for **80**

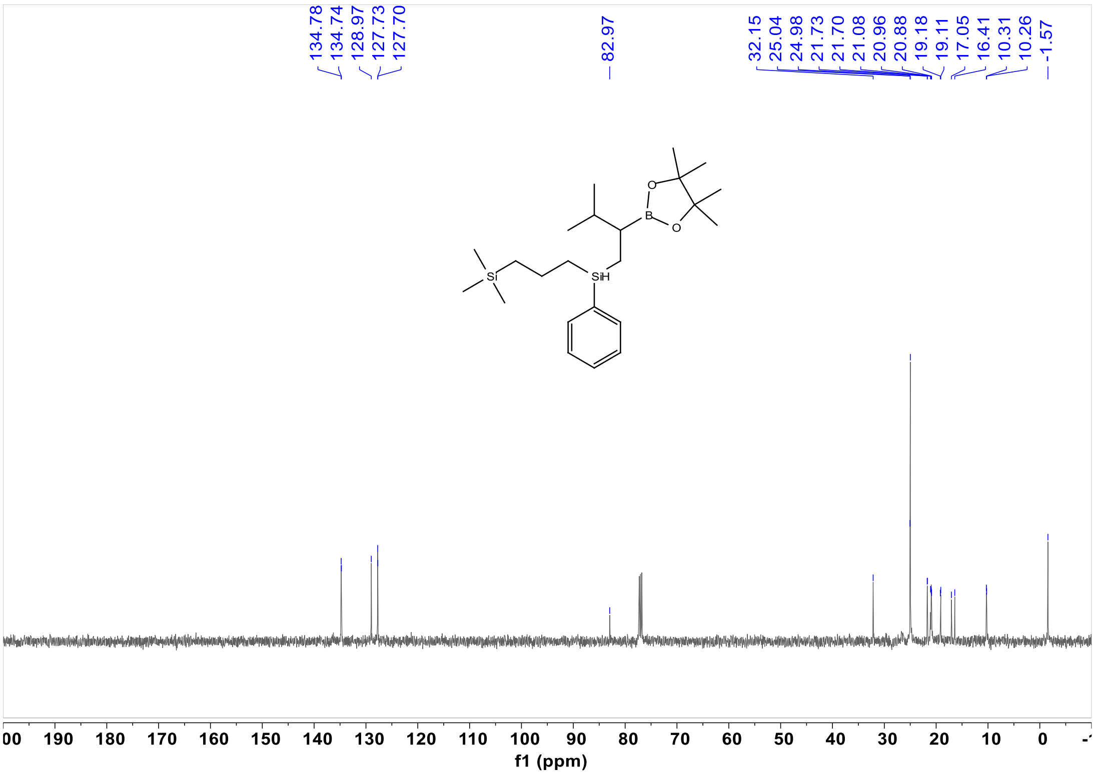

Supplementary Figure 254. ^{13}C NMR spectra for **80**

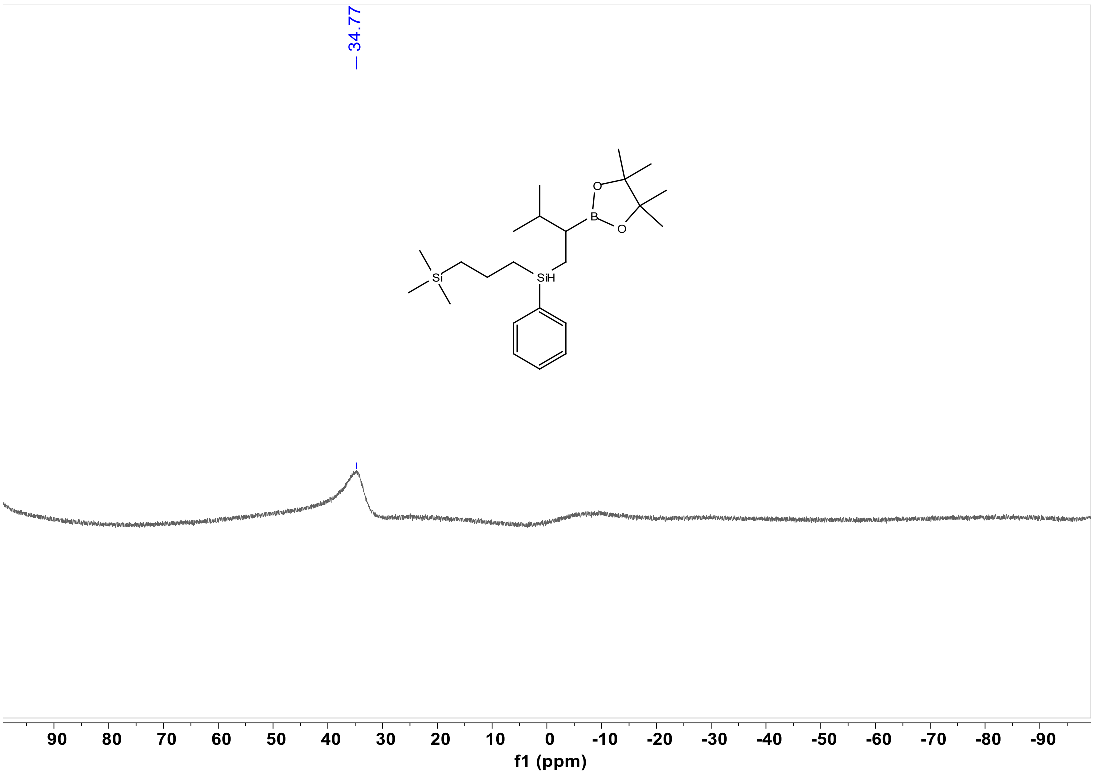

Supplementary Figure 255. ^{11}B NMR spectra for **80**

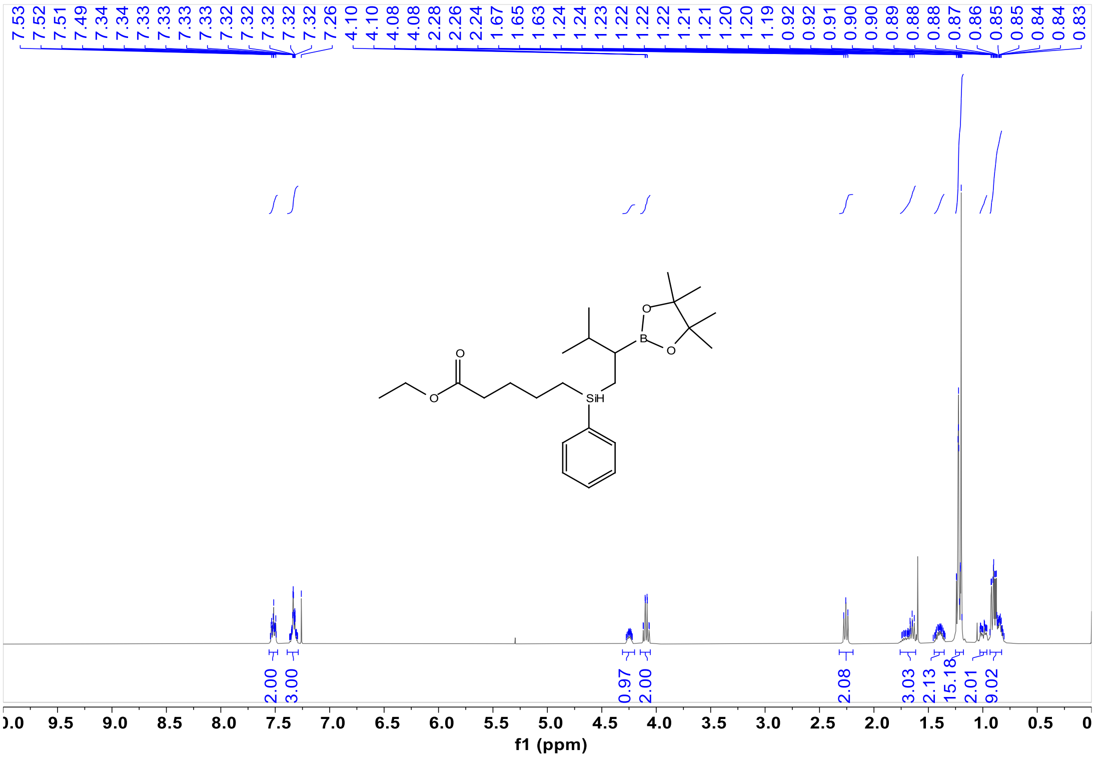

Supplementary Figure 256. ^{1}H NMR spectra for **81**

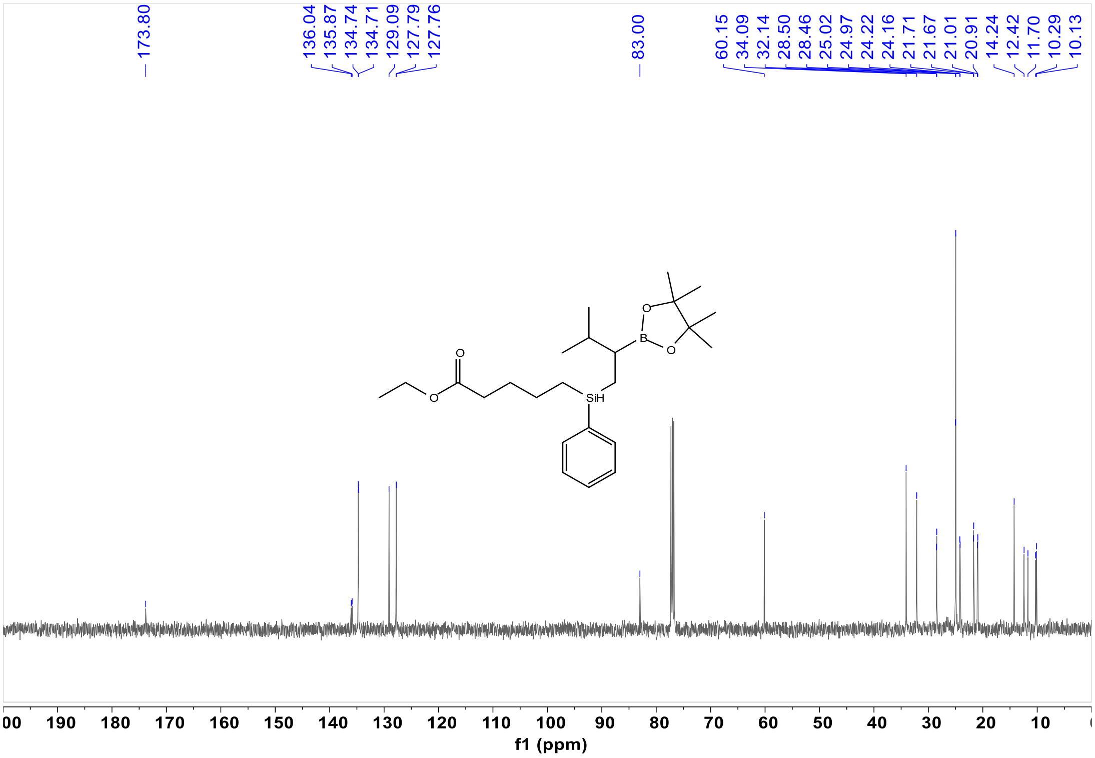

Supplementary Figure 257. ^{13}C NMR spectra for **81**

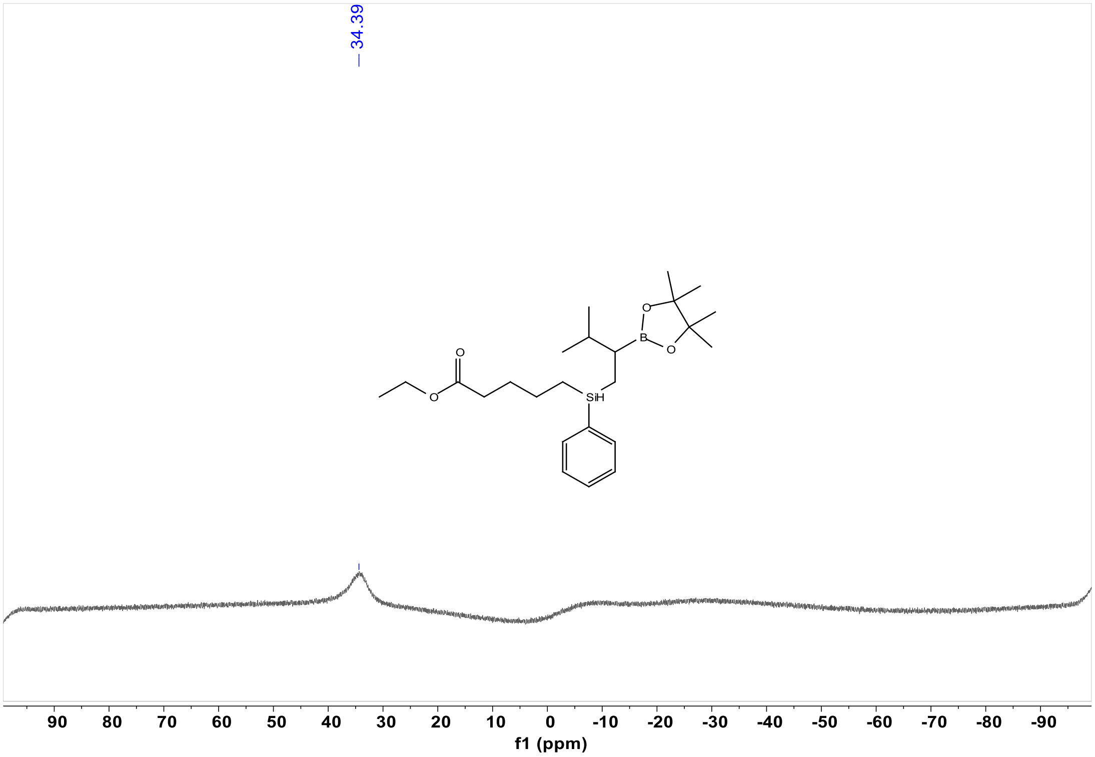

Supplementary Figure 258. ^{11}B NMR spectra for **81**

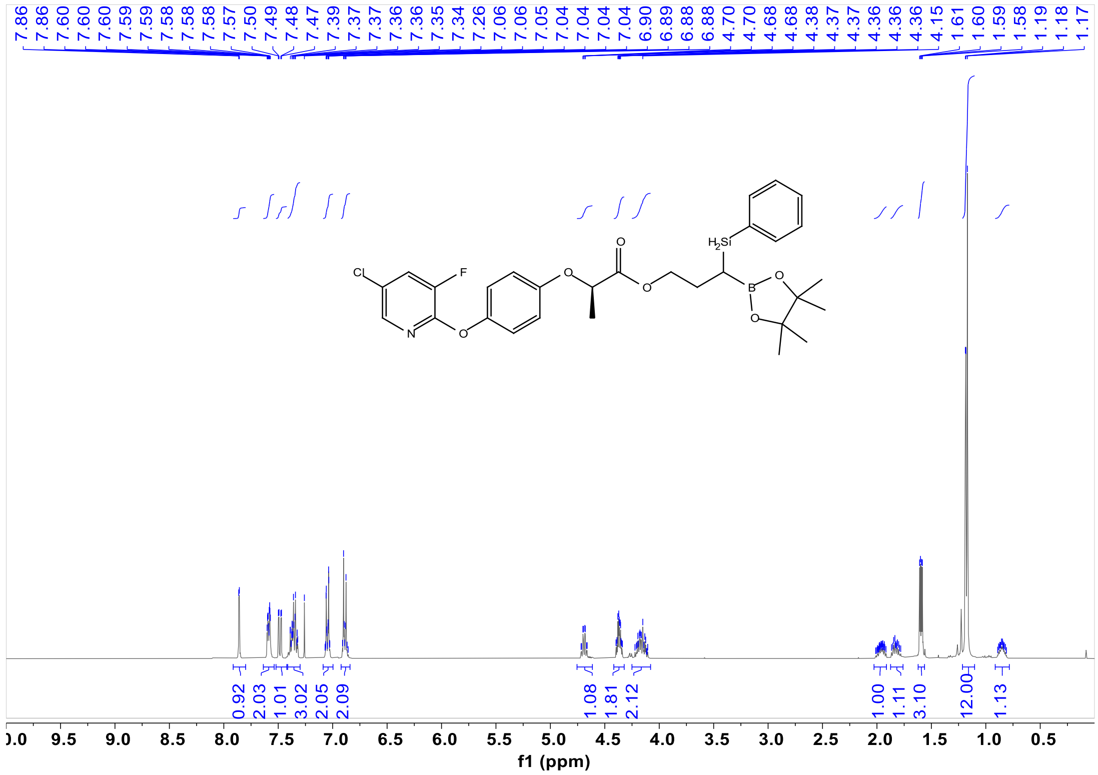

Supplementary Figure 259. ^{1}H NMR spectra for **82**

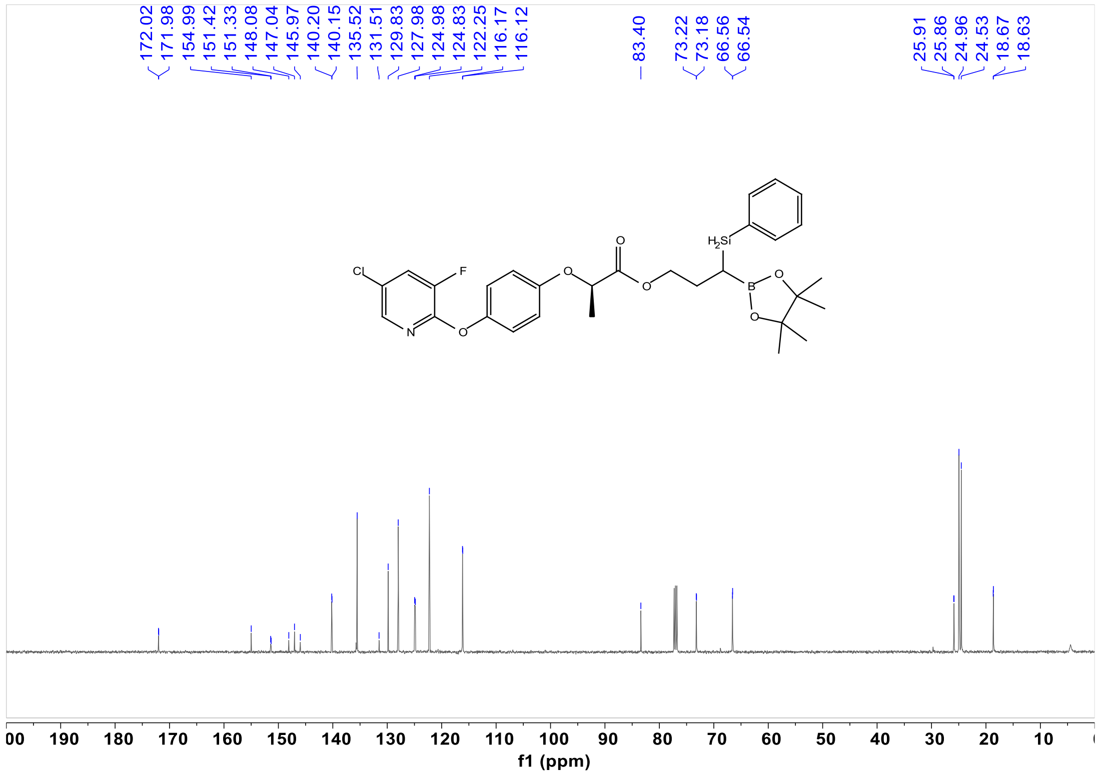

Supplementary Figure 260. ^{13}C NMR spectra for **82**

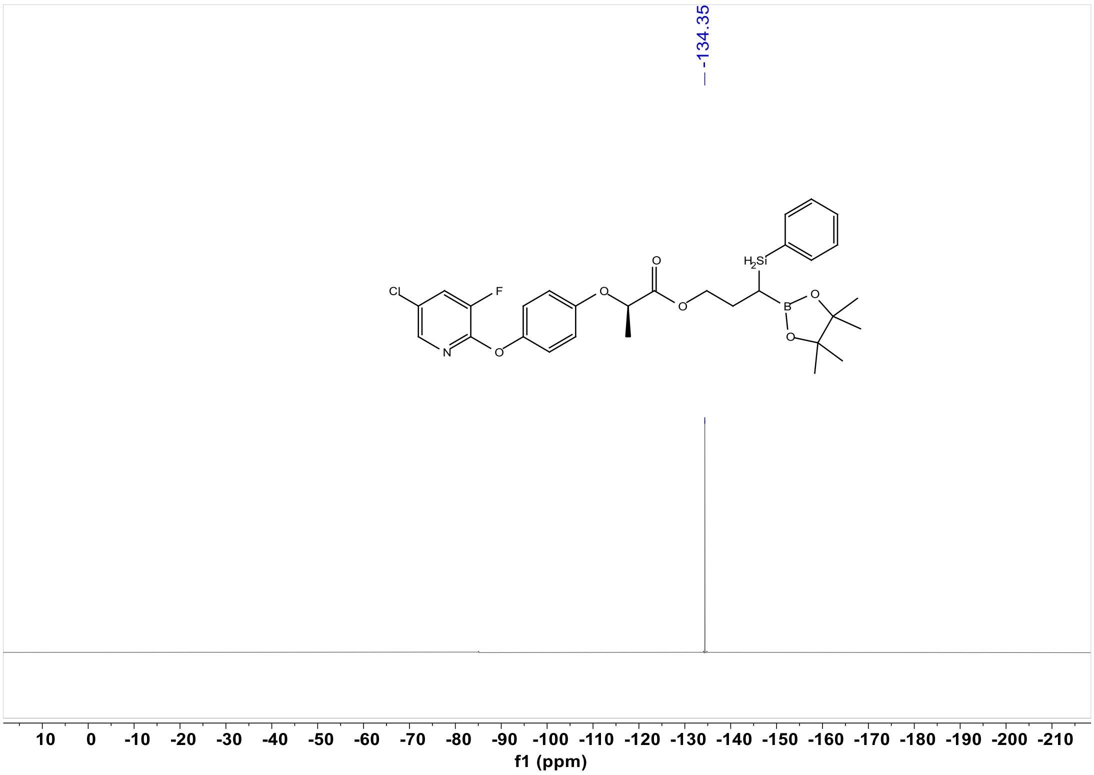

Supplementary Figure 261. ^{19}F NMR spectra for **82**

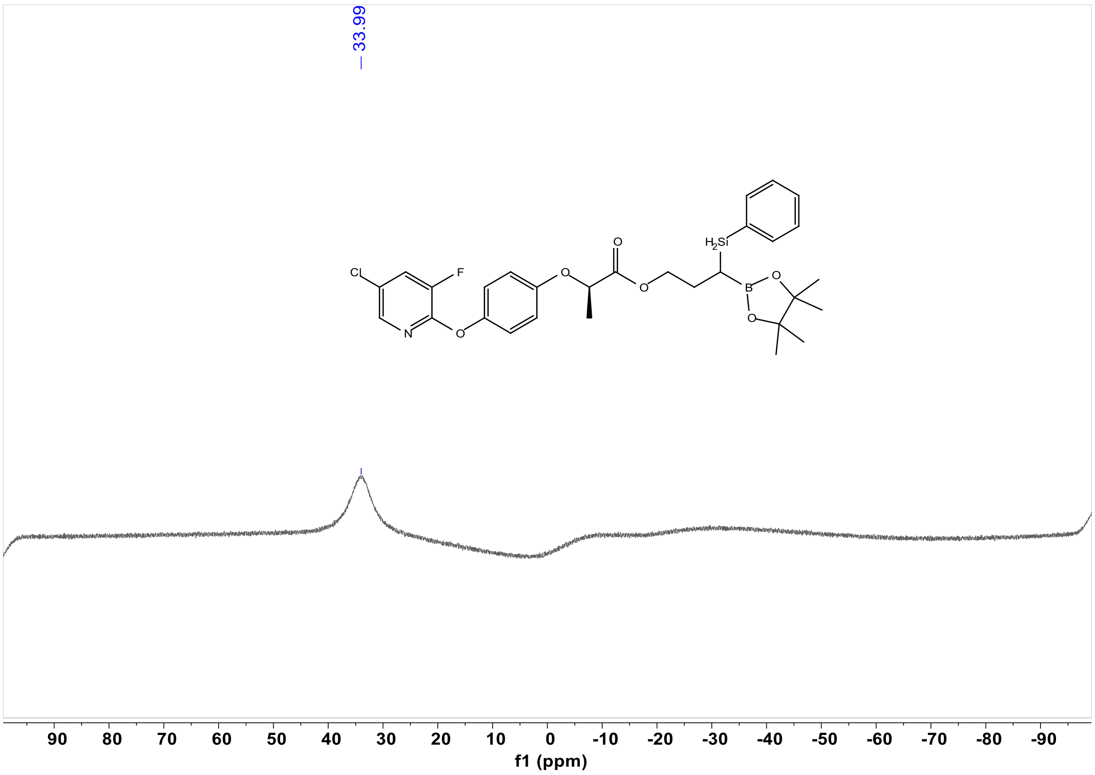

Supplementary Figure 262. ^{11}B NMR spectra for **82**

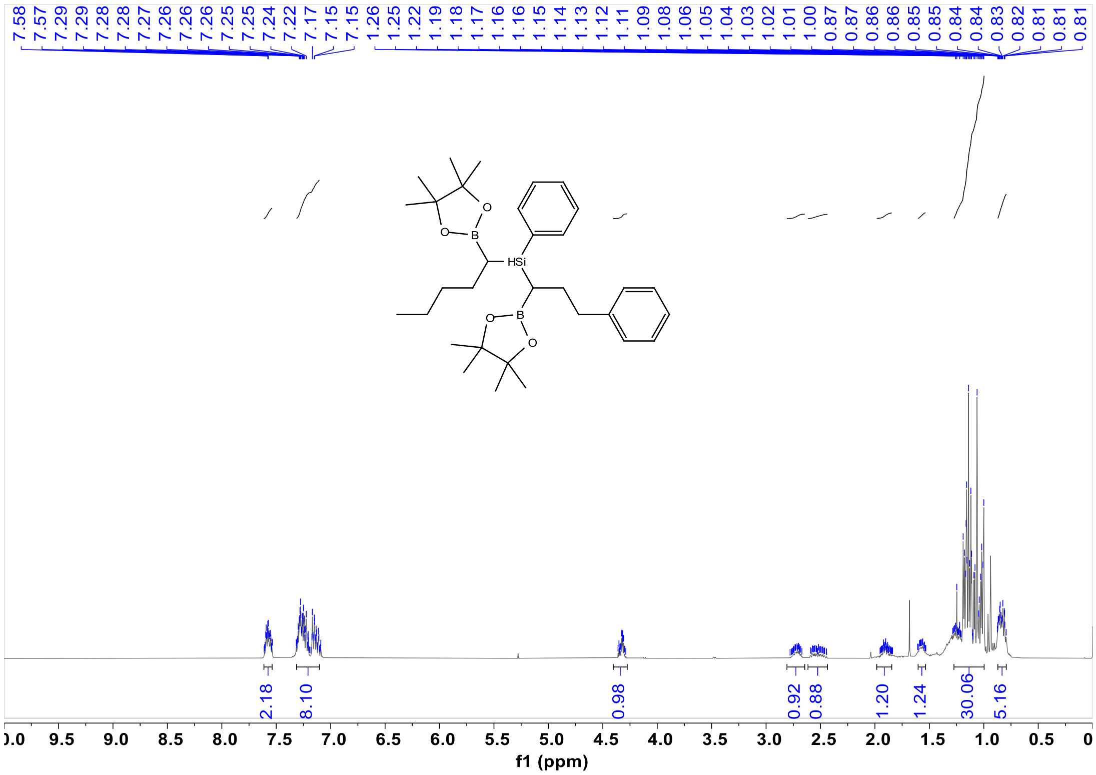

Supplementary Figure 263. ^{1}H NMR spectra for **83**

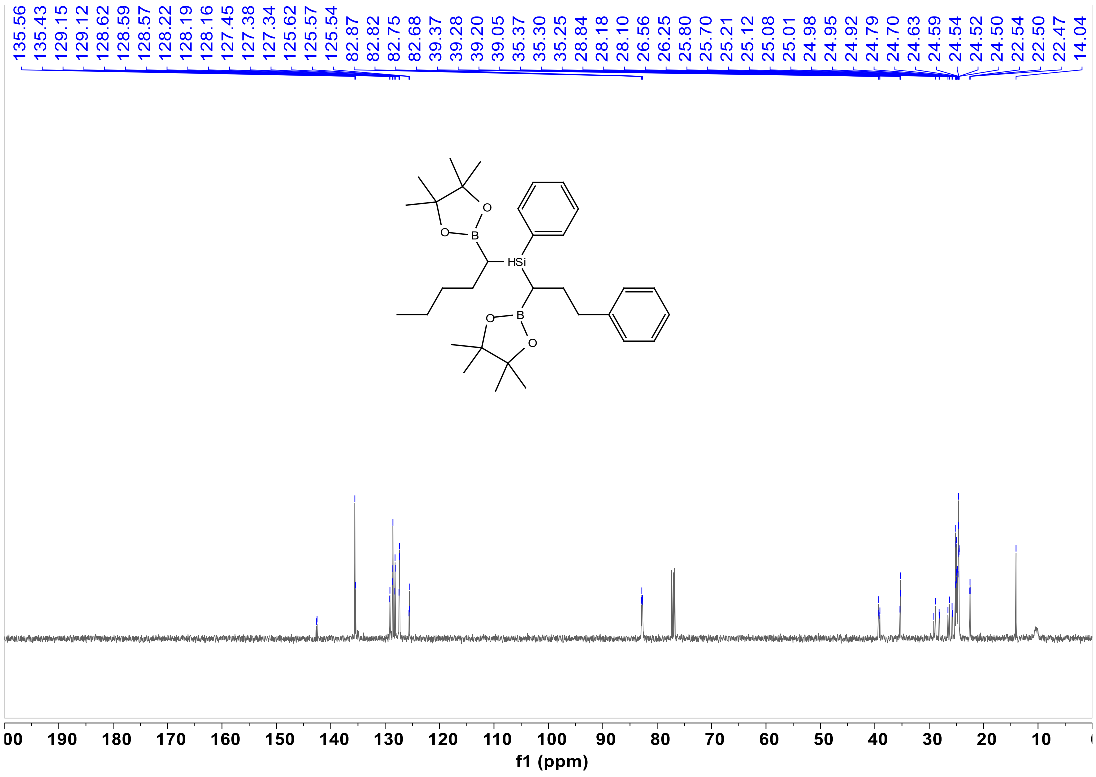

Supplementary Figure 264. ^{13}C NMR spectra for **83**

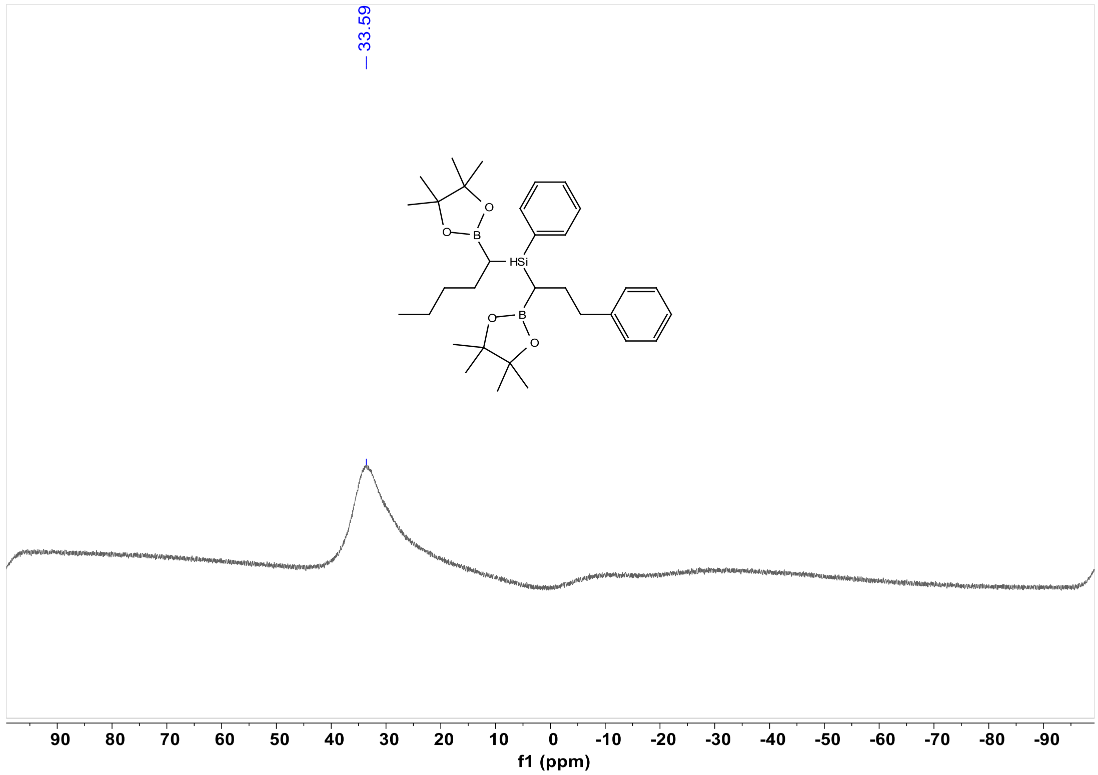

Supplementary Figure 265. ^{11}B NMR spectra for **83**

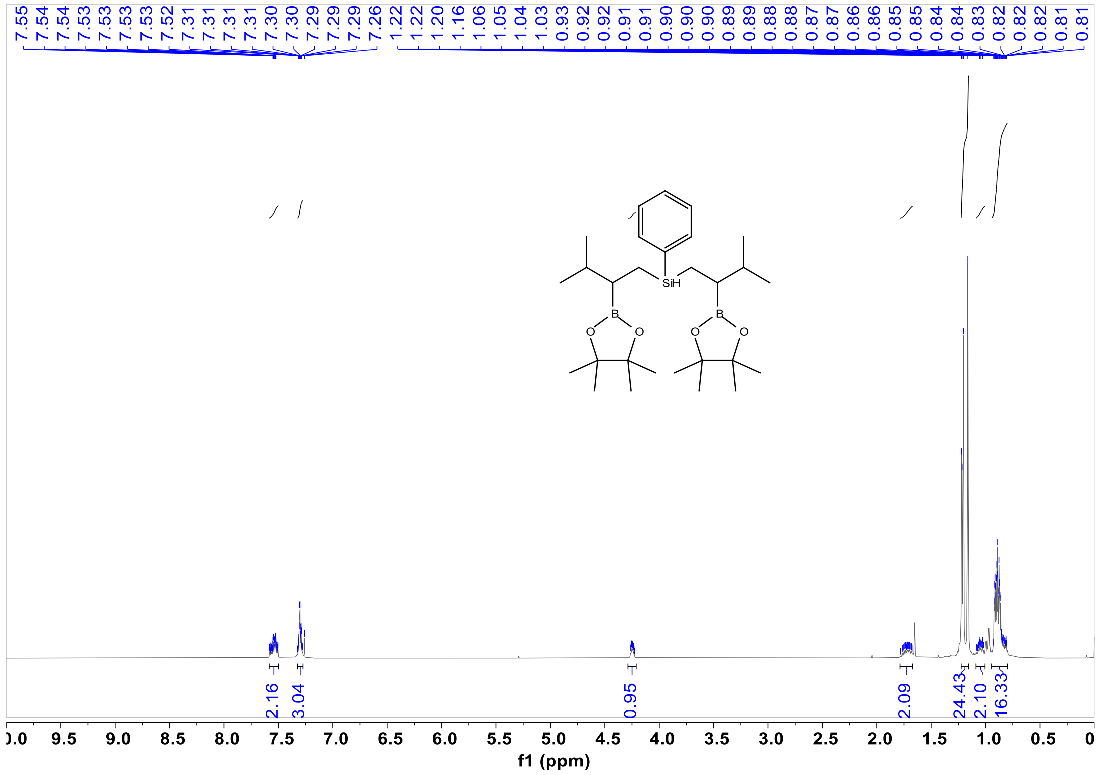

Supplementary Figure 266. ^{1}H NMR spectra for **84**

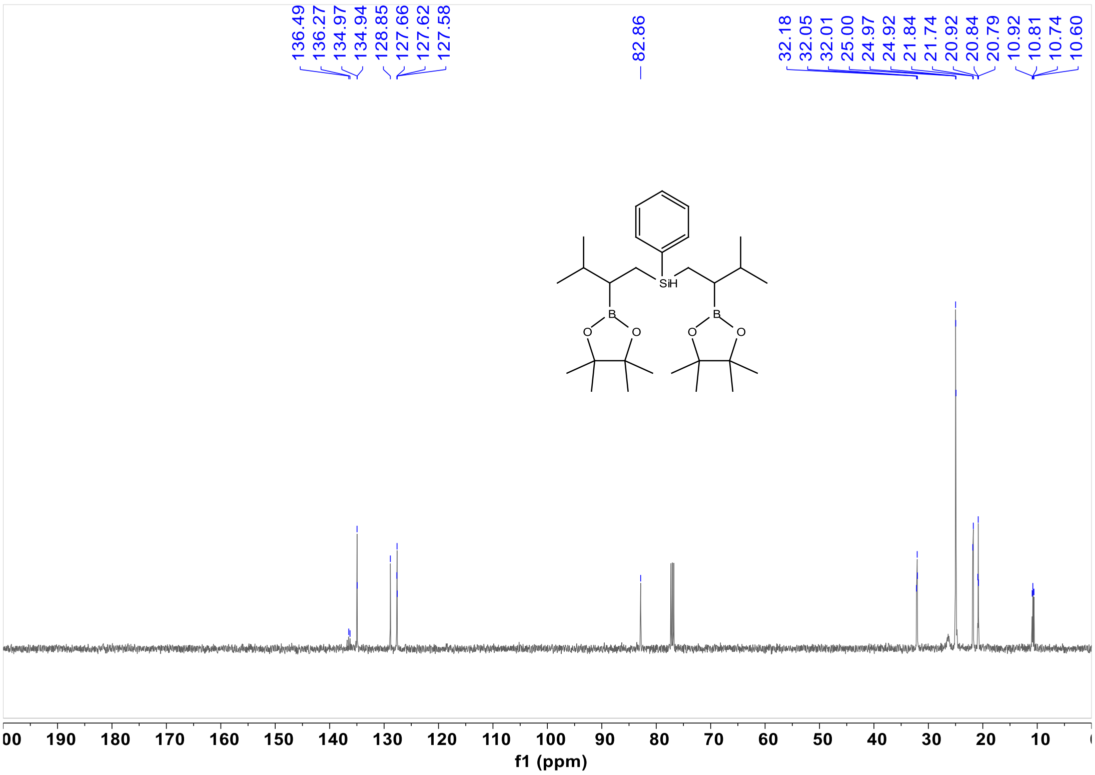

Supplementary Figure 267. ^{13}C NMR spectra for **84**

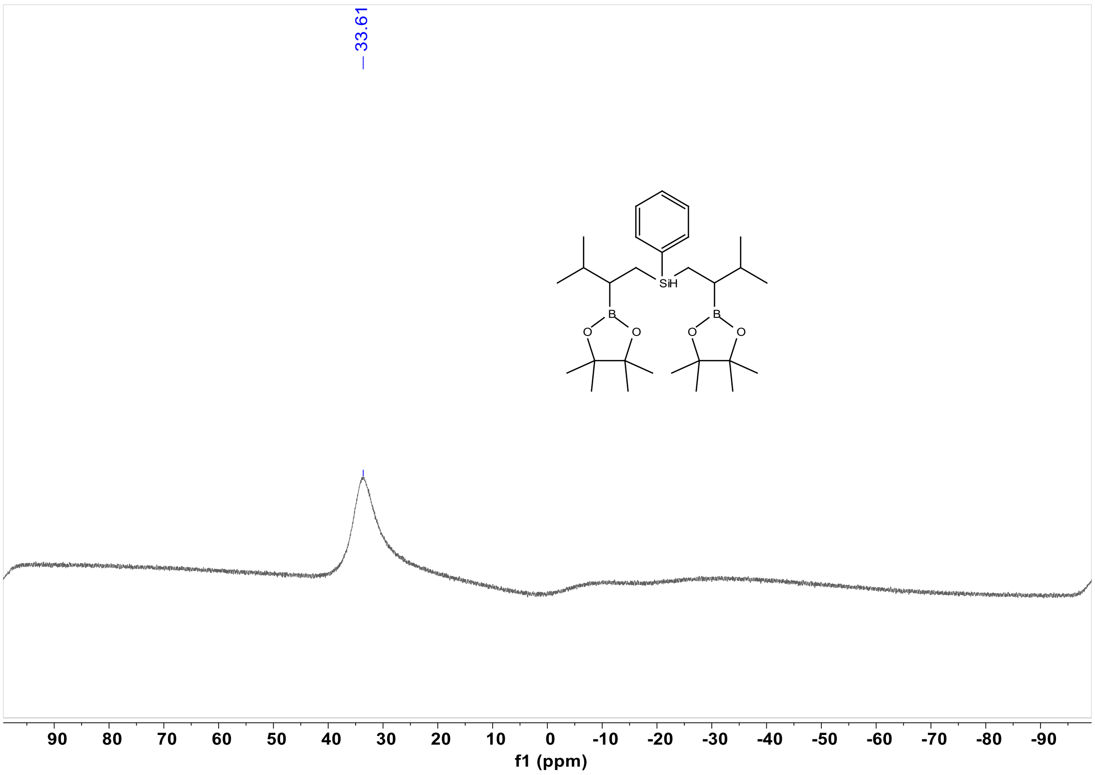

Supplementary Figure 268. ^{11}B NMR spectra for **84**

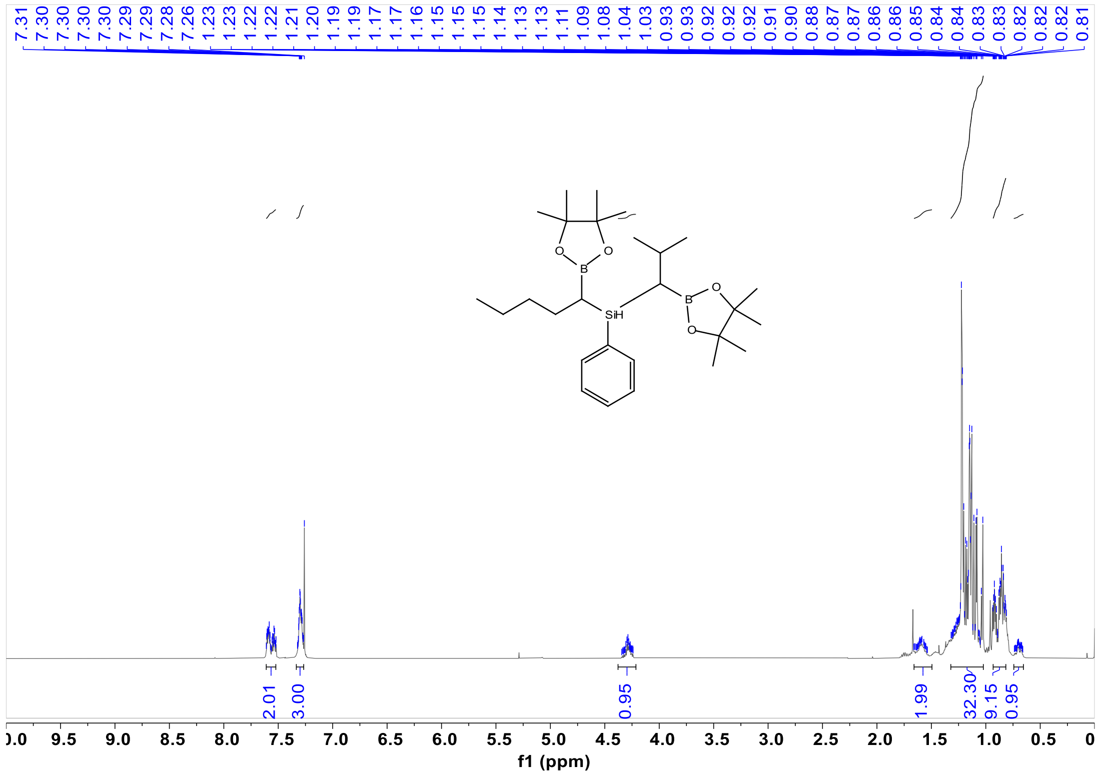

Supplementary Figure 269. ^{1}H NMR spectra for **85**

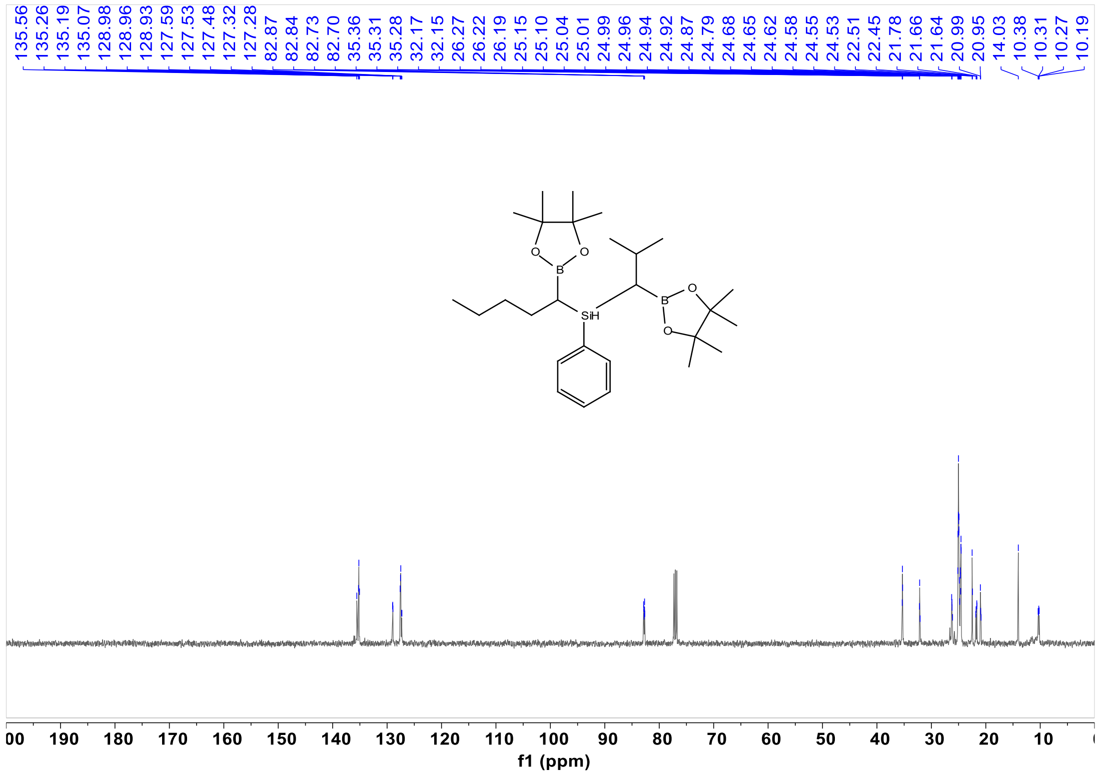

Supplementary Figure 270. ^{13}C NMR spectra for **85**

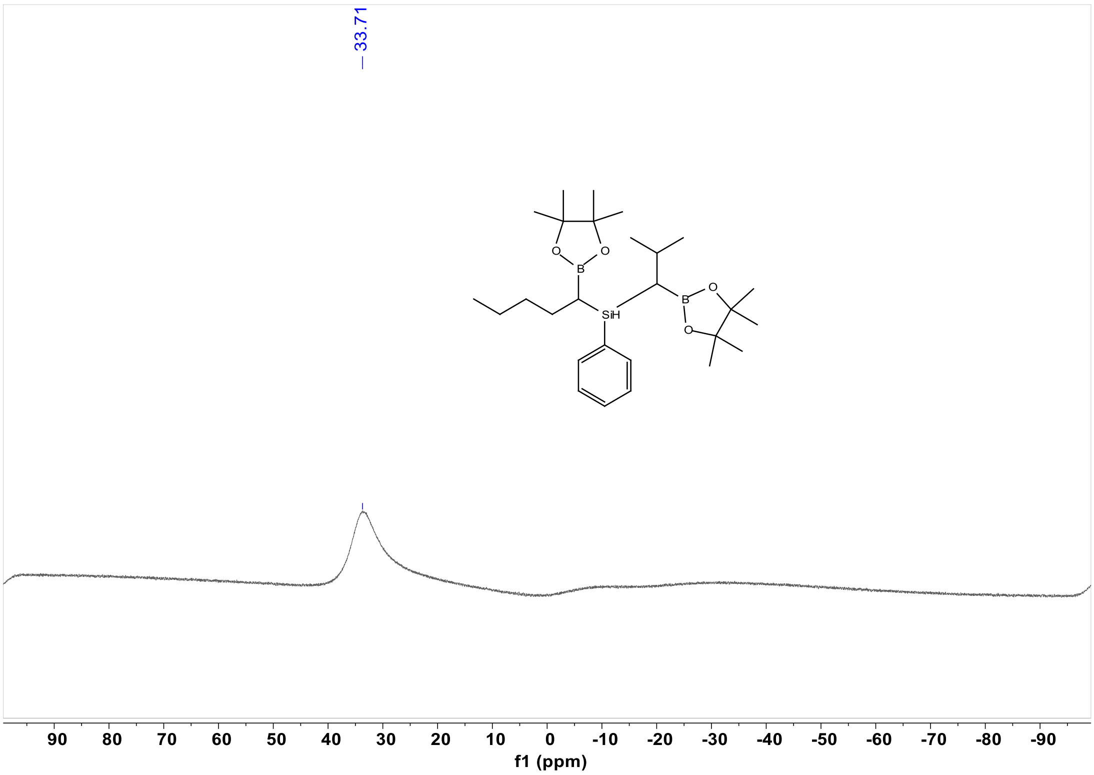

Supplementary Figure 271. ^{11}B NMR spectra for **85**

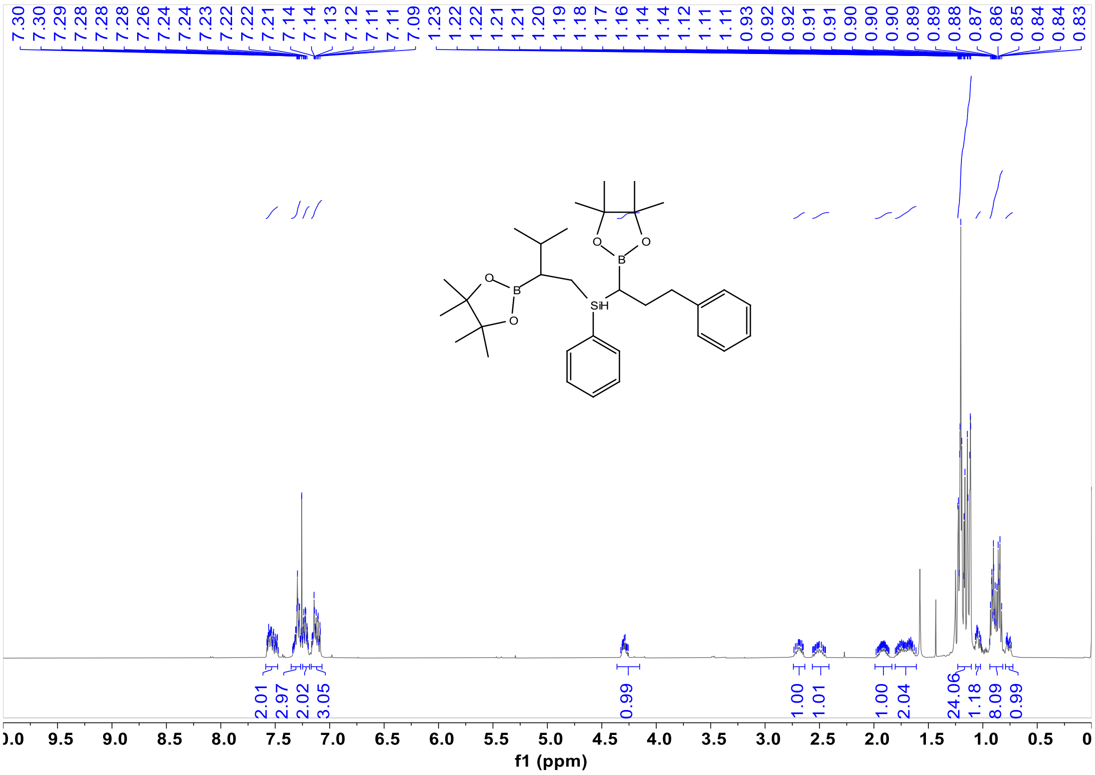

Supplementary Figure 272. ^{1}H NMR spectra for **86**

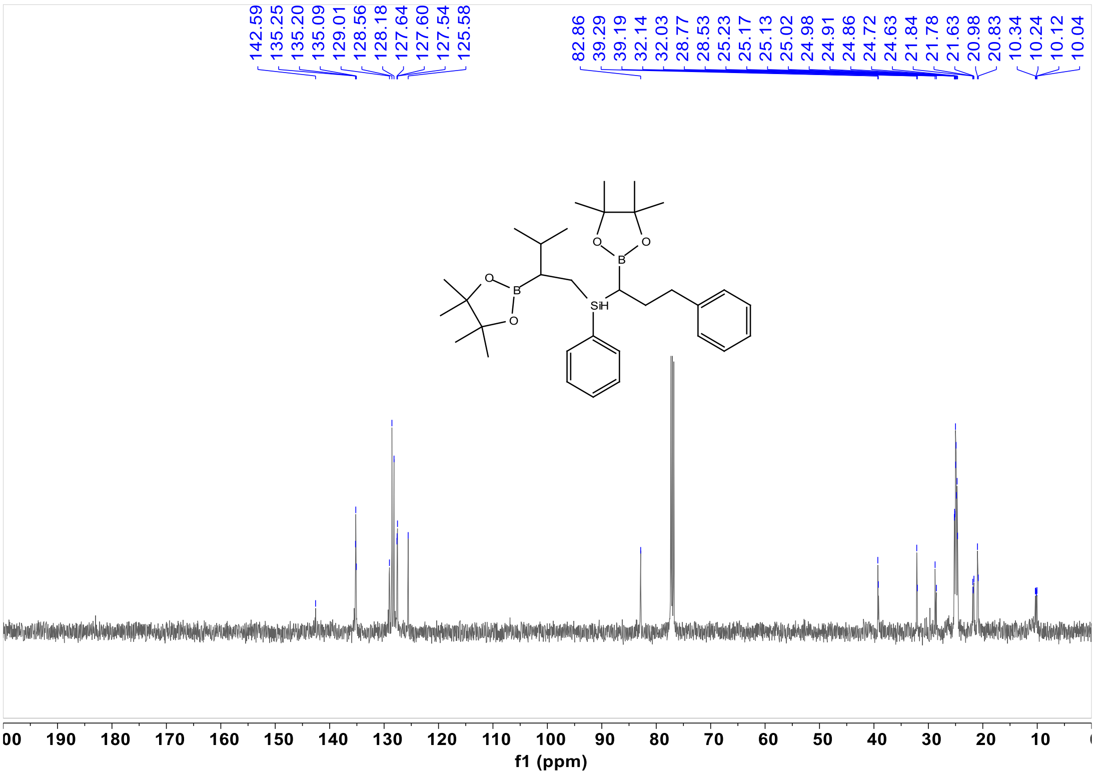

Supplementary Figure 273. ^{13}C NMR spectra for **86**

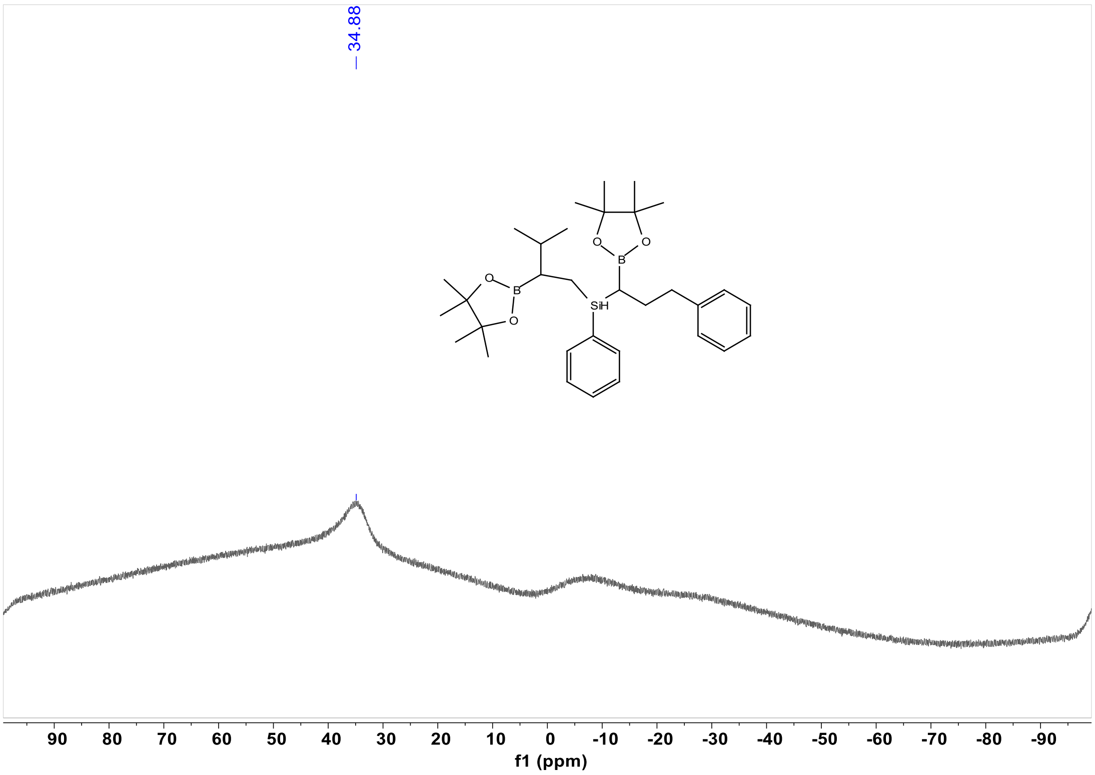

Supplementary Figure 274. ^{11}B NMR spectra for **86**

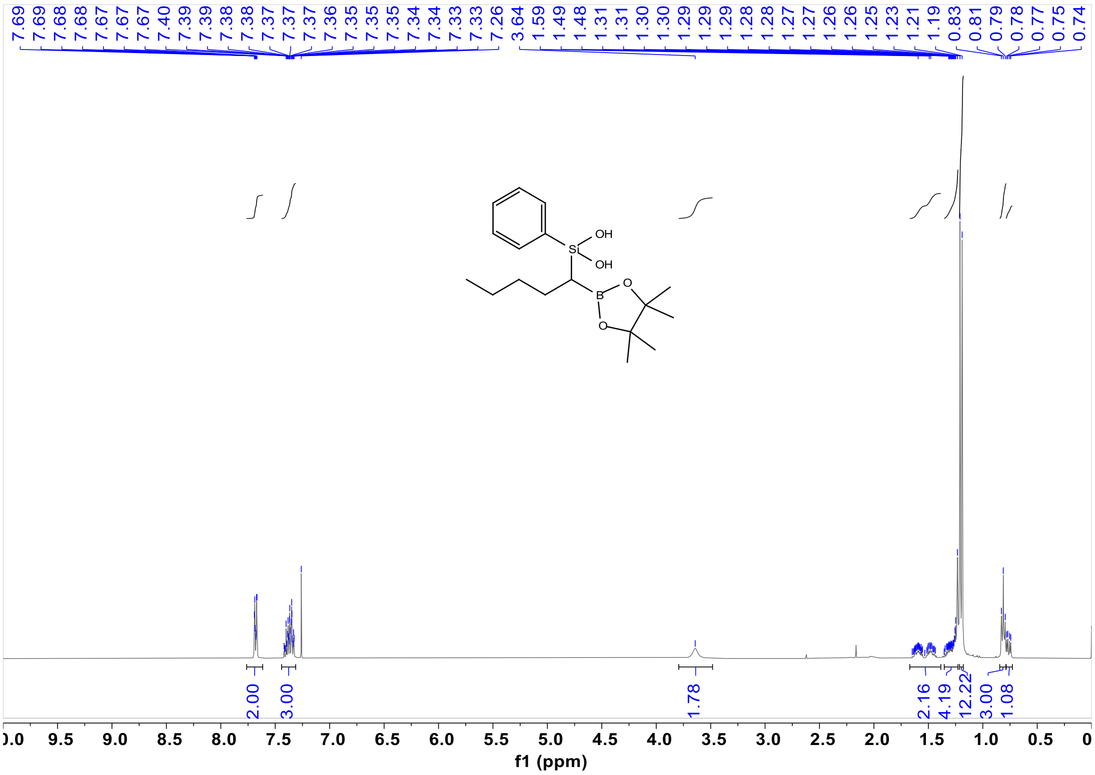

Supplementary Figure 275. ^{1}H NMR spectra for **87**

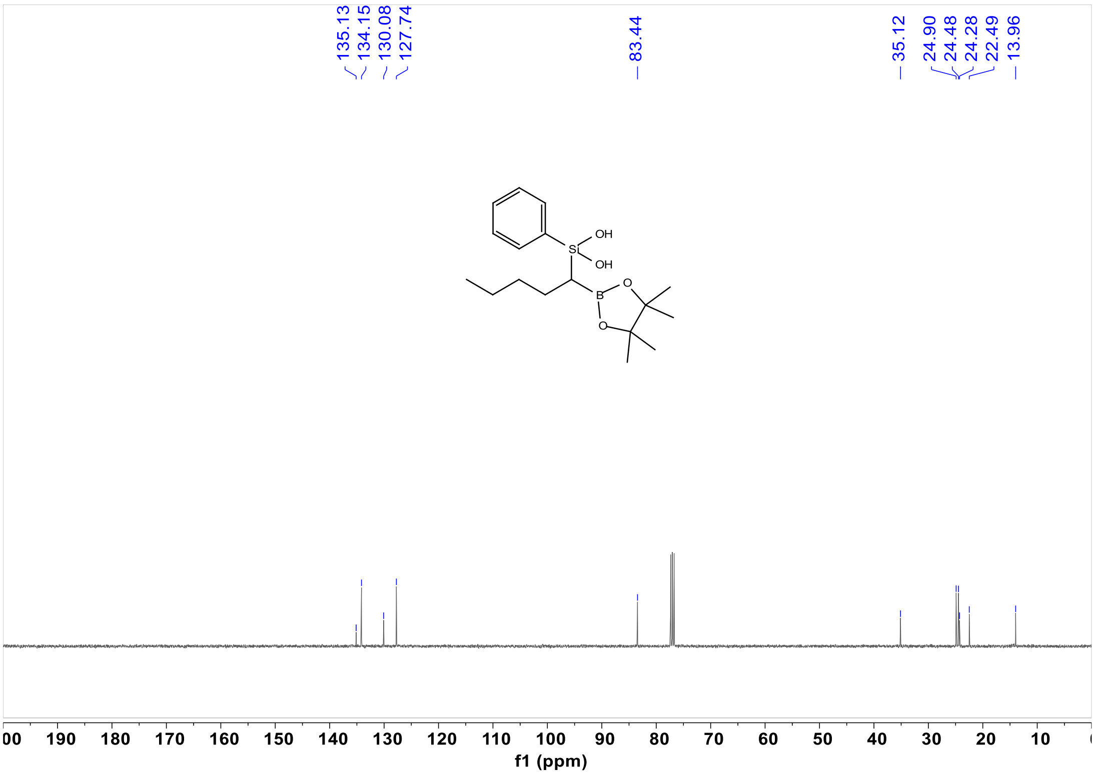

Supplementary Figure 276. ^{13}C NMR spectra for **87**

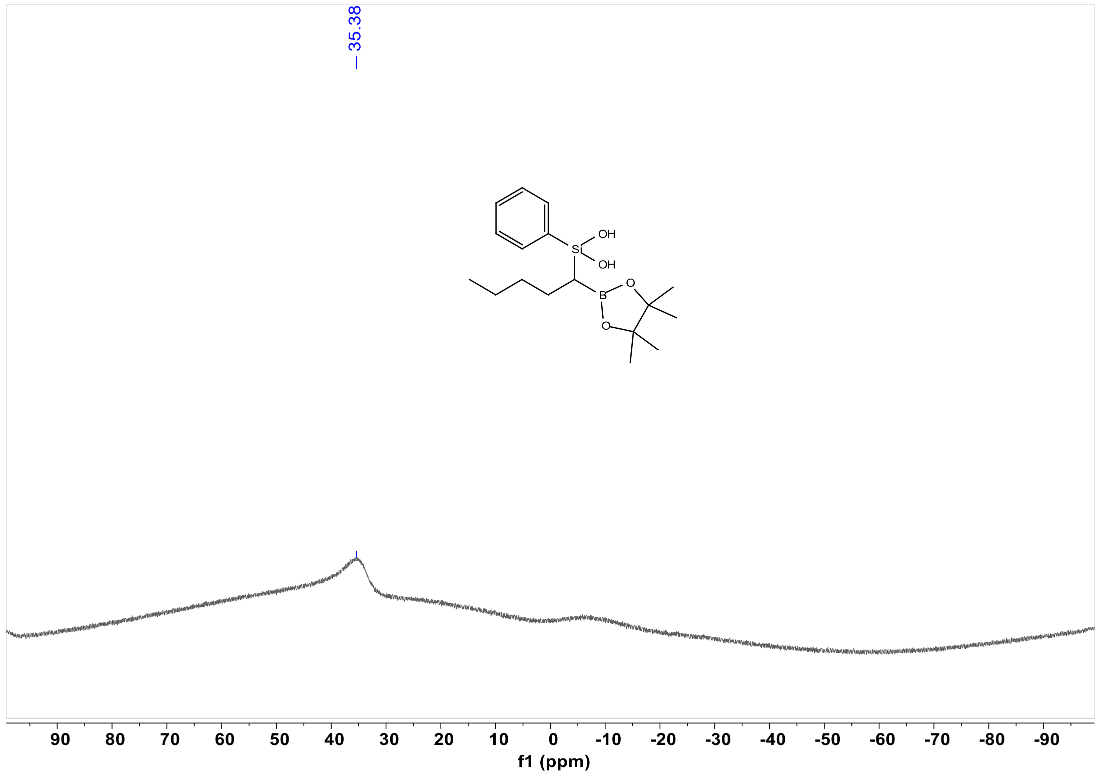

Supplementary Figure 277. ^{11}B NMR spectra for **87**

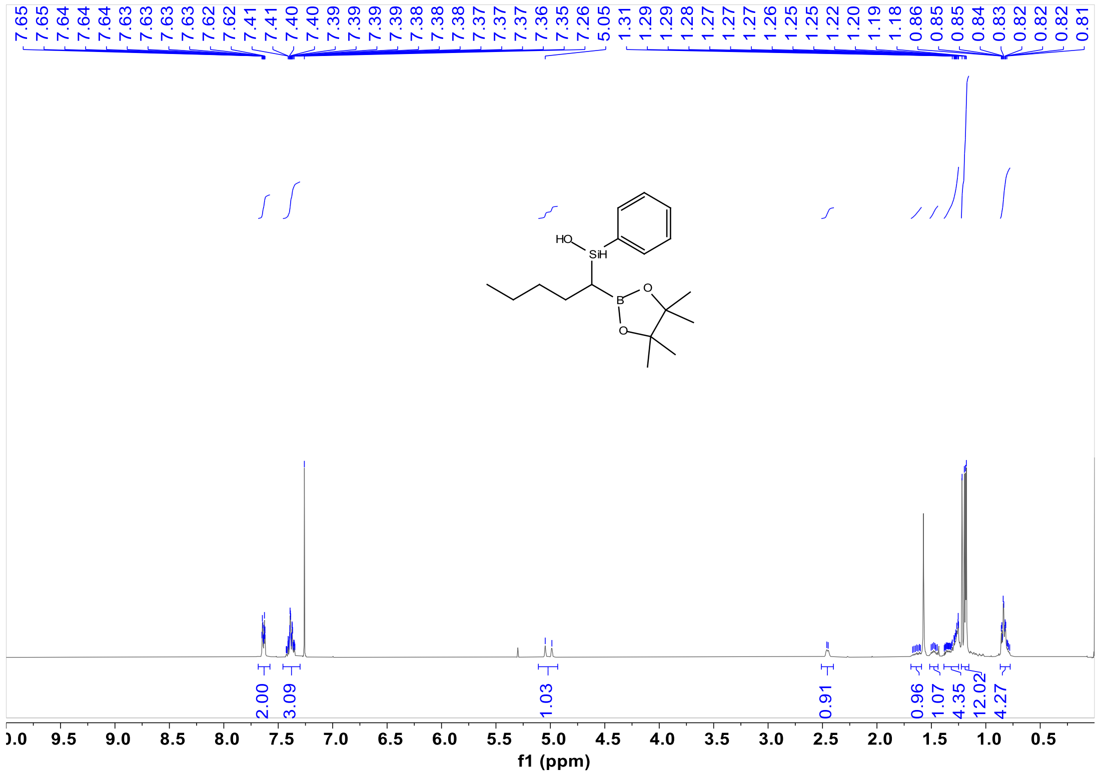

Supplementary Figure 278. ^{1}H NMR spectra for **88**

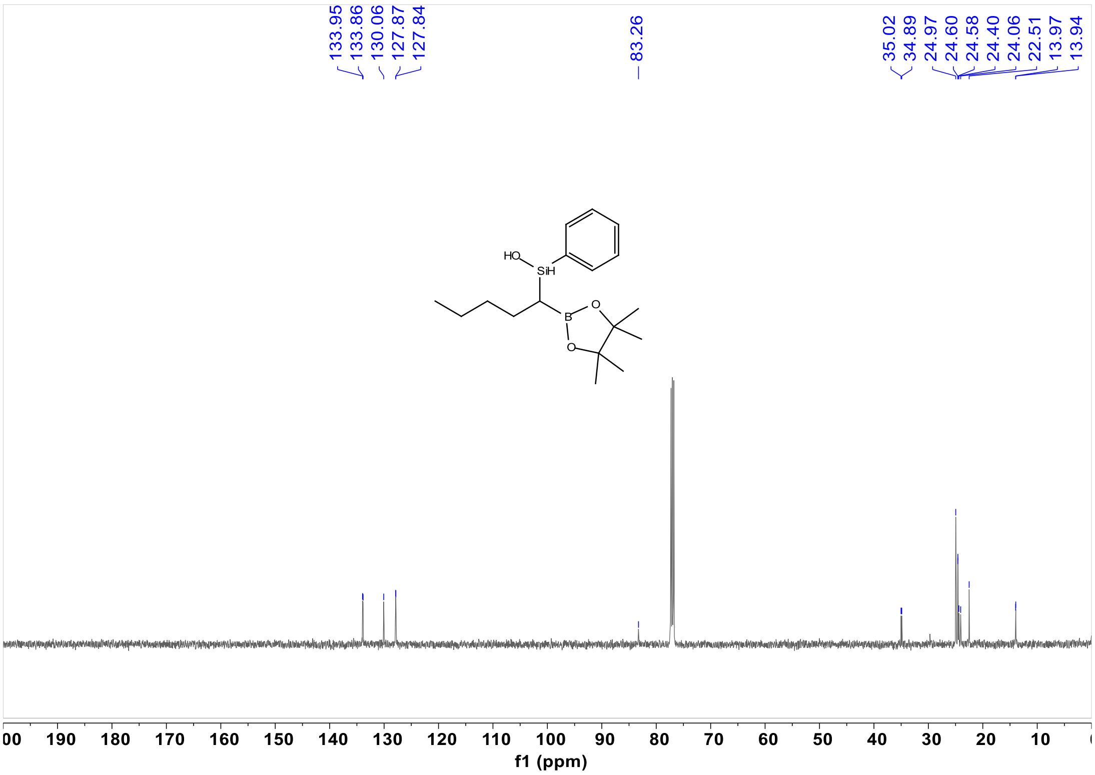

Supplementary Figure 279. ^{13}C NMR spectra for **88**

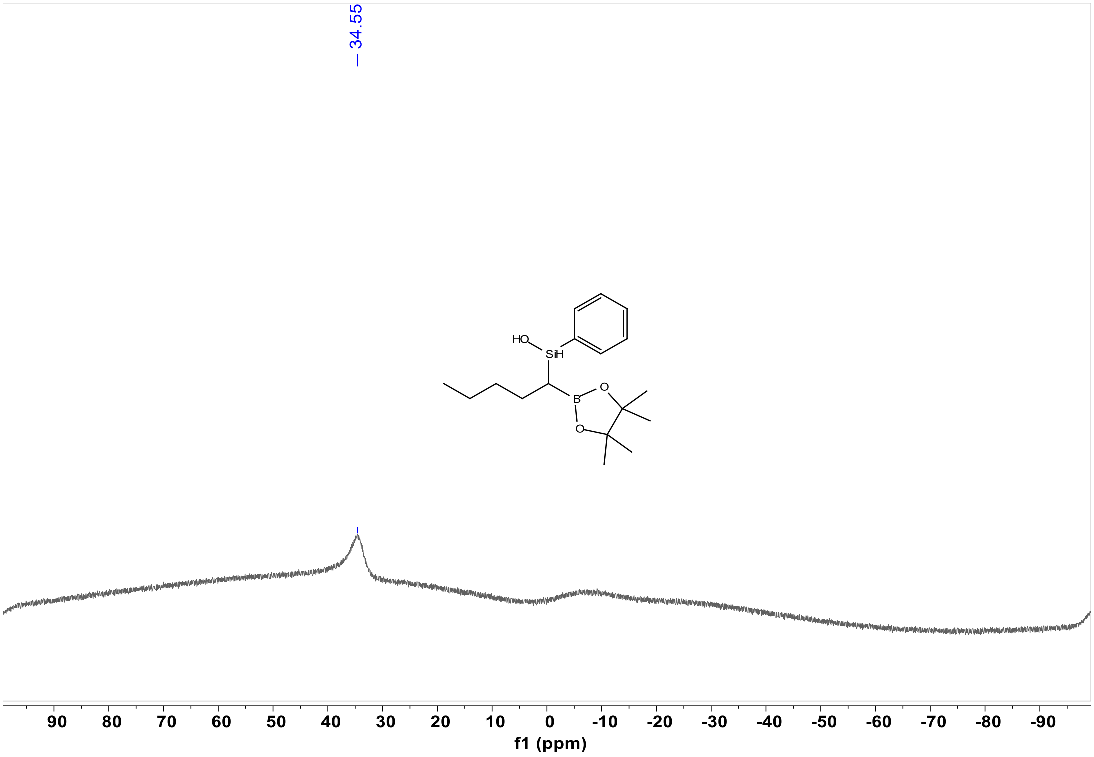

Supplementary Figure 280. ^{11}B NMR spectra for **88**

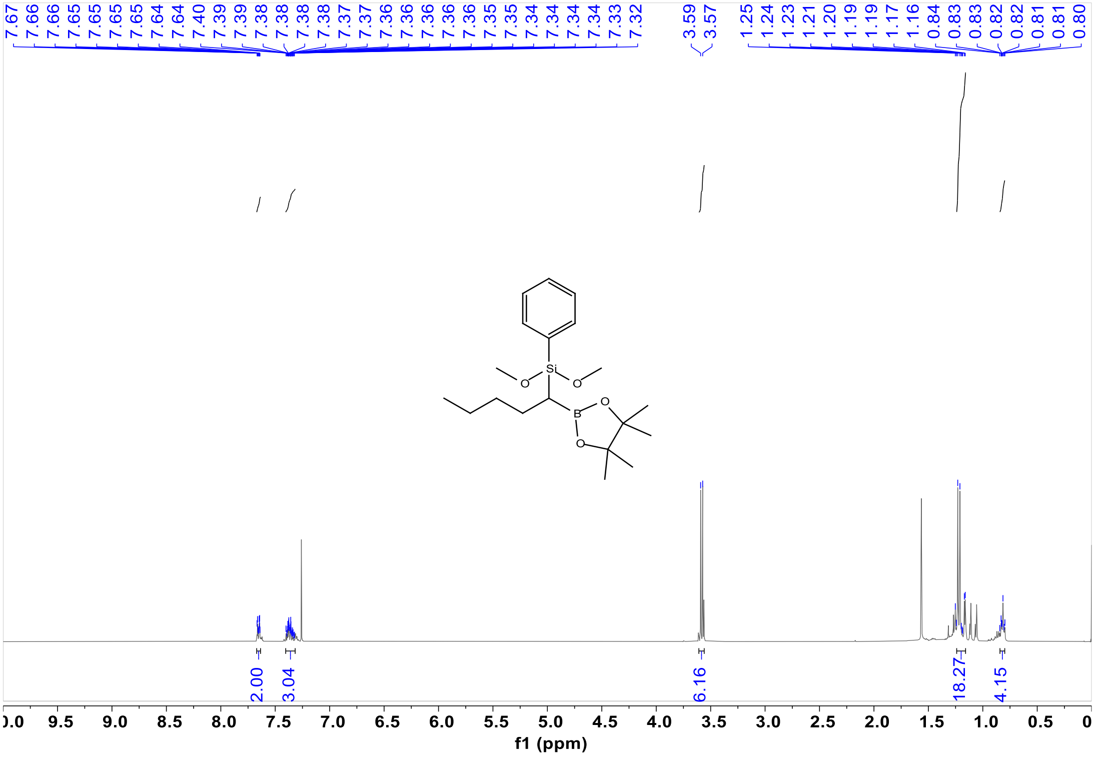

Supplementary Figure 281. ^{1}H NMR spectra for **89**

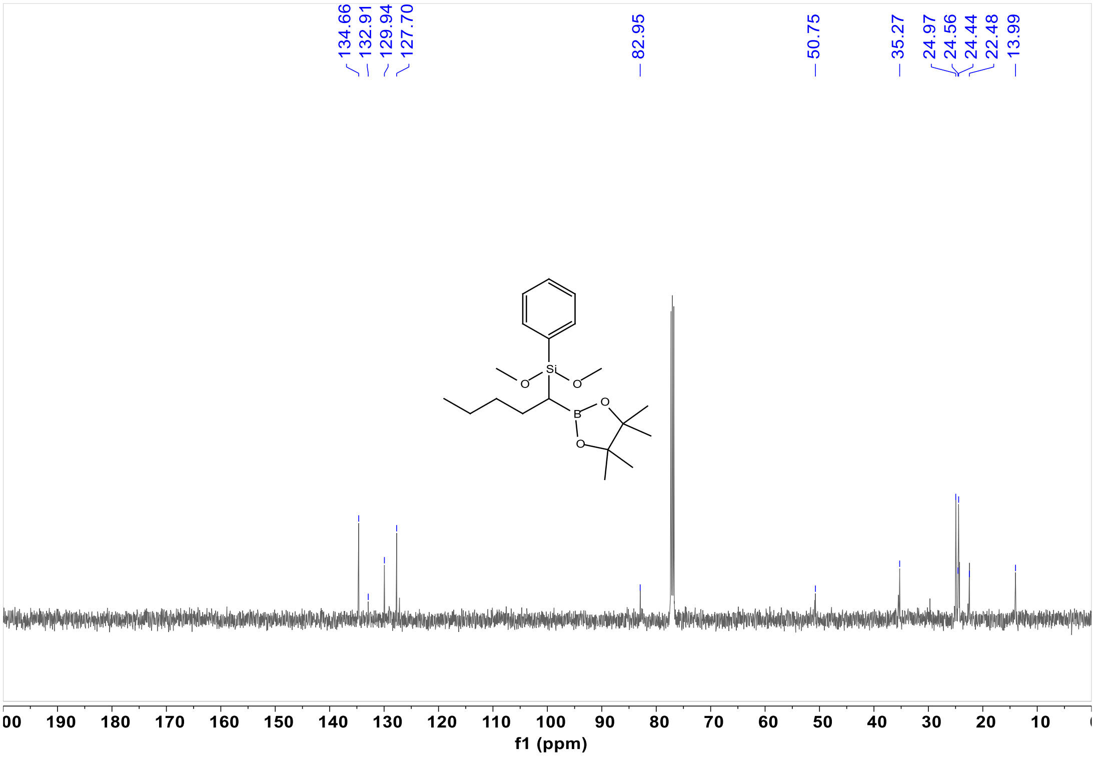

Supplementary Figure 282. ^{13}C NMR spectra for **89**

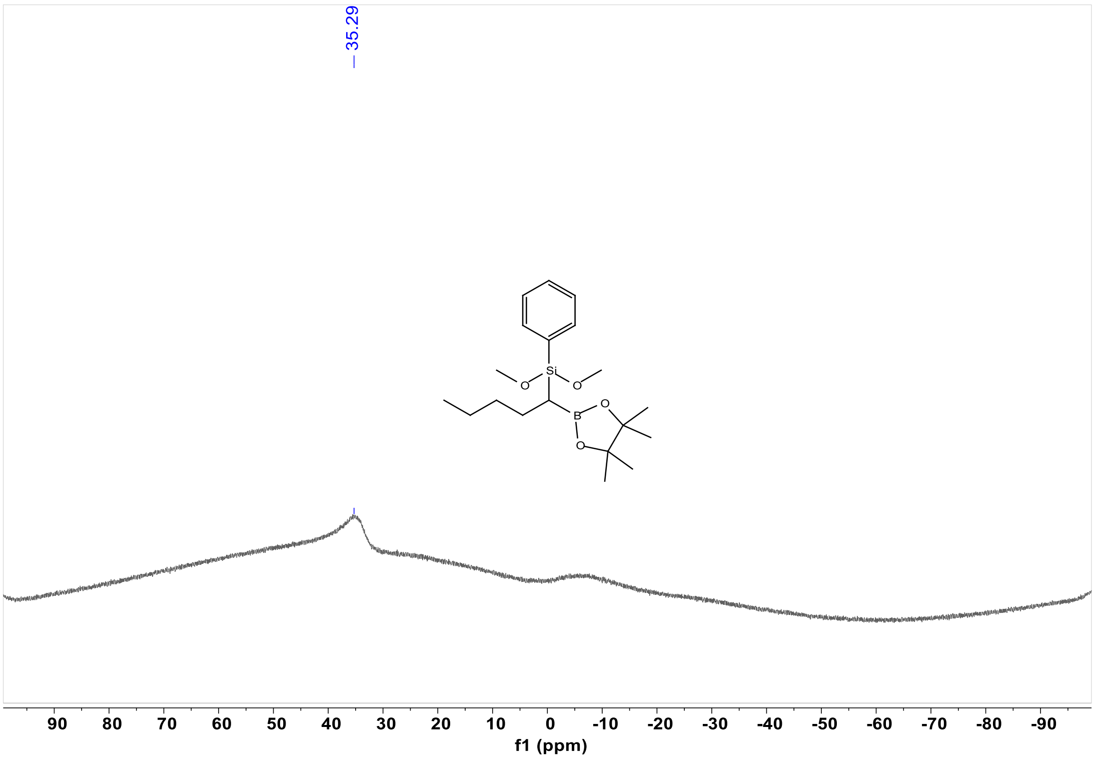

Supplementary Figure 283. ^{11}B NMR spectra for **89**

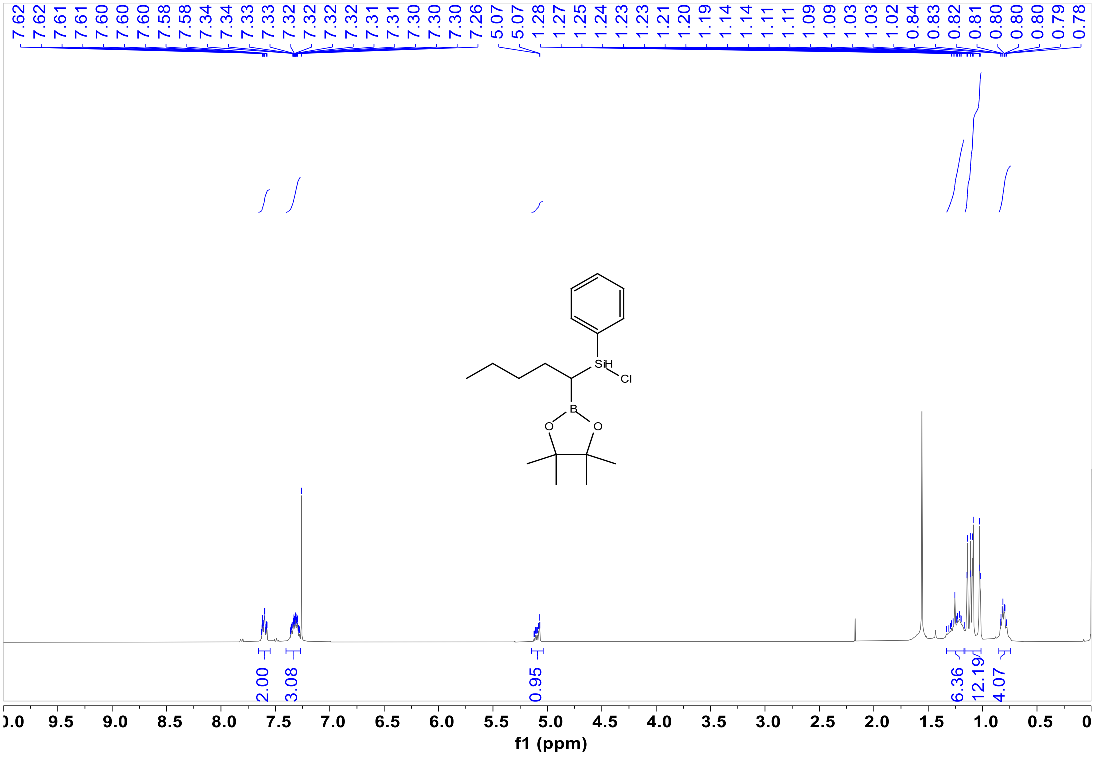

Supplementary Figure 284. ^{1}H NMR spectra for **90**

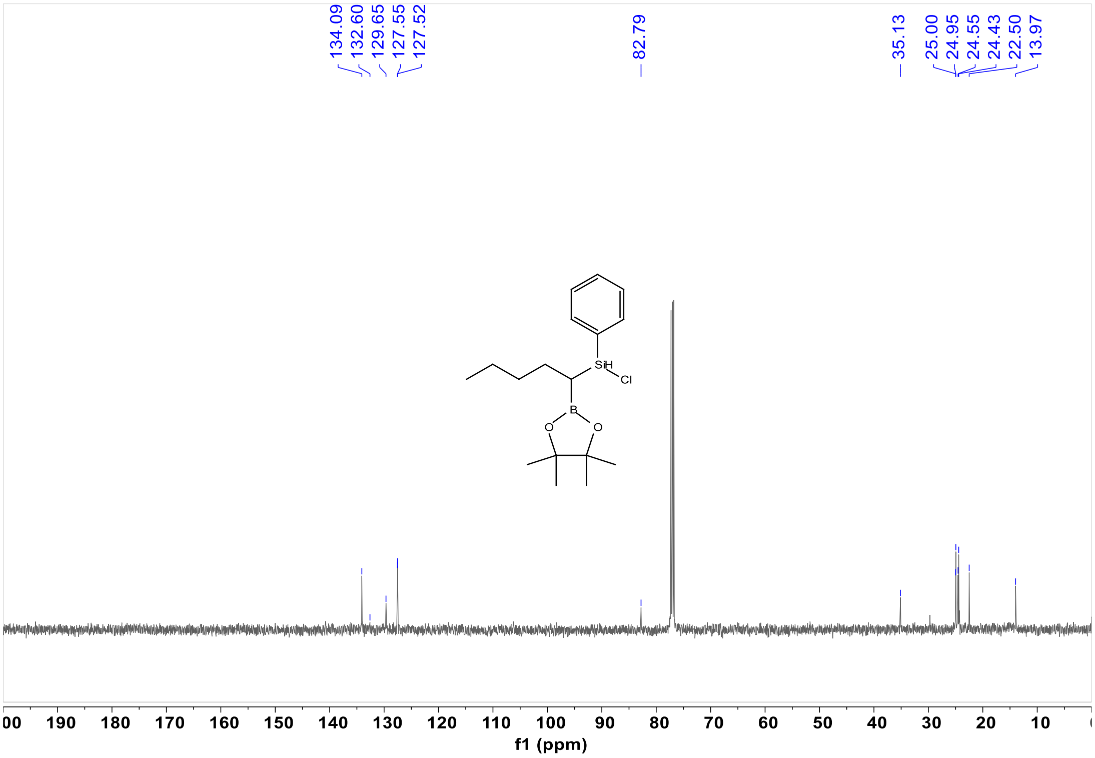

Supplementary Figure 285. ^{13}C NMR spectra for **90**

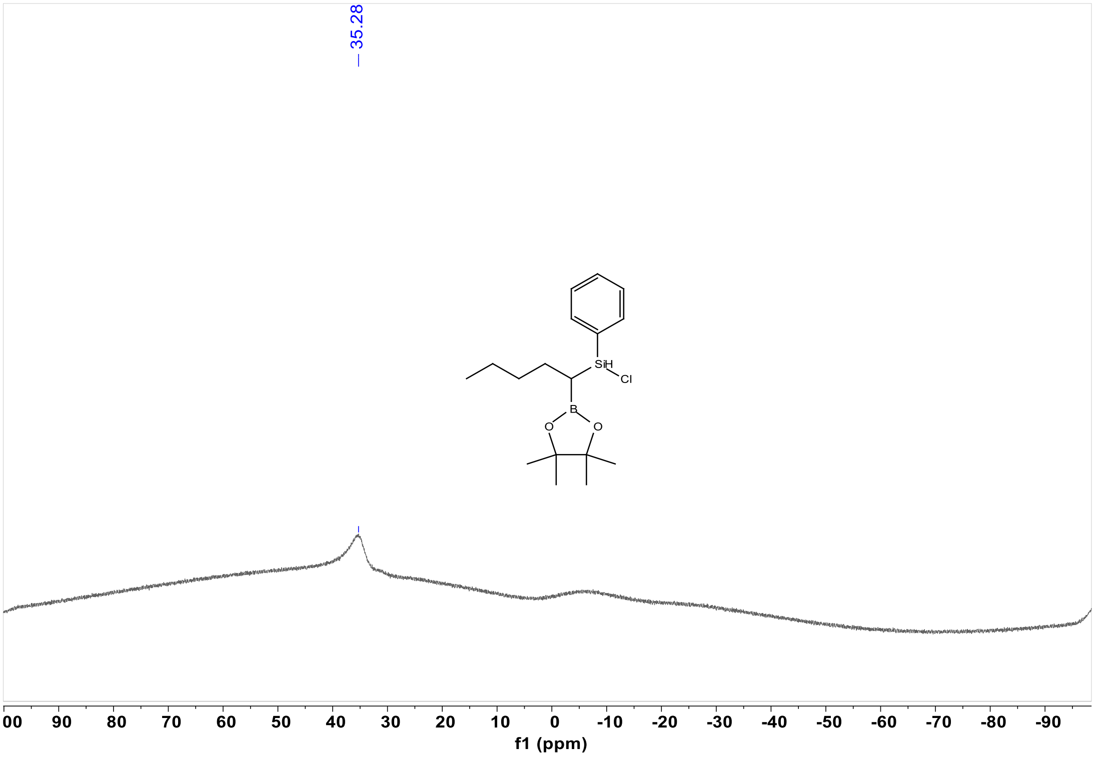

Supplementary Figure 286. ^{11}B NMR spectra for **90**

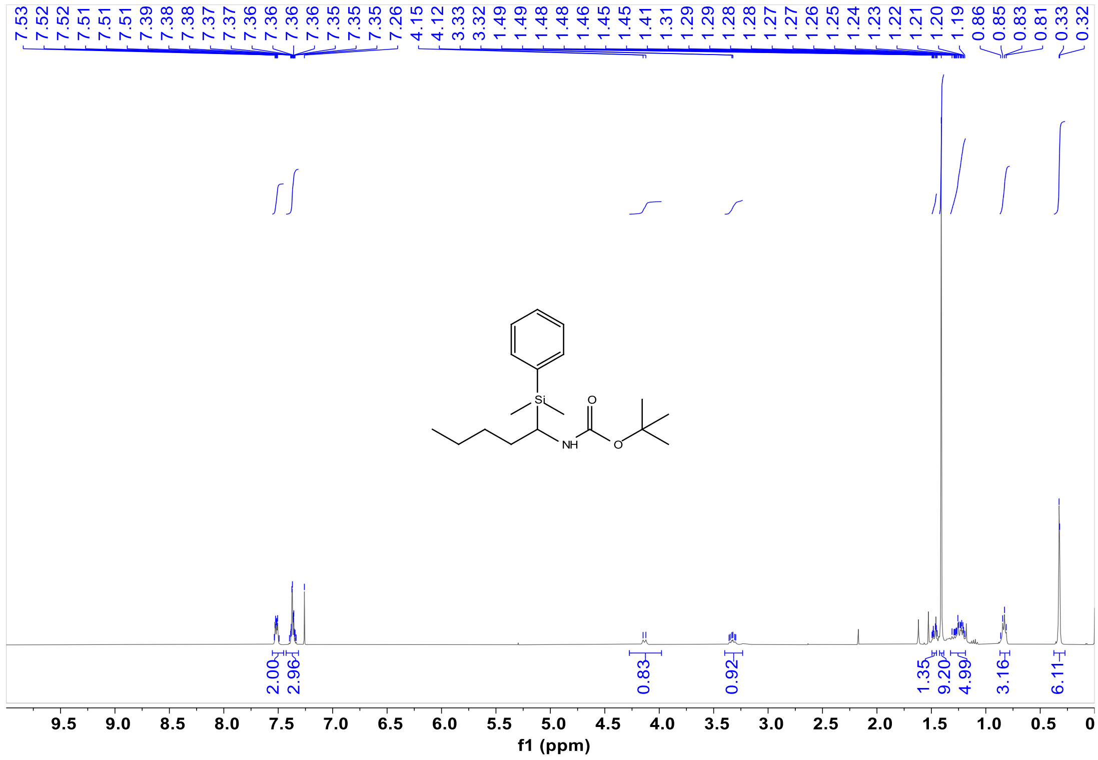

Supplementary Figure 287. ^{1}H NMR spectra for **91**

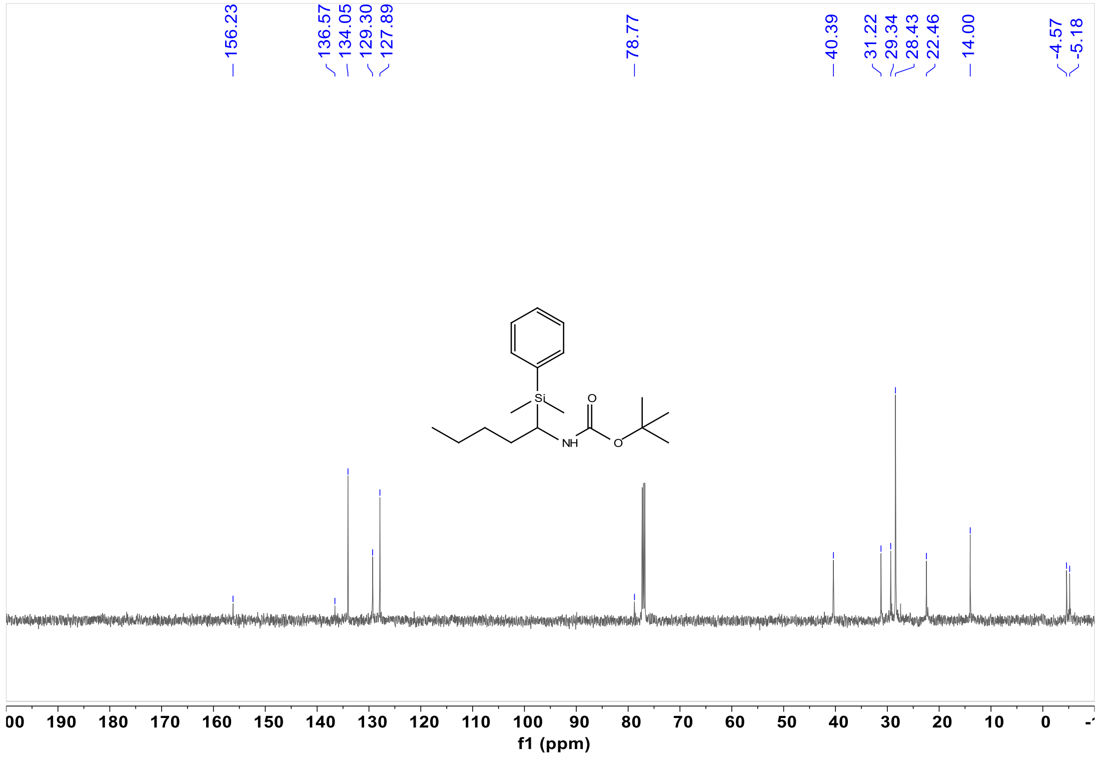

Supplementary Figure 288. ^{13}C NMR spectra for **91**

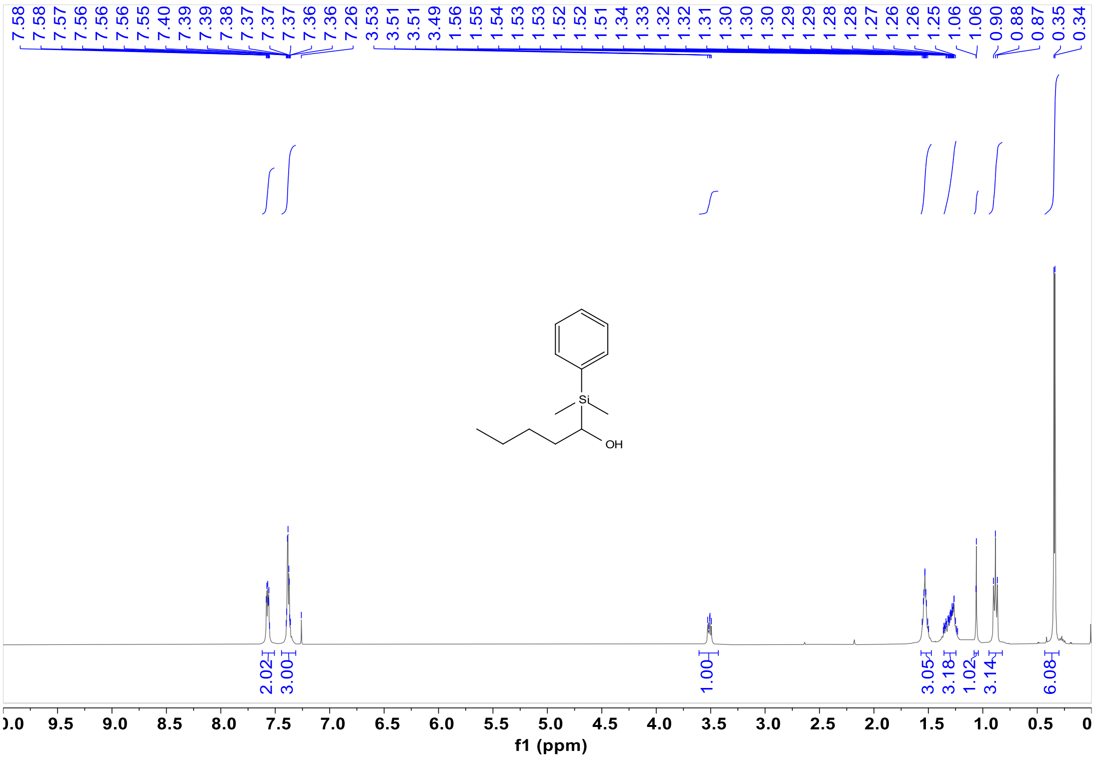

Supplementary Figure 289. ^{1}H NMR spectra for **92**

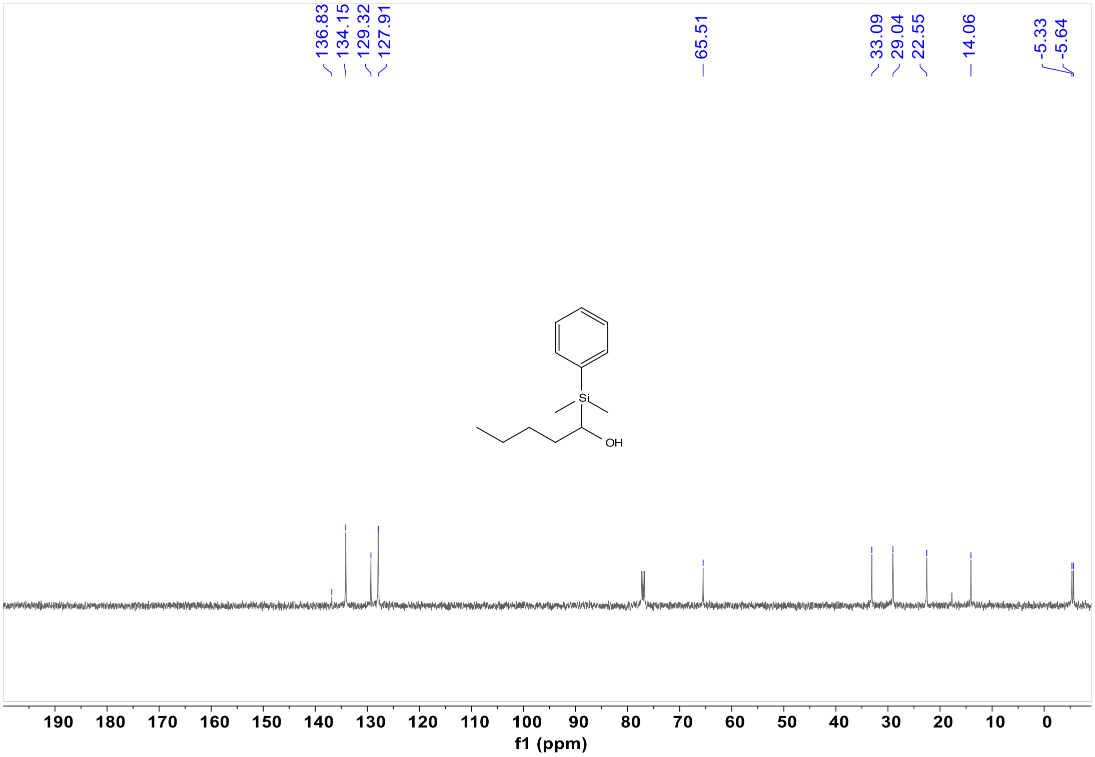

Supplementary Figure 290. ^{13}C NMR spectra for **92**

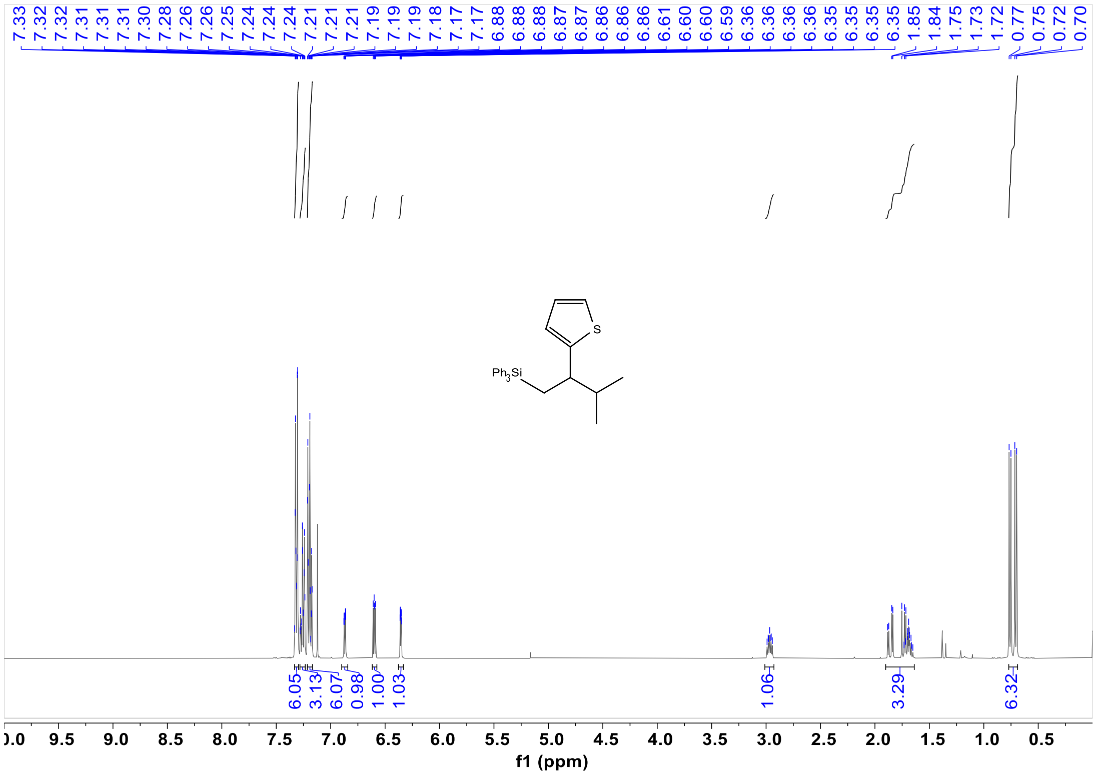

Supplementary Figure 291. ^{1}H NMR spectra for **93**

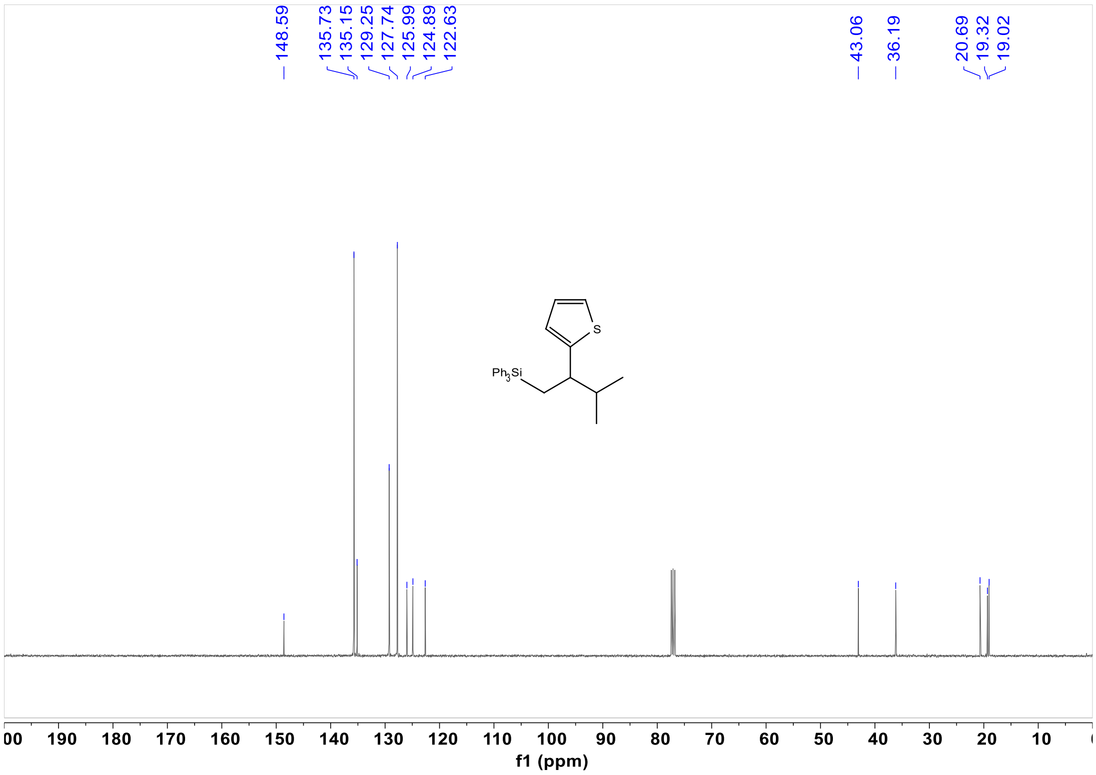

Supplementary Figure 292. ^{13}C NMR spectra for **93**

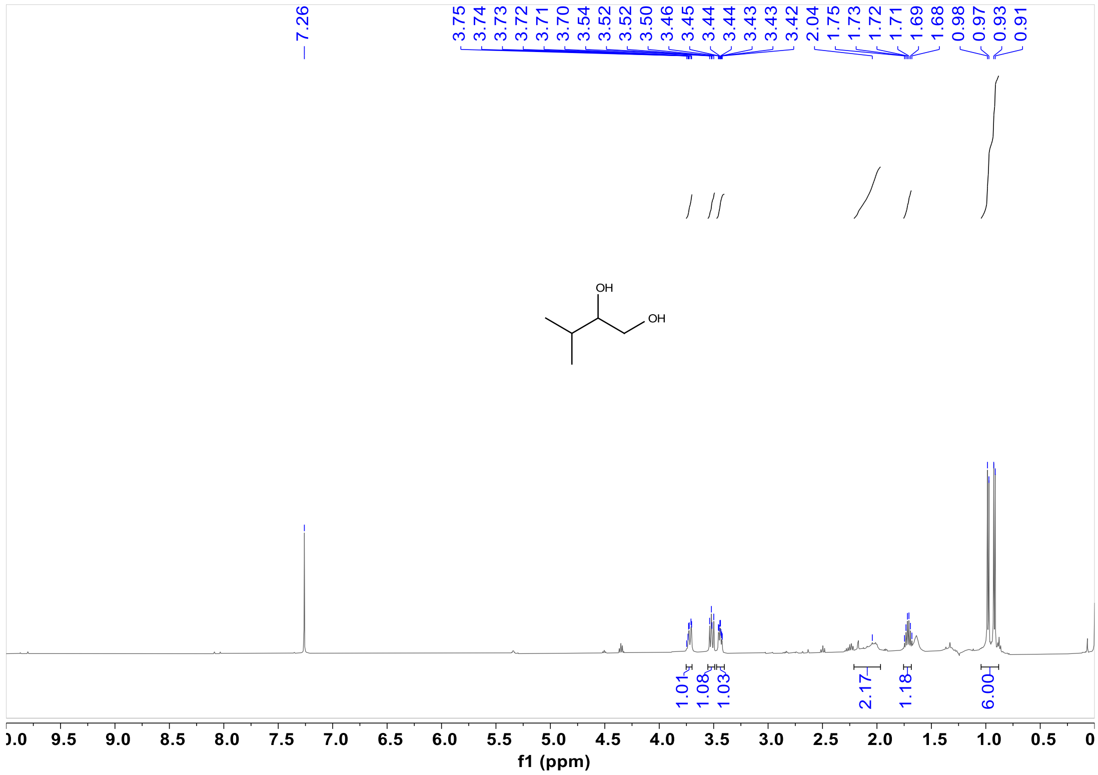

Supplementary Figure 293. ¹H NMR spectra for **94**

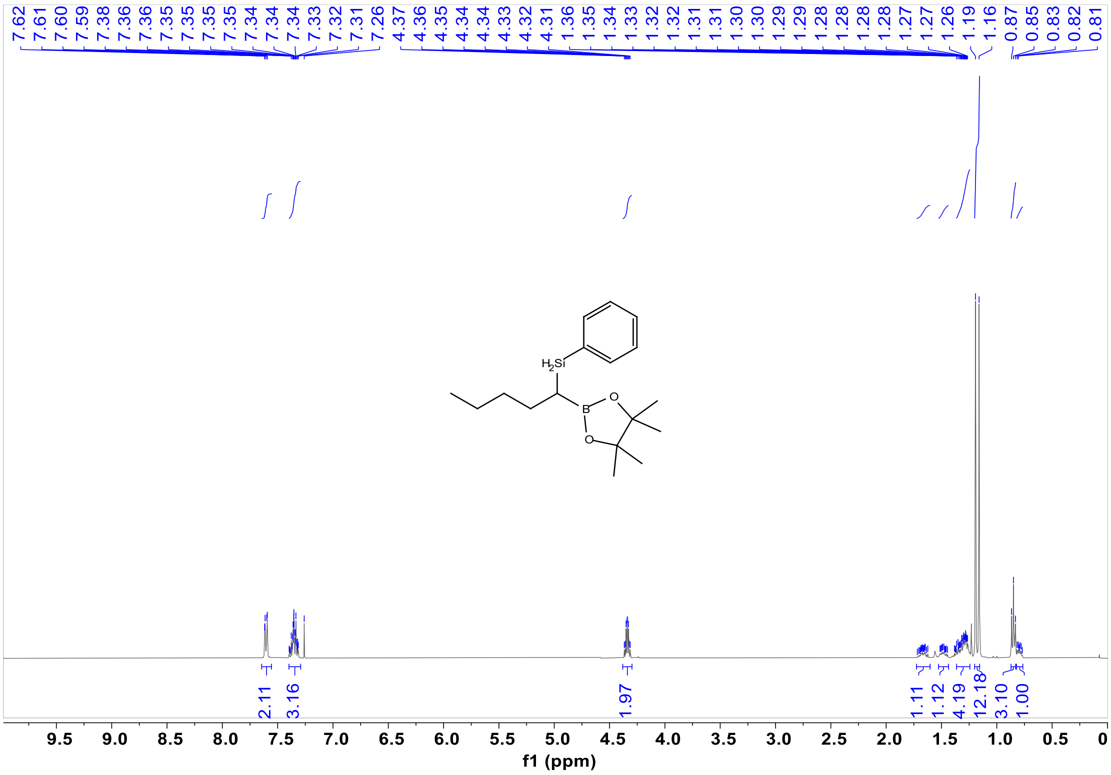

Supplementary Figure 294. ¹H NMR spectra for **95**

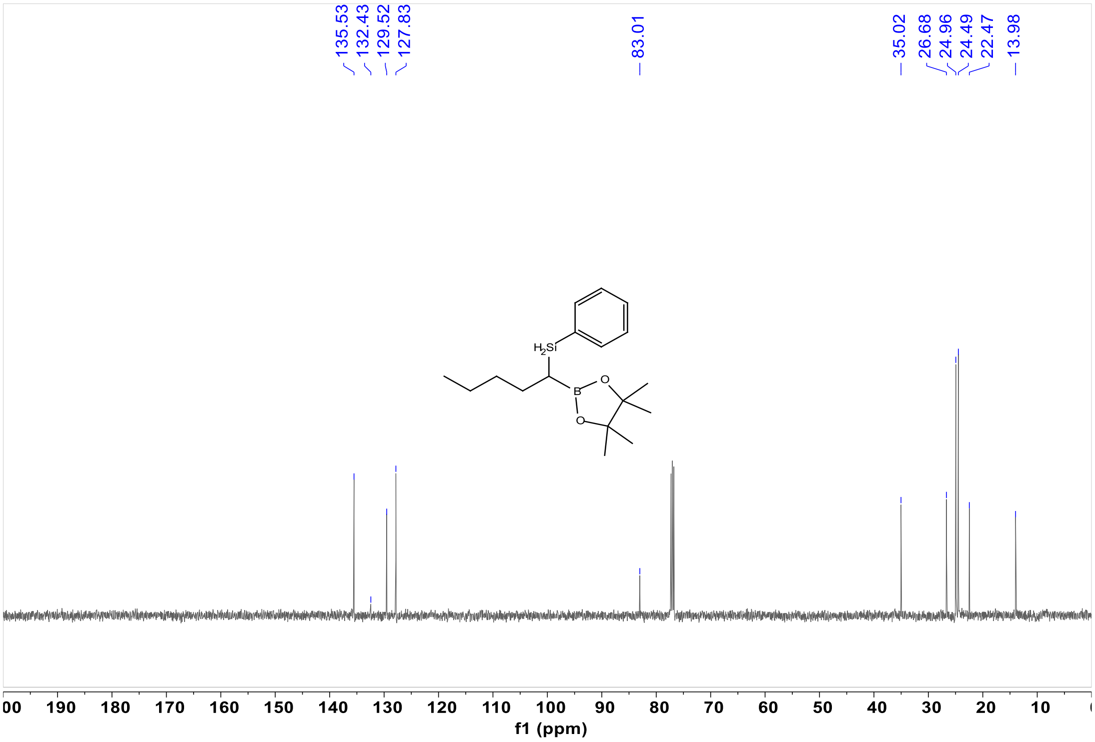

Supplementary Figure 295. ^{13}C NMR spectra for **95**

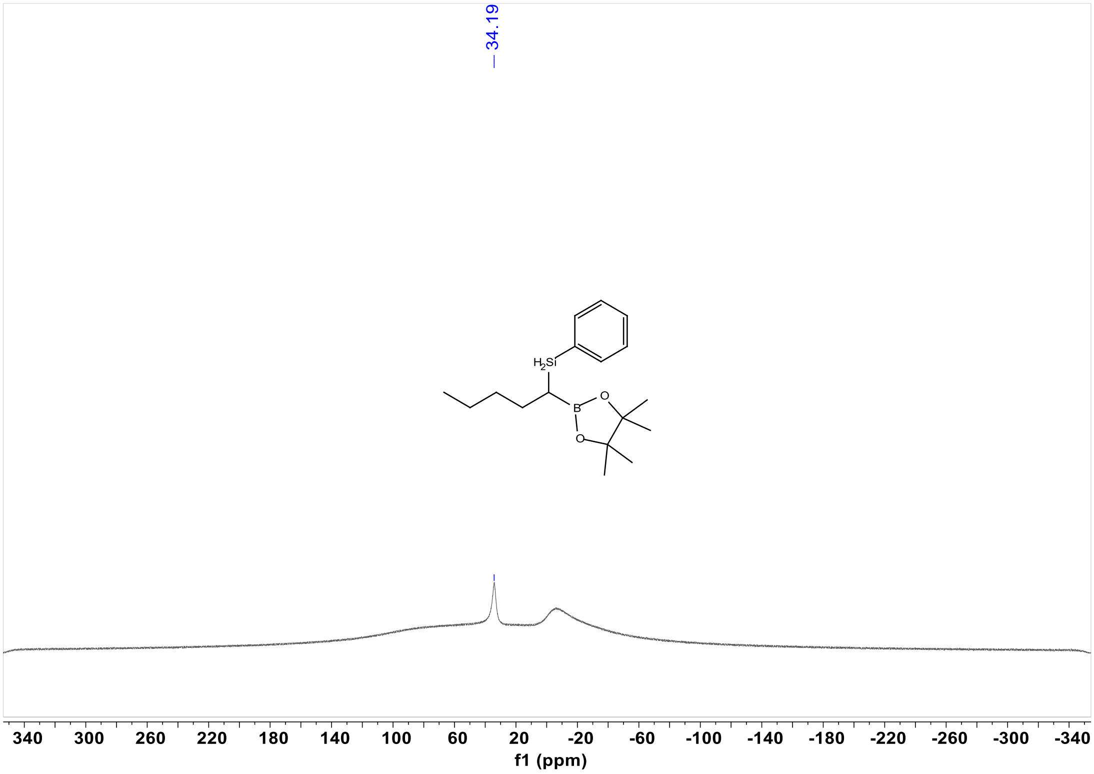

Supplementary Figure 296. ^{11}B NMR spectra for **95**

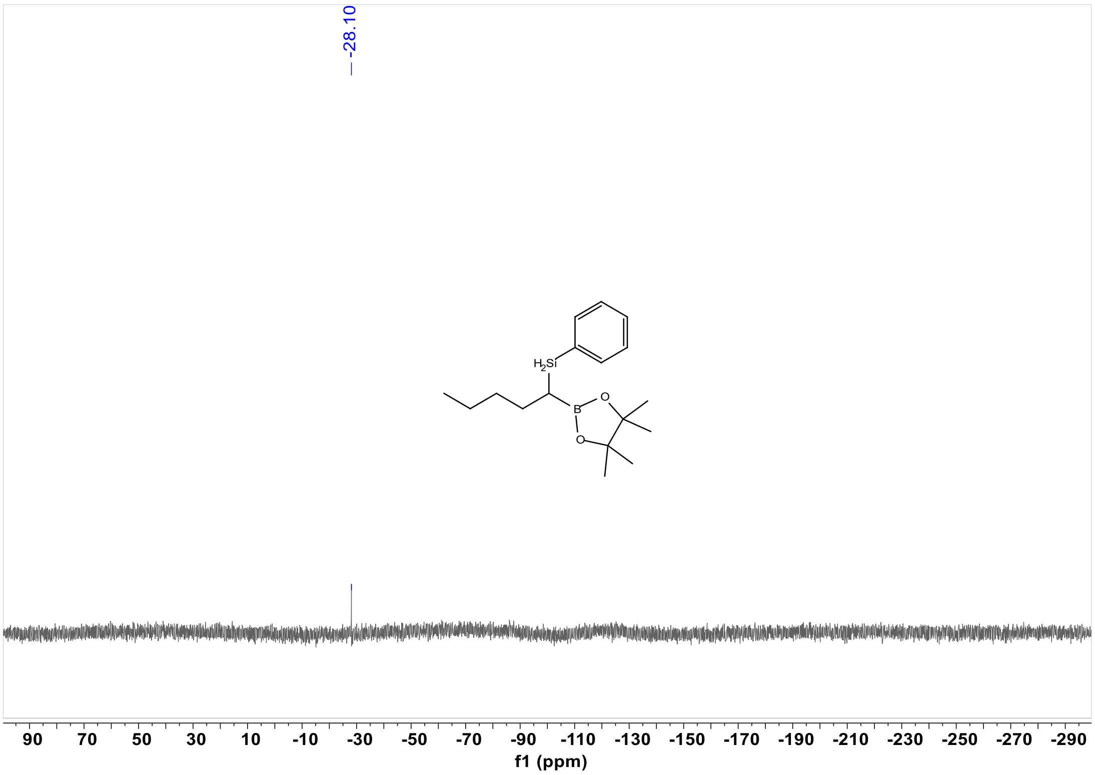

Supplementary Figure 297. ^{29}Si NMR spectra for **95**

Supplementary References

1. Jaladi, A. K., Choi, H. S. & An, D. K. Catalyst-free and solvent-free hydroboration of alkynes. *New J. Chem.* **44**, 13626−13632 (2020).
2. Roy, I. D. *et al.* A second-generation ligand for the enantioselective rhodium-catalyzed addition of arylboronic acids to alkenylazaarenes. *Chem. Commun. (Camb.)* **50**, 2865−2868 (2014).
3. Yoo, K. S., Yoon, C. H. & Jung, K. W. Oxidative palladium(II) catalysis: a highly efficient and chemoselective cross-coupling method for carbon−carbon bond formation under base-free and nitrogenous-ligand conditions. *J. Am. Chem. Soc.* **128**, 16384−16393 (2006).
4. Hellenbrand, T., Hofner, G., Wein, T. & Wanner, K. T. Synthesis of 4-substituted nipecotic acid derivatives and their evaluation as potential GABA uptake inhibitors. *Bioorg. Med. Chem.* **24**, 2072−2096 (2016).
5. Szymaniak, A. A., Zhang, C., Coombs, J. R. & Morken, J. P. Enantioselective synthesis of nonracemic geminal silylboronates by Pt-catalyzed hydrosilylation. *ACS Catal* **8**, 2897−2901 (2018).
6. Trost, B. M. *et al.* Ruthenium-catalysed multicomponent synthesis of the 1,3-dienyl-6-oxy polyketide motif. *Nat. Chem.* **12**, 629−637 (2020).
7. Ardolino, M. J. & Morken, J. P. Congested C–C bonds by Pd-catalyzed enantioselective allyl–allyl cross-coupling, a mechanism-guided solution. *J. Am. Chem. Soc.* **136**, 7092−7100 (2014).
8. Guo, J., Wang, H., Xing, S., Hong, X. & Lu, Z. Cobalt-catalyzed asymmetric synthesis of gem-bis(silyl)alkanes by double hydrosilylation of aliphatic terminal alkynes. *Chem* **5**, 881−895 (2019).
9. Wang, K. *et al.* Selective Manganese-catalyzed oxidation of hydrosilanes to silanols under neutral reaction conditions. *Angew. Chem. Int. Ed. Engl.* **58**, 6380−6384 (2019).
10. Ohmura, T., Sasaki, I., Torigoe, T. & Suginome, M. A (Borylmethyl)silane bearing three hydrolyzable groups on silicon: synthesis via iridium-catalyzed C(sp^3)–H borylation and conversion to functionalized siloxanes. *Organometallics* **35**, 1601−1603 (2016).
11. Šimarek, A., Lamač, M., Horáček, M. & Pinkas, J. $B(C_6F_5)_3$ catalysis accelerates the hydrosilane chlorination by Ph_3CCl. *Appl. Organomet. Chem.* **32**, e4442 (2018).
12. Wang, D., Mück-Lichtenfeld, C., Daniliuc, C. G. & Studer, A. Radical aryl migration from boron to carbon. *J. Am. Chem. Soc.* **143**, 9320−9326 (2021).
13. Chan, J. W., Hoyle, C. E., Lowe, A. B., & Bowman, M. Nucleophile-initiated thiol-Michael reactions: effect of organocatalyst, thiol, and ene. *Macromolecules* **43**, 6381-6388 (2010).
14. Ljungdahl, T., Pettersson, K., Albinsson, B., & Mårtensson, J. Solvent and base dependence of copper-free palladium-catalyzed cross-couplings between terminal alkynes and arylic iodides: development of efficient conditions for the construction of gold (III)/free-base porphyrin dimers. *J. Org. Chem.* 71, 1677-1687 (2006).
15. Corcoran, E. B., McMullen, J. P., Lévesque, F., Wismer, M. K. & Naber, J. R. Photon equivalents as a parameter for scaling photoredox reactions in flow: translation of

photocatalytic C−N cross-coupling from lab scale to multikilogram scale. *Angew. Chem. Int. Ed.* **59**, 11964−11968 (2020).
16. Demas, J. N., Bowman, W. D., Zalewski, E. F. & Velapoldi, R. A. Determination of the quantum yield of the ferrioxalate actinometer with electrically calibrated radiometers. *J. Phys. Chem.* **85**, 2766−2771 (1981).
17. Hatchard, C. G., Parker, C. A. & Bowen, E. J. A new sensitive chemical actinometer - II. Potassium ferrioxalate as a standard chemical actinometer. *Proc. R. Soc. Lond. A* **235**, 518−536 (1956).
18. Gaussian 16, Revision A.01, Frisch, M. J., Trucks, G. W., Schlegel, H. B., Scuseria, G. E., Robb, M. A., Cheeseman, Scalmani, J. R., Barone, G. V., Petersson, G. A., Nakatsuji, H., Li, X., Caricato, M., Marenich, A. V., Bloino, J. B., Janesko, G., Gomperts, R., Mennucci, B. H., Hratchian, P., Ortiz, J. V., Izmaylov, A. F., Sonnenberg, J. L., Williams-Young, D., Ding, F., Lipparini, F., Egidi, F., Peng, J. B., Petrone, A., Henderson, T., Zakrzewski, D. V. G., Gao, J., Rega, N., Zheng, G., Liang, W., Hada, M., Ehara, M., Toyota, K., Fukuda, R., Hasegawa, J., Ishida, M., Nakajima, T., Honda, Y., Kitao, O., Nakai, H., Vreven, T., Throssell, K., Montgomery, J. A. Jr., Peralta, J. E., Ogliaro, F., Bearpark, M. J., Heyd, J. J., Brothers, E. N., Kudin, K. N., Staroverov, V. N., Keith, T. A., Normand, R. J., Raghavachari, K. A., Rendell,P., Burant, J. C., Iyengar, S. S., Tomasi, J., Cossi, M., Millam, J. M., Klene, M., Adamo, C., Cammi, R., Ochterski, J. W., Martin, R. L., Morokuma, K., Farkas, O., Foresman, J. B. & Fox, D. J. Gaussian, Inc., Wallingford CT, (2019).
19. Zhao, Y. & Truhlar, D. G. The M06 suite of density functionals for main group thermochemistry, thermochemical kinetics, noncovalent interactions, excited states, and transition elements: two new functionals and systematic testing of four M06-class functionals and 12 other functionals. *Theor. Chem. Acc.* **120**, 215−241 (2008).
20. Grimme, S., Antony, J., Ehrlich, S. & Krieg, H. A consistent and accurate ab *initio* parametrization of density functional dispersion correction (DFT-D) for the 94 elements H-Pu. *J. Chem. Phys.* **132**, 154104 (2010).
21. Ditchfield, R., Hehre, W. J. & Pople, J. A. Self-consistent molecular ‐ orbital methods. IX. an extended gaussian ‐ type basis for molecular ‐ orbital studies of organic molecules. *J. Chem. Phys.* **54**, 724−728 (1971).
22. Hehre, W. J., Ditchfield, R. & Pople, J. A. Self—consistent molecular orbital methods. XII. further extensions of gaussian—type basis sets for use in molecular orbital studies of organic molecules. *J. Chem. Phys.* **56**, 2257−2261 (1972).
23. Hariharan, P. C. & Pople, J. A. The influence of polarization functions on molecular orbital hydrogenation energies. *Theoretica chimica acta* **28**, 213−222 (1973).
24. Dill, J. D. & Pople, J. A. Self-consistent molecular orbital methods. XV. Extended Gaussian-type basis sets for lithium, beryllium, and boron. *J. Chem. Phys.* **62**, 2921−2923 (1975).
25. Francl, M. M. *et al.* Self-consistent molecular orbital methods. XXIII. A polarization-type basis set for second-row elements. *J. Chem. Phys.* **77**, 3654−3665 (1982).
26. Binkley, J. S., Pople, J. A. & Hehre, W. J. Self-consistent molecular orbital methods. 21. Small split-valence basis sets for first-row elements. *J. Am. Chem. Soc.* **102**, 939−947 (1980).

27. Cammi, R., Mennucci, B. & Tomasi, J. Fast Evaluation of geometries and properties of excited molecules in solution: a tamm-dancoff model with application to 4-dimethylaminobenzonitrile. *J. Phys. Chem. A* **104**, 5631−5637 (2000).
28. Cancès, E., Mennucci, B. & Tomasi, J. A new integral equation formalism for the polarizable continuum model: Theoretical background and applications to isotropic and anisotropic dielectrics. *J. Chem. Phys.* **107**, 3032−3041 (1997).
29. Jr., T. H. D. Gaussian basis sets for use in correlated molecular calculations. I. The atoms boron through neon and hydrogen. *J. Chem. Phys.* **90**, 1007−1023 (1989).
30. Woon, D. E. & Jr., T. H. D. Gaussian basis sets for use in correlated molecular calculations. III. The atoms aluminum through argon. *J. Chem. Phys.* **98**, 1358−1371 (1993).
31. Papajak, E., Zheng, J., Xu, X., Leverentz, H. R. & Truhlar, D. G. Perspectives on basis sets beautiful: seasonal plantings of diffuse basis functions. *J. Chem. Theory Comput.* **7**, 3027−3034 (2011).
32. Lu, T. & Chen, Q. Shermo: A general code for calculating molecular thermochemistry properties. *Comput. Theor. Chem.* **1200**, 113249 (2021).
33. Grimme, S. Supramolecular binding thermodynamics by dispersion-corrected density functional theory. *Chem. Eur. J.* **18**, 9955−9964 (2012).
34. Marcus, R. A. Electron transfer reactions in chemistry: theory and experiment (nobel lecture). *Angew. Chem. Int. Ed. Engl.* **32**, 1111−1121 (1993).
35. Lu, T. & Chen, F. Multiwfn: A multifunctional wavefunction analyzer. *J. Comput. Chem.* **33**, 580−592 (2012).
36. Humphrey, W., Dalke, A. & Schulten, K. VMD: Visual molecular dynamics. *J. Mol. Graph.* **14**, 33−38 (1996).
37. Uoyama, H., Goushi, K., Shizu, K., Nomura, H. & Adachi, C. Highly efficient organic light-emitting diodes from delayed fluorescence. *Nature* **492**, 234−238 (2012).
38. Luo, J. & Zhang, J. Donor–acceptor fluorophores for visible-light-promoted organic synthesis: photoredox/ni dual catalytic C(sp^3)–C(sp^2) cross-coupling. *ACS Catal.* **6**, 873−877 (2016).
39. Phelan, J. P. *et al.* Open-air alkylation reactions in photoredox-catalyzed dna-encoded library synthesis. *J. Am. Chem. Soc.* **141**, 3723−3732 (2019).
40. Zhu, S.-S., Liu, Y., Chen, X.-L., Qu, L.-B. & Yu, B. Polymerization-enhanced photocatalysis for the functionalization of C(sp^3)–H bonds. *ACS Catal.* **12**, 126−134 (2022).
41. Hirshfeld, F. L. Bonded-atom fragments for describing molecular charge densities. *Theor. Chim. Acta.* **44**, 129−138 (1977).
42. Lu, T. & Chen, Q. Independent gradient model based on Hirshfeld partition: A new method for visual study of interactions in chemical systems. *J. Comput. Chem.* **43**, 539−555 (2022).
43. Gao, S., Tang, W., Zhang, M., Wang, C. & Xiao, J. Ru-MACHO-catalyzed highly chemoselective hydrogenation of α-keto esters to 1,2-diols or α-hydroxy esters. *Synlett* **27**, 1748−1752 (2016).
